# Light-controlled disruption of cancer cell dormancy via photoswitchable stress hormone receptor degraders

**DOI:** 10.1073/pnas.2528760123

**Published:** 2026-05-21

**Authors:** Karina M. Freitag, Robin Scheuplein, Chiara Orlacchio, Viola Ansuinelli, Tommaso Fava, Vincent Fischer, Bohan Zhang, Miriam Kretschmer, Mahshid Gazorpak, Erick M. Carreira, Katharina Gapp

**Affiliations:** ^a^Laboratory of Organic Chemistry, Department of Chemistry and Applied Biosciences, ETH Zürich, Zürich 8093, Switzerland; ^b^Laboratory of Epigenetics and Neuroendocrinology, Institute for Neuroscience, Department of Health Science and Technology, ETH Zürich, Zürich 8057, Switzerland; ^c^Neuroscience Center Zürich, ETH Zürich and University of Zürich, Zürich 8057, Switzerland

**Keywords:** photopharmacology, PROTAC, stress hormone signaling, cancer cell dormancy

## Abstract

Cancer cell dormancy is a key contributor to therapy resistance and disease relapse. The glucocorticoid receptor (GR), a major mediator of stress hormone signaling, has emerged as a central regulator of dormancy in non-lymphoid solid tumors, particularly lung cancer. However, systemic GR inhibition or degradation using conventional Proteolysis Targeting Chimeras (PROTACs) risks widespread on-target toxicity due to their constitutive activity. We hypothesized that integrating photoswitchable elements into PROTACs, termed photoPROTACs, would enable wavelength-specific, spatiotemporally precise modulation of GR degradation and dormancy-associated signaling pathways. Here, we synthesized a diverse series of photoPROTACs incorporating photoswitchable arylazotriazole or arylazopyrazole scaffolds, including previously unreported (OEt)_2_- and (NMe_2_)_2_-substituted photoswitches. Arylazopyrazole-based GR photoPROTACs bearing Me_2_- and (OEt)_2_ substituents exhibited near-quantitative photoisomerization (95% *Z-*isomer; 89 to 92% *E-*isomer), no photobleaching, and thermal half-lives in the range of 3 to 12.2 d in dimethyl sulfoxide (DMSO). Among them, KH-5-306 and KH-5-309 induced potent, specific, and reversible GR degradation in their thermodynamically stable *E-*isomeric form at low nanomolar concentrations, with markedly reduced activity in the *Z-*isomeric state. Transcriptomic profiling showed that *E-*KH-5-309 disrupts GR-driven dormancy-associated gene expression programs in a non–small cell lung cancer (NSCLC) model, while the *Z-*isomer remains functionally inert. Our findings establish a framework for the rational design of photoswitchable PROTACs beyond GR and demonstrate their potential to achieve spatiotemporal control of stress hormone receptor signaling, enabling mechanistic insights into GR function and the targeted disruption of cancer cell dormancy.

## Significance

Stress hormone signaling through the glucocorticoid receptor (GR) induces a reversible, drug-tolerant dormancy state in cancer cells. However, systemic GR depletion is not viable due to its essential roles in non-pathological physiology. In this study, we developed light-responsive Proteolysis Targeting Chimeras (photoPROTACs) that enable reversible, wavelength-specific control of GR degradation. PhotoPROTACs featuring arylazopyrazole photoswitches showed potent, isomer-dependent GR degradation and high target specificity at nanomolar concentrations. In addition, transcriptomic profiling in lung cancer cells revealed that only the active isomer disrupts dormancy-associated gene networks, highlighting the potential of photoPROTACs to target GR-driven dormancy exclusively in cancerous tissue.

Dormancy represents a key stalemate in cancer therapy, due to its implication in treatment resistance and disease relapse (1). Among the molecular regulators implicated in this phenomenon, the glucocorticoid receptor (GR), one of the key stress hormone receptors, has emerged as a central driver of dormancy in multiple human non-lymphoid solid cancer types, particularly in non–small cell lung cancer (NSCLC) (2–4). Counterintuitively, GR-activating agents such as dexamethasone (DEX) are routinely co-administered with anticancer therapies to alleviate inflammation and mitigate treatment-related side effects, leveraging GR’s immunosuppressive functions (5–7). Moreover, endogenous glucocorticoid levels are often elevated in patients at the time of diagnosis, further compounding GR activation (8). Together, these factors may inadvertently promote a dormant, treatment-resistant state in tumor cells, underscoring the urgent need for strategies that selectively abrogate GR signaling in cancer cells while preserving its systemic anti-inflammatory role.

We previously developed a potent proteolysis-targeting chimera (PROTAC) capable of inducing GR degradation at the protein level with high specificity and efficacy across species, both in vitro and in vivo (9). However, conventional PROTACs are constitutively active, leading to indiscriminate GR degradation in both malignant and healthy tissues. A promising solution lies in the integration of photopharmacological components to achieve light-dependent activation of PROTACs, so-called photoPROTACs (10). These can be reversibly modulated by specific wavelengths of light, allowing precise control over protein degradation. This light-inducible strategy affords spatiotemporal regulation of PROTAC activity, enhancing therapeutic precision while minimizing off-target effects and systemic toxicity (11).

To date, several classes of photoresponsive elements have been explored in PROTAC design (12). Azobenzene-based linkers, for example, offer reversible switching between *Z-* and *E-* isomers, enabling light-dependent modulation of PROTAC activity (10, 13). Recently, arylazopyrazoles emerged as new photoswitches incorporated into PROTACs due to their near-quantitative photoisomerization and tunable thermal half-lives (14, 15). Other approaches have relied on photocaging groups such as diethylaminocoumarin or nitrobenzyl derivatives to render the PROTAC inactive until light exposure irreversibly removes the caging group, thereby restoring activity (16, 17). Despite these advances, the chemical space of photoresponsive PROTACs remains underexplored.

In this study, we report three classes of photoPROTACs featuring arylazotriazole- or arylazopyrazole-based photoswitches that were designed for light-inducible GR degradation. Comprehensive photophysical characterization revealed that arylazopyrazole-based photoPROTACs exhibited superior photoisomerization, high photostability, and thermal half-lives ranging from 3 to 12.2 d in dimethyl sulfoxide (DMSO). In vitro screening identified two lead compounds, KH-5-306 and KH-5-309, which selectively degrade GR in their thermodynamically stable *E-*isomeric form, with minimal activity in their photoactivated *Z-*isomeric state. Both photoPROTACs achieve potent GR depletion at nanomolar concentrations, sparing structurally related proteins and demonstrating high target specificity. Transcriptomic profiling by bulk RNA sequencing and qPCR revealed that the *E-*isomer of KH-5-309 effectively counteracts DEX-induced dormancy-associated gene expression programs in lung cancer, while the *Z-*isomer remained functionally inert. Collectively, these findings underscore the utility of arylazopyrazole-based photoswitches as a versatile platform for modulating PROTAC activity and provide a foundation for the dynamic, light-controlled regulation of GR signaling in cancer.

## Results

### Design Rationale for photoPROTACs.

Building on our previously reported GR-targeting PROTAC KH-103, we aimed to develop a light-controllable degrader by incorporating a photoswitchable linker. DEX and lenalidomide were employed as ligands for GR and cereblon (CRBN), respectively. Initial experiments revealed that GR degradation is highly sensitive to linker length, with activity confined to a narrow but tunable range (*SI Appendix*, Fig. S1). To exploit this, the *E-*isomeric forms of the photoswitchable linkers were engineered to match the active linker length range, while photoisomerization to the *Z-*isomer was expected to contract the linker to an inactive conformation, thus enabling reversible, light-dependent control of GR degradation.

**Fig. 1.**
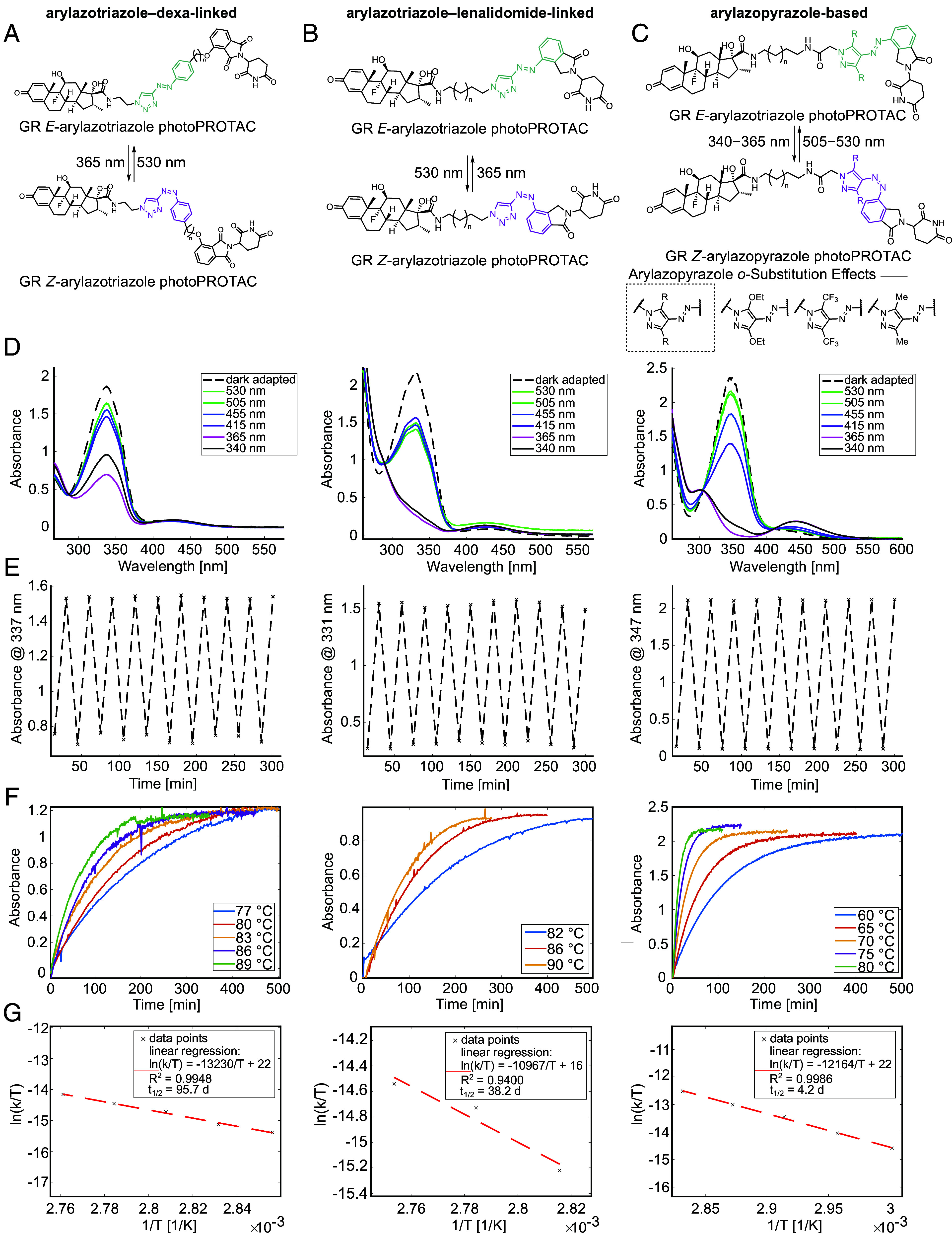
Design and characterization of photoswitchable PROTACs (photoPROTACs) targeting GR. (*A*–*C*) Chemical structures of the *E*- and *Z*- isomers of photoPROTACs incorporating photoswitchable linkers at different positions: (*A*) arylazotriazole–dexa-linked, (*B*) arylazotriazole–lenalidomide-linked, and (*C*) arylazopyrazole-based with 3,5-bis functionalized pyrazoles near the CRBN-binding site. (D) UV-vis absorption spectra of representative photoPROTACs before and after irradiation (30 min at 340 nm or 20 min at 365 to 530 nm). Spectra were recorded at 100 μM in DMSO–water (2:1) for (*A* and *B*) and in DMSO for (*C*), with π–π* λ_max_ values of 337 nm (*A*), 331 nm (*B*), and 347 nm (*C*). (*E*) Photostability profiles over 300 min, measured at the respective λ_max_ following alternating irradiation (15 min) at 365 nm and 455 nm (*A*), 415 nm (*B*), or 530 nm (*C*). (*F*) Thermal relaxation kinetics at the respective λ_max_ after 365 nm irradiation (20 min), monitored over 77 to 89 °C (*A*), 82 to 90 °C (*B*), and 60 to 80 °C (*C*). (*G*) Eyring plots for thermal relaxation. For (*B*), instability at elevated temperatures limited the number of usable data points. Representative plots for (*C*) correspond to the Me_2_-substituted photoPROTAC. See *SI Appendix*, Figs. S53–S55 for analysis of (OEt)_2_–and (CF_3_)_2_-substituted analogs. n refers to linker length. For the arylazotriazole–dexa-linked photoPROTAC (panel *A*), n = 2 (KH-5-169); for the arylazotriazole–lenalidomide-linked photoPROTAC (panel *B*), n = 9 (KH-5-226); and for the Me_2_-substituted arylazopyrazole-based photoPROTAC (panel *C*), n = 1 (KH-5-298).

Based on this framework, we synthesized three groups of GR-targeting photoPROTACs incorporating either arylazotriazole or arylazopyrazole photoswitches (*SI Appendix*, Fig. S2). Representative *E-* and *Z-*isomer structures for each group are shown in Fig. 1 *A*–*C*, with the photoswitchable moiety highlighted.

The first group incorporated an arylazotriazole photoswitch conjugated to the GR ligand (Fig. 1*A* and *SI Appendix*, Figs. S3–S5; arylazotriazole–dexa-linked photoPROTACs). Arylazotriazole scaffolds, first developed in the Carreira group, offer robust bistability, high photoisomerization yields, and allow for the facile assembly of PROTAC architectures by a desilylative CuAAC reaction (10). In the second group, the linker was relocated to the lenalidomide exit vector (Fig. 1*B* and *SI Appendix*, Figs. S6–S8; arylazotriazole–lenalidomide-linked photoPROTACs). Inspired by the beneficial effects observed when an azobenzene photoswitch was fused to the CRBN-recruiting element in prior work by Trauner and colleagues (18), this design aimed to minimize potential interference with GR binding, while preserving the validated topology of GR PROTAC KH-103, which favored alkyl linkers over PEG-based spacers. The third group employed arylazopyrazole-based linkers with systematically varied substituents to tune photophysical properties and probe structure–activity relationships (Fig. 1*C* and *SI Appendix*, Figs. S9–S18; arylazopyrazole-based). Specifically, we synthesized and characterized Me_2_-, (OEt)_2_-, (CF_3_)_2_-, and (NMe_2_)_2_-substituted arylazopyrazoles. These functional groups were introduced to enhance isomer-specific differentiation by inducing unfavorable interactions between the photoactive moiety and the CRBN binding pocket, which are more pronounced in one isomeric state than the other, thereby amplifying the functional contrast between *E-* and *Z-*isomers. Across all three design groups, various linker lengths were tested to explore their impact on activity.

### Arylazopyrazole Photoswitches.

We synthesized and characterized four arylazopyrazole scaffolds bearing Me_2_, (OEt)_2_, (CF_3_)_2_, and (NMe_2_)_2_ substituents. The arylazopyrazoles were evaluated for their photoisomerization and thermal relaxation behavior across multiple media (DMSO, MeCN, MeOH, and DMSO–Buffer). In DMSO, Me_2_ and (OEt)_2_ derivatives exhibited well-separated *E/Z* n–π* absorbance bands (*SI Appendix*, Fig. S19). Both also retained switching capability in aqueous buffer, highlighting their robustness for biological applications (*SI Appendix*, Fig. S20). In contrast, (CF_3_)_2_ showed reduced band separation and lower switching efficiency, while (NMe_2_)_2_ displayed broadened n–π* features and lost any detectable photoswitching activity in MeOH and buffer, likely due to protonation of the azo unit, consistent with prior observations for *ortho*- and *para*-dialkylamino-substituted azobenzenes (19, 20) (*SI Appendix*, Figs. S19–S22). Thermal relaxation trends confirmed that (NMe_2_)_2_ was the least stable, whereas Me_2_ and (OEt)_2_ offered robust bistability (*SI Appendix*, Fig. S23). Despite successful synthesis, (NMe_2_)_2_ derivatives proved unsuitable for biological use due to their poor bistability in aqueous environments. Consequently, we focused on Me_2_-, (OEt)_2_-, and (CF_3_)_2_-substituted photoswitches for arylazopyrazole-based GR photoPROTAC assembly.

### Photophysical Properties of the photoPROTACs.

To evaluate the suitability of our photoPROTACs for biological applications requiring precise light control and sustained photostability, we systematically characterized the photophysical properties of a representative compound from each photoPROTAC group.

Arylazotriazole–dexa-linked photoPROTACs showed limited *E*- to *Z-*isomer switching efficiency in DMSO–water mixtures (2:1, 100 μM), reaching a photostationary state (PSS) of 49% *Z-*isomer upon irradiation at 415 nm and 70% *E-*isomer at 530 nm (Fig. 1 *D*, *Left* and *SI Appendix*, Figs. S24–S30). Despite this, they exhibited excellent photostability over multiple irradiation cycles (365/455 nm) and long *Z-*isomer half-lives on the order of weeks (Fig. 1 *E*–*G*, *Left*). In contrast, arylazotriazole–lenalidomide-linked photoPROTACs demonstrated superior switching behavior in DMSO–water mixtures (2:1, 100 μM), achieving PSS values of 68% *Z-*isomer (365 nm) and 73% *E-*isomer (505 nm) (Fig. 1 *D*, *Center* and *SI Appendix*, Figs. S31–S37). These compounds also showed no photobleaching and remained bistable for several weeks (Fig. 1 *E*–*G*, *Center*).

Both Me_2_- and (OEt)_2_-substituted arylazopyrazole variants exhibited remarkable photoswitching performance in DMSO, reaching 95% *Z*-isomer upon irradiation at 365 to 370 nm and 89 to 92% *E*-isomer at 525 to 530 nm (Fig. 1 *D*, *Right* and *SI Appendix*, Figs. S38–S44 and S52). No photobleaching was observed, and switching was fully reversible (Fig. 1 *E*, *Right* and *SI Appendix*, Figs. S53–S55). While both variants showed similar photoisomerization yields, the (OEt)_2_-substituted compounds displayed prolonged *Z*-isomer thermal half-lives of 9.4 to 12.2 d at 25 °C in DMSO, compared to 3.0 to 4.2 d for the Me_2_ analogs (Fig. 1 *F* and *G*, *Right* and *SI Appendix*, Figs. S53–S55). In contrast, (CF_3_)_2_-substituted photoPROTACs displayed slightly reduced photoisomerization yields in DMSO, achieving 81.5% *Z*-isomer at 340 nm and 71.1% *E*-isomer at 530 nm (*SI Appendix*, Figs. S45–S51). However, they exhibited considerable thermal stability in DMSO, with negligible relaxation at the physiologically relevant temperature of 37 °C over 8 to 16 h (*SI Appendix*, Fig. S56).

### Evaluation of GR Degradation by photoPROTACs in HEK293T Cells.

All photoPROTAC groups were assessed for their ability to degrade GR in HEK293T cells after 18 h of incubation in their photoactive *E-*isomer, using immunoblotting. Prior to treatment, compounds were irradiated at their respective activation wavelengths to generate the desired isomeric form and were subsequently maintained in the dark throughout the incubation period to prevent light-induced switching.

The arylazotriazole–dexa-linked photoPROTACs failed to induce GR degradation and were therefore discontinued (*SI Appendix*, Fig. S57).

In the arylazotriazole–lenalidomide-linked photoPROTAC series, reproducible GR degradation was only achieved at elevated concentrations (1 μM) and no significant difference in GR degradation was observed between the *E-* and *Z-*isomers (*SI Appendix*, Figs. S58–S59). Additionally, the compounds exhibited poor stability in DMSO, limiting their utility for extended in vitro studies.

**Fig. 2.**
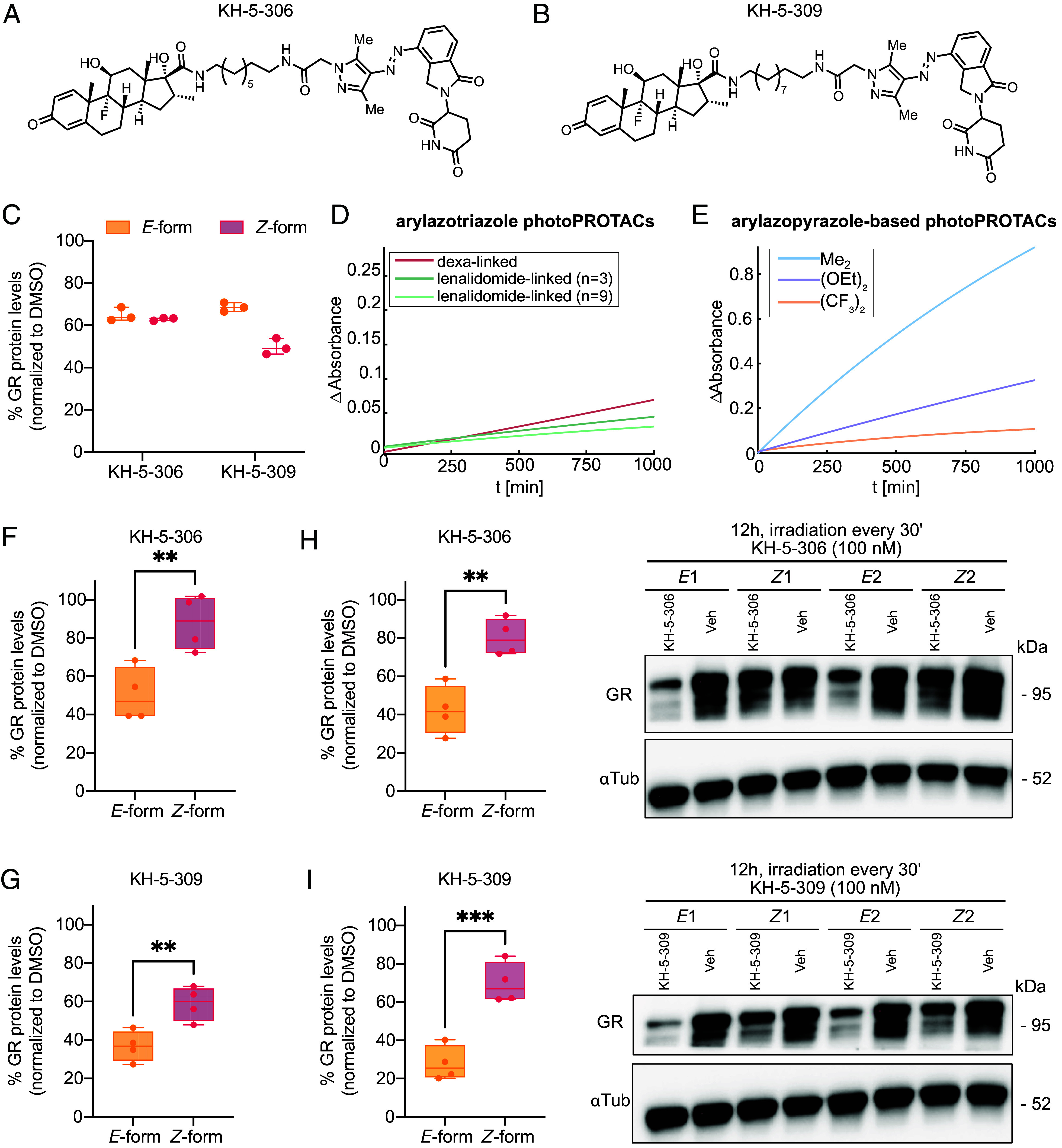
Isomer-specific GR degradation and thermal relaxation behavior of arylazopyrazole-based photoPROTACs. (*A* and *B*) Chemical structure of KH-5-306 (*A*) and KH-5-309 (*B*). (*C*) Quantification of GR levels by immunoblotting in HEK293T cells treated for 18 h with 100 nM of KH-5-306 or KH-5-309 in their *E-* or *Z-* isomeric form. (*D* and *E*) Thermal relaxation kinetics of photoPROTACs over 1,000 min at 37 °C in DMSO following 365 nm irradiation for 20 min. In panel *D*, the red line corresponds to the arylazotriazole–dexa-linked photoPROTAC with n = 2 (KH-5-169; λ_max_ = 337 nm), and the green lines represent arylazotriazole–lenalidomide-linked photoPROTACs with n = 3 (KH-5-210; λ_max_ = 331 nm) and n = 9 (KH-5-226; λ_max_ = 331 nm). In panel *E*, the light blue line corresponds to the Me_2_-substituted arylazopyrazole photoPROTAC with n = 1 (KH-5-298; λ_max_ = 347 nm), the purple line to the (OEt)_2_-substituted analog with n = 1 (KH-5-450; λ_max_ = 350 nm), and the yellow line to the (CF_3_)_2_-substituted analog with n = 1(KH-5-327; λ_max_ = 330 nm). (*F* and *G*) Quantification of GR levels in HEK293T cells treated with 100 nM KH-5-306 (*F*) or KH-5-309 (*G*) in their *E-* or *Z-*form for 12 h, with 365 nm irradiation every hour for 30 s. Unpaired two-tailed *t* tests revealed significant differences between *E-*form and *Z-*form for KH-5-306 [t(6) = 3.759, *P* < 0.01] and KH-5-309 [t(6) = 3.741, *P* < 0.01]. (*H* and I) Representative immunoblot and quantification of GR levels in HEK293T cells treated for 12 h with 100 nM KH-5-306 (*H*) or KH-5-309 (*I*) in either the *E-* or *Z-*form, with 365 nm irradiation every 30 min for 30 s. Unpaired two-tailed t-tests showed significant differences between *E-*form and *Z-*form for KH-5-306 [t(6) = 4.753, *P* < 0.01] and KH-5-309 [t(6) = 6.042, *P* < 0.001]. Veh: vehicle control. Box-and-whisker plots show the median (line) and interquartile range (25th to 75th percentile; box), with whiskers extending to the minimum and maximum values. All individual data points are shown.

The Me_2_-substituted arylazopyrazole-based photoPROTACs, particularly KH-5-306 and KH-5-309, demonstrated robust GR degradation at 100 nM (Fig. 2 *A*–*C*). However, no difference in activity was observed between the *E-* and *Z-*isomers. Similarly, the (CF_3_)_2_- and (OEt)_2_-substituted arylazopyrazole photoPROTACs also induced GR degradation, with both *E-* and *Z-*isomers remaining active, even when incubation times were shortened to 12 h to mitigate thermal relaxation of the *Z-*isomer back to the *E-*isomer (*SI Appendix*, Fig. S60).

Given the absence of isomer-selective degradation, we hypothesized that the presumed bistability of the photoisomers may be compromised under physiological conditions. To compare the substituent-dependent thermal relaxation of the arylazopyrazole photoPROTACs at a physiologically relevant temperature, we monitored relaxation kinetics at 37 °C in DMSO, as attempts in DMSO–buffer were hindered by solvent evaporation. Under these conditions, the (OEt)_2_- and Me_2_-substituted arylazopyrazole photoPROTACs displayed pronounced thermal relaxation, whereas the (CF_3_)_2_-arylazopyrazole photoPROTACs retained excellent bistability, with negligible changes in the *E/Z* ratio after 1,000 min (Fig. 2*E* and *SI Appendix*, Fig. S62). Similarly, the arylazotriazole–lenalidomide photoPROTACs remained highly bistable in DMSO–water at 37 °C (Fig. 2*D* and *SI Appendix*, Fig. S61).

To isolate the impact of the aqueous environment, we leveraged the thermal relaxation data obtained for the photoswitches in DMSO–buffer mixtures at 25 °C. In DMSO–buffer, the (OEt)_2_-arylazopyrazole photoswitch exhibited more rapid thermal relaxation compared to the Me_2_-arylazopyrazole photoswitch, reversing the trend observed in DMSO. This solvent-dependent inversion indicates that aqueous environments accelerate relaxation of the (OEt)_2_-arylazopyrazole photoswitch more strongly than expected based on its Eyring-derived half-life of 9.4 to 12.2 d in DMSO (*SI Appendix*, Fig. S23). Notably, the (CF_3_)_2_-substituted photoPROTACs were discontinued due to altered UV–vis absorption spectra in DMSO–buffer mixtures, indicating impaired photoswitching behavior and limiting their applicability in biological systems (*SI Appendix*, Fig. S63).

To counteract thermal relaxation and preserve the *Z-*isomer during cellular assays, cells treated with the *Z-*isomer were exposed to 30s pulses of 365 nm light every hour (25 mW) during the 12 h incubation period. Under these conditions, both KH-5-306 and KH-5-309 displayed isomer-dependent GR degradation (Fig. 2 *F* and *G*), with more than 60% GR degradation observed in the KH-5-309 *E-*isomer. In contrast, (OEt)_2_-arylazopyrazole photoPROTACs remained insensitive to isomeric state under this irradiation regime (*SI Appendix*, Fig. S64).

To further stabilize the *Z-*isomer, the irradiation frequency was increased to every 30 min over a 12 h period (Fig. 2 *H* and *I*). Under these conditions, the isomer-specific degradation of GR by KH-5-309 became even more pronounced, with GR levels differing by an additional 19% between the *E-* and *Z-*isomers compared to the hourly irradiation protocol (*SI Appendix*, Fig. S65). Overall, the average difference in GR levels between the two isomeric states of KH-5-309 reached 40%, underscoring the importance of sustained photoisomer stabilization for achieving robust isoform-selective activity (Fig. 2*G*).

### Proteasome Dependence and Target Specificity of photoPROTAC-Induced GR Degradation.

**Fig. 3.**
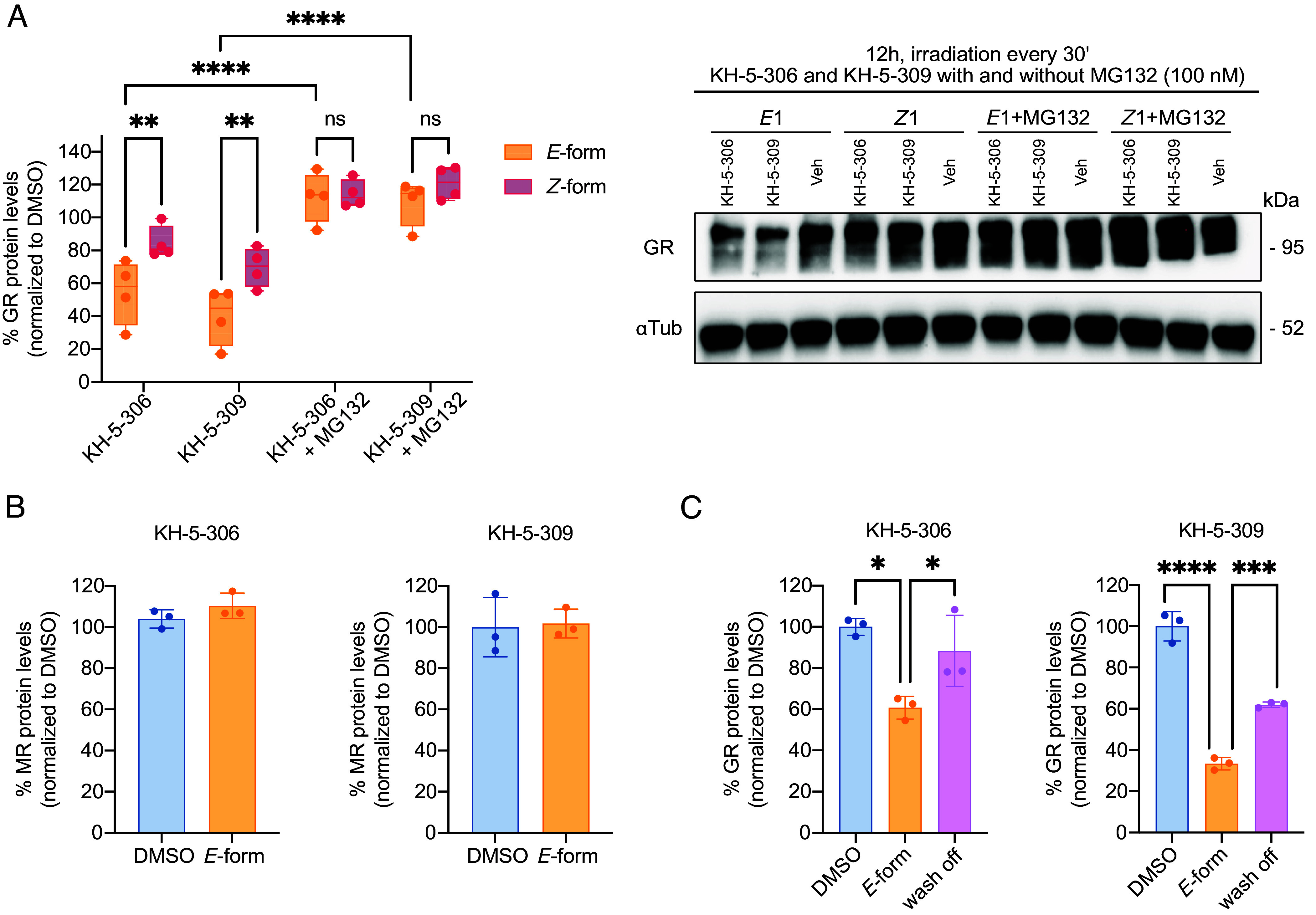
Proteasome-dependent, selective, and reversible effects of arylazopyrazole-based photoPROTACs. (*A*) Representative immunoblot and quantification of GR levels in HEK293T cells treated for 12 h with 100 nM KH-5-306 or KH-5-309 in their *E-* or *Z-* isomeric form, with or without co-treatment with the proteasome inhibitor MG132. Two-way ANOVA revealed significant main effects for treatment [F(3, 24) = 39.39, *P* < 0.0001] and isomeric-form [F(1, 24) = 14.13, *P* = 0.001], but no significant interaction [F(3,24) = 2.013, *P* = 0.1389]. Follow-up Tukey’s multiple comparisons test showed significant differences between *E-*form and *Z-*form for KH-5-306 (*P* = 0.0051) and KH-5-309 (*P* = 0.0057), but not for KH-5-306 + MG132 (*P* = 0.8390) or KH-5-309 + MG132 (*P* = 0.2423). Additionally, significant differences were observed between *E-*form KH-5-306 and KH-5-306 + MG132 (*P* < 0.0001), and between *E-*form KH-5-309 and KH-5-309 + MG132 (*P* < 0.0001). (*B*) Quantification of MR levels in HEK293T cells following 12 h treatment with 500 nM KH-5-306 or KH-5-309. (*C*) Quantification of GR levels following 12 h treatment of HEK293T cells with 500 nM KH-5-306 or KH-5-309, followed by compound washout and media replacement for an additional 12 h recovery period. Ordinary one-way ANOVA revealed significant differences for KH-5-306 [F(2,6) = 10.50, *P* = 0.0110] and KH-5-309 [F(2,6) = 162.6, *P* < 0.0001]. Post hoc Tukey’s multiple comparisons showed significant differences between DMSO and *E-*form KH-5-306 (*P* = 0.0101), and between *E-*form KH-5-306 and washout (*P* = 0.0459). Similarly, significant differences were observed between DMSO and *E-*form KH-5-309 (*P* < 0.0001), and between *E-*form KH-5-309 and washout (*P* = 0.0006). Veh: vehicle control. Box-and-whisker plots (panel *A*) show the median (line) and interquartile range (25th to 75th percentile; box), with whiskers extending to the minimum and maximum values. Bar graphs (panels *B* and *C*) show mean ± SD. All individual data points are shown.

To confirm that the photoPROTAC-induced GR degradation was indeed mediated via the ubiquitin–proteasome system, co-incubation experiments were performed with the proteasome inhibitor MG132. Immunoblot analysis revealed that GR degradation was completely abrogated in the presence of MG132 for both isomers of KH-5-306 and KH-5-309, confirming that the observed GR depletion is proteasome-dependent (Fig. 3*A*). Furthermore, no degradation of the mineralocorticoid receptor (MR), the closest structural paralog of GR, was detected under identical conditions, even when compounds were applied at 500 nM, demonstrating the target specificity of the photoPROTACs (Fig. 3*B*). Finally, the GR degradation induced by the *E-*isomer was shown to be reversible (Fig. 3*C*). Washing off the *E-*isomer after 12 h initiated recovery of GR protein levels by 24 h, indicating that the effect is not permanent and depends on the continued presence of the active isomer.

### Isomer-Specific Reversal of DEX-Induced Dormancy in A549 Cells by KH-5-309.

Having confirmed the light-dependent activity of arylazopyrazole-based photoPROTACs, we next assessed their functional efficacy in a disease-relevant model. While we collected data for both compounds, subsequent analyses focused on KH-5-309 due to its superior GR degradation capacity.

**Fig. 4.**
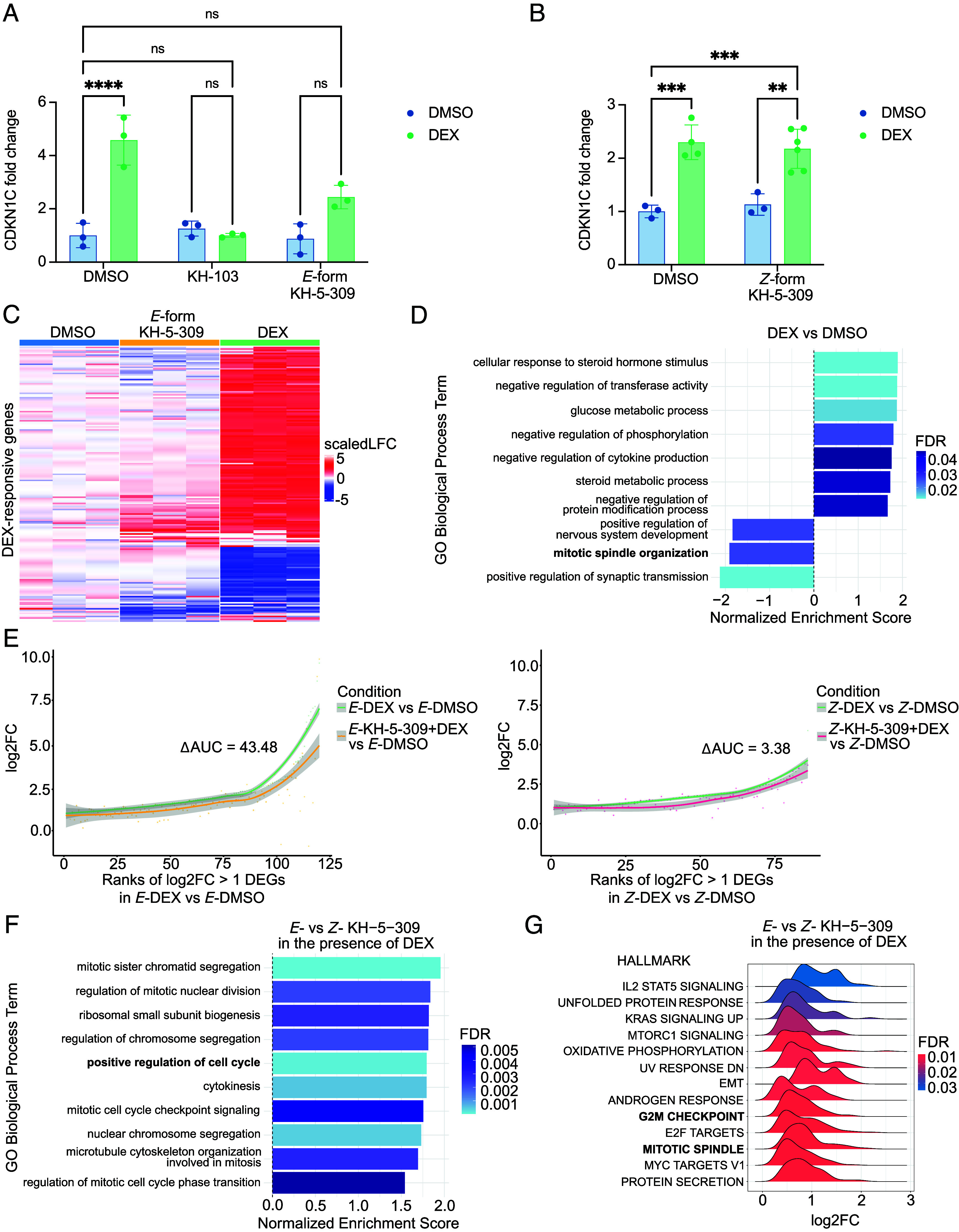
Isomer-specific reversal of DEX-induced dormancy in A549 cells by KH-5 309. (*A*) Relative expression of CDKN1C measured by RT-qPCR following treatment with KH-103, *E-*form KH-5-309, DEX, or combinations. Two-way ANOVA revealed significant main effects of DEX [F(1,12) = 42.59, *P* < 0.0001] and treatment [F(2,12) = 15.33, *P* = 0.0005], as well as a significant interaction [F(2,12) = 19.65, *P* = 0.0002]. Sidak’s post hoc test showed a significant difference between DMSO and DEX (*P* < 0.0001), but no significant differences between DMSO and DEX + KH-103 (*P* > 0.9999), KH-103 and KH-103 + DEX (*P* > 0.9999), DMSO and DEX + *E-*form KH-5-309 (*P* = 0.0863), or *E-*form KH-5-309 and DEX + *E-*form KH-5-309 (*P* = 0.0516). (*B*) RT-qPCR analysis of CDKN1C expression following treatment with *Z-*isomer of KH-5-309, DEX, or their combination. Two-way ANOVA revealed significant main effects of DEX [F(1,12) = 55.28, *P* < 0.0001], but no significant effect of the *Z-*form KH-5-309 [F(1,12) = 0.0007, *P* = 0.9795], and no significant interaction [F(1,12) = 0.6456, *P* = 0.4373]. Sidak’s post hoc test showed a significant difference between DMSO and DEX (*P* = 0.0007), DMSO and *Z-*form KH-5-309 + DEX (*P* = 0.0008), and between *Z-*form KH-5-309 and *Z-*form KH-5-309 + DEX (*P* = 0.0023). (*C*) Heatmap showing expression levels of DEX-responsive genes in A549 cells treated for 12 h with either DMSO or the *E-*isomer of KH-5-309. During the final 2 h of each treatment, cells were exposed to either DEX or additional DMSO. This panel compares gene expression profiles across the following conditions: DMSO (12 h), *E-*form KH-5-309 (12 h), and DEX (12 h DMSO with DEX added during the final 2 h). (*D*) Gene Ontology (GO) Biological Process enrichment analysis for non-irradiated samples highlighting biological processes enriched in response to DEX treatment compared to DMSO control. The bar graph displays normalized enrichment scores (NES) for significantly enriched GO terms, with color indicating the false discovery rate (FDR). (*E*) Curve plot showing log2FC of differentially expressed genes (DEGs) between DEX and DMSO, and in samples pre-treated with either the *E-* (*Left*) or *Z-* (*Right*) isomer of KH-5-309 for 12 h, with DEX added during the final 2 h. (*F*) GO Biological Process enrichment comparing samples treated with *E-*isomer + DEX vs. *Z-*isomer + DEX. (*G*) Gene Set Enrichment Analysis (GSEA) using Hallmark gene sets from MSigDB, highlighting transcriptional differences between *E-*isomer + DEX and *Z-*isomer + DEX treatments. Log2FC distributions are shown for significantly enriched gene sets, with color indicating FDR. EMT: epithelial–mesenchymal transition. Bar graphs show mean ± SD with all individual data points overlaid.

Previous work by Prekovic et al. demonstrated that DEX induces a drug-tolerant, dormancy-like state in A549 cells, a human lung adenocarcinoma cell line (2). This state is characterized by elevated expression of the cyclin-dependent kinase inhibitor CDKN1C (p57). Importantly, CDKN1C was shown to be necessary for the establishment of this DEX-induced cell dormancy. In our experimental setup, we similarly observed robust CDKN1C upregulation following 2 h of DEX exposure, confirming that our treatment regime induces a comparable dormancy-like state in A549 cells (Fig. 4*A*).

To evaluate whether GR degradation could reverse the dormant state, we used our previously developed potent GR degrader KH-103 as a positive control. Following validation of GR depletion in A549 cells (*SI Appendix*, Fig. S66), KH-103 effectively suppressed DEX-induced CDKN1C expression, as confirmed by RT-qPCR (Fig. 4*A*) and our previously published RNA-seq data (9) (*SI Appendix*, Fig. S67).

We then examined the isomer-specific activity of KH-5-309 in the same cellular context. Consistent with its light-dependent mechanism, KH-5-309 robustly degraded GR in its *E-*isomeric form, whereas the *Z-*isomer exhibited a statistically significant reduction in activity (*SI Appendix*, Fig. S68).

To test functional efficacy, A549 cells were treated with either isomer for 12 h, with DEX or DMSO added during the final 2 h. RT-qPCR analysis of CDKN1C expression revealed that the *E-*isomer of KH-5-309 reversed the DEX-induced increase to baseline levels (Fig. 4*A*), whereas the *Z-*isomer had no effect (Fig. 4*B*).

To exclude confounding effects of the intermittent irradiation regimen used to maintain *Z*-isomer enrichment, we performed multiple orthogonal controls. First, flow cytometry-based live-dead imaging revealed no irradiation induced cytotoxicity under the 30 min light pulse protocol used in all assays (*SI Appendix*, Fig. S69). Second, bulk RNA-seq analysis of irradiated vs. non-irradiated DMSO-treated A549 cells identified 68 significantly affected genes (*SI Appendix*, Fig. S70). Overrepresentation analysis of these genes identified only two enriched GO terms (estrogen metabolic process and SMAD signaling), neither of which relate to UV stress or dormancy (*SI Appendix*, Fig. S71). Third, complementary enrichment analysis using GO terms and hallmark pathways, detected no pathway-level changes associated with irradiation or dormancy (*SI Appendix*, Fig. S72). Finally, to rule out activation of UV-stress pathways at the protein level, we performed label-free proteomics. Comparison of irradiated vs. non-irradiated DMSO-treated cells revealed only two significantly downregulated proteins (CCN2 and DESI1). While CCN2 has been implicated in cell-cycle–associated differentiation, DESI1 has no known role in proliferation and importantly GSEA showed no significant enrichment of pathways related to UV-stress response, stress signaling, or cell-cycle regulation. These data demonstrate that the detected protein-level changes are limited in scope and do not reflect the activation of broader phenotypic stress programs (*SI Appendix*, Fig. S73). Together, these cellular, transcriptomic, and proteomic data confirm that the irradiation protocol does not perturb the underlying biological pathways studied here.

Having validated the light regimen, we further characterized the pharmacological profile of the isomers. Treatment with the *E-*isomer alone altered expression of only 14 DEX-responsive genes (Fig. 4*C*), suggesting a passive mode of action. This aligns with prior observations using KH-103 and contrasts with the partial agonistic activity of the clinically available GR inhibitor Mifepristone (9). Rank-order analyses further showed that *E*-KH-5-309 and KH-103 diverge in their transcriptional signatures, demonstrating that the two degraders influence distinct sets of partial-agonism genes (*SI Appendix*, Fig. S74). The *Z*-isomer affected none of these gene sets, consistent with its inactivity. In addition, a log2FC comparison of the *E*-KH-5-309 vs. DMSO and *E*- vs. *Z*-KH-5-309 DEGs revealed a near-diagonal relationship (Pearson = 0.80), underscoring that the *Z*-isomer does not introduce nonspecific transcriptional changes and functions as a clean, biologically inert control (*SI Appendix*, Fig. S75).

As expected, DEX treatment induced robust transcriptional changes (Fig. 4*C*). GO enrichment analysis revealed significant downregulation of proliferation-associated gene programs, including mitotic spindle organization, consistent with dormancy-associated cell cycle arrest (2) (Fig. 4*D*). To quantify global gene expression differences, we used an area under the curve (AUC) metric. The *E-*isomer of KH-5-309 strongly counteracted DEX-upregulated genes, with a ΔAUC of 43.48 between DEX-only and DEX+*E-*KH-5-309 conditions (Fig. 4*E*). In contrast, the *Z-*isomer showed minimal effect (ΔAUC = 3.38). Consistent with these observations, a Wilcoxon signed-rank test confirmed a statistically significant difference in gene expression between DEX-only and DEX + *E-*KH-5-309 (*P* < 0.0001), but not between DEX-only and DEX + *Z-*KH-5-309 (*P* > 0.05).

Further gene expression analysis using an interaction model comparing the differential effects of *E-* and *Z-*isomers in the presence of DEX revealed that treatment with *E-*KH-5-309 restored multiple proliferation-associated GO terms, including positive regulation of the cell cycle, suggesting an isomer-specific reversal of DEX-induced dormancy (Fig. 4*F*). Supporting this, direct comparison between DEX + *E-*KH-5-309 and DEX-only conditions confirmed that these effects were specifically driven by the *E-*isomer (*SI Appendix*, Fig. S76). Furthermore, enrichment analysis using the Hallmark Gene Set Collection from MSigDB (21), applied to the same interaction model, revealed significant upregulation of cell cycle-related pathways, most notably the G2M checkpoint and mitotic spindle, again specifically in the *E-*isomer condition (Fig. 4*G* and *SI Appendix*, Fig. S77).

Together, these findings demonstrate that photoPROTAC-dependent GR degradation via KH-5-309 can effectively reverse DEX-induced dormancy-associated transcriptional programs in A549 cells, with functional activity restricted to the *E-*isomer.

## Discussion

This study presents the development and characterization of light-inducible GR-targeting photoPROTACs, enabling conditional and reversible GR degradation. By systematically varying photoPROTAC architecture, we identified critical structural features that govern GR degradation efficiency and isomer specificity. Notably, the Me_2_- substituted arylazopyrazole-based photoPROTACs demonstrated superior photophysical properties, including near quantitative photoisomerization, no photobleaching, and thermal half-lives in the range of 3.0 to 4.2 d. Lead compounds KH-5-306 and KH-5-309 demonstrated reversible and isomer-selective GR degradation in vitro under intermittent irradiation. Importantly, KH-5-309 effectively disrupted DEX-induced dormancy in a NSCLC model in an isomer-specific manner, underscoring its potential for therapeutic intervention in GR-driven cancer phenotypes.

Our findings confirm the critical role of linker length, photoswitch placement, and functionalization to steer isomer-specific activity of GR-targeting photoPROTACs. First, we validated that linker-induced steric hindrance can be leveraged for the design of light-controlled GR degradation, as previously shown for α-BRD2 targeting photoPROTAC (10).

Second, the moderate photoisomerization yields and inactivity of the arylazotriazole–dexa-linked photoPROTACs proved that the positioning of the photoswitchable linker affects the thermal bistability and GR degradation efficiency. Shifting the arylazotriazole linker to the lenalidomide exit vector improved PSS rates and GR degradation, likely by preventing interferences with the GR binding interface.

Third, 3,5-difunctionalized arylazopyrazole photoswitches exhibited near-quantitative switching and high fatigue resistance, as demonstrated by their ability to undergo multiple photoisomerization cycles without degradation. These properties translated into significantly enhanced isomer-specific GR degradation profiles, potentially driven by functional group-induced changes in CRBN binding interactions that differ between isomeric states, or by optimized spatial orientation of CRBN and GR during ternary complex formation. This aligns with previous reports showing that substituent modifications on arylazopyrazoles significantly influence photochemical behavior, including PSS ratios, switching wavelength, and thermal half-lives (14, 19, 22, 23). Compared to previously reported diazo-linked photoPROTACs, the (OEt)_2_- and Me_2_-functionalized arylazopyrazole photoPROTACs developed here display higher PSS values and substantially prolonged *Z*-isomer thermal half-lives, supporting their suitability for biologically relevant light-controlled protein degradation (*SI Appendix*, Table S2) (10, 15, 18, 24).

To ensure physiological relevance, photophysical properties were evaluated in DMSO–buffer mixtures as DMSO-based measurements may lead to overestimation of in vitro performance. Standard cell culture media cannot be used as it contains UV-active components such as aromatic amino acids that interfere with spectroscopic analysis. This approach revealed the necessity of intermittent irradiation to maintain *Z-*isomer enrichment and achieve selective degradation, emphasizing the dynamic nature of photoPROTAC function in cellular environments. Importantly, the requirement for intermittent irradiation is not unique to this system but reflects the operational state of present photoswitchable degrader technologies (15, 18, 24). Our complementary viability, proteomic, and transcriptomic controls confirm that this illumination regimen does not perturb cellular biology, enabling robust and isomer-specific reversal of GR-driven dormancy.

Given the therapeutic relevance of PROTACs in NSCLC, including their ability to overcome resistance mechanisms (25), our photoPROTACs offer an additional layer of control through light-dependent activation.

In A549 cells, GR activation by DEX induces a dormant state that contributes to therapy resistance (2). Application of KH-5-309 under light-controlled conditions effectively reversed this dormancy, restoring proliferative capacity. Given GR’s ubiquitous expression and essential physiological roles, systemic GR depletion is not viable. These results therefore demonstrate the utility of GR-targeting photoPROTACs as precision tools for modulating GR activity in cancer contexts, where localized and reversible intervention is essential to avoid systemic toxicity.

Despite promising in vitro performance, residual GR degradation by the *Z-*isomers and the requirement for repeated 365 nm irradiation pose challenges for in vivo applications. Limited tissue penetration of short-wavelength light and the potential for phototoxicity further constrain broad translational deployment. These considerations underscore the need for photoswitches that can be activated at longer wavelengths, where biological tissues are more transparent and phototoxicity is minimized. Established strategies within the photopharmacology field, such as red-shifted azobenzene derivatives (26–28), two-photon near-infrared excitation (29, 30), and upconversion-assisted activation (31), provide a clear and feasible path toward longer-wavelength control.

At the same time, these optical constraints define the environments in which photoPROTACs already provide uniquely powerful capabilities. In in vitro and ex vivo systems where light delivery is straightforward, such as cell culture, organoids, and tissue slices, light-dependent GR degradation affords a level of dynamic control that cannot be achieved with conventional chemical inhibitors or genetic perturbations. This includes the ability to modulate GR activity during defined cell cycle, developmental or stress-response windows, as well as to impose patterned perturbations within distinct cellular niches. Such localized modulation is particularly relevant for dissecting the multifaceted roles of glucocorticoid signaling in cancer. For example, restricting GR degradation to the tumor core while preserving GR signaling in surrounding immune or stromal compartments provides a mechanistic resolution that current methodologies cannot access.

In addition to in vitro and ex vivo settings, several established illumination technologies expand the near-term applicability of photoPROTACs in vivo. Neuroscience offers a well-developed framework for deep-tissue optical control. Fiber-optic implants (32, 33) and micro-LED devices (34) routinely used in optogenetics enable precise and repeated light delivery in the brain, illustrating that tissue depth does not preclude optical activation when dedicated light-delivery hardware is employed (35, 36). Similarly, optically accessible tissues provide realistic translational entry points. In skin cancer models, spatial precision may be enhanced using a dual-layer illumination strategy in which peripheral tissue is exposed to an inactivating wavelength to neutralize diffusing compound. Likewise, nasal or airway applications benefit from direct endoscopic access, allowing light-defined spatial confinement of photoPROTAC activity while minimizing systemic exposure (37, 38). Such endoscopic light-delivery routes are already widely used in lung cancer photodynamic therapy, further underscoring the translational feasibility of optical control in these tissues (39, 40). In such contexts, the low nanomolar concentrations required for maximal photoPROTAC activity further reduce off-target risk. Together, these considerations highlight the near-term translational potential of photoPROTACs in tissues where controlled light delivery is already standard of care.

Notably, the *Z-*isomer of KH-5-309 functions as an equally robust negative control as DMSO, providing an internally matched baseline for distinguishing activity-dependent transcriptional effects. In contrast, *E*-KH-5-309 and KH-103 display distinct partial-agonism transcriptional signatures. These findings suggest that residual agonism is shaped primarily by the activity state (i.e., the ability to engage the ternary complex) rather than by the mere presence of a PROTAC molecule. However, this will not necessarily hold for other targets, particularly ligand-independent proteins, where degrader presence alone can elicit transcriptional changes, highlighting the unique value of inactive photoisomers for disentangling activity-dependent from scaffold-dependent effects.

The successful application of GR photoPROTACs in reversing DEX-induced dormancy in NSCLC underscores their potential in oncology, particularly for overcoming hormone-induced resistance mechanisms. Furthermore, the synthetic and photophysical principles established here can be extended to other targets, paving the way for a new generation of precision photopharmacological tools.

This study establishes key design principles for achieving light-controlled, isoform-selective protein degradation using arylazopyrazole-based photoswitches. By applying these principles, we developed GR-targeting photoPROTACs capable of modulating GR activity with high spatial and temporal precision. Their ability to reverse DEX-induced dormancy in NSCLC highlights their potential as both research tools and therapeutic agents in GR-driven pathologies.

## Materials and Methods

### Synthesis and Characterization of photoPROTACs.

Synthetic schemes for the synthesis of the arylazotriazole and arylazopyrazole photoPROTACs are given in the *SI Appendix*, *Supplementary* *Figures*. All experimental procedures and NMR spectra are provided in the *SI Appendix*, *Synthetic* *Procedures* and NMR *Spectra*).

### In-Vitro Cell Culture.

HEK293 cells were donated by the Schratt lab (ETHZ) and maintained in Dulbecco’s Modified Eagle Medium (DMEM; Gibco 10567014) supplemented with GlutaMAX, 1 g/L D-glucose, 110 mg/L sodium pyruvate, 10% fetal bovine serum (FBS; Gibco A5670701), and 1% Penicillin-Streptomycin (P/S; Gibco 15140122). A549 cells (CCL-185) were purchased from American Type Culture Collection and were maintained in DMEM/F12 (1:1; Gibco 11330032) medium supplemented with L-glutamine, 15 mM HEPES, 10% FBS, and 1% P/S. All cells were maintained at 37 °C in a humidified atmosphere with 5% CO_2_.

### Treatment of Cells with photoPROTACs and Compounds.

Cells were seeded in 24-well plates to reach ~70% confluency at the time of treatment. All photoPROTACs and DEX (Sigma-Aldrich D4902) were used at 100 nM except indicated otherwise. Prior to treatment, photoPROTACs were irradiated with their respective wavelengths to induce either the *E-* or *Z-*isomeric state, then added to the cells for 12 or 18 h. MG-132 (MedChemExpress HY-13259) was used at 20 μM. All mentioned compounds were dissolved in DMSO (Sigma-Aldrich, 472301).

### Intermittent Irradiation.

Cells treated with the *Z-*isomer were irradiated every 30 or 60 min for 30 s at 25 mW using a Lumidox II device equipped with 365 nm LEDs. Cells treated with the *E-*isomer were removed from incubators at the same times yet omitting light exposure. Following incubation, protein lysates were collected and stored at –20 °C for subsequent analysis.

### Protein Collection and Quantification.

Cells were lysed in RadioImmunoPrecipitation Assay buffer (RIPA buffer; Invitrogen) supplemented with 1 × protease inhibitor (Roche) by incubating on ice for 20 min. Lysates were then centrifuged at 13,000 rpm for 15 min at 4 °C to separate the protein-containing supernatant from cell debris. Protein samples were resolved on 10% SDS-PAGE gels (mini-PROTEAN TGX, Bio-Rad). Proteins were transferred to nitrocellulose membranes using the Trans-Blot Turbo semidry transfer system (Bio-Rad). Membranes were blocked in 5% milk in TBST for 1 h, followed by overnight incubation at 4 °C with primary antibodies. After washing, membranes were incubated with secondary antibodies for 1 h and developed using Clarity and Clarity Max Western Enhanced Chemiluminescence substrates (Bio-Rad). Protein bands were visualized using the ChemiDoc™ MP Imaging System (Bio-Rad). Precision Plus Protein Dual Color Standards (catalog #1610374) were used as molecular weight markers.

### Antibodies.

The following primary antibodies were used: GR (G-5, Santa Cruz sc-393232, 1:100), MR (E9W1M Rabbit mAB, Cell Signalling 1:1000), alpha-tubulin (11H10, Cell Signalling 2125S, dilution 1:1000), and GAPDH (ABS16, Merck Millipore, 1:1000), Secondary antibodies included goat anti-mouse IgG antibody (Merck Millipore AP308P, (H + L) HRP conjugate, 1:20000) and goat anti-rabbit IgG (Merck Millipore AP307P, (H + L) HRP conjugate, 1:20000).

### RNA Extraction.

Total RNA was extracted using the Quick-RNA™ MicroPrep Kit with Zymo-Spin™ IC Columns (Capped) (Zymo Research, Cat# R1051), following the manufacturer’s instructions. RNA concentration was quantified using both NanoDrop spectrophotometer and the Qubit™ RNA High Sensitivity (HS) Assay Kit (Q32852, Invitrogen).

### C-DNA Synthesis.

700 ng of total RNA was reverse-transcribed into Complementary DNA (cDNA) using M-MLV Reverse Transcriptase (Promega, M1701), Oligo(dt) 15 primers (Promega, C1101), dNTP mix (Thermo Scientific, R0192), and RNasin Ribonuclease Inhibitor (Promega, N2511), following the manufacturers’ instructions.

### RT-qPCR.

cDNA samples were diluted 1:4 in nuclease-free water, and 2 μL of the diluted cDNA was used per reaction. RT-qPCR was performed in technical triplicates using the LightCycler® 480 SYBR Green I Master Mix (Roche, 04707516001) on a CFX384 Real-Time PCR System (Bio-Rad). Gene expression of the target gene CDKN1C was normalized to the housekeeping genes HPRT and PPIA. All primers were obtained from Micro-Synth, and all primer sequences are listed in *SI Appendix*, Table S1.

### RNA Sequencing Library Preparation and Sequencing.

A549 cells (≤ passage 10) were seeded to ~70% confluency and treated for 12 h with 100 nM KH-5-309, 100 nM DEX, DMSO (vehicle), or the indicated combinations. DEX was added during the final 2 h of treatment. Total RNA was isolated using the Quick-RNA™ MicroPrep Kit with Zymo-Spin™ IC Columns (Capped) (Zymo Research, Cat# R1051) according to the manufacturer’s instructions, and integrity assessed through Agilent 2100 Bioanalyzer. All RNA samples were processed at Novogene (Munich, Germany).

Nondirectional Poly-A library preparations were performed with the Novogene NGS RNA Library Prep Set (PT042) according to standard protocols. Briefly, messenger RNA was purified from total RNA using poly-T oligo-attached magnetic beads. The mRNA was then fragmented, and first-strand cDNA synthesis was carried out using random hexamer primers. The second strand of cDNA was then synthesized using dTTP. Subsequent steps included end repair, A-tailing, adapter ligation, and size selection. The libraries were then PCR amplified and purified. For quality control and quantification, libraries were assessed using Qubit and real-time PCR. Size distribution was evaluated using a bioanalyzer (Agilent 2100). After quality control, libraries were pooled based on effective concentration and target data yield. Clusters of index-coded samples were generated, and libraries were sequenced on NovaSeq X Plus with 25B flow cell with PE150 to generate 6G of paired-end reads.

### RNA Sequencing Data Processing and Analysis.

Raw RNA-seq reads were quantified using Salmon v1.10.0 in mapping-based mode with bias correction (--validateMappings, --seqBias, --gcBias) against GENCODE GRCh38.p14 transcript annotations. Transcript-level estimates were aggregated to gene-level using a custom tx2gene mapping file and imported into R using the tximport package. Lowly expressed genes were filtered using filterByExpr() from edgeR, and surrogate variable analysis was performed using SEtools::svacor to correct for hidden batch effects. Differential expression analysis was conducted using generalized linear models and likelihood ratio tests in edgeR, with significance thresholds of FDR < 0.05 and |log^2^FC| > 1.

To account for any minimal transcriptional changes introduced by intermittent irradiation, all differential expression analyses were performed using an interaction term model:

$$\left(E_{\text{KH-5-309 + DEX}}-Z_{\text{KH-5-309 + DEX}}-E_{\text{DEX}}-Z_{\text{DEX}}\right).$$

Because the *Z* handled samples (KH 5 309 + DEX and DEX) receive identical irradiation, this model accounts for all irradiation derived baseline effects prior to testing for treatment specific responses.

### Proteomics Sample Preparation.

The samples dissolved in RIPA lysis buffer were boiled at 95 °C for 10 min and treated with High Intensity Focused Ultrasound for 1 min before centrifugation at 20,000×*g* for 10 min. Protein concentration was determined using the Lunatic UV/Vis polychromatic spectrophotometer (Unchained Labs).

For each sample 25 µg of protein were taken and reduced with 5 mM TCEP[tris(2-carboxyethyl)phosphine] and alkylated with 15 mM chloroacetamide at 30 °C for 30 min.

Samples were processed using the single‐pot solid‐phase enhanced sample preparation (SP3). The SP3 protein purification, digest, and peptide clean-up was performed using a KingFisher Flex System (Thermo Fisher Scientific) and Carboxylate-Modified Magnetic Particles (GE Life Sciences; GE65152105050250, GE45152105050250) (41, 42). Beads were conditioned following the manufacturer’s instructions, consisting of 3 washes with water at a concentration of 1 µg/µL. Samples were diluted with 100% ethanol to a final concentration of 60% ethanol. The beads, wash solutions, and samples were loaded into 96 deep well- or microplates and transferred to the KingFisher. Following steps were carried out on the robot: collection of beads from the last wash, protein binding to beads, washing of beads in wash solutions 1 to 3 (80% ethanol), protein digestion [overnight at 37 °C with a trypsin:protein ratio of 1:50 in 50 mM Triethylammoniumbicarbonat (TEAB)] and peptide elution from the magnetic beads using MilliQ water. The digest solution and water elution were combined and dried to completeness and resolubilized in 20 µL of MS sample buffer (3% acetonitrile, 0.1% formic acid).

### LC–MS/MS.

Mass spectrometry analysis was performed on an Orbitrap Exploris 480 mass spectrometer (Thermo Fisher Scientific) equipped with a Nanospray Flex Ion Source (Thermo Fisher Scientific) and coupled to an M-Class Ultra Performance Liquid Chromatography (Waters). Solvent composition at the two channels was 0.1% formic acid for channel A and 0.1% formic acid, 99.9% acetonitrile for channel B. Column temperature was 50 °C. For each sample 200 Abs of peptides were loaded on a commercial nanoEase MZ Symmetry C18 Trap Column (100 Å, 5 µm, 180 µm × 20 mm, Waters) followed by a nanoEase MZ C18 HSS T3 Column (100 Å, 1.8 µm, 75 µm × 250 mm, Waters). The peptides were eluted at a flow rate of 300 nL/min. After a 3 min initial hold at 5% B, a gradient from 5 to 22% B in 80 min and 22 to 32% B in additional 10 min was applied. The column was cleaned after the run by increasing to 95% B and holding 95% B for 10 min prior to reestablishing loading condition for another 10 min.

For the analysis of the individual samples, the mass spectrometer was operated in data-independent mode (DIA). DIA scans covered a range from 400 to 960 m/z in windows of 8 m/z. The resolution of the DIA windows was set to 30,000, with a normalized AGC target value of 1,000%, the maximum injection time set to auto and a fixed normalized collision energy of 30%. Each instrument cycle was completed by a full MS scan monitoring 396 to 1,000 m/z at a resolution of 60,000. The mass spectrometry proteomics data were handled using the local laboratory information management system (43).

### LC–MS/MS Data Analysis.

The acquired MS data were processed for identification and quantification using DIANN v1.9 (44). Spectra were searched using the Uniprot human database in FASTA file format (1 sequence per gene), and common protein contaminants. Carbamidomethylation of cysteine was fixed modifications, while methionine oxidation was variable. Enzyme specificity was set to trypsin/P, allowing a minimal peptide length of seven amino acids and a maximum of two missed cleavages.

The R package prolfqua (45) was used to analyze the differential expression and to determine group differences, CI, and FDRs for all quantifiable proteins. The protein lists were filtered with a threshold of 1 log2FC and an FDR of 0.05%. The analysis was run on the local computing infrastructure (46).

### Flow Cytometry.

Cells were washed once with PBS, trypsinized, and collected in PBS supplemented with 2% FBS. For viability discrimination, cells were stained immediately prior to acquisition by adding 1 µL of 1 mg/mL DAPI to 1 mL of the cell suspension (final: 1 µg/mL). Samples were gently mixed and kept on ice until analysis. Flow-cytometry measurements were performed on a Cytek Aurora 5-laser spectral cytometer, and data were analyzed using FlowJo (BD). DAPI-negative events were gated as live cells.

### Statistical Analysis.

All statistical analyses, except for RNA-seq data processing, were performed using built-in functions in GraphPad Prism. A confidence level of 95% was applied throughout, and p-values below 0.05 were considered statistically significant. Data were assumed to follow a normal distribution with equal variance across groups. Error bars represent the SEM, unless otherwise specified. The specific statistical tests and parameters used are detailed in the corresponding figure legends. Data presented as box-and-whiskers plots (min to max) include a line indicating the median.

## Supplementary Material

Appendix 01 (PDF)

## Data Availability

Analysis scripts and visualization code are available on GitHub: https://github.com/robinsch99/A549_PhotoPROTAC (47). RNA-seq data generated in this study have been deposited in the Gene Expression Omnibus (GEO) under accession number GSE304966 (48). Proteomics data have been deposited to the ProteomeXchange Consortium via the PRIDE (http://www.ebi.ac.uk/pride) partner repository with the dataset identifier PXD075518 (49). All other data are included in the manuscript and/or *SI Appendix*. Previously published RNA-seq data were obtained from GEO accession GSE229084 (50). The accepted manuscript and all primary data supporting the findings of this study have been deposited in the ETH Research Collection and are available at https://doi.org/10.3929/ethz-c-000798697 (51).
