# Supplementary material for: Light-controlled disruption of cancer cell dormancy via photoswitchable stress hormone receptor degraders
Source: Proc Natl Acad Sci U S A. 2026 May 21;123(21):e2528760123. doi: 10.1073/pnas.2528760123 (PMC13214037; doi:10.1073/pnas.2528760123)

Supporting Information for
Light-Controlled Disruption of Cancer Cell Dormancy via Photoswitchable Stress Hormone Receptor Degraders

Karina M. Freitag[1,a], Robin Scheuplein[1,b,c], Chiara Orlacchio[b], Viola Ansuinelli[b], Tommaso Fava[b], Vincent Fischer[b,c], Bohan Zhang[b], Miriam Kretschmer[b,c], Mahshid Garzorpak[b,c], Erick M. Carreira*[,a], Katharina Gapp*[,b,c]

[a] Department of Chemistry and Applied Biosciences, Laboratory of Organic Chemistry, ETH Zürich, 8093 Zürich, Switzerland

[b] Laboratory of Epigenetics and Neuroendocrinology, Institute for Neuroscience, Department of Health Science and Technology, ETH Zürich, Zürich 8057, Switzerland

[c] Neuroscience Center Zürich, ETH Zürich and University of Zürich, Zürich 8057, Switzerland

[1] These authors contributed equally to this work.

* Co-corresponding authors:

Prof. Dr. Katharina Gapp

ETH Zürich, Laboratory of Epigenetics and Neuroendocrinology, Y17 M 3, Winterthurerstrasse 190, 8057 Zürich, Switzerland

Phone: +41 44 633 85 89

Email: katharina.gapp@hest.ethz.ch

Prof. Dr. Erick M. Carreira

Department of Chemistry and Applied Biosciences, Laboratory of Organic Chemistry, ETH Zürich,

8093 Zürich, Switzerland

Phone: +41 44 632 28 30

Email: erickm.carreira@org.chem.ethz.ch

Table of Contents

1. Supplementary Figures 4

Overview of photoPROTAC Classes and Linker Variants 5

Synthesis of arylazotriazole-dexa-linked photoPROTACs KH-5-168 – KH-5-170 6

Synthesis of arylazotriazole-lenalidomide-linked photoPROTACs KH-5-210 – KH-5-226 9

Synthesis of Me_2-arylazopyrazole photoswitch 13

Synthesis of $(CF_3)_2$-arylazopyrazole photoswitch 14

Synthesis of $(NMe_2)_2$-arylazopyrazole photoswitch 15

Synthesis of $(OEt)_2$-arylazopyrazole photoswitch 17

Synthesis of Me_2-arylazopyrazole photoPROTACs KH-5-298 – KH-5-309 19

Synthesis of $(CF_3)_2$-arylazopyrazole photoPROTACs KH-5-327 – KH-5-340 20

Synthesis of $(OEt)_2$-arylazopyrazole photoPROTACs KH-5-450–KH-5-456 21

UV-Vis Spectra of photoswitches 22

Thermal Relaxation of photoswitches 26

PSS Determination by HPLC Analysis 27

PSS Determination by NMR Spectroscopy 43

Photophysical properties of Me_2-arylazopyrazole photoPROTAC 44

Photophysical properties of $(OEt)_2$-arylazopyrazole photoPROTAC 45

Photophysical properties of $(CF_3)_2$-arylazopyrazole photoPROTAC 46

Western Blot Analysis of GR Degradation Induced by arylazotriazole–dexa-linked photoPROTACs 49

Western Blot Analysis of GR Degradation Induced by arylazotriazole-lenalidomide-linked photoPROTACs 51

Western Blot Analysis of GR Degradation Induced by $(CF_3)_2$-, and $(OEt)_2$- arylazopyrazole photoPROTACs 54

Thermal Relaxation of photoPROTACs 57

UV-Vis Spectra of photoPROTACs in Buffer-DMSO (3:1) 59

Western Blot Analysis of GR Degradation Induced by arylazopyrazole photoPROTACs with intermittent irradiation 60

Western Blot Analysis of GR Degradation in A549 cells Induced by GR PROTAC 63

Application of KH-5-309 in A549 Cells 65

2. Supplementary Table 75

3. Detailed Chemical Methods 77

3.1 General Methods 77

3.2 Solvents and Reagents 77

3.3 Analytical Techniques 77

3.3.1 Nuclear Magnetic Resonance (NMR) Spectroscopy 77

3.3.2 Infrared Spectroscopy 77

3.3.3 Mass Spectrometry 77

3.4 Photophysical Characterization 77
3.4.1 General Procedures 77
3.4.2 UV–Vis Spectroscopy and PSS Composition 77
3.4.3 Thermal Relaxation and Half-Life Determination 78
3.5 Photostability 78
4. Synthetic Procedures 79
Arylazotriazole–dexa-linked photoPROTACs 79
Arylazotriazole–lenalidomide-linked photoPROTACs 85
GR Me_2-Arylazopyrazole photoPROTACs 107
GR $(CF_3)_2$-Arylazopyrazole photoPROTACs 122
GR $(OEt)_2$-Arylazopyrazole photoPROTACs 137
$(NMe_2)_2$-Arylazopyrazole Photoswitch 154
5. NMR Spectra 157
Arylazotriazole-dexa-linked photoPROTACs 157
Arylazotriazole–Lenalidomide-Linked photoPROTACs 169
Me_2-Arylazopyrazole photoPROTACs 194
$(CF_3)_2$-Arylazopyrazole photoPROTACs 223
$(OEt)_2$-Arylazopyrazole photoPROTACs 255
$(NMe_2)_2$-Arylazopyrazole Photoswitch 289

1. Supplementary Figures

A

KH-128

KH-129

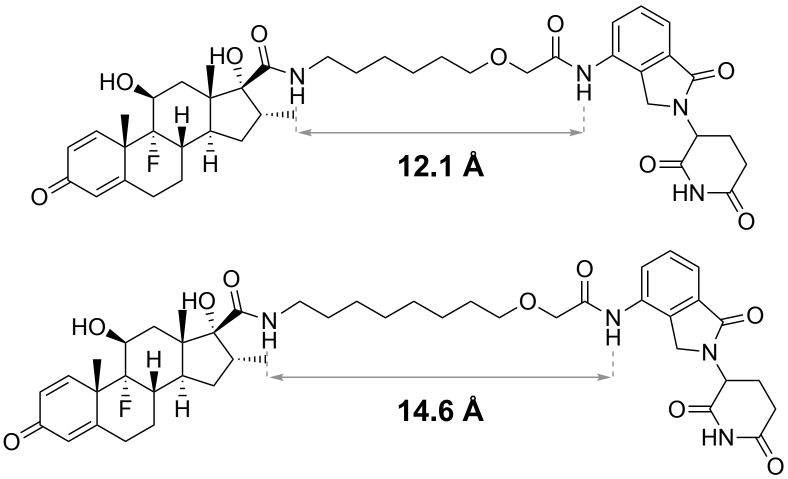

B

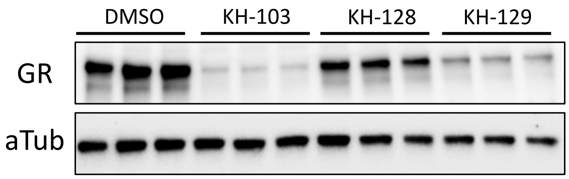

Fig. S1. Rational design considerations for the synthesis of GR photoPROTACs. A) Linker lengths of GR PROTACs KH-128 and KH-129 measured with Chem3D. B) In vitro assessment of the degradation activity of KH-128 and KH-129 compared to our previously published GR PROTAC KH-103. HEK293 cells were incubated with KH-128 and KH-129 (100 nM, DMSO) and GR levels were evaluated by immunoblotting 16 h post-administration.

Overview of photoPROTAC Classes and Linker Variants

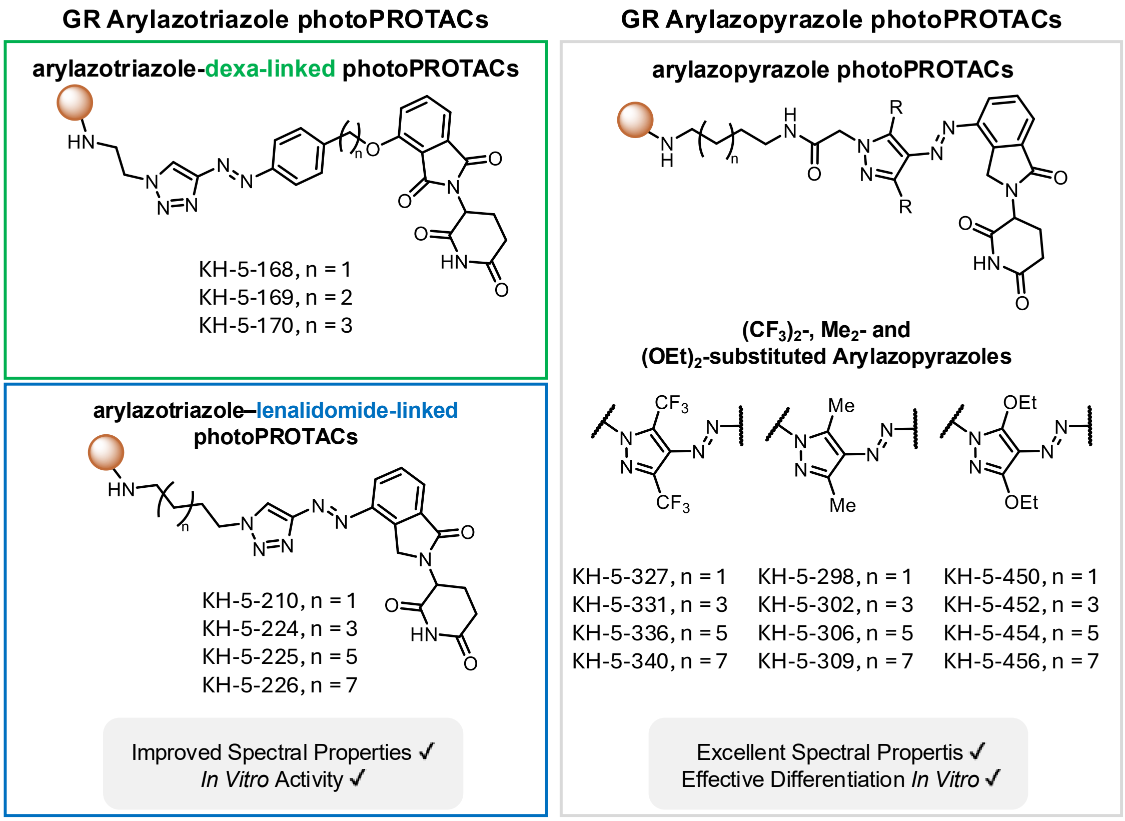

Fig. S2. Merging PROTAC-mediated degradation and photopharmacology to explore a photoPROTAC approach for the conditional depletion of GR. Three structural classes are shown: (i) arylazotriazole-dexa-linked photoPROTACs (KH-5-168, KH-5-169, KH-5-170), (ii) arylazotriazole -lenalidomide-linked photoPROTACs (KH-5-210, KH-5-224, KH-5-225, KH-5-226), and (iii) arylazopyrazole photoPROTACs with $(CF_3)_2$ (KH-5-327 to KH-5-340), Me_2 (KH-5-298 to KH-5-309), and $(OEt)_2$ substitutions (KH-5-450 to KH-5-456). Each series includes photoPROTACs with varying linker lengths (n = 1–7).

Synthesis of arylazotriazole-dexa-linked photoPROTACs KH-5-168 – KH-5-170

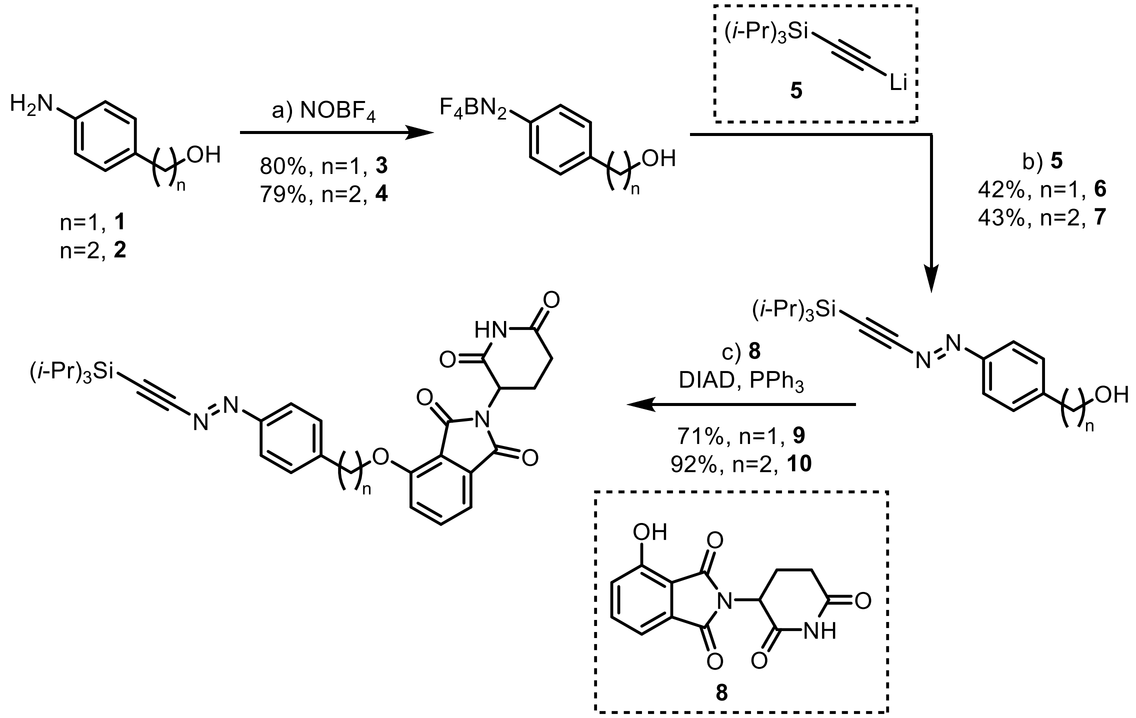

Fig. S3. Synthesis of azoacetylenes **9** and **10**. Reagents and conditions: a) $NOBF_4$, EtOAc, 0 °C, 1 h, 80% for **3**, 79% for **4**; b) **5**, THF, –78 °C to r.t., 1 h, 42% for **6**, 43% for **7**; c) DIAD, Ph_3P, THF, r.t., 2 h, 71% for **9**, 92% for **10**.[1]

[1] Molecules were synthesized in collaboration with Dr. Patrick Pfaff, ETH Zurich. For the synthesis of azoacetylene **9** and **10**, see: Diss. ETH No. 28982.

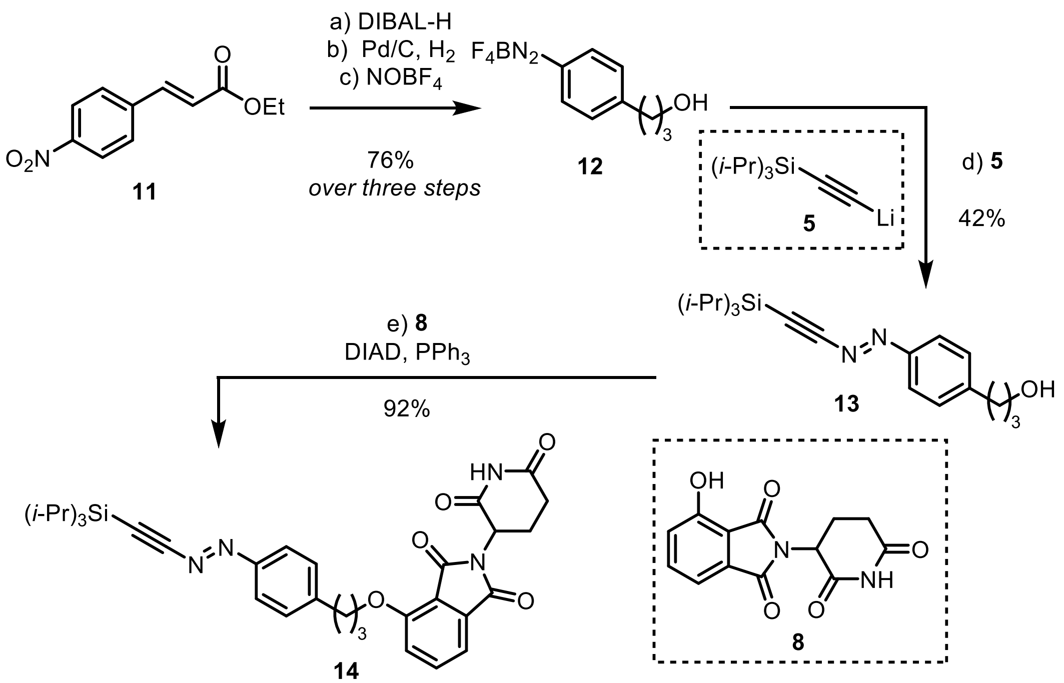

Fig. S4. Synthesis of azoacetylene **14**. Reagents and conditions: a) DIBAL-H, CH_2Cl_2, 0 °C, 2 h, quant.; b) Pd/C, H_2, MeOH, 14 h, r.t., 91%; c) $NOBF_4$, EtOAc, 0 °C, 1 h, 83%; d) **5**, THF, –78 °C to r.t., 1 h, 42%; e) **8**, DIAD, Ph_3P, THF, r.t., 2 h, 92%.[2]

[2] Molecules were synthesized in collaboration with Dr. Patrick Pfaff, ETH Zurich. For the synthesis of azoacetylene **14**, see: Diss. ETH No. 28982.

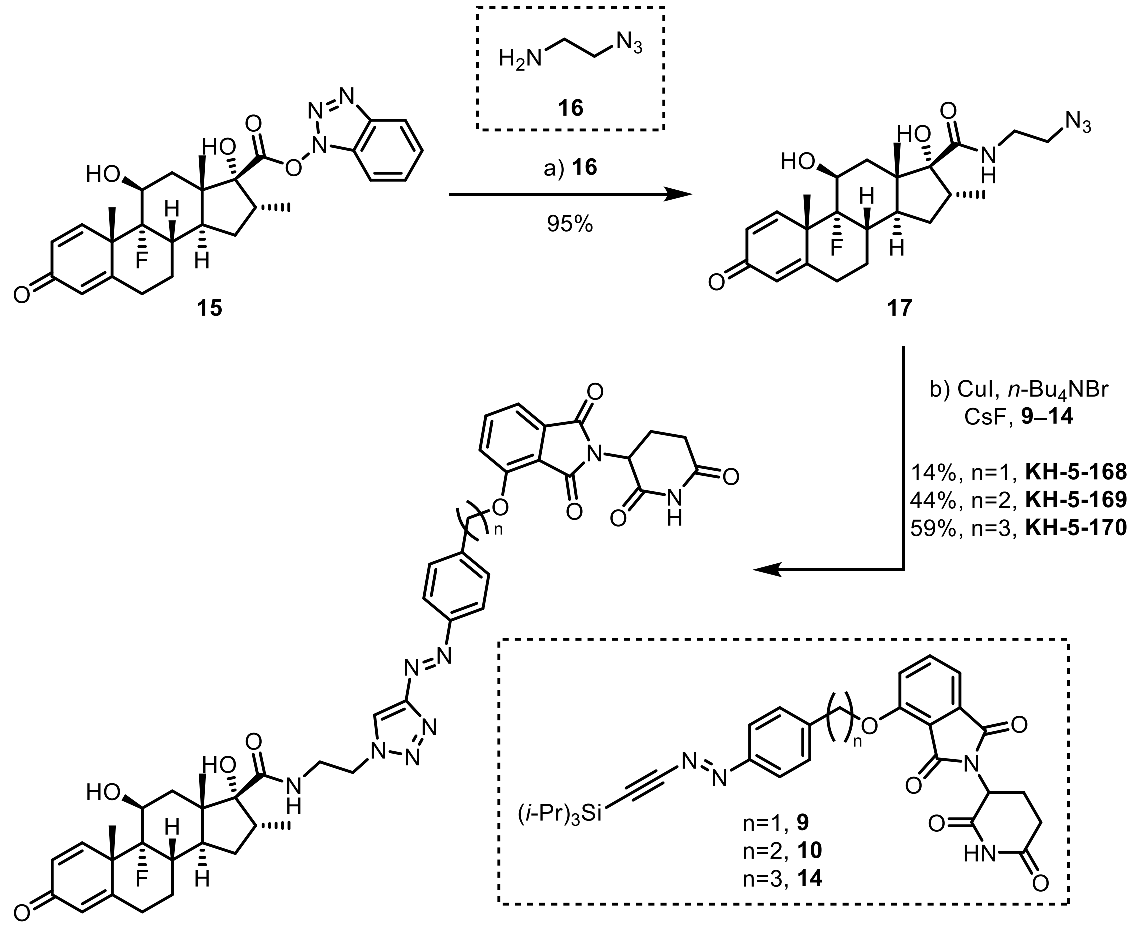

Fig. S5. Synthesis of GR arylazotriazole-dexa-linked photoPROTACs **KH-5-168–KH-5-170**. Reagents and conditions: a) **16**, CH_2Cl_2, 4 °C, 16 h, 95%; b) CuI, *n*-Bu_4NBr, CsF, **9–14**, THF–water (3:1), 40 °C, 24 h, 14% for **KH-5-168**, 44% for **KH-5-169**, 59% for **KH-5-170**.

Synthesis of arylazotriazole-lenalidomide-linked photoPROTACs KH-5-210 – KH-5-226

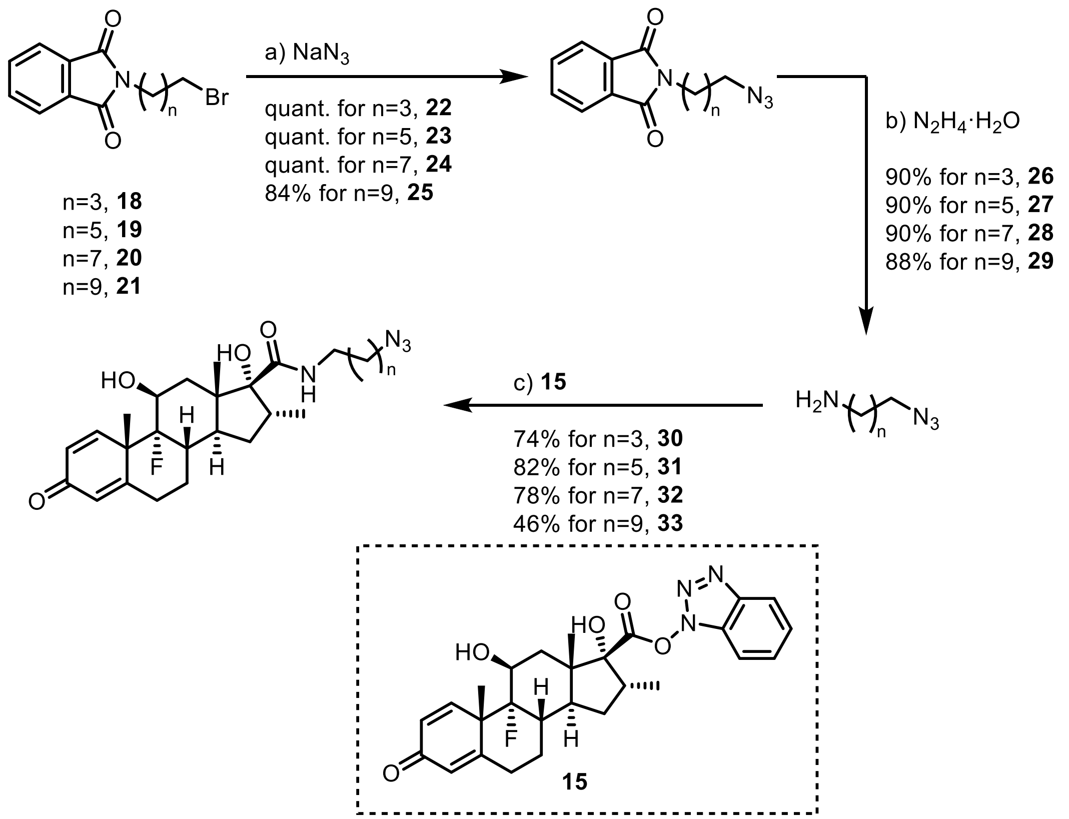

Fig. S6. Synthesis of dexa-azides **30–33**. Reagents and conditions: a) NaN_3, DMF, r.t.– 90 °C, 6–16 h, quant. for **22**, quant. for **23**, quant. for **24**, 84% for **25**; b) $N_2H_4 \cdot H_2O$, THF, 60 °C, 6 h, 90% for **26**, 90% for **27**, 90% for **28**, 88% for **29**; c) **15**, CH_2Cl_2, 0 °C to r.t., 16 h, 74% for **30**, 82% for **31**, 78% for **32**, 46% for **33**.

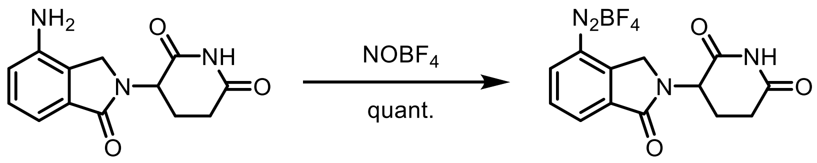

lenalidomide **34** **35**

Fig. S7. Synthesis of diazonium tetrafluoroborate **35**. Reagents and conditions: $NOBF_4$, MeCN–EtOAc (1:1), 0 °C to r.t., quant.

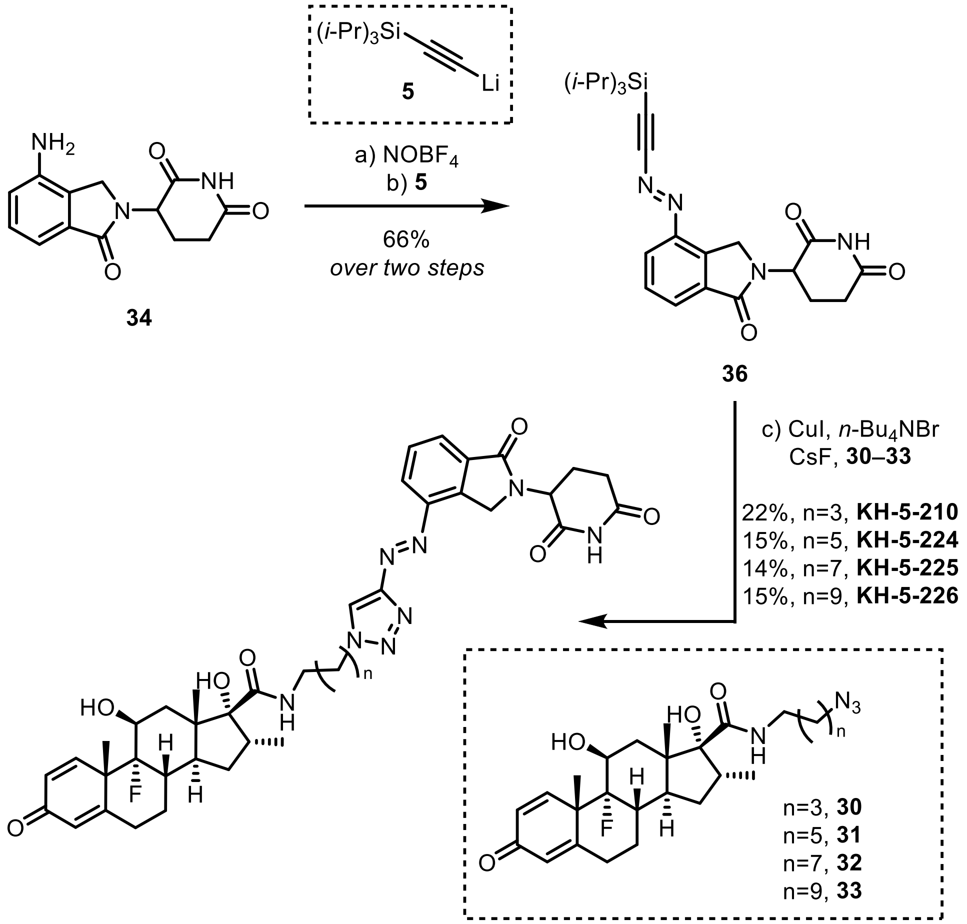

Fig. S8. Synthesis of arylazotriazole–lenalidomide-linked photoPROTACs **KH-5-210–KH-5-226**. Reagents and conditions: a) $NOBF_4$, MeCN–EtOAc (1:1), 0 °C to r.t., quant.; b) **5**, THF, -78 °C, 66%; c) CuI, *n*-Bu_4NBr, CsF, THF–buffer (phosphate buffer, pH=7.0, (3:1)), 40 °C, 24 h, **30–33**, 22% for **KH-5-210**, 15% for **KH-5-224**, 14% for **KH-5-225**, 15% for **KH-5-226**.

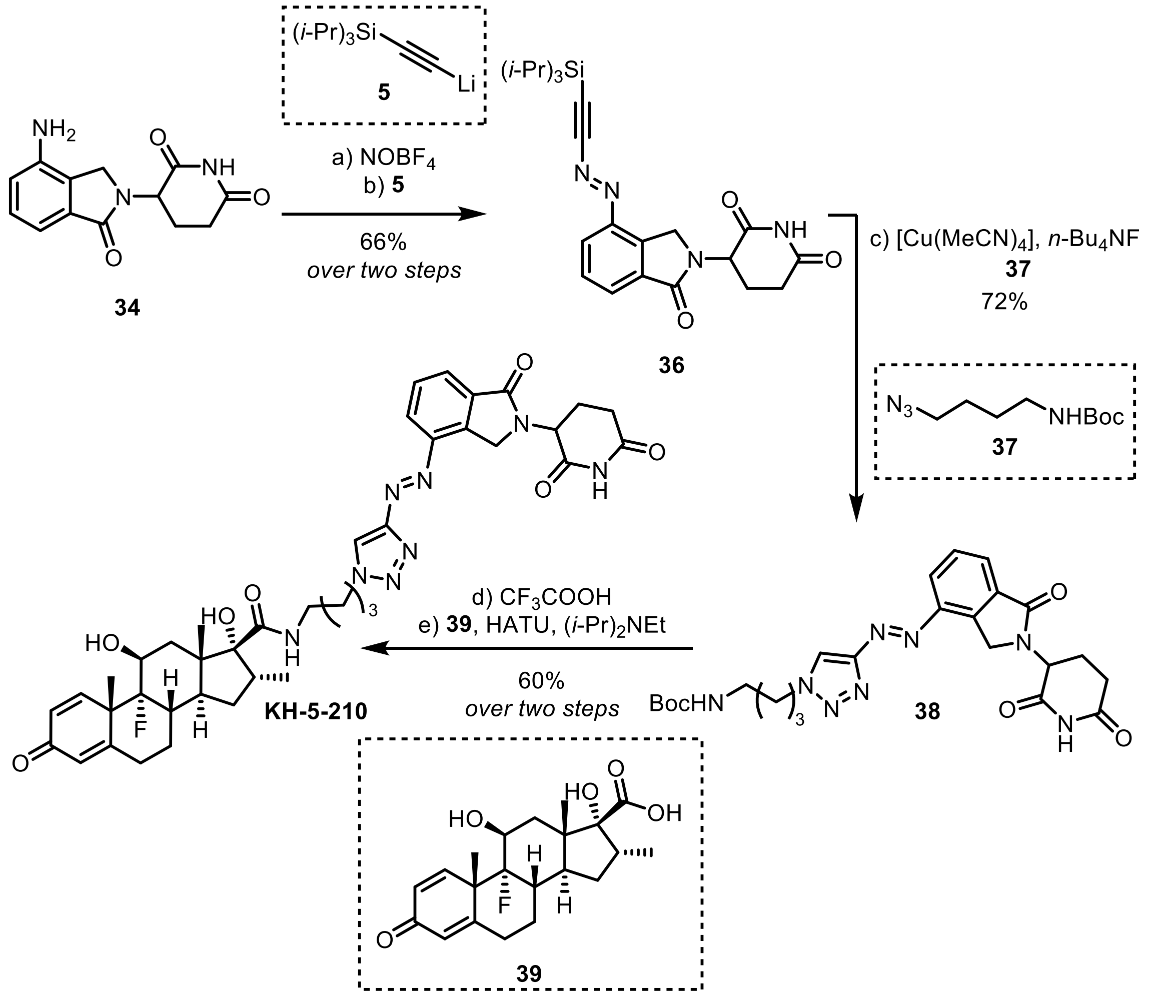

Fig. S9. Optimized synthetic route to access GR arylazotriazole photoPROTAC **KH-5-210**. Reagents and conditions: a) $NOBF_4$, MeCN–EtOAc (1:1), 0 °C to r.t., 2 h, quant.; b) **5**, THF, – 78 °C, 66% over 2 steps; c) $[Cu(MeCN)_4]$, *n*-Bu_4NF (1.0 M in THF), **37**, CH_2Cl_2, r.t., 24 h, 72%; d) CF_3COOH, CH_2Cl_2, r.t. 2 h; e) **39**, HATU, (*i*-Pr)$_2$NEt, DMF, r.t., 24 h, 60% over 2 steps.

Synthesis of Me_2-arylazopyrazole photoswitch

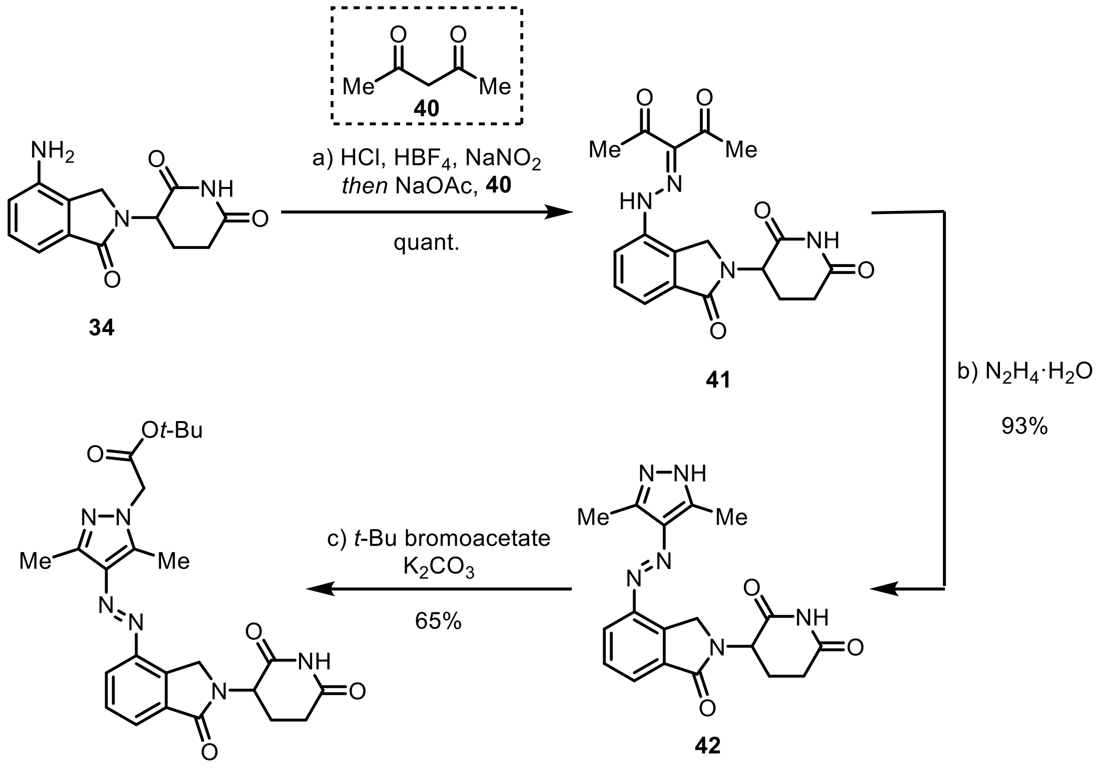

Me_2-arylazopyrazole photoswitch

Fig. S10. Synthesis of **Me_2-arylazopyrazole photoswitch**. Reagents and conditions: a) HCl, HBF_4, $NaNO_2$, NaOAc, acetylacetone (**40**), MeOH–water, 0 °C, 30 min, quant.; b) $N_2H_4 \cdot H_2O$, THF, 70 °C, 45 min, 93%; c) *t*-Bu bromoacetate, K_2CO_3, DMF, r.t., 16 h, 65%.

Synthesis of $(CF_3)_2$-arylazopyrazole photoswitch

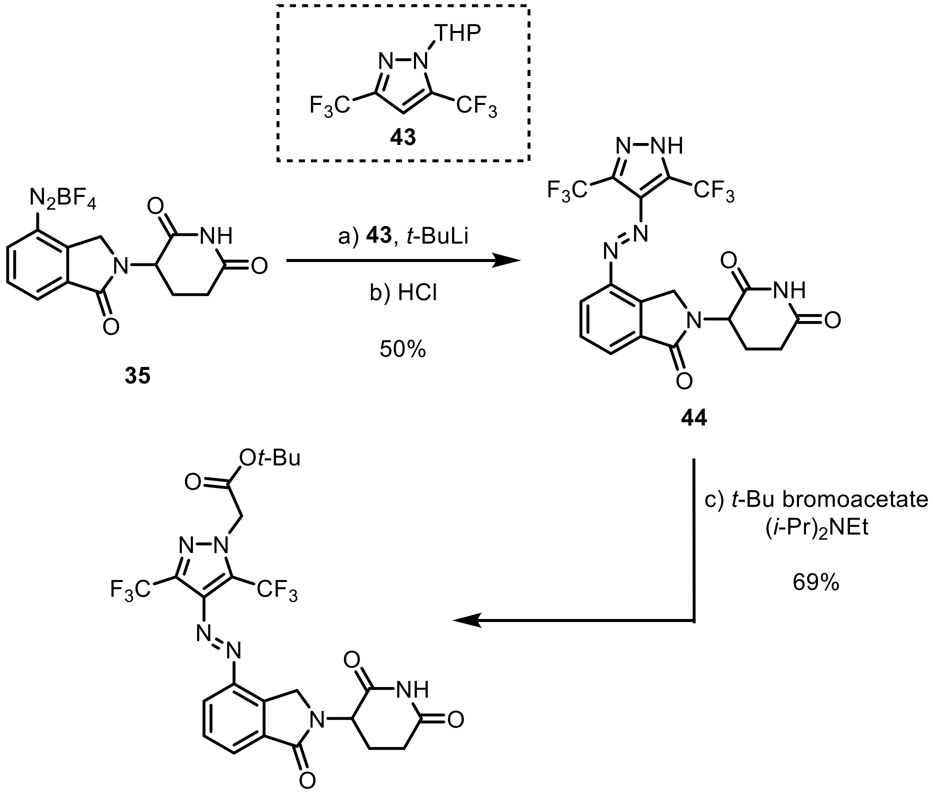

$(CF_3)_2$-arylazopyrazole photoswitch

Fig. S11. Synthesis of **$(CF_3)_2$-arylazopyrazole photoswitch**. Reagents and conditions: a) **43**, *t*-BuLi, THF–Et_2O, –78 °C to r.t to –78 °C, 1 h, 68%; b) HCl (10%), CF_3CH_2OH, r.t., 74%; c) *t*-Bu bromoacetate, (*i*-Pr)$_2$NEt, DMF, 40 °C, 5 h, 69%.

Synthesis of $(NMe_2)_2$-arylazopyrazole photoswitch

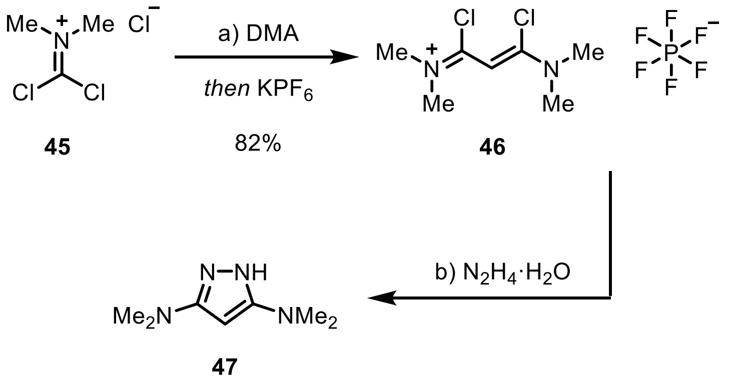

Fig. S12. Synthesis of $(NMe_2)_2$-pyrazole **47**. Reagents and conditions: a) *N*,*N*-Dimethylacetamide (DMA), CH_2Cl_2, 40 °C, then KPF_6, water, r.t., 82%; b) $N_2H_4{\cdot}H_2O$, CH_2Cl_2, 45 °C, 1 h.

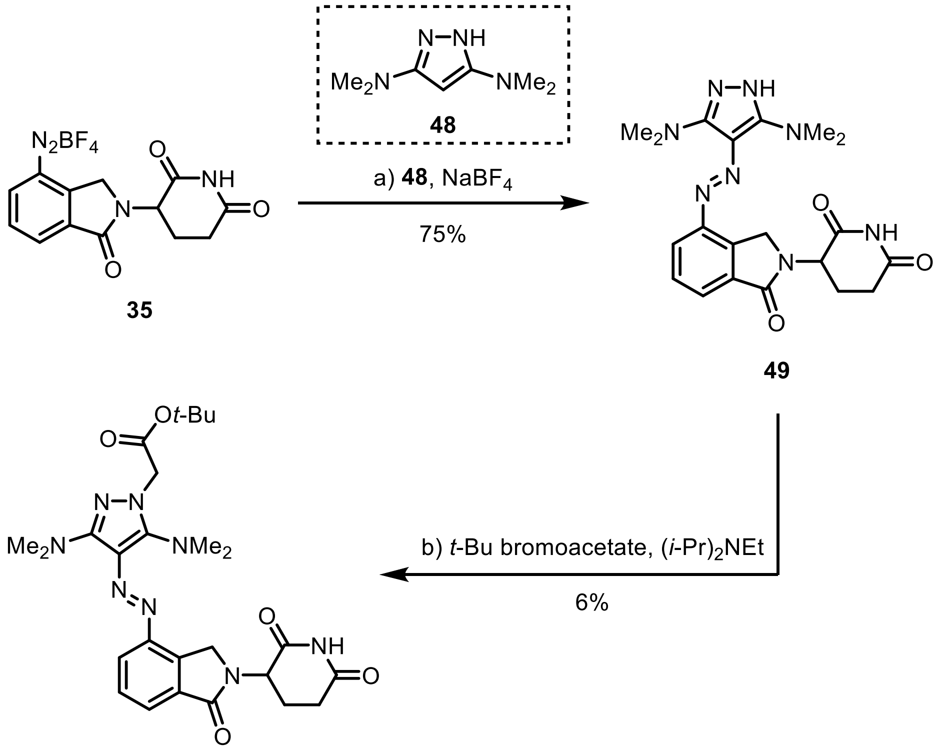

(NMe$_2$)$_2$-arylazopyrazole photoswitch

Fig. S13. Synthesis of **(NMe$_2$)$_2$-arylazopyrazole photoswitch**. Reagents and conditions: a) **48**, $NaBF_4$, MeCN, 0 °C to r.t., 1 h, 75%; b) *t*-Bu bromoacetate, (*i*-Pr)$_2$NEt, DMF, 100 °C, 11 h, 6%.

Synthesis of $(OEt)_2$-arylazopyrazole photoswitch

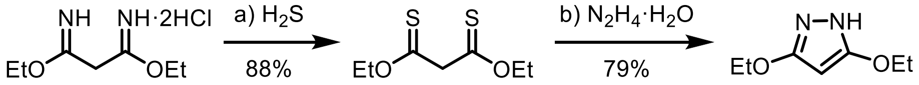

50 **51** **52**

Fig. S14. Synthesis of $(OEt)_2$-arylazopyrazole **52**. Reagents and conditions: a) FeS, aq. HCl (5 M), AcOH–pyridine (9:1), r.t., 52 h, 88%; b) $N_2H_4 \cdot H_2O$, ether, r.t., 6 h, 79%.

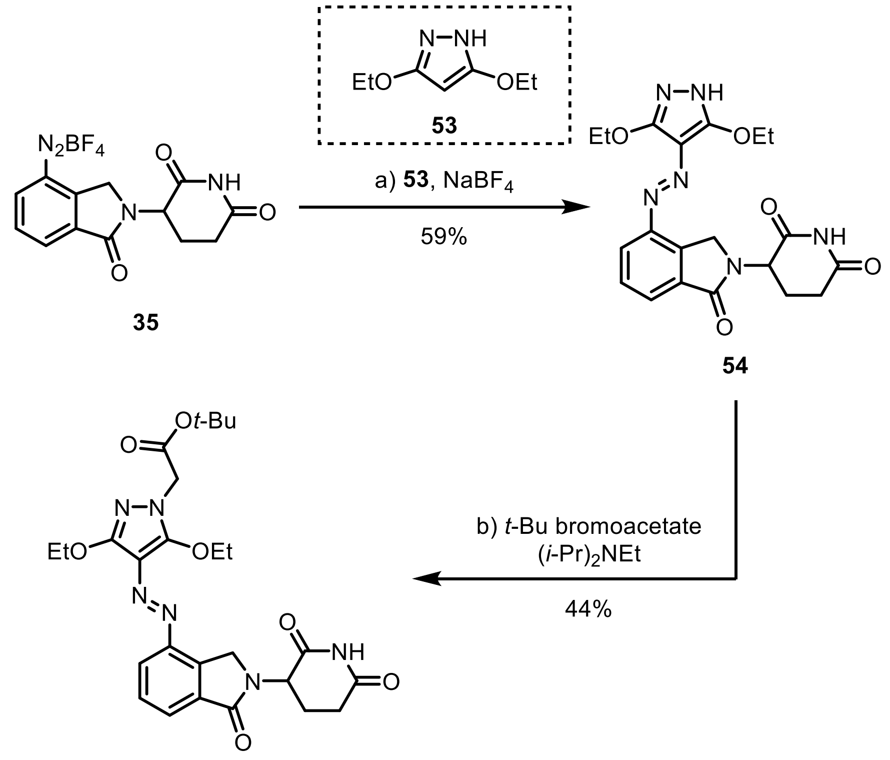

$(OEt)_2$-arylazopyrazole photoswitch

Fig. S15. Synthesis of arylazo **$(OEt)_2$-arylazopyrazole photoswitch**. Reagents and conditions: a) **53**, $NaBF_4$, MeCN, 0 °C, 1 h, 59%; b) *t*-Bu bromoacetate, $(i\text{-}Pr)_2NEt$, DMF, 80 °C, 12 h, 44%.

Synthesis of Me_2-arylazopyrazole photoPROTACs KH-5-298 – KH-5-309

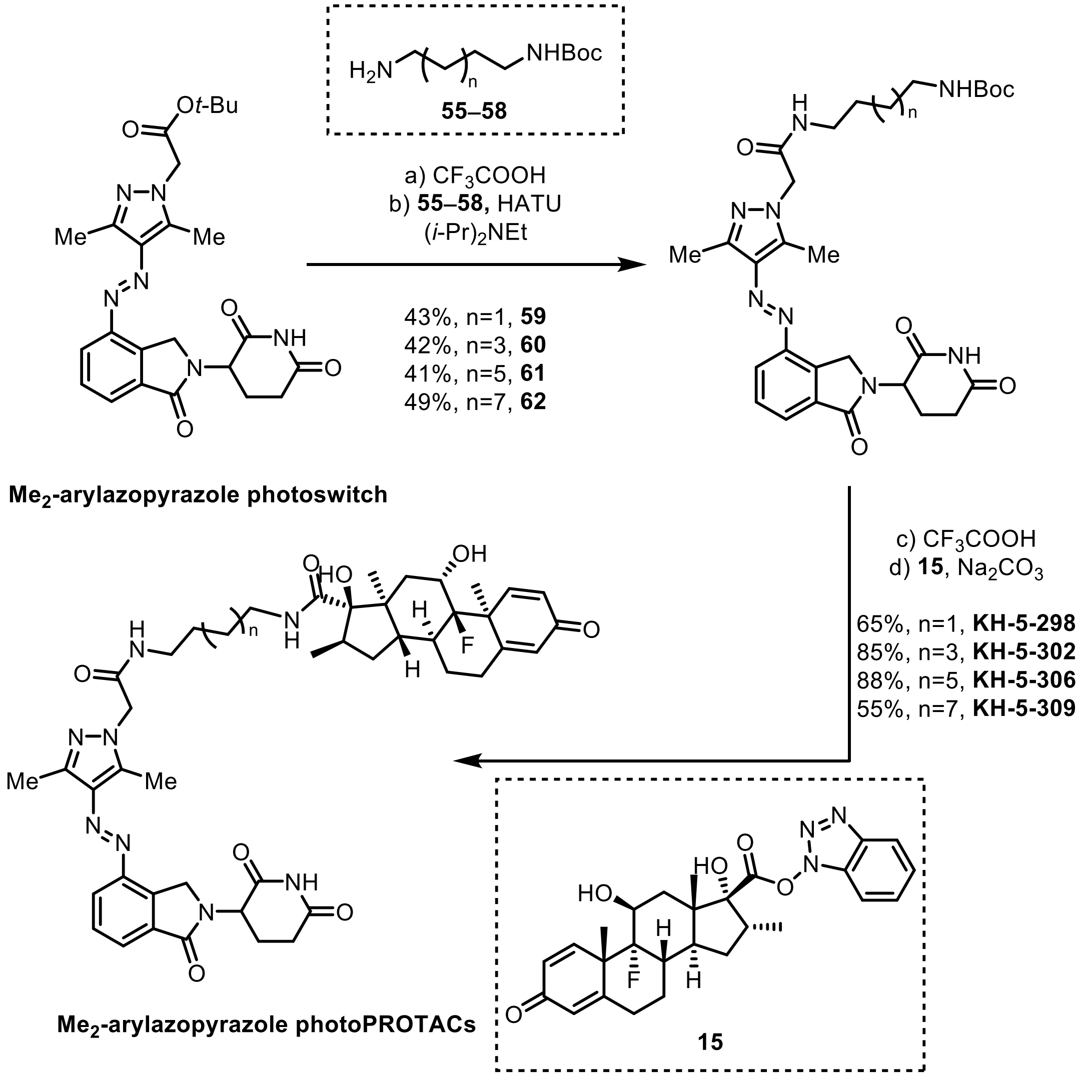

Fig. S16. Synthesis of Me_2-arylazopyrazole photoPROTACs **KH-5-298–KH-5-309.** Reagents and conditions: a) CF_3COOH, CH_2Cl_2, r.t., 2 h; b) **55–58**, HATU, (*i*-Pr)$_2$NEt, DMF, r.t.,16 h, 43% for **59**, 42% for **60**, 41% for **61**, 49% for **62**; c) CF_3COOH, CH_2Cl_2, r.t., 2 h; d) **15**, Na_2CO_3, DMF, r.t., 24 h, 65% for **KH-5-298**, 85% for **KH-5-302**, 88% for **KH-5-306**, 55% for **KH-5-309**.

Synthesis of $(CF_3)_2$-arylazopyrazole photoPROTACs KH-5-327 – KH-5-340

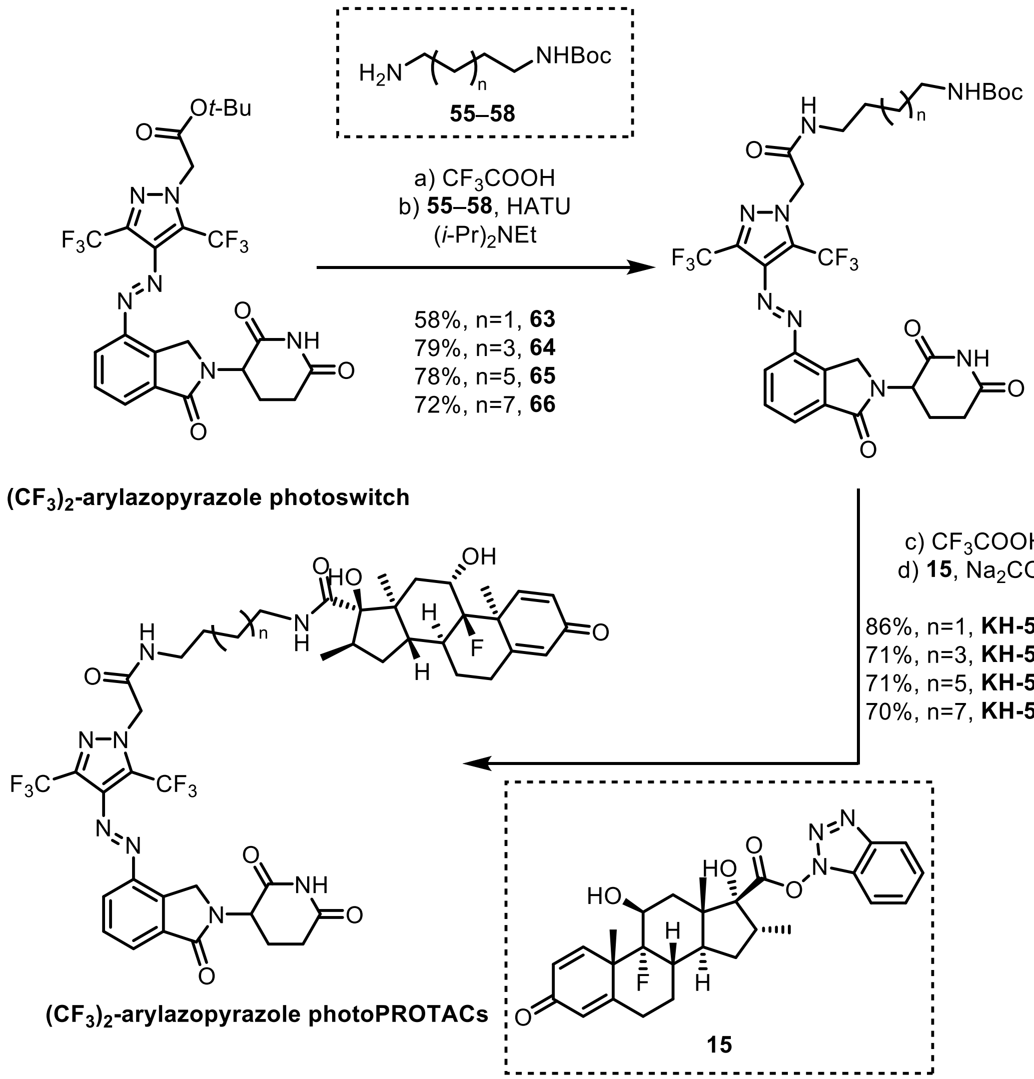

Fig. S17. Synthesis of $(CF_3)_2$-arylazopyrazole photoPROTACs **KH-5-327**–**KH-5-340**. Reagents and conditions: a) CF_3COOH, CH_2Cl_2, r.t., 2 h; b) **55**–**58**, HATU, (*i*-Pr)$_2$NEt, DMF, r.t.,16 h, 58% for **63**, 79% for **64**, 78% for **65**, 72% for **66**; c) CF_3COOH, CH_2Cl_2, r.t., 2 h; d) **15**, Na_2CO_3, DMF, r.t., 24 h, 86% for **KH-5-327**, 71% for **KH-5-331**, 71% for **KH-5-336**, 70% for **KH-5-340**.

Synthesis of (OEt)2-arylazopyrazole photoPROTACs KH-5-450–KH-5-456

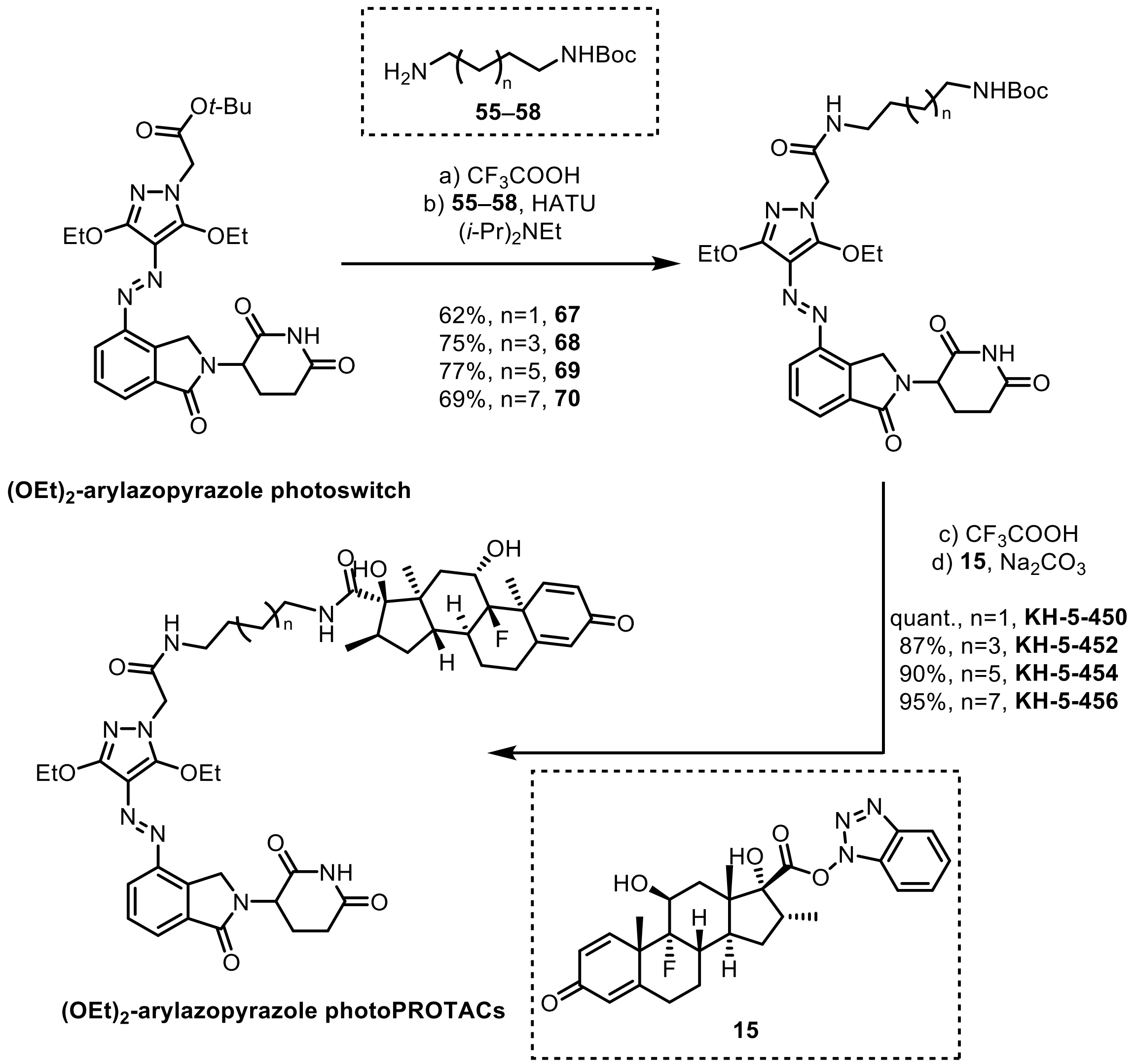

Fig. S18. Synthesis of (OEt)2-arylazopyrazole photoPROTACs **KH-5-450**–**KH-5-456**. Reagents and conditions: a) CF_3COOH, CH_2Cl_2, r.t., 2 h; b) **55**–**58**, HATU, (*i*-Pr)2NEt, DMF, r.t.,16 h, 62% for **67**, 75% for **68**, 77% for **69**, 69% for **70**; c) CF_3COOH, CH_2Cl_2, r.t., 2 h; d) **15**, Na_2CO_3, DMF, r.t., 24 h, quant. for **KH-5-450**, 87% for **KH-5-452**, 90% for **KH-5-454**, 95% for **KH-5-456**.

UV-Vis Spectra of photoswitches

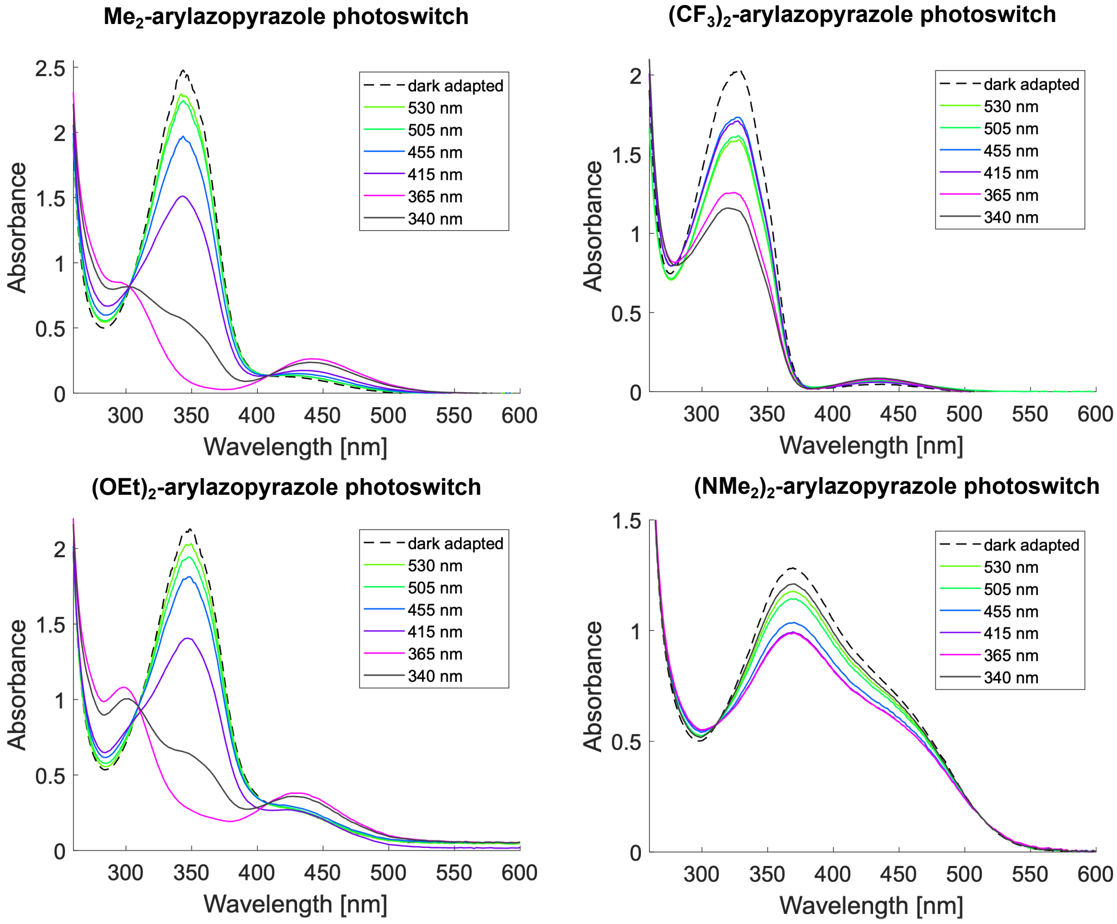

Fig. S19. UV-vis spectra of arylazopyrazole photoswitches in DMSO after irradiation for 30 min (340 nm) or 20 min (365–530 nm)

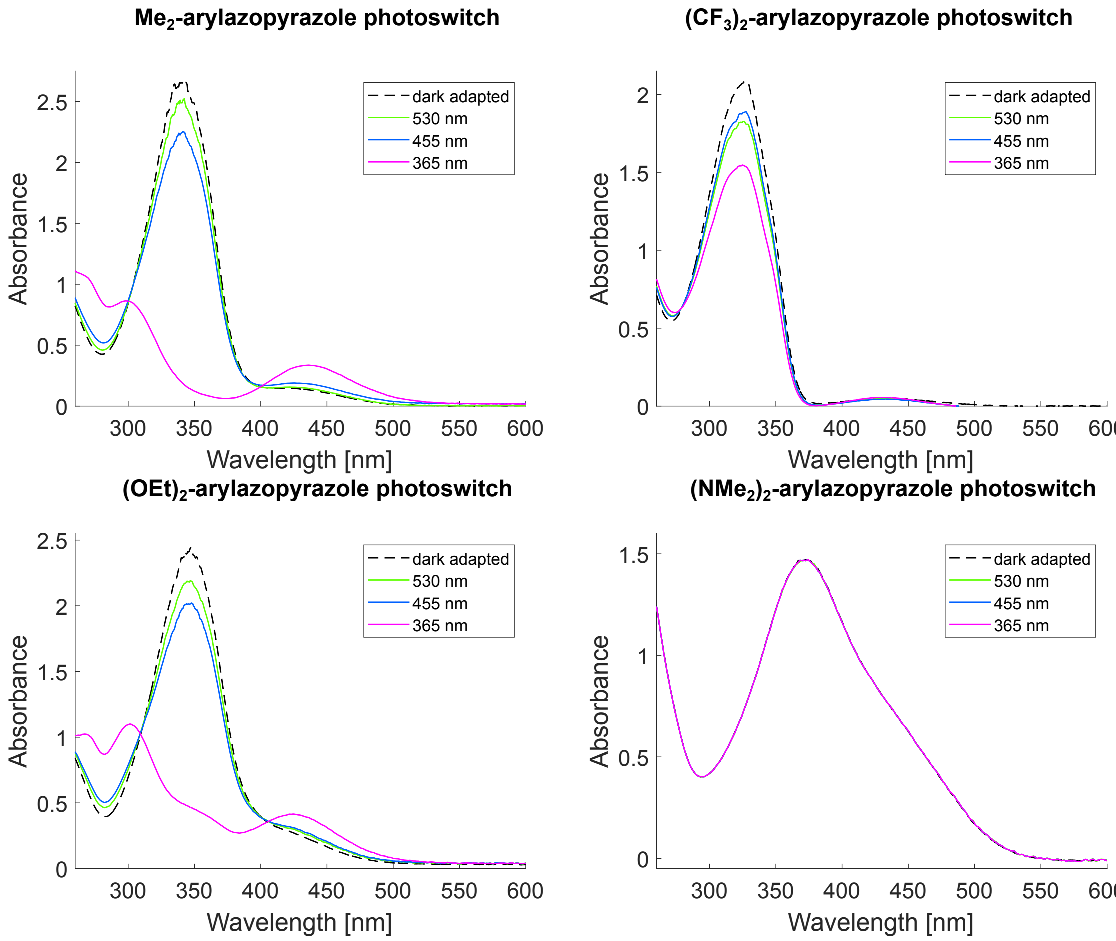

Fig. S20. UV-vis spectra of arylazopyrazole photoswitches in DMSO–buffer (1:1) after irradiation at 365, 455, 530 nm for 20 min.

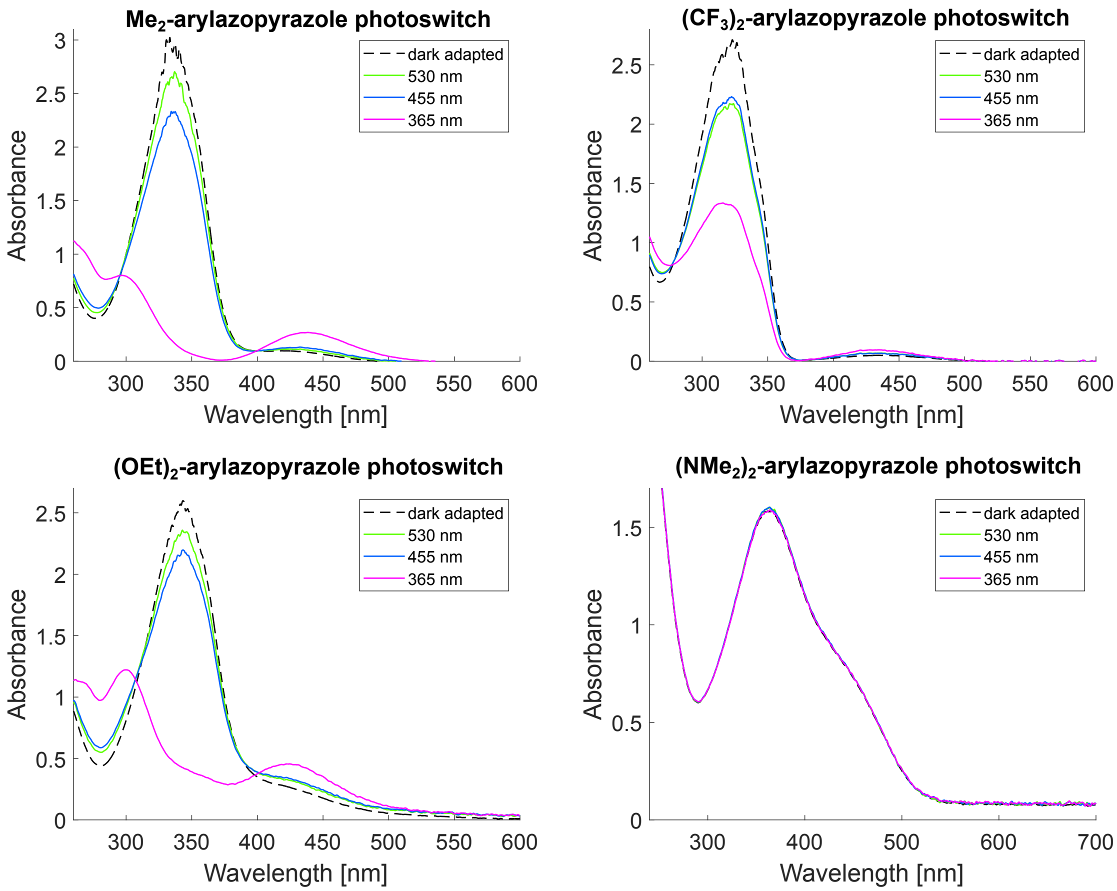

Fig. S21: UV-vis spectra of arylazopyrazole photoswitches in MeOH after irradiation at 365, 455, 530 nm for 20 min.

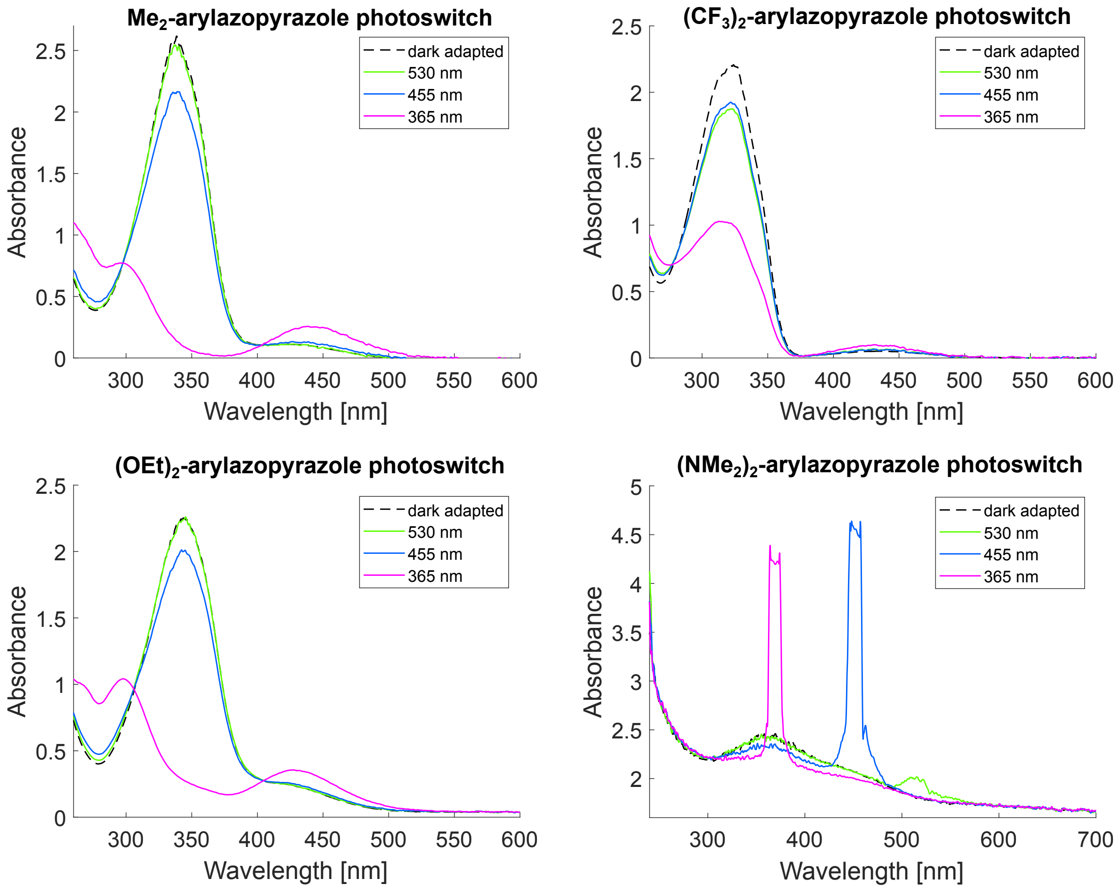

Fig. S22. UV-vis spectra of arylazopyrazole photoswitches in MeCN after irradiation at 365, 455, 530 nm for 20 min.

Thermal Relaxation of photoswitches

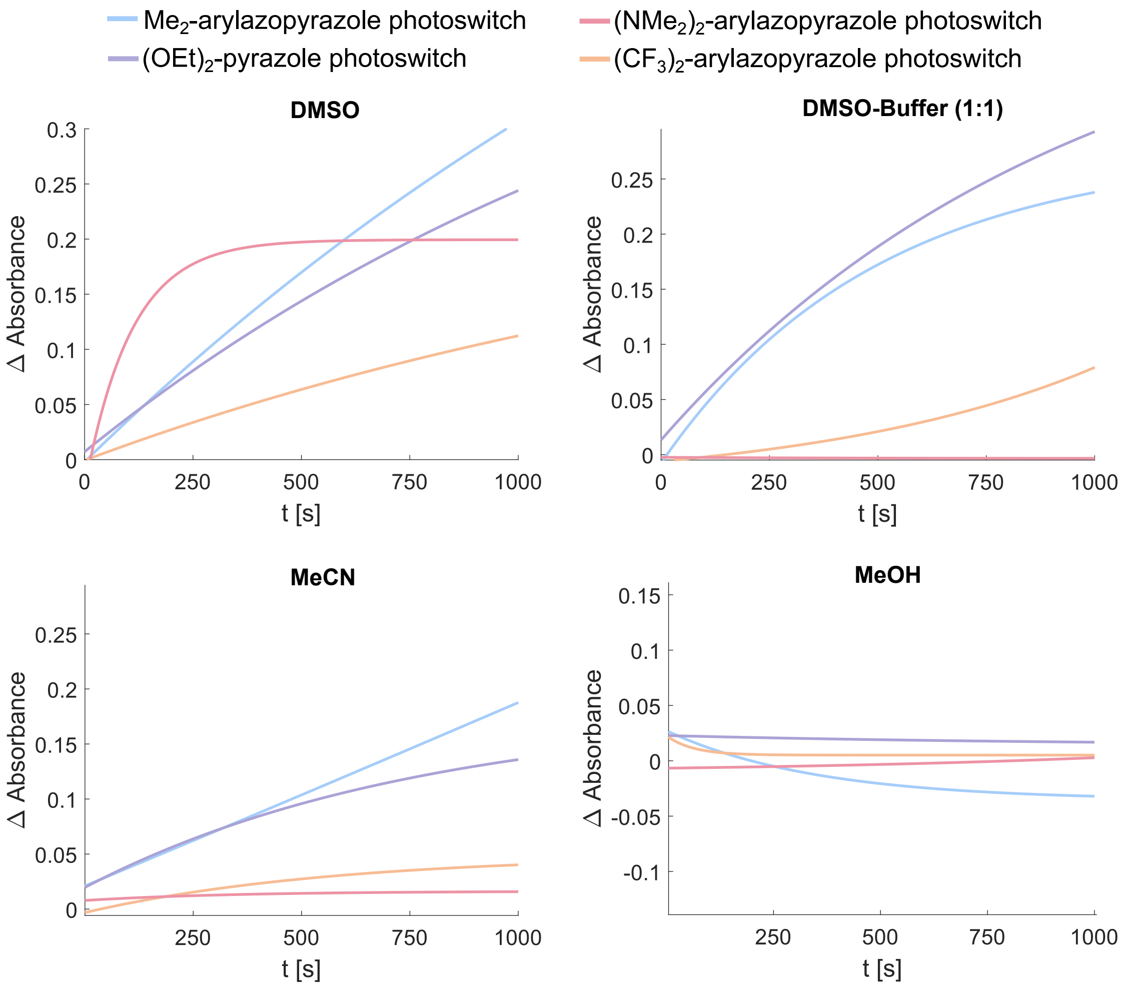

Fig. S23. Overview of solvent effects on thermal relaxation. The fitting functions of the thermal relaxations of arylazopyrazoles in DMSO, DMSO–buffer (1:1), MeCN and MeOH following irradiation at 365 nm for 20 min are shown. The change in absorbance was measured every 2 s over a period of 1000 s at 25 °C.

PSS Determination by HPLC Analysis

HPLC Traces for KH-5-169

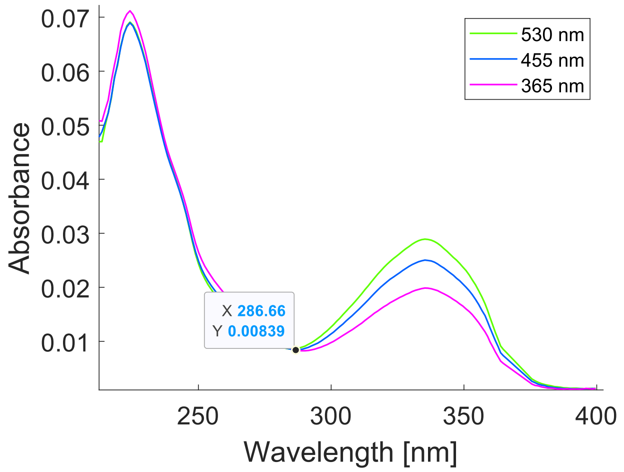

Fig. S24: Isosbestic wavelength at 287 nm for KH-5-169 in HPLC conditions (55% water in MeCN containing 0.1% formic acid). UV-vis traces were extracted from the chromatograms after irradiation at 365, 455 and 530 nm (at the isosbestic wavelength of 287 nm in DMSO–water (2:1))) for each isomer and combined to provide the UV-vis spectra for the isomeric mixture. Isocratic HPLC conditions (ReprosilChiral-NR column, 55% water in MeCN containing 0.1% formic acid).

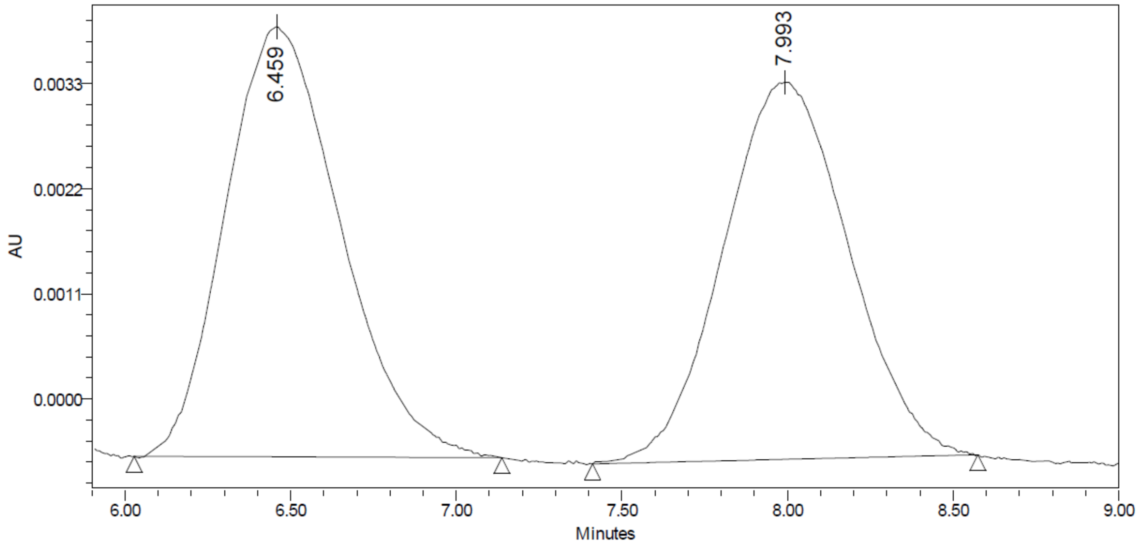

Peak Results

	Name	RT	Height	Area	% Area
1		6.459	4496	79157	38.64
2		7.993	3942	125683	61.36

Fig. S25. HPLC chromatogram of KH-5-169 (100 uM in DMSO–water (2:1)) following irradiation at 340 nm for 30 min. Integrals were determined at the isosbestic point at 287 nm. Isocratic HPLC conditions: ReprosilChiral-NR column, 55% water in MeCN (0.1% formic acid).

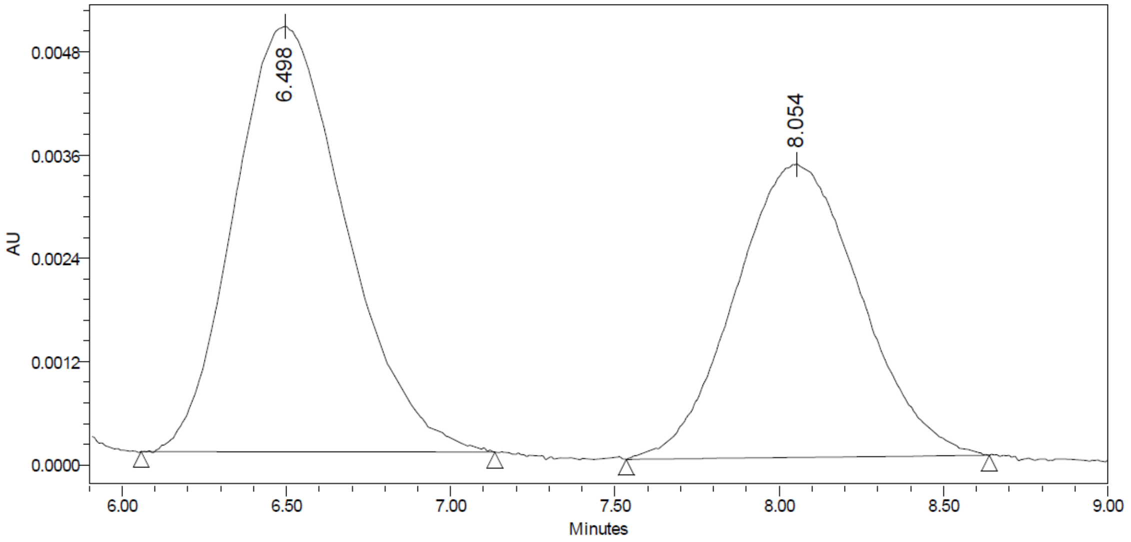

Peak Results

	Name	RT	Height	Area	% Area
1		6.498	4943	105888	43.54
2		8.054	3409	137321	56.46

Fig. S26. HPLC chromatogram of KH-5-169 (100 uM in DMSO–water (2:1)) following irradiation at 365 nm for 20 min. Integrals were determined at the isosbestic point at 287 nm. Isocratic HPLC conditions: ReprosilChiral-NR column, 55% water in MeCN (0.1% formic acid).

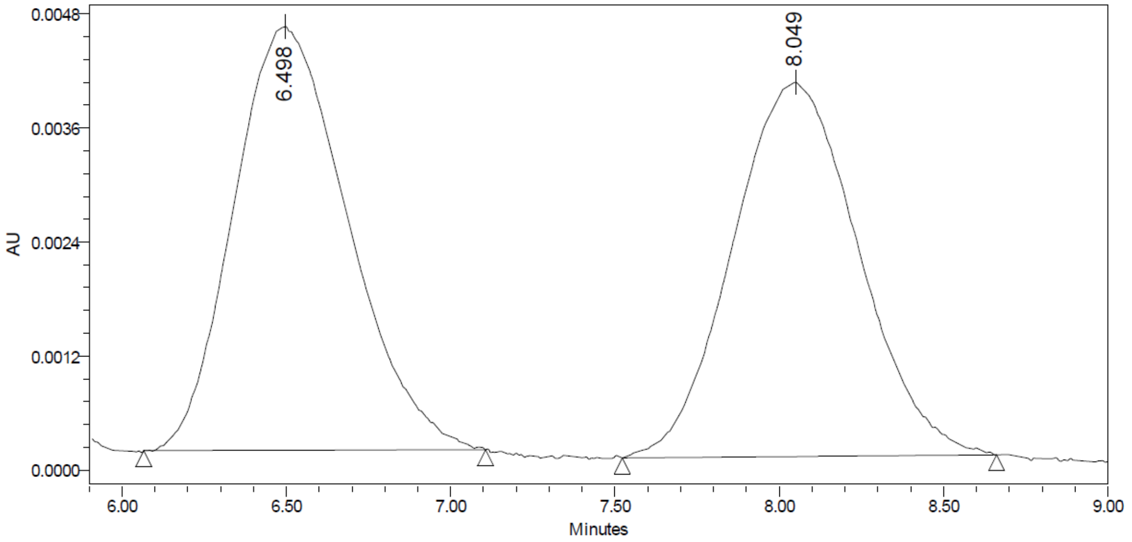

Peak Results

	Name	RT	Height	Area	% Area
1		6.498	4460	95124	48.56
2		8.049	3937	100759	51.44

Fig. S27. HPLC chromatogram of KH-5-169 (100 uM in DMSO–water (2:1)) following irradiation at 415 nm for 20 min. Integrals were determined at the isosbestic wavelength (287 nm for 55% water in MeCN (0.1% formic acid)). Isocratic HPLC conditions: ReprosilChiral-NR column, 55% water in MeCN (0.1% formic acid).

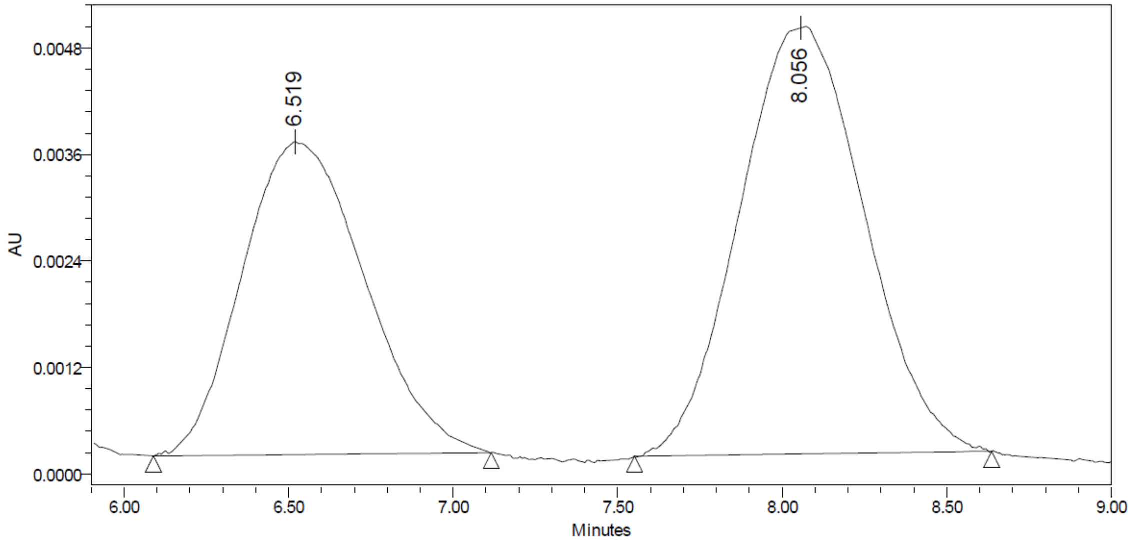

Peak Results

	Name	RT	Height	Area	% Area
1		6.519	3525	93325	42.96
2		8.056	4834	123921	57.04

Fig. S28. HPLC chromatogram of KH-5-169 (100 uM in DMSO–water (2:1)) following irradiation at 455 nm for 20 min. Integrals were determined at the isosbestic point at 287 nm. Isocratic HPLC conditions: ReprosilChiral-NR column, 55% water in MeCN (0.1% formic acid).

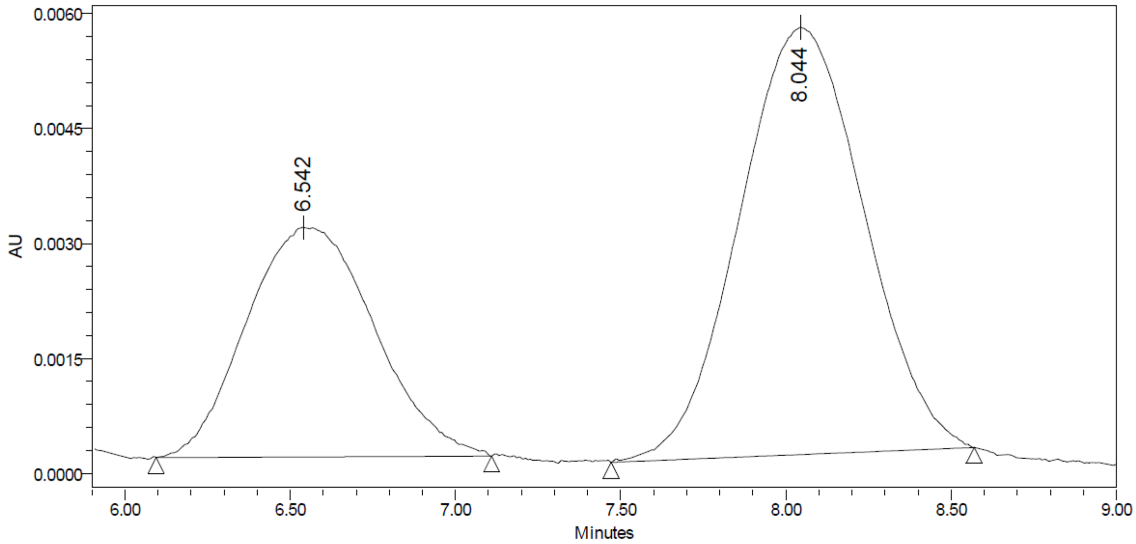

Peak Results

	Name	RT	Height	Area	% Area
1		6.542	2995	87588	33.74
2		8.044	5575	172026	66.26

Fig. S29. HPLC chromatogram of KH-5-169 (100 uM in DMSO–water (2:1)) following irradiation at 505 nm for 20 min. Integrals were determined at the isosbestic point at 287 nm. Isocratic HPLC conditions: ReprosilChiral-NR column, 55% water in MeCN (0.1% formic acid).

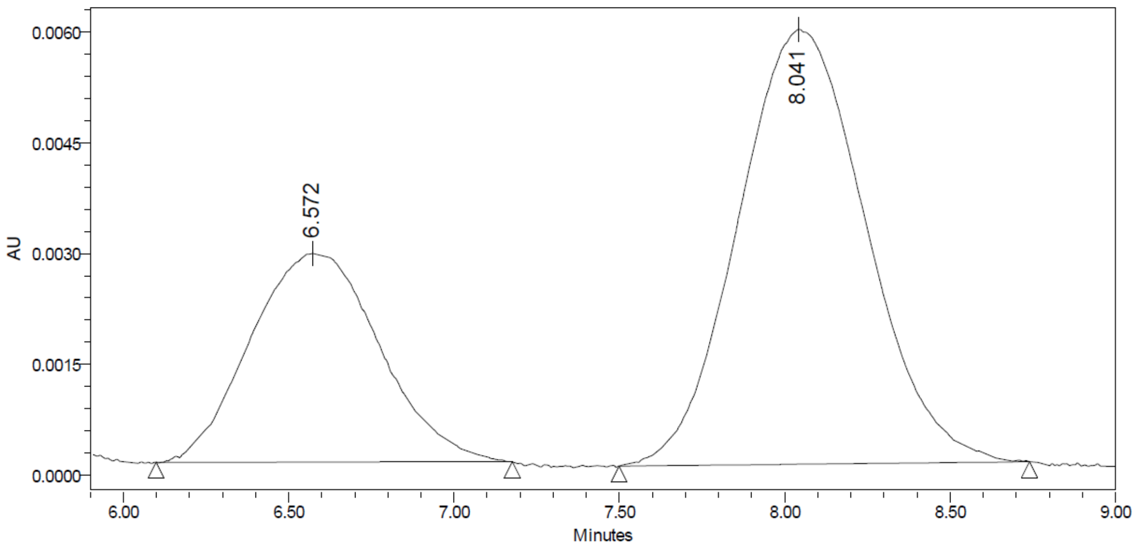

Peak Results

	Name	RT	Height	Area	% Area
1		6.572	2827	77186	29.97
2		8.041	5896	180378	70.03

Fig. S30. HPLC chromatogram of KH-5-169 (100 uM in DMSO–water (2:1)) following irradiation at 530 nm for 20 min. Integrals were determined at the isosbestic point at 287 nm. Isocratic HPLC conditions: ReprosilChiral-NR column, 55% water in MeCN (0.1% formic acid).

HPLC Traces for KH-5-226

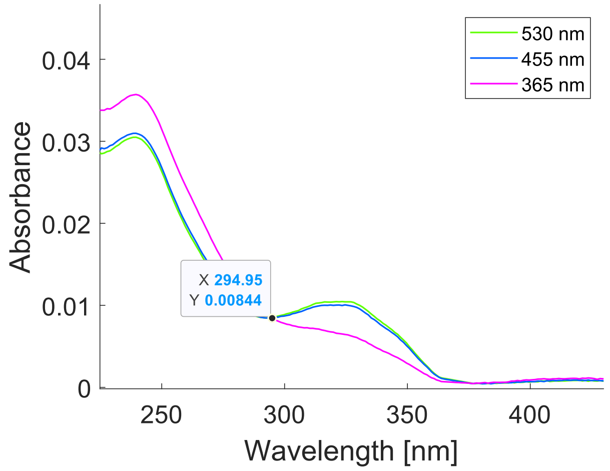

Fig. S31. Isosbestic wavelength at 295 nm for **KH-5-226** in HPLC conditions (60% water in MeCN containing 0.1% formic acid). UV-vis traces were extracted from the chromatograms following irradiation at 365, 455 and 530 nm (at the isosbestic wavelength of 290 nm in DMSO–water (2:1)) for each isomer and combined to provide the UV-vis spectra for the isomeric mixture. Isocratic HPLC conditions (ReprosilChiral-NR column, 60% water in MeCN containing 0.1% formic acid).

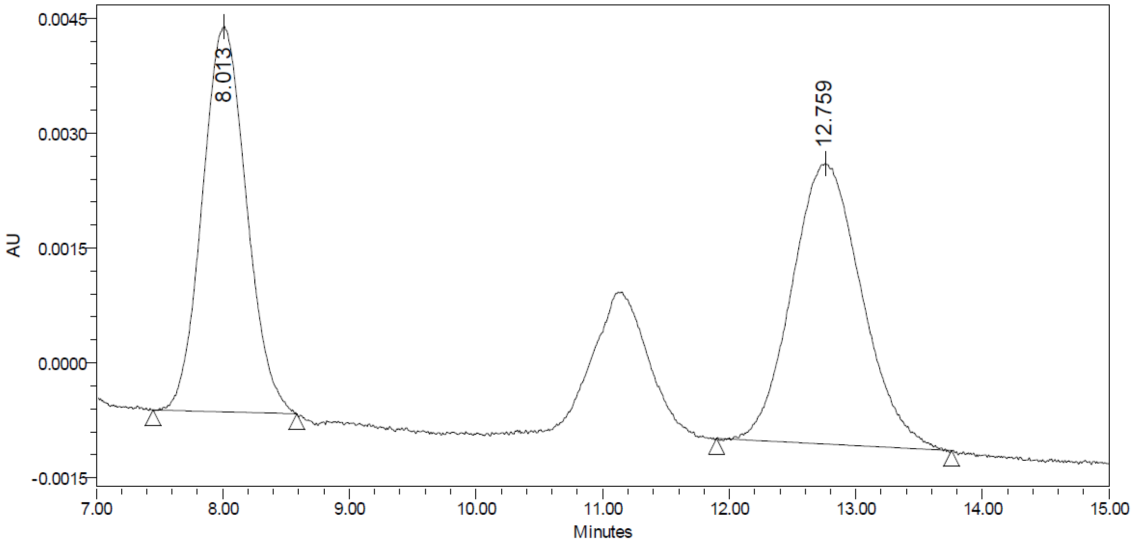

Peak Results

	Name	RT	Height	Area	% Area
1		8.013	5034	119947	45.93
2		12.759	3664	141218	54.07

Fig. S32. HPLC chromatogram of **KH-5-226** (100 uM in DMSO–water (2:1)) following irradiation at 340 nm for 30 min. Integrals were determined at the isosbestic point at 295 nm. Isocratic HPLC conditions: ReprosilChiral-NR column, 60% water in MeCN (0.1% formic acid).

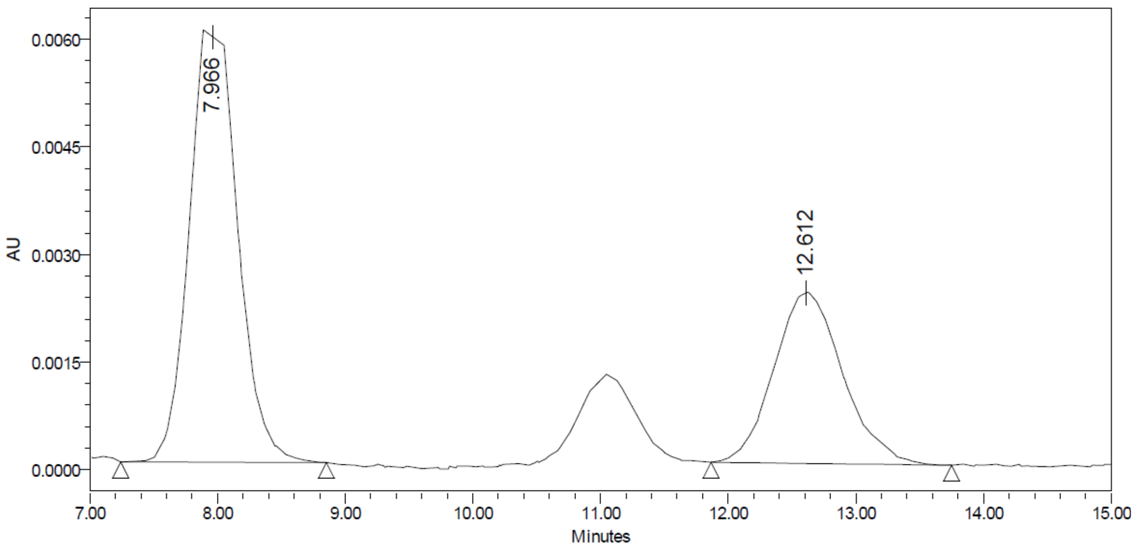

Peak Results

	Name	RT	Height	Area	% Area
1		7.966	6624	194373	67.66
2		12.612	2385	92926	32.34

Fig. S33. HPLC chromatogram of **KH-5-226** (100 uM in DMSO–water (2:1)) following irradiation at 365 nm for 20 min. Integrals were determined at the isosbestic point at 295 nm. Isocratic HPLC conditions: ReprosilChiral-NR column, 60% water in MeCN (0.1% formic acid).

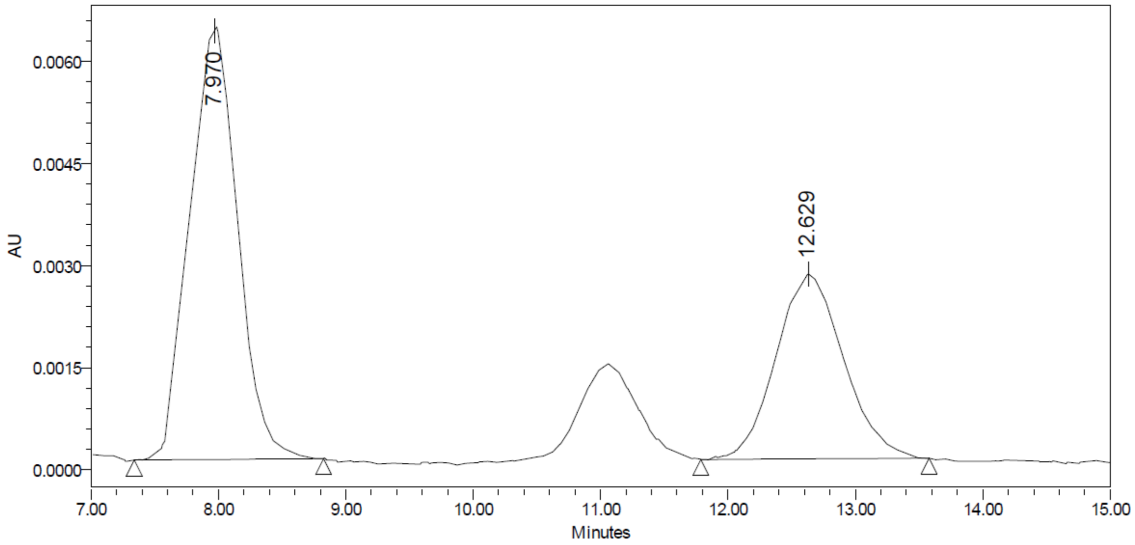

Peak Results

	Name	RT	Height	Area	% Area
1		7.970	6375	139669	53.58
2		12.629	2712	121017	46.42

Fig. S34. HPLC chromatogram of **KH-5-226** (100 uM in DMSO–water (2:1)) following irradiation at 415 nm for 20 min. Integrals were determined at the isosbestic point at 295 nm. Isocratic HPLC conditions: ReprosilChiral-NR column, 60% water in MeCN (0.1% formic acid).

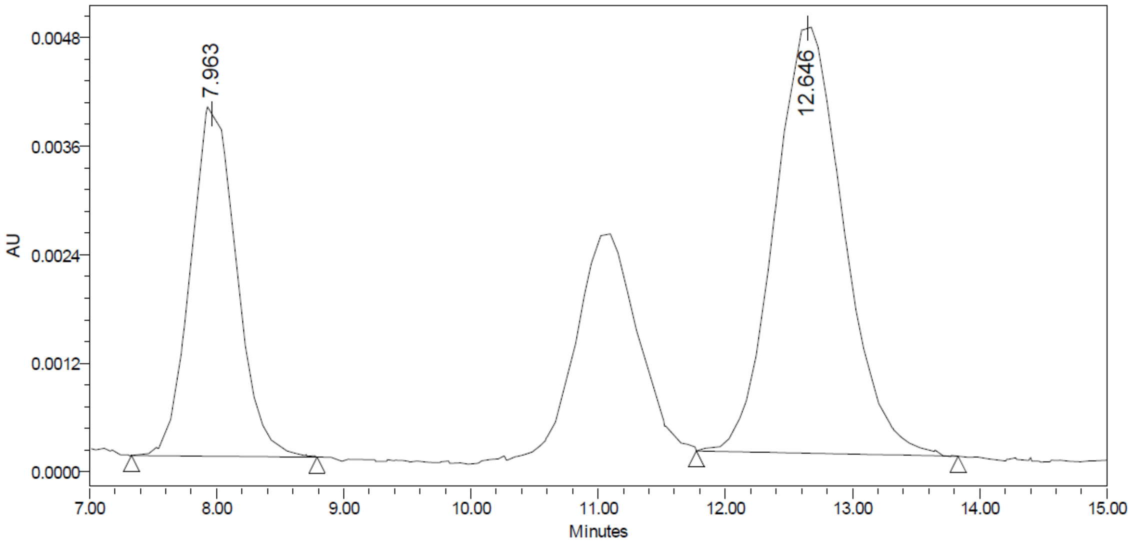

Peak Results

	Name	RT	Height	Area	% Area
1		7.963	3912	68923	29.95
2		12.646	4742	161200	70.05

Fig. S35. HPLC chromatogram of **KH-5-226** (100 uM in DMSO–water (2:1)) following irradiation at 455 nm for 20 min. Integrals were determined at the isosbestic point at 295 nm. Isocratic HPLC conditions: ReprosilChiral-NR column, 60% water in MeCN (0.1% formic acid).

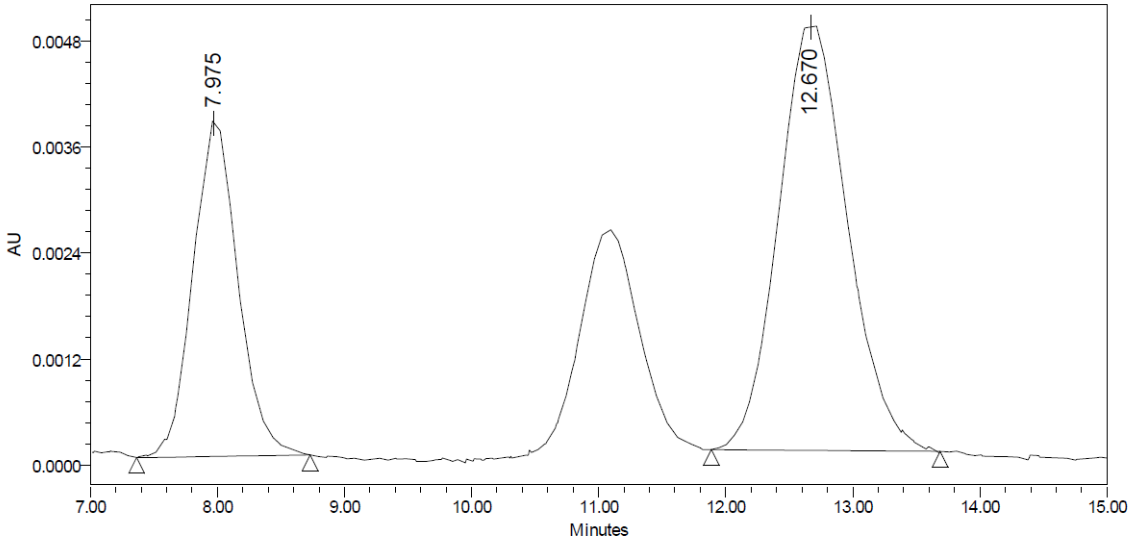

Peak Results

	Name	RT	Height	Area	% Area
1		7.975	3716	65931	27.22
2		12.670	4857	176247	72.78

Fig. S36. HPLC chromatogram of **KH-5-226** (100 uM in DMSO–water (2:1)) following irradiation at 505 nm for 20 min. Integrals were determined at the isosbestic point at 295 nm. Isocratic HPLC conditions: ReprosilChiral-NR column, 60% water in MeCN (0.1% formic acid).

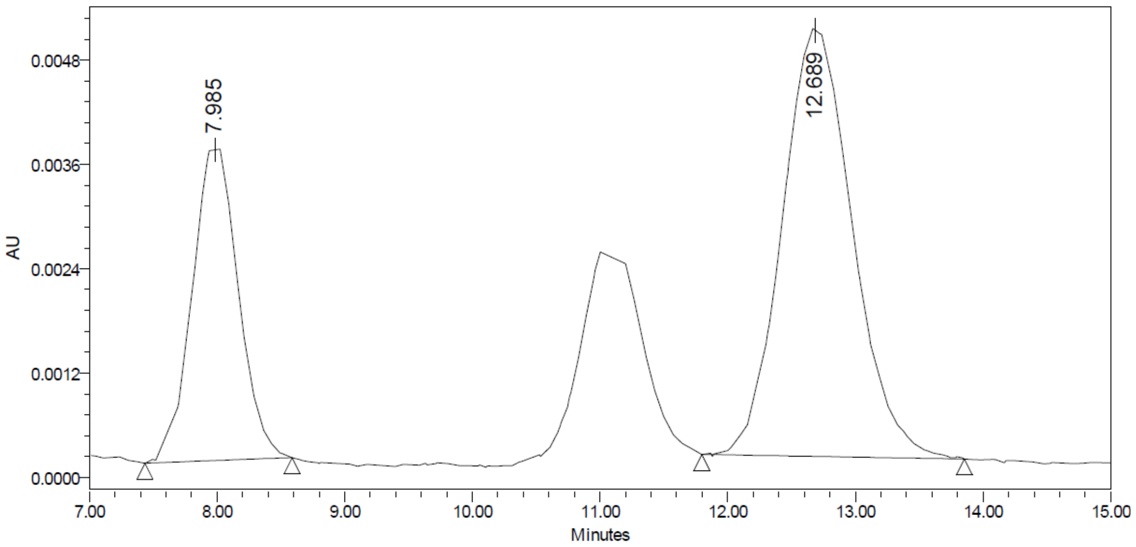

Peak Results

	Name	RT	Height	Area	% Area
1		7.985	3686	110338	34.53
2		12.689	4927	209173	65.47

Fig. S37. HPLC chromatogram of **KH-5-226** (100 uM in DMSO–water (2:1)) following irradiation at 530 nm for 20 min. Integrals were determined at the isosbestic point at 295 nm. Isocratic HPLC conditions: ReprosilChiral-NR column, 60% water in MeCN (0.1% formic acid).

HPLC Traces for KH-5-298

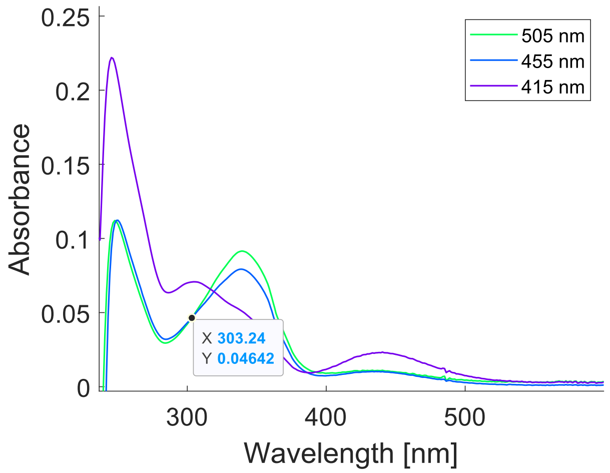

Fig. S38. Isosbestic wavelength at 303 nm for **KH-5-298** in HPLC conditions (5% water in MeCN containing 0.1% formic acid). UV-vis traces were extracted from the chromatograms following irradiation at 415, 455 and 505 nm (at the isosbestic wavelength of 305 nm in DMSO) for each isomer and combined to provide the UV-vis spectra for the isomeric mixture. Isocratic HPLC conditions (ReprosilChiral-NR column, 5% water in MeCN containing 0.1% formic acid).

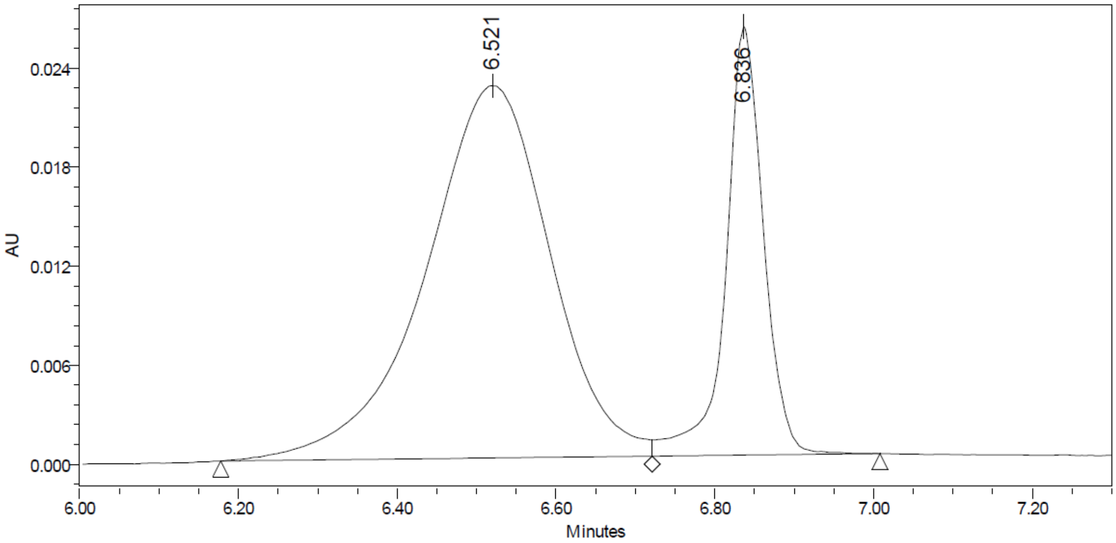

Peak Results

	Name	RT	Height	Area	% Area
1		6.521	22560	261705	69.85
2		6.836	25961	112983	30.15

Fig. S39. HPLC chromatogram of **KH-5-298** (100 uM in DMSO) following irradiation at 340 nm for 20 min. Integrals were determined at the isosbestic point at 303 nm. Isocratic HPLC conditions: ReprosilChiral-NR column, 5% water in MeCN (0.1% formic acid).

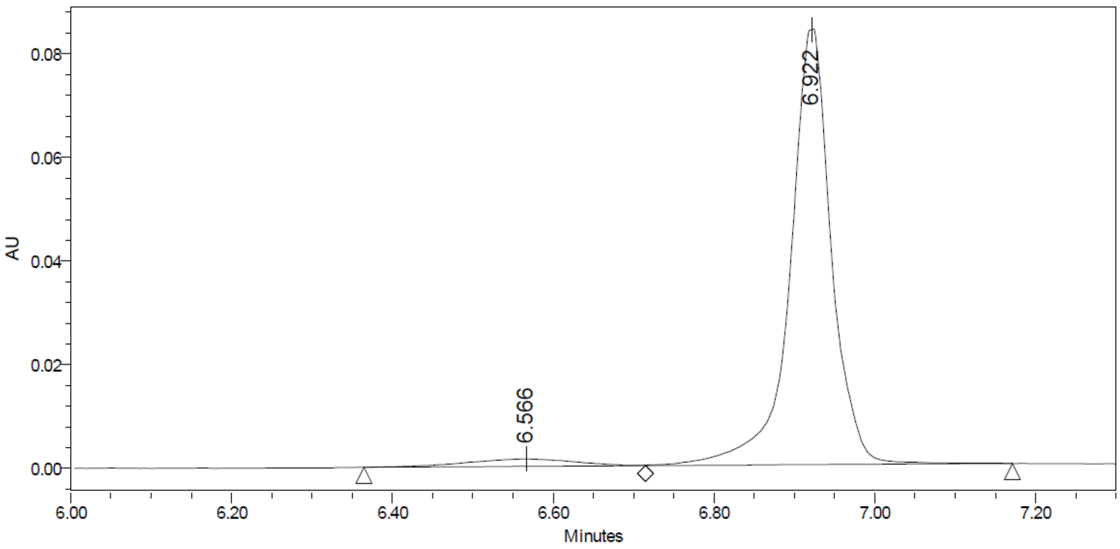

Peak Results

	Name	RT	Height	Area	% Area
1		6.566	1384	13214	4.56
2		6.922	84952	276824	95.44

Fig. S40. HPLC chromatogram of **KH-5-298** (100 uM in DMSO) following irradiation at 365 nm for 20 min. Integrals were determined at the isosbestic point at 303 nm. Isocratic HPLC conditions: ReprosilChiral-NR column, 5% water in MeCN (0.1% formic acid).

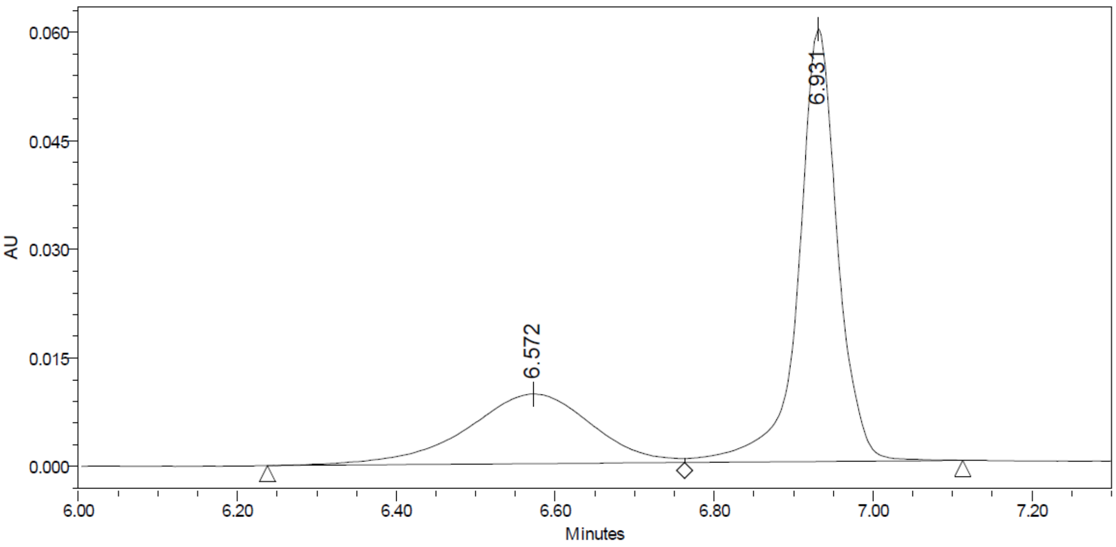

Peak Results

	Name	RT	Height	Area	% Area
1		6.572	9615	106467	29.09
2		6.931	59818	259496	70.91

Fig. S41. HPLC chromatogram of **KH-5-298** (100 uM in DMSO) following irradiation at 415 nm for 20 min. Integrals were determined at the isosbestic point at 303 nm. Isocratic HPLC conditions: ReprosilChiral-NR column, 5% water in MeCN (0.1% formic acid).

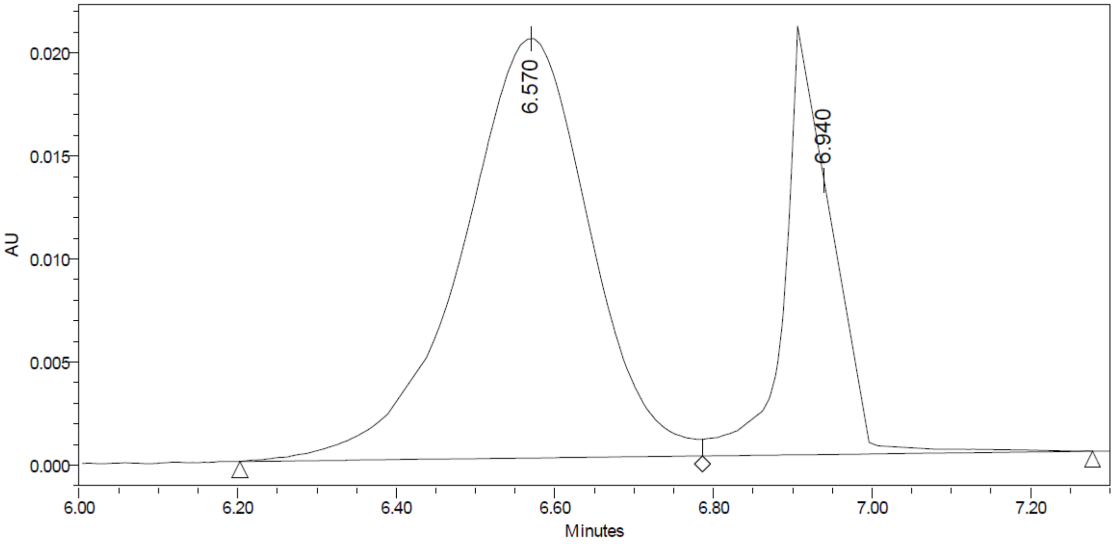

Peak Results

	Name	RT	Height	Area	% Area
1		6.570	20370	210941	86.71
2		6.940	30263	32322	13.29

Fig. S42. HPLC chromatogram of **KH-5-298** (100 uM in DMSO) following irradiation at 455 nm for 20 min. Integrals were determined at the isosbestic point at 303 nm. Isocratic HPLC conditions: ReprosilChiral-NR column, 5% water in MeCN (0.1% formic acid).

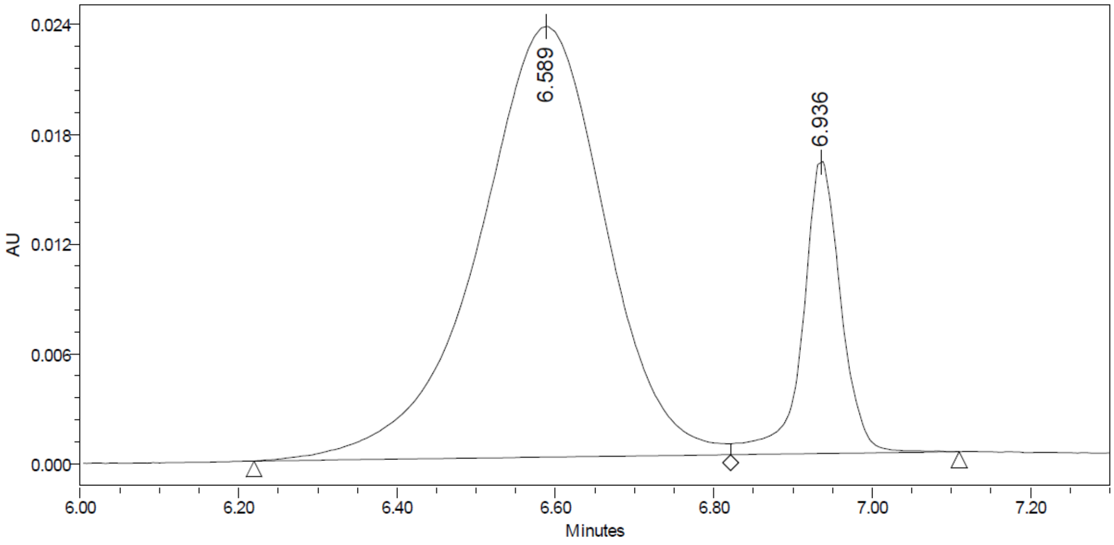

Peak Results

	Name	RT	Height	Area	% Area
1		6.589	23551	224249	82.68
2		6.936	16056	46987	17.32

Fig. S43. HPLC chromatogram of **KH-5-298** (100 uM in DMSO) following irradiation at 505 nm for 20 min. Integrals were determined at the isosbestic point at 303 nm. Isocratic HPLC conditions: ReprosilChiral-NR column, 5% water in MeCN (0.1% formic acid).

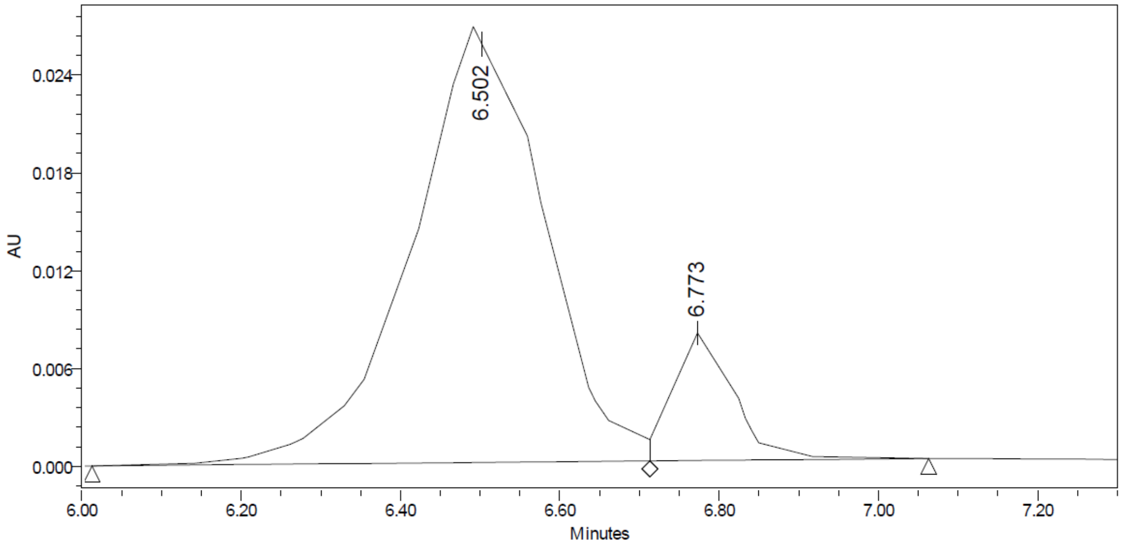

Peak Results

	Name	RT	Height	Area	% Area
1		6.502	26393	410756	88.85
2		6.773	7819	51531	11.15

Fig. S44. HPLC chromatogram of **KH-5-298** (100 uM in DMSO) following irradiation at 530 nm for 20 min. Integrals were determined at the isosbestic point at 303 nm. Isocratic HPLC conditions: ReprosilChiral-NR column, 5% water in MeCN (0.1% formic acid).

HPLC Traces for KH-5-327

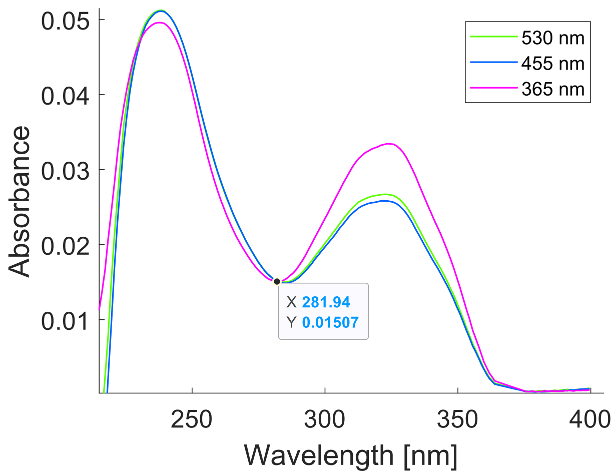

Fig. S45. Isosbestic wavelength at 282 nm for **KH-5-327** in HPLC conditions (55% water in MeCN containing 0.1% formic acid). UV-vis traces were extracted from the chromatograms following irradiation at 365, 455 and 530 nm (at the isosbestic wavelength of 282 nm in DMSO) for each isomer and combined to provide the UV-vis spectra for the isomeric mixture. Isocratic HPLC conditions (ReprosilChiral-NR column, 55% water in MeCN containing 0.1% formic acid).

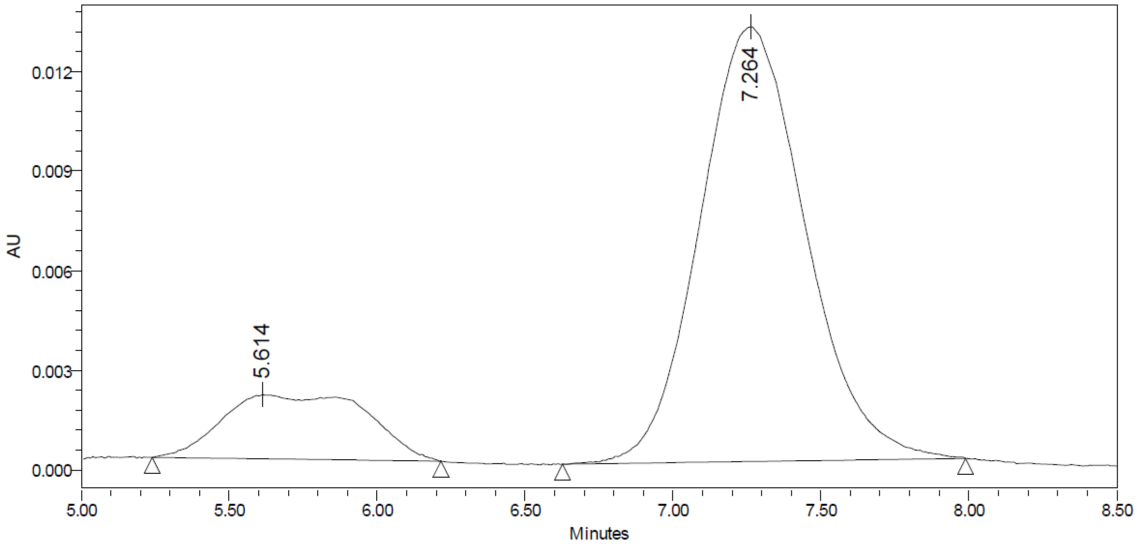

Peak Results

	Name	RT	Height	Area	% Area
1		5.614	1931	65386	18.46
2		7.264	13089	288766	81.54

Fig. S46. HPLC chromatogram of **KH-5-327** (100 uM in DMSO) following irradiation at 340 nm for 30 min. Integrals were determined at the isosbestic point at 282 nm. Isocratic HPLC conditions: ReprosilChiral-NR column, 55% water in MeCN (0.1% formic acid).

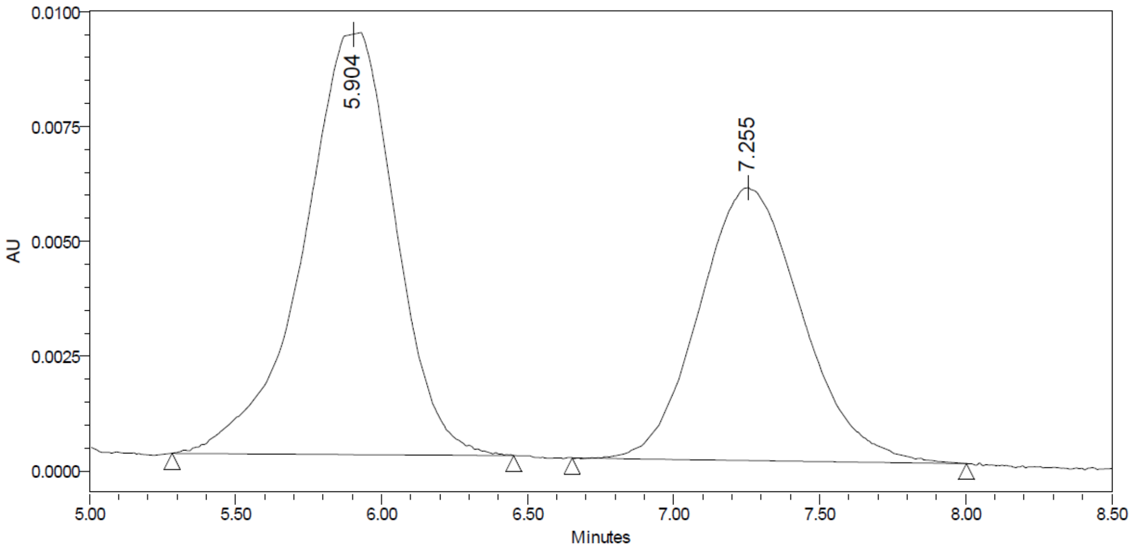

Peak Results

	Name	RT	Height	Area	% Area
1		5.904	9375	176196	54.96
2		7.255	5941	144405	45.04

Fig. S47. HPLC chromatogram of **KH-5-327** (100 uM in DMSO) following irradiation at 365 nm for 20 min. Integrals were determined at the isosbestic point at 282 nm. Isocratic HPLC conditions: ReprosilChiral-NR column, 55% water in MeCN (0.1% formic acid).

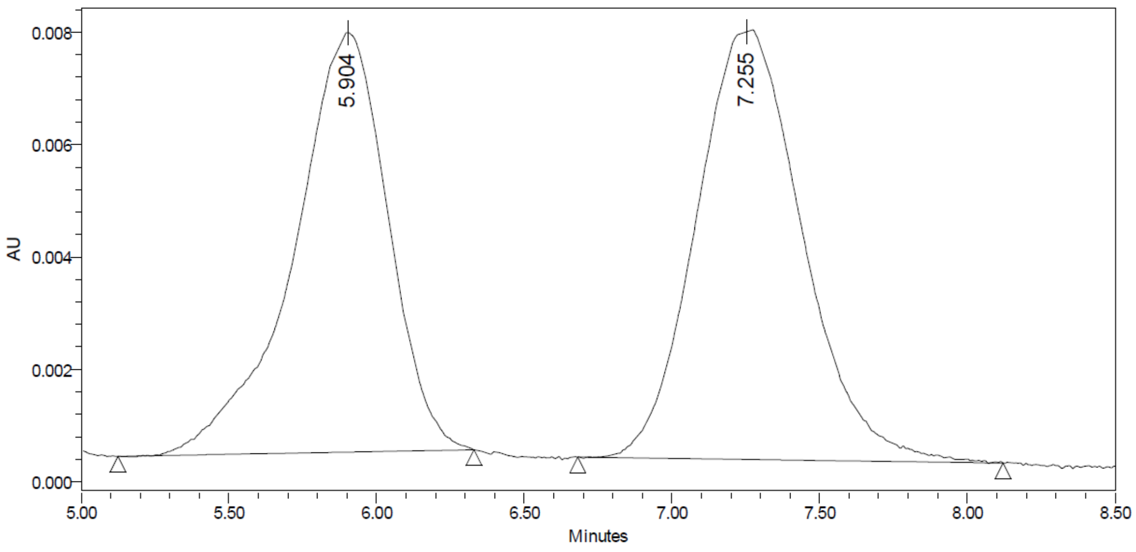

Peak Results

	Name	RT	Height	Area	% Area
1		5.904	7462	144453	43.14
2		7.255	7694	190379	56.86

Fig. S48. HPLC chromatogram of **KH-5-327** (100 uM in DMSO) following irradiation at 415 nm for 20 min. Integrals were determined at the isosbestic point at 282 nm. Isocratic HPLC conditions: ReprosilChiral-NR column, 55% water in MeCN (0.1% formic acid).

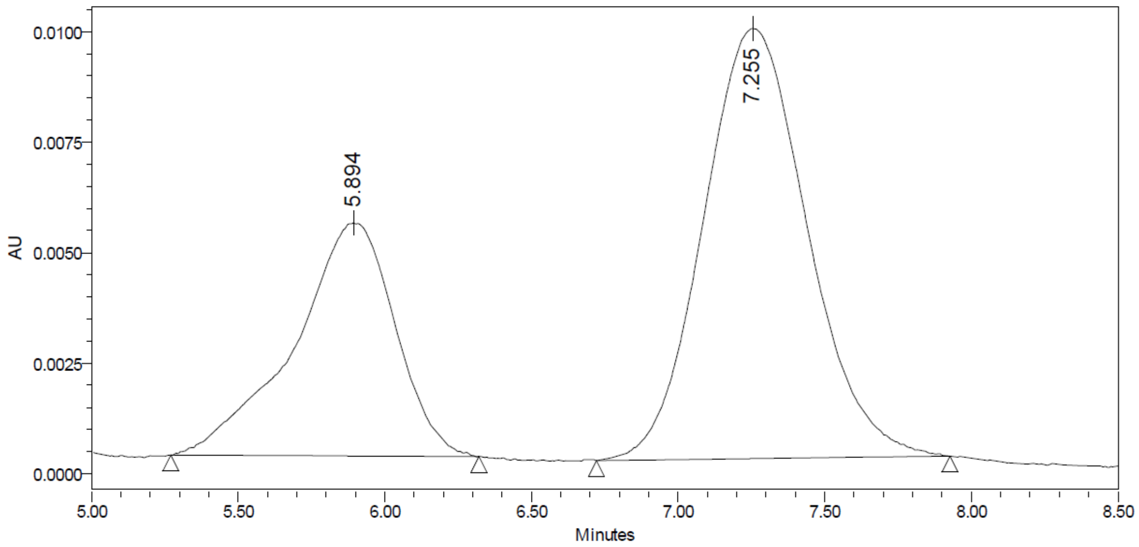

Peak Results

	Name	RT	Height	Area	% Area
1		5.894	5270	127504	32.63
2		7.255	9730	263300	67.37

Fig. S49. HPLC chromatogram of **KH-5-327** (100 uM in DMSO) following irradiation at 455 nm for 20 min. Integrals were determined at the isosbestic point at 282 nm. Isocratic HPLC conditions: ReprosilChiral-NR column, 55% water in MeCN (0.1% formic acid).

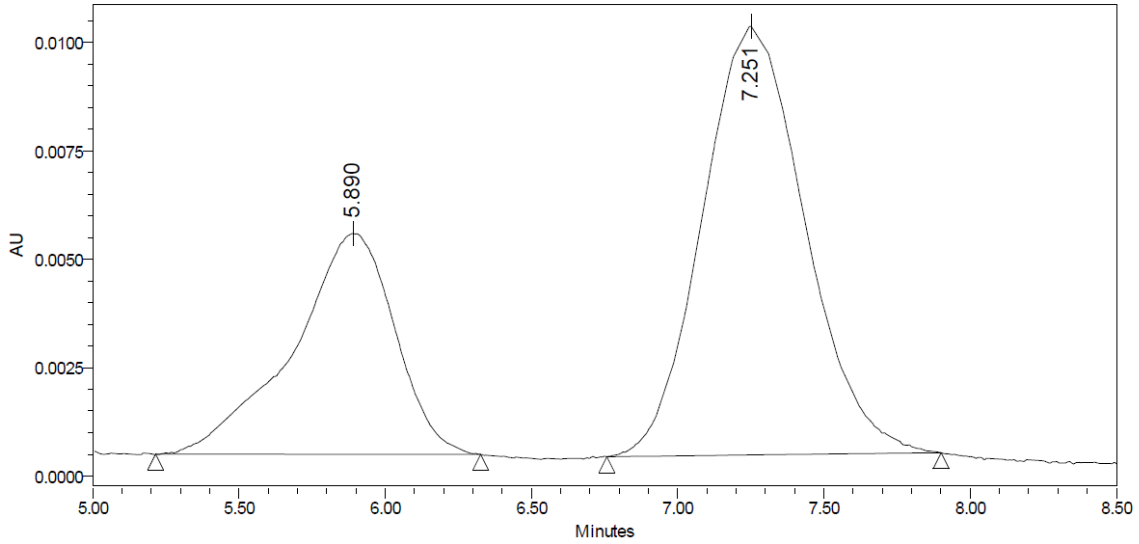

Peak Results

	Name	RT	Height	Area	% Area
1		5.890	5091	158799	59.59
2		7.251	9892	107669	40.41

Fig. S50. HPLC chromatogram of **KH-5-327** (100 uM in DMSO) following irradiation at 505 nm for 20 min. Integrals were determined at the isosbestic point at 282 nm. Isocratic HPLC conditions: ReprosilChiral-NR column, 55% water in MeCN (0.1% formic acid).

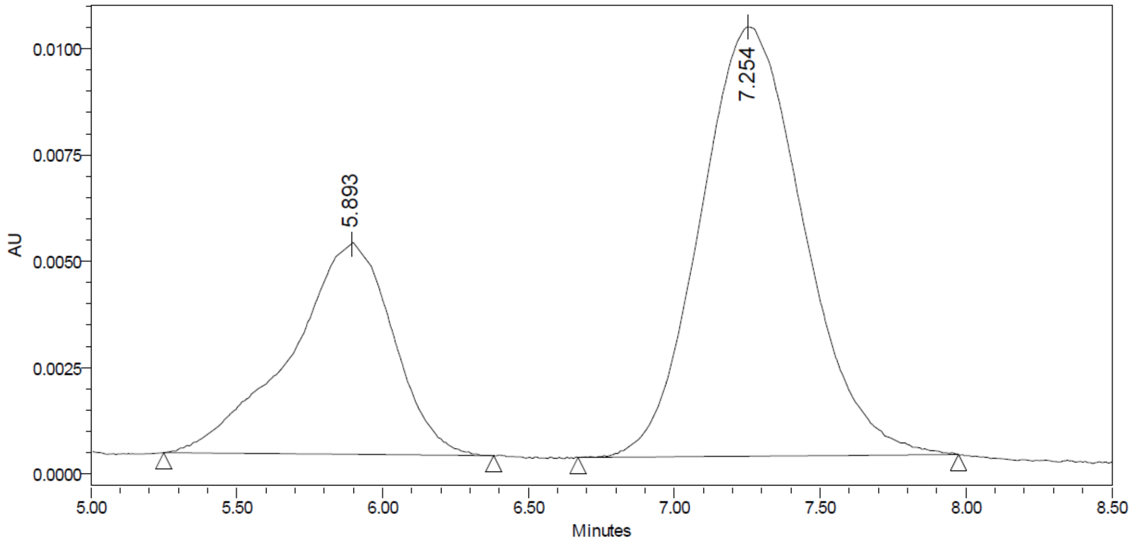

Peak Results

	Name	RT	Height	Area	% Area
1		5.893	4990	112798	28.90
2		7.254	10100	277462	71.10

Fig. S51. HPLC chromatogram of **KH-5-327** (100 uM in DMSO) following irradiation at 530 nm for 20 min. Integrals were determined at the isosbestic point at 282 nm. Isocratic HPLC conditions: ReprosilChiral-NR column, 55% water in MeCN (0.1% formic acid).

PSS Determination by NMR Spectroscopy

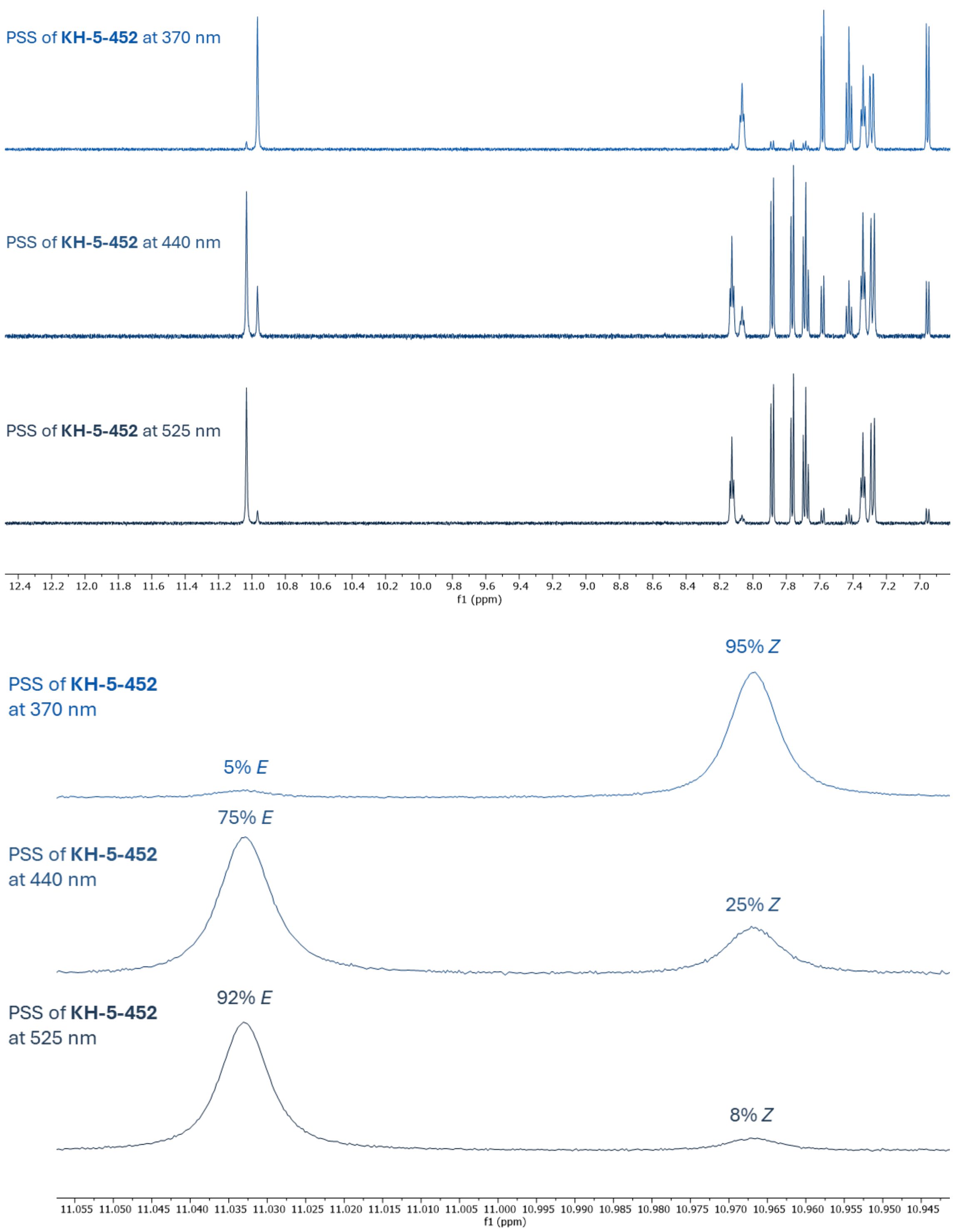

Fig. S52. Upper: ^{1}H NMR spectrum of (OEt)$_2$-arylazopyrazole photoPROTAC **KH-5-452** after reaching the PSS upon irradiation at 370, 440 and 525 nm for 30 min. Lower: ^{1}H NMR signal for quantification of the ratio between *E*- and *Z*-**KH-5-452**.

Photophysical properties of Me_2-arylazopyrazole photoPROTAC

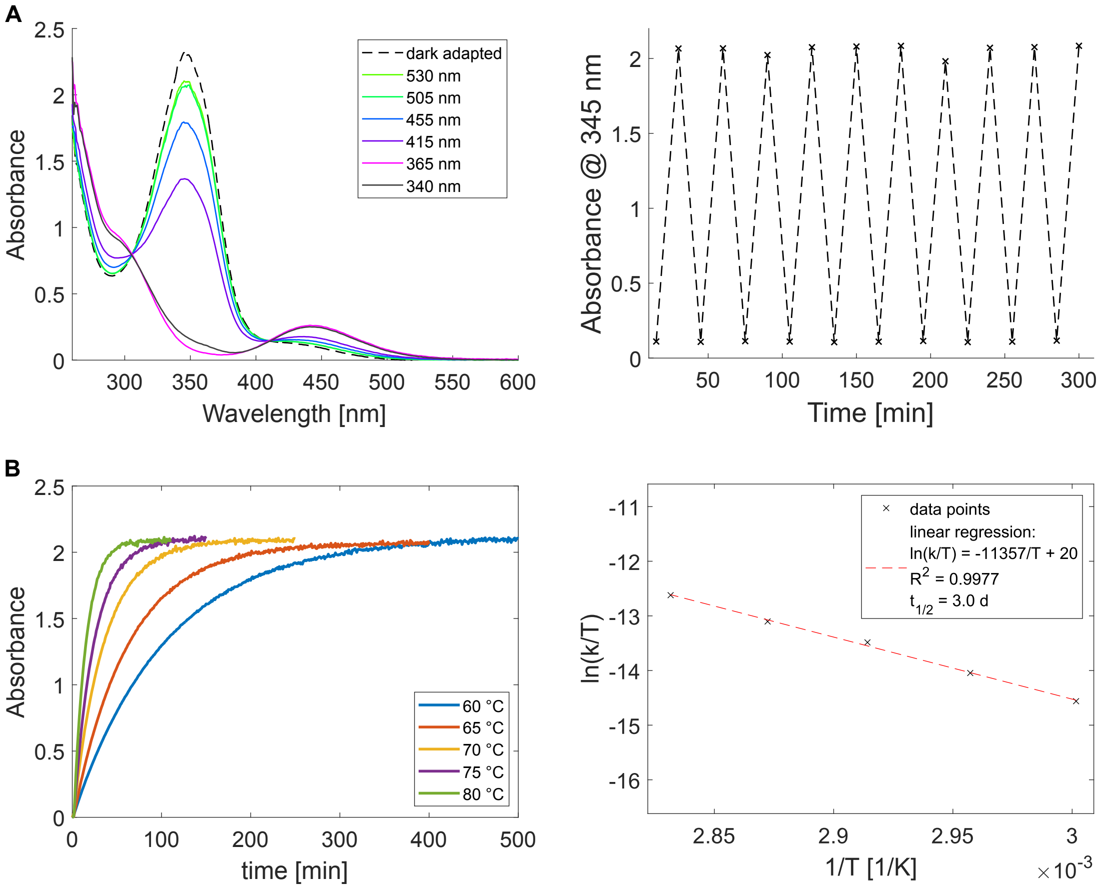

Fig. S53. Photophysical properties of GR Me_2-arylazopyrazole photoPROTAC **KH-5-309**. A) *Left*: UV-vis spectrum in DMSO (100 µM) irradiated for 30 min (340 nm) or 20 min (365–530 nm) with a π–π* λmax of 347 nm. *Right*: Photostability of photoPROTAC **KH-5-309** over a time interval of 300 min. Absorbance at 345 nm of photoPROTAC **KH-5-309** in DMSO (100 µM) was measured following alternating irradiation at 365 nm and 530 nm for 15 min. B) Measurements for the thermal relaxation of **KH-5-309**. *Left*: Thermal relaxation of photoPROTAC **KH-5-309** in DMSO (100 µM) at varying temperatures. After irradiation at 365 nm for 20 min, the absorbance at 345 nm was measured over the temperature range of 60–80 °C. *Right*: Eyring plot for photoPROTAC **KH-5-309**.

Photophysical properties of $(OEt)_2$-arylazopyrazole photoPROTAC

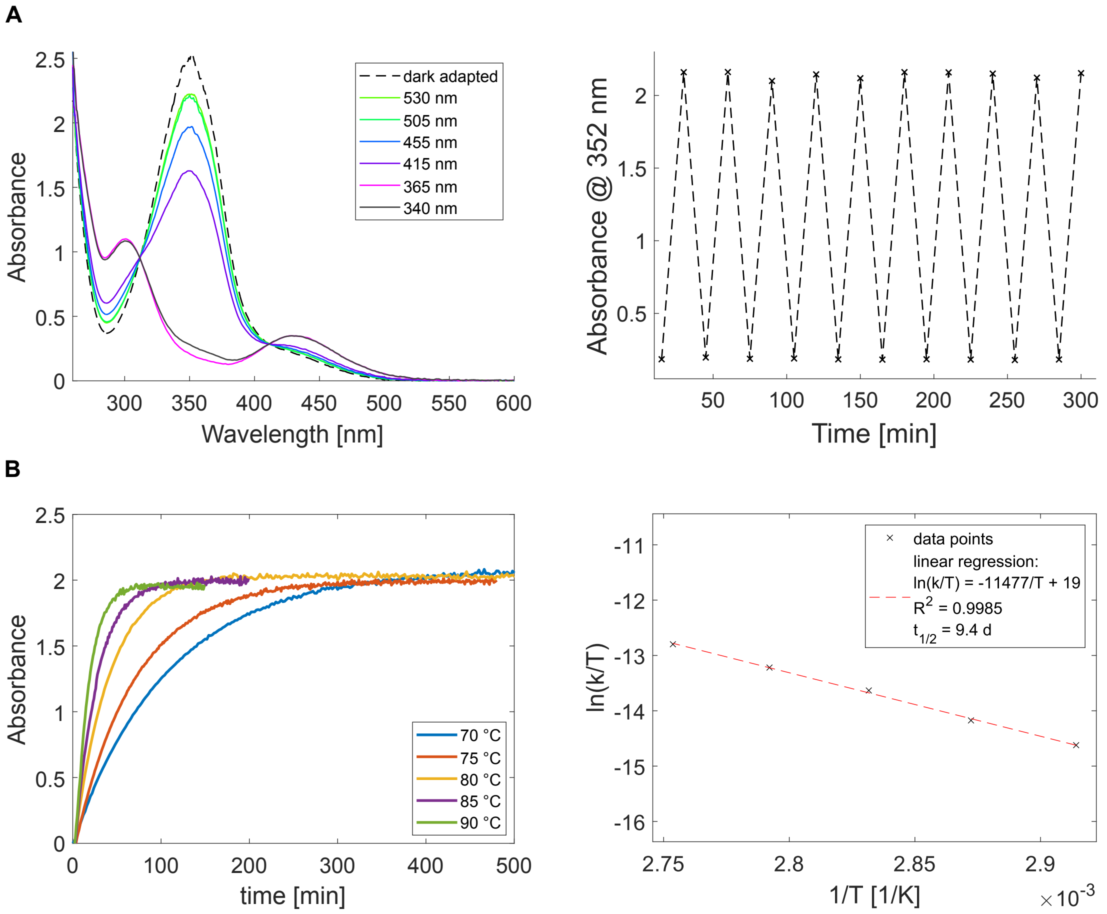

Fig. S54. Photophysical properties of GR $(OEt)_2$-arylazopyrazole photoPROTAC **KH-5-450**. A) *Left*: UV-vis spectrum in DMSO (100 µM) after being irradiated for 30 min (340 nm) or 20 min (365–530 nm) with a π–π* λmax of 352 nm. *Right*: Photostability of photoPROTAC **KH-5-450** over a time interval of 300 min. Absorbance at 352 nm of photoPROTAC **KH-5-450** in DMSO (100 µM) was measured following alternating irradiation at 365 and 505 nm for 15 min. B) Measurements for the thermal relaxation of **KH-5-450**. *Left*: Thermal relaxation of photoPROTAC **KH-5-450** in DMSO (100 µM) at varying temperatures. After irradiation at 365 nm for 20 min, the absorbance at 352 nm was measured over the temperature range of 70–90 °C. *Right*: Eyring plot for photoPROTAC **KH-5-450**.

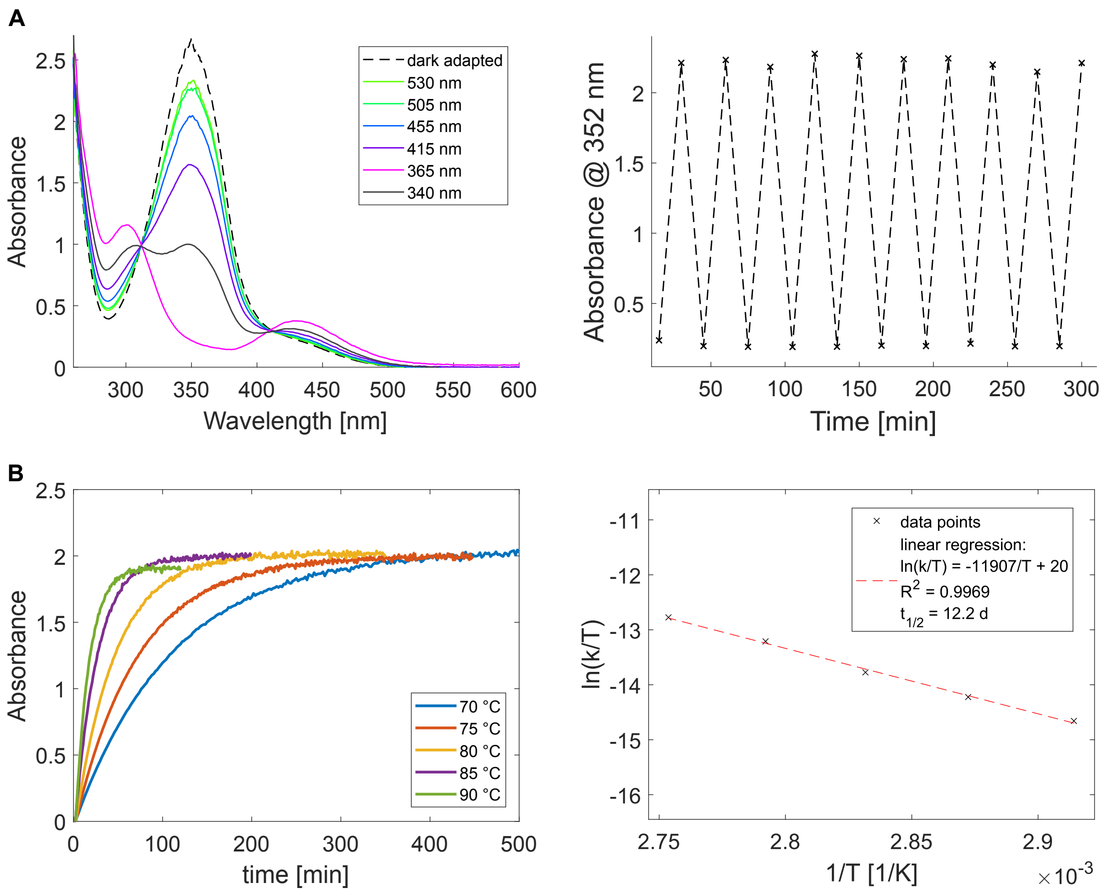

Fig. S55: Photophysical properties of GR $(OEt)_2$-arylazopyrazole photoPROTAC **KH-5-456**. A) *Left*: UV-vis spectrum in DMSO (100 µM) after irradiation for 30 min (340 nm) or 20 min (365–530 nm) with a π–π* λmax of 351 nm. *Right*: Photostability of photoPROTAC **KH-5-456** over a time interval of 300 min. Absorbance at 352 nm of photoPROTAC **KH-5-456** in DMSO (100 µM) was measured following alternating irradiation at 365 nm and 505 nm for 15 min. B) Measurements for the thermal relaxation of **KH-5-456**. *Left*: Thermal relaxation of photoPROTAC **KH-5-456** in DMSO (100 µM) at varying temperatures. After irradiation at 365 nm for 20 min, the absorbance at 345 nm was measured over the temperature range of 70–90 °C. *Right*: Eyring plot for photoPROTAC **KH-5-456**.

Photophysical properties of $(CF_3)_2$-arylazopyrazole photoPROTAC

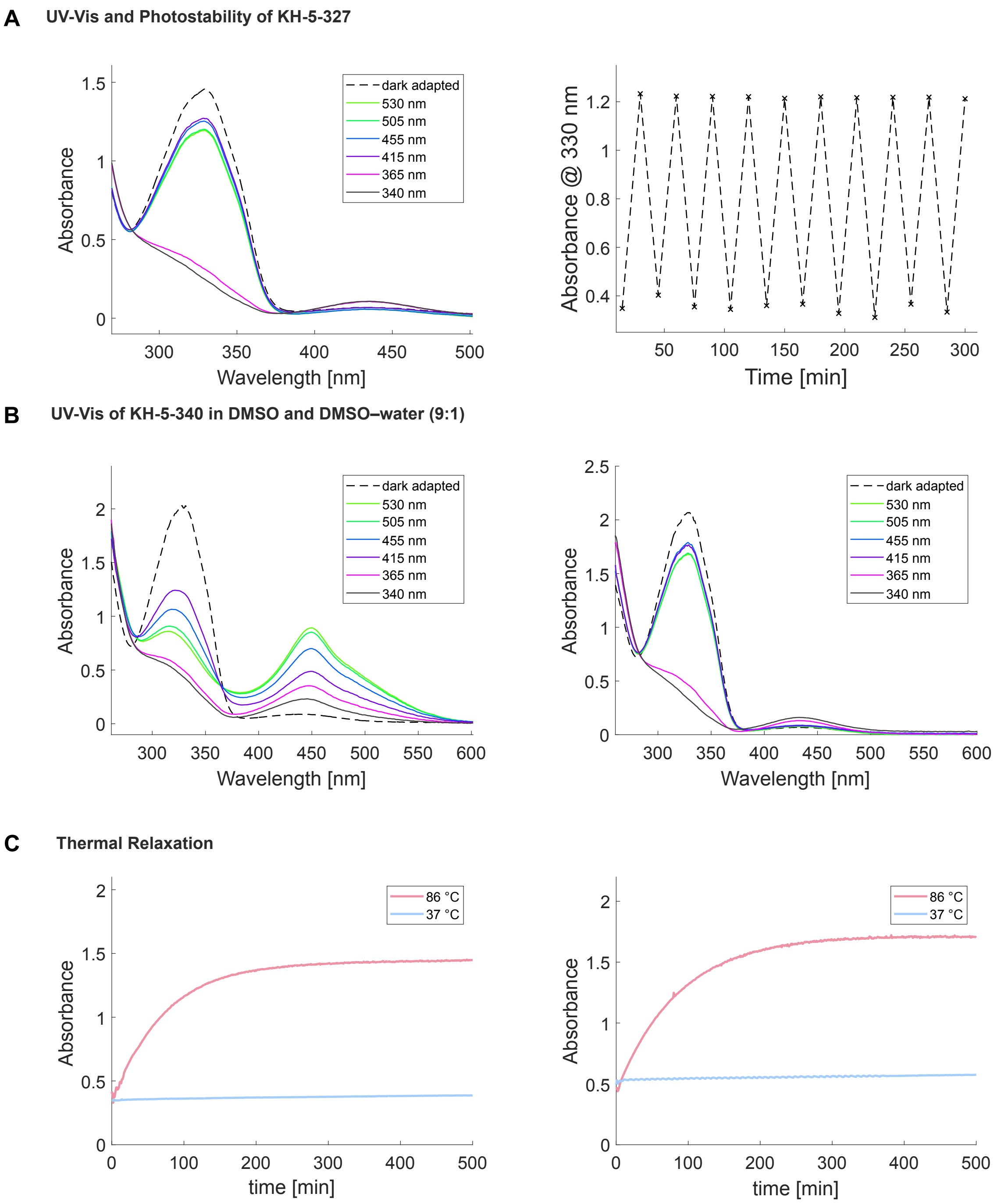

Fig. S56. Photophysical properties of GR $(CF_3)_2$-arylazopyrazole photoPROTACs. A) *Left*: UV-vis spectrum in DMSO (100 µM) after being irradiated for 30 min (340 nm) or 20 min (365–530 nm) with a π–π* λmax of 330 nm. *Right*: Photostability of photoPROTAC **KH-5-327** over a time interval of 300 min. Absorbance at 330 nm of photoPROTAC **KH-5-327** in DMSO (100 µM) was measured following alternating irradiation at 365 and 455 nm for 15 min. B) Comparison of UV-vis spectrum of **KH-5-340** in DMSO (*left*) and a mixture of DMSO–water (9:1, *right*). C) Measurements for the thermal relaxation of **KH-5-327** and **KH-5-340** at 37 *versus* 86 °C after irradiation at 365 nm for 20 min. Measurements were limited by the instability of **KH-5-327** and **KH-5-340** at elevated temperatures, as their decomposition over time prevented reliable thermal half-life determination.

Left: The absorbance of **KH-5-327** (100 μM in DMSO) was measured at 330 nm for 500 min. *Right*: The absorbance of **KH-5-340** (100 μM in DMSO–water (9:1)) was measured at 330 nm for 500 min.

Western Blot Analysis of GR Degradation Induced by arylazotriazole–dexa-linked photoPROTACs

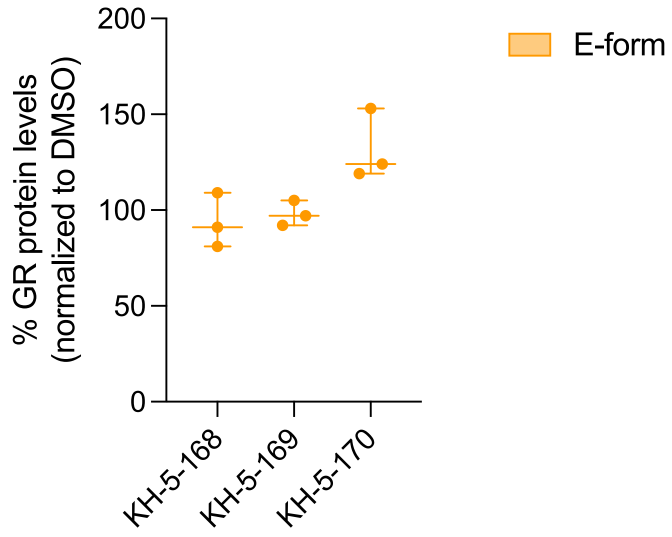

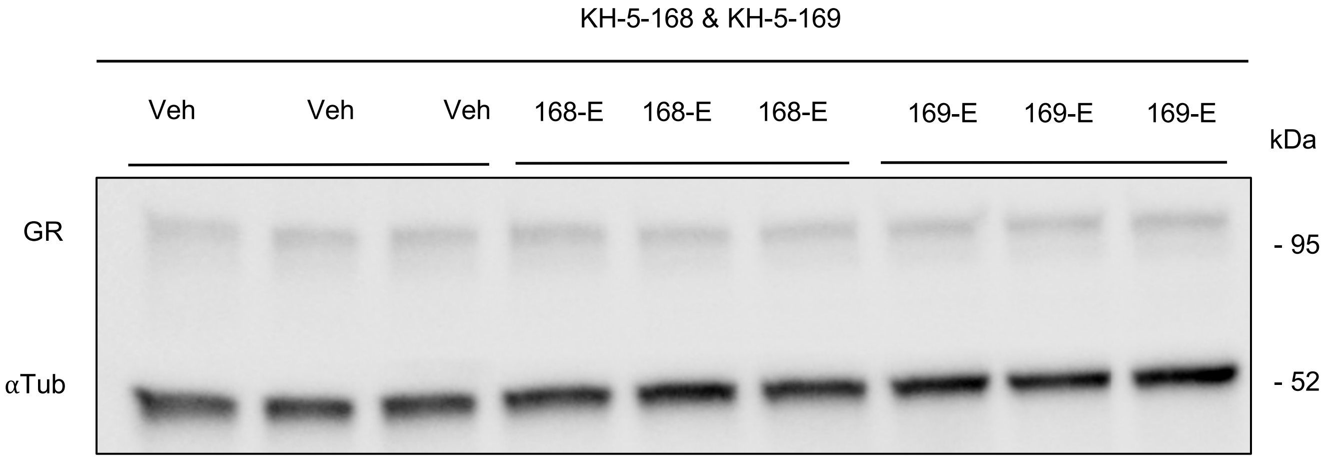

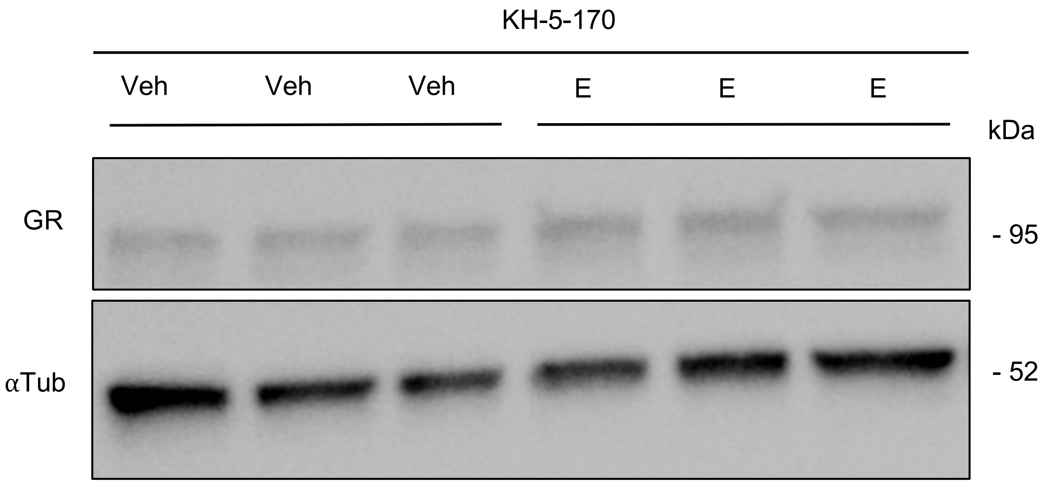

Fig. S57. Arylazotriazole–dexa-linked photoPROTACs. Representative immunoblot and quantification of GR levels by immunoblotting in HEK293T cells treated with 100 nM KH-5-168, KH-5-169, and KH-5-170 in their E-form for 18 h. Veh: vehicle control.

Western Blot Analysis of GR Degradation Induced by arylazotriazole-lenalidomide-linked photoPROTACs

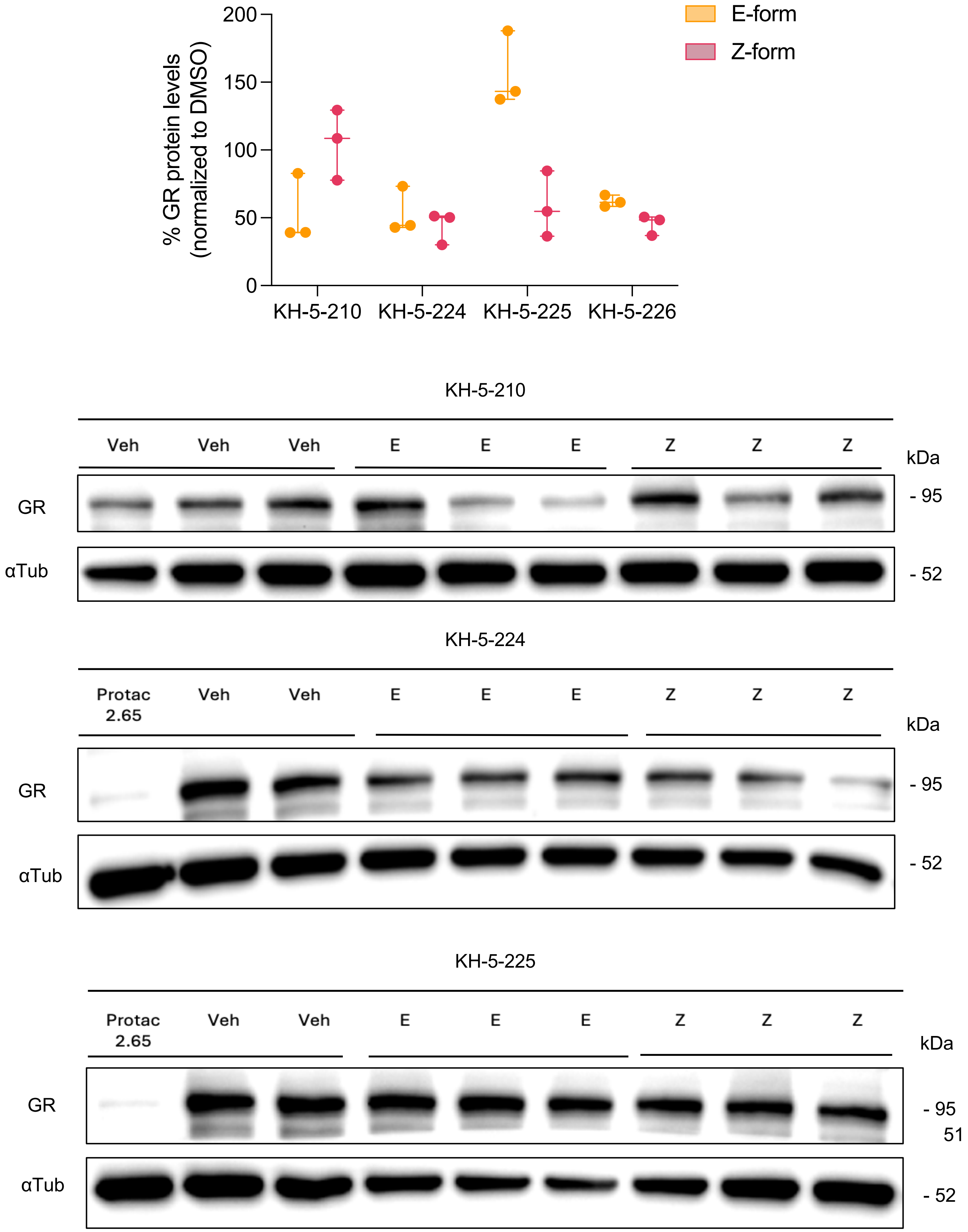

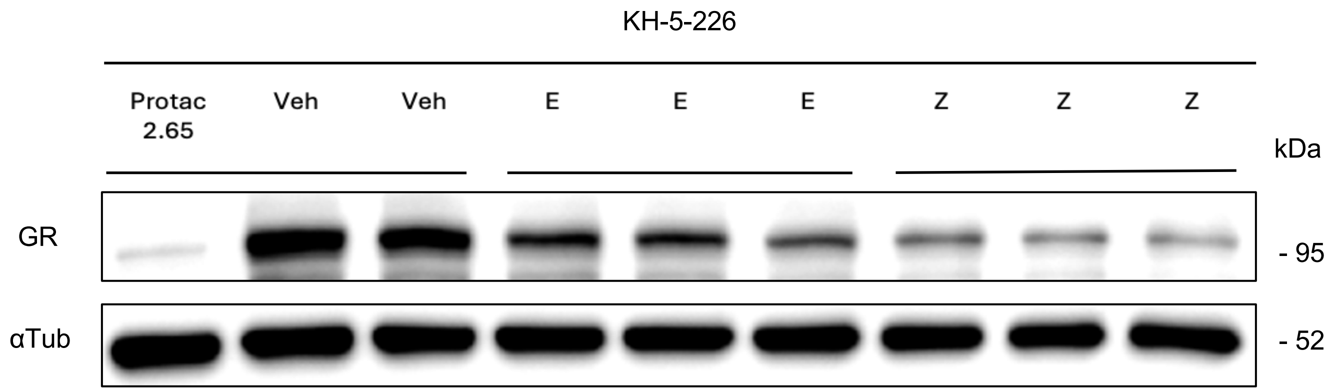

Fig. S58. Arylazotriazole–lenalidomide-linked photoPROTACs. Representative immunoblot and quantification of GR levels by immunoblotting in HEK293T cells treated with 100 nM KH-5-210, KH-5-224, KH-5-225, KH-5-226 in their E- or Z-form for 18 h. Veh: vehicle control.

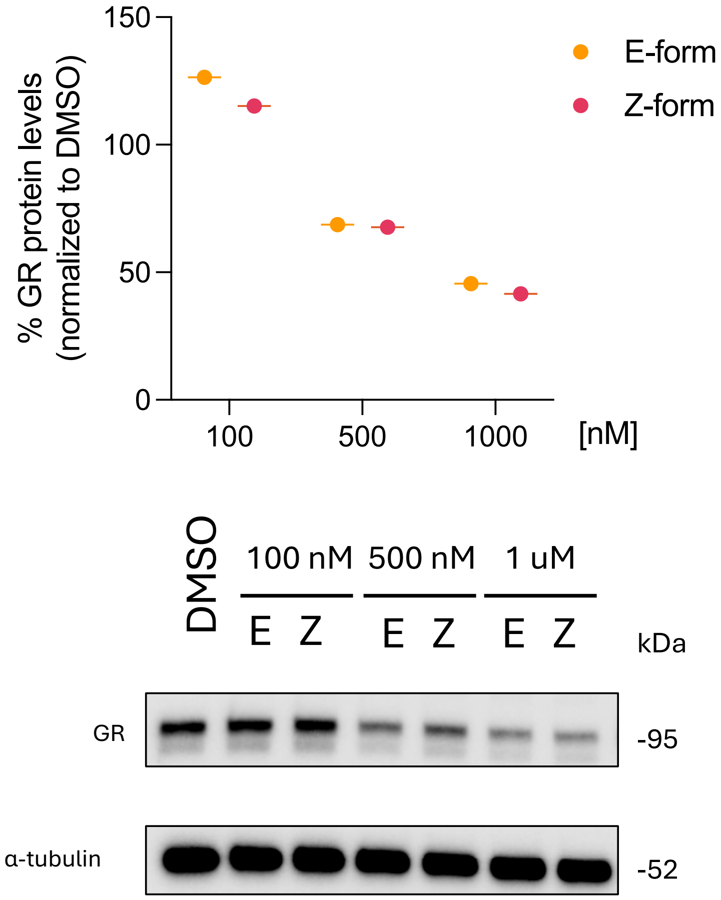

Fig. S59. Arylazotriazole–lenalidomide-linked photoPROTACs. Representative immunoblot and quantification of GR levels by immunoblotting in HEK293T cells treated with 100, 500 or 1000 nM KH-5-210 in their E- or Z-form for 18 h. Veh: vehicle control

Western Blot Analysis of GR Degradation Induced by $(CF_3)_2$-, and $(OEt)_2$- arylazopyrazole photoPROTACs

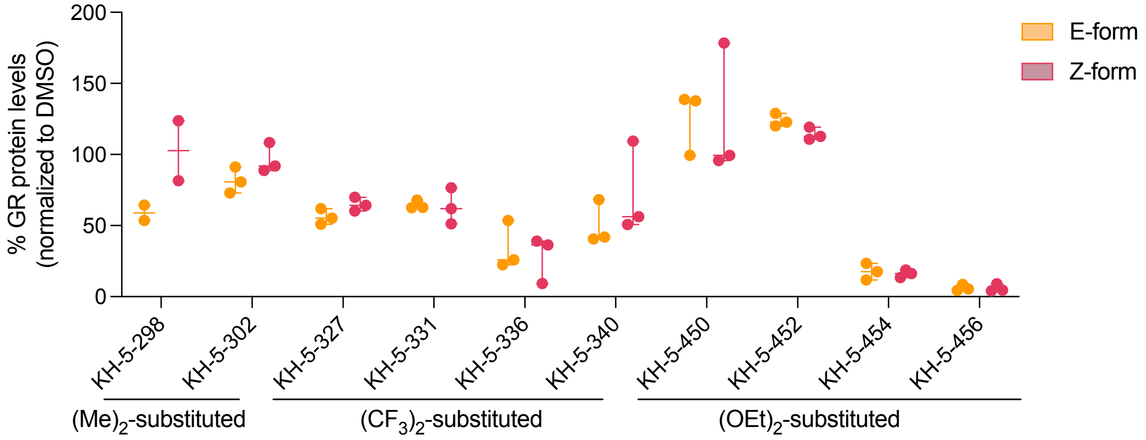

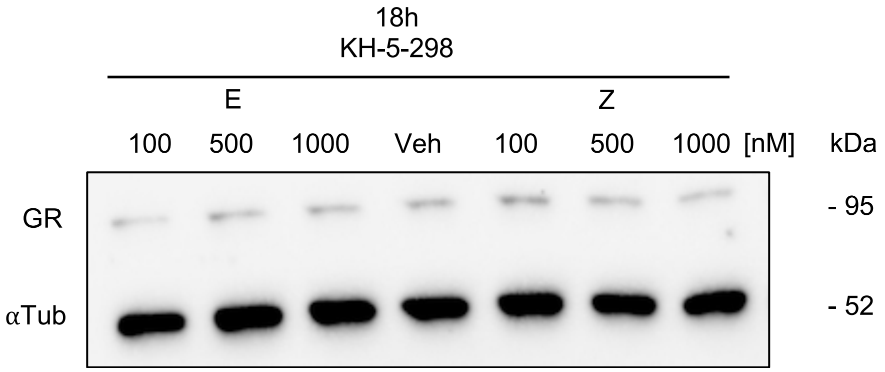

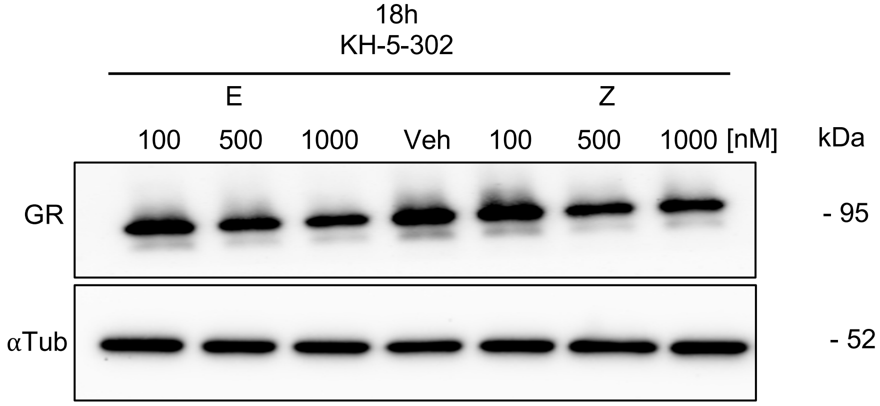

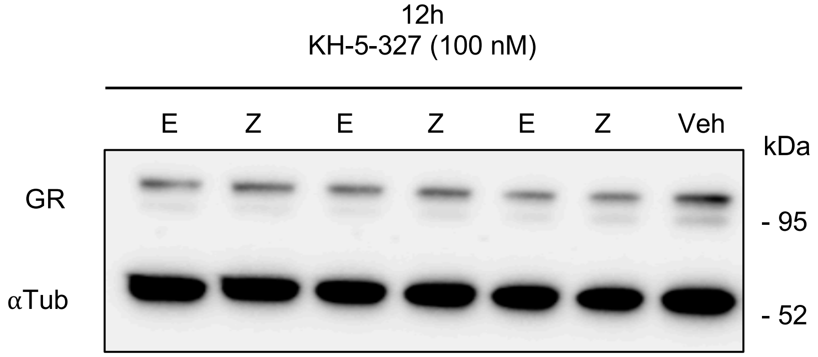

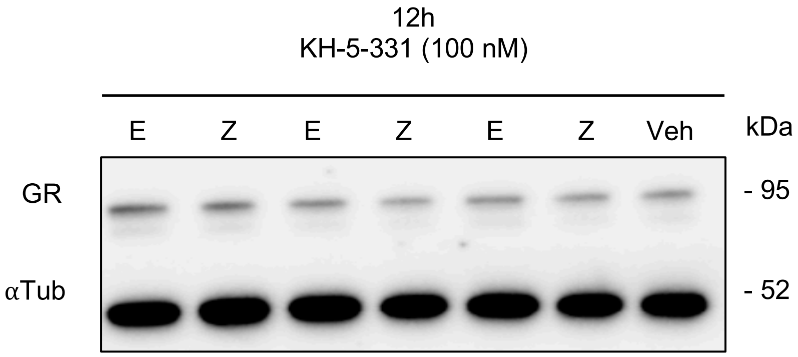
12h
KH-5-331 (100 nM)
E Z E Z E Z Veh
kDa
GR
αTub
- 95
- 52

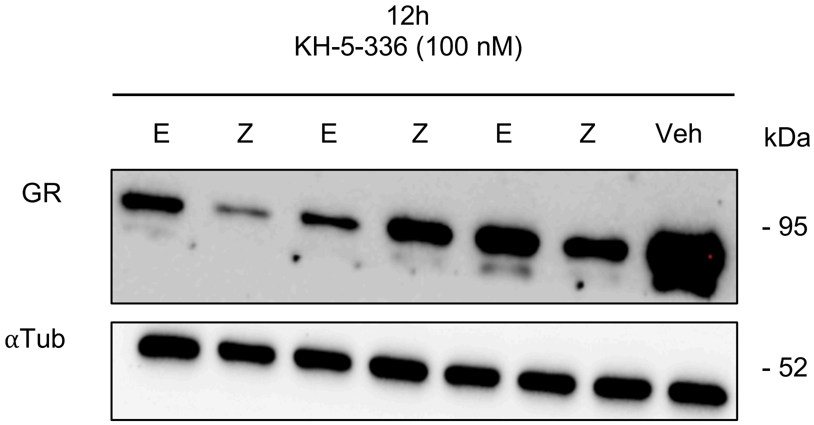
12h
KH-5-336 (100 nM)
E Z E Z E Z Veh
kDa
GR
αTub
- 95
- 52

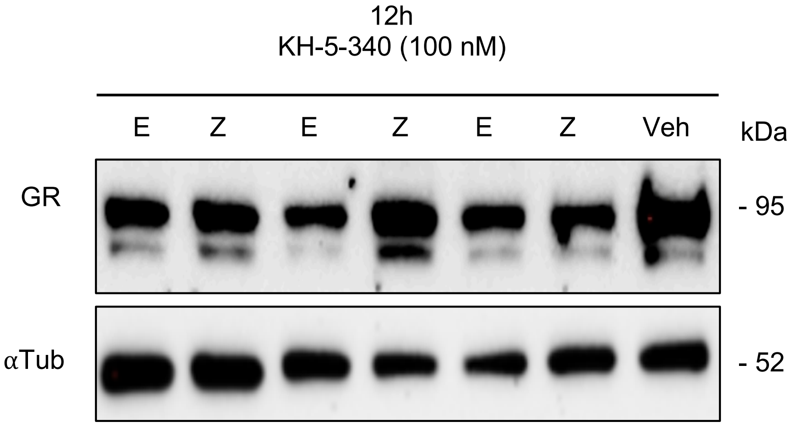
12h
KH-5-340 (100 nM)
E Z E Z E Z Veh
kDa
GR
αTub
- 95
- 52

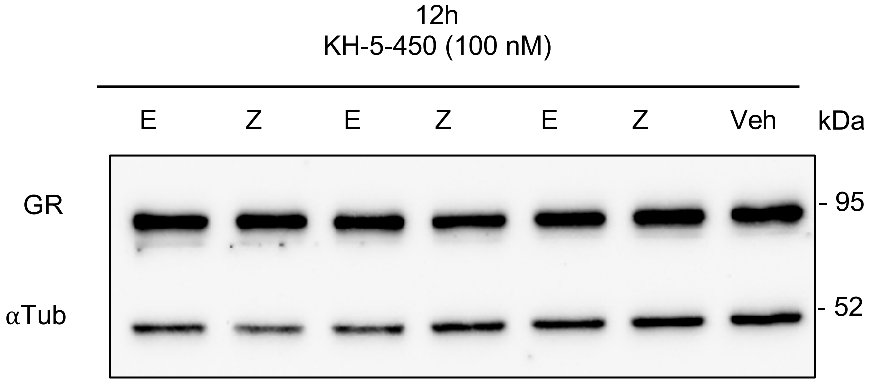
12h
KH-5-450 (100 nM)
E Z E Z E Z Veh
kDa
GR
αTub
- 95
- 52

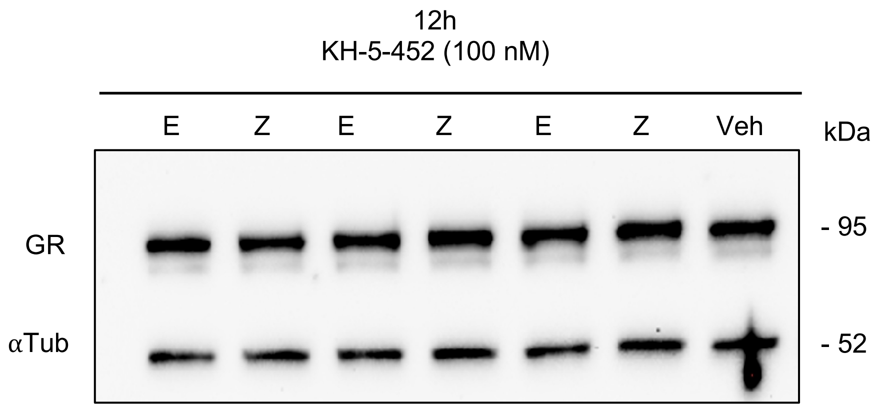

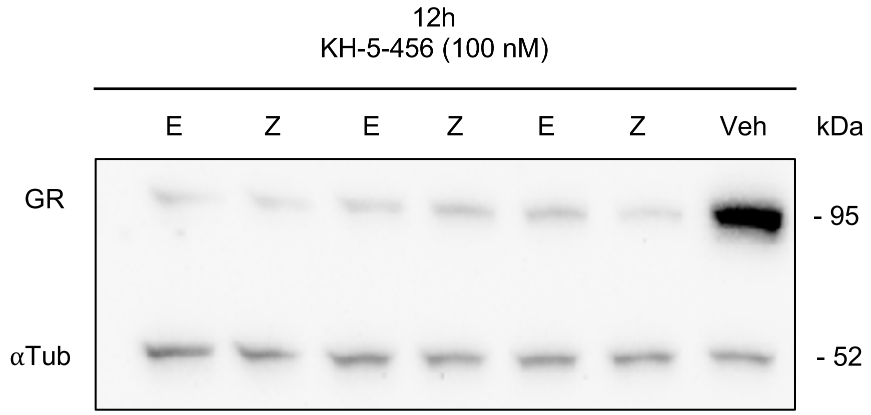

Fig. S60. Overview of GR degradation of $(Me)_2$-, $(CF_3)_2$-, and $(OEt)_2$- arylazopyrazole photoPROTACs. Representative immunoblot and quantification of GR levels by immunoblotting in HEK293T cells treated with 100 nM of KH-5-327 – KH-5-456 in their E- or Z-form for 12 h or 100 nM of KH-5-298 and KH-5-302 for 18 h. Veh: vehicle control

Thermal Relaxation of photoPROTACs

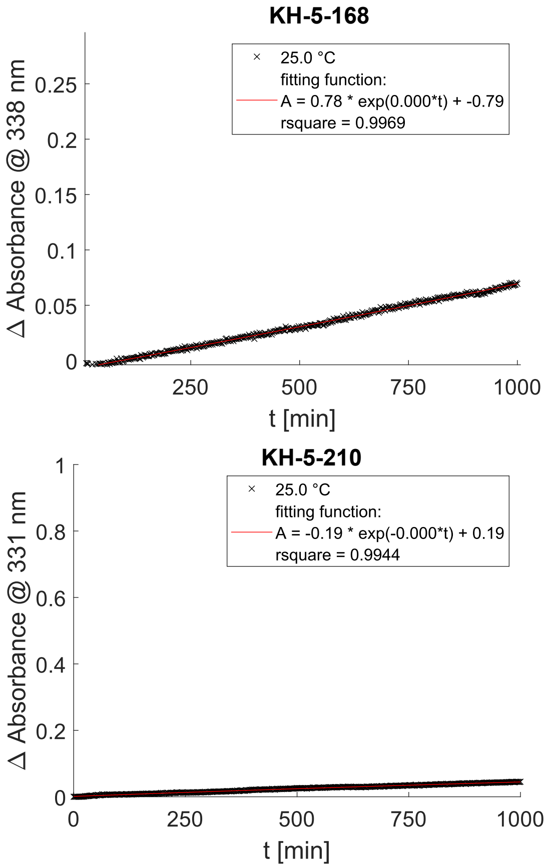

Fig. S61. Thermal relaxations of GR arylazotriazole photoPROTACs **KH-5-168**, **KH-5-226** and **KH-5-210** in DMSO–water (100 μM, 2:1). After irradiation at 365 nm for 20 min, the change in absorbance was measured at 338 nm for **KH-5-168** and 331 nm for **KH-5-226** and **KH-5-210** every min over the period of 1000 min at 37 °C. For the fitting functions, a first-order rate law is assumed.

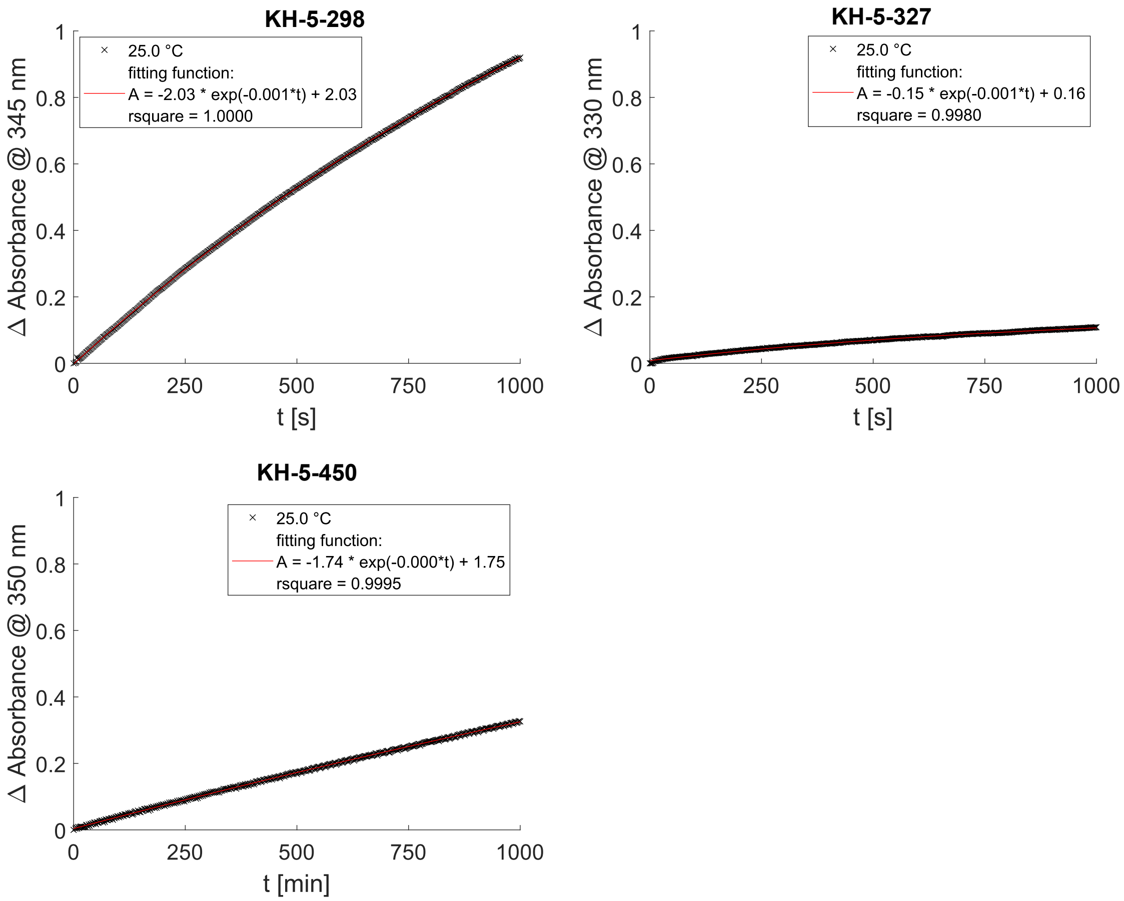

Fig. S62. Thermal relaxations of GR arylazopyrazole photoPROTACs **KH-5-298**, **KH-5-327** and **KH-5-450** in DMSO (100 µM). After irradiation at 365 nm for 20 min, the change in absorbance was measured at 345 nm for **KH-5-298**, 330 nm for **KH-5-327** and 350 nm for **KH-5-450** every min over the period of 1000 min at 37 °C. For fitting functions, a first-order rate law is assumed.

UV-Vis Spectra of photoPROTACs in Buffer-DMSO (3:1)

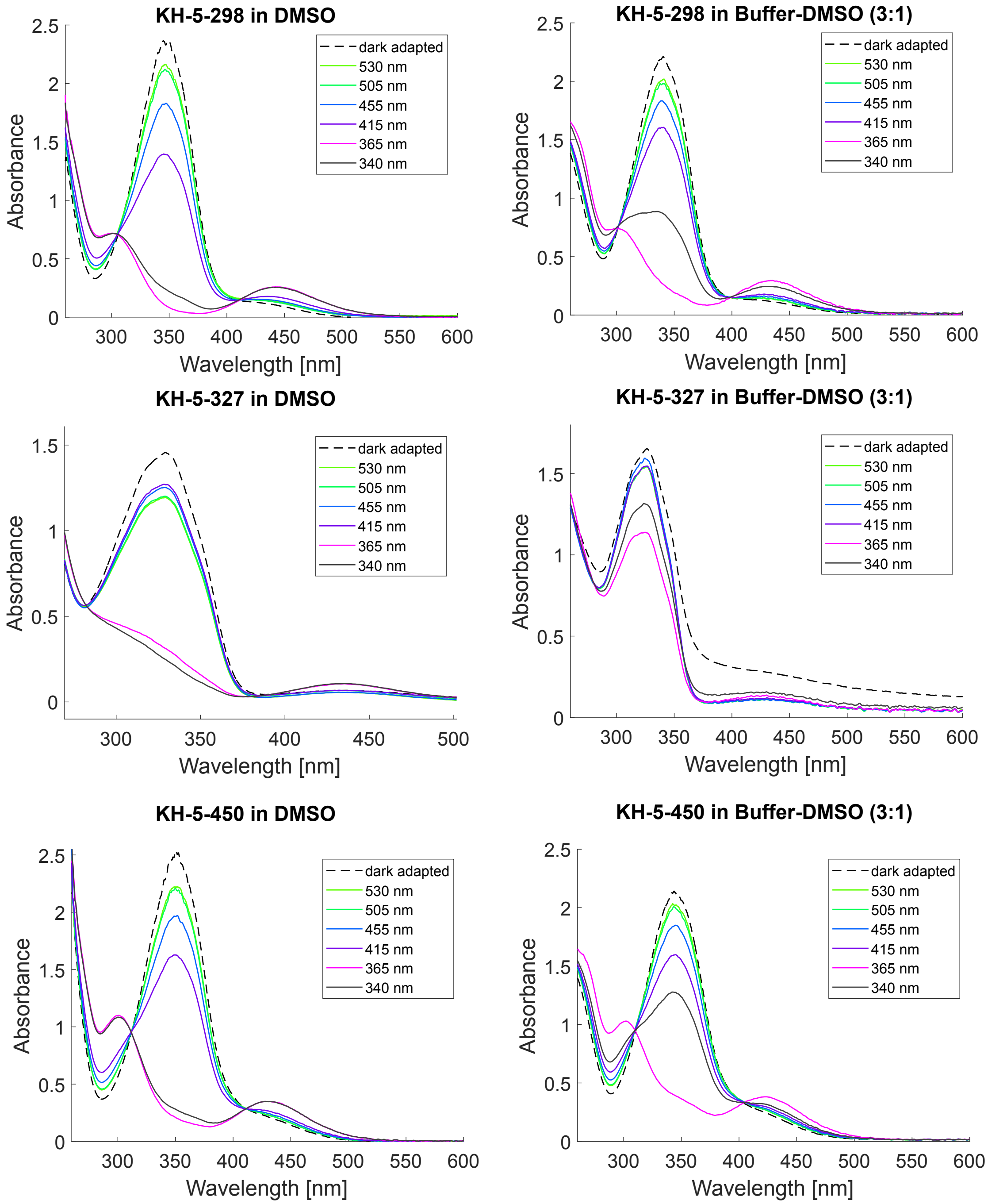

Fig. S63. UV-vis spectra of GR arylazopyrazole photoPROTACs **KH-5-298**, **KH-5-327** and **KH-5-450** (100 µM) in DMSO and buffer–DMSO (3:1) after irradiation for 30 min (340 nm) or 20 min (365–530 nm).

Western Blot Analysis of GR Degradation Induced by arylazopyrazole photoPROTACs with intermittent irradiation

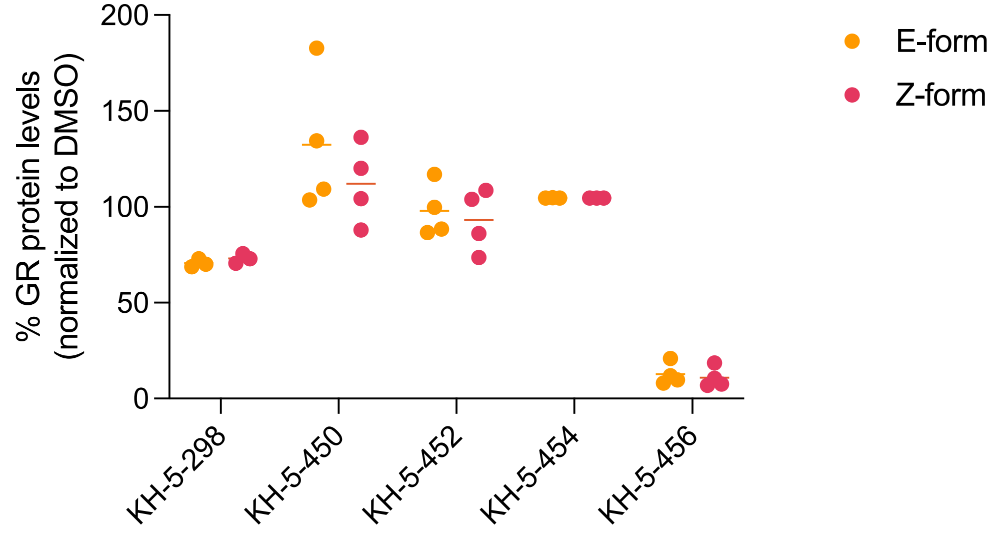

12h, irradiated every hour for 30s
KH-5-298 (nM)

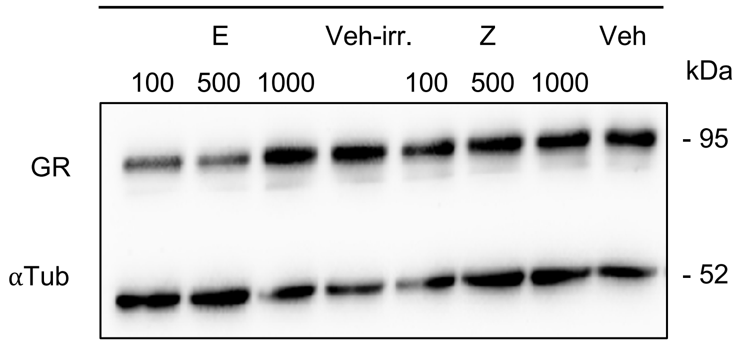

KH-5-452 (1)
(100 nM)

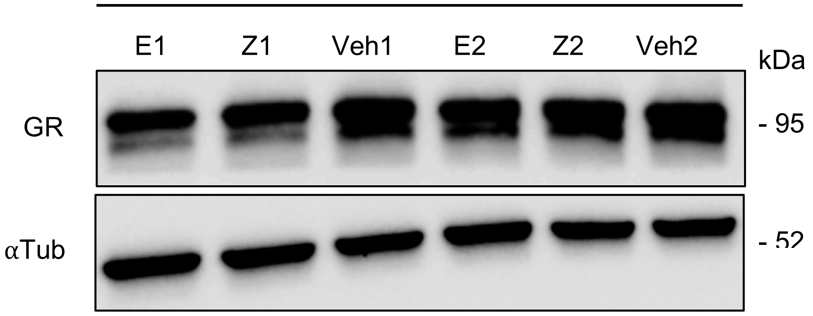

KH-5-452 (2)
(100 nM)

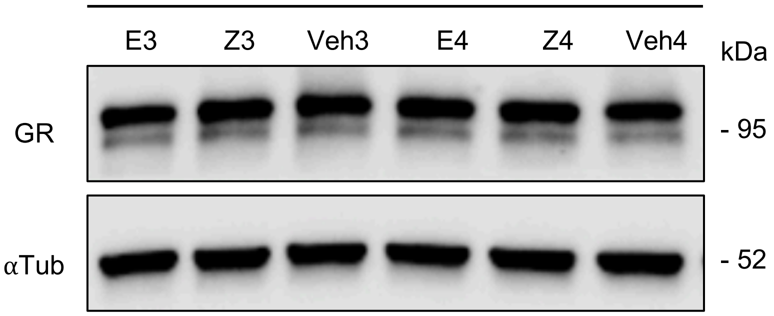

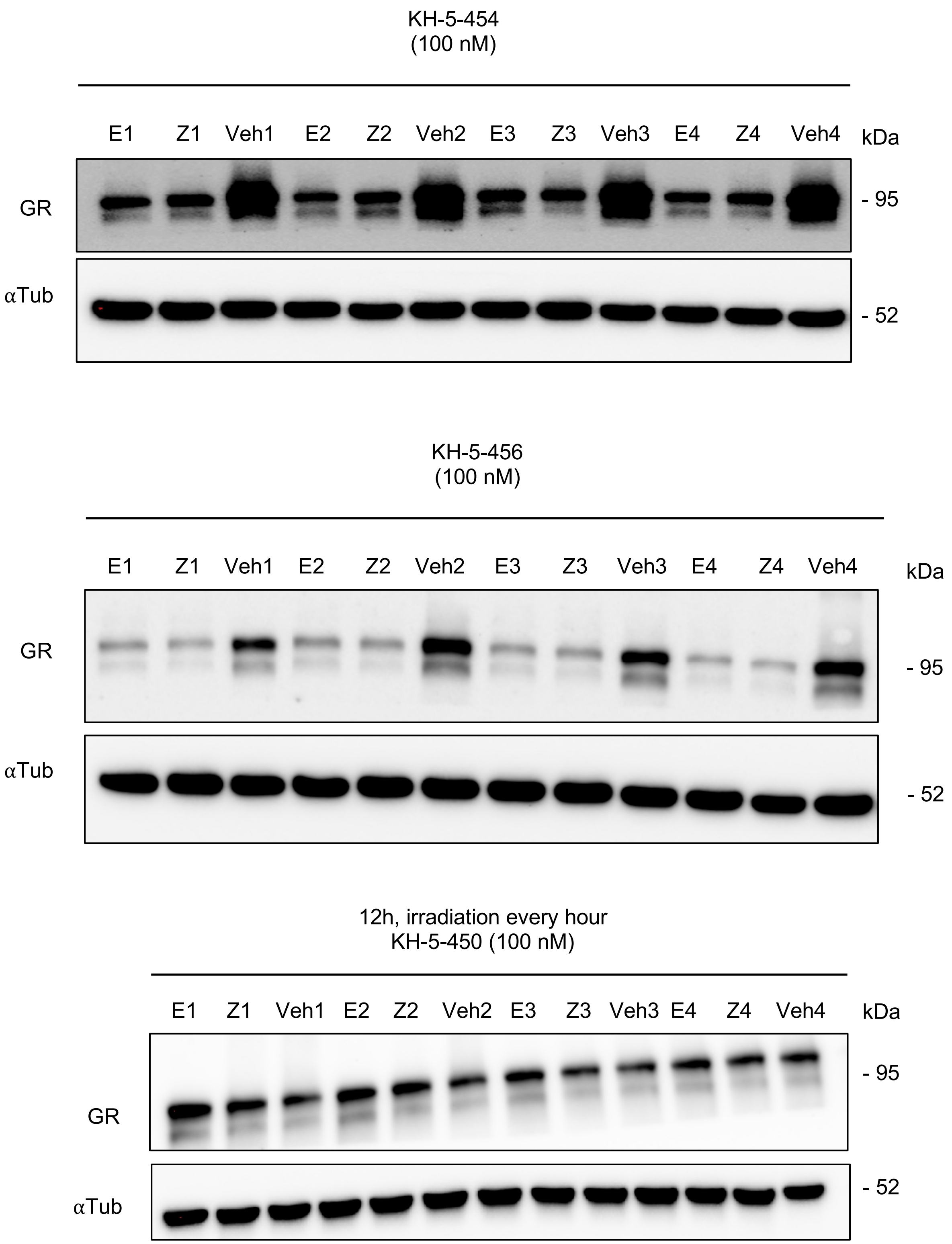

Fig. S64. **(**Me)$_2$- **,** and **(**OEt)$_2$-arylazopyrazole photoPROTACs. Representative immunoblots and quantification of GR levels by immunoblotting in HEK293T cells treated with 100 nM of KH-5-298, KH-5-450, KH-5-452, KH-5-454, or KH-5-456 in their *E*- or *Z*-form for 12 h, with 365 nm irradiation every 1 h for 30 s. Veh: vehicle control.

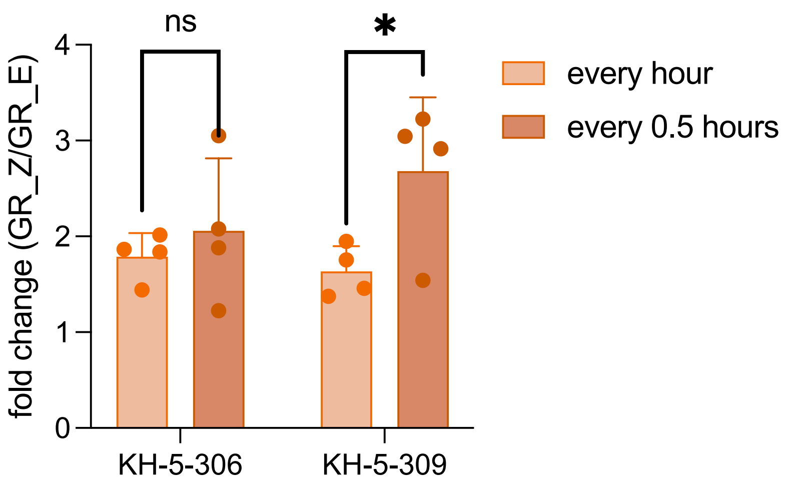

Fig. S65. Fold change in GR levels comparing irradiation every hour versus every 30 min for KH-5-306 and KH-5-309 treatments. Two-way ANOVA: significant main effect for irradiation period ($F(1, 12) = 5.355$, $p = 0.0392$), but no significant interaction between PhotoPROTAC and irradiation period ($F(1, 12) = 1.877$, $p = 0.1958$). Follow-up Šídák's multiple comparisons test: significant difference between every hour and every 0.5 hours for KH-5-309 ($p < 0.05$).

Western Blot Analysis of GR Degradation in A549 cells Induced by GR PROTAC

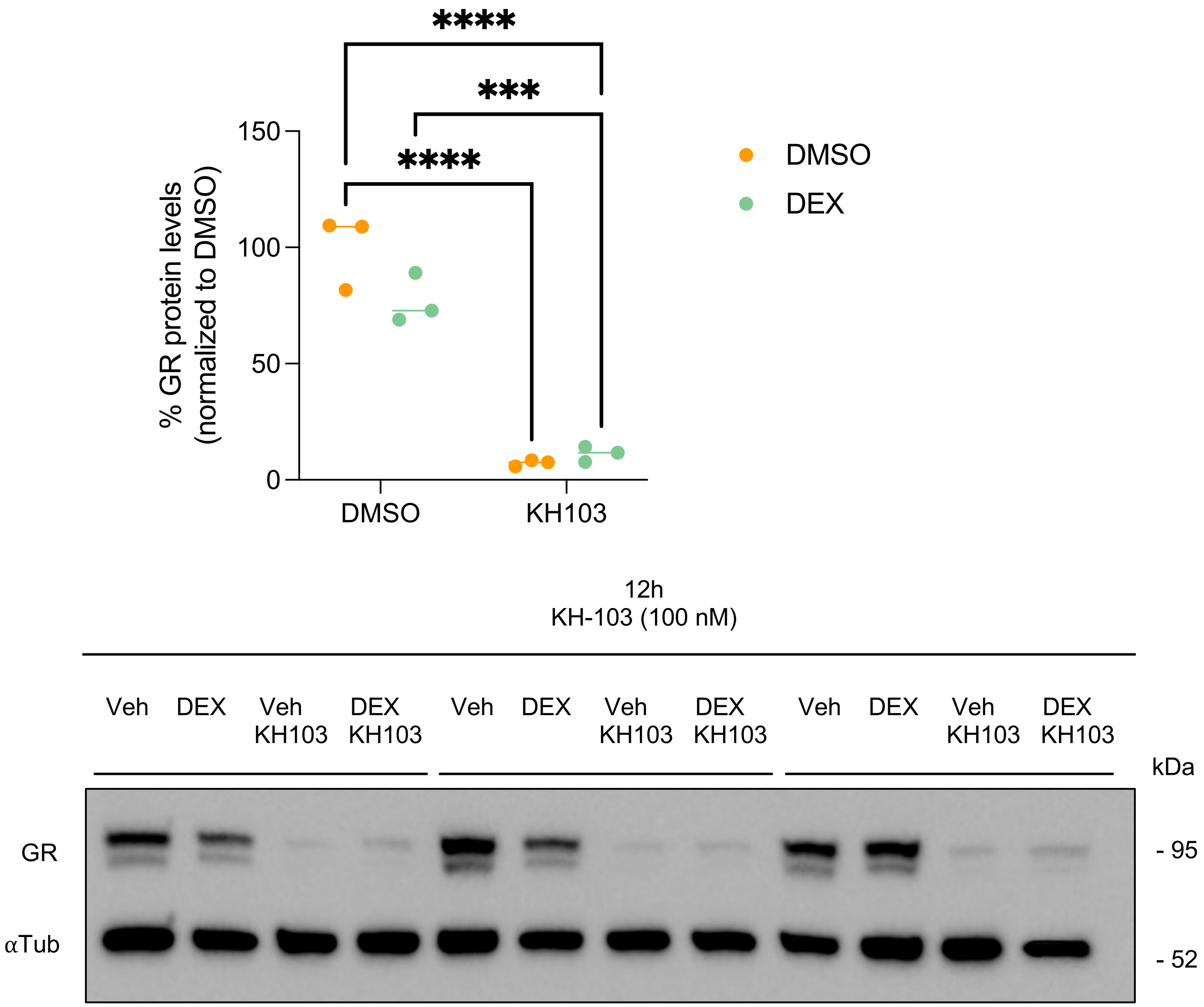

Fig. S66. Representative immunoblot and quantification of GR levels by immunoblotting in A549 cells treated with 100 nM of KH-103 for 12 h. Two-way ANOVA revealed a significant main effect of KH-103 ($F(1,8) = 199.1$, $p < 0.0001$) and a significant interaction between DEX and KH-103 ($F(1,8) = 5.806$, $p = 0.0425$). Tukey's post hoc test showed significant differences between DMSO and KH-103 ($p < 0.0001$), DMSO and KH-103 + DEX ($p < 0.0001$), and between DEX and KH-103 + DEX ($p = 0.0002$). Veh: vehicle control.

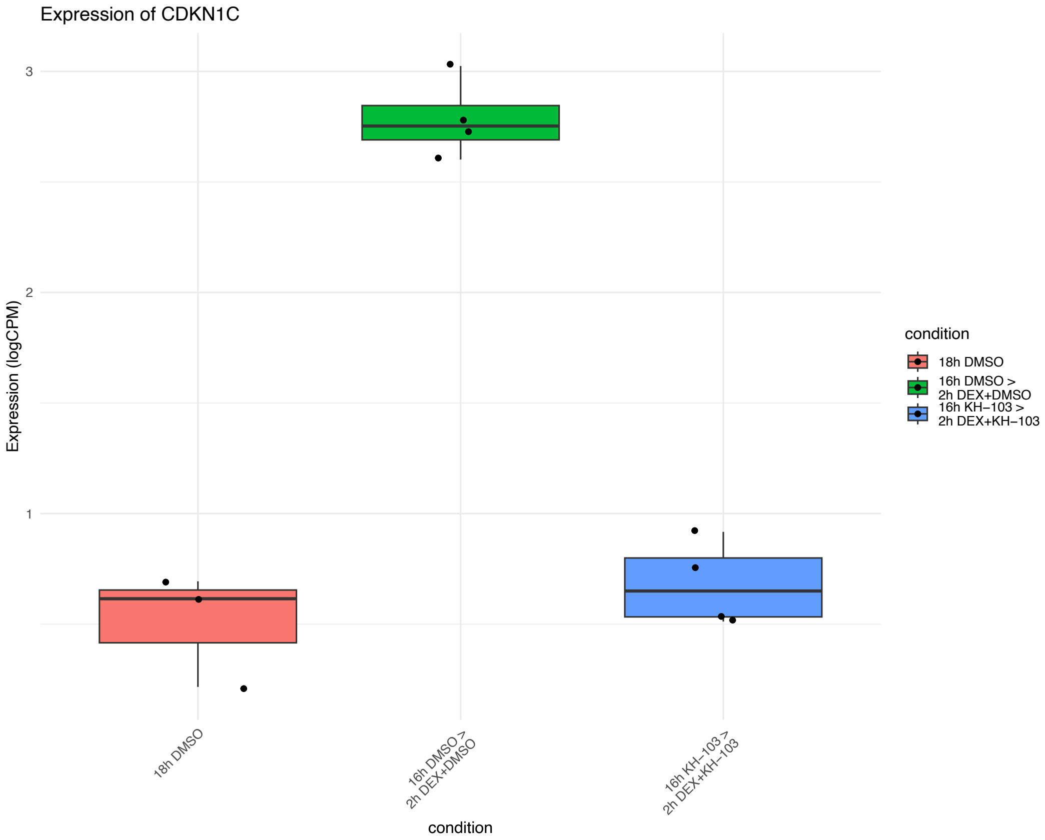

Fig. S67. CDKN1C expression levels (logCPM) in A549 cells measured by RNA-sequencing following three treatment conditions: 18 h DMSO (red), 18 h DMSO including a final 2 h exposure to DEX (green), and 18 h KH-103 including a final 2 h exposure to DEX (blue).

Application of KH-5-309 in A549 Cells

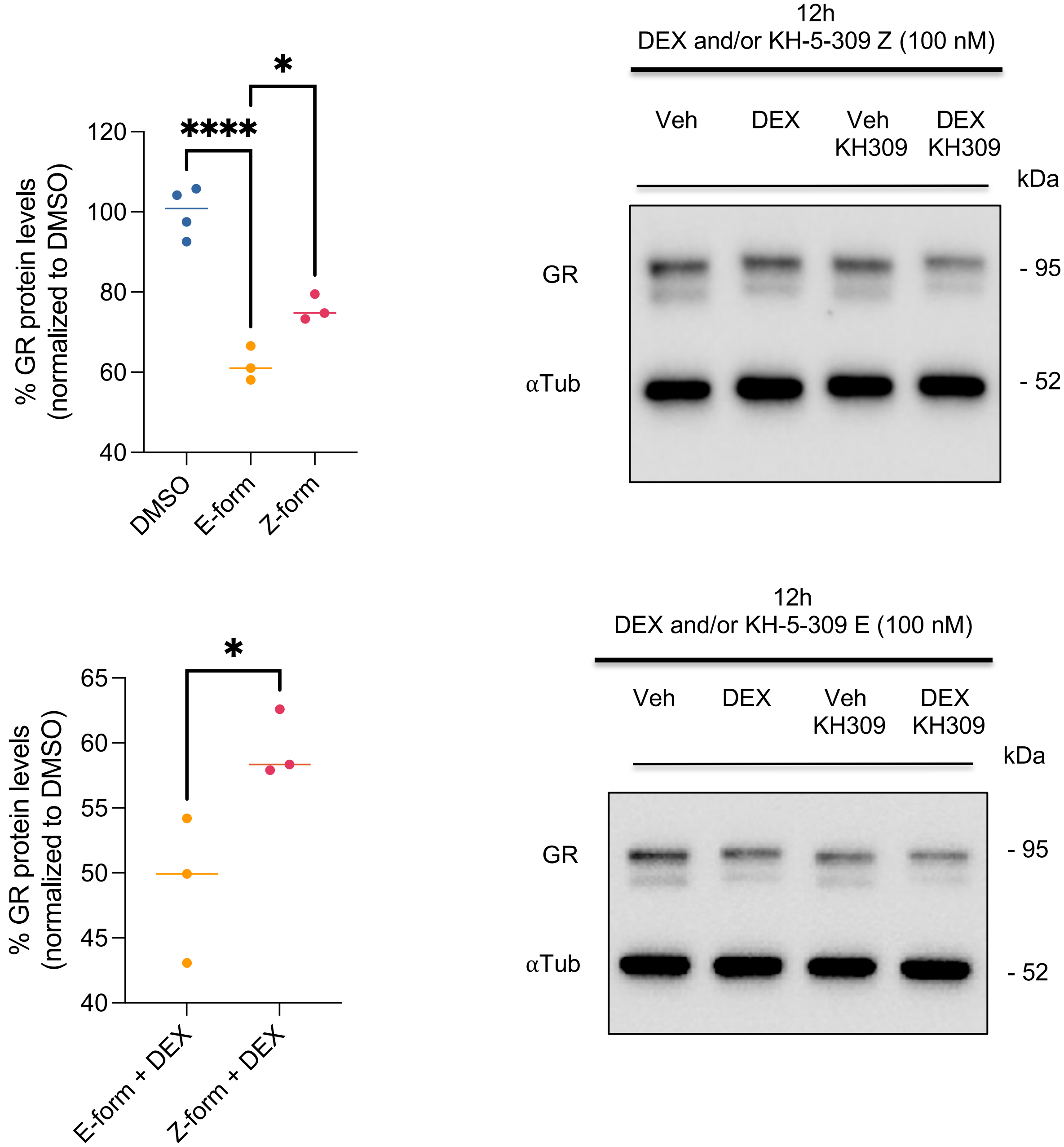

Fig. S68. Representative immunoblot and quantification of GR levels in A549 cells treated for 12 h with 100 nM KH-5-309 in either the *E*- or *Z*-isomeric form. The upper plot shows GR levels following treatment alone (ordinary one-way ANOVA: $F(2,7) = 53.56$, $p < 0.0001$; Tukey's multiple comparisons revealed significant differences between DMSO and E-form KH-5-309 ($p < 0.0001$), and between E-form and Z-form KH-5-309 ($p = 0.0251$)). The lower plot shows GR levels after a final 2 h co-treatment with DEX within the 12 h treatment period (unpaired two-tailed t-test: $t(4) = 2.956$, $p < 0.05$, comparing *E*-form + DEX vs. *Z*-form + DEX). During the 12 h treatment, cells were irradiated with 365 nm light every 30 min for 30 s. Veh: vehicle control.

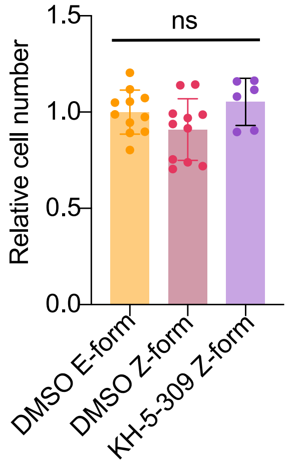

Fig. S69. Flow-cytometry quantification of live DAPI-negative cells comparing *E*-DMSO (non-irradiated), *Z*-DMSO (irradiated), and *Z*-KH-5-309 (irradiated). Ordinary one-way ANOVA revealed no significant differences in the fraction of DAPI-negative live cells across the three conditions ($F(2, 25) = 2.480$, $p = 0.1041$).

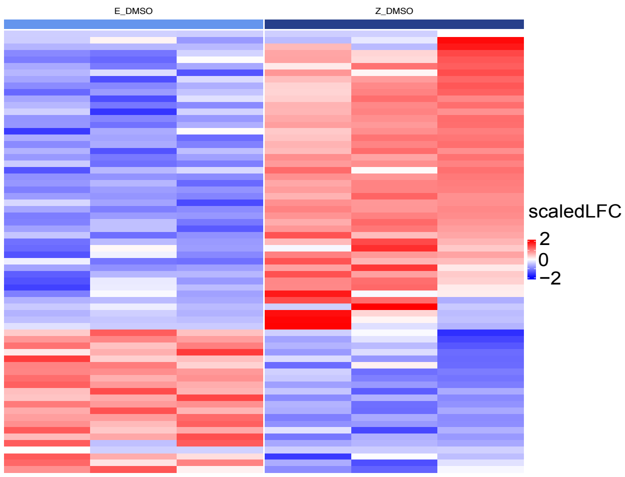

Fig. S70. Heatmap showing scaled log fold change (LFC) of differentially expressed genes (DEGs) in A549 cells treated with DMSO for 12 hours. Expression profiles are compared between non-irradiated (*E*-DMSO) and irradiated (*Z*-DMSO) conditions. Irradiation was performed with 365 nm light for 30 seconds every 30 minutes. Red indicates upregulation; blue indicates downregulation.

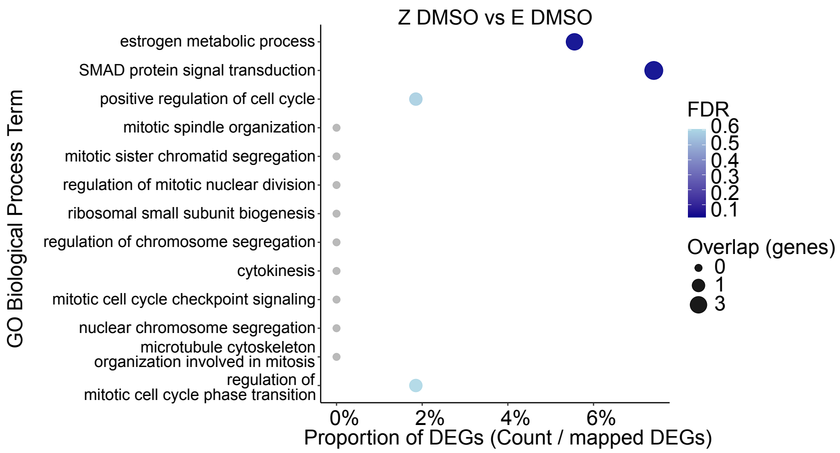

Fig. S71. Bubble plot showing Gene Ontology (GO) Biological Process terms enriched among the 68 differentially expressed genes (DEGs) identified between irradiated (*Z*-DMSO) and non-irradiated (*E*-DMSO) A549 cells treated with DMSO for 12 h. Irradiation was performed with 365 nm light for 30 s every 30 min. The x-axis depicts the proportion of DEGs mapped to each GO term, bubble size reflects the number of overlapping genes, and bubble color indicates the FDR-corrected significance level.

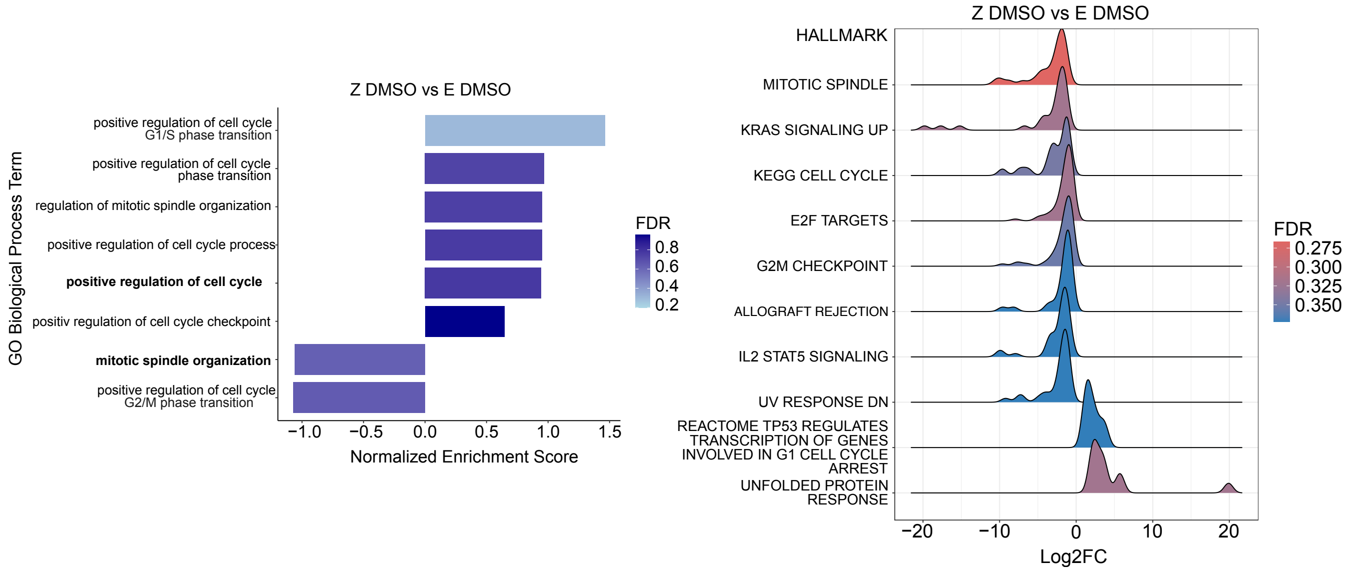

Fig. S72. GSEA results comparing transcriptional profiles of irradiated (*Z*-DMSO) versus non-irradiated (*E*-DMSO) A549 cells treated with DMSO for 12 h. Left: Normalized enrichment scores for GO Biological Process terms, with bar color indicating FDR-corrected significance. Right: Ridge plots showing log2FC distributions of Hallmark gene sets from MSigDB. Color indicates FDR-corrected significance.

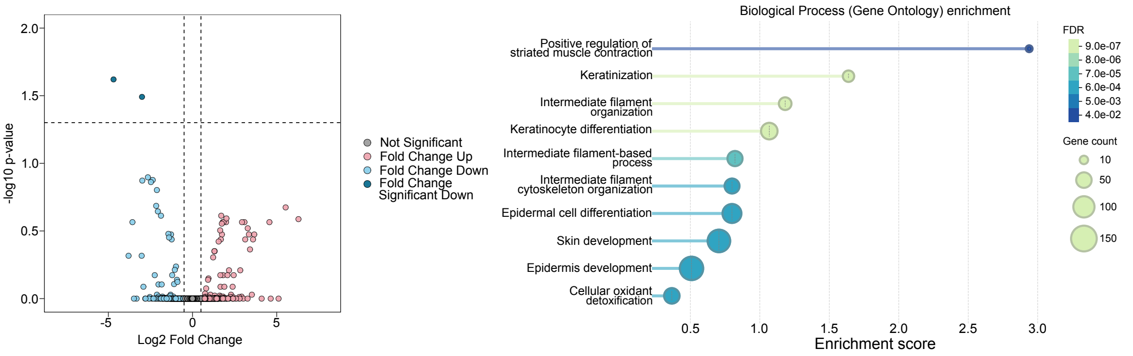

Fig. S73. Label-free quantitative proteomics of irradiated and non-irradiated A549 cells. (Left) Volcano plot showing protein abundance changes between irradiated (*Z*-DMSO) and non-irradiated (*E*-DMSO) A549 cells following 12 h DMSO treatment. Irradiation was performed using 365 nm light for 30 s every 30 min. The x-axis shows the log2FC, and the y-axis shows the –log10 p-value. Proteins are color-coded according to significance and direction of change. (Right) Gene Ontology Biological Process enrichment analysis based on the quantified proteome, with enrichment scores on the x-axis and false discovery rates (FDR) indicated by color. Circle sizes correspond to gene counts within each enriched term.

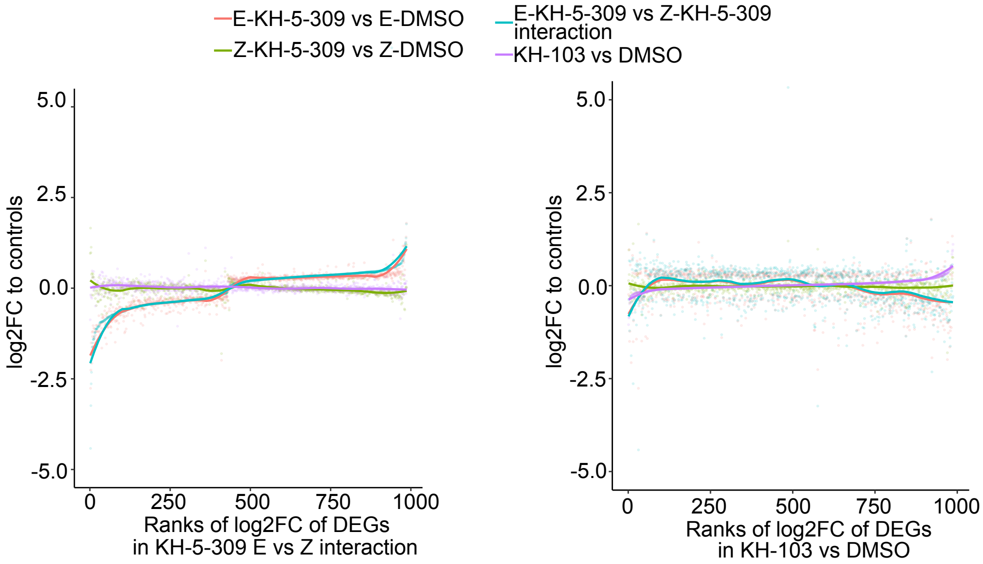

Fig. S74. Rank-ordered log2FC profiles illustrating gene-expression differences across four contrasts: *E*-KH-5-309 vs *E*-DMSO, *Z*-KH-5-309 vs *Z*-DMSO, the *E*- vs *Z*-KH-5-309 interaction term, and KH-103 vs DMSO. Curves are shown using two alternative ranking inputs: (left) DEGs from the *E*- vs *Z*-KH-5-309 interaction term comparison, and (right) DEGs from the KH-103 vs DMSO comparison.

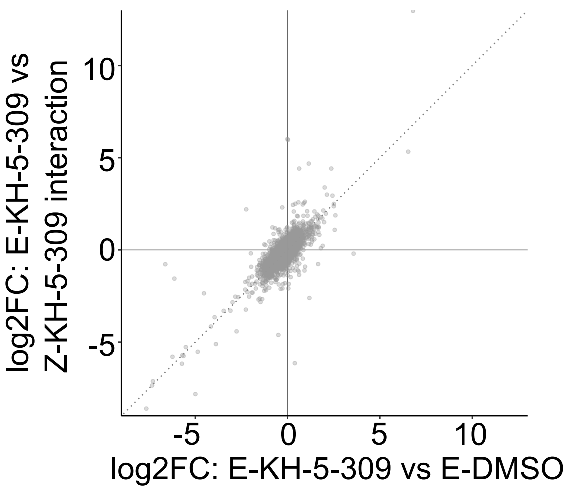

Fig. S75. Scatter plot comparing log2FCs for *E*-KH-5-309 vs. *E*-DMSO (x-axis) and the *E*-KH-5-309 vs. *Z*-KH-5-309 interaction term (y-axis). Each point represents a gene.

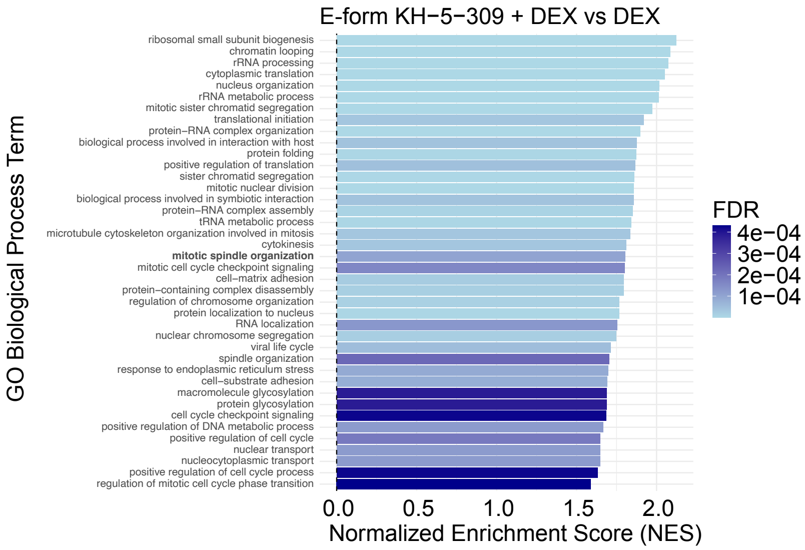

Fig. S76. Gene Ontology (GO) Biological Process enrichment analysis comparing A549 cells treated with E-form KH-5-309 and DEX versus DEX alone. The bar graph displays normalized enrichment scores (NES) for significantly enriched GO terms, with color indicating the false discovery rate (FDR).

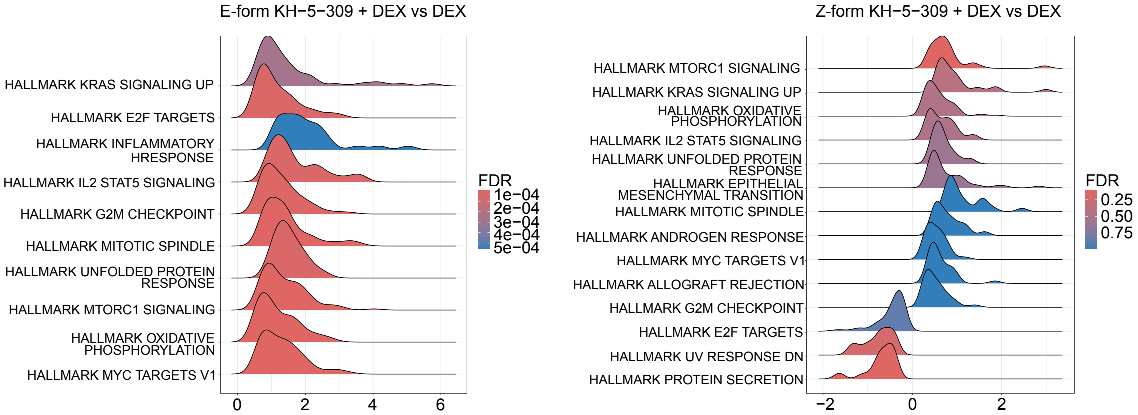

Fig. S77. Gene Set Enrichment Analysis (GSEA) using Hallmark gene sets from MSigDB, comparing transcriptional responses in A549 cells treated with *E*-form KH-5-309 + DEX and *Z*-form KH-5-309 + DEX versus DEX alone. Log2 fold-change (log_2FC) distributions are shown for Hallmark gene sets, with color indicating false discovery rate (FDR).

2. Supplementary Table

Table S1. Primer Sequences for Target Gene Quantification by RT-qPCR

Primer	Distributor	Sequence Forward	Sequence Reverse
HPRT	Micro-Synth	5’-TCAGGCGAACCTCTCGGCTT-3’	5’-TCATCACTAATCACGACGCCAG-3’
PPIA	Micro-Synth	5’-TGGTCAACCCCACCGTGTTC-3’	5’-GTCTGCAAACAGCTCAAAGGAG-3'
CDKN1C	Micro-Synth	5’-GAAACCAGGAAGCCTAGCATC-3’	5’-GAAACCAGGAAGCCTAGCATC-3’

Table S2. Comparison of photophysical and functional properties of published photoPROTACs/ PHOTACs and the GR-targeting photoPROTACs developed in this work. Thermal half-lives reported for photoPROTACs developed in this work were determined by Eyring plot and extrapolated to 25 °C.

**Compound**	**Target**	**PSS (*Z*-isomer)**	**PSS (*E*-isomer)**	**Thermal half-life**	**Potency**	***E/Z* functional difference**	**Reference**
**KH-5-306/309**	**GR**	**95%**	**89% – 92%**	**3 – 4.2 days**	**100 nM**	**40% – 60%**	**This work**
PHOTAC-I-3; PHOTAC-II-5/6	BRD2-4 or FKBP12	>90%	>70%	1.7 – 8.8 h	100 nM – 3 uM	qualitatively reported	Reynders et al. (2020)
photoPROTAC-1	BRD2	68%	95%	several days	50 nM – 1 uM	qualitatively reported	Pfaff et al. (2019)
AP-PROTAC 1/2	BRD2-4, Multi-kinase	85% – 90%	78% – 80%	31.7 h	100 nM – 300 nM	22% – 75%	Zhang et al. (2022)
PHOTAC 12	HDAC6	63% – 80%	>94%	10.3 h	1 uM	40%	Wurnig et al. (2025)

3. Detailed Chemical Methods

3.1 General Methods

All non-aqueous reactions were performed in flame-dried glassware sealed with rubber septa under an atmosphere of dry nitrogen. Anhydrous solvents and reagents were transferred using nitrogen-purged syringes. Reactions were stirred magnetically and monitored by thin-layer chromatography (TLC) on Merck silica gel F254 plates, visualized under UV light or by aqueous potassium permanganate ($KMnO_4$) staining followed by heating. Organic solvents were removed by rotary evaporation at 40 °C under reduced pressure.
Crude products were absorbed onto SiliaFlash® Silica Gel F60 and purified by flash column chromatography using SiliaFlash® Silica Gel F60 (40–63 µm) under 0.3–0.5 bar overpressure. For the purification of compound 3.121, Nacalai Tesque Silica Gel 60 (spherical, neutral, 150–325 mesh, 42–105 µm) was used. Reported yields refer to purified and spectroscopically pure compounds unless otherwise stated.

3.2 Solvents and Reagents

All reagents were purchased from commercial suppliers (ABCR, ACROS, Sigma Aldrich, Fluka, TCI, Strem, Alfa, Combi-Blocks, or Fluorochem) and used without further purification. Anhydrous solvents over molecular sieves were obtained from Acros and used as received. Triethylamine (Et_3N) and DIPEA were freshly distilled from CaH_2 under nitrogen. Deuterated solvents were sourced from Cambridge Isotope Laboratories, Apollo Scientific, or Sigma-Aldrich.

3.3 Analytical Techniques

3.3.1 Nuclear Magnetic Resonance (NMR) Spectroscopy

Proton (^1H) and carbon (^{13}C) NMR spectra were recorded on Bruker AV400, DRX400, AV500, or AVIII 600 MHz spectrometers at 298 K. Chemical shifts (δ) are reported in ppm relative to residual solvent peaks: $CHCl_3$ (7.26 ppm for ^1H, 77.16 ppm for ^{13}C), CH_3OH (3.31 ppm for ^1H, 49.0 ppm for ^{13}C), and $(CH_3)_2CO$ (2.05 ppm for ^1H, 29.84 and 206.26 ppm for ^{13}C). Signal multiplicities are designated as s (singlet), d (doublet), t (triplet), q (quartet), m (multiplet), or br (broad). Coupling constants (J) are given in Hz. All ^{13}C spectra were recorded with broadband ^1H decoupling.

3.3.2 Infrared Spectroscopy

IR spectra were recorded as thin films of neat compounds on a PerkinElmer Two-FT-IR spectrometer. Only characteristic absorption bands are reported in wavenumbers (cm^{-1}).

3.3.3 Mass Spectrometry

High-resolution electrospray ionization mass spectrometry (HRMS-ESI) was performed by the Mass Spectrometry Service of the Laboratorium für Organische Chemie at ETH Zürich (Mr. Louis Bertschi, Mr. Daniel Wirz, and Mr. Michael Meier).

3.4 Photophysical Characterization

3.4.1 General Procedures

All spectra were recorded on a Mettler Toledo UV5Bio spectrometer equipped with a CuveT thermostat unless otherwise specified.

3.4.2 UV–Vis Spectroscopy and PSS Composition

Stock solutions (10 mM in DMSO) were diluted to 100 µM in DMSO, MeCN, MeOH, PBS buffer (pH 7.4), or distilled water. Spectra were recorded in 1 mL quartz cuvettes (1 cm path length) in the dark. Background correction was performed using solvent-only controls. Samples were

irradiated sequentially at 340, 365, 415, 455, 505, and 530 nm (30 min at 340 nm; 20 min at other wavelengths). Spectra were analyzed using a custom MATLAB R2020b script to determine absorption maxima and isosbestic points.

PSS compositions were quantified by HPLC (Waters e2965 with 2998 PDA detector) using 10 µL injections of 100 µM solutions. Separation was achieved on a ReprosilChiral-NR column with MeCN–water mixtures containing 0.1% formic acid. UV–vis spectra of separated isomers were overlaid to determine isosbestic wavelengths, and chromatograms were integrated at these wavelengths.

For $(OEt)_2$-substituted arylazopyrazole photoPROTACs, PSS compositions were determined by ^1H NMR. Samples (5 mg in CD_3SOCD_3) were irradiated for 30 min at 370, 440, or 525 nm using Kessil lamps. Spectra (32 scans, d_1 = 10 s) were recorded immediately and after an additional 10 min to confirm equilibrium.

3.4.3 Thermal Relaxation and Half-Life Determination

Samples were irradiated at the wavelength yielding the highest Z-isomer content (typically 340 or 365 nm), then monitored at the π–π* λ_max over time at controlled temperatures. Decay curves were fitted to a first-order exponential model using MATLAB R2020b:

$$[Z]_t = Ae^{-kt}$$

where $[Z]_t$ is the concentration of the Z-isomer at time *t*, *k* is the rate constant, and *A* is a scaling factor. For compounds with measurable decay at 25 °C, thermal half-lives were calculated as:

$$t_{1/2} = \frac{\ln 2}{k}$$

For compounds with slow relaxation (half-lives of several days to weeks), rate constants were extrapolated using the Eyring equation:

$$ln\left(\frac{k}{T}\right) = -\frac{\Delta H^{\ddagger}}{RT} + ln\left(\frac{k_B}{h}\right) + \frac{\Delta S^{\ddagger}}{R}$$

Activation parameters ($\Delta H^{\ddagger}$, $\Delta S^{\ddagger}$) were extracted from linear regression of rate constants versus temperature, and thermal half-lives at 25 °C were calculated from the extrapolated values.

3.5 Photostability

Photostability was assessed by subjecting 100 µM solutions of each PhotoPROTAC to repeated irradiation cycles. For all compounds, a 15-minute irradiation at the specified wavelength was sufficient to reach the corresponding photostationary state (PSS). Samples were alternately irradiated at the respective switching wavelengths, and a UV–vis absorption spectrum was recorded after each cycle.

The absorbance at the π–π* λ_max of each compound was extracted after every irradiation step and plotted using a custom MATLAB R2020b script to visualize changes in absorbance over time. The absence of photobleaching was confirmed by the reproducibility of the absorbance values and the ability to return to the same PSS after each cycle.

4. Synthetic Procedures

Arylazotriazole–dexa-linked photoPROTACs

Synthesis of 39

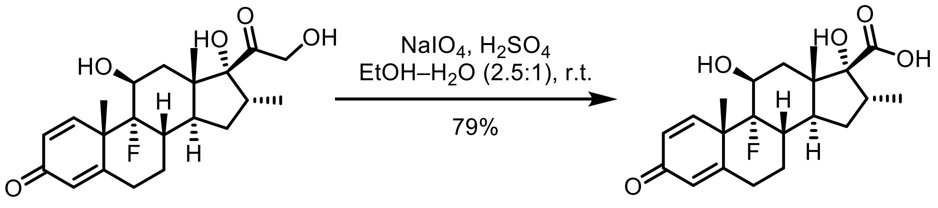

dexamethasone (SI-1) **39**

To a solution of **SI-1** (2.0 g, 5.1 mmol, 1.0 equiv) in EtOH (400 mL) was added water (157 mL) followed by sodium periodate (1.3 g, 6.0 mmol, 1.2 equiv) and sulfuric acid (2.0 M, 10.2 mL, 20.4 mmol, 4.0 equiv). After stirring the resulting reaction solution at r.t. overnight, the reaction mixture was concentrated *in vacuo*. Water and brine were added and the reaction mixture was basified to a pH of 12 with aq. NaOH to dissolve the white precipitate. The aqueous layer was washed with CH_2Cl_2, acidified with 1 M $NaHSO_4$ to a pH of 3 and extracted with EtOAc. The combined organic layers were washed with brine, dried over Na_2SO_4, filtered, and concentrated *in vacuo* to yield **39** (1.5 g, 79%) as a white solid.

^{1}H NMR (500 MHz, CD_3OD): δ = 7.42 (d, *J* = 10.2 Hz, 1H), 6.28 (dd, *J* = 10.1, 1.9 Hz, 1H), 6.08 (d, *J* = 1.8 Hz, 1H), 4.27 – 4.22 (m, 1H), 3.04 – 2.96 (m, 1H), 2.78 – 2.66 (m, 1H), 2.53 – 2.36 (m, 2H), 2.19 – 2.10 (m, 2H), 1.93 – 1.84 (m, 1H), 1.75 (dt, *J* = 13.1, 11.0 Hz, 1H), 1.62 – 1.46 (m, 5H), 1.26 – 1.17 (m, 1H), 1.14 (s, 3H), 0.94 (dd, *J* = 7.2, 0.8 Hz, 3H) ppm.

^{13}C NMR (126 MHz, CD_3OD): δ = 189.0, 176.9, 171.2, 156.1, 129.8, 125.1, 103.2, 101.8, 87.7, 73.1 (d, *J* = 37.3 Hz), 50.3 (d, *J* = 22.6 Hz), 44.3, 37.2, 37.0, 35.7 (d, *J* = 19.3 Hz), 33.6, 32.3, 28.8, 23.6 (d, *J* = 5.7 Hz), 17.7, 15.4 ppm.

^{19}F NMR (471 MHz, CD_3OD): δ = -166.08 ppm.

IR (neat): 3442, 2943, 2873, 1079, 1660, 1602, 1452, 1395, 1377, 1353, 1300, 1242, 1035, 892 cm^{-1}.

HRMS (ESI): *m/z* calcd. for $C_{21}H_{28}FO_5$ $[M+H]^+$ 379.1915, found 379.1916.

$[a]^{25}_D$ = 55.1 (c = 1.0, CH_3OH).

Synthesis of 15

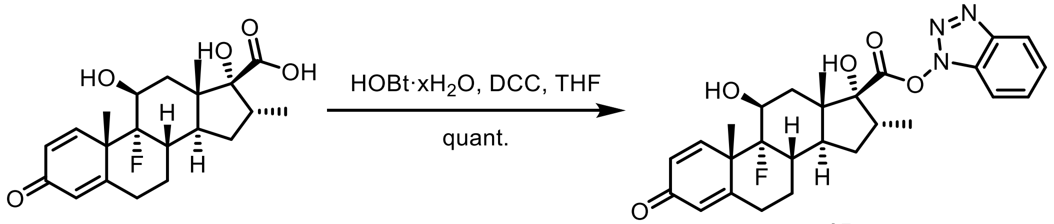

DCC (1.0 M in CH_2Cl_2, 1.7 mL, 1.7 mmol, 1.3 equiv) was added to a mixture of **39** (0.50 g, 1.3 mmol, 1.0 equiv) and HOBt·xH_2O (0.35 mg, 2.3 mmol, 1.7 equiv) in anhydrous THF (50 mL) at 0 °C. After stirring the resulting reaction suspension at 4 °C overnight, the reaction mixture was concentrated *in vacuo*. Column chromatography (SiO_2, eluent: EtOAc–hexanes (1:1) to EtOAc) afforded **15** (874 mg, quant.) as an off-white solid.

^{1}H NMR (500 MHz, $(CD_3)_2CO$): δ = 8.10 (dt, *J* = 8.5, 0.9 Hz, 1H), 7.69 – 7.66 (m, 2H), 7.55 – 7.51 (m, 1H), 7.34 (dd, *J* = 10.1, 0.5 Hz, 1H), 6.22 (dd, *J* = 10.1, 1.9 Hz, 1H), 6.03 (t, *J* = 1.8 Hz, 1H), 5.28 (s, 1H), 4.70 (s, 1H), 4.49 (dt, *J* = 10.1, 3.5 Hz, 1H), 3.13 (dqd, *J* = 11.0, 7.2, 3.9 Hz, 1H), 2.77 (tdd, *J* = 13.8, 6.1, 1.8 Hz, 1H), 2.62 (dtd, *J* = 29.1, 11.9, 4.9 Hz, 1H), 2.48 (dt, *J* = 15.0, 3.7 Hz, 1H), 2.43 – 2.35 (m, 2H), 1.98 – 1.88 (m, 2H), 1.66 (s, 3H), 1.62 – 1.51 (m, 2H), i1.40 (d, *J* = 0.8 Hz, 3H), 1.36 (ddd, *J* = 12.3, 8.2, 3.9 Hz, 1H), 1.14 (d, *J* = 7.2 Hz, 3H) ppm.

^{13}C NMR (126 MHz, $(CD_3)_2CO$): δ = 186.0, 170.7, 166.8 (d, *J* = 1.7 Hz), 152.7, 144.3, 130.3, 129.9, 129.5, 125.9, 125.5 (d, *J* = 1.4 Hz), 121.01 109.6, 102.3, 101.0, 87.6, 72. 6 (d, *J* = 37.3 Hz), 49.5, 49.1 (d, *J* = 22.9 Hz), 43.9 (d, *J* = 1.7 Hz), 38.4 – 37.3 (m), 36. 8, 35.2 (d, *J* = 19.4 Hz), 33.2, 31.5, 28.4 (d, *J* = 1.8 Hz), 23.8 (d, *J* = 5.8 Hz), 17.8, 15.3 ppm.

^{19}F NMR (471 MHz, $(CD_3)_2CO$): δ = -166.02 ppm.

IR (neat): 3383, 2936, 1809, 1662, 1619, 1452, 1375, 1298, 1241, 1142, 1087, 893, 743 cm^{-1}.

HRMS (ESI): *m/z* calcd. for $C_{27}H_{34}FN_4O_5$ $[M+NH_4]^+$ 513.2508, found 513.2509.

[a]^{25}$_D$ = 108.3 (c = 0.5, CH_3OH).

Synthesis of 17

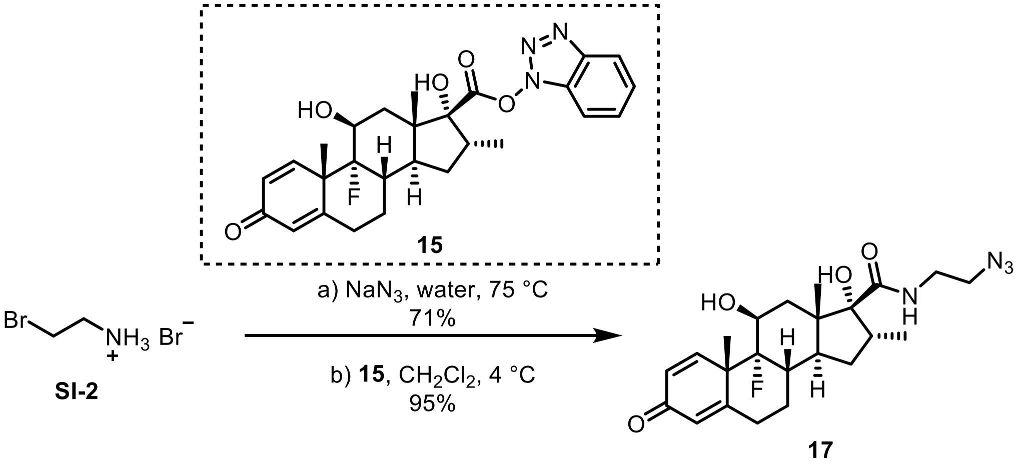

NaN$_3$ (1.9 g, 29 mmol, 3.0 equiv) was added to a solution of **SI-2** (2.0 g, 9.8 mmol, 1.0 equiv) in water (10 mL). After stirring the resulting reaction solution at 75 °C overnight, the reaction mixture was cooled down to 0 °C and 50 M aq. KOH (2 mL) was added. The aqueous layer was extracted thrice with ether and the combined organic layers were dried over Na$_2$SO$_4$, filtered, and concentrated cautiously by needle evaporation to afford **16** as a colorless solution in ether (49.7% in ether, 0.6 g, 71%).

16 (49.7% in ether, 15.4 µL, 63.6 µmol, 1.05 equiv) was added to a solution of **15** (30 mg, 0.6 mmol, 1.0 equiv) in CH$_2$Cl$_2$ (0.5 mL) at 0 °C. After stirring the resulting reaction suspension at 4 °C overnight, the reaction mixture was concentrated *in vacuo*. Column chromatography (SiO$_2$, eluent: 50% EtOAc in hexanes) yielded **17** (25.8 mg, 95%) as an off-white solid.

^{1}H NMR (500 MHz, CD$_3$OD): δ = 7.42 (d, *J* = 10.1 Hz, 1H), 6.28 (dd, *J* = 10.1, 1.9 Hz, 1H), 6.08 (t, *J* = 1.8 Hz, 1H), 4.24 (ddd, *J* = 11.0, 4.2, 2.1 Hz, 1H), 3.50 – 3.39 (m, 3H), 3.35 – 3.32 (m, 1H), 3.12 (ddt, *J* = 11.3, 7.4, 3.6 Hz, 1H), 2.72 (tdd, *J* = 13.8, 6.3, 1.8 Hz, 1H), 2.51 – 2.36 (m, 2H), 2.25 – 2.15 (m, 2H), 1.92 – 1.86 (m, 1H), 1.76 (tdd, *J* = 12.3, 11.0, 1.6 Hz, 1H), 1.59 (s, 3H), 1.57 – 1.46 (m, 2H), 1.20 (ddd, *J* = 12.4, 8.3, 4.3 Hz, 1H), 1.09 (d, *J* = 0.7 Hz, 3H), 0.90 (d, *J* = 7.3 Hz, 3H) ppm.

^{13}C NMR (125 MHz, CD$_3$OD): δ = 189.1, 176.1, 171.2 (d, *J* = 1.7 Hz), 156.1, 129.8, 125.1 (d, *J* = 1.4 Hz), 103.3, 101.9, 88.2, 73.1 (d, *J* = 37.5 Hz), 51.7, 50.4 (d, *J* = 22.8 Hz), 44.8 (d, *J* = 1.6 Hz), 39.5, 36.8 (d, *J* = 1.5 Hz), 36.4, 35.8 (d, *J* = 19.3 Hz), 33.4, 32.3, 28.8 (d, *J* = 1.9 Hz), 23.6 (d, *J* = 5.7 Hz), 17.7, 15.1 ppm.

^{19}F NMR (471 MHz, CD$_3$OD): δ = -165.95 ppm.

IR (neat): 3376, 2933, 2869, 2102, 1661, 1624, 1521, 1451, 1299, 1242, 1126, 1035, 892 cm^{-1}.

HRMS (ESI): *m/z* calcd. for C$_{23}$H$_{32}$FN$_4$O$_4$ [M+H]$^+$ 447.2402, found 447.2410.

[a]$^{25}_D$ = 70.3 (c = 0.7, CH$_3$OH).

Synthesis of KH-5-168

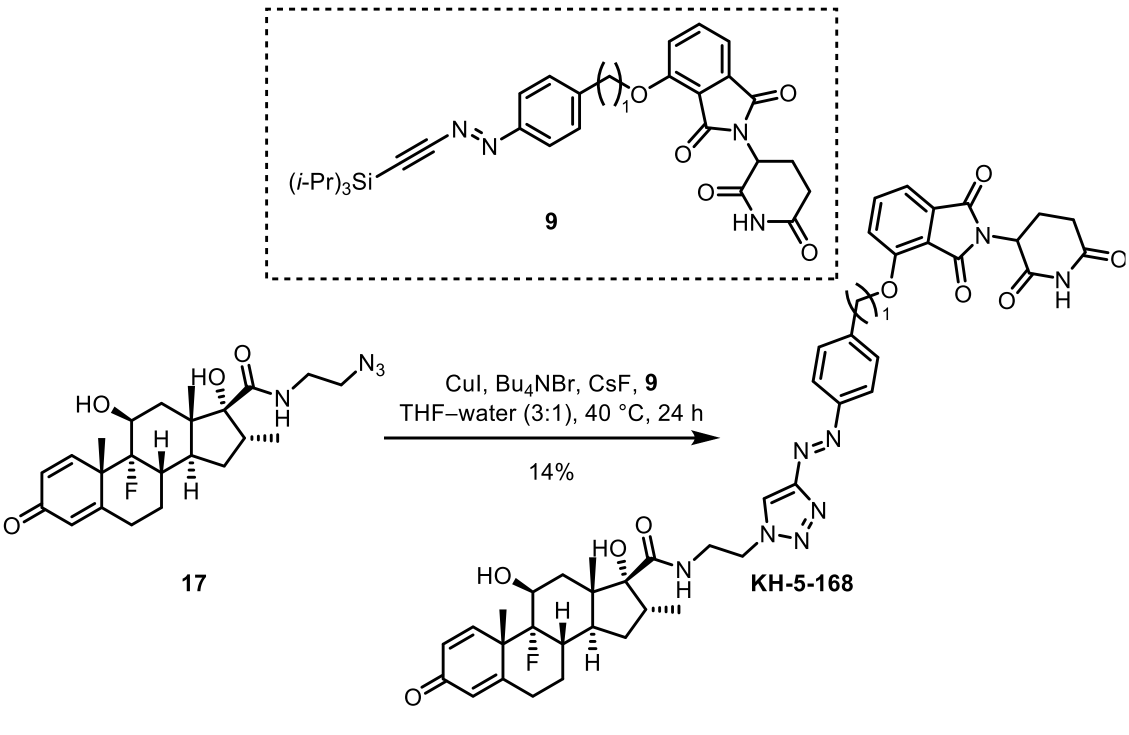

A vial was charged with **17** (5.2 mg, 12 µmol, 1.1 equiv), **9** (6.0 mg, 11 µmol, 1.0 equiv), Bu_4NBr (3.7 mg, 12 µmol, 1.1 equiv) and CuI (0.6 mg, 3.1 µmol, 0.3 equiv). THF (80 µL) and water (27 µL) were added followed by CsF (1.5 M in water, 7.7 µL, 12 µmol, 1.1 equiv) at r.t. The reaction mixture was warmed to 40 °C and stirred at 40 °C overnight. Filtration trough a plug of silica using EtOAc as eluent, removal of all solvents followed by column chromatography (SiO_2, eluent: 10% MeOH in EtOAc) and preparative TLC (SiO_2, eluent: 20% MeOH in EtOAc) yielded **KH-5-168** (1.30 mg, 14%) as an orange-red solid.

^{1}H NMR (600 MHz, CD_3OD): δ = 8.49 (s, 1H), 7.99 – 7.93 (m, 2H), 7.80 – 7.72 (m, 3H), 7.52 (dd, *J* = 8.5, 0.7 Hz, 1H), 7.48 (dd, *J* = 7.3, 0.6 Hz, 1H), 7.37 (d, *J* = 10.1 Hz, 1H), 6.26 (ddd, *J* = 10.1, 1.9, 0.9 Hz, 1H), 6.06 (t, *J* = 1.7 Hz, 1H), 5.48 (s, 2H), 5.19 – 5.11 (m, 1H), 4.64 (t, *J* = 5.8 Hz, 2H), 4.20 (d, *J* = 11.1 Hz, 1H), 3.89 – 3.82 (m, 1H), 3.69 (dt, *J* = 14.3, 5.8 Hz, 1H), 3.04 (ddd, *J* = 11.3, 7.4, 4.2 Hz, 1H), 2.88 (ddd, *J* = 18.1, 14.3, 5.0 Hz, 1H), 2.80 – 2.66 (m, 2H), 2.47 – 2.35 (m, 2H), 2.16 – 2.11 (m, 3H), 1.89 – 1.84 (m, 1H), 1.71 (q, *J* = 11.9 Hz, 1H), 1.58 (s, 4H), 1.51 (td, *J* = 13.1, 5.4 Hz, 1H), 1.37 – 1.33 (m, 1H), 1.17 (ddt, *J* = 12.4, 8.3, 3.9 Hz, 1H), 1.01 (s, 3H), 0.86 (s, 1H) ppm.

^{13}C NMR (126 MHz, CD_3OD): δ = 189.1, 176.3, 174.6, 171.5, 171.2, 168.6, 167.3, 161.5, 157.2, 156.0, 153.6, 141.9, 138.0, 135.3, 129.8, 129.0, 125.1, 124.1, 121.2, 118.9, 118.3, 117.0, 103.1, 102.0, 88.2, 73.1 (d, *J* = 37.5 Hz), 71.2, 51.6, 50.5, 50.3 (d, *J* = 22.6 Hz), 44.9, 40.3, 36.7, 36.5, 35.8, 35.7, 33.3, 32.2, 32.2, 28.8, 24.3 – 22.9 (m), 17.6, 15.1 ppm.

^{19}F NMR (471 MHz, CD_3OD): δ = -166.09 ppm.

IR (neat): 3417, 2942, 2873, 1772, 1714, 1663, 1615, 1394, 1287, 1261, 1197, 1042, 893 cm^{-1}.

HRMS (ESI): *m/z* calcd. for $C_{45}H_{48}FN_8O_9$ $[M+H]^+$ 863.3523, found 863.3519.

Synthesis of KH-5-169

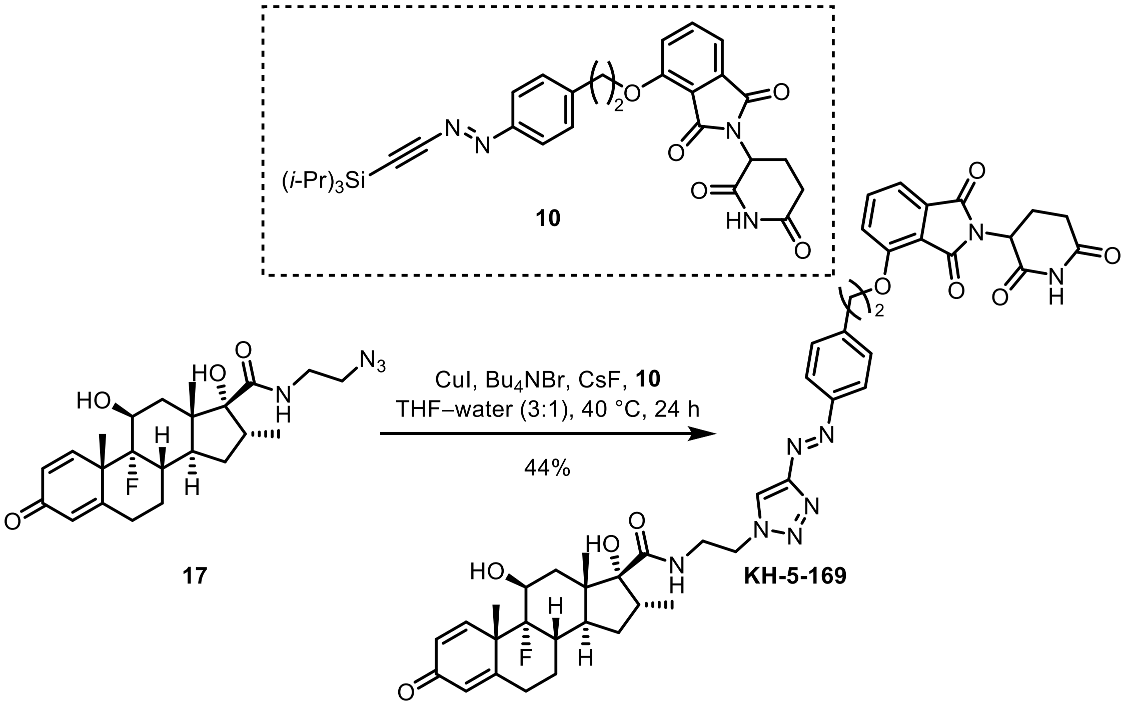

A vial was charged with **17** (3.5 mg, 7.8 µmol, 1.0 equiv), **10** (6.9 mg, 0.12 mmol, 1.5 equiv), Bu_4NBr (3.4 mg, 0.11 mmol, 1.3 equiv) and CuI (0.5 mg, 2.4 µmol, 0.3 equiv). THF (58 µL) and water (11 µL) were added followe by CsF (1.5 M in water, 7.0 µL, 11 µmol, 1.3 equiv) at r.t. The reaction mixture was warmed to 40 °C and stirred at 40 °C overnight. Filtration trough a plug of silica using EtOAc as eluent, removal of all solvents followed by column chromatography (SiO_2, eluent: 10% MeOH in EtOAc) and preparative TLC (SiO_2, eluent: 20% MeOH in EtOAc) yielded **KH-5-169** (3.0 mg, 44%) as an orange-red solid.

^{1}H NMR (600 MHz, CD_3OD): δ = 8.45 (s, 1H), 7.90 – 7.80 (m, 2H), 7.73 (dd, *J* = 8.4, 7.3 Hz, 1H), 7.63 (d, *J* = 8.3 Hz, 2H), 7.46 – 7.38 (m, 2H), 7.34 (dd, *J* = 10.1, 3.1 Hz, 1H), 6.24 (dt, *J* = 10.1, 1.8 Hz, 1H), 6.05 (t, *J* = 1.7 Hz, 1H), 5.15 – 5.08 (m, 1H), 4.63 (t, *J* = 5.8 Hz, 2H), 4.47 (t, *J* = 6.3 Hz, 2H), 4.25 – 4.16 (m, 1H), 3.89 – 3.80 (m, 1H), 3.73 – 3.65 (m, 1H), 3.26 (t, *J* = 6.2 Hz, 2H), 3.03 (ddd, *J* = 11.5, 7.5, 4.3 Hz, 1H), 2.94 – 2.83 (m, 1H), 2.81 – 2.64 (m, 3H), 2.48 – 2.33 (m, 2H), 2.17 – 2.08 (m, 3H), 1.85 (dd, *J* = 12.6, 5.9 Hz, 1H), 1.71 (q, *J* = 11.7 Hz, 1H), 1.56 (d, *J* = 1.0 Hz, 3H), 1.49 (qd, *J* = 13.1, 5.3 Hz, 1H), 1.32 (d, *J* = 14.1 Hz, 2H), 1.16 (ddd, *J* = 12.3, 8.3, 4.2 Hz, 1H), 1.00 (s, 3H), 0.85 (d, *J* = 7.3 Hz, 3H) ppm.

^{13}C NMR (126 MHz, CD_3OD): δ = 189.1, 176.3, 174.6, 171.5, 171.2, 168.6, 167.2, 161.5, 157.6, 156.1, 152.7, 144.5, 138.0, 135.1, 131.6, 129.8, 125.1, 123.9, 120.5, 118.3, 118.1, 116.6, 103.1, 102.0, 88.2, 73.1 (d, *J* = 37.9 Hz), 70.9, 51.5, 50.4, 50.3 (d, *J* = 22.7 Hz), 44.9, 40.3, 36. 7, 36.5, 36.39, 35.8, 35. 7, 33.3, 32.2 (d, *J* = 4.5 Hz), 28. 8, 24.1 – 23.0 (m), 17.6, 15.1 ppm.

^{19}F NMR (471 MHz, CD_3OD): δ = -166.02 ppm.

IR (neat): 3418, 2942, 2873, 1772, 1714, 1662, 1615, 1395, 1287, 1261, 1197, 1037, 893 cm^{-1}.

HRMS (ESI): *m/z* calcd. for $C_{46}H_{50}FN_8O_9$ $[M+H]^+$ 877.3679, found 877.3680.

Synthesis of KH-5-170

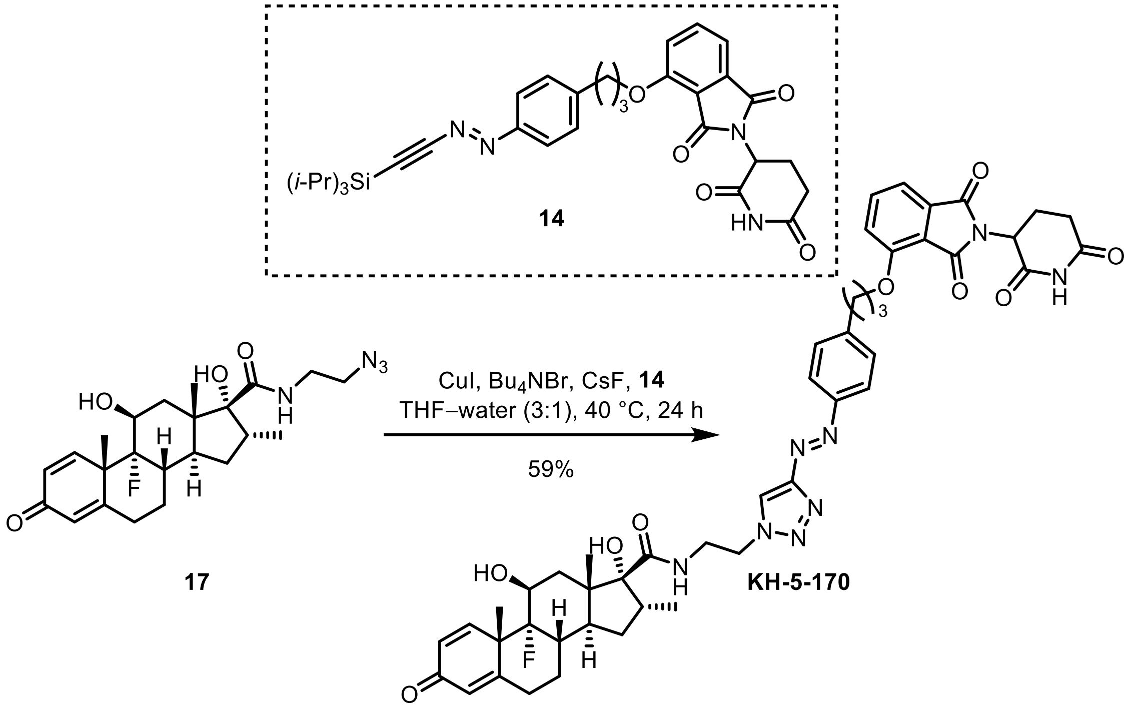

A vial was charged with **17** (3.9 mg, 8.7 µmol, 1.0 equiv), **14** (7.9 mg, 13 µmol, 1.5 equiv), *n*-Bu_4NBr (3.9 mg, 12 µmol, 1.4 equiv) and CuI (0.5 mg, 2.6 µmol, 0.3 equiv). THF (0.65 mL) and water (0.13 mL) were added followed by CsF (1.5 M in water, 8.0 µL, 12 µmol, 1.4 equiv) at r.t. The reaction mixture was warmed to 40 °C and stirred at 40 °C overnight. Filtration trough a plug of silica using EtOAc as eluent, removal of all solvents followed by column chromatography (SiO_2, eluent: 10% MeOH in EtOAc) and preparative TLC (SiO_2, eluent: 20% MeOH in EtOAc) afforded **KH-5-170** (4.6 mg, 59%) as an orange-red solid.

^{1}H NMR (600 MHz, CD_3OD): δ = 8.44 (s, 1H), 7.86 – 7.78 (m, 2H), 7.76 – 7.70 (m, 1H), 7.47 – 7.40 (m, 3H), 7.40 – 7.31 (m, 2H), 6.27 – 6.21 (m, 1H), 6.05 (t, *J* = 1.8 Hz, 1H), 5.15 – 5.06 (m, 1H), 4.63 (t, *J* = 5.8 Hz, 2H), 4.23 (t, *J* = 6.1 Hz, 2H), 4.18 (ddd, *J* = 11.1, 4.2, 2.1 Hz, 1H), 3.89 – 3.79 (m, 1H), 3.72 – 3.65 (m, 1H), 3.03 (qq, *J* = 7.3, 3.7, 3.1 Hz, 1H), 2.98 (t, *J* = 7.5 Hz, 2H), 2.92 – 2.82 (m, 1H), 2.80 – 2.64 (m, 3H), 2.46 – 2.30 (m, 2H), 2.25 – 2.09 (m, 6H), 1.84 (dt, *J* = 12.2, 5.6 Hz, 1H), 1.70 (tdd, *J* = 12.3, 11.1, 1.6 Hz, 1H), 1.56 (s, 3H), 1.48 (qd, *J* = 12.7, 5.1 Hz, 1H), 1.34 (dd, *J* = 14.1, 2.1 Hz, 2H), 1.15 (ddd, *J* = 12.4, 8.3, 4.3 Hz, 1H), 1.00 (s, 3H), 0.85 (d, *J* = 7.4 Hz, 3H) ppm.

^{13}C NMR (126 MHz, CD_3OD): δ = 189.1, 176.3, 174.7, 171.5, 171.2, 168.7, 167.4, 161.5, 157.8, 156.1, 152.4, 147.7, 138.0, 135.1, 130.7, 129.8, 125.1, 124.1, 120.6, 118.3, 118.0, 116.5, 103.1, 102.0, 88.2, 73.1 (d, *J* = 37.7 Hz), 69.3, 51.5, 50.4, 50.3 (d, *J* = 22.8 Hz), 44.9, 40.3, 36.7, 36.5, 35.8, 35.7, 33.3, 32.8, 32.2 (d, *J* = 4.5 Hz), 31.4, 28.8, 23.8 – 23.4 (m), 17.6, 15.2 ppm.

^{19}F NMR (471 MHz, CD_3OD): δ = -166.05 ppm.

IR (neat): 3416, 2942, 2873, 1771, 1713, 1663, 1615, 1487, 1394, 1287, 1261, 1197, 1045, 893 cm^{-1}.

HRMS (ESI): *m/z* calcd. for $C_{47}H_{51}FN_8O_9Na$ $[M+Na]^+$ 913.3655, found 913.3647.

Arylazotriazole–lenalidomide-linked photoPROTACs

Synthesis of 22

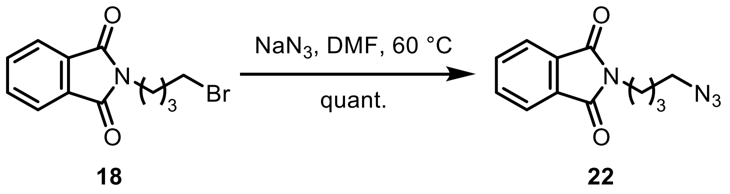

NaN$_3$ (0.98 g, 15 mmol, 3.0 equiv) was added to a solution of **18** (1.4 g, 5.0 mmol, 1.0 equiv) in DMF (10 mL). After stirring the resulting reaction solution at 60 °C for 6 h, the reaction mixture was diluted with water and brine. The aqueous layer was extracted three times with ether–hexanes (2:1) and the combined organic layers were washed with brine, dried over Na$_2$SO$_4$, filtered, and concentrated *in vacuo*. Column chromatography (SiO$_2$, eluent: 5% to 10% EtOAc in hexanes) afforded azide **22** (1.2 g, quant.) as a white solid.

^{1}H NMR (400 MHz, CDCl$_3$): δ = 7.86 – 7.80 (m, 2H), 7.71 (dd, *J* = 5.4, 3.1 Hz, 2H), 3.74 – 3.67 (m, 2H), 3.32 (t, *J* = 6.8 Hz, 2H), 1.82 – 1.71 (m, 2H), 1.70 – 1.58 (m, 2H) ppm.

^{13}C NMR (101 MHz, CDCl$_3$): δ = 168.5, 134.1, 132.2, 123.4, 51.0, 37.4, 26.4, 26.0 ppm.

IR (neat): 2941, 2869, 2092, 1771, 1704, 1614, 1467, 1437, 1395, 1335, 1289, 1260, 1187, 1042, 865, 716 cm^{-1}.

HRMS (ESI): *m/z* calcd. for C$_{12}$H$_{12}$N$_4$O$_2$Na [M+Na]$^+$ 267.0852, found 267.0850.

Synthesis of 23

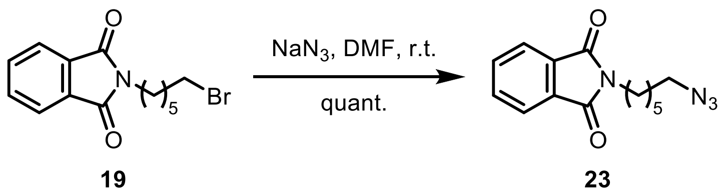

NaN$_3$ (0.78 g, 12 mmol, 3.0 equiv) was added to a solution of **19** (1.2 g, 4.0 mmol, 1.0 equiv) in DMF (8 mL). After stirring the resulting reaction solution at r.t. overnight, the reaction mixture was diluted with water and brine. The aqueous layer was extracted three times with ether–hexanes (2:1) and the combined organic layers were washed with brine, dried over Na_2SO_4, filtered, and concentrated *in vacuo*. Column chromatography (SiO_2, eluent: 5% to 10% EtOAc in hexanes) yielded azide **23** (1.1 g, quant.) as a colorless oil.

^{1}H NMR (400 MHz, $CDCl_3$): δ = 7.82 (dd, *J* = 5.4, 3.1 Hz, 2H), 7.69 (dd, *J* = 5.5, 3.0 Hz, 2H), 3.66 (t, *J* = 7.2 Hz, 2H), 3.23 (t, *J* = 6.9 Hz, 2H), 1.73 – 1.62 (m, 2H), 1.62 – 1.53 (m, 2H), 1.45 – 1.30 (m, 4H) ppm.

^{13}C NMR (101 MHz, $CDCl_3$): δ = 168.5, 134.0, 132.2, 123.3, 51.4, 37.9, 28.8, 28.5, 26.5, 26.5 ppm.

IR (neat): 2936, 2861, 2092, 1772, 1706, 1615, 1467, 1436, 1395, 1366, 1261, 1245, 1055, 716 cm^{-1}.

HRMS (ESI): *m/z* calcd. for $C_{14}H_{16}N_4O_2Na$ $[M+Na]^+$, 295.1165 found 295.1164.

Synthesis of 24

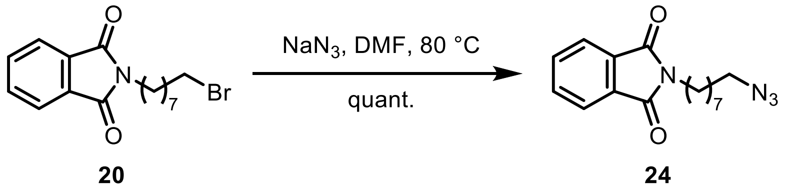

NaN$_3$ (0.58 mg, 8.9 mmol, 3.0 equiv) was added to a solution of **20** (1.0 g, 3.0 mmol, 1.0 equiv) in DMF (6 mL). After stirring the resulting reaction solution at 80 °C for 6 h, the reaction mixture was diluted with water and brine. The aqueous layer was extracted three times with ether–hexanes (2:1) and the combined organic layers were washed with brine, dried over Na_2SO_4, filtered, and concentrated *in vacuo*. Column chromatography (SiO_2, eluent: 5% to 10% EtOAc in hexanes) yielded azide **24** (1.2 g, quant.) as a colorless oil.

^{1}H NMR (400 MHz, $CDCl_3$): δ = 7.85 – 7.79 (m, 2H), 7.69 (dd, *J* = 5.5, 3.0 Hz, 2H), 3.66 (t, *J* = 7.3 Hz, 2H), 3.22 (t, *J* = 7.0 Hz, 2H), 1.72 – 1.61 (m, 2H), 1.61 – 1.51 (m, 2H), 1.33 (d, *J* = 9.0 Hz, 8H) ppm.

^{13}C NMR (101 MHz, $CDCl_3$): δ = 168.5, 133.9, 132.3, 123.2, 51.52, 38.1, 29.1 (d, *J* = 1.2 Hz), 28.9, 28.6, 26.8, 26.7 ppm.

IR (neat): 2931, 2857, 2092, 1772, 1707, 1615, 1467, 1437, 1394, 1364, 1289, 1257, 1188, 1062, 936, 877, 717 cm^{-1}.

HRMS (ESI): *m/z* calcd. for $C_{16}H_{20}N_4O_2Na$ $[M+Na]^+$, 323.1478 found 323.1482.

Synthesis of 25

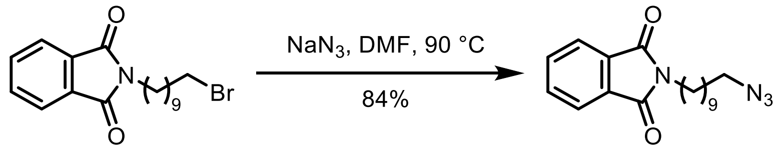

NaN_3 (0.80 mg, 12 mmol, 3.0 equiv) was added to a solution of **21** (1.5 g, 4.1 mmol, 1.0 equiv) in DMF (8.3 mL). After stirring the resulting reaction solution at 90 °C overnight, the reaction mixture was diluted with water and brine. The aqueous layer was extracted three times with ether–hexanes (2:1) and the combined organic layers were washed with brine, dried over Na_2SO_4, filtered, and concentrated *in vacuo*. Column chromatography (SiO_2, eluent: 5% to 10% EtOAc in hexanes) yielded azide **25** (1.2 g, 84%) as a colorless oil.

^{1}H NMR (400 MHz, $CDCl_3$): δ = 7.82 (dt, *J* = 7.6, 3.8 Hz, 2H), 7.74 – 7.66 (m, 2H), 3.71 – 3.61 (m, 2H), 3.23 (t, *J* = 7.0 Hz, 2H), 1.66 (p, *J* = 7.4 Hz, 2H), 1.57 (p, *J* = 7.0 Hz, 2H), 1.41 – 1.21 (m, 12H) ppm.

^{13}C NMR (101 MHz, $CDCl_3$): δ = 168.6, 134.0, 132.3, 123.3, 51.6, 38.2, 29.5 (d, *J* = 2.3 Hz), 29.2 (d, *J* = 2.3 Hz), 28.9, 28.7, 26.9, 26.8 ppm.

IR (neat): 2927, 2855, 2092, 1772, 1709, 1615, 1467, 1437, 1394, 1366, 1287, 1260, 1188, 1070, 960, 890, 718 cm^{-1}.

HRMS (ESI): *m/z* calcd. for $C_{18}H_{24}N_4O_2Na$ $[M+Na]^+$, 351.1791 found 351.1787.

Synthesis of 30

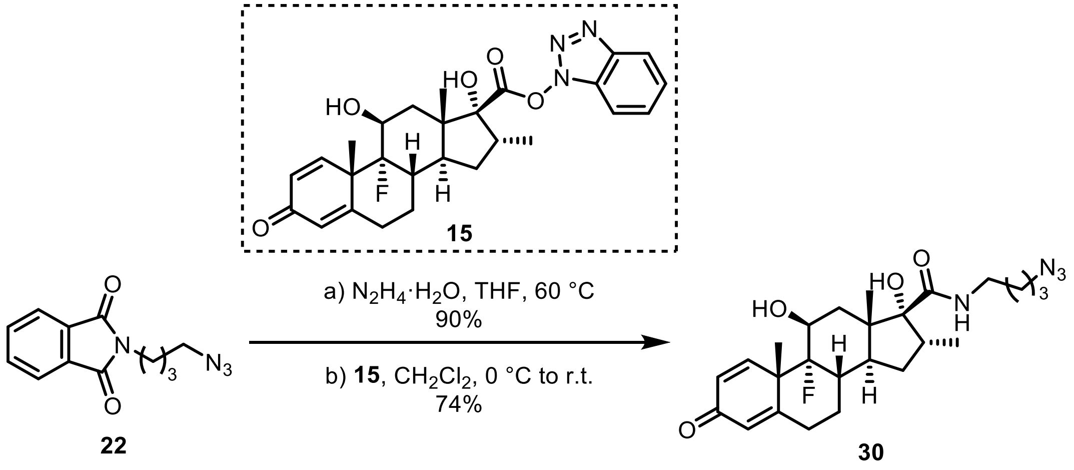

$N_2H_4 \cdot H_2O$ (0.98 g, 15 mmol, 3.0 equiv) was added to a solution of **22** (82 mg, 0.3 mmol, 1.0 equiv) in THF (3 mL). After stirring the resulting reaction solution at 60 °C for 6 h, the reaction mixture was cooled down to r.t. and diluted with CH_2Cl_2. The organic layer was then washed three times with 0.2 M HCl. The aqueous layer was basified until pH ≥ 14 with 1.0 M NaOH and extracted thrice with CH_2Cl_2. The combined organic layers were dried over Na_2SO_4, filtered, and cautiously concentrated by needle evaporation. The crude product **26** (28.4 mg, 90%) was received as a yellowish oil and used for the next step without further purification.

Amine **26** (17.3 mg, 151 µmol, 1.5 equiv) was added to a solution of **15** (50 mg, 0.10 mmol, 1.0 equiv) in CH_2Cl_2 (1 mL) at 0 °C. After stirring the resulting reaction solution at 0 °C overnight, the reaction mixture was allowed to warm to r.t. and stirred for another hour at r.t. The solvent was removed *in vacuo* and the crude product was purified by column chromatography (SiO_2, eluent: EtOAc–hexanes (1:1) to EtOAc–hexanes (2:1)) and preparative TLC (SiO_2, eluent: EtOAc–hexanes (2:1)) to afford azide **30** (35.2 mg, 74% ()) as a colorless foam.

^{1}H NMR (400 MHz, $CDCl_3$): δ = 7.24 (dd, *J* = 10.2, 1.3 Hz, 1H), 6.72 (q, *J* = 4.7, 3.4 Hz, 1H), 6.29 (dd, *J* = 10.1, 2.0 Hz, 1H), 6.08 (t, *J* = 1.7 Hz, 1H), 4.32 (dd, *J* = 10.1, 4.9 Hz, 1H), 3.42 – 3.09 (m, 5H), 2.67 – 2.50 (m, 2H), 2.33 (dddd, *J* = 43.2, 27.0, 13.0, 4.0 Hz, 4H), 2.12 (td, *J* = 12.2, 11.8, 8.4 Hz, 1H), 1.88 – 1.67 (m, 2H), 1.66 – 1.46 (m, 8H), 1.39 (dd, *J* = 14.3, 2.0 Hz, 1H), 1.22 (dt, *J* = 12.4, 3.6 Hz, 1H), 1.11 (s, 3H), 0.92 (d, *J* = 7.3 Hz, 3H) ppm.

^{13}C NMR (101 MHz, $CDCl_3$): δ = 186.9, 172.8, 167.0, 152.9, 129.7, 125.0, 101.7, 99.9, 87.1, 72.3 (d, *J* = 38.6 Hz), 51.1, 48.6 (d, *J* = 22.3 Hz), 48.1, 43.9 (d, *J* = 1.6 Hz), 38.8, 36.6, 35.4, 34.5 (d, *J* = 19.5 Hz), 32.3, 31.2, 27.5, 26.8 (d, *J* = 90.2 Hz), 23.0 (d, *J* = 5.7 Hz), 17.5, 14.6 ppm.

^{19}F NMR (376 MHz, $CDCl_3$): δ = -165.41 ppm.

IR (neat): 3394, 2941, 2872, 2097, 1662, 1525, 1452, 1296, 1242, 1073, 1035, 1013, 893 cm^{-1}.

HRMS (ESI): *m/z* calcd. for $C_{25}H_{36}FN_4O_4$ $[M+H]^+$ 475.2715, found 475.2708.

Synthesis of 31

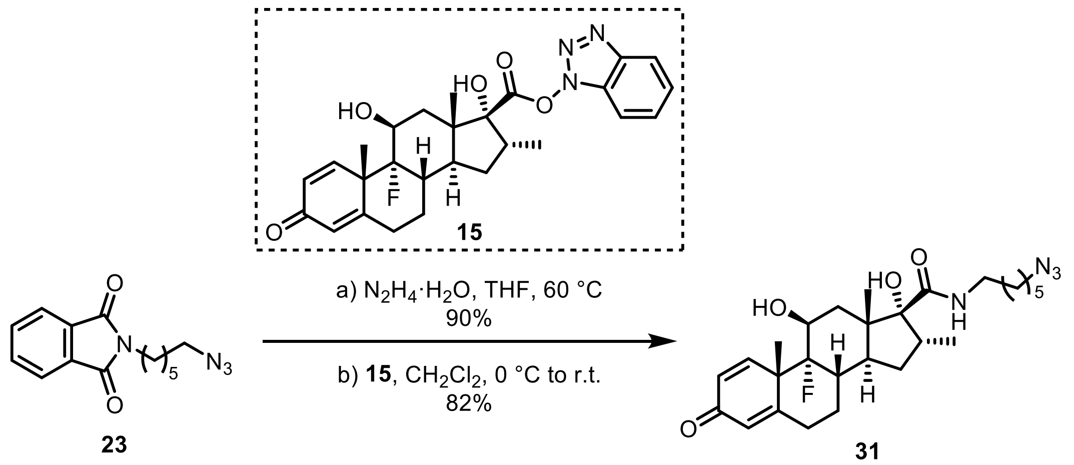

$N_2H_4 \cdot H_2O$ (0.21 µL, 2.1 mmol, 7.0 equiv) was added to a solution of **23** (82 mg, 0.31 µmol, 1.0 equiv) in THF (3 mL). After stirring the resulting reaction solution at 60 °C for 6 h, the reaction mixture was cooled down to r.t. and diluted with CH_2Cl_2. The organic layer was then washed three times with 0.2 M HCl. The aqueous layer was basified until pH ≥ 14 with 1.0 M NaOH and extracted thrice with CH_2Cl_2. The combined organic layers were dried over Na_2SO_4, filtered, and cautiously concentrated by needle evaporation. The crude product **27** (38.4 mg, 90%) was received as a yellowish oil and used for the next step without further purification.

Amine **27** (21.5 mg, 151 µmol, 1.5 equiv) was added to a solution of **15** (50 mg, 0.1 mmol, 1.0 equiv) in CH_2Cl_2 (1 mL) at 0 °C. After stirring the resulting reaction solution at 0 °C overnight, the reaction mixture was allowed to warm to r.t. and stirred for another hour at r.t. The solvent was removed *in vacuo* and the crude product was purified by column chromatography (SiO_2, eluent: EtOAc–hexanes (1:1) to EtOAc–hexanes (2:1)) and preparative TLC (SiO_2, eluent: EtOAc–hexanes (2:1)) to afford azide **31** (41.6 mg, 82%) as a colorless foam.

^{1}H NMR (400 MHz, $CDCl_3$): δ = 7.21 (d, *J* = 10.1 Hz, 1H), 6.59 (t, *J* = 6.0 Hz, 1H), 6.32 (dd, *J* = 10.1, 1.9 Hz, 1H), 6.10 (t, *J* = 1.7 Hz, 1H), 4.40 – 4.27 (m, 1H), 3.35 (dt, *J* = 13.5, 6.8 Hz, 1H), 3.29 (s, 2H), 3.24 – 3.13 (m, 2H), 2.61 (tdd, *J* = 13.8, 6.1, 1.8 Hz, 1H), 2.45 – 2.24 (m, 4H), 2.20 – 2.08 (m, 1H), 1.91 (s, 1H), 1.88 – 1.71 (m, 2H), 1.67 – 1.48 (m, 8H), 1.47 – 1.32 (m, 5H), 1.29 – 1.17 (m, 1H), 1.12 (s, 3H), 0.93 (d, *J* = 7.3 Hz, 3H) ppm.

^{13}C NMR (101 MHz, $CDCl_3$): δ = 186.7, 172.5, 166.3 (d, *J* = 1.6 Hz), 152.3, 130.0, 125.3 (d, *J* = 1.4 Hz), 101.5, 99.8, 87.0, 72.4 (d, *J* = 38.9 Hz), 51.3, 48.5 (d, *J* = 22.7 Hz), 48.1, 44.0 (d, *J* = 1.6 Hz), 39.3, 36.7, 35.4, 34.5 (d, *J* = 19.5 Hz), 32.4, 31.2, 29.9, 28.7, 27.5 (d, *J* = 1.8 Hz), 26.5 (d, *J* = 10.4 Hz), 23.1 (d, *J* = 5.7 Hz), 17.5, 14.6 ppm.

^{19}F NMR (376 MHz, $CDCl_3$): δ = -165.56 ppm.

IR (neat): 3392, 2936, 2868, 2096, 1662, 1525, 1452, 1296, 1243, 1071, 1034, 1013, 893 cm^{-1}.

HRMS (ESI): *m/z* calcd. for $C_{27}H_{40}FN_4O_4$ $[M+H]^+$ 503.3028, found 503.3024.

Synthesis of 32

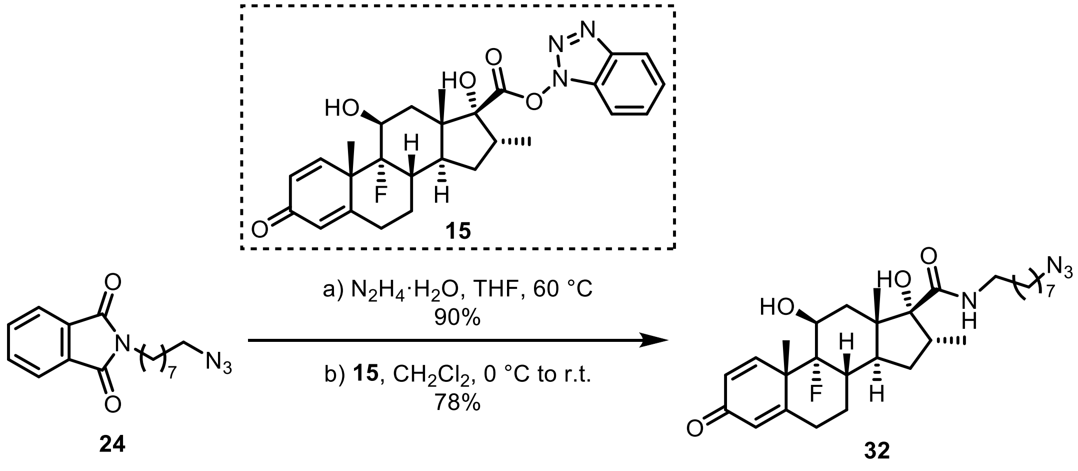

$N_2H_4 \cdot H_2O$ (210 mg, 204 µL, 2.10 mmol, 7.0 equiv) was added to a solution of **24** (90 mg, 0.30 µmol, 1.0 equiv) in THF (3 mL). After stirring the resulting reaction solution at 60 °C for 6 h, the reaction mixture was cooled down to r.t. and diluted with CH_2Cl_2. The organic layer was then washed three times with 0.2 M HCl. The aqueous layer was basified until pH ≥ 14 with 1.0 M NaOH and extracted thrice with CH_2Cl_2. The combined organic layers were dried over Na_2SO_4, filtered, and cautiously concentrated by needle evaporation. The crude product **28** (45.9 mg, 90%) was received as a yellowish oil and used for the next step without further purification.

Amine **28** (25.8 mg, 151 µmol, 1.50 equiv) was added to a solution of **15** (50 mg, 0.1 mmol, 1.0 equiv) in CH_2Cl_2 (1 mL) at 0 °C. After stirring the resulting reaction solution at 0 °C overnight, the reaction mixture was allowed to warm to r.t. and stirred for another hour at r.t. The solvent was removed *in vacuo* and the crude product was purified by column chromatography (SiO_2, eluent: EtOAc–hexanes (1:2) to EtOAc–hexanes (2:1)) and preparative TLC (SiO_2, eluent: EtOAc–hexanes (2:1)) to afford azide **32** (41.6 mg, 78%) as a colorless foam.

^{1}H NMR (400 MHz, $CDCl_3$): δ = 7.20 (d, *J* = 10.1 Hz, 1H), 6.55 (t, *J* = 5.8 Hz, 1H), 6.32 (dd, *J* = 10.1, 1.9 Hz, 1H), 6.11 (t, *J* = 1.7 Hz, 1H), 4.39 – 4.31 (m, 1H), 3.36 – 3.12 (m, 5H), 2.61 (tdd, *J* = 13.8, 6.1, 1.8 H, 1H), 2.35 (dddd, *J* = 25.0, 14.7, 8.2, 4.1 Hz, 3H), 2.23 – 2.10 (m, 2H), 1.90 – 1.71 (m, 2H), 1.73 – 1.44 (m, 8H), 1.43 – 1.28 (m, 9H), 1.28 – 1.19 (m, 1H), 1.12 (s, 3H), 0.94 (d, *J* = 7.3 Hz, 3H) ppm.

^{13}C NMR (101 MHz, $CDCl_3$): δ = 186.7, 172.4, 166.2, 152.1, 130.0, 125.3, 101.5, 99.7, 86.9, 72.4 (d, *J* = 38.9 Hz), 51.6, 48.4 (d, *J* = 22.8 Hz), 48.0, 44.0 (d, *J* = 1.6 Hz), 39.5, 36.8, 35.4, 34.5 (d, *J* = 19.6 Hz), 32.4, 31.2, 30.0, 29.2, 29.2, 29.0, 27.5, 26.8 (d, *J* = 19.1 Hz), 23.1 (d, *J* = 5.8 Hz), 17.6, 14.6 ppm.

^{19}F NMR (376 MHz, $CDCl_3$): δ = -165.56 ppm.

IR (neat): 3386, 2932, 2858, 2095, 1662, 1623 1526, 1452, 1297, 1243, 1072, 1035, 893 cm^{-1}.

HRMS (ESI): *m/z* calcd. for $C_{29}H_{43}FN_4O_4Na$ $[M+Na]^+$ 553.3161, found 553.3159.

Synthesis of 33

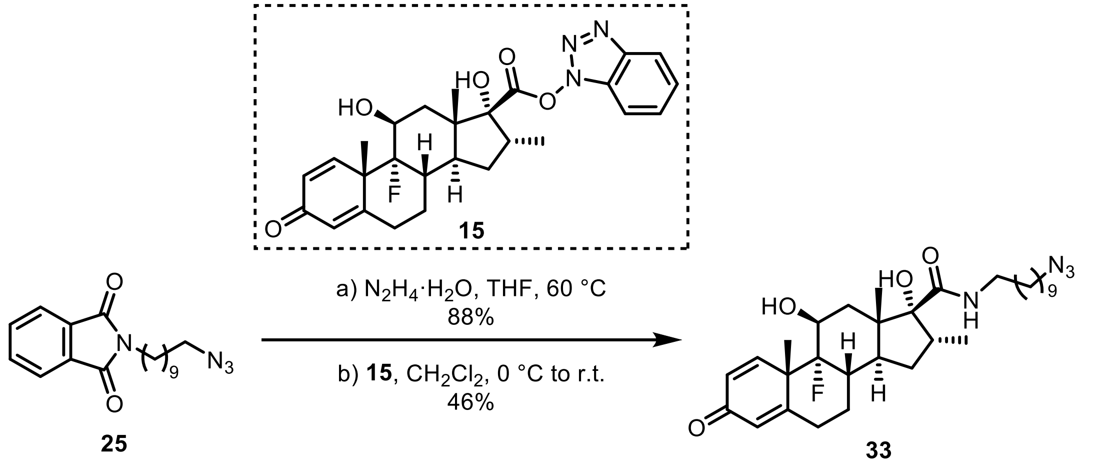

$N_2H_4{\cdot}H_2O$ (0.2 mL, 2.0 mmol, 6.6 equiv) was added to a solution of **25** (100 mg, 304 µmol, 1.0 equiv) in THF (3 mL). After stirring the resulting reaction solution at 60 °C for 6 h, the reaction mixture was cooled down to r.t. and diluted with CH_2Cl_2. The organic layer was then washed three times with 0.2 M HCl. The aqueous layer was basified until pH ≥ 14 with 1.0 M NaOH and extracted thrice with CH_2Cl_2. The combined organic layers were dried over Na_2SO_4, filtered, and cautiously concentrated by needle evaporation. The crude product **29** (53.4 mg, 88%) was received as a yellowish oil and used for the next step without further purification.

Amine **29** (30 mg, 0.15 mmol, 1.5 equiv) was added to a solution of **15** (50 mg, 0.10 µmol, 1.0 equiv) in CH_2Cl_2 (1 mL) at 0 °C. After stirring the resulting reaction solution at 0 °C overnight, the reaction mixture was allowed to warm to r.t. and stirred for another hour at r.t. The solvent was removed *in vacuo* and the crude product was purified by column chromatography (SiO_2, eluent: EtOAc–hexanes (1:1) to EtOAc–hexanes (2:1)) and preparative TLC (SiO_2, eluent: EtOAc–hexanes (2:1)) to afford azide **33** (25.9 mg, 46%) as a colorless foam.

^{1}H NMR (400 MHz, $CDCl_3$): δ = 7.23 (dd, *J* = 10.1, 1.8 Hz, 1H), 6.58 (q, *J* = 5.0, 4.6 Hz, 1H), 6.30 (dt, *J* = 10.2, 2.0 Hz, 1H), 6.09 (d, *J* = 2.0 Hz, 1H), 4.37 – 4.28 (m, 1H), 3.23 (dt, *J* = 13.1, 6.8 Hz, 5H), 2.60 (tdd, *J* = 13.7, 6.0, 1.8 Hz, 1H), 2.48 – 2.03 (m, 5H), 1.88 – 1.67 (m, 2H), 1.61 – 1.43 (m, 8H), 1.42 – 1.17 (m, 15H), 1.12 (s, 3H), 0.93 (d, *J* = 7.3 Hz, 3H) ppm.

^{13}C NMR (101 MHz, $CDCl_3$): δ = 186.7, 172.2, 166.4, 152.4, 129.8, 125.0, 101.5, 99.7, 86.8, 74.7 – 68.8 (m), 51.5, 48.4 (d, *J* = 22.8 Hz), 48.0, 43.8 (d, *J* = 1.6 Hz), 39.4, 36.5, 35.3, 34.4 (d, *J* = 19.5 Hz), 32.2, 31.1, 29.8, 29.4 (d, *J* = 3.3 Hz), 29.2, 29.1, 28.8, 27.4, 26.8 (d, *J* = 24.6 Hz), 22.9 (d, *J* = 5.7 Hz), 17.4, 14.5 ppm.

^{19}F NMR (376 MHz, $CDCl_3$): δ = -165.48 ppm.

IR (neat): 3391, 2930, 2856, 2095, 1662, 1623 1525, 1453, 1296, 1243, 1072, 1035, 892 cm^{-1}.

HRMS (ESI): *m/z* calcd. for $C_{31}H_{47}FN_4O_4Na$ $[M+Na]^+$ 581.3474, found 581.3465.

Synthesis of 35

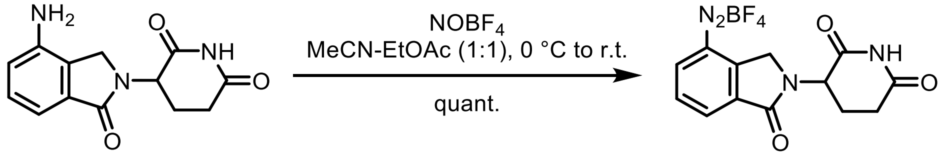

34 **35**

Lenalidomide (**34**, 3.0 g, 11 mmol, 1.0 equiv) was added as a solid to a solution of $NOBF_4$ (1.5 g, 13 mmol, 1.2 equiv) in MeCN (19.3 mL) and EtOAc (19.3 mL) at 0 °C. The reaction mixture was stirring for 30 min at 0 °C followed by 30 min at r.t. After cooling the reation mixture down to 0 °C , $NOBF_4$ (0.64 g, 5.5 mmol, 0.5 equiv) was added once more and the resulting mixture was stirred for another 30 min at 0 °C followed by 30 min at r.t. After addition of cold ether, the reaction mixture was filtered and washed with cold ether to afford **35** (3.9 g, quant.) without further purification as a pale red solid. **35** could be stored for several months (≥ 6 months) at –20 °C without decomposition.

^{1}H NMR (400 MHz, CD_3CN): δ = 8.93 (s, 1H), 8.68 (dd, *J* = 8.3, 1.0 Hz, 1H), 8.54 (ddd, *J* = 7.7, 1.0, 0.5 Hz, 1H), 8.06 (ddt, *J* = 8.4, 7.8, 0.7 Hz, 1H), 5.14 (dd, *J* = 13.4, 5.2 Hz, 1H), 4.97 – 4.85 (m, 2H), 2.91 – 2.70 (m, 3H), 2.50 – 2.37 (m, 1H), 2.19 (dtd, *J* = 12.9, 5.3, 2.6 Hz, 1H) ppm.

^{13}C NMR (101 MHz, CD_3CN): δ = 172.9, 170.8, 165.4, 146.6, 137.7, 137.5, 136.2, 133.0, 111.7, 53.4, 47.5, 32.0, 23.4 ppm.

^{19}F NMR (376 MHz, CD_3CN): δ = -151.57 ppm.

IR (neat): 3222, 3095, 2288, 1602, 1460, 1444, 1419, 1376, 1342, 1286, 1237, 1267, 1208, 1197, 1054, 935, 918, 750 cm^{-1}.

HRMS (ESI): *m/z* calcd. for $C_{23}H_{12}N_2O_3Na$ $[M+Na]^+$ 267.0740, found 267.0738.

Synthesis of 5

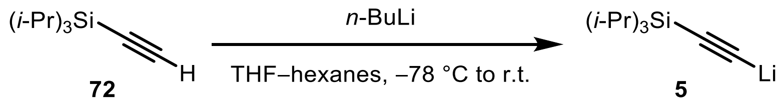

n-BuLi (1.6 M in hexane, 9.75 mL, 15.6 mmol, 1.0 equiv) was added to a solution of (*i*-Pr)$_3$Si-acetylene **72** (2.9 g, 3.5 mL, 0.17 mol, 1.0 equiv) in dry THF (6.2 mL) at –78 °C. After the addition, the mixture was allowed to warm to r.t. and additional THF (6.84 mL) was added to dilute the solution to a 0.6 M Li-(*i*-Pr)$_3$Si-acetylene solution in THF **5**. **5** could be stored for several weeks (≥ 1 month) at 0 °C without decomposition

Synthesis of 36

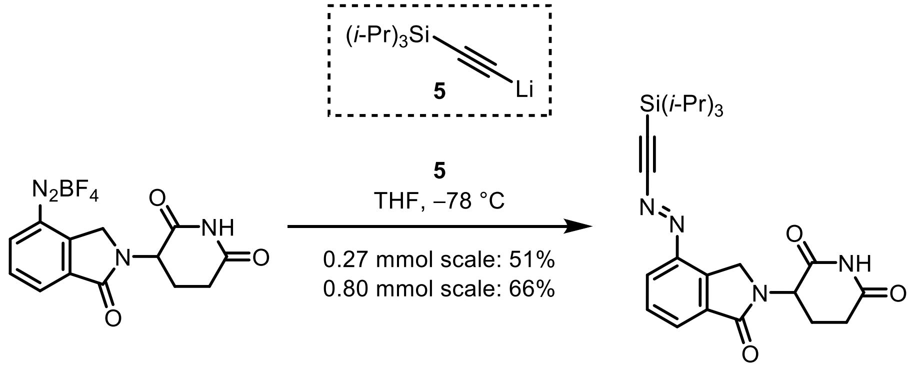

35 36

0.27 mmol scale
35 (96 mg, 0.27 mmol, 1.0 equiv) in dry THF (0.9 mL) was cooled down to -78 °C and stirred for 5 min at –78 °C. Li-(*i*-Pr)$_3$Si-acetylene **5** (0.6 M in THF-hexane, 0.45 mL, 0.27 mmol, 1.0 equiv) was added and stirred for 5 min at -78 °C. Residual **5** was quenched with EtOAc at -78 °C and the reaction solution was further diluted with EtOAc, washed with brine and dried over Na_2SO_4. Column chromatography (SiO_2, eluent: EtOAc–hexanes (1:4) to EtOAc–hexanes (1:2)) afforded **36** (62.3 mg, 51%) as a dark red solid.

0.80 mmol scale
35 (300 mg, 0.80 mmol, 1.0 equiv) in dry THF (2.8 mL) was cooled down to -78 °C and stirred for 10 min at –78 °C. Li-(*i*-Pr)$_3$Si-acetylene **5** (0.6 M in THF-hexane, 1.5 mL, 0.9 mmol, 1.1 equiv) was added and stirred for 45 min at -78 °C. After the addition of adiitional Li-(*i*-Pr)$_3$Si-acetylene **5** (0.6 M in THF-hexane, 0.87 mL, 0.52 mmol, 0.65 equiv), the resulting reaction solution was stirred for another 45 min at –78 °C. Residual **5** was quenched with EtOAc at -78 °C and the reaction solution was further diluted with EtOAc, washed with brine and dried over Na_2SO_4. Column chromatography (SiO_2, eluent: EtOAc–hexanes (1:4) to EtOAc–hexanes (1:2)) afforded **36** (249 mg, 66%) as a dark red solid.

^{1}H NMR (400 MHz, $CDCl_3$): δ = 8.23 – 8.16 (m, 2H), 8.05 (dd, *J* = 7.5, 1.1 Hz, 1H), 7.72 (t, *J* = 7.7 Hz, 1H), 5.24 (dd, *J* = 13.4, 5.1 Hz, 1H), 4.70 – 4.52 (m, 2H), 2.97 – 2.78 (m, 2H), 2.45 (qd, *J* = 13.1, 5.1 Hz, 1H), 2.22 (dtd, *J* = 13.0, 5.2, 2.6 Hz, 1H), 1.29 – 1.20 (m, 5H), 1.16 (d, *J* = 6.3 Hz, 16H) ppm.

^{13}C NMR (101 MHz, $CDCl_3$): δ = 171.3, 169.5, 168.2, 148.5, 133.6, 133.3, 131.3, 129.7, 128.0, 118.9, 111.0, 52.1, 48.5, 31.7, 23.5, 18.8, 11.5 ppm.

IR (neat): 3243, 3103, 2944, 2891, 2866, 1781, 1719, 1465, 1387, 1355, 1325, 1260, 1197, 1120, 1075, 1020, 996, 882, 789, 746, 677 cm^{-1}.

HRMS (ESI): *m/z* calcd. for $C_{24}H_{33}N_4O_3Si$ $[M+H]^+$ 453.2316, found 453.2310.

Synthesis of KH-5-210

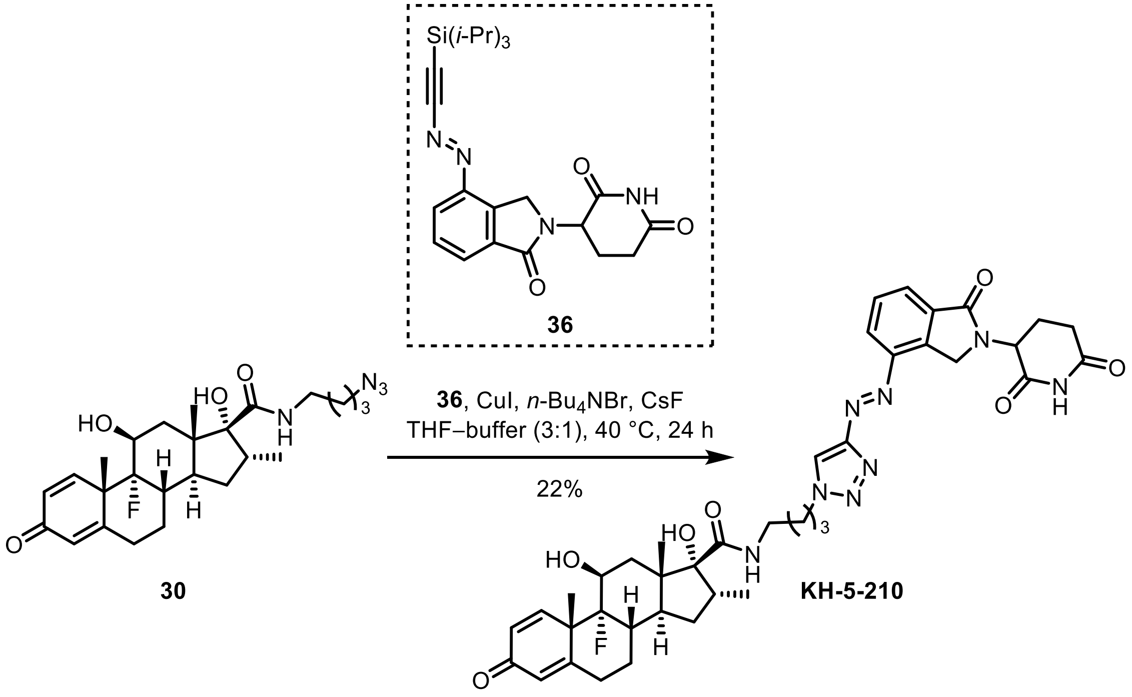

A vial was charged with azide **30** (20 mg, 42 µmol, 1.0 equiv), azoacetylene **36** (28.6 mg, 63.2 µmol, 1.5 equiv), n-Bu_4NBr (19 mg, 60 µmol, 1.4 equiv) and CuI (2.4 mg, 13 µmol, 0.30 equiv). THF (0.3 mL) and phosphate buffer (1.0 M, pH = 7.4, 60 µL) were added followed by CsF (1.5 M in water, 40 µL, 60 µmol, 1.4 equiv) at r.t. The reaction mixture was warmed to 40 °C and stirred at 40 °C for 24 h. After filtration trough a plug of silica usind EtOAc as eluent, the mixture was concentrated *in vacuo*. Column chromatography (SiO_2, eluent: EtOAc to acetone–EtOAc (1:2)) followed by preparative TLC (SiO_2, eluent: acetone–EtOAc (1:5)) and C_{18}-reversed phase column chromatography (MeCN–H_2O (1:1) to MeCN) afforded **KH-5-210** (7.10 mg, 22%) as an orange-red solid.

*Note: The synthetic procedure for **KH-5-210** was optimized (vide infra).*

Optimized Synthesis of KH-5-210

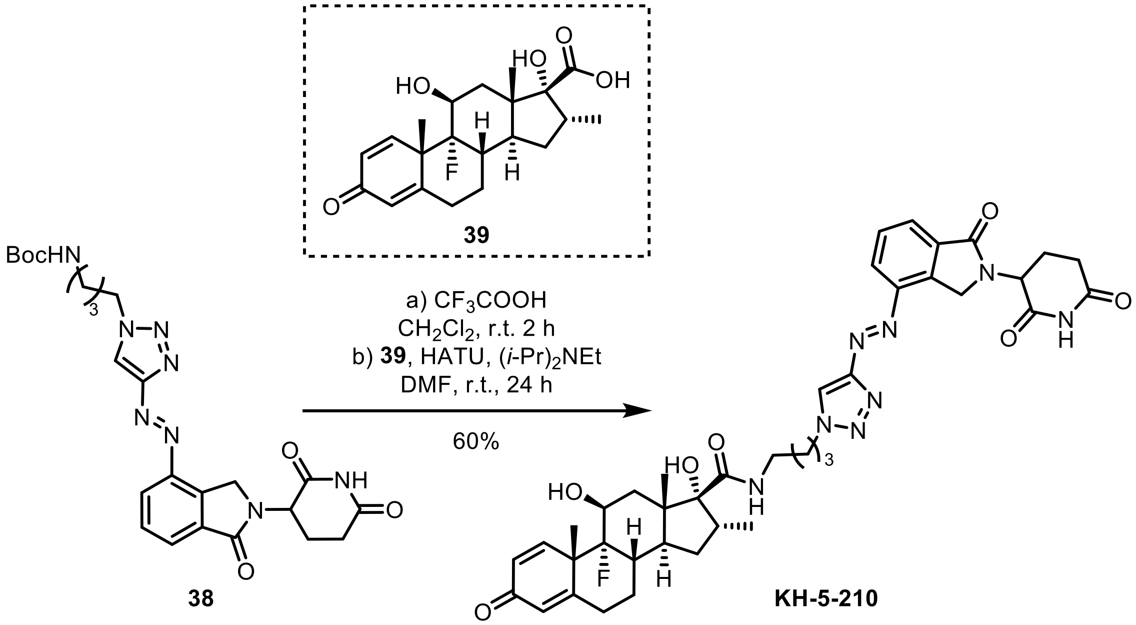

38 (41 mg, 0.80 mmol, 1.0 equiv) was dissolved in CH_2Cl_2 (0.4 mL) and CF_3COOH (0.4 mL, 5.2 mmol, 65 equiv) was added at r.t. After stirring the resulting reaction solution for 2 h at r.t., the reaction solution was siluted with EtOAc and toluene and all solvent was removed *in vacuo*. The crude product was used for the following step without further purification.

The crude product (32 mg, 0.78 mmol, 1.0 equiv), **39** (31 mg, 0.8 mmol, 1.05 equiv), HATU (34 mg, 0.78 mmol, 1.0 equiv) were dissolved in DMF (0.8 mL). (*i*-Pr)$_2$NEt (0.33 mL, 0.23 mmol, 3.0 equiv) was added and the resulting reaction mixture was stirred for 24 h at r.t. After concentrating the reaction mixture *in vacuo*., column chromatography (SiO_2, eluent: EtOAc to acetone–EtOAc (1:2)) followed by preparative TLC (SiO_2, eluent: EtOAc–acetone (4:1)) and C_{18}-reversed phase column chromatography (MeCN–H_2O (1:1) to MeCN) afforded **KH-5-210** (35.9 mg, 60%) as an orange-red solid.

^{1}H NMR (500 MHz, CD_3OD): δ = 9.82 (s, 1H), 8.49 (s, 1H), 8.20 (dt, *J* = 7.8, 1.2 Hz, 1H), 7.94 (dd, *J* = 7.5, 1.1 Hz, 1H), 7.86 – 7.78 (m, 1H), 7.28 (dd, *J* = 10.2, 2.3 Hz, 1H), 7.23 (t, *J* = 6.1 Hz, 1H), 6.17 (ddd, *J* = 10.1, 1.9, 1.4 Hz, 1H), 5.99 (s, 1H), 5.29 – 5.22 (m, 1H), 4.88 – 4.74 (m, 2H), 4.59 (td, *J* = 6.9, 1.6 Hz, 2H), 4.32 (ddt, *J* = 9.6, 5.9, 3.4 Hz, 2H), 3.37 (dtd, *J* = 12.9, 6.5, 3.5 Hz, 1H), 3.26 (ddt, *J* = 13.1, 11.0, 6.5 Hz, 1H), 3.17 (dt, *J* = 9.4, 3.6 Hz, 1H), 3.08 – 2.97 (m, 1H), 2.79 (s, 1H), 2.68 (dtd, *J* = 27.3, 13.6, 5.3 Hz, 2H), 2.46 (dtd, *J* = 29.2, 11.9, 4.8 Hz, 1H), 2.35 (dd, *J* = 13.9, 5.0 Hz, 1H), 2.30 – 2.23 (m, 1H), 2.22 – 2.15 (m, 2H), 2.07 (dq, *J* = 3.2, 1.0 Hz, 1H), 2.02 (d, *J* = 6.7 Hz, 2H), 1.90 – 1.82 (m, 1H), 1.79 – 1.70 (m, 1H), 1.59 (d, *J* = 3.5 Hz, 3H), 1.59 – 1.44 (m, 4H), 1.22 – 1.15 (m, 1H), 1.10 (dd, *J* = 1.6, 0.7 Hz, 3H), 0.89 (d, *J* = 7.3 Hz, 3H) ppm.

^{13}C NMR (126 MHz, CD_3OD): δ = 186.1, 173.5, 172.7, 171.3, 168.1, 167.1, 161.5, 152. 9, 148.2, 135.4 (d, *J* = 4.6 Hz), 135.2, 130.3 (d, *J* = 25.0 Hz), 130.0 (d, *J* = 9.6 Hz), 126. 8, 125.4, 116.4 (d, *J* = 3.8 Hz), 102.7, 101.3, 87.7 (d, *J* = 1.4 Hz), 72.8 (d, *J* = 38.1 Hz), 53.0, 51.0, 49.3 – 48.8 (m),

44.6, 38.8, 36.7, 35.9, 35.2 (d, *J* = 19.4 Hz), 33.1, 32.2, 31.6, 30.7 (d, *J* = 18.7 Hz), 28.4, 28.3, 27.5 (d, *J* = 2.2 Hz), 24.0, 23.7 (d, *J* = 5.8 Hz), 17.8, 15.2 ppm.

^{19}F NMR (471 MHz, CD_3OD): δ = -165.64 (d, *J* = 7.4 Hz) ppm.

IR (neat): 3415, 2927, 2855, 1701, 1663, 1522, 1452, 1365, 1298, 1267, 1235, 1036, 894 cm^{-1}.

HRMS (ESI): *m/z* calcd. for $C_{40}H_{47}FN_8O_7$ $[M+H]^+$ 771.3625, found 771.3626.

Synthesis of 37

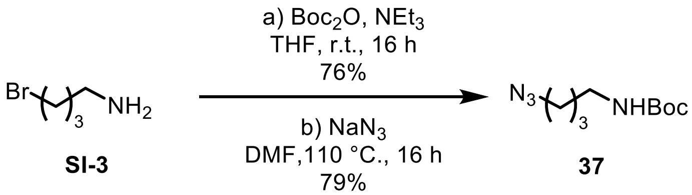

Boc_2O (0.94 mL, 4.0 mmol, 0.95 equiv) followed by NEt_3 (1.7 mL, 8.6 mmol, 2.0 equiv) was added to a solution of **SI-3** (1.0 g, 4.3 mmol, 1.0 equiv) in THF (19 mL). After stirring the resulting reaction solution at r.t. overnight, the reaction mixture was diluted with water. The aqueous layer was extracted thrice with EtOAc and the combined organic layers were washed with HCl (aq. 0.5 M) and an aq. sat. solution of sodium bicarbonate followed by brine, dried over Na_2SO_4, filtered and concentrated cautiously by needle evaporation to afford the crude product as a colorless oil (823 mg, 76%).

NaN_3 (414 mg, 6.37 mmol, 3.00 equiv) was added to a solution of the crude product (535 mg, 2.12 mmol, 1.00 equiv) DMF (3.9 mL). After stirring the resulting reaction solution at 110 °C overnight, the reaction mixture was allowed to cool down to ambient temperature and diluted with water. The aqueous layer was extracted thrice with CH_2Cl_2 and the combined organic layers were washed with an aq. sat. solution of LiCl (5%), brine, dried over Na_2SO_4, filtered and concentrated cautiously by needle evaporation to afford the crude product **37** as a colorless oil (360 mg, 79% (60% over two steps)).

^{1}H NMR (400 MHz, $CDCl_3$): δ = 4.54 (s, 1H), 3.30 (t, *J* = 6.5 Hz, 2H), 3.15 (q, *J* = 6.2 Hz, 2H), 1.68 – 1.51 (m, 4H), 1.44 (s, 9H) ppm.

^{13}C NMR (101 MHz, $CDCl_3$): δ = 156.1, 79.4, 51.3, 40.1, 28.6, 27.6, 26.3 ppm.

IR (neat): 3354, 2977, 2935, 2870, 2094, 1689, 1517, 1454, 1392, 1366, 1250, 1169, 1040, 1010 cm^{-1}.

HRMS (ESI): *m/z* calcd. for $C_9H_{18}N_4NaO_2$ $[M+Na]^+$ 237.1322, found 237.1322.

Synthesis of 38

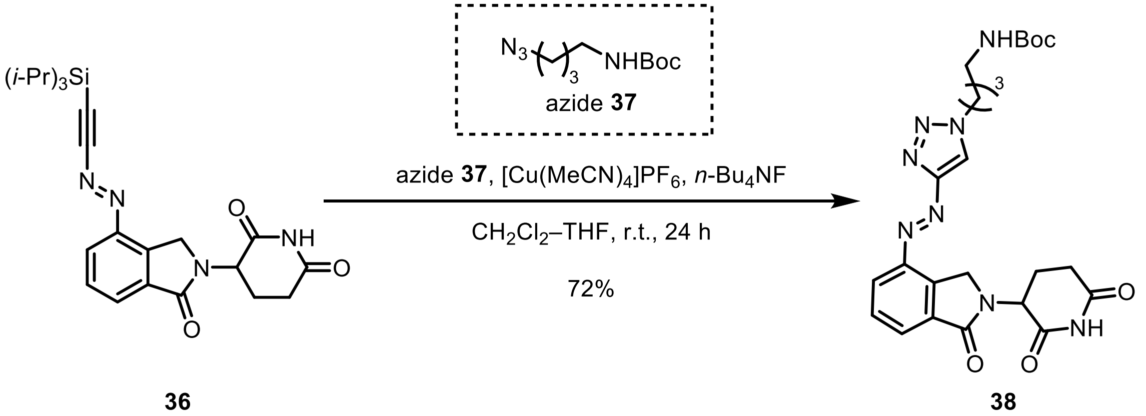

A vial was charged with azoacetylene **36** (100 mg, 221 μmol, 1.0 equiv), azide **37** (47 mg, 0.22 mmol, 1.0 equiv), $[Cu(MeCN)_4]PF_6$ (83 mg, 0.22 μmol, 1.0 equiv) was subsequently added in the glovebox and the vial was taken out of the glovebox. CH_2Cl_2 (2.2 mL) was degassed for 15 min using argon and was added to the vial followed by *n*-Bu_4NF (1.0 M in THF, 0.22 mL, 0.22 mmol, 1.0 equiv). After stirring the reaction mixture for 24 h at r.t., the solvent was removed and column chromatography (SiO_2, eluent: EtOAc–hexanes (3:1) to EtOAc followed by 15% acetone in EtOAc) afforded **38** (80.7 mg, 72%) as a deep dark yellow solid.

^{1}H NMR (500 MHz, $(CD_3)_2CO$): δ = 9.81 (s, 1H), 8.47 (d, *J* = 1.5 Hz, 1H), 8.21 – 8.13 (m, 1H), 7.93 (dq, *J* = 7.5, 1.1 Hz, 1H), 7.80 (td, *J* = 7.7, 2.2 Hz, 1H), 6.04 (s, 1H), 5.26 (dd, *J* = 13.4, 5.2 Hz, 1H), 4.87 – 4.74 (m, 2H), 4.57 (t, *J* = 7.1 Hz, 2H), 3.15 (q, *J* = 6.5 Hz, 2H), 3.02 (ddd, *J* = 17.6, 13.7, 5.4 Hz, 1H), 2.66 (td, *J* = 13.4, 4.5 Hz, 1H), 2.26 (dtd, *J* = 12.9, 5.3, 2.4 Hz, 1H), 2.09 (s, 2H), 1.55 (p, *J* = 6.9 Hz, 2H), 1.39 (s, 9H) ppm.

^{13}C NMR (126 MHz, $(CD_3)_2CO$): δ = 172.7, 171.3, 168.2, 161.6, 156.8, 148.2, 135.4, 135.2, 130.3, 130.0, 126.7, 116.0, 78.5, 55.5, 53.0, 51.0, 49.1, 40.2, 32.2, 30.6, 28.7, 28.2, 27.7, 23.9 ppm.

IR (neat): 3357, 3221, 3109, 2976, 2932, 2871, 1692, 1521, 1452, 1429, 1272, 1234, 1202, 1172, 1146, 1039, 846, 753 cm^{-1}.

HRMS (ESI): *m/z* calcd. for $C_{24}H_{30}N_8O_5Na$ $[M+Na]^+$ 533.2231, found 533.2223.

Synthesis of KH-5-224

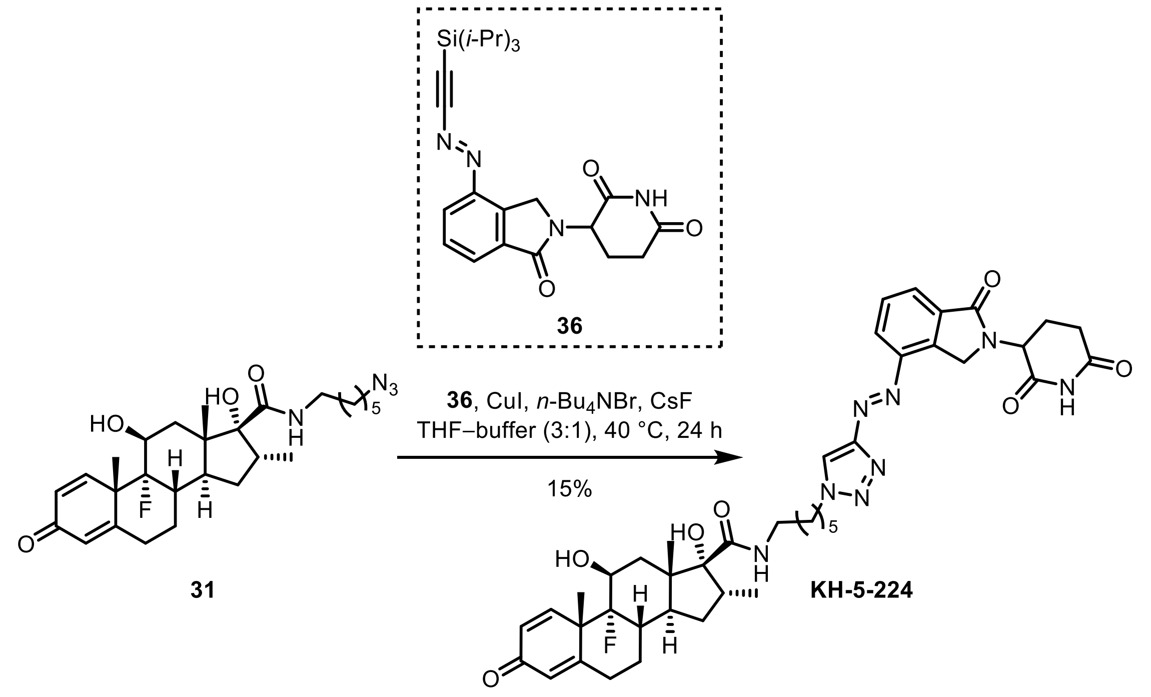

A vial was charged with azide **31** (16.5 mg, 32.8 µmol, 1.0 equiv), azoacetylene **36** (22.3 mg, 49.2 µmol, 1.5 equiv), *n*-Bu_4NBr (15 mg, 47 µmol, 1.4 equiv) and CuI (1.9 mg, 9.9 µmol, 0.3 equiv). THF (0.25 mL) and phosphate buffer (1.0 M, pH = 7.4, 50 µL) were added followed by CsF (1.5 M in water, 31 µL, 47 µmol, 1.4 equiv) at r.t. The reaction mixture was warmed to 60 °C and stirred at 60 °C for 24 h. After filtration trough a plug of silica usind EtOAc as eluent, the mixture was concentrated *in vacuo*. Column chromatography (SiO_2, eluent: EtOAc to acetone–EtOAc (1:2)) followed by preparative TLC (SiO_2, eluent: acetone–EtOAc (1:5)) and C_{18}-reversed phase column chromatography (MeCN–H_2O (1:1) to MeCN) afforded **KH-5-224** (3.9 mg, 15%) as an orange-red solid.

^{1}H NMR (500 MHz, $(CD_3)_2CO$): δ = 9.82 (s, 1H), 8.49 (s, 1H), 8.19 (dd, *J* = 7.8, 1.0 Hz, 1H), 7.94 (dd, *J* = 7.5, 1.0 Hz, 1H), 7.80 (t, *J* = 7.7 Hz, 1H), 7.30 (d, *J* = 10.3 Hz, 1H), 7.07 (q, *J* = 7.7, 6.1 Hz, 1H), 6.18 (ddd, *J* = 10.1, 1.9, 1.1 Hz, 1H), 5.99 (t, *J* = 1.8 Hz, 1H), 5.26 (dd, *J* = 13.3, 5.2 Hz, 1H), 4.89 – 4.74 (m, 2H), 4.55 (t, *J* = 7.0 Hz, 2H), 4.34 (d, *J* = 22.7 Hz, 2H), 3.27 (dq, *J* = 13.3, 6.7 Hz, 1H), 3.15 (dq, *J* = 13.4, 6.7 Hz, 2H), 3.02 (ddd, *J* = 17.5, 13.7, 5.4 Hz, 1H), 2.79 (d, *J* = 5.2 Hz, 1H), 2.75 – 2.58 (m, 2H), 2.46 (dtd, *J* = 29.2, 12.0, 4.9 Hz, 1H), 2.39 – 2.32 (m, 1H), 2.26 (ddq, *J* = 10.6, 5.3, 2.7 Hz, 1H), 2.23 – 2.14 (m, 2H), 2.08 – 2.06 (m, 1H), 2.04 – 1.99 (m, 2H), 1.90 – 1.82 (m, 1H), 1.73 (q, *J* = 11.7 Hz, 1H), 1.60 (d, *J* = 1.0 Hz, 3H), 1.56 – 1.44 (m, 4H), 1.44 – 1.36 (m, 4H), 1.22 – 1.15 (m, 1H), 1.11 (s, 3H), 0.89 (d, *J* = 7.3 Hz, 3H) ppm.

^{13}C NMR (126 MHz, $(CD_3)_2CO$): δ = 186.1, 173.2, 172.7, 171.3, 168.1, 167.1, 161.6, 152.9, 148.2, 135.4, 135.2, 130.3 (d, *J* = 23.4 Hz), 130.1, 126.7, 125.4 (d, *J* = 1.3 Hz), 116.1, 102.7, 101.3, 87.6, 72.7 (d, *J* = 37.9 Hz), 53.0, 51.4, 49.3 – 48.7 (m), 44.5, 39.6, 36.8, 35.9, 35.3 (d, *J* = 19.5 Hz), 33.1, 32.2, 31.6, 30.6 (d, *J* = 36.1 Hz), 28.4 (d, *J* = 1.8 Hz), 26.9 (d, *J* = 1.6 Hz), 26.7, 23.9, 23.7 (d, *J* = 5.8 Hz), 17.8, 15.2 ppm.

^{19}F NMR (471 MHz, $(CD_3)_2CO$): δ = -165.50 (d, *J* = 4.2 Hz) ppm.

IR (neat): 3417, 3109, 2929, 2859, 1701, 1663, 1522, 1452, 1365, 1234, 1203, 1035, 893 cm^{-1}.

HRMS (ESI): *m/z* calcd. for $C_{42}H_{51}FN_8O_7$ $[M+H]^+$ 799.3938, found 799.3935.

Synthesis of KH-5-225

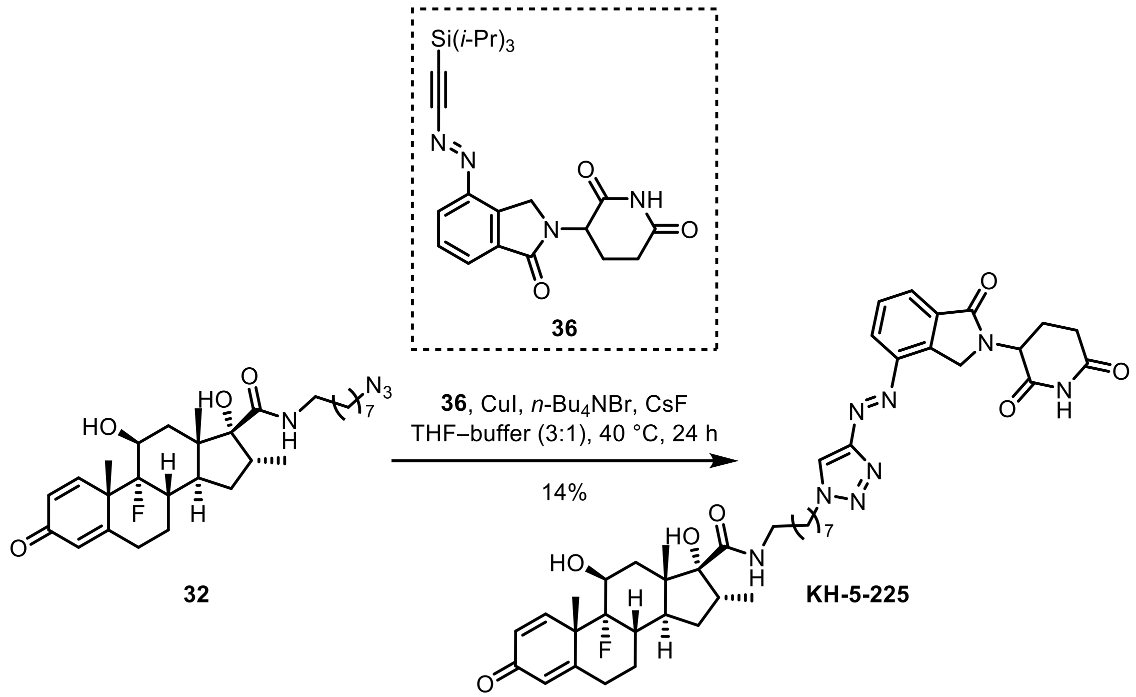

A vial was charged with azide **32** (21.9 mg, 41.3 µmol, 1.0 equiv), azoacetylene **36** (28 mg, 62 µmol, 1.5 equiv), *n*-Bu_4NBr (19 mg, 59 µmol, 1.4 equiv) and CuI (2.5 mg, 13 µmol, 0.3 equiv). THF (0.3 mL) and phosphate buffer (1.0 M, pH = 7.4, 40 µL) were added followed by CsF (1.5 M in water, 39 µL, 59 µmol, 1.4 equiv) at r.t. The reaction mixture was warmed to 60 °C and stirred at 60 °C for 24 h. After filtration trough a plug of silica usind EtOAc as eluent, the mixture was concentrated *in vacuo*. Column chromatography (SiO_2, eluent: EtOAc to acetone–EtOAc (1:3)) followed by preparative TLC (SiO_2, eluent: acetone–EtOAc (1:5)) and C_{18}-reversed phase column chromatography (MeCN-H_2O (1:1) to MeCN) afforded **KH-5-225** (4.9 mg, 14%) as an orange-red solid.

^{1}H NMR (500 MHz, $(CD_3)_2CO$): δ = 9.82 (s, 1H), 8.49 (d, *J* = 1.8 Hz, 1H), 8.19 (dd, *J* = 7.8, 1.1 Hz, 1H), 7.94 (dd, *J* = 7.5, 1.1 Hz, 1H), 7.80 (t, *J* = 7.7 Hz, 1H), 7.31 (d, *J* = 10.2 Hz, 1H), 7.04 (dd, *J* = 11.8, 5.8 Hz, 1H), 6.17 (ddd, *J* = 10.2, 1.9, 1.2 Hz, 1H), 5.99 (t, *J* = 1.8 Hz, 1H), 5.26 (dd, *J* = 13.4, 5.2 Hz, 1H), 4.88 – 4.75 (m, 2H), 4.55 (t, *J* = 7.1 Hz, 2H), 4.42 – 4.30 (m, 2H), 3.25 (dq, *J* = 13.5, 6.7 Hz, 1H), 3.20 – 3.10 (m, 2H), 3.02 (ddd, *J* = 17.6, 13.7, 5.4 Hz, 1H), 2.80 – 2.77 (m, 1H), 2.75 – 2.61 (m, 2H), 2.53 – 2.40 (m, 1H), 2.40 – 2.32 (m, 1H), 2.25 (dtd, *J* = 13.1, 5.5, 2.5 Hz, 1H), 2.22 – 2.16 (m, 2H), 2.08 – 2.06 (m, 1H), 2.01 (t, *J* = 7.0 Hz, 2H), 1.89 – 1.82 (m, 1H), 1.78 – 1.68 (m, 1H), 1.60 (s, 3H), 1.55 – 1.44 (m, 4H), 1.35 (dd, *J* = 23.0, 4.5 Hz, 8H), 1.20 – 1.15 (m, 2H), 1.11 (s, 3H), 0.89 (d, *J* = 7.3 Hz, 3H) ppm.

^{13}C NMR (126 MHz, $(CD_3)_2CO$): δ = 186.1, 173.1, 172.7, 171.3, 168.1, 167.1, 161.6, 152.9, 148.2, 135.4, 135.2, 130.3 (d, *J* = 24.0 Hz), 130.1, 126.7, 125.4, 116.1, 102.7, 101.3, 87.6, 72.7 (d, *J* = 37.6 Hz), 53.0, 51.4, 49.4 – 48.7 (m), 44.5, 39.8, 36.8, 35.9, 35.2 (d, *J* = 19.4 Hz), 33.1, 32.2, 31.6, 30.7, 30.6, 28.4 (d, *J* = 1.9 Hz), 27.5, 26.9, 23.9, 23.7 (d, *J* = 5.9 Hz), 17.7, 15.2 ppm.

^{19}F NMR (471 MHz, $(CD_3)_2CO$): δ = -165.42 ppm.

IR (neat): 3406, 3120, 2927, 2856, 1702, 1663, 1523, 1452, 1363, 1233, 1203, 1081, 1035, 977 cm^{-1}.

HRMS (ESI): *m/z* calcd. for $C_{44}H_{56}FN_8O_7$ $[M+H]^+$ 827.4251, found 827.4246.

Synthesis of KH-5-226

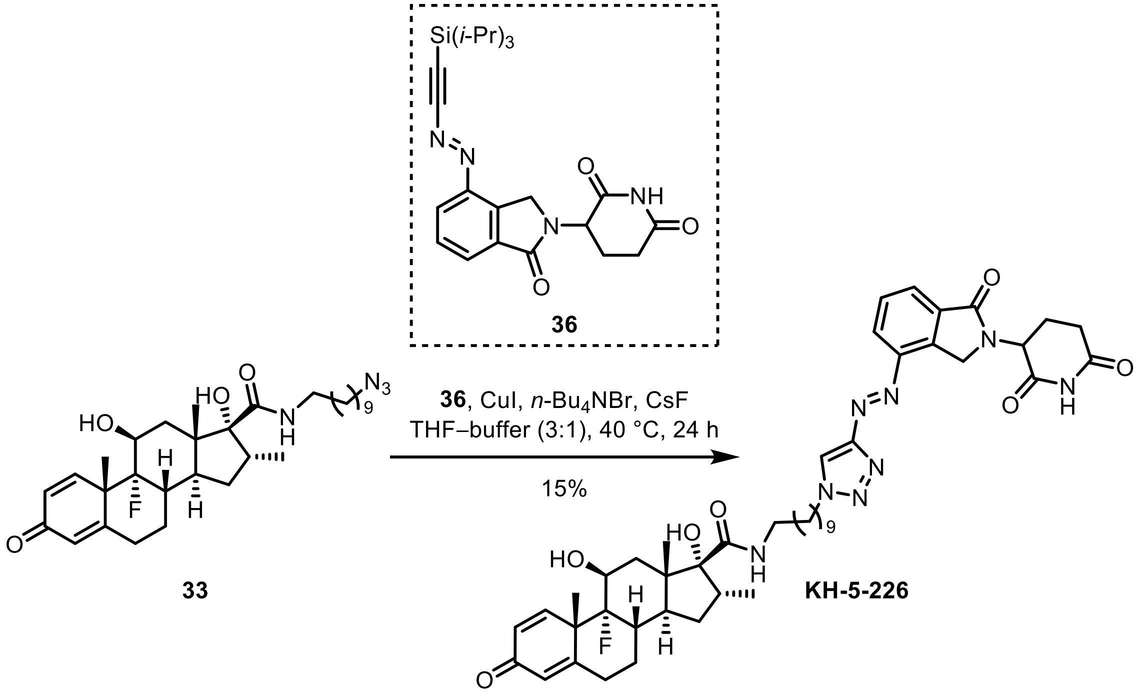

A vial was charged with azide **33** (16.9 mg, 30.2 µmol, 1.00 equiv), azoacetylene **36** (20.5 mg, 45.4 µmol, 1.50 equiv), *n*-Bu_4NBr (14.2 mg, 43.9 µmol, 1.45 equiv) and CuI (1.73 mg, 9.07 µmol, 0.300 equiv). THF (0.23 mL) and phosphate buffer (1.0 M, pH = 7.4, 35 µL) were added followed by CsF (1.5 M in water, 29.3 µL, 43.9 µmol, 1.45 equiv) at r.t. The reaction mixture was warmed to 40 °C and stirred at 60 °C for 24 h. After filtration trough a plug of silica usind EtOAc as eluent, the mixture was concentrated *in vacuo*. Column chromatography (SiO_2, eluent: EtOAc to acetone–EtOAc (1:2)) followed by preparative TLC (SiO_2, eluent: acetone–EtOAc (1:5)) and C_{18}-reversed phase column chromatography (MeCN–H_2O (1:1) to MeCN) afforded **KH-5-226** (3.3 mg, 13%) as an orange-red solid.

^{1}H NMR (500 MHz, $(CD_3)_2CO$): δ = 9.82 (s, 1H), 8.48 (d, *J* = 1.8 Hz, 1H), 8.19 (dd, *J* = 7.8, 0.9 Hz, 1H), 7.94 (dd, *J* = 7.5, 1.0 Hz, 1H), 7.80 (t, *J* = 7.7 Hz, 1H), 7.30 (dd, *J* = 10.1, 2.2 Hz, 1H), 7.03 (d, *J* = 9.1 Hz, 1H), 6.17 (dt, *J* = 10.2, 1.6 Hz, 1H), 5.99 (t, *J* = 1.8 Hz, 1H), 5.26 (dd, *J* = 13.4, 5.2 Hz, 1H), 4.87 – 4.75 (m, 2H), 4.54 (t, *J* = 7.0 Hz, 2H), 4.38 – 4.28 (m, 2H), 3.23 (tt, *J* = 12.6, 6.3 Hz, 1H), 3.15 (ddd, *J* = 10.9, 8.5, 5.0 Hz, 2H), 3.02 (ddd, *J* = 17.6, 13.7, 5.4 Hz, 1H), 2.78 (dd, *J* = 4.4, 2.2 Hz, 1H), 2.75 – 2.61 (m, 2H), 2.46 (dtd, *J* = 29.3, 11.9, 4.9 Hz, 1H), 2.35 (dd, *J* = 13.8, 5.0 Hz, 1H), 2.30 – 2.22 (m, 1H), 2.22 – 2.15 (m, 2H), 2.07 (dd, *J* = 2.2, 0.9 Hz, 1H), 2.01 (t, *J* = 7.1 Hz, 2H), 1.86 (d, *J* = 12.1 Hz, 1H), 1.72 (q, *J* = 11.8 Hz, 1H), 1.60 (s, 3H), 1.55 – 1.44 (m, 4H), 1.39 – 1.29 (m, 12H), 1.17 (dt, *J* = 11.0, 3.8 Hz, 1H), 1.11 (s, 3H), 0.89 (dd, *J* = 7.3, 1.6 Hz, 3H) ppm.

^{13}C NMR (126 MHz, $(CD_3)_2CO$): δ = 186.1, 173.1, 172.7, 171.3, 168.2, 167.1 (d, *J* = 1.6 Hz), 161.7, 152.9, 148.2, 135.4 (d, *J* = 4.0 Hz), 135.2, 130.3 (d, *J* = 27.3 Hz), 130.0 (d, *J* = 8.7 Hz), 126.7 (d, *J* = 1.6 Hz), 125.4, 116.0 (d, *J* = 2.3 Hz), 102.6 (d, *J* = 3.6 Hz), 101.5 (d, *J* = 3.7 Hz), 87.6, 72.7 (dd, *J* = 37.9, 2.7 Hz), 53.0 (d, *J* = 2.0 Hz), 51.5, 49.3 – 48.7 (m), 44.5 (d, *J* = 1.6 Hz), 39.8, 36.8, 35.9, 35.3 (d, *J* = 19.4 Hz), 33.1, 32.2, 31.6, 30.8 – 30.6 (m), 28.4 (d, *J* = 1.9 Hz), 27.6 (d, *J* = 2.1 Hz), 27.0 (d, *J* = 3.2 Hz), 23.9, 23.7 (d, *J* = 5.7 Hz), 17.7, 15.2 ppm.

^{19}F NMR (471 MHz, $(CD_3)_2CO$): δ = -165.40 (d, *J* = 2.7 Hz) ppm.

IR (neat): 3406, 3109, 2928, 2855, 1701, 1663, 1524, 1453, 1373, 1267, 1234, 1203, 1144, 1036, 893 cm^{-1}.

HRMS (ESI): *m/z* calcd. for $C_{46}H_{60}FN_8O_7$ $[M+H]^+$ 855.4564, found 855.4555.

GR Me_2-Arylazopyrazole photoPROTACs

Synthesis of 42

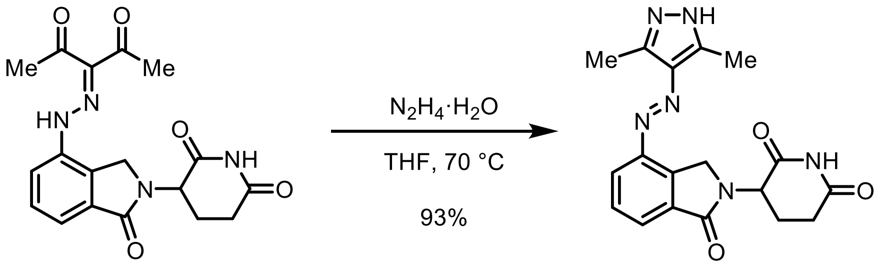

41 **42**

$N_2H_4{\cdot}H_2O$ (7.0 µL, 0.14 µmol, 1.05 equiv) was added to a suspension of **41** (500 mg, 1.35 mmol, 1.0 equiv) in anhydrous THF (13 mL) and the reaction mixture was warmed to 70 °C. After stirring the mixture for 45 min, the reaction mixture was concentrated in *vacuo* and purified by column chromatography (SiO_2, eluent: EtOAc to acetone–EtOAc (1:1)) to afford **42** (461 mg, 93%) as a yellow solid.

^{1}H NMR (600 MHz, $(CD_3)_2SO$): δ = ^{1}H NMR (600 MHz, DMSO-d_6) δ 12.94 (s, 1H), 10.99 (s, 1H), 8.00 (dd, *J* = 7.8, 1.1 Hz, 1H), 7.78 (dd, *J* = 7.5, 1.0 Hz, 1H), 7.69 (t, *J* = 7.6 Hz, 1H), 5.14 (dd, *J* = 13.3, 5.1 Hz, 1H), 4.75 – 4.56 (m, 2H), 2.92 (ddd, *J* = 17.4, 13.6, 5.4 Hz, 1H), 2.66 – 2.59 (m, 1H), 2.52 (s, 3H), 2.48 – 2.44 (m, 1H), 2.41 (s, 3H), 2.10 – 2.03 (m, 1H) ppm.

^{13}C NMR (151 MHz, $(CD_3)_2SO$): δ = 172.9, 171.0, 167.5, 147.5, 142.9, 139.23, 134.9, 134.2, 133.6, 129.3, 125.5, 123.5, 51.8, 48.0, 31.3, 22.4, 13.9, 10.2 ppm.

IR (neat): 3504, 3363, 3271, 3021, 2829, 1725, 1680, 1580, 1505, 1438, 1418, 1407, 1382, 1359, 1338, 1245, 1212, 1111, 978, 908, 752 cm^{-1}.

HRMS (ESI): *m/z* calcd. for $C_{18}H_{19}N_6O_3$ $[M+H]^+$ 367.1513, found 367.1504.

Synthesis of 41

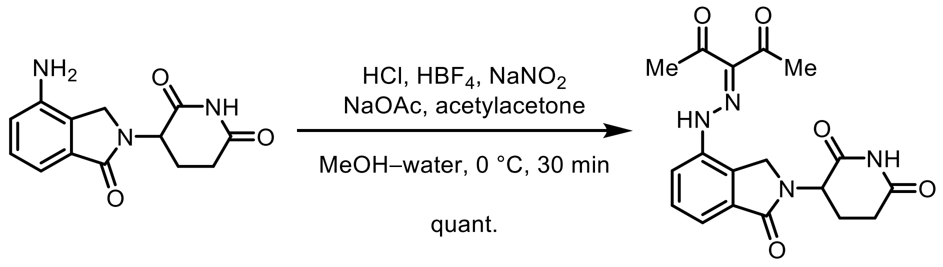

34 **41**

Lenalidomide **34** (1.0 g, 3.9 mmol, 1.0 equiv) was dissolved in HCl (1 M, 100 mL) and HBF_4 (4 mL). $NaNO_2$ (2 M in water, 2.1 mL, 4.2 mmol, 1.1 equiv) was added and the mixture was stirred for 1 h at 0 °C. Simultaneously, a saturated solution of NaOAc (ca. 50 g) in water (100 mL) was prepared, MeOH (40 mL) and acetylacetone (0.49 mL, 4.7 mmol, 1.2 equiv) were added and the resulting suspension was cooled to 0 °C. The diazonium salt mixture was then added and the resulting mixture was stirred for 30 min at 0 °C during which a solid precipitated from the reaction mixture. The solid was filtered off, washed with water and ether and dried in *vacuo*. The solid was then resuspended in acetone, filtered, washed with acetone until all of the brownish impurity was filtered off and dried in *vacuo* to afford **41** (1.42 g, quant.) as a yellowish solid which was used in the next step without further purification.

^{1}H NMR (400 MHz, $(CD_3)_2SO$): δ = 11.10 (s, 1H), 7.84 (d, *J* = 7.3 Hz, 1H), 7.67 (q, *J* = 7.6 Hz, 2H), 5.24 (dd, *J* = 13.3, 5.1 Hz, 1H), 4.97 – 4.64 (m, 2H), 3.42 (s, 1H), 3.27 (d, *J* = 5.2 Hz, 1H), 3.02 (ddd, *J* = 18.2, 13.5, 5.4 Hz, 1H), 2.72 (d, *J* = 17.9 Hz, 1H), 2.60 (s, 6H), 2.16 (dt, *J* = 11.9, 5.3 Hz, 1H) ppm.

^{13}C NMR (101 MHz, $(CD_3)_2SO$): δ = 197.4, 196.3, 172.9, 170.9, 167.3, 137.1 134.8, 133.9, 129.5, 129.0, 112.0, 119.4, 51.8, 47.8, 31.3, 27.0, 22.5 ppm.

IR (neat): 3323, 2926, 1711, 1685, 1669, 1508, 1416, 1354, 1310, 1267, 1231, 1204, 1175, 1090, 1021, 986, 947, 923, 820, 793, 750 cm^{-1}.

HRMS (ESI): *m/z* calcd. for $C_{18}H_{19}N_4O_5$ $[M+H]^+$ 371.1350, found 371.1354.

Synthesis of Me_2-arylazopyrazole photoswitch

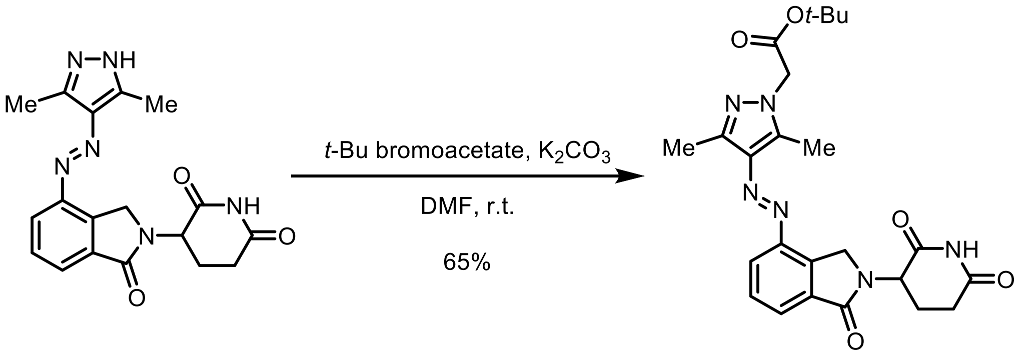

42

Me_2-arylazopyrazole photoswitch

t-Bu bromoacetate (44 µL, 0.30 mmol, 1.1 equiv) was added to a suspension of **42** (100 mg, 273 µmol, 1.0 equiv) in anhydrous DMF (2.7 mL) and the reaction mixture was stirred at r.t. overnight. The reaction mixture was concentrated in *vacuo* and purified by column chromatography (SiO_2, eluent: hexane–EtOAc (1:1) to acetone–EtOAc (1:1)) to afford **Me_2-arylazopyrazole photoswitch** (84.7 mg, 65%) as a yellow solid.

^{1}H NMR (500 MHz, $(CD_3)_2CO$): δ = 9.78 (s, 1H), 8.06 (dd, *J* = 7.8, 1.1 Hz, 1H), 7.80 (dd, *J* = 7.4, 1.1 Hz, 1H), 7.73 – 7.67 (m, 1H), 5.23 (dd, *J* = 13.3, 5.2 Hz, 1H), 4.90 (s, 2H), 4.81 (q, *J* = 18.1 Hz, 2H), 3.00 (ddd, *J* = 17.5, 13.6, 5.4 Hz, 1H), 2.82 – 2.76 (m, 1H), 2.69 – 2.63 (m, 1H), 2.58 (s, 3H), 2.42 (s, 3H), 2.26 (dtd, *J* = 12.9, 5.4, 2.5 Hz, 1H), 1.48 (s, 9H) ppm.

^{13}C NMR (126 MHz, $(CD_3)_2CO$): δ = 172.8, 171.3, 168.59, 167.5, 148.9, 142.7, 142.3, 136.7, 135.4, 134.9, 123.0, 127.0, 124.6, 83.0, 53.0, 52.1, 48.99, 32.3, 28.1, 23.9, 14.5, 10.1 ppm.

IR (neat): 3472, 3199, 3096, 2977, 2926, 1743, 1694, 1554, 1507, 1410, 1368, 1337, 1319, 1263, 1231, 1202, 1154, 1097, 999, 842, 826, 751 cm^{-1}.

HRMS (ESI): *m/z* calcd. for $C_{24}H_{29}N_6O_5$ $[M+H]^+$ 481.2194, found 481.2181.

Synthesis of 59

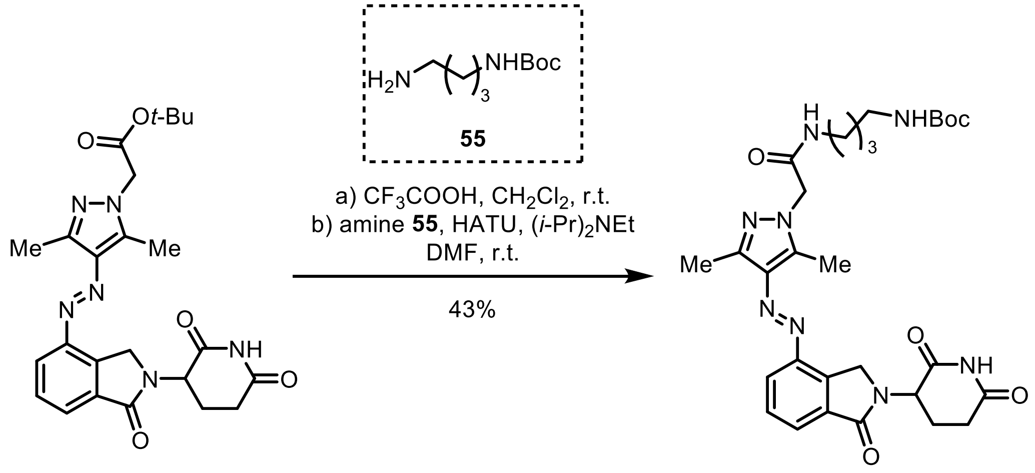

Me$_2$-arylazopyrazole photoswitch **59**

Me$_2$-arylazopyrazole photoswitch (50 mg, 0.10 mmol, 1.0 equiv) was dissolved in CH_2Cl_2 (0.5 mL) and CF_3COOH (0.5 mL) was added. The resulting reaction mixture was stirred at r.t. for 2 h after which it was diluted with toluene and concentrated *in vacuo*. The crude product was used in the next step without further purification.

The crude product, amine **55** (21.5 mg, 114 µmol, 1.1 equiv) and HATU (43.2 mg, 114 µmol, 1.1 equiv) were dissolved in anhydrous DMF (1 mL) and (i-Pr)$_2$NEt (55 µL, 0.31 mmol, 3.0 equiv) was added. The resulting reaction mixture was stirred overnight after which it was concentrated *in vacuo.* Preparative TLC (SiO_2, eluent: acetone–EtOAc (1:1)) and C_{18}-reversed phase column chromatography (eluent: MeCN–H_2O (1:1) to MeCN) afforded **59** (26.8 mg, 43%) as a yellow solid.

Note: The product proved to be unstable in MeOH and was slightly soluble in acetone for handling. For C_{18}-reversed phase column chromatography it proved to be crucial to fully dissolve the product in MeCN–water (1:1) before loading on the column.

^{1}H NMR (500 MHz, $(CD_3)_2SO$): δ = 10.99 (s, 1H), 8.21 (t, J = 5.6 Hz, 1H), 8.01 (dd, J = 7.8, 1.1 Hz, 1H), 7.79 (dd, J = 7.5, 1.1 Hz, 1H), 7.70 (t, J = 7.6 Hz, 1H), 6.80 (t, J = 5.8 Hz, 1H), 5.14 (dd, J = 13.3, 5.1 Hz, 1H), 4.77 (s, 2H), 4.76 – 4.54 (m, 2H), 3.08 (q, J = 6.2 Hz, 2H), 2.91 (q, J = 6.9, 6.5 Hz, 3H), 2.68 – 2.59 (m, 1H), 2.53 (s, 3H), 2.49 – 2.43 (m, 1H), 2.38 (s, 3H), 2.06 (dtd, J = 10.2, 5.4, 2.2 Hz, 1H), 1.37 (s, 13H) ppm.

^{13}C NMR (126 MHz, $(CD_3)_2SO$): δ = 172. 9, 171.0, 167.5, 165.8, 155.6, 147.5, 141.8, 141.1, 135.3, 134.4, 133.6, 129.4, 125.4, 123.6, 77.4, 51.9, 51.6, 48.0, 38.6, 31.3, 28.3, 27.0, 26.4, 22.4, 14.1, 9.8 ppm.

IR (neat): 3347, 3195, 3095, 2978, 2930, 1731, 1711, 1678, 1530, 1515, 1416, 1366, 1262, 1237, 1176, 1099, 816, 751 cm^{-1}.

HRMS (ESI): *m/z* calcd. for $C_{29}H_{39}N_8O_6$ $[M+H]^+$ 595.2987, found 595.2984.

Synthesis of 60

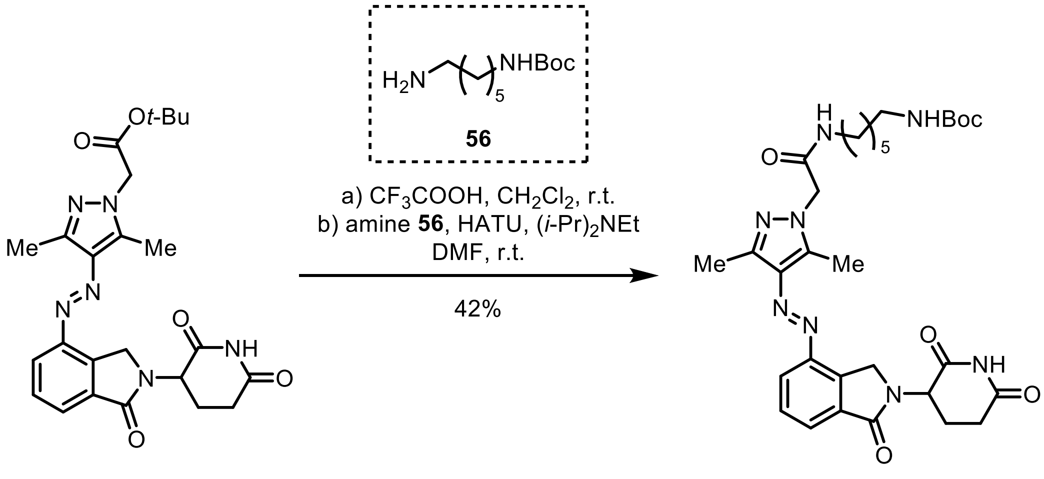

Me$_2$-arylazopyrazole photoswitch (50 mg, 0.10 mmol, 1.0 equiv) was dissolved in CH_2Cl_2 (0.5 mL) and CF_3COOH (0.5 mL) was added. The resulting reaction mixture was stirred at r.t. for 2 h after which it was diluted with toluene and concentrated *in vacuo*. The crude product was used in the next step without further purification.

The crude product, amine **56** (33.6 mg, 156 µmol, 1.5 equiv) and HATU (51.1 mg, 135 µmol, 1.3 equiv) were dissolved in anhydrous DMF (1 mL) and (i-Pr)$_2$NEt (54 µL, 0.31 mmol, 3.0 equiv) was added. The resulting reaction mixture was stirred overnight after which it was concentrated *in vacuo.* Preparative TLC (SiO_2, eluent: acetone–EtOAc (1:1)) and C_{18}-reversed phase column chromatography (eluent: MeCN–H_2O (1:1) to MeCN) afforded **60** (26.9 mg, 42%) as a yellow solid.

Note: The product proved to be instable in MeOH and was slightly soluble in acetone for handling. For C_{18}-reversed phase column chromatography it proved to be crucial to fully dissolve the product in MeCN–water (1:1) before loading on the column.

^{1}H NMR (500 MHz, $(CD_3)_2SO$): δ = 10.99 (s, 1H), 8.20 (t, J = 5.6 Hz, 1H), 8.02 (dd, J = 7.8, 1.1 Hz, 1H), 7.79 (dd, J = 7.5, 1.1 Hz, 1H), 7.70 (t, J = 7.6 Hz, 1H), 6.81 – 6.72 (m, 1H), 5.14 (dd, J = 13.3, 5.1 Hz, 1H), 4.77 (s, 2H), 4.76 – 4.54 (m, 2H), 3.08 (q, J = 6.6 Hz, 2H), 2.96 – 2.84 (m, 3H), 2.61 (ddd, J = 17.3, 4.6, 2.5 Hz, 1H), 2.54 (s, 3H), 2.49 – 2.44 (m, 1H), 2.38 (s, 3H), 2.11 – 2.02 (m, 1H), 1.36 (s, 13H), 1.29 – 1.19 (m, 4H) ppm.

^{13}C NMR (126 MHz, $(CD_3)_2SO$): δ = 172.9, 171.0, 167.5, 165.8, 155.6, 147.5, 141.8, 141.1, 135.3, 134.4, 133.6, 129.4, 125.4, 123.6, 77.3, 51.9, 51.6, 48.0, 38.7, 31.3, 29.4, 28.9, 28.3, 26.1, 26.0, 22.4, 14.1, 9.8 ppm.

IR (neat): 3319, 3222, 3087, 2930, 2858, 1675, 1555, 1511, 1411, 1366, 1251, 1171, 1043, 1000, 753 cm^{-1}.

HRMS (ESI): *m/z* calcd. for $C_{32}H_{47}N_8O_7$ [M+CH_3OH+H]$^+$ 655.3562, found 655.3567.

Synthesis of 61

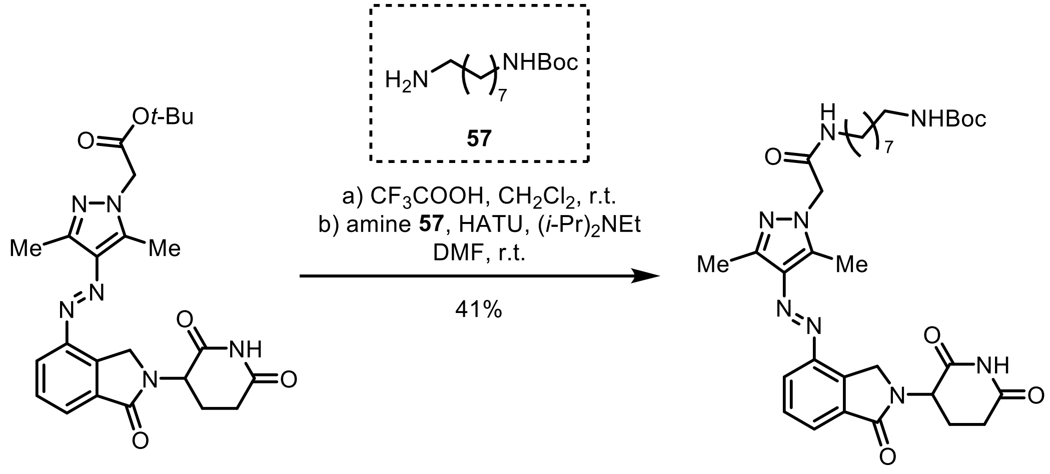

Me$_2$-arylazopyrazole photoswitch 61

Me$_2$-arylazopyrazole photoswitch (50 mg, 0.10 mmol, 1.0 equiv) was dissolved in CH_2Cl_2 (0.5 mL) and CF_3COOH (0.5 mL) was added. The resulting reaction mixture was stirred at r.t. for 2 h after which it was diluted with toluene and concentrated *in vacuo*. The crude product was used in the next step without further purification.

The crude product, amine **57** (38 mg, 156 µmol, 1.5 equiv) and HATU (51.1 mg, 135 µmol, 1.3 equiv) were dissolved in anhydrous DMF (1 mL) and (*i*-Pr)$_2$NEt (54 µL, 0.31 mmol, 3.0 equiv) was added. The resulting reaction mixture was stirred overnight after which it was concentrated *in vacuo.* Preparative TLC (SiO_2, eluent: acetone–EtOAc (1:1)) and C_{18}-reversed phase column chromatography (eluent: MeCN–H_2O (1:1) to MeCN) afforded **61** (27.4 mg, 41%) as a yellow solid.

Note: The product proved to be instable in MeOH and was slightly soluble in acetone for handling. For C_{18}-reversed phase column chromatography it proved to be crucial to fully dissolve the product in MeCN–water (1:1) before loading on the column.

^{1}H NMR (500 MHz, $(CD_3)_2SO$): δ = 10.99 (s, 1H), 8.20 (t, *J* = 5.6 Hz, 1H), 8.01 (dd, *J* = 7.8, 1.1 Hz, 1H), 7.79 (dd, *J* = 7.5, 1.1 Hz, 1H), 7.70 (t, *J* = 7.6 Hz, 1H), 6.74 (t, *J* = 5.6 Hz, 1H), 5.17 – 5.10 (m, 1H), 4.77 (s, 2H), 4.76 – 4.54 (m, 2H), 3.08 (q, *J* = 6.7 Hz, 2H), 2.95 – 2.85 (m, 3H), 2.61 (ddd, *J* = 17.2, 4.3, 2.1 Hz, 1H), 2.53 (s, 3H), 2.49 – 2.45 (m, 1H), 2.38 (s, 3H), 2.09 – 2.03 (m, 1H), 1.36 (s, 13H), 1.31 – 1.17 (m, 8H) ppm.

^{13}C NMR (126 MHz, $(CD_3)_2SO$): δ = 172.9, 171.0, 167.5, 165.8, 155.6, 147.5, 141.8, 141.1, 135.3, 134.4, 133.6, 129.4, 125.4, 123.6, 77.3, 51.9, 51.6, 48.1, 38.7, 31.3, 29.5, 28.9, 28.7, 28.3, 26.3, 26.2, 22.4, 14.1, 9.8 ppm.

IR (neat): 3301, 3187, 3088, 2927, 2855, 1719, 1663, 1563, 1510, 1446, 1412, 1366, 1234, 1210, 1172, 999, 755 cm^{-1}.

HRMS (ESI): *m/z* calcd. for $C_{33}H_{47}N_8O_6$ [M +H]$^+$ 651.3613, found 651.3617.

Synthesis of 62

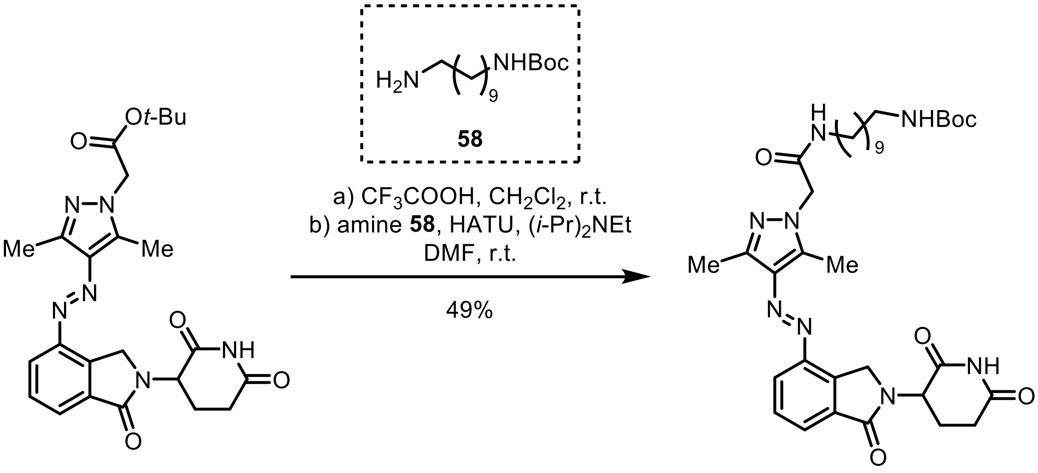

Me$_2$-arylazopyrazole photoswitch **62**

Me$_2$-arylazopyrazole photoswitch (50 mg, 0.10 mmol, 1.0 equiv) was dissolved in CH_2Cl_2 (0.5 mL) and CF_3COOH (0.5 mL) was added. The resulting reaction mixture was stirred at r.t. for 2 h after which it was diluted with toluene and concentrated *in vacuo*. The crude product was used in the next step without further purification.

The crude product, amine **58** (42.4 mg, 156 µmol, 1.5 equiv) and HATU (51.1 mg, 135 µmol, 1.3 equiv) were dissolved in anhydrous DMF (1 mL) and (*i*-Pr)$_2$NEt (54 µL, 0.31 mmol, 3.0 equiv) was added. The resulting reaction mixture was stirred overnight after which it was concentrated *in vacuo.* Preparative TLC (SiO_2, eluent: acetone-EtOAc (1:1)) and C_{18}-reversed phase column chromatography (eluent: MeCN–H_2O (1:1) to MeCN) afforded **62** (34.3 mg, 49%) as a yellow solid.

Note: The product proved to be instable in MeOH and was slightly soluble in acetone for handling. For C_{18}-reversed phase column chromatography it proved to be crucial to fully dissolve the product in MeCN–water (1:1) before loading on the column.

^{1}H NMR (500 MHz, $(CD_3)_2SO$): δ = 10.99 (s, 1H), 8.19 (t, *J* = 5.6 Hz, 1H), 8.02 (dd, *J* = 7.8, 1.1 Hz, 1H), 7.79 (dd, *J* = 7.5, 1.1 Hz, 1H), 7.70 (t, *J* = 7.6 Hz, 1H), 6.74 (t, *J* = 5.7 Hz, 1H), 5.14 (dd, *J* = 13.3, 5.1 Hz, 1H), 4.77 (s, 2H), 4.75 – 4.59 (m, 2H), 3.08 (td, *J* = 7.0, 5.7 Hz, 2H), 2.99 – 2.84 (m, 3H), 2.67 – 2.56 (m, 1H), 2.53 (s, 3H), 2.49 – 2.43 (m, 1H), 2.38 (s, 3H), 2.09 – 2.02 (m, 1H), 1.43 (q, *J* = 7.1 Hz, 2H), 1.39 (s, 11H), 1.30 – 1.17 (m, 12H) ppm.

^{13}C NMR (126 MHz, $(CD_3)_2SO$): δ = 172.9, 171.0, 167.5, 165.8, 155.6, 147.5, 141.8, 141.1, 135.3, 134.4, 133.6, 129.4, 125.4, 123.6, 77.3, 51.9, 51.6, 48.0, 38.7, 31.3, 29.5, 29.1 – 28.8 (m), 28.7, 28.3, 26.4, 26.3, 22.4, 14.1, 9.8 ppm.

IR (neat): 3333, 2923, 2853, 1715, 1678, 1540, 1520, 1444, 1412, 1367, 1246, 1210, 1171, 1046, 1011, 752 cm^{-1}.

HRMS (ESI): *m/z* calcd. for $C_{35}H_{51}N_8O_6$ $[M+H]^+$ 679.3926, found 679.3919.

Synthesis of KH-5-298

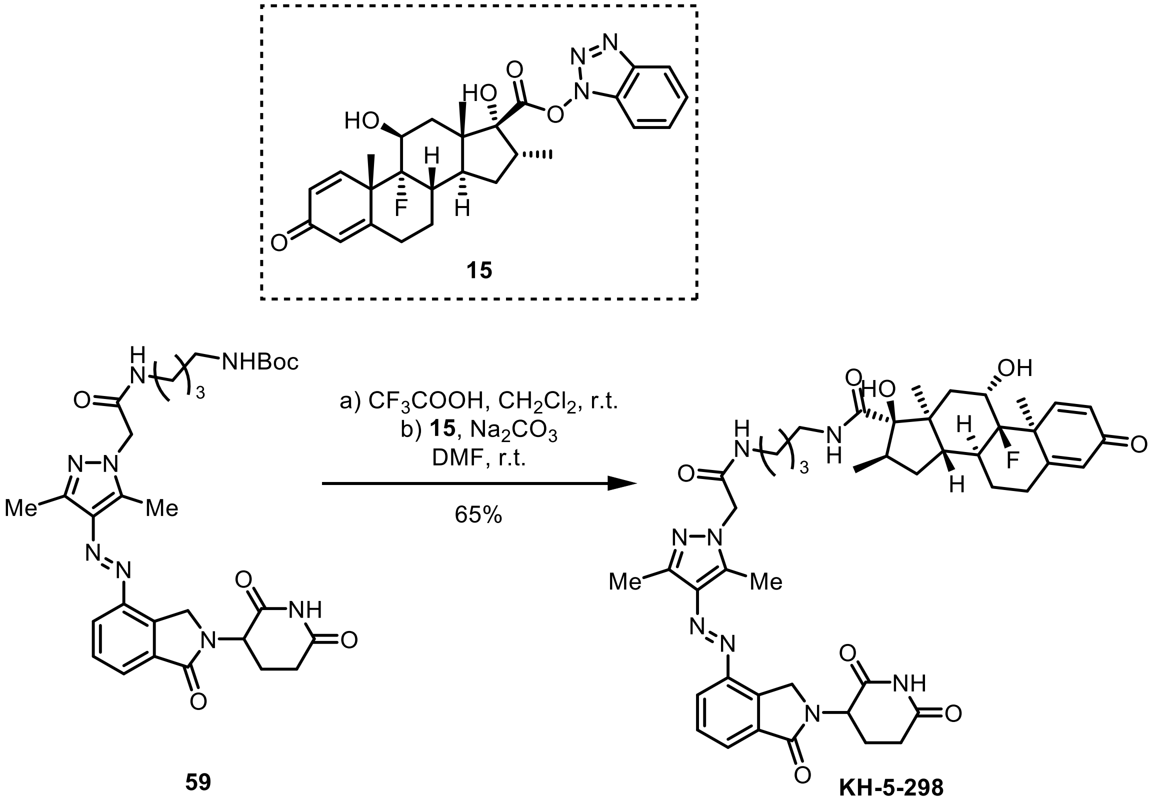

59 KH-5-298

59 (16.6 mg, 27.9 µmol, 1.00 equiv) was dissolved in CH_2Cl_2 (0.15 mL) and CF_3COOH (0.15 mL) was added. The resulting reaction mixture was stirred at r.t. for 2 h after which it was diluted with toluene and concentrated *in vacuo*. The crude product was used in the next step without further purification.

The crude product, **15** (14.4 mg, 29.1 µmol, 1.05 equiv) and Na_2CO_3 (8.8 mg, 83 µmol, 3.0 equiv) were dissolved in anhydrous DMF (0.28 mL). The resulting reaction mixture was stirred for 24 h after which it was concentrated *in vacuo.* Preparative TLC (SiO_2, eluent: acetone–EtOAc (1:1)) and C_{18}-reversed phase column chromatography (eluent: MeCN–H_2O (1:1) to MeCN) afforded **KH-5-298** (15.5 mg, 65%) as a yellow solid.

Note: The product proved to be instable in MeOH and was slightly soluble in acetone for handling. For C_{18}-reversed phase column chromatography it proved to be crucial to fully dissolve the product in MeCN–water (1:1) before loading on the column.

^{1}H NMR (500 MHz, $(CD_3)_2SO$): δ = 10.99 (s, 1H), 8.30 – 8.21 (m, 1H), 8.01 (dd, *J* = 7.9, 1.1 Hz, 1H), 7.79 (dd, *J* = 7.5, 1.1 Hz, 1H), 7.70 (t, *J* = 7.7 Hz, 1H), 7.37 (t, *J* = 6.0 Hz, 1H), 7.25 (dd, *J* = 10.1, 2.8 Hz, 1H), 6.19 (dt, *J* = 10.1, 2.1 Hz, 1H), 5.99 (s, 1H), 5.19 – 5.10 (m, 2H), 4.83 – 4.57 (m, 5H), 4.09 (d, *J* = 11.1 Hz, 1H), 3.21 – 3.13 (m, 1H), 3.10 (d, *J* = 5.9 Hz, 2H), 3.07 – 2.97 (m, 2H), 2.92 (ddd, *J* = 18.0, 13.5, 5.4 Hz, 1H), 2.66 – 2.57 (m, 2H), 2.56 (s, 3H), 2.39 (s, 3H), 2.29 (qd, *J* =

12.2, 4.8 Hz, 1H), 2.11 – 1.98 (m, 3H), 1.82 – 1.70 (m, 1H), 1.59 (q, *J* = 11.7 Hz, 1H), 1.50 – 1.28 (m, 9H), 1.04 (ddd, *J* = 12.1, 8.2, 4.3 Hz, 1H), 0.93 (s, 3H), 0.79 (d, *J* = 7.2 Hz, 3H) ppm.

^{13}C NMR (126 MHz, $(CD_3)_2SO$): δ = 185.3, 172.9, 172.2, 171.0, 167.5, 167.2, 166.0 (d, *J* = 1.2 Hz), 152.9, 147.5, 141.8, 141.2, 135.3, 134.4, 133.6, 129.4, 129.0, 125.4, 124.1, 123.6, 102.2, 100.8, 86.4, 70.9 (d, *J* = 37.3 Hz), 51.9 (d, *J* = 2.0 Hz), 51.6, 48.2 – 47.9 (m), 47.4, 43.0, 38.7, 38.3, 35.5, 34.3, 33.8 (d, *J* = 19.3 Hz), 32.0, 31.3, 30.2, 27.2, 26.8, 26.4, 22.9 (d, *J* = 5.7 Hz), 22.4, 17.0, 15.2, 14.1, 9.8 ppm.

^{19}F NMR (471 MHz, $(CD_3)_2SO$): δ = -164.16 (d, J = 1.6 Hz) ppm.

IR (neat): 3357, 3081, 2934, 2870, 1663, 1513, 1412, 1372, 1234, 1202, 1142, 1027, 1007, 893, 821, 754 cm^{-1}.

HRMS (ESI): *m/z* calcd. for $C_{45}H_{56}FN_8O_8$ $[M+H]^+$ 855.4200, found 855.4200.

Synthesis of KH-5-302

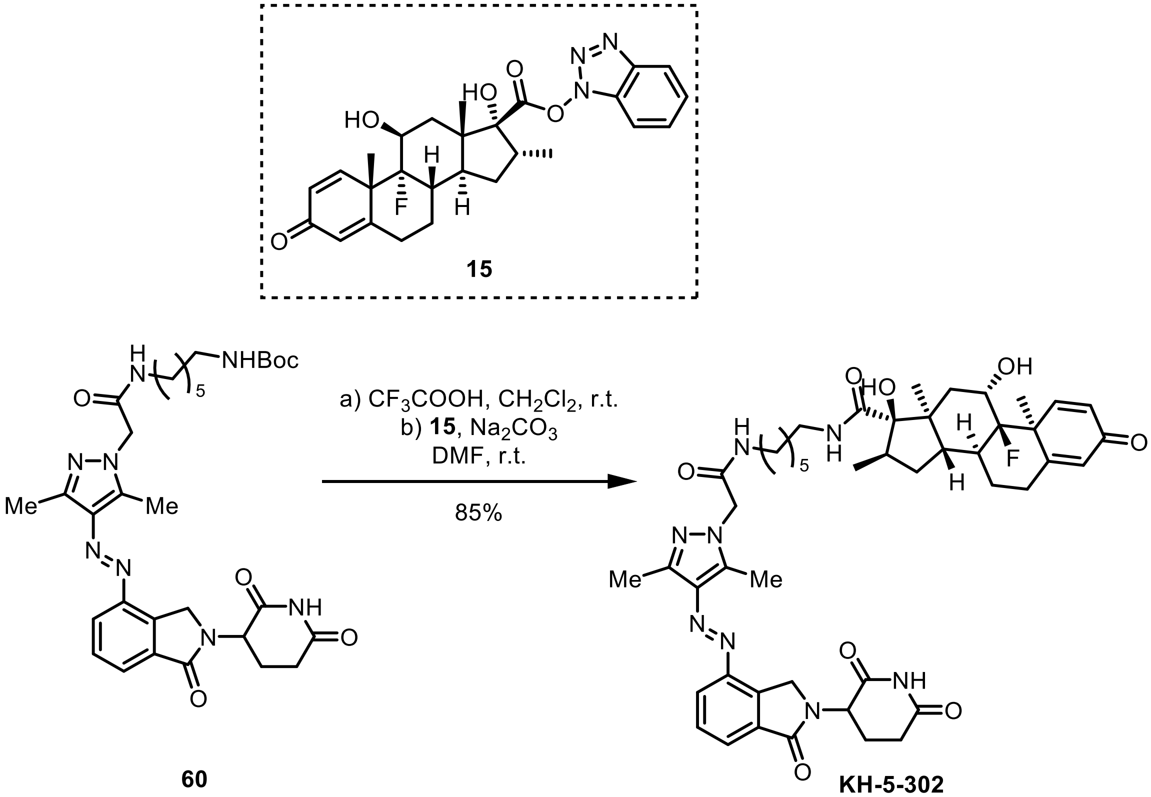

60 KH-5-302

60 (13 mg, 21 µmol, 1.0 equiv) was dissolved in anhydrous CH_2Cl_2 (0.1 mL) and CF_3COOH (0.1 mL) was added. The resulting reaction mixture was stirred at r.t. for 2 h after which it was diluted with toluene and concentrated *in vacuo*. The crude product was used in the next step without further purification.

The crude product, **15** (10.8 mg, 21.7 µmol, 1.05 equiv) and Na_2CO_3 (6.6 mg, 62 µmol, 3.0 equiv) were dissolved in anhydrous DMF (0.21 mL). The resulting reaction mixture was stirred for 24 h after which it was concentrated *in vacuo.* Preparative TLC (SiO_2, eluent: acetone–EtOAc (1:1)) and C_{18}-reversed phase column chromatography (eluent: MeCN–H_2O (1:1) to MeCN) afforded **KH-5-302** (15.6 mg, 85%) as a yellow solid.

Note: The product proved to be instable in MeOH and was slightly soluble in acetone for handling. For C_{18}-reversed phase column chromatography it proved to be crucial to fully dissolve the product in MeCN–water (1:1) before loading on the column.

^{1}H NMR (500 MHz, $(CD_3)_2SO$): δ = 10.99 (s, 1H), 8.22 (t, *J* = 5.6 Hz, 1H), 8.02 (dd, *J* = 7.8, 1.1 Hz, 1H), 7.79 (dd, *J* = 7.5, 1.1 Hz, 1H), 7.70 (t, *J* = 7.7 Hz, 1H), 7.34 (t, *J* = 6.0 Hz, 1H), 7.27 (dd, *J* = 10.1, 1.2 Hz, 1H), 6.20 (dd, *J* = 10.1, 1.9 Hz, 1H), 5.99 (d, *J* = 1.8 Hz, 1H), 5.24 (dd, *J* = 4.4, 1.7 Hz, 1H), 5.14 (dd, *J* = 13.2, 5.1 Hz, 1H), 4.82 – 4.59 (m, 5H), 4.13 – 4.07 (m, 1H), 3.12 (dq, *J* = 20.7, 6.6 Hz, 3H), 3.01 (p, *J* = 6.3 Hz, 2H), 2.92 (ddd, *J* = 18.1, 13.6, 5.4 Hz, 1H), 2.66 – 2.56 (m, 2H), 2.54 (s, 3H), 2.38 (s, 3H), 2.29 (ddt, *J* = 17.0, 11.3, 5.4 Hz, 1H), 2.09 – 1.99 (m, 3H), 1.81 –

1.72 (m, 1H), 1.60 (q, *J* = 11.8 Hz, 1H), 1.47 (d, *J* = 1.3 Hz, 3H), 1.46 – 1.35 (m, 5H), 1.34 – 1.22 (m, 5H), 1.05 (ddd, *J* = 12.3, 8.3, 4.4 Hz, 1H), 0.94 (s, 3H), 0.79 (d, *J* = 7.2 Hz, 3H) ppm.

^{13}C NMR (126 MHz, $(CD_3)_2SO$): δ = 185.3, 172.9, 172.1, 171.0, 167.5, 167.2, 165.9, 152.9, 147.5, 141.8, 141.2, 135.3, 134.4, 133.6, 129.4, 129.0, 125.4, 124.1, 123.6, 102.3, 100.9, 86.3, 70.8 (d, *J* = 37.2 Hz), 51.9, 51.6, 48.3 – 47.9 (m), 47.4, 43.0, 38.7, 38.6, 35.5, 34.3, 33.8 (d, *J* = 19.4 Hz), 32.0, 31.3, 30.3, 29.3, 28.9, 27.3, 26.1, 22.9 (d, *J* = 5.7 Hz), 22.4, 16.9, 15.3, 14.1, 9.8 ppm.

^{19}F NMR (471 MHz, $(CD_3)_2SO$): δ = -164.04 ppm.

IR (neat): 3361, 3084, 2933, 2865, 1693, 1663, 1559, 1513, 1411, 1372, 1266, 1235, 1203, 1027, 1007, 893, 821, 755 cm^{-1}.

HRMS (ESI): *m/z* calcd. for $C_{47}H_{60}FN_8O_8$ $[M+H]^+$ 883.4513, found 883.4520.

Synthesis of KH-5-306

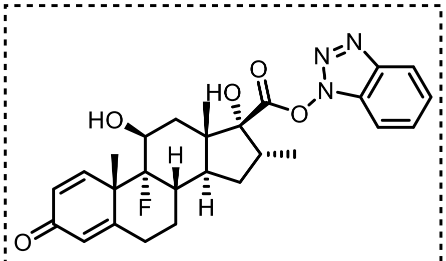

15

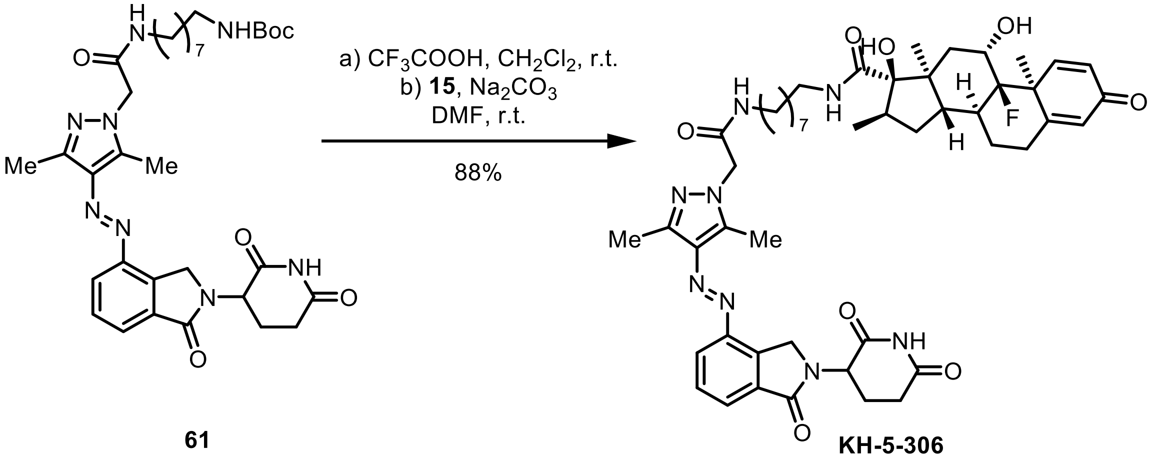

61 **KH-5-306**

61 (23.6 mg, 36.3 µmol, 1.0 equiv) was dissolved in CH_2Cl_2 (0.18 mL) and CF_3COOH (0.18 mL) was added. The resulting reaction mixture was stirred at r.t. for 2 h after which it was diluted with toluene and concentrated *in vacuo*. The crude product was used in the next step without further purification.

The crude product, **15** (18.8 mg, 37.9 µmol, 1.05 equiv) and Na_2CO_3 (11.5 mg, 108 µmol, 3.00 equiv) were dissolved in anhydrous DMF (0.36 mL). The resulting reaction mixture was stirred for 24 h after which it was concentrated *in vacuo.* Preparative TLC (SiO_2, eluent: acetone–EtOAc (1:1)) and C_{18}-reversed phase column chromatography (eluent: MeCN–H_2O (1:1) to MeCN) afforded **KH-5-306** (29.1 mg, 88%) as a yellow solid.

Note: The product proved to be instable in MeOH and was slightly soluble in acetone for handling. For C_{18}-reversed phase column chromatography it proved to be crucial to fully dissolve the product in MeCN–water (1:1) before loading on the column.

^{1}H NMR (500 MHz, $(CD_3)_2SO$): δ = 10.99 (s, 1H), 8.20 (t, *J* = 5.6 Hz, 1H), 8.02 (dd, *J* = 7.8, 1.1 Hz, 1H), 7.79 (dd, *J* = 7.5, 1.1 Hz, 1H), 7.70 (t, *J* = 7.6 Hz, 1H), 7.35 – 7.25 (m, 2H), 6.21 (dd, *J* = 10.1, 1.9 Hz, 1H), 6.00 (t, *J* = 1.8 Hz, 1H), 5.24 (dd, *J* = 4.5, 1.7 Hz, 1H), 5.14 (dd, *J* = 13.2, 5.1 Hz, 1H), 4.81 – 4.58 (m, 5H), 4.13 – 4.07 (m, 1H), 3.11 (dp, *J* = 19.3, 6.8 Hz, 3H), 3.01 (p, *J* = 6.6 Hz, 2H), 2.92 (ddd, *J* = 18.1, 13.5, 5.4 Hz, 1H), 2.61 (dd, *J* = 13.4, 6.9 Hz, 2H), 2.54 (s, 3H), 2.38 (s, 3H), 2.31 (qd, *J* = 16.4, 14.5, 5.0 Hz, 1H), 2.09 – 1.99 (m, 3H), 1.79 – 1.72 (m, 1H), 1.60 (q, *J* = 11.8 Hz, 1H), 1.48 (s, 3H), 1.46 – 1.31 (m, 5H), 1.30 – 1.21 (m, 9H), 1.05 (ddd, *J* = 12.2, 8.3, 4.3 Hz, 1H), 0.94 (s, 3H), 0.78 (d, *J* = 7.2 Hz, 3H) ppm.

^{13}C NMR (126 MHz, $(CD_3)_2SO$): δ = ^{13}C NMR (126 MHz, DMSO-d_6) δ 185.29, 172.89, 172.10, 171.01, 167.46, 167.21, 165.82, 152.89, 147.50, 141.79, 141.13, 135.28, 134.39, 133.57, 129.37, 128.97, 125.40, 124.07, 123.61, 102.26, 100.87, 86.33, 70.84 (d, J = 37.1 Hz), 51.86, 51.61, 48.29 – 47.85 (m), 47.43, 43.02, 38.77, 38.64, 35.52, 34.35, 33.83 (d, J = 19.4 Hz), 31.97, 31.26, 30.32, 29.38, 28.95, 28.72, 28.69, 27.29 (d, J = 1.9 Hz), 26.37 (d, J = 3.0 Hz), 22.89 (d, J = 5.8 Hz), 22.37, 16.93, 15.15, 14.07, 9.80 ppm.

^{19}F NMR (471 MHz, $(CD_3)_2SO$): δ = -164.03 ppm.

IR (neat): 3356, 2993, 2949, 2710, 2522, 1672, 1468, 1406, 1178, 1200, 1132, 1071, 1034, 930, 830, 799, 719 cm^{-1}.

HRMS (ESI): *m/z* calcd. for $C_{49}H_{63}FN_8O_8Na$ $[M+Na]^+$ 933.4645, found 933.4649.

Synthesis of KH-5-309

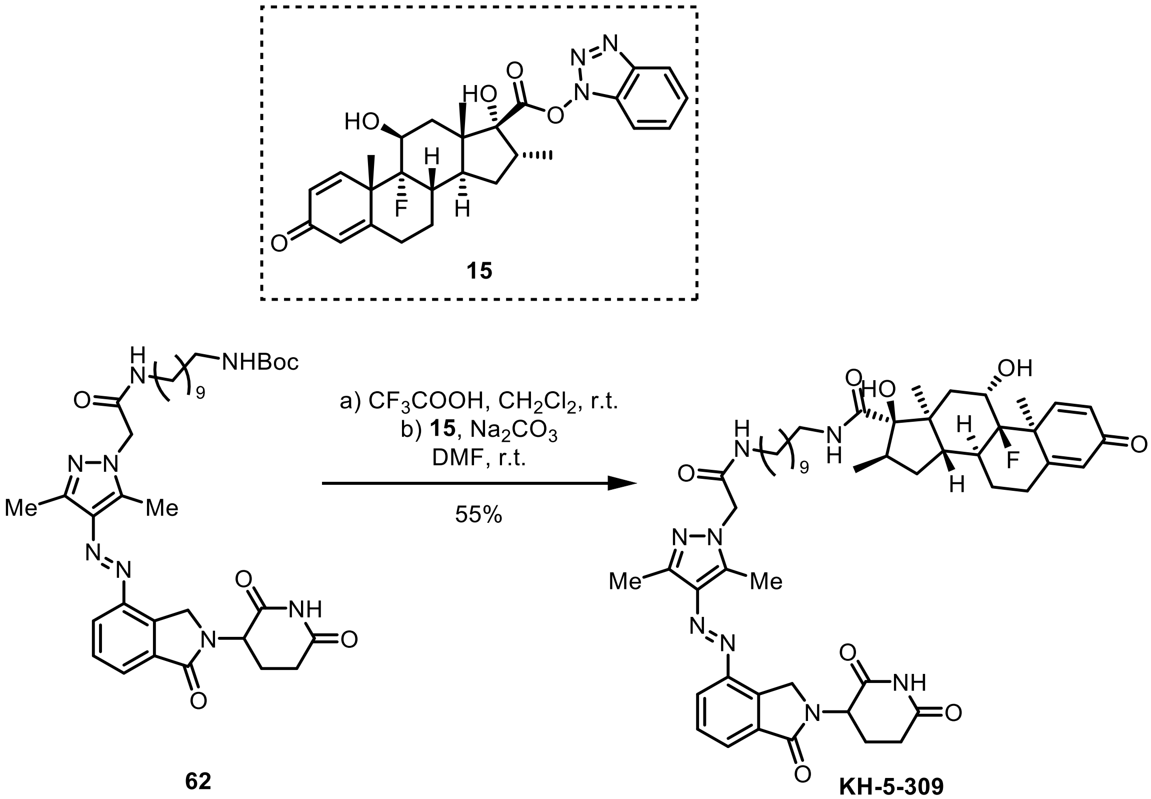

62 KH-5-309

62 (34.2 mg, 50.0 µmol, 1.0 equiv) was dissolved in CH_2Cl_2 (0.18 mL) and CF_3COOH (0.18 mL) was added. The resulting reaction mixture was stirred at r.t. for 2 h after which it was diluted with toluene and concentrated *in vacuo*. The crude product was used in the next step without further purification.

The crude product, **15** (26.2 mg, 52.8 µmol, 1.05 equiv) and Na_2CO_3 (16 mg, 0.15 mmol, 3.0 equiv) were dissolved in anhydrous DMF (0.5 mL). The resulting reaction mixture was stirred for 24 h after which it was concentrated *in vacuo.* Preparative TLC (SiO_2, eluent: acetone-EtOAc (1:1)) and C_{18}-reversed phase column chromatography (eluent: MeCN–H_2O (1:1) to MeCN) afforded **KH-5-309** (26.0 mg, 55%) as a yellow solid.

Note: The product proved to be instable in MeOH and was slightly soluble in acetone for handling. For C_{18}-reversed phase column chromatography it proved to be crucial to fully dissolve the product in MeCN–water (1:1) before loading on the column.

^{1}H NMR (500 MHz, $(CD_3)_2SO$): δ = 10.99 (s, 1H), 8.19 (t, J = 5.6 Hz, 1H), 8.05 – 7.99 (m, 1H), 7.79 (dd, J = 7.5, 1.1 Hz, 1H), 7.70 (t, J = 7.5 Hz, 1H), 7.35 – 7.27 (m, 2H), 6.21 (dd, J = 10.1, 1.9 Hz, 1H), 6.00 (t, J = 1.7 Hz, 1H), 5.24 (dd, J = 4.5, 1.7 Hz, 1H), 5.14 (dd, J = 13.2, 5.1 Hz, 1H), 4.84 – 4.55 (m, 5H), 4.10 (d, J = 11.2 Hz, 1H), 3.10 (dq, J = 13.1, 6.5 Hz, 3H), 2.99 (pd, J = 8.0, 7.4, 4.2 Hz, 2H), 2.94 – 2.85 (m, 1H), 2.66 – 2.57 (m, 2H), 2.54 (d, J = 3.1 Hz, 3H), 2.39 (d, J = 4.1 Hz, 3H), 2.35 – 2.28 (m, 1H), 2.12 – 1.98 (m, 3H), 1.81 – 1.72 (m, 1H), 1.60 (q, J = 11.8 Hz, 1H), 1.48 (s,

3H), 1.46 – 1.35 (m, 5H), 1.25 (s, 13H), 1.05 (ddd, J = 12.2, 8.1, 4.1 Hz, 1H), 0.94 (s, 3H), 0.78 (d, J = 7.1 Hz, 3H) ppm.

^{13}C NMR (126 MHz, $(CD_3)_2SO$): δ = 185.3, 172.9, 172.1, 171.0, 167.5, 167.2, 165.8, 152.9, 147.5, 141.8, 141.1, 135.3, 134.4, 133.6, 129.4, 129.0, 125.4, 124.1, 123.6, 102.3, 100.9, 86.3, 70.9 (d, J = 37.0 Hz), 51.9, 51.6, 48.4 – 47.8 (m), 47.4, 43.0, 38.8, 38.7, 35.5, 34.4, 33.8 (d, J = 19.1 Hz), 32.0, 31.3, 30.3, 29.6, 29.4, 29.1 – 28.9 (m), 28.8 (d, J = 5.6 Hz), 27.3, 26.4 (d, J = 4.9 Hz), 22.9 (d, J = 5.7 Hz), 22.4, 16.9, 15.1, 14.1, 9.8 ppm.

^{19}F NMR (471 MHz, $(CD_3)_2SO$): δ = -164.03 ppm.

IR (neat): 3375, 3090, 2929, 2856, 1663, 1537, 1412, 1370, 1298, 1265, 1233, 1142, 1035, 893 cm^{-1}.

HRMS (ESI): *m/z* calcd. for $C_{51}H_{68}FN_8O_8$ $[M+H]^+$ 939.5139, found 939.5133.

GR $(CF_3)_2$-Arylazopyrazole photoPROTACs

Synthesis 43

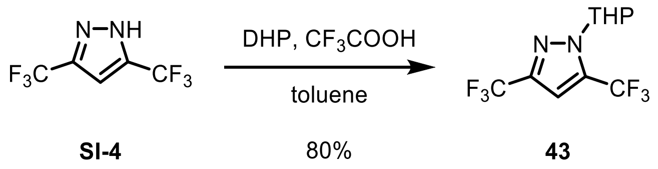

DHP (6.7 mL, 74 mmol, 1.5 equiv) was added to a solution of pyrazole **SI-4** (10 g, 49 mmol, 1.0 equiv) in toluene (67 mL) and CF_3COOH (0.19 mL, 2.4 mmol, 0.05 equiv). After refluxing the reaction solution overnight, the solvent was removed and the crude product was purified by column chromatography (SiO_2, eluent: hexanes to 10% EtOAc in hexanes) to afford **43** (11.3 g, 80%) as a colorless oil.

^{1}H NMR (400 MHz, $CDCl_3$): δ = 6.92 (s, 1H), 5.53 (dd, *J* = 9.1, 2.9 Hz, 1H), 4.02 (dtd, *J* = 11.7, 3.7, 1.6 Hz, 1H), 3.74 – 3.62 (m, 1H), 2.46 (dddd, *J* = 13.7, 11.0, 9.0, 4.5 Hz, 1H), 2.18 – 2.07 (m, 1H), 2.00 (dqd, *J* = 12.0, 3.3, 1.6 Hz, 1H), 1.79 – 1.67 (m, 2H), 1.64 (dddd, *J* = 11.5, 6.2, 3.0, 1.4 Hz, 1H) ppm.

^{13}C NMR (101 MHz, $CDCl_3$): δ = 141.9 (t, *J* = 39.5 Hz), 135.0 – 132.5 (m), 119.9 (qd, *J* = 269.3, 125.3 Hz), 107.6 – 106.0 (m), 86.7 – 86.3 (m), 67.9, 29.3, 24.6, 22.0 ppm.

^{19}F NMR (376 MHz, $CDCl_3$): δ = -59.65, -62.49 ppm.

IR (neat): 2961, 2867, 1568, 1471, 1459, 1292, 1274, 1246, 1225, 1207, 1129, 1087, 1044, 998, 976, 917, 883, 830, 748 cm^{-1}.

HRMS (ESI): *m/z* calcd. for $C_{10}H_{10}F_6N_2ONa$ $[M+Na]^+$ 311.0590, found 311.0591.

Synthesis of 44

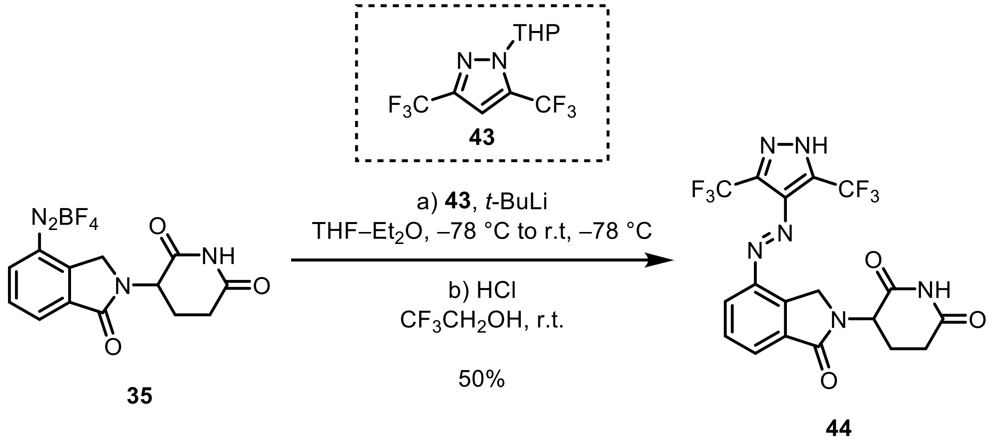

t-BuLi (1.7 M in pentane, 0.27 mL, 0.46 mmol, 1.1 equiv) was added to a solution of pyrazole **43** (144 mg, 501 µmol, 1.2 equiv) in ether (2 mL) at –78 °C. The resulting reaction solution was allowed to warm to r.t. during a period of 30 min. In the meantime, **35** (0.15 g, 0. 42 µmol, 1.0 equiv) was dissolved in THF (2.5 mL) and cooled to –78 °C. The lithiated pyrazole mixture was then added slowly to **35** in THF at –78 °C. After stirring the reaction mixture for 30 min at -78 °C, EtOAc was added and the reaction mixture was allowed to warm to r.t. The reaction mixture was filtered through a short plug of silica (SiO_2, eluent: EtOAc) and washed with EtOAc to afford **43** (159 mg, 68%) as an orange foam.

HCl (77 µL, 2.6 mmol, 3.3 equiv) was added to a solution of **43** (430 mg, 770 µmol, 1.00 equiv) in CF_3CH_2OH (7.7 mL). After stirring the resulting reaction solution for 1.5 h at r.t., it was concentrated *in vacuo*. The solid was resuspended in EtOAc–acetone (1:1), filtered and washed with EtOAc–acetone (1:1) to afford **44** (270 mg, 74% (50% over two steps)) as a yellow solid.

^{1}H NMR (600 MHz, $(CD_3)_2SO$): δ = 15.9 (s, 1H), 11.1 (s, 1H), 8.1 (dd, J = 7.8, 1.0 Hz, 1H), 8.0 (dd, J = 7.5, 1.0 Hz, 1H), 7.9 – 7.8 (m, 1H), 5.2 (dd, J = 13.3, 5.1 Hz, 1H), 4.7 (d, J = 18.7 Hz, 1H), 4.6 (d, J = 18.7 Hz, 1H), 3.0 – 2.9 (m, 1H), 2.7 (dddd, J = 17.3, 4.6, 2.3, 1.0 Hz, 1H), 2.4 – 2.3 (m, 1H), 2.1 – 2.1 (m, 1H) ppm.

^{13}C NMR (151 MHz, $(CD_3)_2SO$): δ = 173.3, 171.4, 167.4, 147.0, 135.1, 134.7, 134.0, 130.5, 129.1, 127.3, 123.5 – 117.4 (m), 52.3, 48.4, 31.7, 23.1 ppm.

^{19}F NMR (565 MHz, $(CD_3)_2SO$): δ = -59.91 ppm.

IR (neat): 3199, 3095, 2855, 1711, 1671, 1599,1495, 1361, 1235, 1197, 1141, 1028, 979, 754, 669 cm^{-1}.

HRMS (ESI): m/z calcd. for $C_{18}H_{13}F_6N_6O_3$ $[M+H]^+$ 475.0948, found 475.0947.

Synthesis of $(CF_3)_2$-arylazopyrazole photoswitch

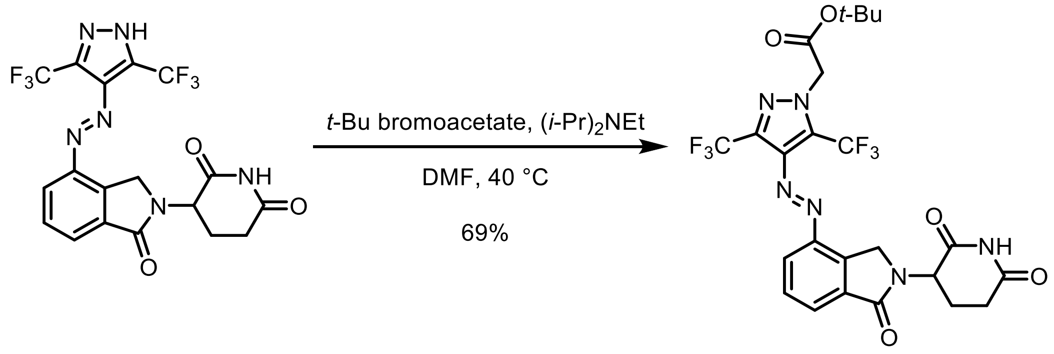

44 **$(CF_3)_2$-arylazopyrazole photoswitch**

t-Bu bromoacetate (16 µL, 0.11 mmol, 1.03 equiv) was added in four portions to a suspension of **44** (50 mg, 0.11 mmol, 1.0 equiv) in DMF (1.1 mL) and (i-Pr)$_2$NEt (50 µL, 0.29 mmol, 3.0 equiv) over the course of four hours. After stirring the reaction mixture for another hour, it was concentrated in *vacuo* and purified by column chromatography (SiO_2, eluent: hexane–EtOAc (1:1)) to afford **$(CF_3)_2$-arylazopyrazole photoswitch** (43.1 mg, 69%) as an orange foam.

^{1}H NMR (500 MHz, $(CD_3)_2CO$): δ = 9.83 (s, 1H), 8.22 (dd, *J* = 7.9, 1.1 Hz, 1H), 8.01 (dd, *J* = 7.5, 1.1 Hz, 1H), 7.88 – 7.83 (m, 1H), 5.39 (q, *J* = 1.4 Hz, 2H), 5.25 (dd, *J* = 13.4, 5.1 Hz, 1H), 4.83 – 4.70 (m, 2H), 3.03 (ddd, *J* = 17.5, 13.6, 5.4 Hz, 1H), 2.82 (ddd, *J* = 17.5, 4.5, 2.4 Hz, 1H), 2.64 – 2.51 (m, 1H), 2.30 (dtd, *J* = 12.9, 5.3, 2.4 Hz, 1H), 1.50 (s, 9H) ppm.

^{13}C NMR (126 MHz, $(CD_3)_2CO$): δ = 172.7, 171.16, 168.0, 166.3, 147.9, 135.9, 135.4, 132.9 (q, *J* = 39.9 Hz), 131.7 (q, *J* = 40.1 Hz), 130.6, 130.1, 128.0, 124.7 – 116.8 (m), 84.4, 56.4 (d, *J* = 2.6 Hz), 53.1, 48.9, 32.2, 28.0, 23.9 ppm.

^{19}F NMR (471 MHz, $(CD_3)_2CO$): δ = -58.15, -62.90 ppm.

IR (neat): 3214, 3102, 2981, 2937, 1744, 1701, 1497, 1458, 1430, 1415, 1396, 1371, 1276, 1247, 1186, 1151, 1099, 1054, 869, 832, 751 cm^{-1}.

HRMS (ESI): *m/z* calcd. for $C_{24}H_{23}F_6N_6O_5$ $[M+H]^+$ 589.1629, found 589.1626.

Synthesis of 63

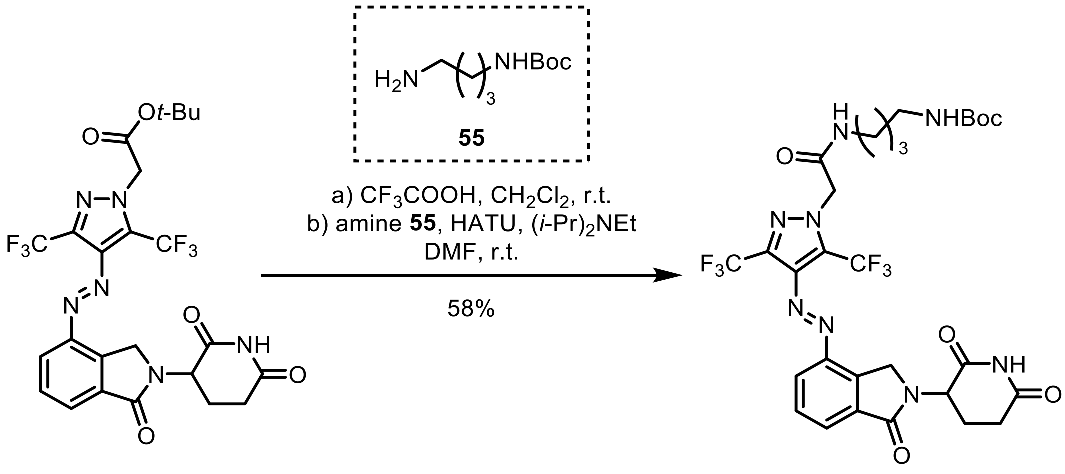

(CF$_3$)$_2$-arylazopyrazole photoswitch **63**

(CF$_3$)$_2$-arylazopyrazole photoswitch (30 mg, 0.51 mmol, 1.0 equiv) was dissolved in anhydrous CH_2Cl_2 (0.25 mL) and CF_3COOH (0.25 mL) was added. The resulting reaction mixture was stirred at r.t. for 2 h after which it was diluted with toluene and concentrated *in vacuo*. The crude product was used in the next step without further purification.

The crude product, amine **55** (10.5 mg, 55.8 μmol, 1.1 equiv) and HATU (20.2 mg, 53.3 μmol, 1.05 equiv) were dissolved in anhydrous DMF (0.5 mL) and (*i*-Pr)$_2$NEt (26 μL, 0.15 mmol, 3.00 equiv) was added. The resulting reaction mixture was stirred overnight after which it was concentrated *in vacuo.* Column chromatography (SiO_2, eluent: hexanes–EtOAc (1:2) to EtOAc) afforded **63** (20.5 mg, 58%) as a yellow solid.

^{1}H NMR (500 MHz, $(CD_3)_2CO$): δ = 9.82 (s, 1H), 8.21 (dd, *J* = 7.8, 1.1 Hz, 1H), 8.00 (dd, *J* = 7.5, 1.0 Hz, 1H), 7.88 – 7.82 (m, 1H), 7.68 (d, *J* = 6.1 Hz, 1H), 5.96 (s, 1H), 5.33 (d, *J* = 1.3 Hz, 2H), 5.24 (dd, *J* = 13.4, 5.1 Hz, 1H), 4.82 – 4.69 (m, 2H), 3.35 – 3.26 (m, 2H), 3.11 – 3.04 (m, 2H), 3.04 – 2.98 (m, 1H), 2.82 (ddd, *J* = 17.5, 4.5, 2.4 Hz, 1H), 2.57 (qd, *J* = 13.3, 4.5 Hz, 1H), 2.30 (dtd, *J* = 12.9, 5.3, 2.4 Hz, 1H), 1.62 – 1.49 (m, 4H), 1.39 (s, 9H) ppm.

^{13}C NMR (126 MHz, $(CD_3)_2CO$): δ = 172.7 (d, *J* = 9.3 Hz), 171.1 (d, *J* = 8.8 Hz), 168.0, 165.3 (d, *J* = 10.6 Hz), 156.8, 148.0, 137.2, 135.9, 135.4, 133.5 – 131.2 (m), 130.6, 130.0, 127.8, 125.4 – 117.6 (m), 78.4, 57.0, 53.1, 48.9, 40.6 (d, *J* = 16.0 Hz), 40.0 (d, *J* = 16.2 Hz), 32.2 (d, *J* = 2.8 Hz), 28.7, 28.2, 27.4 (d, *J* = 2.0 Hz), 24.0 ppm.

^{19}F NMR (471 MHz, $(CD_3)_2CO$) δ -57.95, -62.90 ppm.

IR (neat): 3347, 3195, 3095, 2978, 2930, 1731, 1711, 1678, 1530, 1515, 1416, 1366, 1262, 1237, 1176, 1099, 816, 751 cm^{-1}.

HRMS (ESI): *m/z* calcd. for $C_{29}H_{32}F_6N_8O_6Na$ $[M+Na]^+$ 725.2241, found 725.2242.

Synthesis of 64

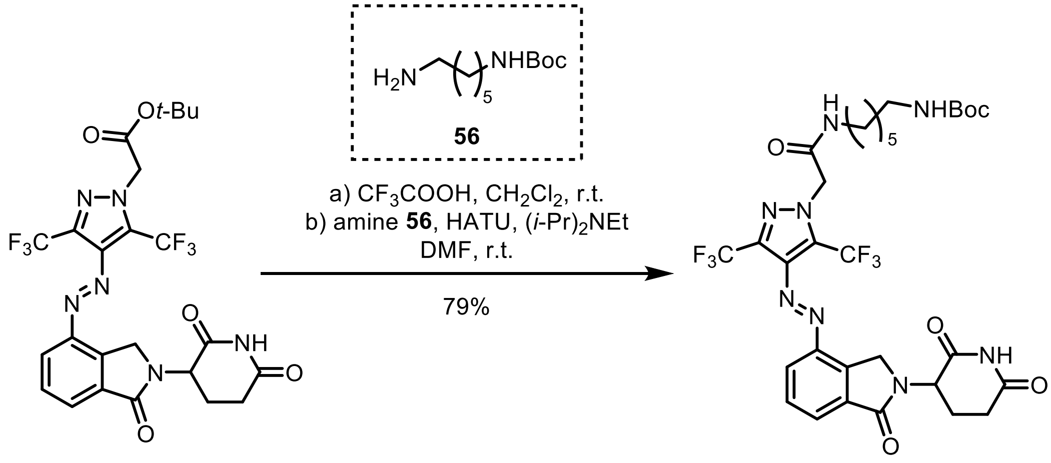

(CF$_3$)$_2$-arylazopyrazole photoswitch

64

(CF$_3$)$_2$-arylazopyrazole photoswitch (30 mg, 0.51 mmol, 1.0 equiv) was dissolved in CH_2Cl_2 (0.25 mL) and CF_3COOH (0.25 mL) was added. The resulting reaction mixture was stirred at r.t. for 2 h after which it was diluted with toluene and concentrated *in vacuo*. The crude product was used in the next step without further purification.

The crude product, amine **56** (12.1 mg, 55.8 µmol, 1.1 equiv) and HATU (20.2 mg, 53.3 µmol, 1.05 equiv) were dissolved in anhydrous DMF (0.5 mL) and (*i*-Pr)$_2$NEt (26 µL, 0.15 mmol, 3.00 equiv) was added. The resulting reaction mixture was stirred overnight after which it was concentrated *in vacuo.* Column chromatography (SiO_2, eluent: hexanes–EtOAc (1:2) to EtOAc) afforded **64** (29.1 mg, 79%) as a yellow solid.

^{1}H NMR (500 MHz, $(CD_3)_2CO$): δ = 9.83 (s, 1H), 8.21 (dd, *J* = 7.8, 1.1 Hz, 1H), 8.00 (dd, *J* = 7.5, 1.1 Hz, 1H), 7.88 – 7.81 (m, 1H), 7.66 – 7.59 (m, 1H), 5.93 (s, 1H), 5.32 (d, *J* = 1.4 Hz, 2H), 5.24 (dd, *J* = 13.4, 5.1 Hz, 1H), 4.84 – 4.69 (m, 2H), 3.32 – 3.24 (m, 2H), 3.10 – 2.98 (m, 3H), 2.82 (ddd, *J* = 17.6, 4.5, 2.4 Hz, 1H), 2.64 – 2.52 (m, 1H), 2.30 (ddq, *J* = 10.5, 5.3, 2.7 Hz, 1H), 1.58 – 1.44 (m, 4H), 1.43 – 1.31 (m, 13H) ppm.

^{13}C NMR (126 MHz, $(CD_3)_2CO$): δ = 172.85, 171.2, 168.1, 165.3, 156.7, 147.9, 137.2, 135.9, 135.3, 133.3 – 131.5 (m), 130.6, 130.0, 127.8, 125.6 – 116.2 (m), 78.3, 57.0 (d, *J* = 2.8 Hz), 53.1, 49.0, 40.9, 40.1, 32.2, 30.7, 30.1, 28.7, 27.0 (d, *J* = 6.8 Hz), 23.9 ppm.

^{19}F NMR (471 MHz, $(CD_3)_2CO$) δ = -57.95, -62.90 ppm.

IR (neat): 3664, 3423, 3352, 2986, 2935, 2867, 1691, 1544, 1458, 1369, 1250, 1190, 1162, 1100, 1051, 849, 754, 559 cm^{-1}.

HRMS (ESI): *m/z* calcd. for $C_{31}H_{36}F_6N_8O_6Na$ $[M+Na]^+$ 753.2554, found 753.2548.

Synthesis of 65

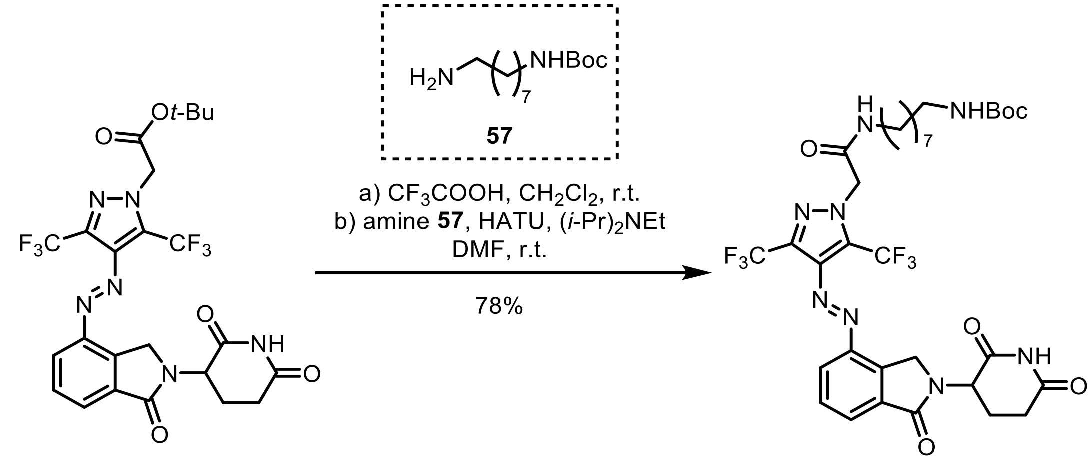

$(CF_3)_2$-arylazopyrazole photoswitch

65

$(CF_3)_2$-arylazopyrazole photoswitch (30 mg, 0.51 mmol, 1.0 equiv) was dissolved in CH_2Cl_2 (0.25 mL) and CF_3COOH (0.25 mL) was added. The resulting reaction mixture was stirred at r.t. for 2 h after which it was diluted with toluene and concentrated *in vacuo*. The crude product was used in the next step without further purification.

The crude product, amine **57** (13.6 mg, 55.8 µmol, 1.1 equiv) and HATU (20.2 mg, 53.3 µmol, 1.05 equiv) were dissolved in anhydrous DMF (0.5 mL) and (*i*-Pr)$_2$NEt (26 µL, 0.15 mmol, 3.00 equiv) was added. The resulting reaction mixture was stirred overnight after which it was concentrated *in vacuo.* Column chromatography (SiO_2, eluent: hexanes–EtOAc (1:2) to EtOAc) afforded **65** (29.9 mg, 78%) as a yellow solid.

^{1}H NMR (500 MHz, $(CD_3)_2CO$): δ = 9.88 – 9.78 (m, 1H), 8.21 (dd, *J* = 7.8, 1.1 Hz, 1H), 8.00 (dd, *J* = 7.5, 1.0 Hz, 1H), 7.87 – 7.82 (m, 1H), 7.62 (t, *J* = 5.8 Hz, 1H), 5.89 (s, 1H), 5.32 (d, *J* = 1.3 Hz, 2H), 5.24 (dd, *J* = 13.4, 5.1 Hz, 1H), 4.83 – 4.69 (m, 2H), 3.28 (td, *J* = 7.0, 5.7 Hz, 2H), 3.08 – 2.99 (m, 3H), 2.82 (ddd, *J* = 17.5, 4.5, 2.4 Hz, 1H), 2.57 (qd, *J* = 13.3, 4.5 Hz, 1H), 2.30 (dtd, *J* = 12.8, 5.3, 2.4 Hz, 1H), 1.60 – 1.43 (m, 4H), 1.39 (s, 9H), 1.36 – 1.26 (m, 8H) ppm.

^{13}C NMR (126 MHz, $(CD_3)_2CO$): δ = 172.7, 171.2, 168.0, 165.3, 156.7, 147.9, 137.2, 135.9, 135.4, 133.1 – 131.6 (m), 130.6, 130.0, 127.8, 125.1 – 117.8 (m), 78.3, 57.0 (d, *J* = 2.7 Hz), 53.1, 49.0, 41.1, 40.2, 32.2, 30.8, 30.0, 29.9, 28.7, 27.4 (d, *J* = 1.7 Hz), 24.0 ppm.

^{19}F NMR (471 MHz, $(CD_3)_2CO$) δ = -57.95, -62.90 ppm.

IR (neat): 3321, 3100, 2983, 2931, 2858, 1680, 1700, 1556, 1458, 1367, 1250, 1164, 1148, 1101, 1051, 850, 754 cm^{-1}.

HRMS (ESI): *m/z* calcd. for $C_{33}H_{40}F_6N_8O_6Na$ $[M+Na]^+$ 781.2867, found 781.2861.

Synthesis of 66

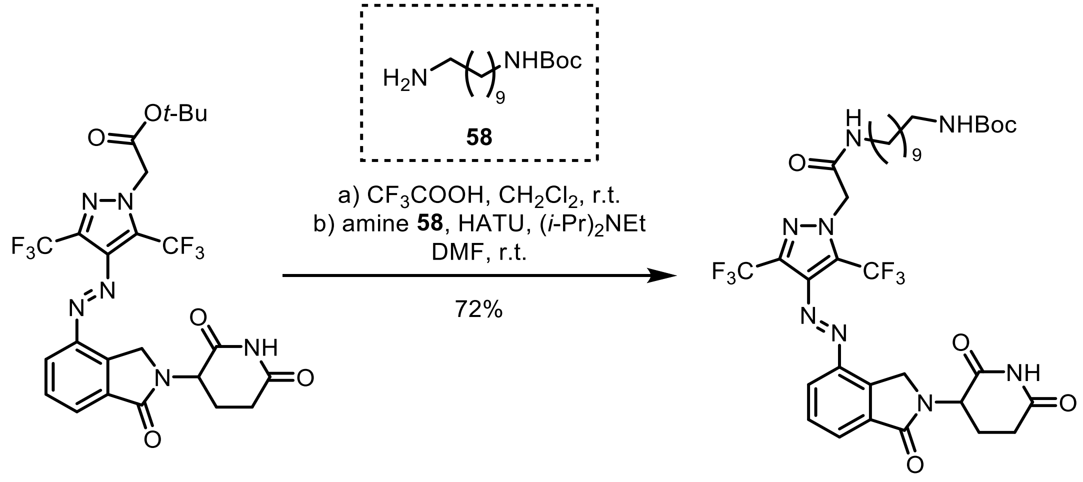

$(CF_3)_2$-arylazopyrazole photoswitch

66

$(CF_3)_2$-arylazopyrazole photoswitch (30 mg, 0.51 mmol, 1.0 equiv) was dissolved in CH_2Cl_2 (0.25 mL) and CF_3COOH (0.25 mL) was added. The resulting reaction mixture was stirred at r.t. for 2 h after which it was diluted with toluene and concentrated *in vacuo*. The crude product was used in the next step without further purification.

The crude product, amine **58** (15.2 mg, 55.8 µmol, 1.1 equiv) and HATU (20.2 mg, 53.3 µmol, 1.05 equiv) were dissolved in anhydrous DMF (0.5 mL) and (*i*-Pr)$_2$NEt (26 µL, 0.15 mmol, 3.00 equiv) was added. The resulting reaction mixture was stirred overnight after which it was concentrated *in vacuo.* Column chromatography (SiO_2, eluent: hexanes–EtOAc (1:2) to EtOAc) afforded **66** (28.8 mg, 72%) as a yellow solid.

^{1}H NMR (500 MHz, $(CD_3)_2CO$): δ = 9.82 (s, 1H), 8.21 (dd, *J* = 7.8, 1.1 Hz, 1H), 8.00 (dd, *J* = 7.6, 1.1 Hz, 1H), 7.85 (t, *J* = 7.7 Hz, 1H), 7.61 (t, *J* = 5.7 Hz, 1H), 5.89 (s, 1H), 5.32 (d, *J* = 1.4 Hz, 2H), 5.24 (dd, *J* = 13.3, 5.1 Hz, 1H), 4.89 – 4.65 (m, 2H), 3.33 – 3.24 (m, 2H), 3.09 – 2.96 (m, 3H), 2.82 (ddd, *J* = 17.5, 4.5, 2.4 Hz, 1H), 2.57 (qd, *J* = 13.3, 4.5 Hz, 1H), 2.30 (dtd, *J* = 12.9, 5.3, 2.5 Hz, 1H), 1.52 (tp, *J* = 20.7, 7.6 Hz, 4H), 1.39 (s, 9H), 1.31 (d, *J* = 9.0 Hz, 12H) ppm.

^{13}C NMR (126 MHz, $(CD_3)_2CO$): δ = 172.7, 171.2, 168.0, 165.3, 156.7, 147.9, 137.2, 135.9, 135.4, 132.4 (dd, *J* = 40.0, 33.3 Hz), 130.6, 130.0, 127.8, 126.4 – 116.3 (m), 78.3, 57.0, 53.1, 49.0, 41.1, 40.3, 32.2, 30.8, 30.2, 30.2, 30.2, 30.0, 30.0, 29.4, 28.7, 27.5, 23.9 ppm.

^{19}F NMR (471 MHz, $(CD_3)_2CO$) δ = -57.95, -62.90 ppm.

IR (neat): 3320, 3190, 3098, 2928, 2855, 1717, 1667, 1555, 1496, 1476, 1453, 1384, 1367, 1283, 1250, 1188, 1164, 1147, 1102, 1052, 849, 755 cm^{-1}.

HRMS (ESI): *m/z* calcd. for $C_{35}H_{44}F_6N_8O_6Na$ $[M+Na]^+$ 809.3180, found 809.3180.

Synthesis of KH-5-327

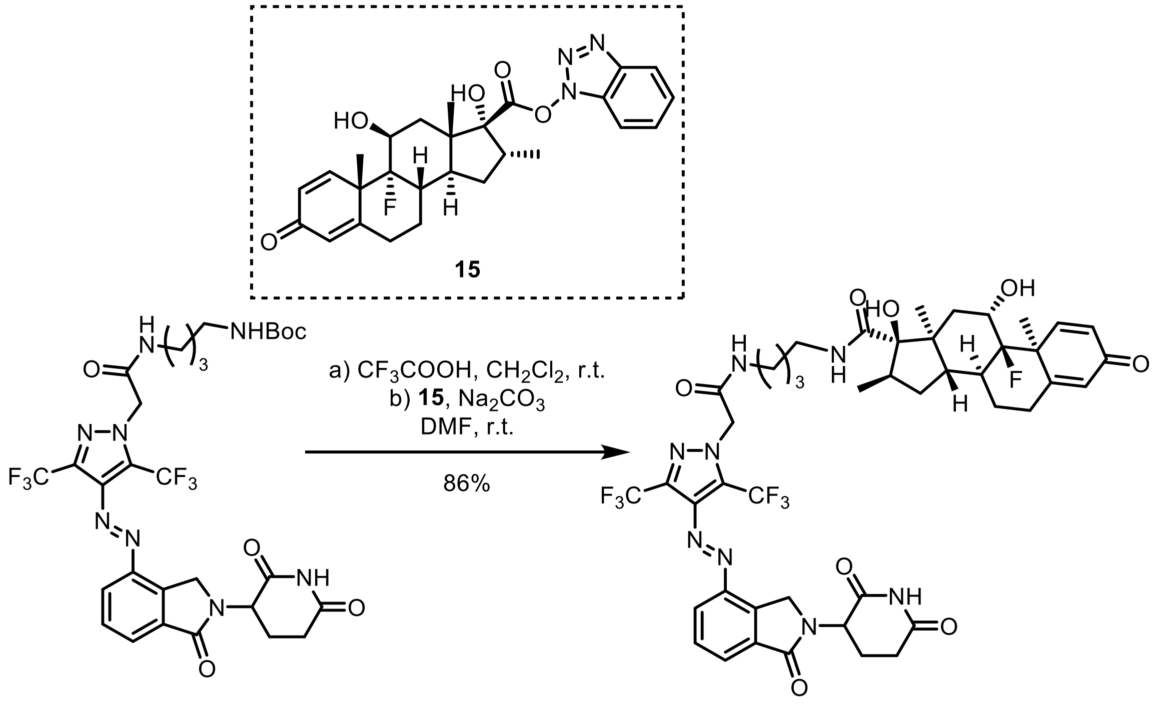

63 **KH-5-327**

63 (16.9 mg, 24.1 µmol, 1.00 equiv) was dissolved in CH_2Cl_2 (0.13 mL) and CF_3COOH (0.13 mL) was added. The resulting reaction mixture was stirred at r.t. for 2 h after which it was diluted with toluene and concentrated *in vacuo*. The crude product was used in the next step without further purification.

The crude product, **15** (14 mg, 29 µmol, 1.2 equiv) and Na_2CO_3 (7.6 mg, 72 µmol, 3.0 equiv) were dissolved in anhydrous DMF (0.25 mL). The resulting reaction mixture was stirred for 24 h at r.t., after which it was concentrated *in vacuo.* Column chromatography (SiO_2, eluent: EtOAc to EtOAc–acetone (1:1)) and C_{18}-reversed phase column chromatography (eluent: MeCN–H_2O (1:1) to MeCN–H_2O (7:3)) afforded **KH-5-327** (19.9 mg, 86%) as a yellow foam.

^{1}H NMR (500 MHz, $(CD_3)_2SO$): δ = 11.04 (s, 1H), 8.49 – 8.43 (m, 1H), 8.15 (dt, *J* = 7.8, 1.3 Hz, 1H), 8.00 (dt, *J* = 7.6, 1.2 Hz, 1H), 7.84 (td, *J* = 7.7, 1.8 Hz, 1H), 7.37 (t, *J* = 6.0 Hz, 1H), 7.25 (dd, *J* = 10.1, 1.6 Hz, 1H), 6.18 (ddd, *J* = 10.1, 2.6, 1.9 Hz, 1H), 5.98 (s, 1H), 5.25 (s, 2H), 5.21 – 5.08 (m, 2H), 4.72 – 4.51 (m, 3H), 4.09 (d, *J* = 11.3 Hz, 1H), 3.21 – 3.09 (m, 3H), 3.08 – 2.99 (m, 2H), 2.93 (ddd, *J* = 17.9, 13.3, 5.3 Hz, 1H), 2.69 – 2.55 (m, 2H), 2.31 (td, *J* = 15.8, 13.0, 9.6 Hz, 3H), 2.14 – 1.99 (m, 3H), 1.81 – 1.71 (m, 1H), 1.67 – 1.55 (m, 1H), 1.48 – 1.28 (m, 9H), 1.05 (ddd, *J* = 12.2, 8.2, 4.3 Hz, 1H), 0.94 (s, 3H), 0.79 (d, *J* = 7.2 Hz, 3H) ppm.

^{13}C NMR (126 MHz, $(CD_3)_2SO$): δ = 185.3, 172.8, 172.2, 170.9, 167.2, 166.9, 164.6, 152.8, 146.4, 135.7, 134.7, 134.0, 131.6 – 130.3 (m), 130.1, 129.0, 128.8, 127.1, 124.1, 123.7 – 115.3 (m), 102.2, 100.8, 86.4, 71.0 (d, *J* = 37.3 Hz), 56.1, 51.9, 48.0 (d, *J* = 22.8 Hz), 47.9 (d, *J* = 3.2 Hz), 47.4, 43.0, 38.8, 38.2, 35.5, 34.4, 33.8 (d, *J* = 19.3 Hz), 32.0, 31.2, 30.3, 27.3, 26.7, 26.3, 22.8 (d, *J* = 5.7 Hz), 22.6, 17.0, 15.1 ppm.

^{19}**F NMR** (377 MHz, $(CD_3)_2SO$): δ = -56.73, -61.36, -164.14 ppm.

IR (neat): 3417, 3093. 2937, 2872, 1689, 1663, 1529, 1500, 1456, 1432, 1416, 1366, 1299, 1276, 1184, 1150, 1100, 1051, 1031, 894, 752 cm^{-1}.

HRMS (ESI): *m/z* calcd. for $C_{45}H_{49}F_7N_8O_8Na$ $[M+Na]^+$ 985.3454, found 985.3435.

Synthesis of KH-5-331

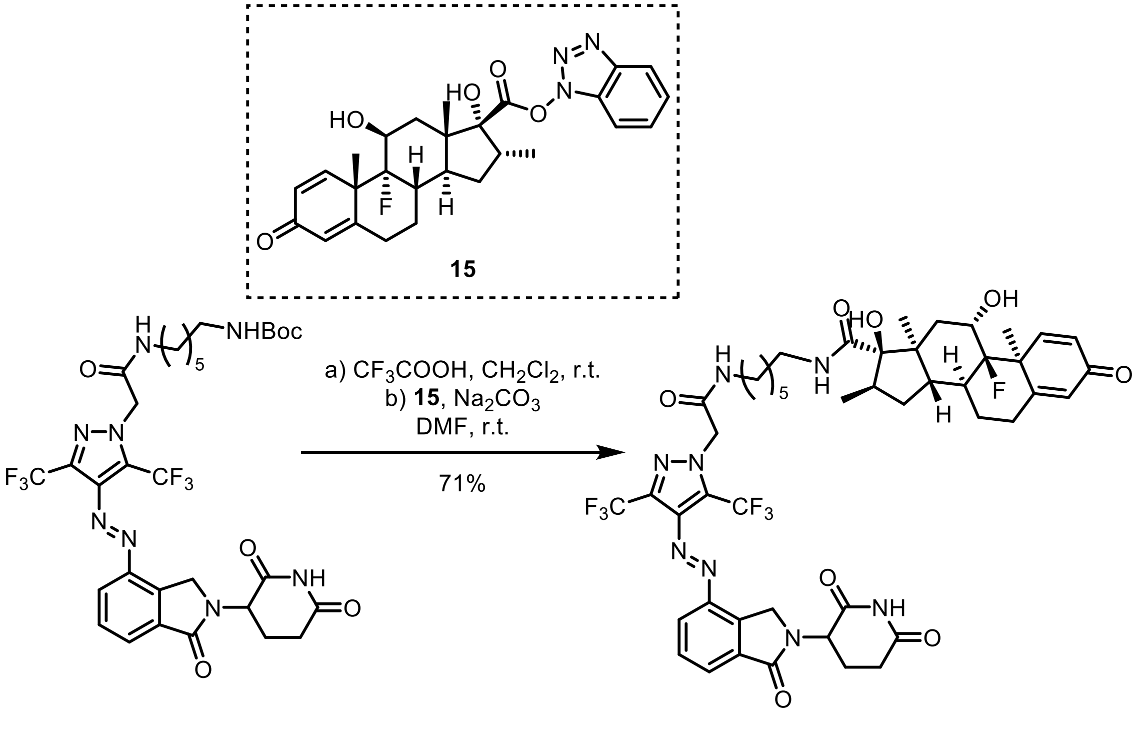

64 (23.8 mg, 32.6 µmol, 1.0 equiv) was dissolved in CH_2Cl_2 (0.18 mL) and CF_3COOH (0.18 mL) was added. The resulting reaction mixture was stirred at r.t. for 2 h after which it was diluted with toluene and concentrated *in vacuo*. The crude product was used in the next step without further purification.

The crude product, **15** (19. mg, 38.8 µmol, 1.2 equiv) and Na_2CO_3 (10.3 mg, 97.1 µmol, 3.0 equiv) were dissolved in anhydrous DMF (0.32 mL). The resulting reaction mixture was stirred for 24 h at r.t., after which it was concentrated *in vacuo.* Column chromatography (SiO_2, eluent: EtOAc to EtOAc–acetone (1:1)) and C_{18}-reversed phase column chromatography (eluent: MeCN–H_2O (1:1) to MeCN–H_2O (2:1)) afforded **KH-5-331** (22.9 mg, 71%) as a yellow foam.

^{1}H NMR (500 MHz, $(CD_3)_2SO$): δ = 11.04 (s, 1H), 8.41 (t, *J* = 5.7 Hz, 1H), 8.15 (dd, *J* = 7.9, 1.1 Hz, 1H), 8.01 (dd, *J* = 7.6, 1.1 Hz, 1H), 7.84 (t, *J* = 7.7 Hz, 1H), 7.34 (t, *J* = 5.9 Hz, 1H), 7.28 (dd, *J* = 10.1, 1.0 Hz, 1H), 6.20 (ddd, *J* = 10.1, 1.9, 0.8 Hz, 1H), 5.99 (d, *J* = 1.7 Hz, 1H), 5.28 – 5.21 (m, 3H), 5.17 (dd, *J* = 13.3, 5.1 Hz, 1H), 4.74 – 4.51 (m, 3H), 4.15 – 4.05 (m, 1H), 3.14 (dq, *J* = 13.4, 6.8 Hz, 3H), 3.07 – 2.97 (m, 2H), 2.93 (ddd, *J* = 17.2, 13.6, 5.5 Hz, 1H), 2.70 – 2.55 (m, 2H), 2.32 (ddt, *J* = 17.7, 11.1, 5.9 Hz, 3H), 2.15 – 1.99 (m, 3H), 1.80 – 1.71 (m, 1H), 1.60 (q, *J* = 11.7 Hz, 1H), 1.47 (d, *J* = 1.0 Hz, 3H), 1.47 – 1.34 (m, 6H), 1.34 – 1.21 (m, 4H), 1.05 (ddd, *J* = 12.3, 8.3, 4.3 Hz, 1H), 0.94 (s, 3H), 0.79 (d, *J* = 7.2 Hz, 3H) ppm.

^{13}C NMR (126 MHz, $(CD_3)_2SO$): δ = 185.3, 172.8, 172.1, 170.9, 167.2, 166.9, 164.5, 152.9, 146.4, 135.7, 134.7, 134.0, 130.7 (qd, *J* = 39.4, 29.0 Hz), 130.1, 129.0, 128.7, 127.1, 124.1, 123.8 – 113.8 (m), 102.2, 100.9, 86.3, 70.9 (d, *J* = 37.1 Hz), 56.1, 51.9, 48.0 (d, *J* = 22.8 Hz), 47.9, 47.4, 43.0,

38.8, 38.6, 35.5, 34.4, 33.8 (d, J = 19.3 Hz), 32.0, 31.2, 30.3, 29.3, 28.8, 27.3, 26.0, 26.0, 22.9 (d, J = 5.7 Hz), 22.6, 16.9, 15.1 ppm.

^{19}F NMR (471 MHz, $(CD_3)_2SO$): δ = -56.74, -61.36, -164.05 ppm.

IR (neat): 3384, 3096, 2935, 2875, 1690, 1663, 1529, 1504, 1456, 1370, 1276, 1248, 1183, 1149, 1100, 1051, 1035, 894, 821, 753 cm^{-1}.

HRMS (ESI): *m/z* calcd. for $C_{47}H_{53}F_7N_8O_8Na$ $[M+Na]^+$ 1013.3767, found 1013.3783.

Synthesis of KH-5-336

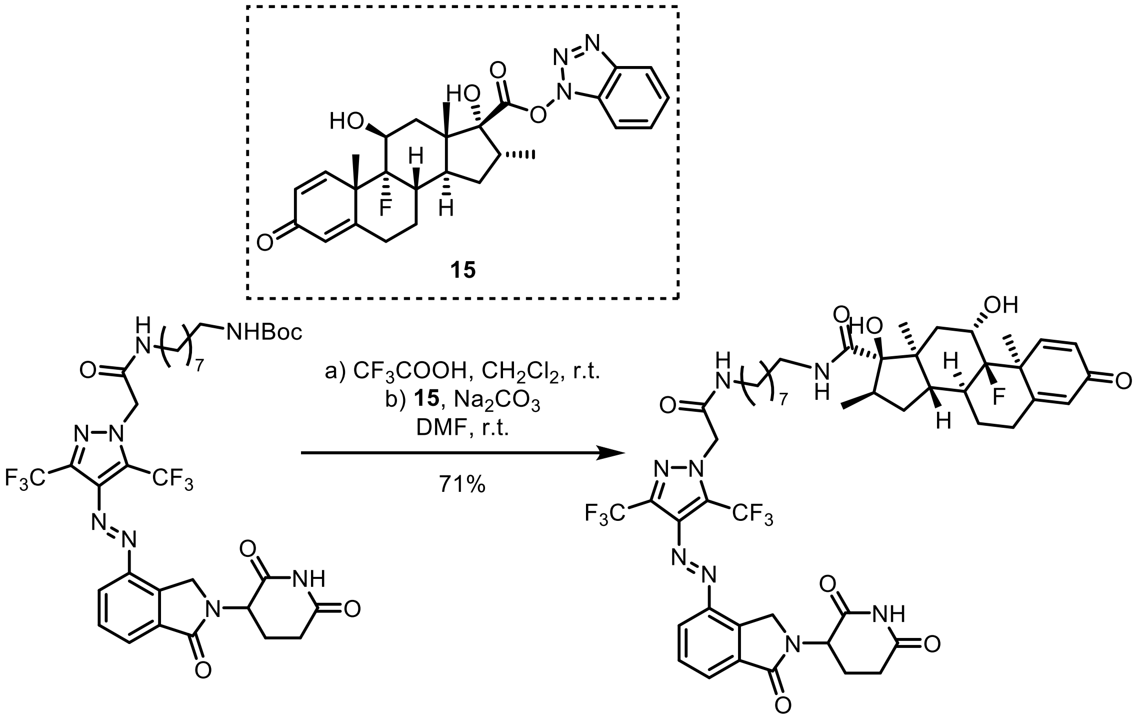

65 **KH-5-336**

65 (29.9 mg, 39.4 µmol, 1.0 equiv) was dissolved in CH_2Cl_2 (0.23 mL) and CF_3COOH (0.23 mL) was added. The resulting reaction mixture was stirred at r.t. for 2 h after which it was diluted with toluene and concentrated *in vacuo*. The crude product was used in the next step without further purification.

The crude product, **15** (23.4 mg, 47.2 µmol, 1.2 equiv) and Na_2CO_3 (12.5 mg, 118 µmol, 3.0 equiv) were dissolved in anhydrous DMF (0.39 mL). The resulting reaction mixture was stirred for 24 h at r.t., after which it was concentrated *in vacuo.* Column chromatography (SiO_2, eluent: EtOAc to EtOAc –acetone (1:1)) and C_{18}-reversed phase column chromatography (eluent: MeCN–H_2O (1:1) to MeCN–H_2O (2:1)) afforded **KH-5-336** (28.5 mg, 71%) as a yellow foam.

^{1}H NMR (500 MHz, $(CD_3)_2SO$): δ = 11.04 (s, 1H), 8.39 (t, *J* = 5.6 Hz, 1H), 8.19 – 8.12 (m, 1H), 8.04 – 7.96 (m, 1H), 7.84 (td, *J* = 7.7, 4.3 Hz, 1H), 7.37 – 7.24 (m, 2H), 6.21 (dt, *J* = 10.0, 2.2 Hz, 1H), 5.99 (t, *J* = 1.8 Hz, 1H), 5.24 (q, *J* = 2.0 Hz, 3H), 5.17 (dd, *J* = 13.3, 5.1 Hz, 1H), 4.71 – 4.52 (m, 3H), 4.14 – 4.07 (m, 1H), 3.11 (q, *J* = 6.7 Hz, 3H), 3.07 – 2.95 (m, 2H), 2.96 – 2.87 (m, 1H), 2.68 – 2.55 (m, 2H), 2.32 (dtd, *J* = 13.1, 8.6, 4.4 Hz, 3H), 2.14 – 1.97 (m, 3H), 1.82 – 1.72 (m, 1H), 1.60 (q, *J* = 11.6 Hz, 1H), 1.48 (d, *J* = 2.8 Hz, 3H), 1.46 – 1.31 (m, 6H), 1.26 (s, 9H), 1.05 (ddd, *J* = 12.1, 8.1, 4.2 Hz, 1H), 0.94 (s, 3H), 0.78 (d, *J* = 7.2 Hz, 3H) ppm.

^{13}C NMR (126 MHz, $(CD_3)_2SO$): δ = 185.3, 172.8, 172.1, 170.9, 167.2, 166.9, 164.4, 152.9, 146.4, 135.7, 134.7, 134.0, 131.4 – 130.2 (m), 130.1, 129.0, 128.7, 127.0, 124.1, 119.6 (qd, *J* = 271.7, 270.3, 163.6 Hz), 102.3, 100.9, 86.3, 70.9 (d, *J* = 37.0 Hz), 56.1, 51.9, 48.1 (d, *J* = 22.8 Hz), 47.9, 47.4, 43.0, 38.9, 38.7, 35.5, 34.4, 33.8 (d, *J* = 19.3 Hz), 32.0, 31.2, 30.3, 29.4, 28.9, 28.7, 28.7, 27.3, 26.4, 26.2, 22.9 (d, *J* = 5.7 Hz), 22.6, 16.9, 15.1 ppm.

^{19}F NMR (471 MHz, $(CD_3)_2SO$): δ = -56.74, -61.36, -164.04 ppm.

IR (neat): 3417, 3102, 2934, 2859, 1690, 1663, 1529, 1498, 1456, 1432, 1416, 1370, 1335, 1298, 1248, 1184, 1150, 1100, 1051, 1035, 894, 753 cm^{-1}.

HRMS (ESI): *m/z* calcd. for $C_{49}H_{58}F_7N_8O_8$ $[M+H]^+$ 1019.4260, found 1019.4243.

Synthesis of KH-5-340

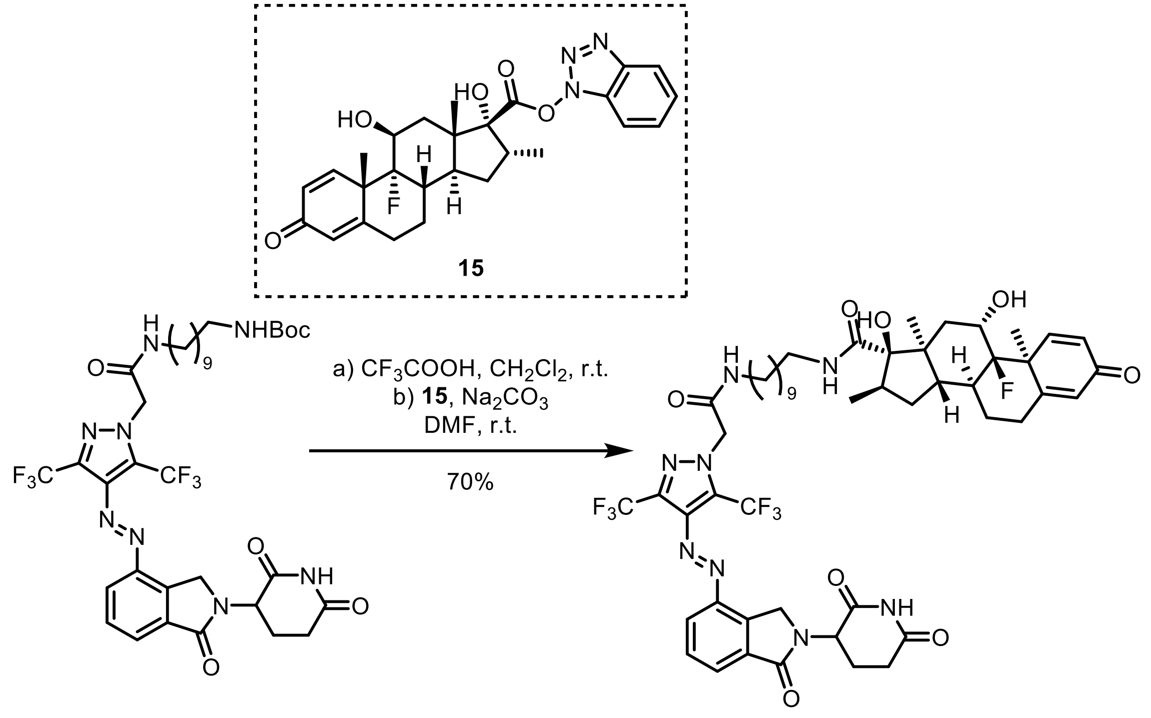

66 (28.8 mg, 36.6 µmol, 1.0 equiv) was dissolved in CH_2Cl_2 (0.15 mL) and CF_3COOH (0.15 mL) was added. The resulting reaction mixture was stirred at r.t. for 2 h after which it was diluted with toluene and concentrated *in vacuo*. The crude product was used in the next step without further purification.

The crude product, **15** (21.7 mg, 43.7 µmol, 1.20 equiv) and Na_2CO_3 (11.6 mg, 109 µmol, 3.00 equiv) were dissolved in anhydrous DMF (0.36 mL). The resulting reaction mixture was stirred for 24 h at r.t., after which it was concentrated *in vacuo.* Column chromatography (SiO_2, eluent: EtOAc to EtOAc –acetone (1:1)) and C_{18}-reversed phase column chromatography (eluent: MeCN–H_2O (1:1) to MeCN–H_2O (2:1)) afforded **KH-5-340** (26.5 mg, 70%) as a yellow solid.

^{1}H NMR (500 MHz, $(CD_3)_2SO$): δ = 11.04 (s, 1H), 8.38 (t, *J* = 5.6 Hz, 1H), 8.15 (dt, *J* = 7.9, 1.1 Hz, 1H), 8.01 (dd, *J* = 7.6, 1.1 Hz, 1H), 7.84 (t, *J* = 7.6 Hz, 1H), 7.38 – 7.25 (m, 2H), 6.21 (dd, *J* = 10.1, 1.9 Hz, 1H), 5.99 (d, *J* = 1.7 Hz, 1H), 5.24 (q, *J* = 1.9 Hz, 3H), 5.17 (dd, *J* = 13.3, 5.1 Hz, 1H), 4.73 – 4.50 (m, 3H), 4.14 – 4.07 (m, 1H), 3.19 – 3.06 (m, 3H), 2.99 (ddd, *J* = 14.2, 11.4, 6.2 Hz, 2H), 2.95 – 2.87 (m, 1H), 2.69 – 2.55 (m, 2H), 2.41 – 2.25 (m, 3H), 2.15 – 1.98 (m, 3H), 1.83 – 1.71 (m, 1H), 1.59 (q, *J* = 11.7 Hz, 1H), 1.48 (s, 3H), 1.39 (tdd, *J* = 19.6, 13.4, 7.8 Hz, 6H), 1.25 (s, 12H), 1.04 (ddd, *J* = 12.2, 8.2, 4.2 Hz, 1H), 0.94 (s, 3H), 0.78 (d, *J* = 7.2 Hz, 3H) ppm.

^{13}C NMR (126 MHz, $(CD_3)_2SO$): δ = 185.3, 172.8, 172.1, 170.9, 167.2, 166.9, 164.4, 152.9, 146.4, 135.7, 134.7, 134.0, 130.7 (qd, *J* = 39.6, 31.7 Hz), 130.1, 129.0, 128.7, 127.0, 124.1, 123.8 – 115.3 (m), 102.3, 100.9, 86.3, 70.9 (d, *J* = 37.0 Hz), 56.1, 51.9, 48.1 (d, *J* = 22.8 Hz), 47.9, 47.4, 43.0,

38.9, 38.7, 35.5, 34.4, 33.8 (d, J = 19.3 Hz), 32.0, 31.2, 30.3, 29.4, 29.0, 28.9, 28.8, 28.7, 27.3, 26.4, 26.3, 22.9 (d, J = 5.7 Hz), 22.6, 16.9, 15.1 ppm.

^{19}F NMR (471 MHz, $(CD_3)_2SO$): δ = -56.75, -61.37, -164.04 ppm.

IR (neat): 3422, 3099, 2931, 2857, 1691, 1664, 1529, 1504, 1456, 1436, 1371, 1354, 1248, 1185, 1152, 1100, 1051, 894, 820, 752 cm^{-1}.

HRMS (ESI): *m/z* calcd. for $C_{51}H_{62}F_7N_8O_8$ $[M+H]^+$ 1047.4573, found 1047.4549.

GR $(OEt)_2$-Arylazopyrazole photoPROTACs

Synthesis of 51

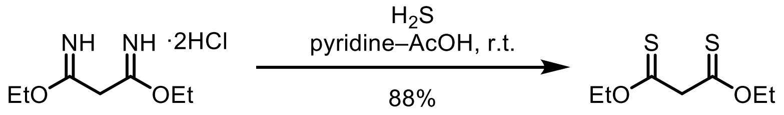

50 **51**

The three-necked reaction flask was charged with **50** (15 g, 0.65 mol, 1.0 equiv) and AcOH (88 mL) and pyridine (9.5 g, 9.7 mL, 0.12 mol, 1.9 equiv) were added. On one side, the reaction flask was connected to a gas washing bottle connected to a quenching solution containing a sat. aq. solution of $CuSO_4·5H_2O$ (1.6 L, 506 g, 1.30 mol, 20.0 equiv). On the other side, the reaction flask was connected to a $CaSO_4$ drying tube that was connected to a three-necked flask equipped with a dropping funnel and an outlet that could be regulated to link the flask to a nitrogen flow or a gas washing bottle and another quenching solution of sat. aq $CuSO_4·5H_2O$ (200 mL, 65 g, 0.17 mol, 2.6 equiv). This three-necked flask was charged with finely crushed FeS (57.1 g, 649 mmol, 10 equiv) and the dropping funnel was filled with aq. HCl (15%, 263 mL, 1.3 mol, 4.94 M, 20 equiv). After the setup was carefully checked, HCl was slowly added to FeS to have a steady H_2S flow. The *in situ* generated H_2S was bubbled through the reaction solution for 2 h at r.t. After 2h, the H_2S flow ceased and all outlets from the reaction flask were closed. The H_2S-saturated reaction solution was stirred for 24 h. An aliquot was aalayzed by ^{1}H NMR showing a mixture of monothiolated and hydrolyzed monothiolated starting material as well as the desired dithioester **51.** Thus, all quenching solutions were exchanged for fresh ones and the three-necked flask and dropping funnel was refilled with equal amounts of FeS and HCl. Again, H_2S was bubbled to the reaction solution for 2 h at r.t. After the addition, all valves were closed and the H_2S-saturated reaction solution was stirred for 24 h at r.t. 100 mL of ether was added and the diluted reaction solution was added to 1 L of water. The aqueous layer was extracted with ether (2 x 100 mL, 2 x 50 mL), all combined organic extracts were washed with water and brine, dried over Na_2SO_4, and was concentrated *in vacuo.* Column chromatography (SiO_2, eluent: pentane to ether–pentane (1:2)) afforded **51** (11 g, 88%) as a yellow oil.

^{1}H NMR (400 MHz, ($CDCl_3$): δ = 4.54 (qd, *J* = 7.1, 0.8 Hz, 4H), 4.15 (s, 2H), 1.40 (td, *J* = 7.1, 0.8 Hz, 6H) ppm.

^{13}C NMR (101 MHz, ($CDCl_3$): δ = 213.7, 69.0, 63.5, 13.6 ppm.

IR (neat): 2982, 2956, 2937, 2897, 1462, 1444, 1390, 1366, 1324, 1296, 1236, 1213, 1167, 1093, 1025, 947, 899, 852, 809 cm^{-1}.

HRMS (ESI): *m/z* calcd. for $C_7H_{11}O_2S_2$ $[M+H]^+$ 191.0195, found 191.0195.

Note: Please refer to the figure below for detailed information about the setup:

Description of the setup:
Left: tubing was attached to each outlet leading to the back of the hood (vent), quenching solution and gas washing bottle led to the three-necked flask equipped with a dropping funnel and a three-way cock to further connect the setup to the nitrogen flow of the Schlenk line.
Middle: A drying tube is installed in between the three-necked flask and the reaction vessel. H_2S is bubbled through the reaction solution by a gas bubbler.

Right: The reaction vessel is connected to the quenching solution *via* a gas washing bottle. Tubing is connected to the quenching solution leading to the back of the hood (vent).

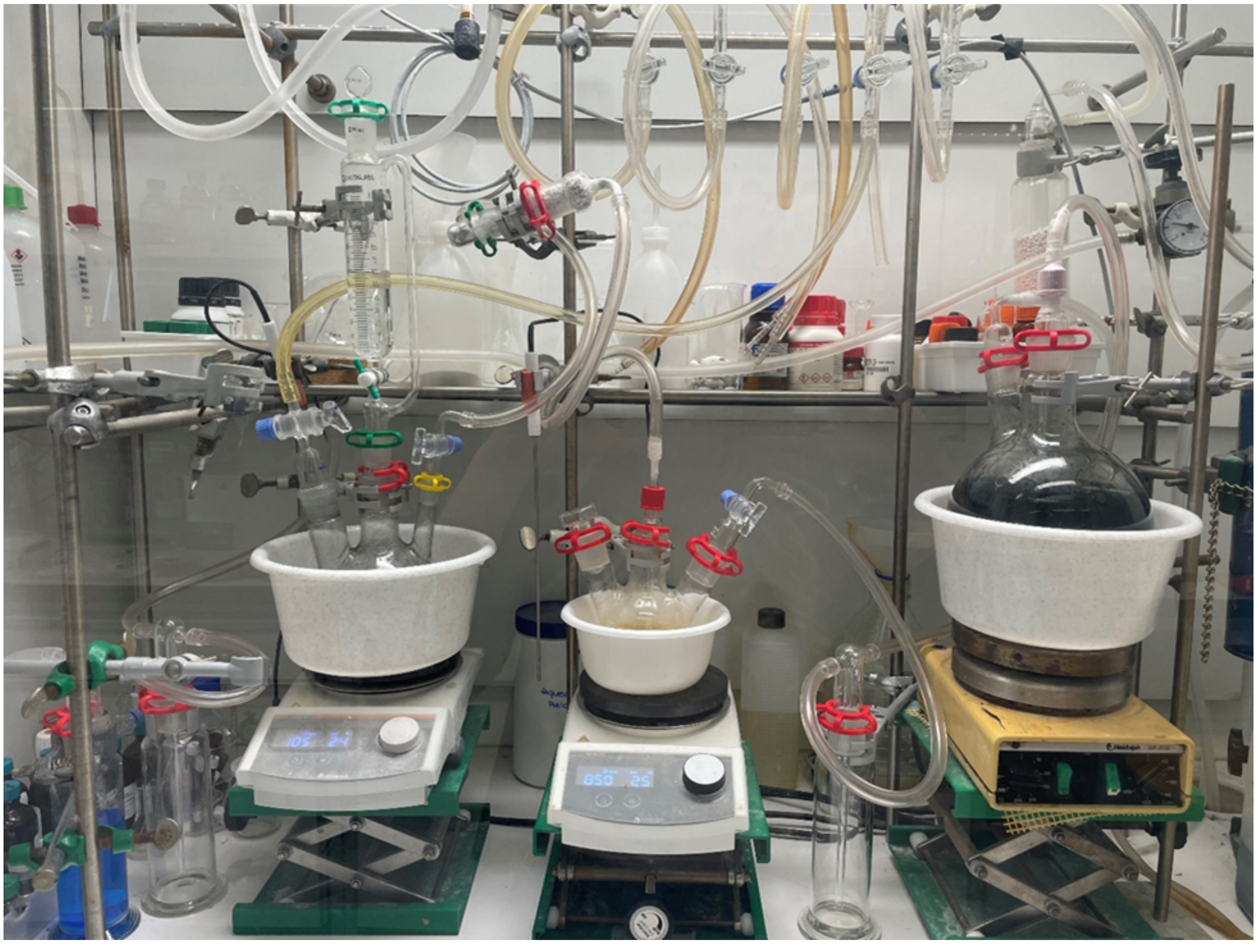

Fig. S78. Setup for the synthesis of **51**.

Synthesis of 52

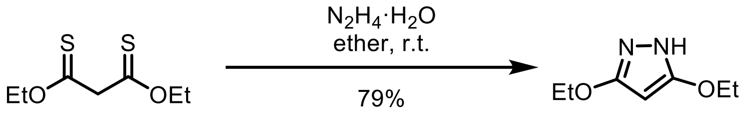

51 (1.0 g, 5.2 mmol, 1.0 equiv) was dissolved in ether (13 mL) and $N_2H_4{\cdot}H_2O$ (0.25 mL, 5.2 mmol, 1.0 equiv) was added slowly at r.t. The resulting reaction mixture was stirred at r.t. for 6 h after which it was diluted and extracted with ether and the combined organic layers were dried over Na_2SO_4 and concentrated *in vacuo* to afford **52** (638 mg, 79%) as a white solid.

^{1}H NMR (400 MHz, ($CDCl_3$): δ = 5.00 (s, 1H), 4.12 (q, *J* = 7.1 Hz, 4H), 1.37 (t, *J* = 7.1 Hz, 6H) ppm.

^{13}C NMR (101 MHz, ($CDCl_3$): δ = 159.7, 71.9, 65.6, 14.8 ppm.

IR (neat): 3192, 3152, 3093, 2981, 2938, 2870, 2753, 1582, 1525, 1497, 1474, 1443, 1386, 1357, 1182, 1152, 1110, 1091, 1040, 999, 899, 763, 730 cm^{-1}.

HRMS (ESI): *m/z* calcd. for $C_7H_{13}O_2N_2$ $[M+H]^+$ 157.0972, found 157.0971.

Synthesis of 54

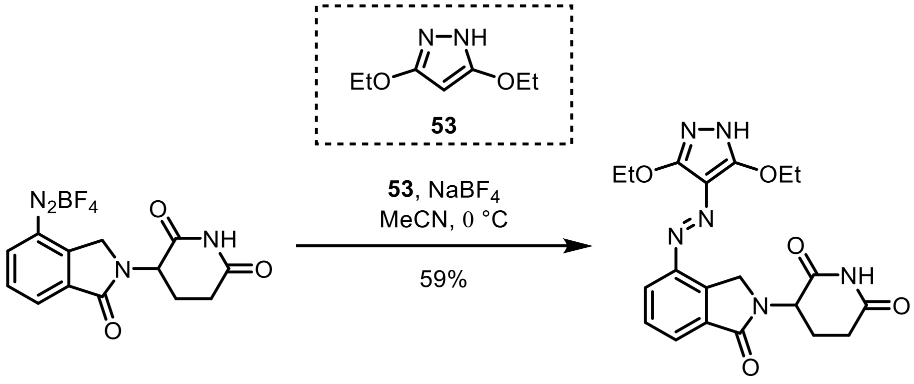

35   54

35 (0.10 g, 0.28 mmol, 1.0 equiv) in MeCN (1.5 mL) was added to a suspension of **53** (43.5 mg, 279 μmol, 1.0 equiv) and $NaBF_4$ (0.31 g, 2.8 mmol, 10 equiv) in MeCN (6.3 mL) at 0 °C. The flask was subsequently washed with MeCN (1 mL). The reaction mixture was stirred for 60 min at 0 °C. EtOAc was added and the mixture was filtered and washed with EtOAc. The filtrate was concentrated under reduced pressure and the obtained solid was transferred to a funnel and washed with EtOAc–acetone (1:1) and acetone to afford **54** (70 mg, 59%) as a red solid.

^{1}H NMR (400 MHz, $(CD_3)_2SO$): δ = 11.04 (s, 1H), 7.86 (d, *J* = 7.6 Hz, 1H), 7.72 (d, *J* = 7.4 Hz, 1H), 7.65 (t, *J* = 7.6 Hz, 1H), 5.16 (dd, *J* = 13.3, 5.1 Hz, 1H), 4.73 – 4.43 (m, 2H), 4.34 (q, *J* = 7.0 Hz, 4H), 2.94 (ddd, *J* = 18.0, 13.5, 5.4 Hz, 1H), 2.62 (d, *J* = 17.7 Hz, 1H), 2.32 (qd, *J* = 13.2, 4.3 Hz, 1H), 2.07 (d, *J* = 3.6 Hz, 1H), 1.36 (t, *J* = 7.0 Hz, 6H) ppm.

^{13}C NMR (101 MHz, $(CD_3)_2SO$): δ = 172.9, 171.1, 167.6, 148.2, 133.5 (d, *J* = 22.8 Hz), 129.3, 125.5, 122.7, 111.9, 51.7, 47.8, 31.2, 22.8, 14.7 ppm.

IR (neat): 3103, 1709, 1669, 1545, 1490, 1403, 1333, 1303, 1261, 1063, 1023, 754, 550 cm^{-1}.

HRMS (ESI): *m/z* calcd. for $C_{20}H_{23}N_6O_5$ $[M+H]^+$ 427.1724, found 427.1719.

Synthesis of $(OEt)_2$-arylazopyrazole photoswitch

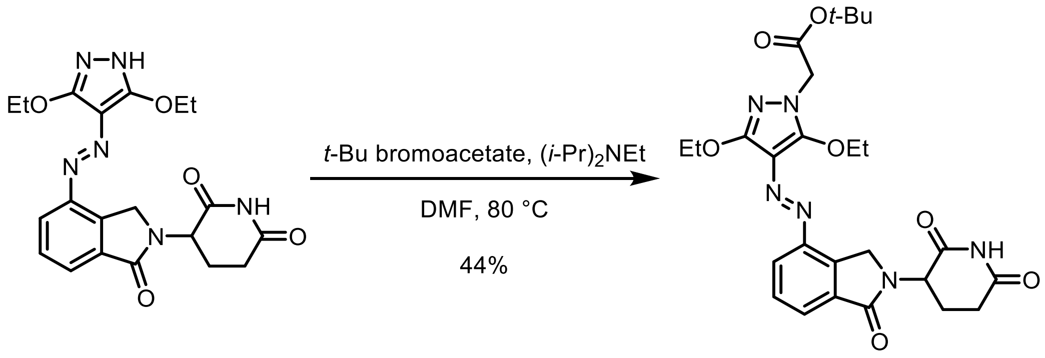

54 **$(OEt)_2$-arylazopyrazole photoswitch**

t-Bu bromoacetate (0.12 mL, 0.83 mmol, 1.0 equiv) was added in portions to a suspension of **54** (350 mg, 821 µmol, 1.00 equi) in DMF (8.4 mL) and (i-Pr)$_2$NEt (0.35 mL, 2.0 mmol, 2.5 equiv) over the course of 12 h at 80 °C. After stirring the reaction mixture for another hour, it was concentrated in *vacuo* and purified by column chromatography (SiO_2, eluent: 40% EtOAc in hexanes to EtOAc–hexanes (2:1)) to afford **$(OEt)_2$-arylazopyrazole photoswitch** (194 mg, 44%) as an orange foam.

^{1}H NMR (400 MHz, $(CD_3)_2CO$): δ = 9.82 (s, 1H), 7.91 (dd, J = 7.9, 1.1 Hz, 1H), 7.76 (dd, J = 7.5, 1.1 Hz, 1H), 7.65 (t, J = 7.7 Hz, 1H), 5.25 (dd, J = 13.3, 5.1 Hz, 1H), 4.81 – 4.68 (m, 4H), 4.62 (s, 2H), 4.25 (q, J = 7.0 Hz, 2H), 3.01 (ddd, J = 17.4, 13.6, 5.4 Hz, 1H), 2.96 – 2.88 (m, 1H), 2.84 – 2.75 (m, 1H), 2.55 (qd, J = 13.3, 3.3 Hz, 1H), 2.27 (ddd, J = 10.0, 5.3, 2.6 Hz, 1H), 1.47 (s, 9H), 1.40 (dt, J = 23.4, 7.0 Hz, 6H) ppm.

^{13}C NMR (101 MHz, $(CD_3)_2CO$): δ = 172.8 (d, J = 2.6 Hz), 171.3, 168.7, 167.3, 154.8, 151.0, 149.2, 135.8, 134.7, 129.9, 125.4, 124.2, 114.9, 82.8, 71.7, 64.3, 52.9, 49.8, 48.4, 32.2, 28.1, 24.0, 15.6, 15.0 ppm.

IR (neat): 3210, 3103, 2979, 2936, 1694, 1552, 1494, 1473, 1413, 1384, 1369, 1355, 1337, 1300, 1265, 1232, 1203, 1155, 1124, 1091, 1042, 1017, 965, 935, 868, 846, 753 cm^{-1}.

HRMS (ESI): *m/z* calcd. for $C_{26}H_{33}N_6O_7$ $[M+H]^+$ 541.2405, found 541.2404.

Synthesis of 67

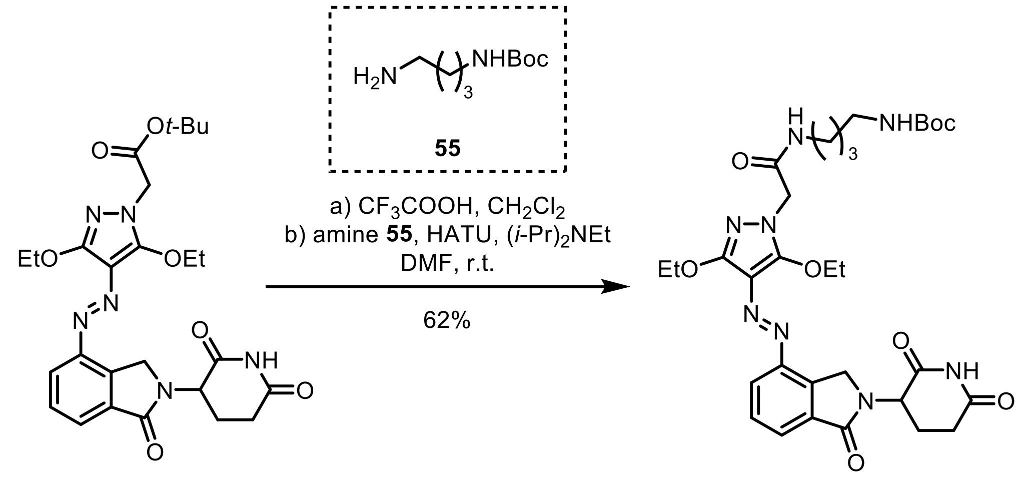

(OEt)$_2$-arylazopyrazole photoswitch **67**

(OEt)$_2$-arylazopyrazole photoswitch (25 mg, 46 µmol, 1.0 equiv) was dissolved in CH_2Cl_2 (0.25 mL) and CF_3COOH (0.25 mL) was added. The resulting reaction mixture was stirred at r.t. for 2 h after which it was diluted with toluene and concentrated *in vacuo*. The crude product was used in the next step without further purification.

The crude product, amine **55** (9.5 mg, 51 µmol, 1.1 equiv) and HATU (18.3 mg, 48.3 µmol, 1.05 equiv) were dissolved in anhydrous DMF (0.46 mL) and (i-Pr)$_2$NEt (30 µL, 0.17 mmol, 3.8 equiv) was added. The resulting reaction mixture was stirred overnight after which it was concentrated *in vacuo.* Column chromatography (SiO_2, eluent: EtOAc) and C_{18}-reversed phase column chromatography (eluent: MeCN–H_2O (1:1)) afforded **67** (28.8 mg, 62%) as a yellow solid.

^{1}H NMR (400 MHz, $(CD_3)_2CO$): δ = 9.83 (s, 1H), 7.92 (dd, J = 7.9, 1.1 Hz, 1H), 7.76 (dd, J = 7.5, 1.1 Hz, 1H), 7.65 (t, J = 7.6 Hz, 1H), 7.32 (t, J = 5.8 Hz, 1H), 5.95 (s, 1H), 5.24 (dd, J = 13.3, 5.1 Hz, 1H), 4.79 – 4.68 (m, 4H), 4.56 (s, 2H), 4.25 (q, J = 7.0 Hz, 2H), 3.30 – 3.22 (m, 2H), 3.12 – 3.04 (m, 2H), 3.04 – 2.96 (m, 1H), 2.79 (ddd, J = 17.5, 4.5, 2.4 Hz, 1H), 2.55 (qd, J = 13.3, 4.5 Hz, 1H), 2.27 (dtd, J = 12.9, 5.3, 2.4 Hz, 1H), 1.52 (tdd, J = 6.6, 4.5, 2.4 Hz, 4H), 1.44 (t, J = 7.0 Hz, 3H), 1.41 – 1.34 (m, 12H) ppm.

^{13}C NMR (101 MHz, $(CD_3)_2CO$): δ = 172.7, 171.3, 168.7, 166.8, 156.7, 155.2, 150.9, 149.2, 135.7, 134.7, 130.0, 125.4, 124.1, 115.0, 78.4, 71.8, 64.4, 52.9, 50.4, 48.4, 40.7, 39.7, 32.2, 28.7, 28.2, 27.6, 24.1, 15.7, 15.0 ppm.

IR (neat): 3322, 3095, 2977, 2934, 1687, 1546, 1496, 1473, 1438, 1407, 1384, 1365, 1336, 1251, 1232, 1202, 1170, 1124, 1040, 1016, 867, 817, 753, 618 cm^{-1}.

HRMS (ESI): *m/z* calcd. for $C_{31}H_{43}N_8O_8$ $[M+H]^+$ 655.3198, found 655.3191.

Synthesis of 68

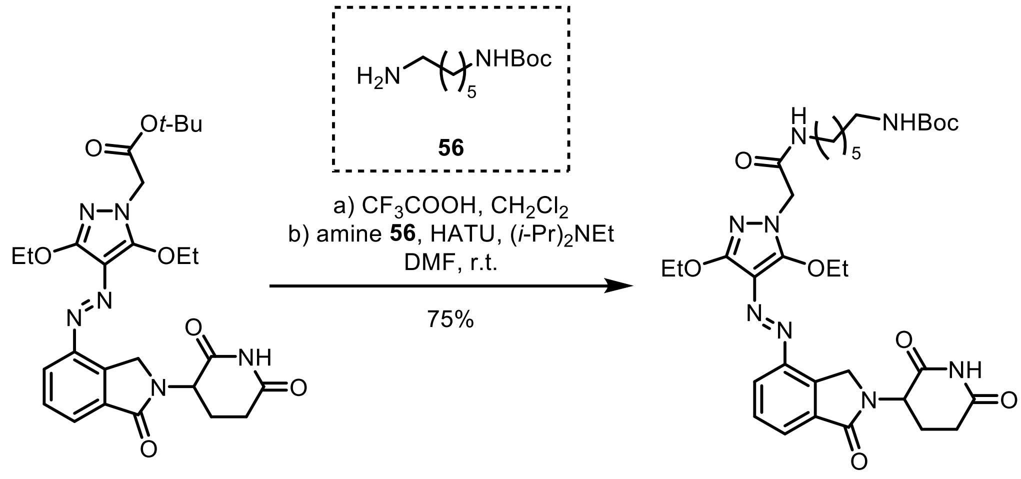

(OEt)$_2$-arylazopyrazole photoswitch **68**

(OEt)$_2$-arylazopyrazole photoswitch (25 mg, 46 µmol, 1.0 equiv) was dissolved in CH_2Cl_2 (0.25 mL) and CF_3COOH (0.25 mL) was added. The resulting reaction mixture was stirred at r.t. for 2 h after which it was diluted with toluene and concentrated *in vacuo*. The crude product was used in the next step without further purification.

The crude product, amine **56** (11 mg, 51 µmol, 1.1 equiv) and HATU (18.3 mg, 48.3 µmol, 1.05 equiv) were dissolved in anhydrous DMF (0.46 mL) and (*I* Pr)$_2$NEt (30 µL, 0.17 mmol, 3.8 equiv) was added. The resulting reaction mixture was stirred overnight after which it was concentrated *in vacuo.* Column chromatography (SiO_2, eluent: EtOAc) and C_{18}-reversed phase column chromatography (eluent: MeCN–H_2O (1:1)) afforded **68** (23.5 mg, 75%) as a yellow solid.

^{1}H NMR (400 MHz, $(CD_3)_2CO$): δ = 9.83 (s, 1H), 7.92 (dd, *J* = 7.9, 1.1 Hz, 1H), 7.76 (dd, *J* = 7.5, 1.1 Hz, 1H), 7.66 (t, *J* = 7.6 Hz, 1H), 7.30 (d, *J* = 6.0 Hz, 1H), 5.91 (s, 1H), 5.24 (dd, *J* = 13.3, 5.2 Hz, 1H), 4.87 – 4.63 (m, 4H), 4.56 (s, 2H), 4.25 (q, *J* = 7.1 Hz, 2H), 3.24 (q, *J* = 6.6 Hz, 2H), 3.14 – 2.94 (m, 3H), 2.83 – 2.75 (m, 1H), 2.55 (qd, *J* = 13.3, 4.5 Hz, 1H), 2.27 (dtd, *J* = 12.9, 5.3, 2.4 Hz, 1H), 1.57 – 1.20 (m, 23H) ppm.

^{13}C NMR (101 MHz, $(CD_3)_2CO$): δ = 172.7, 171.3, 168.7, 166.8, 156.7, 155.2, 150.9, 149.2, 135.7, 134.8, 123.0, 125.4, 124.1, 115.0, 78.3, 71.8, 64.4, 52.9, 50.4, 48.4, 40.9, 39.8, 32.2, 30.8, 28.7, 27.1 (d, *J* = 6.4 Hz), 24.1, 15.7, 15.0 ppm.

IR (neat): 3321, 3095, 2976, 2932, 2859, 1689, 1547, 1497, 1473, 1437, 1408, 1384, 1365, 1355, 1337, 1233, 1203, 1171, 1124, 1040, 1016, 753 cm^{-1}.

HRMS (ESI): *m/z* calcd. for $C_{33}H_{47}N_8O_8$ $[M+H]^+$ 683.3511, found 683.3499.

Synthesis of 69

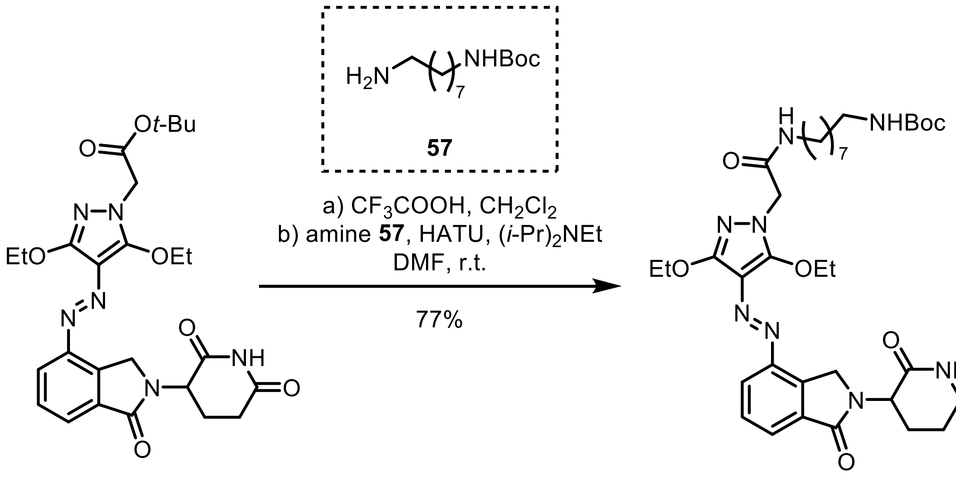

(OEt)$_2$-arylazopyrazole photoswitch 69

(OEt)$_2$-arylazopyrazole photoswitch (25 mg, 46 µmol, 1.0 equiv) was dissolved in CH_2Cl_2 (0.25 mL) and CF_3COOH (0.25 mL) was added. The resulting reaction mixture was stirred at r.t. for 2 h after which it was diluted with toluene and concentrated *in vacuo*. The crude product was used in the next step without further purification.

The crude product, amine **57** (12.4 mg, 50.6 µmol, 1.1 equiv) and HATU (18.3 mg, 48.3 µmol, 1.05 equiv) were dissolved in anhydrous DMF (0.46 mL) and (*i*-Pr)$_2$NEt (30 µL, 0.17 mmol, 3.8 equiv) was added. The resulting reaction mixture was stirred overnight after which it was concentrated *in vacuo.* Column chromatography (SiO_2, eluent: EtOAc) and C_{18}-reversed phase column chromatography (eluent: MeCN–H_2O (1:1)) afforded **69** (25.3 mg, 77%) as a yellow solid.

^{1}H NMR (400 MHz, $(CD_3)_2CO$): δ = 9.83 (s, 1H), 7.92 (dt, *J* = 7.8, 1.2 Hz, 1H), 7.76 (dd, *J* = 7.5, 1.3 Hz, 1H), 7.66 (td, *J* = 7.6, 1.7 Hz, 1H), 7.27 (t, *J* = 5.8 Hz, 1H), 5.89 (s, 1H), 5.25 (ddd, *J* = 13.4, 5.2, 1.7 Hz, 1H), 4.87 – 4.64 (m, 4H), 4.56 (d, *J* = 1.7 Hz, 2H), 4.25 (qd, *J* = 7.2, 1.7 Hz, 2H), 3.24 (q, *J* = 7.1, 6.7 Hz, 2H), 3.09 – 2.96 (m, 3H), 2.82 – 2.75 (m, 1H), 2.55 (qd, *J* = 13.2, 4.4 Hz, 1H), 2.27 (dtd, *J* = 12.8, 5.4, 2.5 Hz, 1H), 1.57 – 1.24 (m, 27H) ppm.

^{13}C NMR (101 MHz, $(CD_3)_2CO$): δ = 172.7, 171.3, 168.69, 166.8, 156.6, 155.2, 150.9, 149.2, 135.7, 134.8, 123.0, 125.4, 124.1, 115.0, 78.3, 71.8, 64.4, 52.9, 50.4, 48.4, 41.1, 40.0, 32.2, 30.8, 30.3, 30.0, 28.7, 27.5 (d, *J* = 3.2 Hz), 24.1, 15.7, 15.0 ppm.

IR (neat): 3329, 3095, 2978, 2930, 2856, 1689, 1546, 1496, 1473, 1406, 1384, 1365, 1355, 1337, 1298, 1249, 1232, 1202, 1170, 1124, 1039, 1016, 664 cm^{-1}.

HRMS (ESI): *m/z* calcd. for $C_{35}H_{51}N_8O_8$ $[M+H]^+$ 711.3824, found 711.3817.

Synthesis of 70

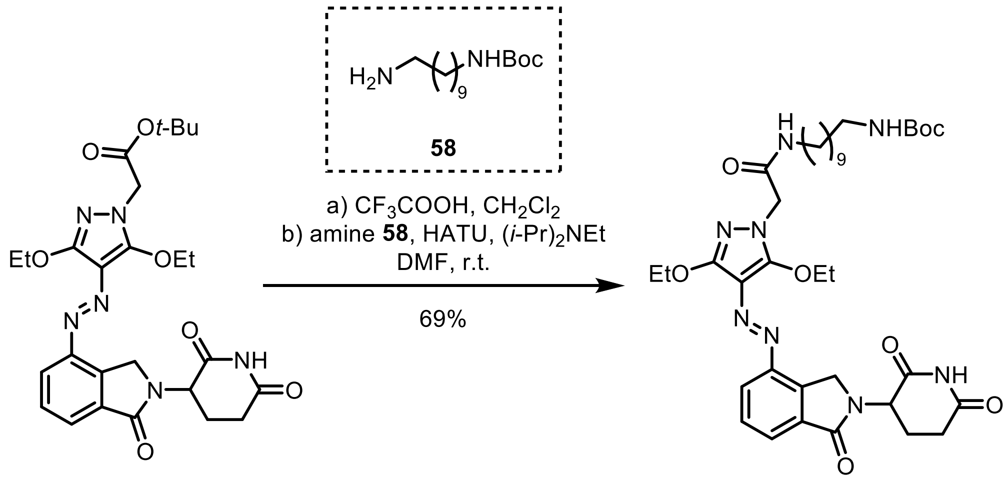

(OEt)₂-arylazopyrazole photoswitch

70

(OEt)$_2$-arylazopyrazole photoswitch (25 mg, 46 µmol, 1.0 equiv) was dissolved in CH_2Cl_2 (0.25 mL) and CF_3COOH (0.25 mL) was added. The resulting reaction mixture was stirred at r.t. for 2 h after which it was diluted with toluene and concentrated *in vacuo*. The crude product was used in the next step without further purification.

The crude product, amine **58** (13.8 mg, 50.6 µmol, 1.1 equiv) and HATU (18.3 mg, 48.3 µmol, 1.05 equiv) were dissolved in anhydrous DMF (0.46 mL) and (i-Pr)$_2$NEt (30 µL, 0.17 mmol, 3.8 equiv) was added. The resulting reaction mixture was stirred overnight after which it was concentrated *in vacuo.* Column chromatography (SiO_2, eluent: EtOAc) and C_{18}-reversed phase column chromatography (eluent: MeCN–H_2O (1:1) to MeCN–H_2O (3:2)) afforded **70** (23.3 mg, 69%) as a yellow solid.

^{1}H NMR (400 MHz, $(CD_3)_2CO$): δ = 9.82 (s, 1H), 7.92 (dd, J = 7.8, 1.1 Hz, 1H), 7.76 (dd, J = 7.5, 1.1 Hz, 1H), 7.72 – 7.61 (m, 1H), 7.26 (t, J = 5.7 Hz, 1H), 5.88 (s, 1H), 5.24 (dd, J = 13.3, 5.1 Hz, 1H), 4.85 – 4.66 (m, 4H), 4.56 (s, 2H), 4.25 (q, J = 7.1 Hz, 2H), 3.23 (td, J = 7.0, 5.8 Hz, 2H), 3.09 – 2.96 (m, 3H), 2.82 – 2.75 (m, 1H), 2.55 (qd, J = 13.3, 4.5 Hz, 1H), 2.28 (ddq, J = 10.5, 5.3, 2.7 Hz, 1H), 1.54 – 1.42 (m, 7H), 1.42 – 1.35 (m, 12H), 1.29 (d, J = 4.3 Hz, 12H) ppm.

^{13}C NMR (101 MHz, $(CD_3)_2CO$): δ = 172.7, 171.3, 168.7, 166.8, 156.6, 155.2, 150.9, 149.3, 135.8, 134.8, 130.0, 125.4, 124.1, 115.0, 78.3, 71.8, 64.4, 52.9, 50.4, 48.4, 41.1, 40.0, 32.2, 30.3, 30.3, 28.7, 27.5 (d, J = 4.0 Hz), 24.1, 15.7, 15.0 ppm.

IR (neat): 3318, 3094, 2977, 2928, 2855, 1689, 1546, 1496, 1472, 1439, 1406, 1384, 1365, 1355, 1266, 1247, 1232, 1202, 1171, 1124, 1039, 1016, 752 cm^{-1}.

HRMS (ESI): *m/z* calcd. for $C_{37}H_{55}N_8O_8$ $[M+H]^+$ 739.4137, found 739.4126.

Synthesis of KH-5-450

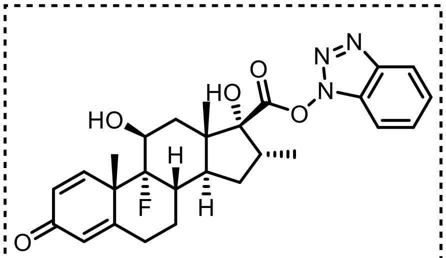

15

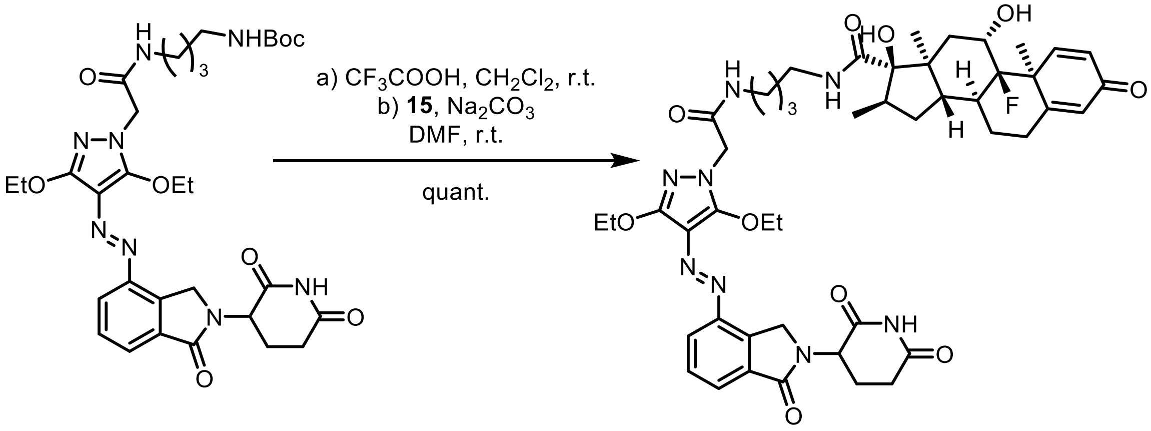

67

KH-5-450

67 (19.3 mg, 29.5 µmol, 1.00 equiv) was dissolved in CH_2Cl_2 (0.15 mL) and CF_3COOH (0.15 mL) was added. The resulting reaction mixture was stirred at r.t. for 2 h after which it was diluted with toluene and concentrated *in vacuo*. The crude product was used in the next step without further purification.

The crude product, **15** (17.5 mg, 35.3 µmol, 1.20 equiv) and Na_2CO_3 (9.35 mg, 88.2 µmol, 3.00 equiv) were dissolved in anhydrous DMF (0.28 mL). The resulting reaction mixture was stirred for 24 h at r.t., after which it was concentrated *in vacuo.* Column chromatography (SiO_2, eluent: EtOAc to EtOAc –acetone (1:1)) and C_{18}-reversed phase column chromatography (eluent: MeCN–H_2O (1:1) to MeCN–H_2O (2:1)) afforded **KH-5-450** (26.8 mg, quant.) as a yellow solid.

^{1}H NMR (500 MHz, $(CD_3)_2CO$): δ = 9.84 (s, 1H), 7.91 (ddd, *J* = 7.9, 2.3, 1.1 Hz, 1H), 7.83 – 7.76 (m, 1H), 7.75 (ddd, *J* = 7.5, 2.1, 1.1 Hz, 1H), 7.64 (tdd, *J* = 7.4, 1.3, 0.7 Hz, 1H), 7.20 (dd, *J* = 17.0, 10.1 Hz, 1H), 7.11 (dt, *J* = 7.0, 4.8 Hz, 1H), 6.19 (ddd, *J* = 15.0, 10.1, 1.9 Hz, 1H), 5.93 (dt, *J* = 10.1, 1.8 Hz, 1H), 5.23 (ddd, *J* = 13.3, 11.0, 5.2 Hz, 1H), 4.79 – 4.69 (m, 4H), 4.68 – 4.57 (m, 2H), 4.51 (dt, *J* = 9.7, 2.7 Hz, 1H), 4.30 – 4.22 (m, 2H), 4.22 – 4.15 (m, 1H), 3.94 (d, *J* = 15.8 Hz, 1H), 3.54 – 3.44 (m, 1H), 3.35 – 3.23 (m, 1H), 3.17 (dtd, *J* = 13.6, 6.9, 6.3, 3.3 Hz, 2H), 3.03 (tdd, *J* = 19.1, 11.7, 5.2 Hz, 2H), 2.79 (ddd, *J* = 17.5, 4.4, 2.3 Hz, 1H), 2.66 – 2.47 (m, 2H), 2.38 (dtd, *J* = 29.3, 12.0, 4.8 Hz, 1H), 2.32 – 2.21 (m, 2H), 2.20 – 2.09 (m, 2H), 1.84 – 1.76 (m, 1H), 1.74 – 1.49 (m, 6H), 1.50 – 1.40 (m, 7H), 1.37 (td, *J* = 7.1, 2.3 Hz, 3H), 1.16 (tt, *J* = 8.1, 4.2 Hz, 1H), 1.13 – 1.08 (m, 3H), 0.88 (dd, *J* = 7.2, 2.7 Hz, 3H) ppm.

^{13}C NMR (126 MHz, $(CD_3)_2CO$): δ = 186.3 (d, *J* = 12.1 Hz), 173.3 (d, *J* = 4.9 Hz), 172.8 (d, *J* = 4.3 Hz), 171.3 (d, *J* = 9.9 Hz), 168.7 (d, *J* = 3.2 Hz), 168.0 (d, *J* = 5.8 Hz), 167.3 (dd, *J* = 3.5, 1.7 Hz), 154.9 (d, *J* = 18.8 Hz), 153.3 (d, *J* = 3.5 Hz), 151.1 (d, *J* = 13.4 Hz), 149.2 (d, *J* = 2.0 Hz), 135.8 (d, *J* = 17.1 Hz), 134.7 (d, *J* = 5.0 Hz), 130.1 (dd, *J* = 38.0, 4.0 Hz), 126.4 – 124.7 (m), 124.1 (d, *J* = 2.1 Hz), 115.0 (d, *J* = 7.8 Hz), 102.7 (d, *J* = 9.9 Hz), 101.3 (d, *J* = 10.1 Hz), 87.8, 73.0 (d, *J* = 38.1 Hz), 71.8 (d, *J* = 2.0 Hz), 64.4 (d, *J* = 1.2 Hz), 52.9 (d, *J* = 11.7 Hz), 50.2, 49.2 (dd, *J* = 22.7, 2.9 Hz), 48.8 (d, *J* = 3.9 Hz), 48.4 (d, *J* = 27.5 Hz), 44.6 (t, *J* = 1.7 Hz), 40.6 (d, *J* = 8.0 Hz), 39.1 (d, *J* = 4.1 Hz), 36.2 (d, *J* = 6.8 Hz), 35.7 (d, *J* = 4.6 Hz), 35.2 (dd, *J* = 19.5, 1.7 Hz), 33.1, 32.2 (d, *J* = 1.3 Hz), 31.5 (d, *J* = 3.0 Hz), 28.4, 27.8 (d, *J* = 5.1 Hz), 27.6 (d, *J* = 7.1 Hz), 24.1 (d, *J* = 11.0 Hz), 23.6 (dd, *J* = 5.9, 2.3 Hz), 17.7 (d, *J* = 1.7 Hz), 15.7, 15.3, 15.0 ppm.

IR (neat): 3385, 3095, 2936, 2871, 1663, 1547, 1496, 1473, 1438, 1406, 1384, 1356, 1299, 1266, 1232, 1203, 1126, 1035, 1014, 893, 818, 801, 754, 722 cm^{-1}.

HRMS (ESI): *m/z* calcd. for $C_{47}H_{60}FN_8O_{10}$ $[M+H]^+$ 915.4411, found 915.4393.

Synthesis of KH-5-452

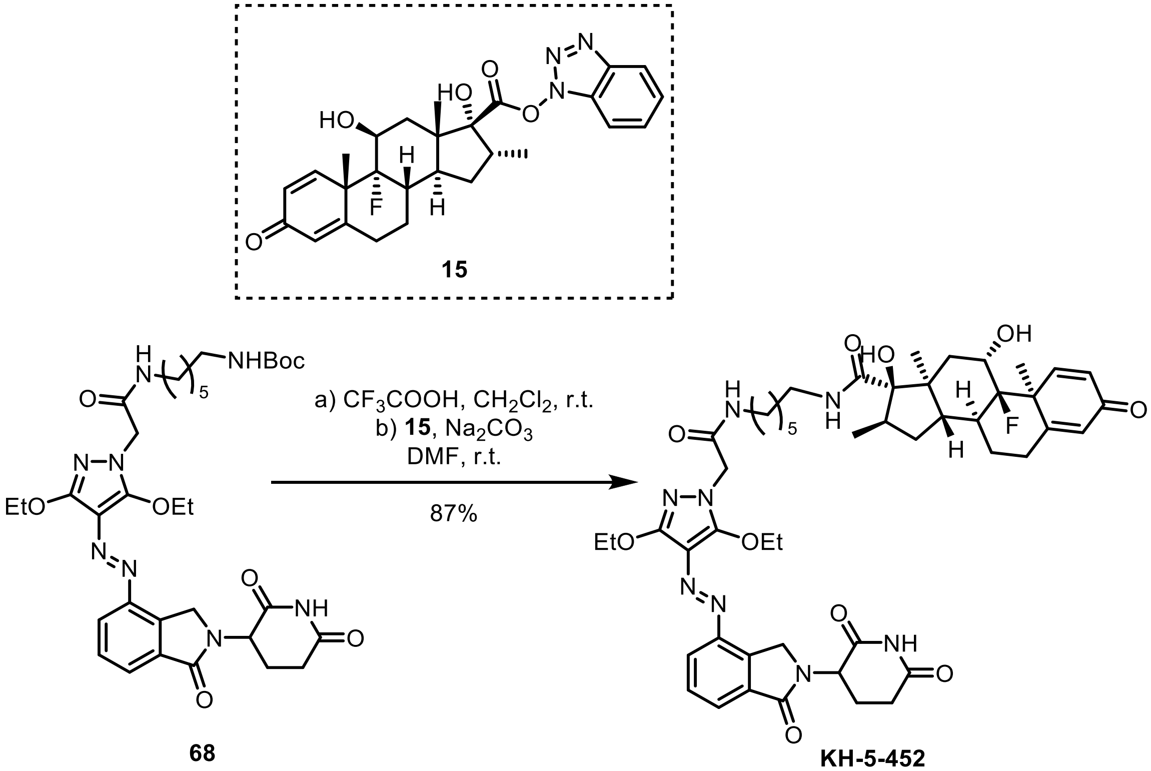

68 (23.9 mg, 35.0 µmol, 1.00 equiv) was dissolved in CH_2Cl_2 (0.18 mL) and CF_3COOH (0.18 mL) was added. The resulting reaction mixture was stirred at r.t. for 2 h after which it was diluted with toluene and concentrated *in vacuo*. The crude product was used in the next step without further purification.

The crude product, **15** (20.7 mg, 41.8 µmol, 1.20 equiv) and Na_2CO_3 (11.1 mg, 105 µmol, 3.00 equiv) were dissolved in anhydrous DMF (0.35 mL). The resulting reaction mixture was stirred for 24 h at r.t., after which it was concentrated *in vacuo.* Column chromatography (SiO_2, eluent: EtOAc to EtOAc –acetone (1:1)) and C_{18}-reversed phase column chromatography (eluent: MeCN–H_2O (1:1) to MeCN–H_2O (2:1)) afforded **KH-5-452** (28.7 mg, 87%) as a yellow solid.

^{1}H NMR (500 MHz, $(CD_3)_2CO$): δ = 9.86 (s, 1H), 7.91 (dd, *J* = 7.9, 1.1 Hz, 1H), 7.76 (dt, *J* = 7.4, 0.9 Hz, 1H), 7.68 – 7.61 (m, 1H), 7.53 (t, *J* = 5.8 Hz, 1H), 7.24 (t, *J* = 9.9 Hz, 1H), 7.07 (d, *J* = 5.8 Hz, 1H), 6.18 (ddd, *J* = 10.1, 4.8, 1.9 Hz, 1H), 5.96 (dt, *J* = 5.8, 1.8 Hz, 1H), 5.24 (ddd, *J* = 13.3, 5.1, 3.6 Hz, 1H), 4.78 – 4.69 (m, 4H), 4.61 (d, *J* = 1.6 Hz, 2H), 4.60 – 4.54 (m, 1H), 4.25 (qd, *J* = 7.1, 0.9 Hz, 2H), 3.95 (d, *J* = 5.2 Hz, 1H), 3.34 (dq, *J* = 12.9, 6.4 Hz, 1H), 3.27 (q, *J* = 6.5 Hz, 2H), 3.13 (dddd, *J* = 12.7, 9.1, 6.6, 3.7 Hz, 2H), 3.01 (ddd, *J* = 17.6, 13.7, 5.4 Hz, 1H), 2.79 (ddd, *J* = 17.5, 4.4, 2.4 Hz, 1H), 2.70 – 2.61 (m, 1H), 2.61 – 2.49 (m, 1H), 2.48 – 2.34 (m, 1H), 2.33 – 2.23 (m, 2H), 2.21 – 2.12 (m, 2H), 1.81 (dd, *J* = 12.4, 6.1 Hz, 1H), 1.76 – 1.65 (m, 1H), 1.59 – 1.46 (m,

8H), 1.44 (t, J = 7.0 Hz, 4H), 1.37 (td, J = 7.0, 2.7 Hz, 7H), 1.21 – 1.13 (m, 1H), 1.10 (s, 3H), 0.88 (dd, J = 7.3, 1.4 Hz, 3H) ppm.

^{13}C NMR (126 MHz, $(CD_3)_2CO$): δ = 186.2, 173.4, 172.8, 171.4 (d, J = 3.5 Hz), 168.7, 167.6 (d, J = 4.5 Hz), 167.3, 155.1 (d, J = 2.6 Hz), 153.1 (d, J = 3.6 Hz), 151.0 (d, J = 5.0 Hz), 149.2, 135.8 (d, J = 10.0 Hz), 134.7 (d, J = 2.0 Hz), 130.1 (d, J = 26.4 Hz), 125.4 (d, J = 16.6 Hz), 124.1, 115.0 (d, J = 2.9 Hz), 102.8 (d, J = 3.4 Hz), 101.4 (d, J = 3.4 Hz), 87.7, 72.6 (dd, J = 37.7, 1.7 Hz), 71.8, 64.4, 52.9 (d, J = 5.4 Hz), 50.3 (d, J = 2.0 Hz), 49.2 (d, J = 22.8 Hz), 48.8, 48.4 (d, J = 8.2 Hz), 44.5, 39.6, 39.5 (d, J = 5.5 Hz), 36.7, 35.8 (d, J = 2.0 Hz), 35.2 (d, J = 19.4 Hz), 33.1, 32.2, 31.6, 28.4 (d, J = 1.8 Hz), 26.8 (d, J = 6.2 Hz), 26.7 (d, J = 4.4 Hz), 24.1 (d, J = 3.6 Hz), 23.7 (d, J = 5.7 Hz), 17.7, 15.7, 15.3, 15.0 ppm.

IR (neat): 3379, 3096, 2934, 2868, 1663, 1548, 1496, 1473, 1436, 1406, 1385, 1355, 1299, 1266, 1233, 1204, 1126, 1034, 1014, 893, 818, 802, 754 cm^{-1}.

HRMS (ESI): *m/z* calcd. for $C_{49}H_{64}FN_8O_{10}$ $[M+H]^+$ 943.4724, found 943.4708.

Synthesis of KH-5-454

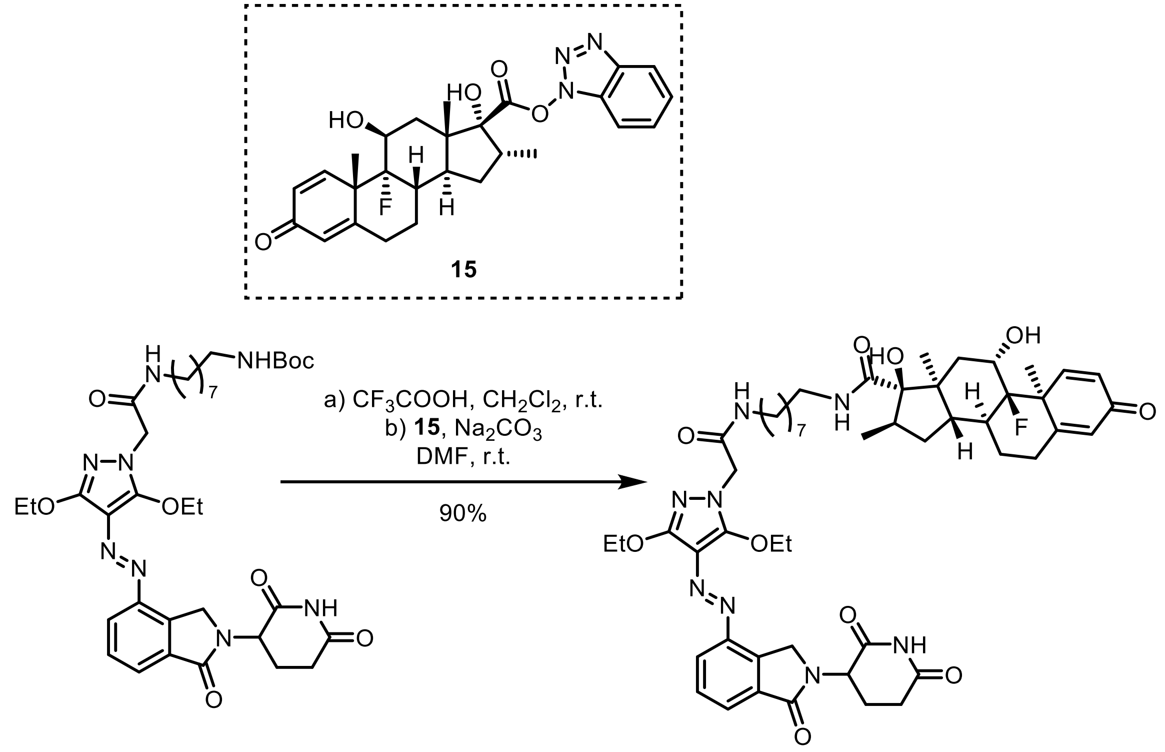

69 (25.3 mg, 35.6 µmol, 1.0 equiv) was dissolved in CH_2Cl_2 (0.18 mL) and CF_3COOH (0.18 mL) was added. The resulting reaction mixture was stirred at r.t. for 2 h after which it was diluted with toluene and concentrated *in vacuo*. The crude product was used in the next step without further purification.

The crude product, **15** (21.1 mg, 42.6 µmol, 1.2 equiv) and Na_2CO_3 (11.3 mg, 107 µmol, 3.0 equiv) were dissolved in anhydrous DMF (0.35 mL). The resulting reaction mixture was stirred for 24 h at r.t., after which it was concentrated *in vacuo.* Column chromatography (SiO_2, eluent: EtOAc to EtOAc –acetone (1:1)) and C_{18}-reversed phase column chromatography (eluent: MeCN–H_2O (1:1) to MeCN–H_2O (2:1)) afforded **KH-5-454** (31 mg, 90%) as a yellow solid.

^{1}H NMR (500 MHz, $(CD_3)_2CO$): δ = 9.88 (s, 1H), 7.91 (dt, *J* = 7.9, 1.0 Hz, 1H), 7.76 (dt, *J* = 7.5, 0.9 Hz, 1H), 7.65 (t, *J* = 7.7 Hz, 1H), 7.42 (td, *J* = 5.9, 2.4 Hz, 1H), 7.30 (dd, *J* = 10.1, 1.5 Hz, 1H), 7.08 (t, *J* = 6.0 Hz, 1H), 6.18 (dt, *J* = 10.2, 1.9 Hz, 1H), 5.98 (q, *J* = 1.9 Hz, 1H), 5.24 (dd, *J* = 13.3, 5.2 Hz, 1H), 4.77 – 4.67 (m, 4H), 4.59 (s, 2H), 4.53 (dd, *J* = 4.2, 1.9 Hz, 1H), 4.29 (ddt, *J* = 10.1, 3.3, 1.6 Hz, 1H), 4.25 (qd, *J* = 7.0, 0.8 Hz, 2H), 3.95 (s, 1H), 3.37 – 3.20 (m, 3H), 3.20 – 3.08 (m, 2H), 3.01 (ddd, *J* = 17.5, 13.7, 5.4 Hz, 1H), 2.80 (ddd, *J* = 17.5, 4.5, 2.4 Hz, 1H), 2.73 – 2.63 (m, 1H), 2.60 – 2.48 (m, 1H), 2.42 (ddd, *J* = 29.2, 11.9, 4.8 Hz, 1H), 2.37 – 2.30 (m, 1H), 2.30 – 2.23 (m, 1H), 2.22 – 2.14 (m, 2H), 1.84 (dt, *J* = 11.8, 5.4 Hz, 1H), 1.71 (tdd, *J* = 12.3, 11.0, 1.6 Hz, 1H), 1.57

(d, J = 1.6 Hz, 3H), 1.55 – 1.45 (m, 6H), 1.43 (t, J = 7.0 Hz, 3H), 1.37 (t, J = 7.1 Hz, 3H), 1.31 (s, 8H), 1.17 (ddd, J = 12.3, 8.3, 4.2 Hz, 1H), 1.11 (s, 3H), 0.89 (dd, J = 7.3, 1.0 Hz, 3H) ppm.

^{13}C NMR (126 MHz, $(CD_3)_2CO$): δ = 186.2 (d, J = 1.1 Hz), 173.3, 172.8, 171.4 (d, J = 1.2 Hz), 168.8, 167.3 (d, J = 1.7 Hz), 167.2 (d, J = 2.1 Hz), 155.1 (d, J = 1.7 Hz), 153.1, 150.9, 149.2 (d, J = 1.4 Hz), 135.7 (d, J = 5.6 Hz), 134.7, 130.0 (d, J = 20.7 Hz), 125.5 (d, J = 7.9 Hz), 125.3, 124.2, 115.0, 102.8 (d, J = 1.5 Hz), 101.4 (d, J = 1.5 Hz), 87.6, 72.6 (d, J = 37.6 Hz), 71.8, 64.4, 52.9 (d, J = 1.4 Hz), 50.3, 49.2 (d, J = 22.9 Hz), 48.8, 48.4 (d, J = 1.4 Hz), 44.5 (d, J = 1.6 Hz), 39.9 (d, J = 10.0 Hz), 36.7 (d, J = 1.5 Hz), 35.9, 35.2 (d, J = 19.3 Hz), 33.1, 32.2, 31.6, 28.4 (d, J = 1.8 Hz), 27.5 (d, J = 1.7 Hz), 27.3 (d, J = 1.6 Hz), 24.1, 23.7 (d, J = 5.7 Hz), 17.7, 15.7, 15.2, 15.0 ppm.

IR (neat): 3385, 3101, 2934, 2862, 1663, 1627, 1548, 1496, 1477, 1442, 1412, 1385, 1355, 1302, 1271, 1233, 1204, 1125, 1034, 1018, 893, 816, 754, 816 cm^{-1}.

HRMS (ESI): *m/z* calcd. for $C_{51}H_{68}FN_8O_{10}$ [M+H]$^+$ 971.5037, found 971.5028.

Synthesis of KH-5-456

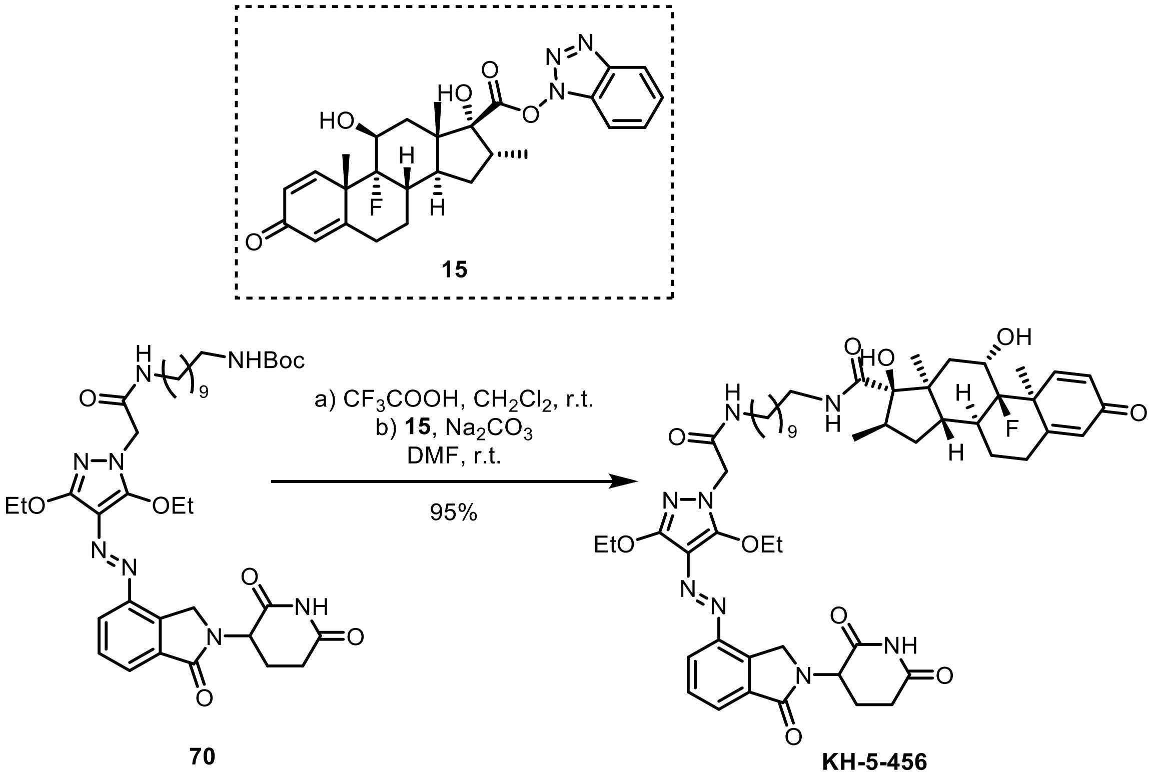

70 (23.3 mg, 31.5 µmol, 1.0 equiv) was dissolved in CH_2Cl_2 (0.16 mL) and CF_3COOH (0.16 mL) was added. The resulting reaction mixture was stirred at r.t. for 2 h after which it was diluted with toluene and concentrated *in vacuo*. The crude product was used in the next step without further purification.

The crude product, **15** (18.7 mg, 37.8 µmol, 1.20 equiv) and Na_2CO_3 (10.0 mg, 94.4 µmol, 3.00 equiv) were dissolved in anhydrous DMF (0.31 mL). The resulting reaction mixture was stirred for 24 h at r.t., after which it was concentrated *in vacuo.* Column chromatography (SiO_2, eluent: EtOAc to EtOAc –acetone (1:1)) and C_{18}-reversed phase column chromatography (eluent: MeCN–H_2O (1:1) to MeCN–H_2O (2:1)) afforded **KH-5-456** (29.8 mg, 95%) as a yellow oil.

^{1}H NMR (500 MHz, $(CD_3)_2CO$): δ = 9.88 (s, 1H), 7.92 (dd, *J* = 7.6, 1.0 Hz, 1H), 7.77 (dt, *J* = 7.4, 0.9 Hz, 1H), 7.70 – 7.62 (m, 1H), 7.34 (t, *J* = 5.8 Hz, 1H), 7.30 (d, *J* = 10.2 Hz, 1H), 7.07 (t, *J* = 6.0 Hz, 1H), 6.18 (ddd, *J* = 10.1, 1.9, 0.6 Hz, 1H), 5.99 (t, *J* = 1.8 Hz, 1H), 5.25 (dd, *J* = 13.4, 5.1 Hz, 1H), 4.79 – 4.67 (m, 4H), 4.57 (s, 2H), 4.43 (dt, *J* = 3.8, 1.9 Hz, 1H), 4.31 (dt, *J* = 10.5, 3.0 Hz, 1H), 4.25 (q, *J* = 7.0 Hz, 2H), 3.95 (s, 1H), 3.29 – 3.20 (m, 3H), 3.15 (dddd, *J* = 12.6, 7.9, 6.4, 3.1 Hz, 2H), 3.01 (ddd, *J* = 17.5, 13.6, 5.4 Hz, 1H), 2.80 (ddd, *J* = 17.5, 4.5, 2.4 Hz, 1H), 2.69 (tdd, *J* = 13.8, 6.1, 1.8 Hz, 1H), 2.60 – 2.50 (m, 1H), 2.50 – 2.38 (m, 1H), 2.34 (ddd, *J* = 13.8, 5.2, 1.9 Hz, 1H), 2.28 (dtdd, *J* = 13.0, 5.5, 2.5, 0.8 Hz, 1H), 2.23 – 2.15 (m, 2H), 1.85 (dt, *J* = 11.9, 5.5 Hz, 1H), 1.72 (tdd, *J* = 12.2, 11.0, 1.6 Hz, 1H), 1.59 (s, 3H), 1.55 – 1.45 (m, 6H), 1.44 (t, *J* = 7.0 Hz, 3H), 1.37 (t, *J* = 7.1 Hz, 3H), 1.29 (d, *J* = 6.5 Hz, 12H), 1.18 (ddd, *J* = 12.3, 8.3, 4.2 Hz, 1H), 1.11 (s, 3H), 0.89 (dd, *J* = 7.3, 0.8 Hz, 3H) ppm.

^{13}C NMR (126 MHz, $(CD_3)_2CO$): δ = 186.2, 173.2, 172.8, 171.3 (d, *J* = 1.0 Hz), 168.8, 167.3 (d, *J* = 1.8 Hz), 167.0, 155.2, 153.1, 150.9, 149.2, 135.7 (d, *J* = 1.4 Hz), 134.7, 130.0 (d, *J* = 17.8 Hz), 125.5 (d, *J* = 1.5 Hz), 125.3 (d, *J* = 1.4 Hz), 124.2, 115.0, 102.7, 101.3, 87.6, 72.7 (d, *J* = 37.8 Hz), 71.8, 64.4, 52.9, 50.4, 49.2 (d, *J* = 22.9 Hz), 48.8, 48.4, 44.5 (d, *J* = 1.6 Hz), 39.9 (d, *J* = 10.8 Hz), 36.7 (d, *J* = 1.5 Hz), 35.9, 35.2 (d, *J* = 19.3 Hz), 33.1, 32.2, 31.6, 30.6, 28.4 (d, *J* = 1.8 Hz), 27.6, 27.5, 24.1, 23.7 (d, *J* = 5.7 Hz) ppm.

IR (neat): 3378, 3097, 2930, 2854, 1663, 1632, 1546, 1496, 1439, 1412, 1383, 1354, 1300, 1268, 1232, 1203, 1124, 1034, 1011, 893, 819, 754 cm^{-1}.

HRMS (ESI): *m/z* calcd. for $C_{53}H_{72}FN_8O_{10}$ $[M+H]^+$ 999.5350, found 999.5334.

(NMe$_2$)$_2$-Arylazopyrazole Photoswitch

Synthesis of 46

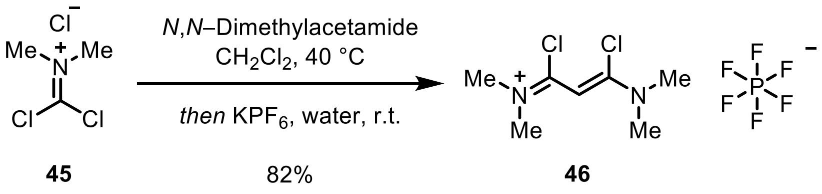

N,N–Dimethylacetamide (1.0 mL, 11 mmol, 1.0 equiv) was added to a solution of phosgene iminium chloride **45** (3.5 g, 22 mmol, 2.0 equiv) in CH_2Cl_2 (225 mL) at r.t. After refluxing the resulting reaction suspension overnight, the solvent was removed *in vacuo*, and the residue was resuspended in cold water. A solution of KPF_6 (2.0 g, 11 mmol, 1.0 equiv) in water (5 mL) was immediately added and the obtained solid was filtered, washed thrice with ether, and dried under vacuum. **46** (3.0 g, 82%) was used for the next step without further purification.

^{1}H NMR (400 MHz, ($CDCl_3$)): δ = 5.55 (s, 1H), 3.49 (s, 12H) ppm.

^{13}C NMR (101 MHz, ($CDCl_3$)): δ = 159.0, 89.5, 44.1 ppm.

^{19}F NMR (377 MHz, ($CDCl_3$)): δ = -71.94, -73.83 ppm.

IR (neat): 3048, 2943, 2885, 2808, 1564, 1461, 1427, 1389, 1305, 1226, 1131, 1057, 881, 745, 736, 698, 554 cm^{-1}.

HRMS (ESI): *m/z* calcd. for $C_7H_{13}Cl_2N_2$ $[M]^+$ 195.0450, found 195.0447.

Synthesis of 49

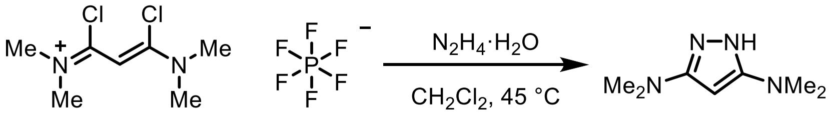

46 **47**

$N_2H_4{\cdot}H_2O$ (46.2 mg, 45.0 µL, 923 µmol, 1.05 equiv) was added to a suspension of **46** (300 mg, 877 µmol, 1.00 equiv) in CH_2Cl_2 (7 mL) and the reaction mixture was warmed to 45 °C. After stirring the mixture for 60 min, it was concentrated in *vacuo* to afford crude **47** as a white solid which was used for the next step without further purification.

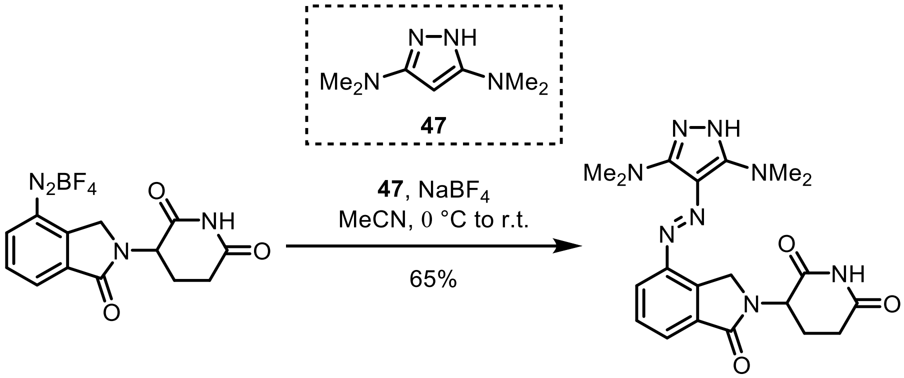

35 **49**

35 (315 mg, 877 µmol, 1.00 equiv) in MeCN (4 mL) was added to a suspension of **47** and $NaBF_4$ (0.96 mg, 8.8 mmol, 10 equiv) in MeCN (15 mL) at 0 °C and the flask was washed twice with MeCN (2 x 1 mL). The reaction mixture was warmed up to 5 °C over a period of 30 min and then stirred for another 30 min at r.t. After the addition of EtOAc, the mixture was filtered and washed with EtOAc. The filtrate was concentrated under reduced pressure and the obtained solid was transferred to a funnel and washed with EtOAc–acetone (1:1) and acetone to afford **49** (242 mg, 65%) as a red solid.

^{1}H NMR (400 MHz, $(CD_3)_2SO$): δ = 11.00 (s, 1H), 7.68 (dd, *J* = 7.6, 1.4 Hz, 1H), 7.64 – 7.52 (m, 2H), 5.16 (dd, *J* = 13.3, 5.1 Hz, 1H), 4.74 – 4.47 (m, 2H), 3.04 (s, 12H), 2.93 (ddd, *J* = 17.2, 13.6, 5.3 Hz, 1H), 2.68 – 2.57 (m, 1H), 2.50 – 2.40 (m, 1H), 2.08 – 2.01 (m, 1H) ppm.

^{13}C NMR (101 MHz, $(CD_3)_2SO$): δ =172.9, 171.1, 168.1, 148.4, 135.9, 133.1, 129.1, 120.7, 118.9 (d, *J* = 3.6 Hz), 51.8, 46.9, 41.1, 31.3, 22.4 ppm.

IR (neat): 3226, 3100, 2945, 2856, 1691, 1607, 1541, 1380, 1338, 1267, 1234, 1203, 1058, 844, 747 cm^{-1}.

HRMS (ESI): *m/z* calcd. for $C_{20}H_{25}N_8O_3$ $[M+H]^+$ 425.2044, found 425.2043.

Synthesis of $(NMe_2)_2$-arylazopyrazole photoswitch

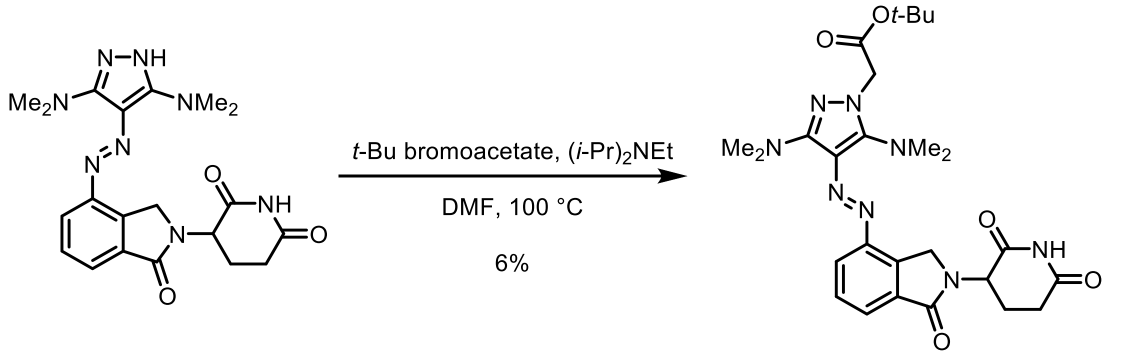

49 **$(NMe_2)_2$-arylazopyrazole photoswitch**

t-Bu bromoacetate (54 µL, 0.37 mmol, 1.03 equiv) was added in portions to a solution of **49** (150 mg, 353 µmol, 1.0 equiv) in DMF (3.6 mL) and (i-Pr)$_2$NEt (150 µL, 867 µmol, 2.5 equiv) over the course of 10 h at 100 °C. After stirring the reaction mixture for another hour, it was concentrated in *vacuo* and purified by column chromatography (SiO_2, eluent: EtOAc to acetone–EtOAc (1:1)) and C_{18}-reversed phase column chromatography (eluent: MeCN–H_2O (1:1)) to afford **$(NMe_2)_2$-arylazopyrazole photoswitch** (11 mg, 6%) as a red oil.

^{1}H NMR (400 MHz, $(CD_3)_2CO$): δ = 9.78 (s, 1H), 7.92 (dd, *J* = 7.9, 1.1 Hz, 1H), 7.75 (dd, *J* = 7.4, 1.1 Hz, 1H), 7.65 (t, *J* = 7.7 Hz, 1H), 5.24 (dd, *J* = 13.3, 5.1 Hz, 1H), 4.90 – 4.76 (m, 2H), 4.70 (s, 2H), 3.05 (s, 6H), 3.02 – 2.95 (m, 1H), 2.82 (s, 6H), 2.81 – 2.75 (m, 1H), 2.62 (qd, *J* = 13.3, 4.5 Hz, 1H), 2.27 (ddq, *J* = 10.5, 5.3, 2.7 Hz, 1H), 1.48 (s, 9H) ppm.

^{13}C NMR (101 MHz, $(CD_3)_2CO$): δ = 172.8, 171.3, 168.8, 168.0, 155.5, 149.3, 145.6, 136.8, 134.8, 130.0, 126.0, 123.8, 123.0, 82.5, 53.0, 50.9, 48.5, 42.2, 41.34, 32.3, 28.2, 24.0 ppm.

IR (neat): 3455, 3200, 3103, 2981, 2934, 2871, 1697, 1442, 1479, 1440, 1414, 1369, 1300, 1268, 1232, 1201, 1154, 1082, 1054, 1028, 994, 937, 860 751 cm^{-1}.

HRMS (ESI): *m/z* calcd. for $C_{26}H_{35}N_8O_5$ $[M+H]^+$ 539.2725, found 539.2717.

5. NMR Spectra

Arylazotriazole-dexa-linked photoPROTACs

^{1}H NMR (500 MHz, CD_3OD) of compound **39**:

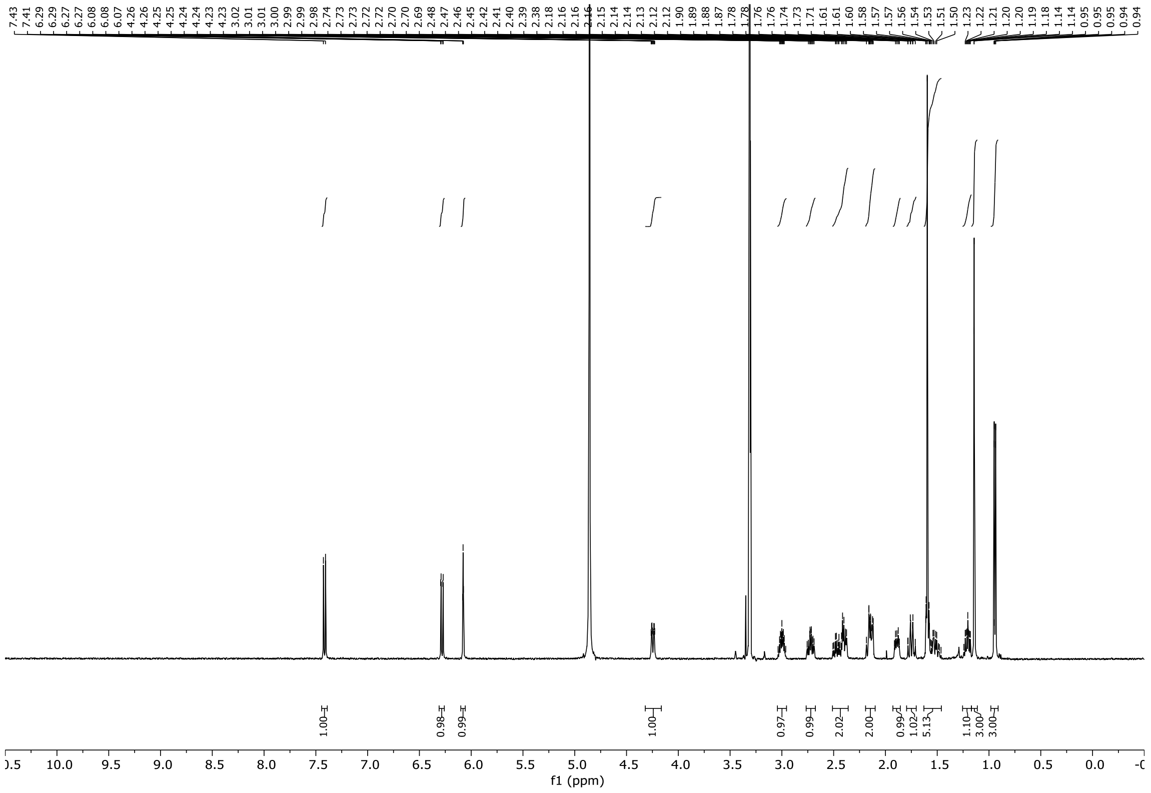

^{13}C NMR (126 MHz, CD_3OD) of compound **39**:

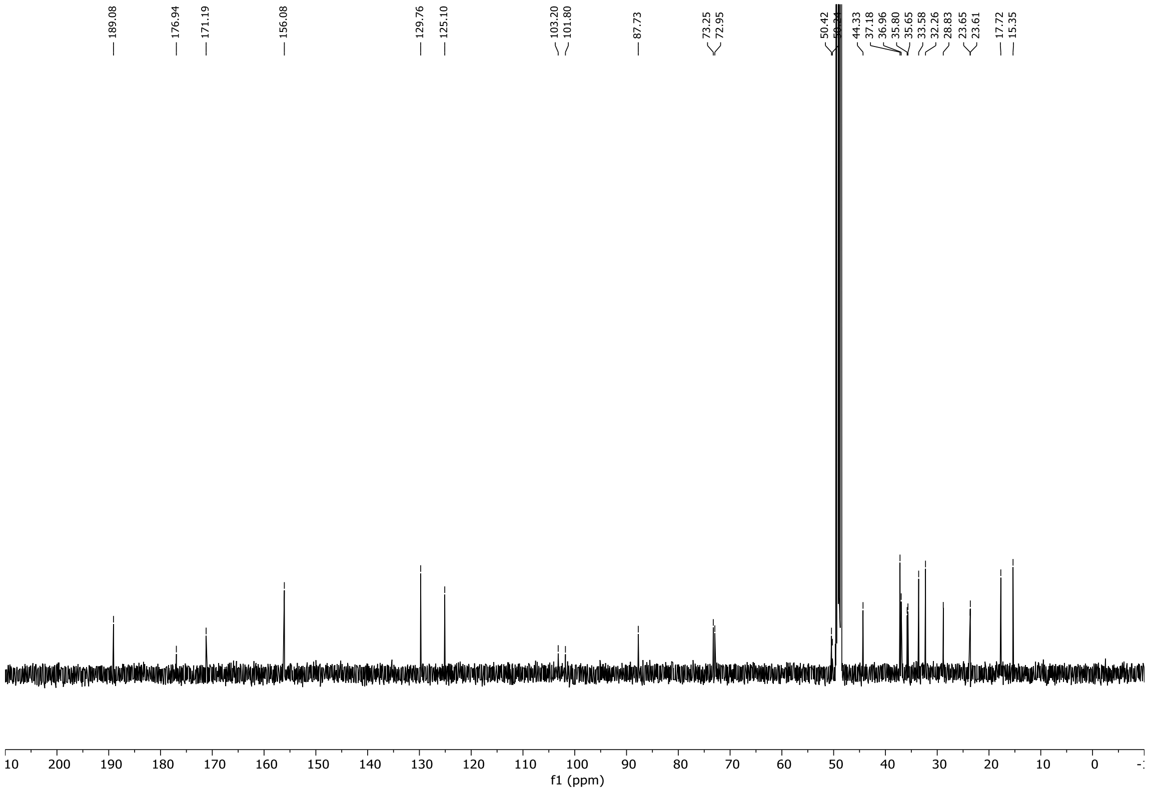

^{19}F NMR (376 MHz, CD_3OD) of compound **39**:

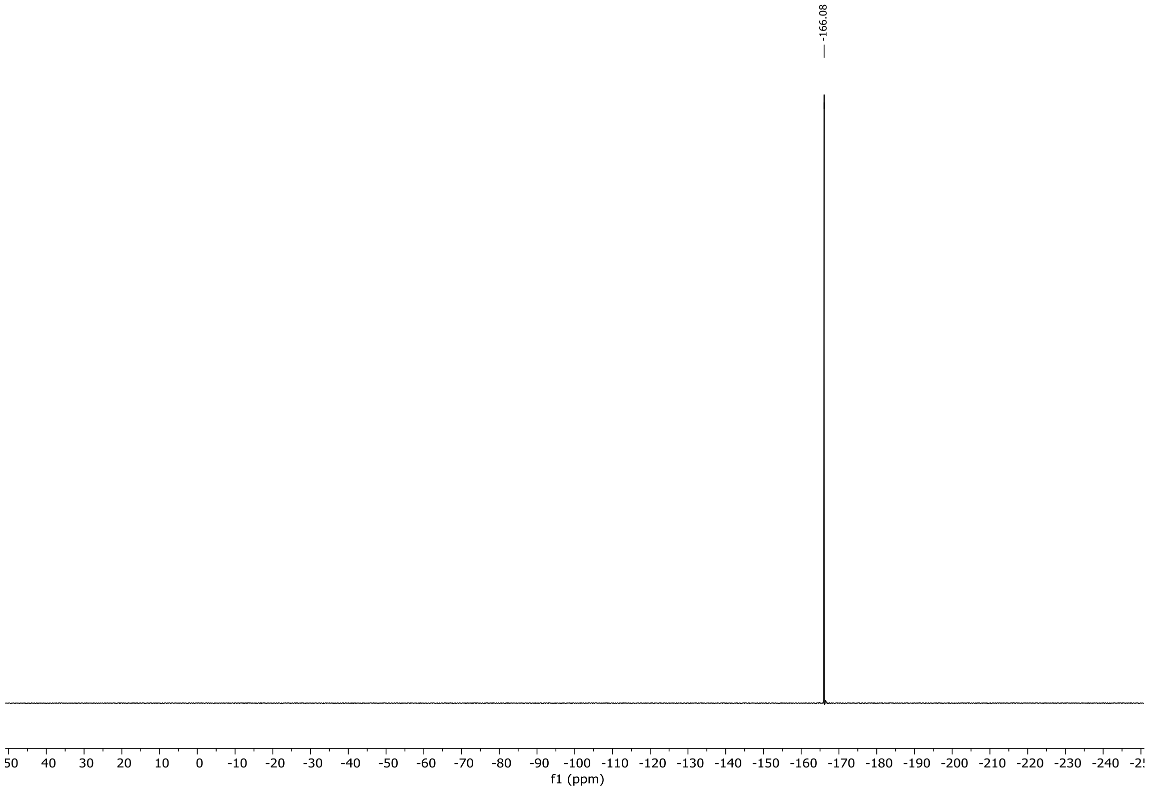

^{1}H NMR (500 MHz, $(CD_3)_2CO$) of compound **15**:

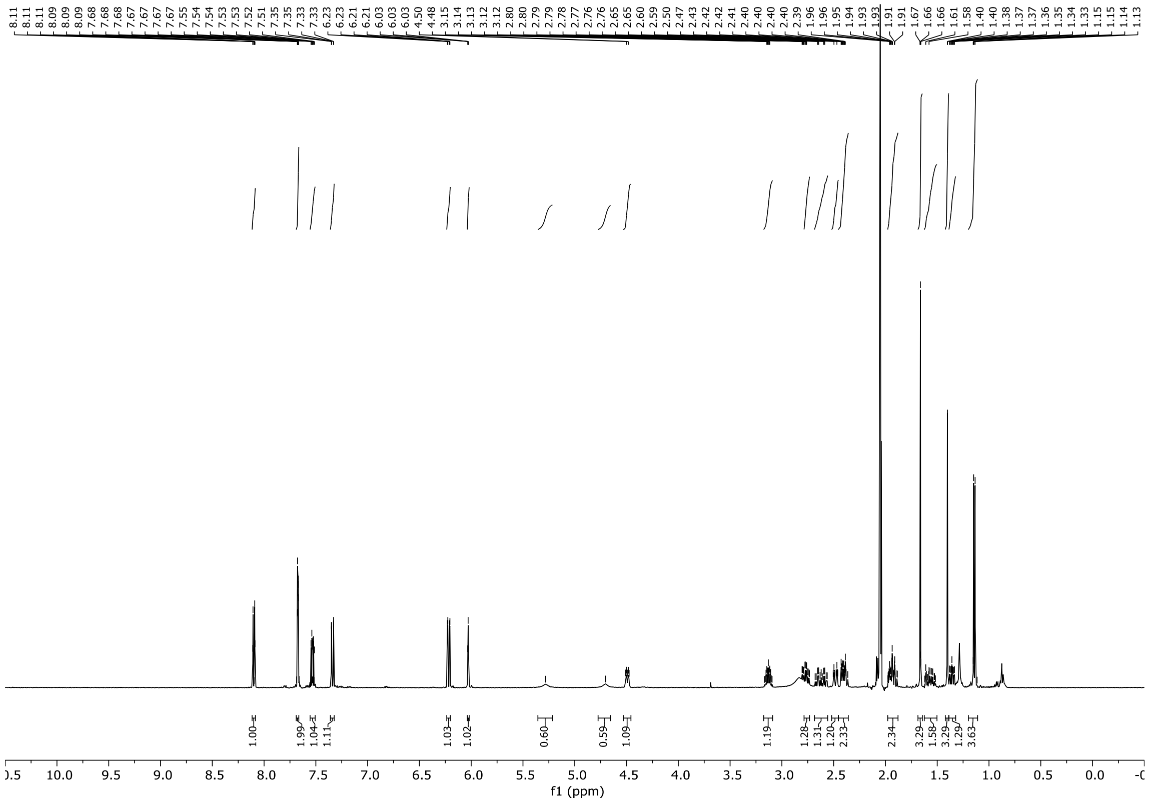

^{13}C NMR (126 MHz, $(CD_3)_2CO$) of compound **15**:

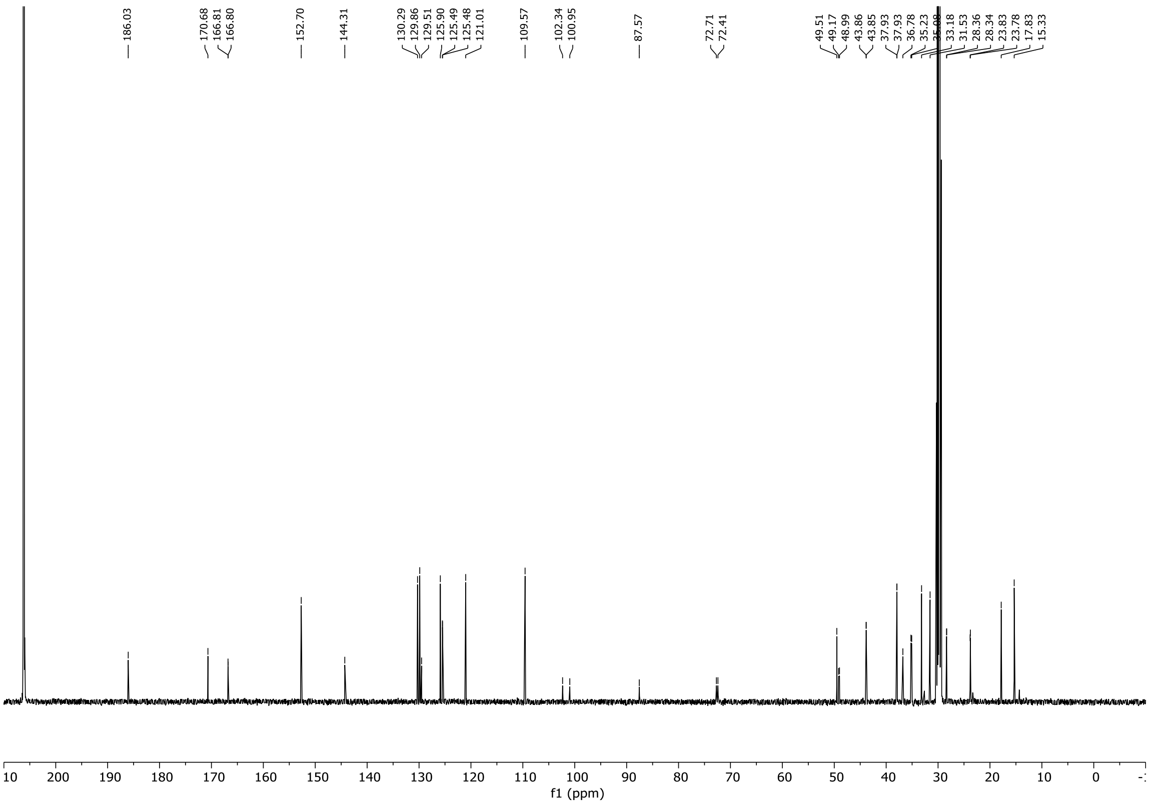

^{19}F NMR (471 MHz, $(CD_3)_2CO$) of compound **15**:

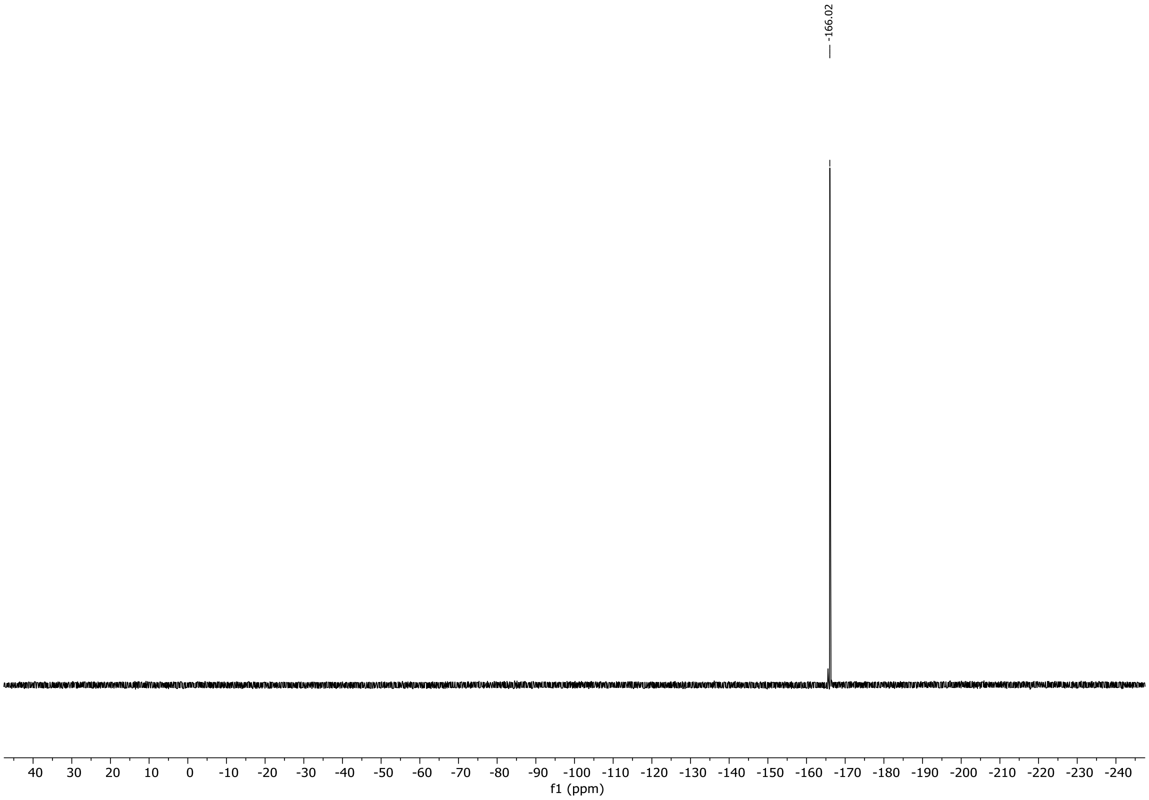

^{1}H NMR (500 MHz, CD_3OD) of compound **17**:

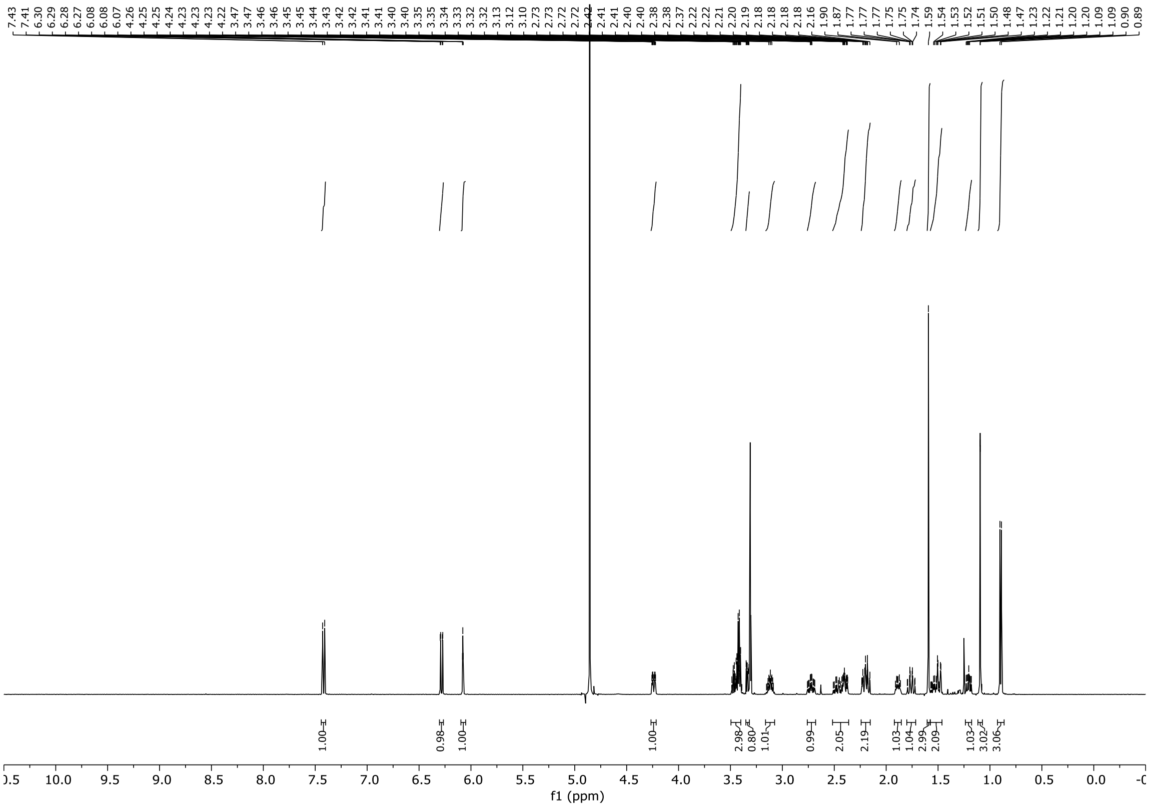

^{13}C NMR (125 MHz, CD_3OD) of compound **17**:

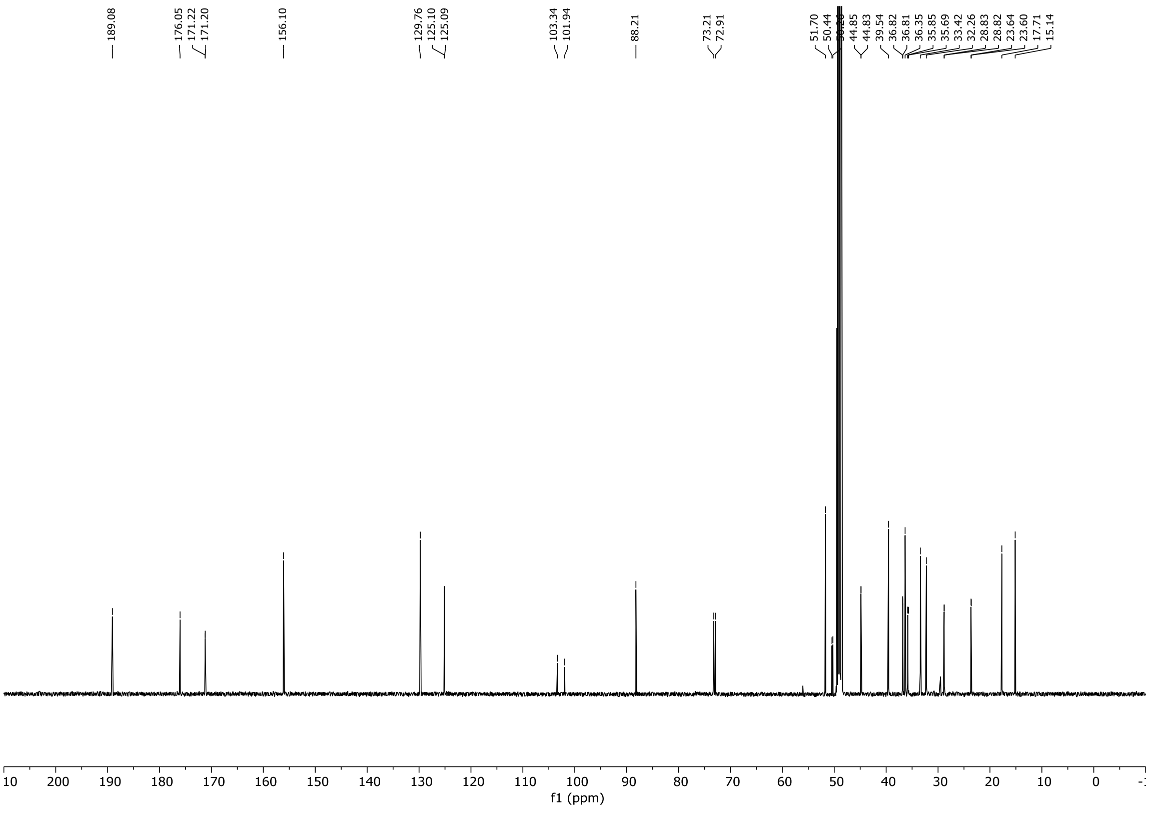

^{19}F NMR (471 MHz, CD_3OD) of compound **17**:

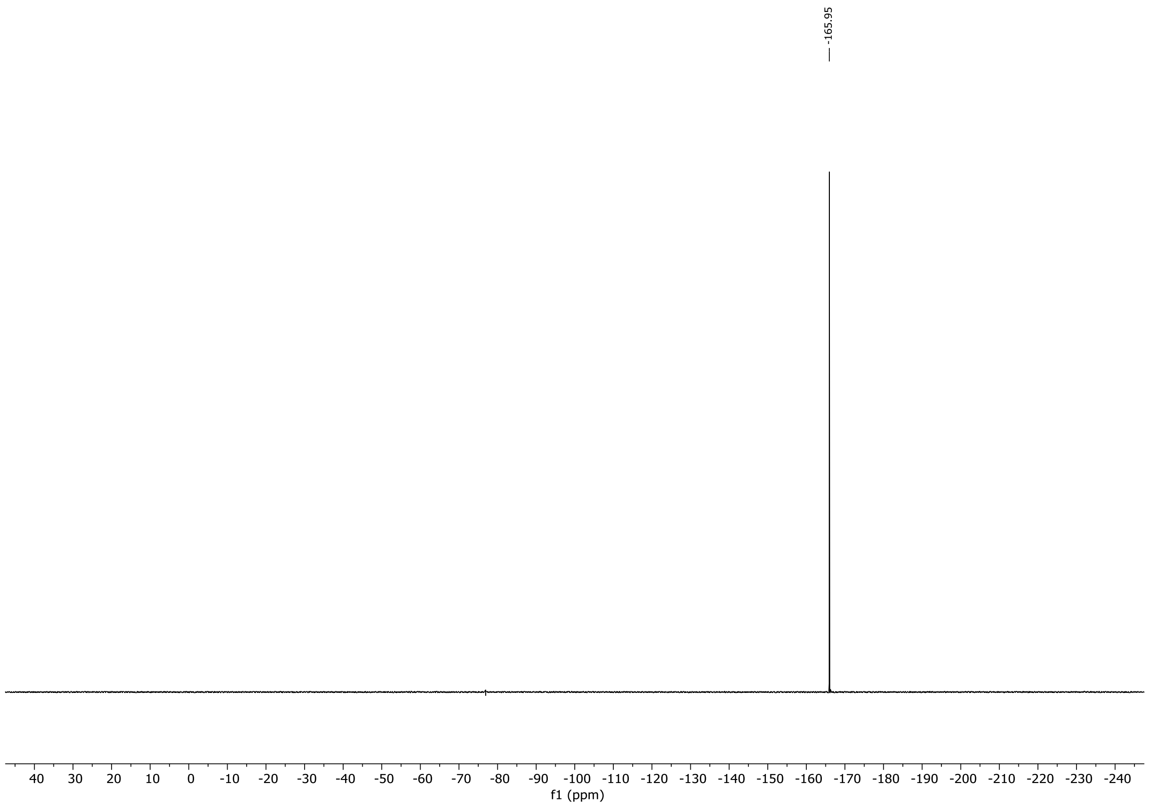

^{1}H NMR (600 MHz, CD_3OD) of compound **KH-5-168**:

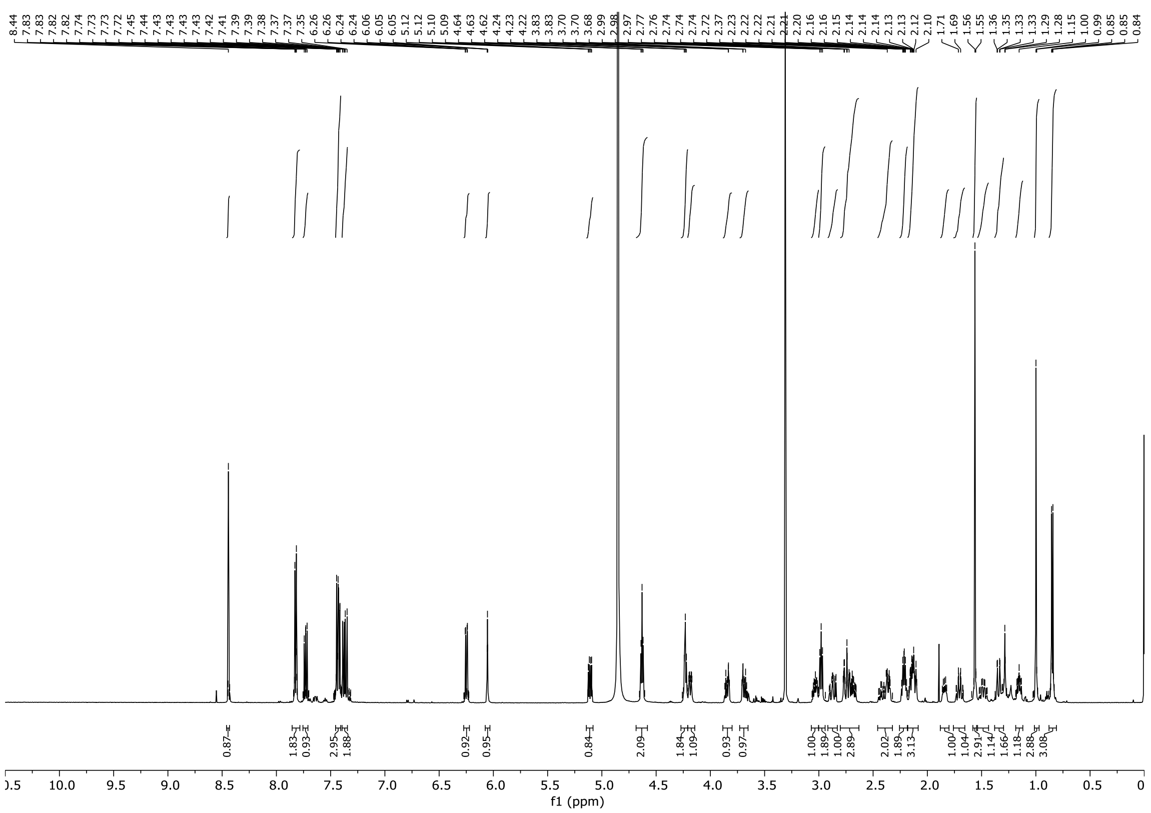

^{13}C NMR (150 MHz, CD_3OD) of compound **KH-5-168**:

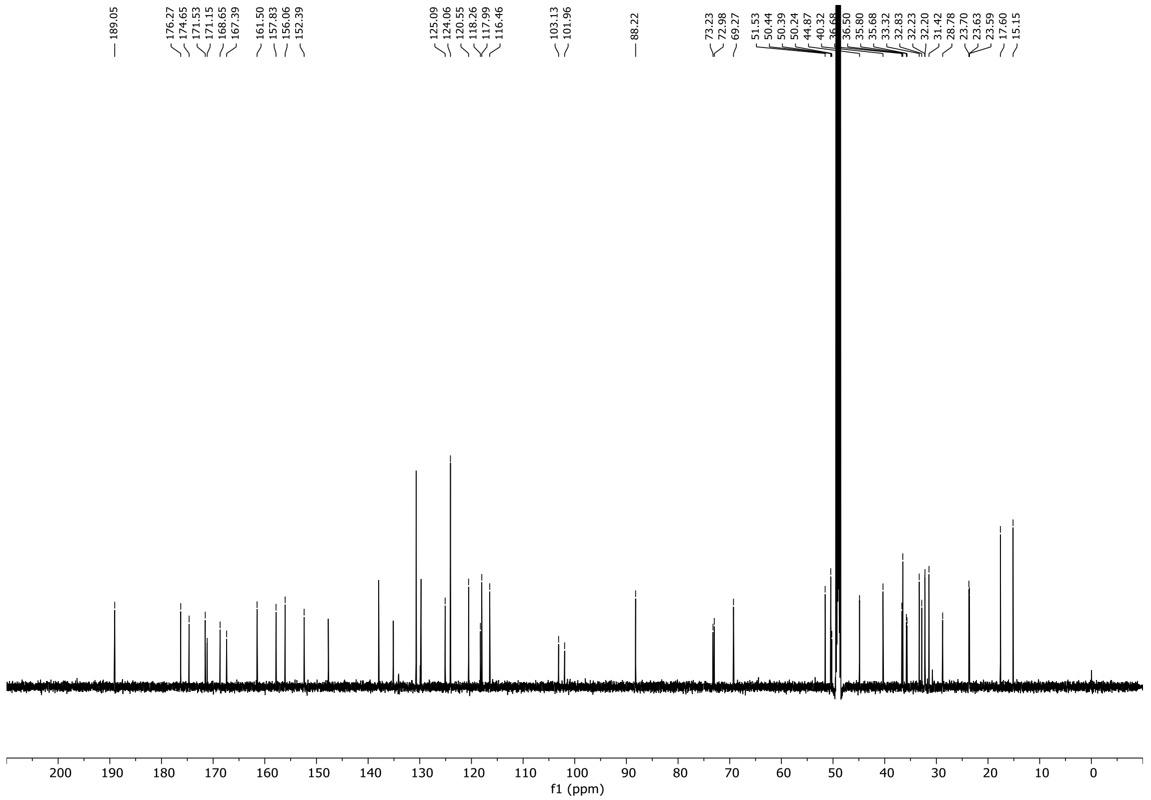

^{19}F NMR (471 MHz, CD_3OD) of compound **KH-5-168**:

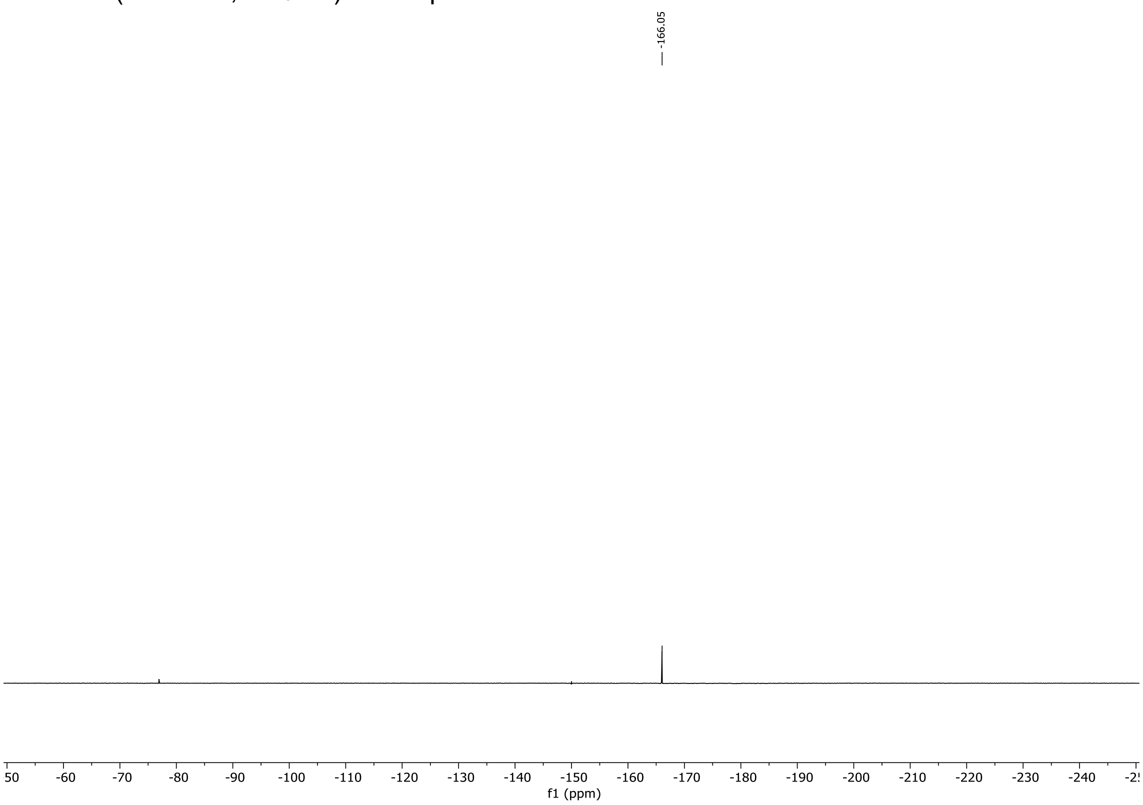

^{1}H NMR (600 MHz, CD_3OD) of compound **KH-5-169**:

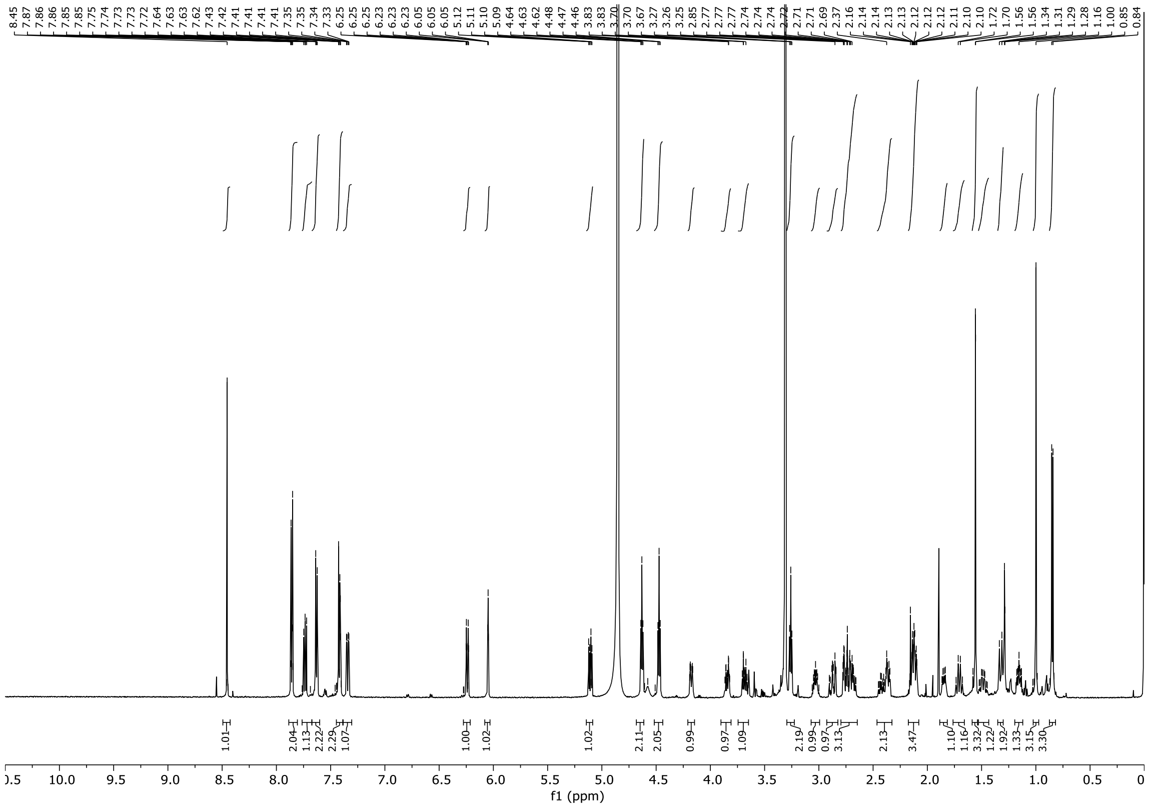

^{13}C NMR (150 MHz, CD_3OD) of compound **KH-5-169**:

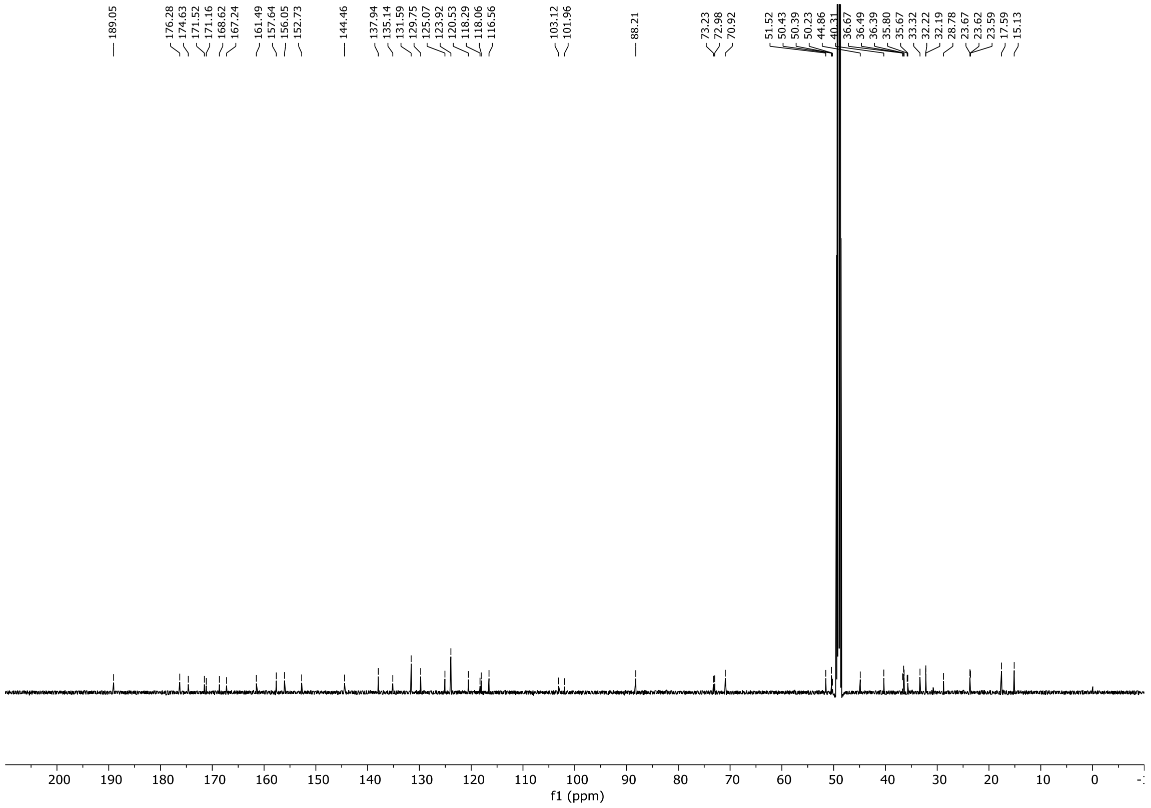

^{19}F NMR (471 MHz, CD_3OD) of compound **KH-5-169**:

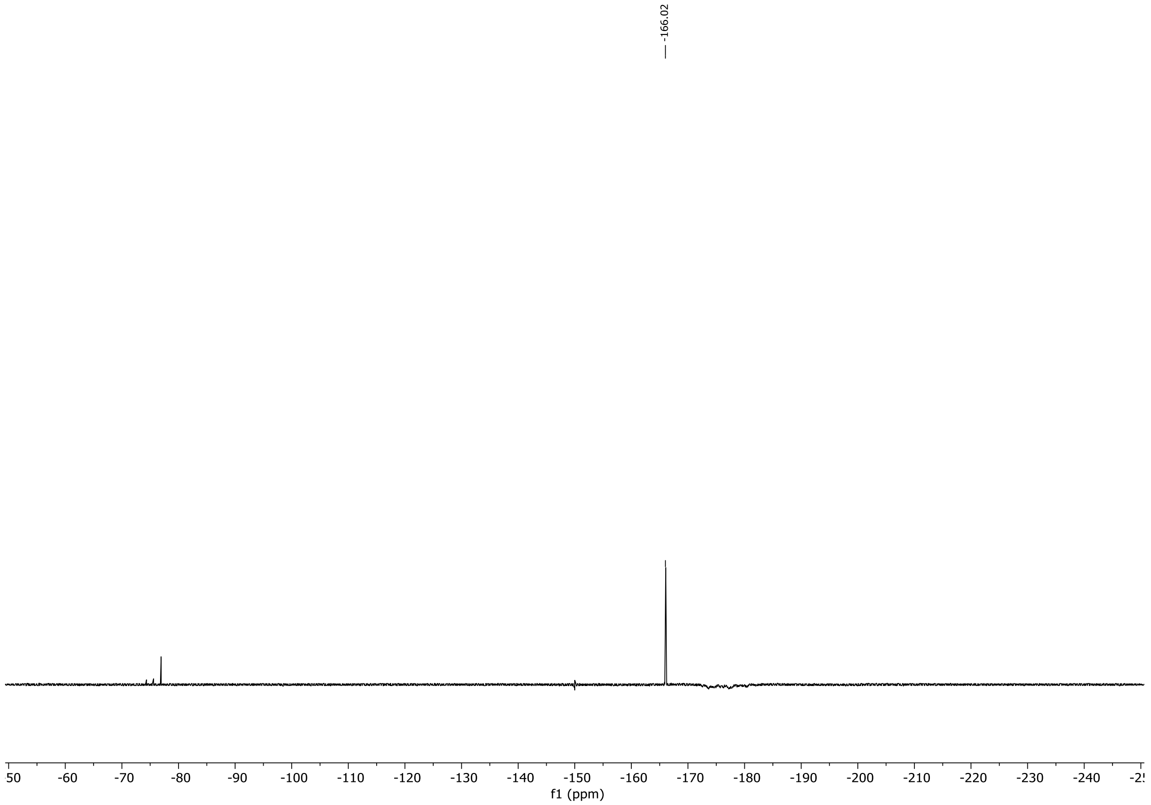

^{1}H NMR (600 MHz, CD_3OD) of compound **KH-5-170**:

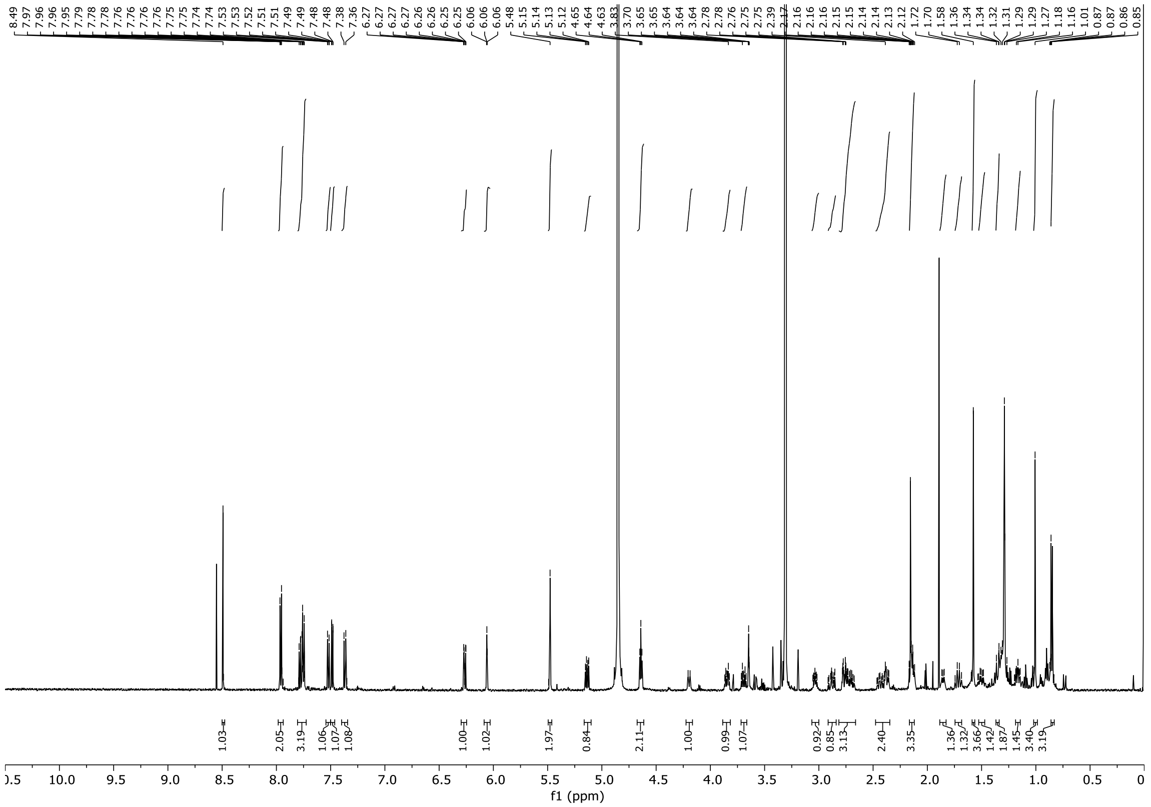

^{13}C NMR (150 MHz, CD_3OD) of compound **KH-5-170**:

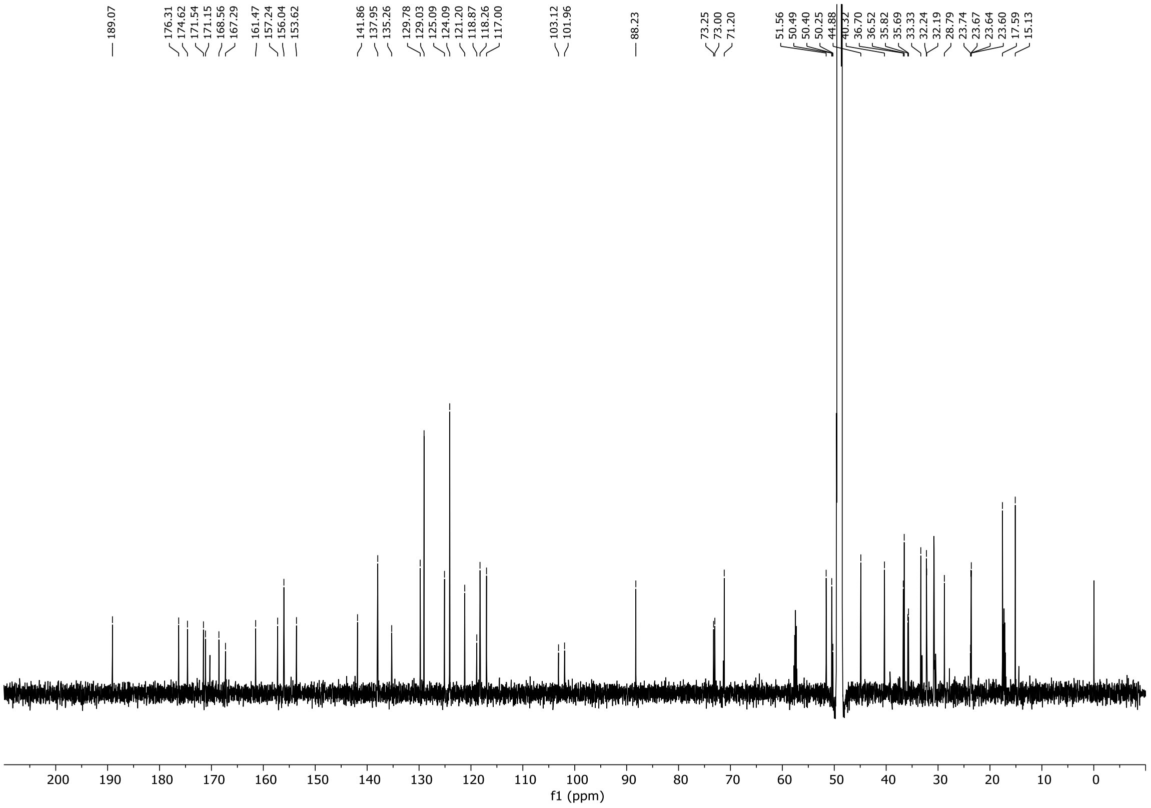

^{19}F NMR (471 MHz, CD_3OD) of compound **KH-5-170**:

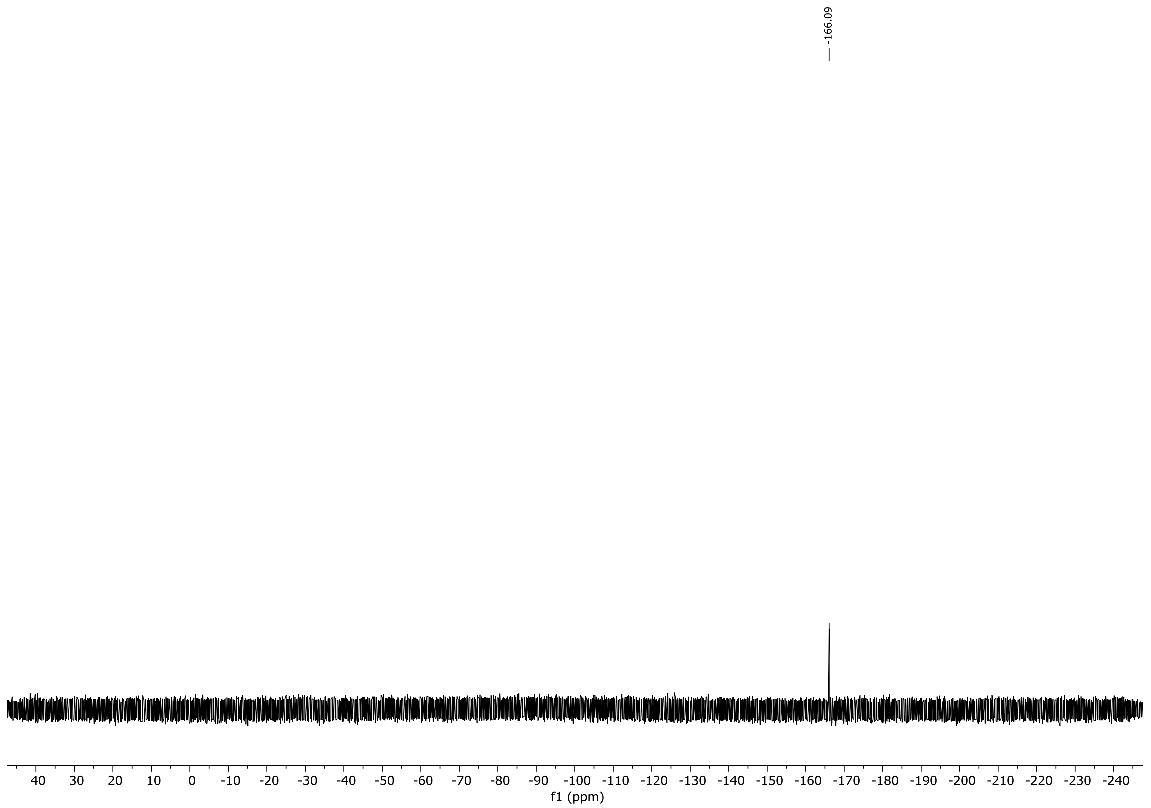

Arylazotriazole–Lenalidomide-Linked photoPROTACs

^{1}H NMR (400 MHz, $CDCl_3$) of compound **22**:

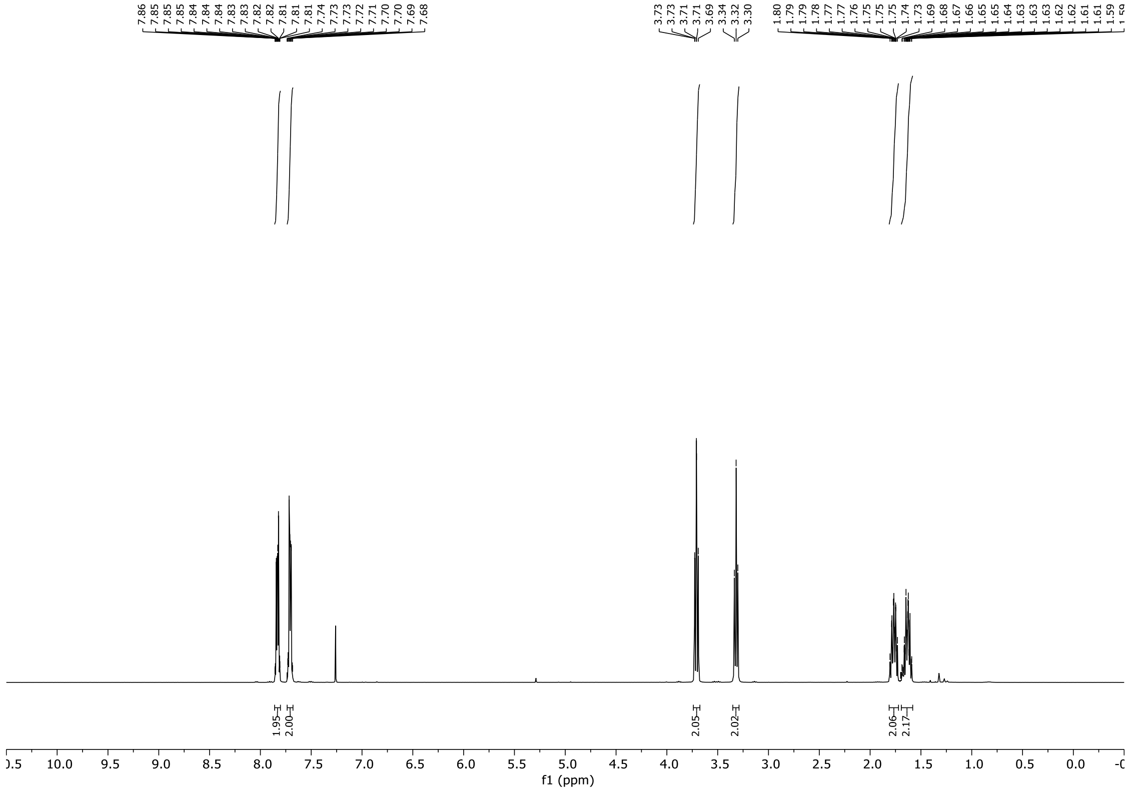

^{13}C NMR (101 MHz, $CDCl_3$) of compound **22**:

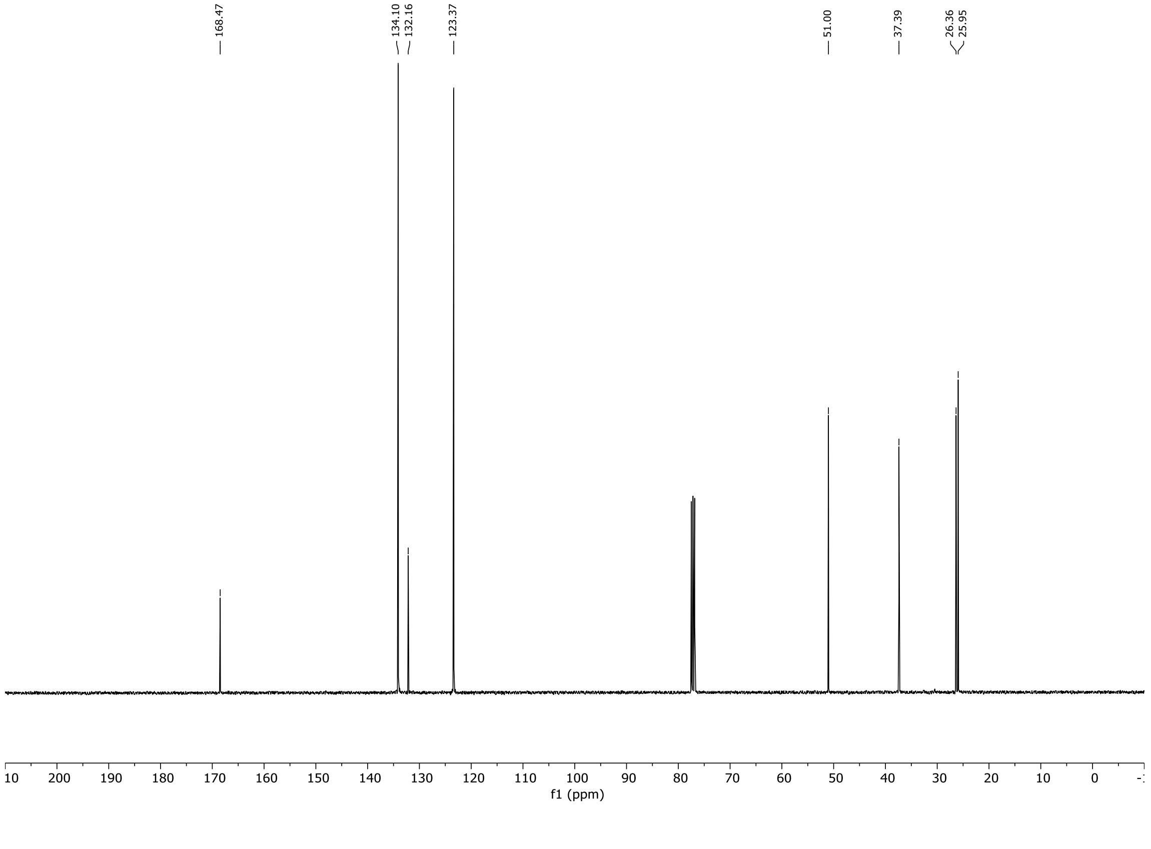

^{1}H NMR (400 MHz, $CDCl_3$) of compound **23**:

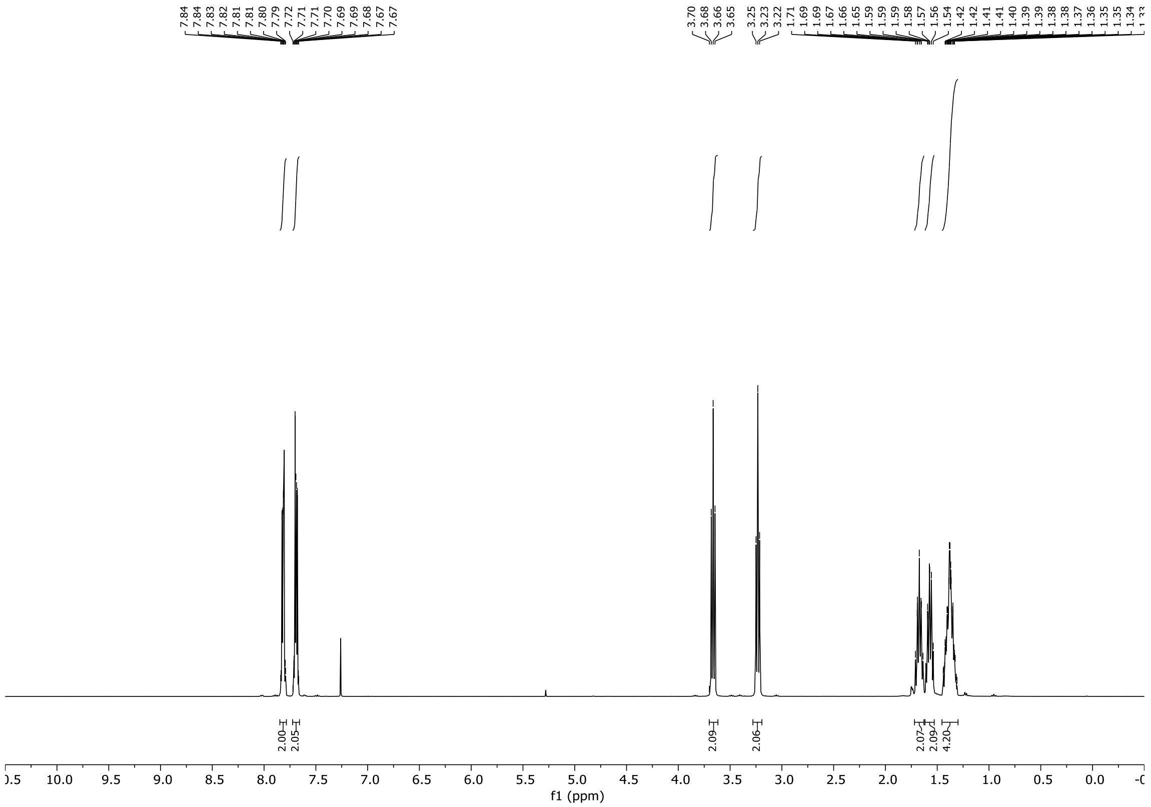

^{13}C NMR (101 MHz, $CDCl_3$) of compound **23**:

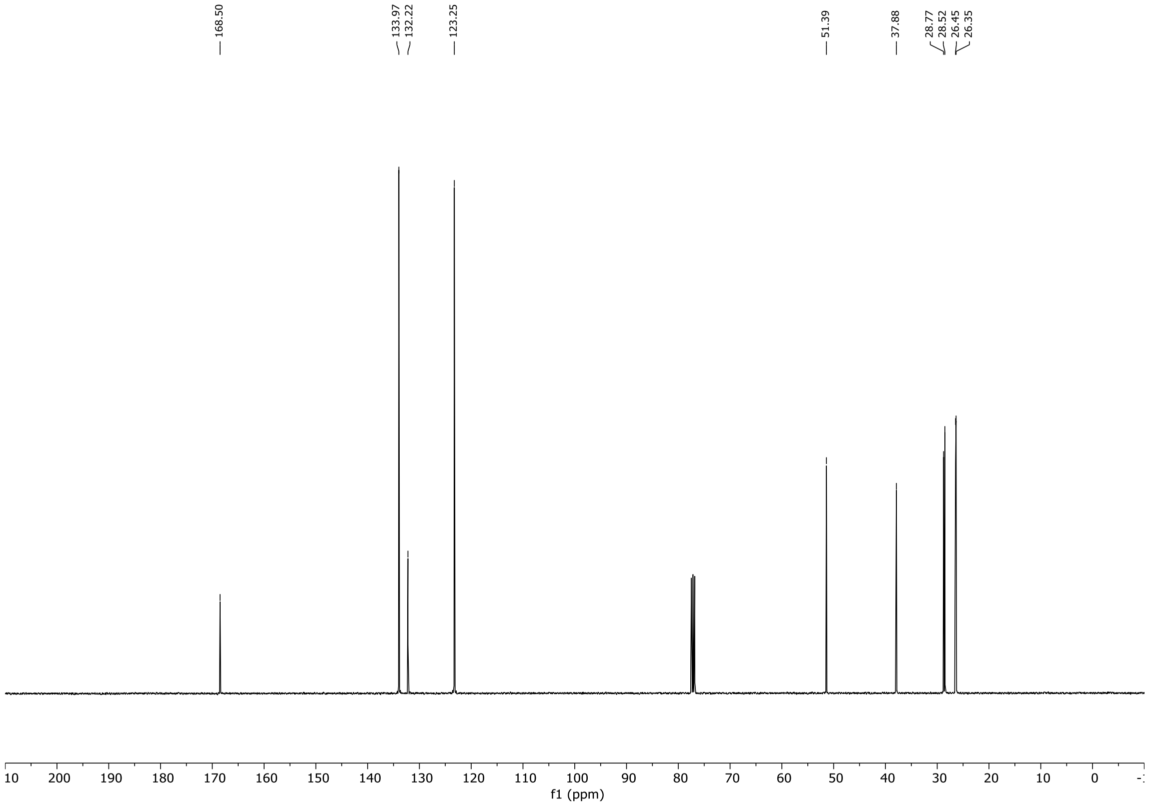

^1H NMR (400 MHz, $CDCl_3$) of compound **24**:

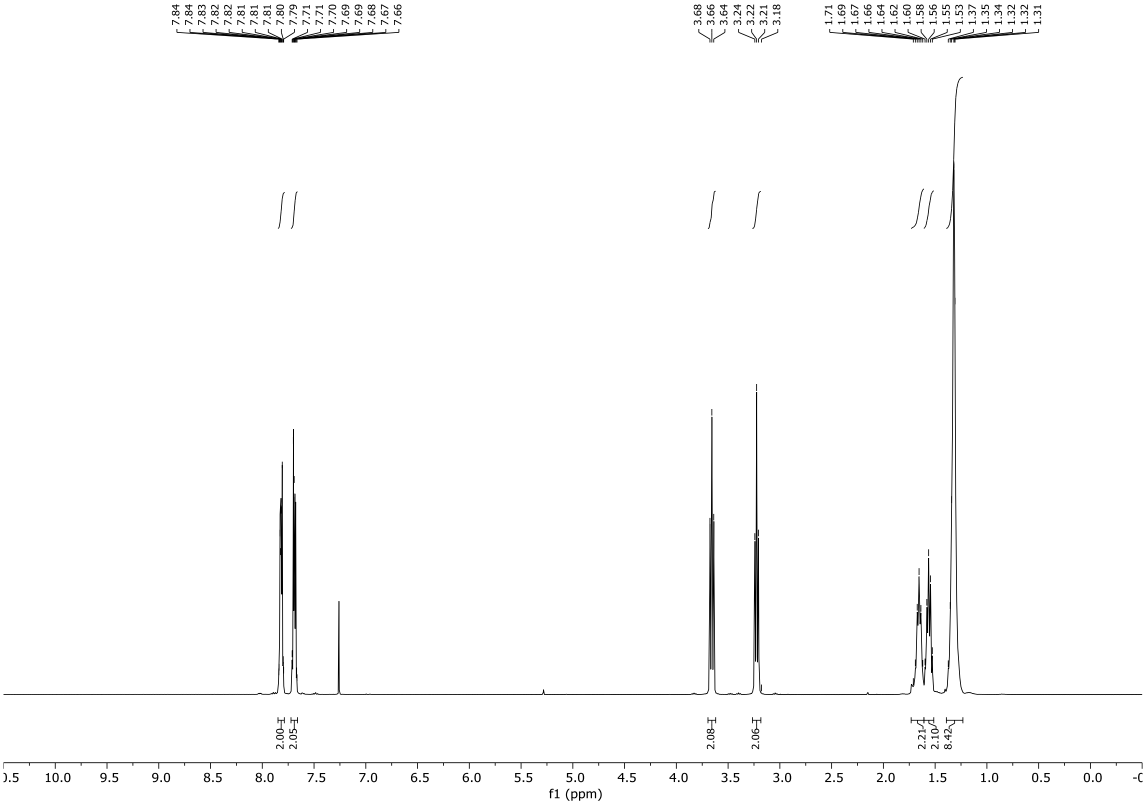

^{13}C NMR (101 MHz, $CDCl_3$) of compound **24**:

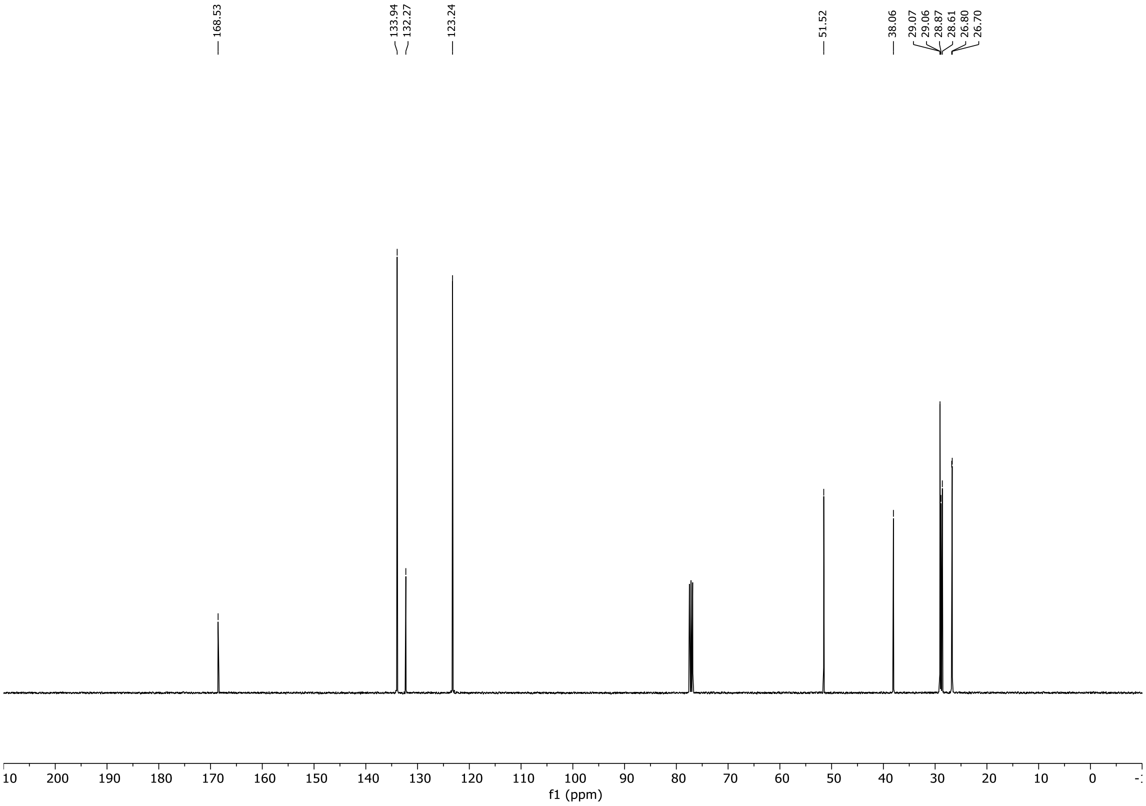

^1H NMR (400 MHz, $CDCl_3$) of compound **25**:

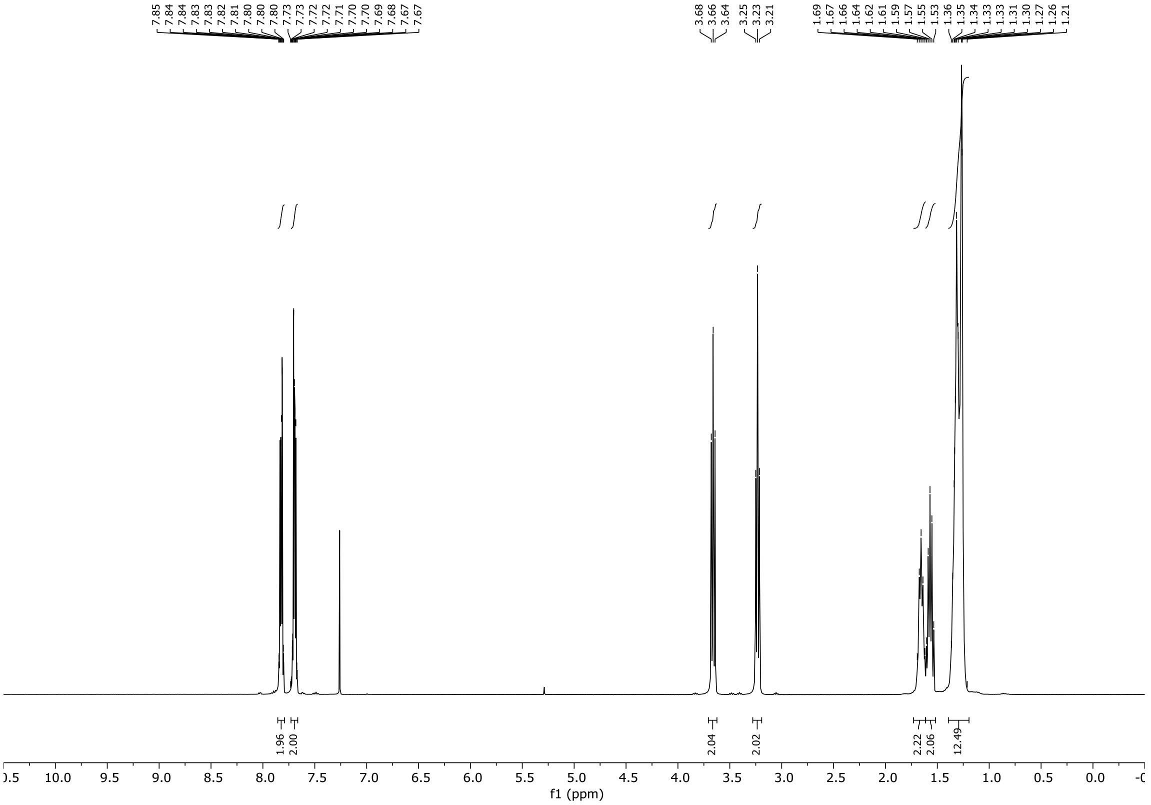

^{13}C NMR (101 MHz, $CDCl_3$) of compound **25**:

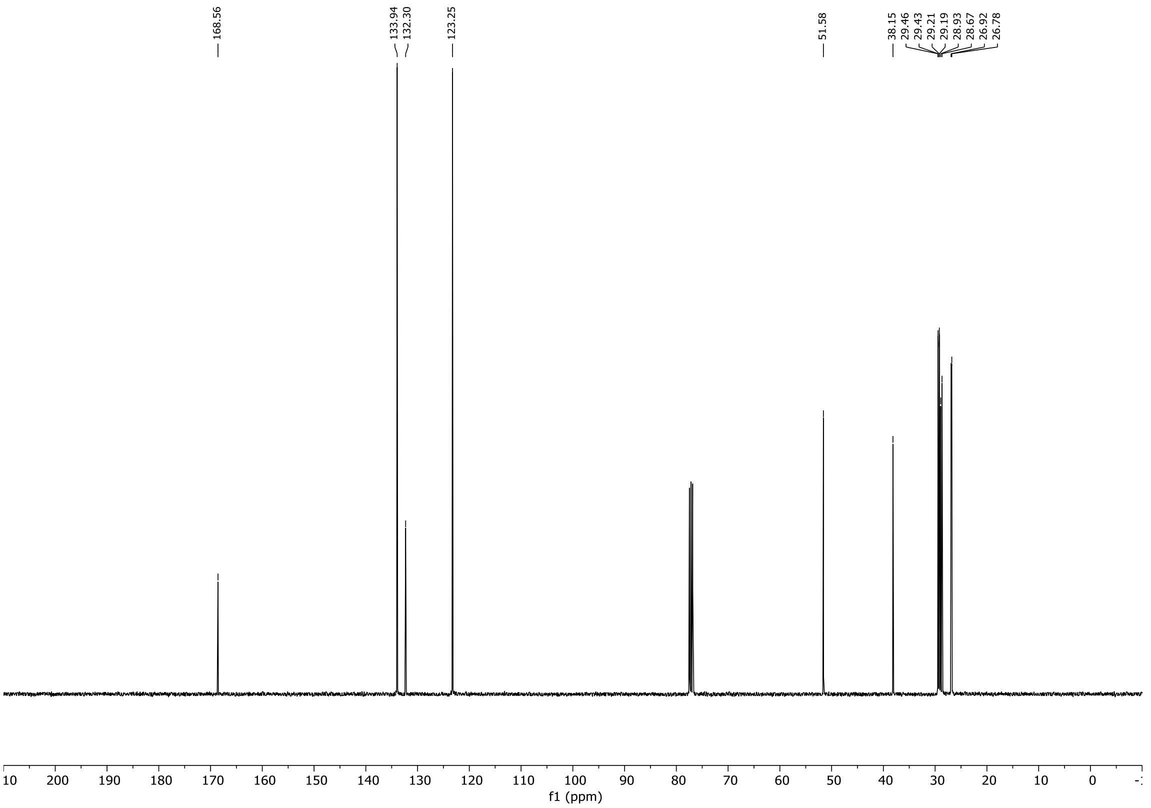

^{1}H NMR (400 MHz, $CDCl_3$) of compound **30**:

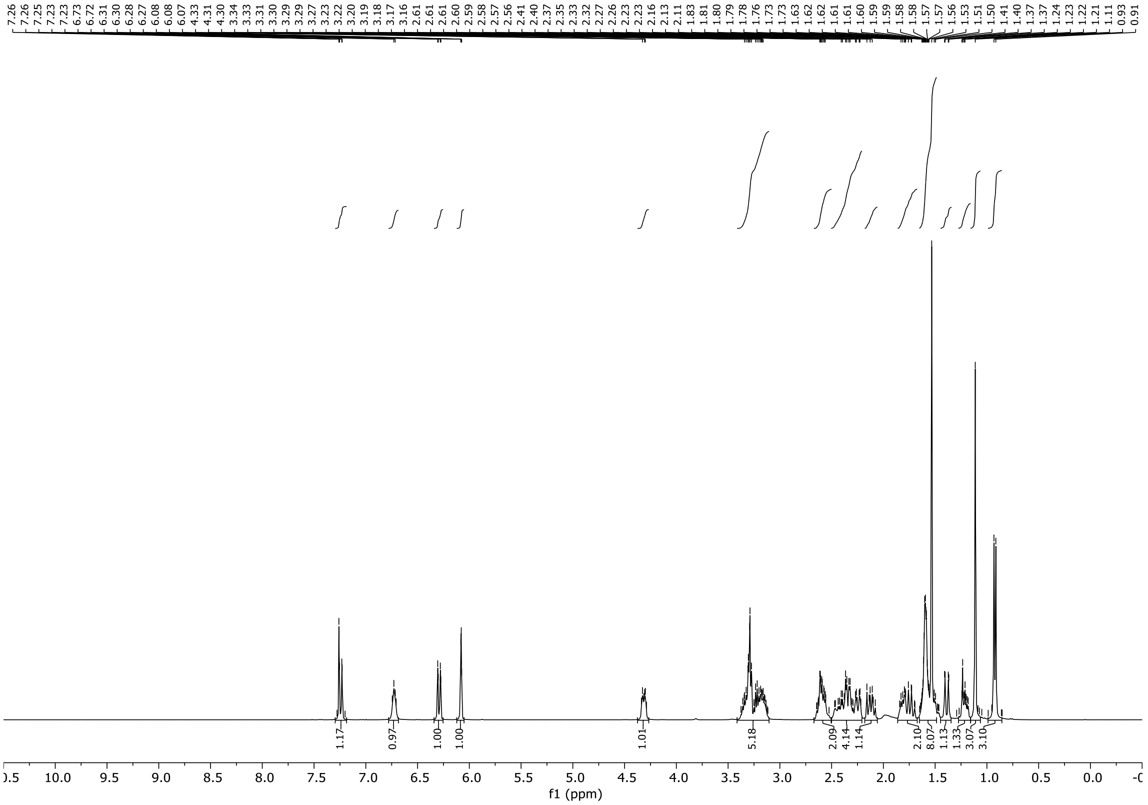

^{13}C NMR (101 MHz, $CDCl_3$) of compound **30**:

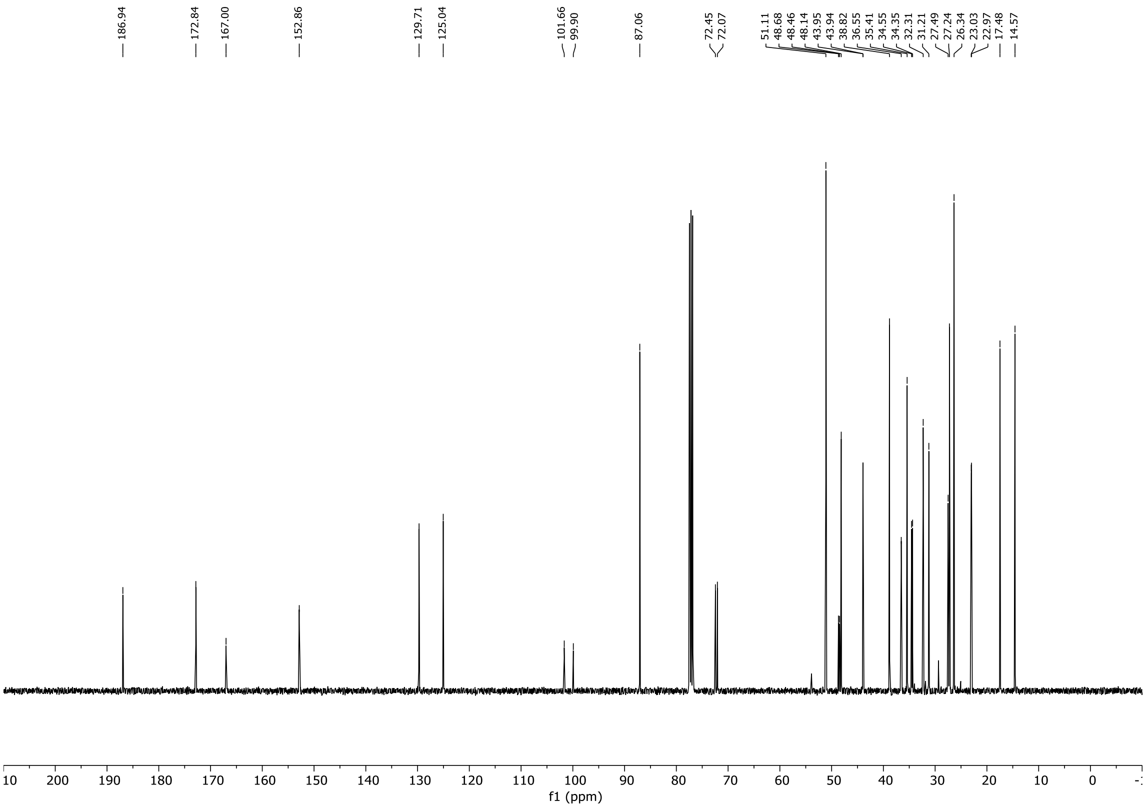

^{19}F NMR (376 MHz, CDCl3) of compound **30**:

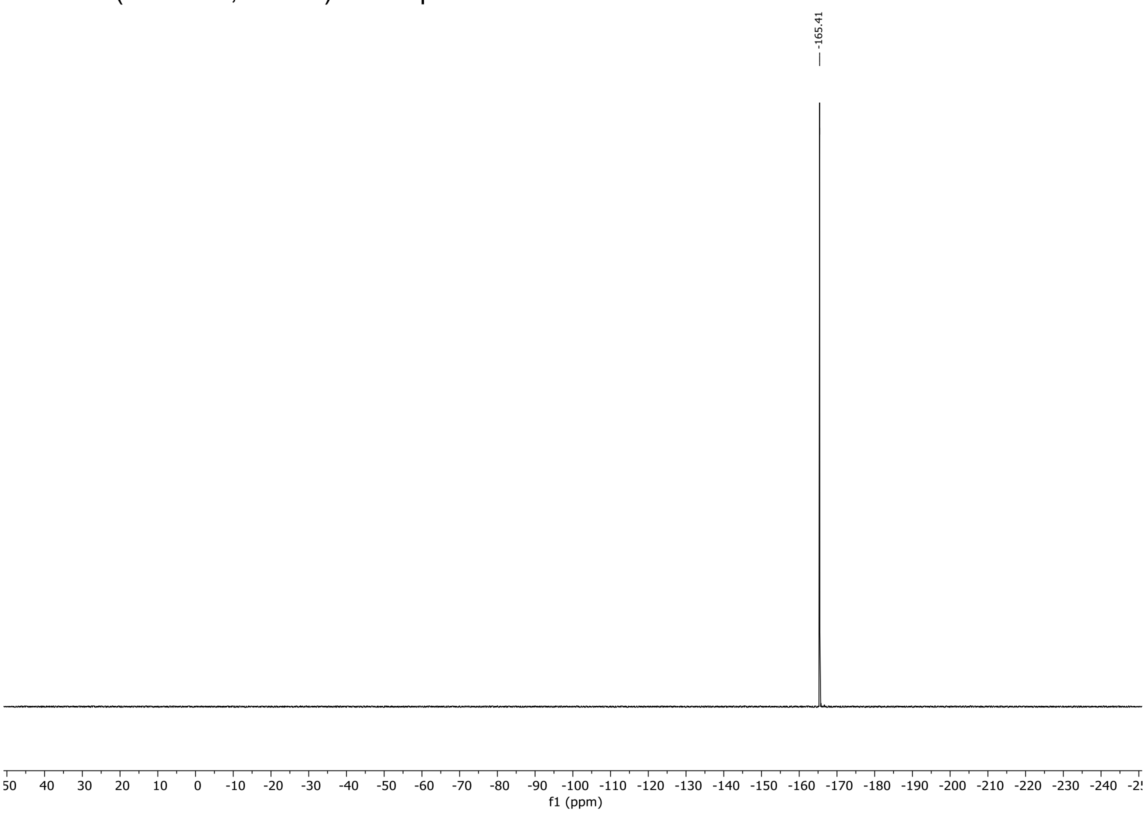

^{1}H NMR (400 MHz, $CDCl_3$) of compound **31**:

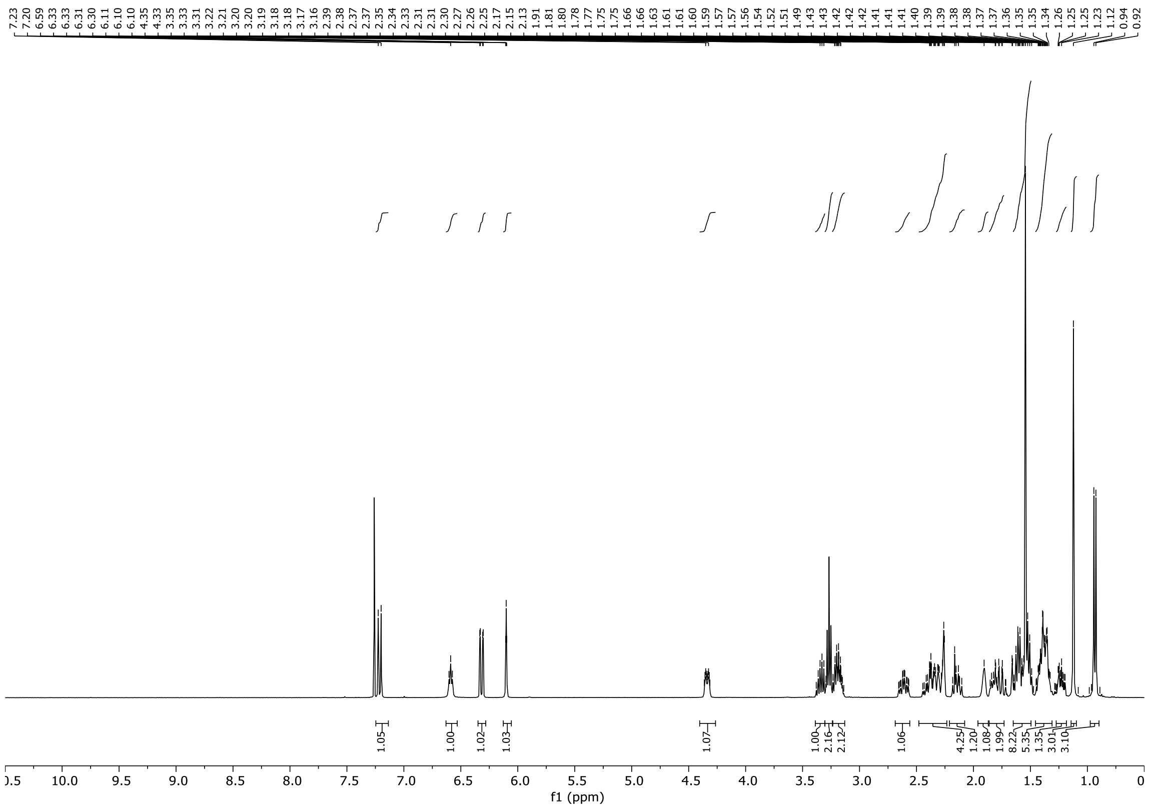

^{13}C NMR (101 MHz, $CDCl_3$) of compound **31**:

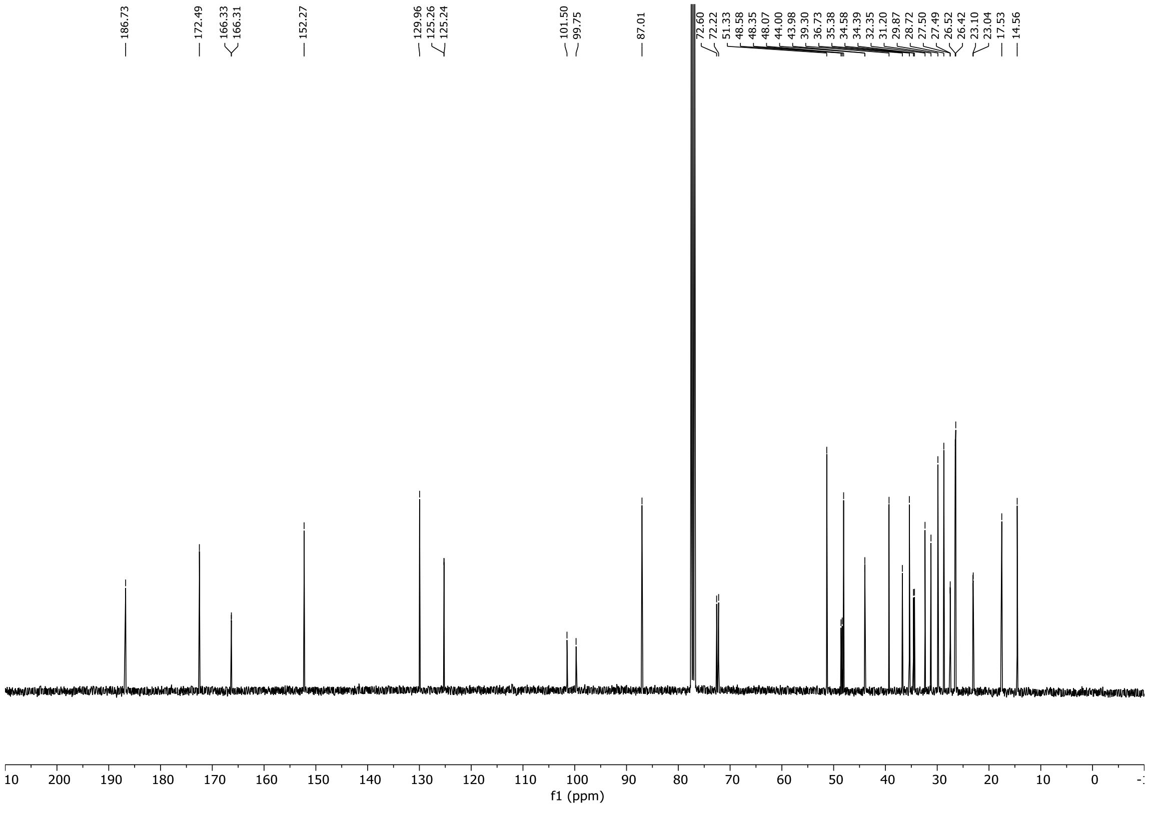

^{19}F NMR (376 MHz, CDCl3) of compound **31**:

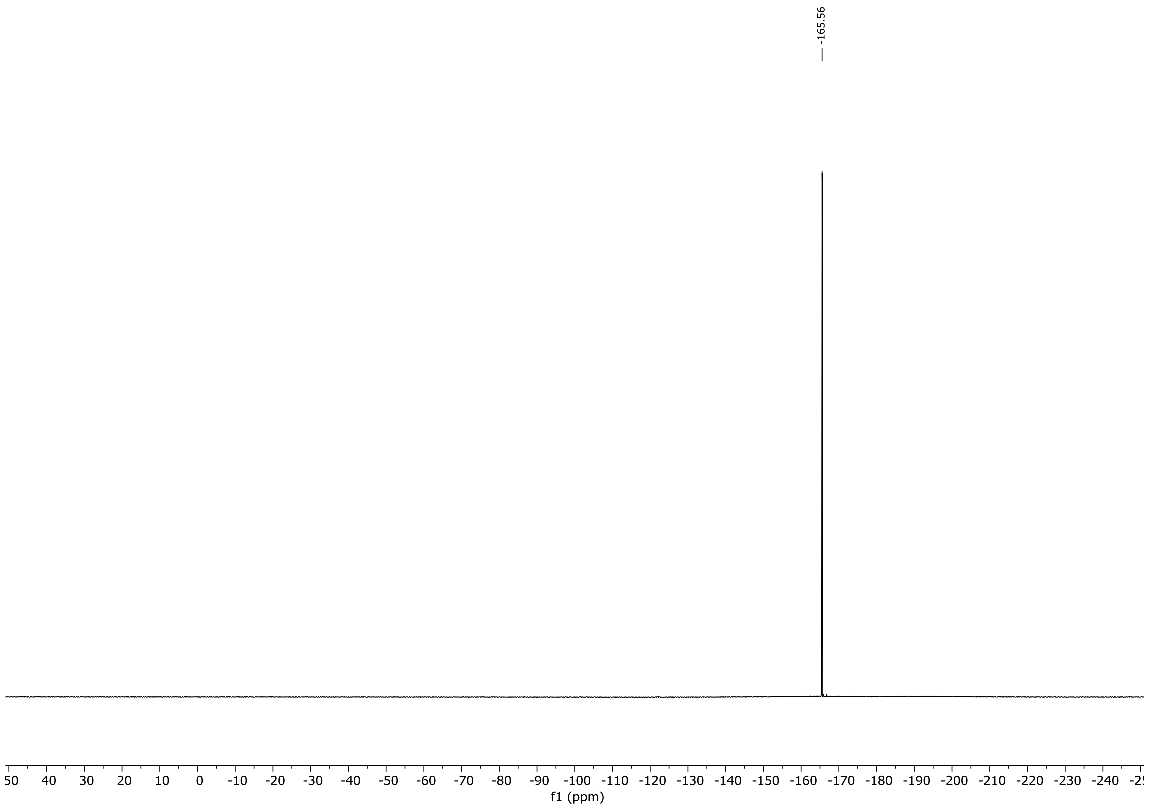

^{1}H NMR (400 MHz, $CDCl_3$) of compound **32**:

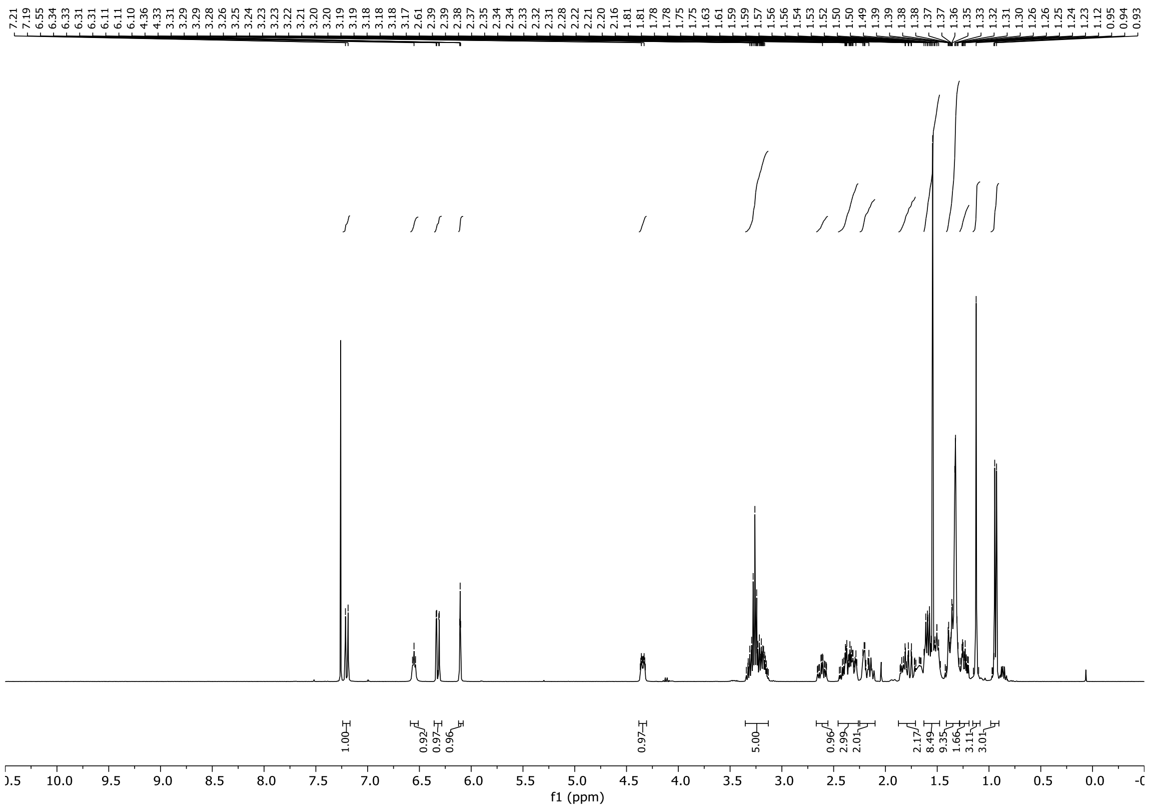

^{13}C NMR (101 MHz, $CDCl_3$) of compound **32**:

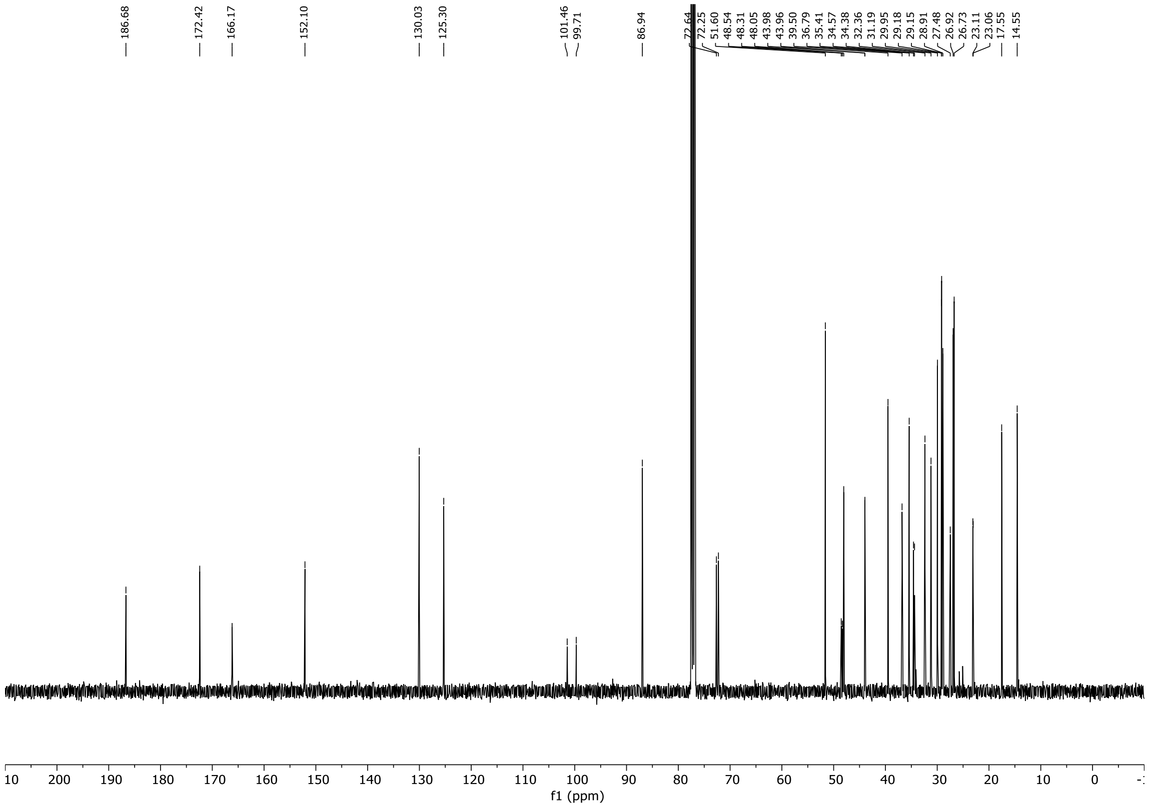

^{19}F NMR (377 MHz, CDCl3) of compound **32**:

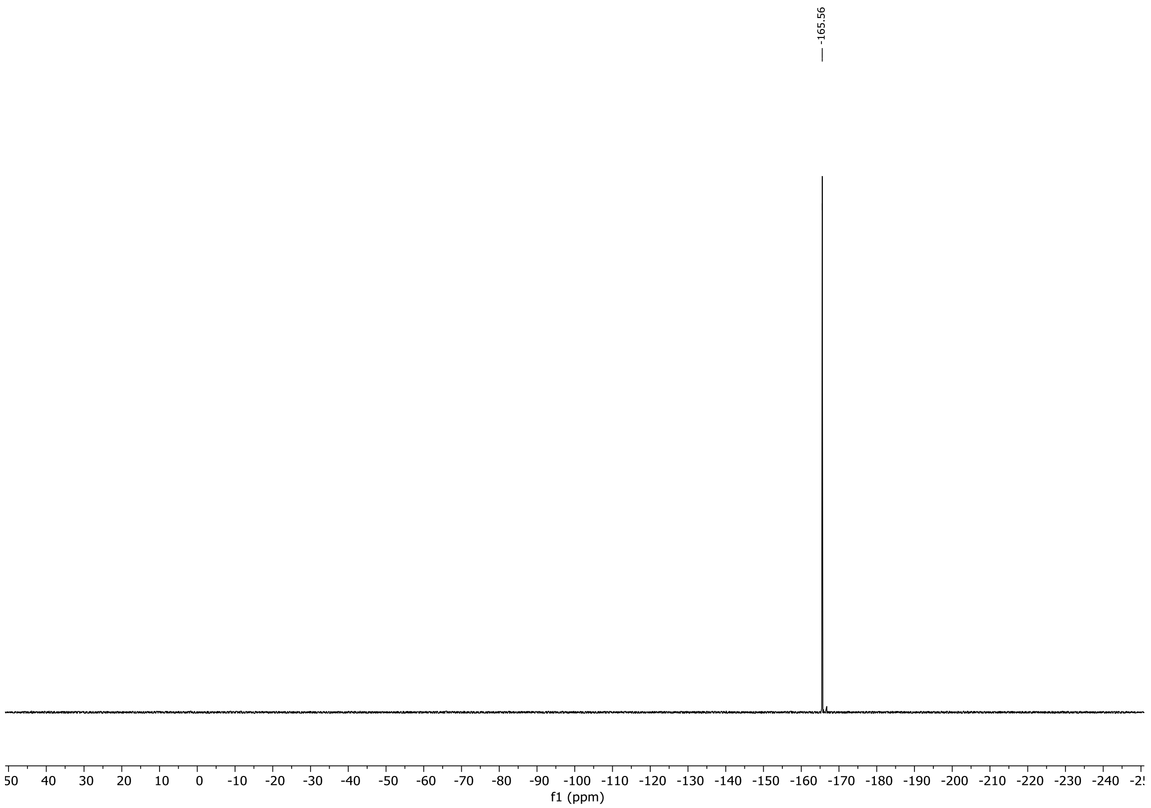

^1H NMR (400 MHz, $CDCl_3$) of compound **33**:

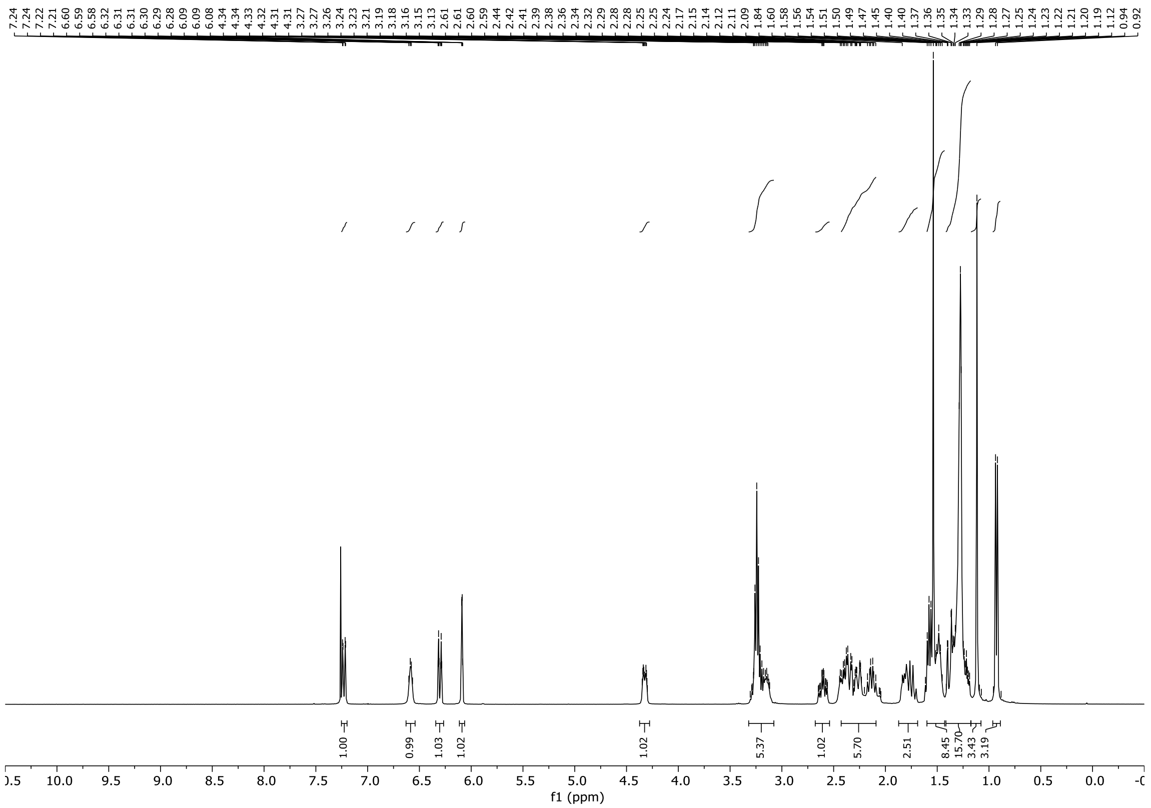

^{13}C NMR (101 MHz, $CDCl_3$) of compound **33**:

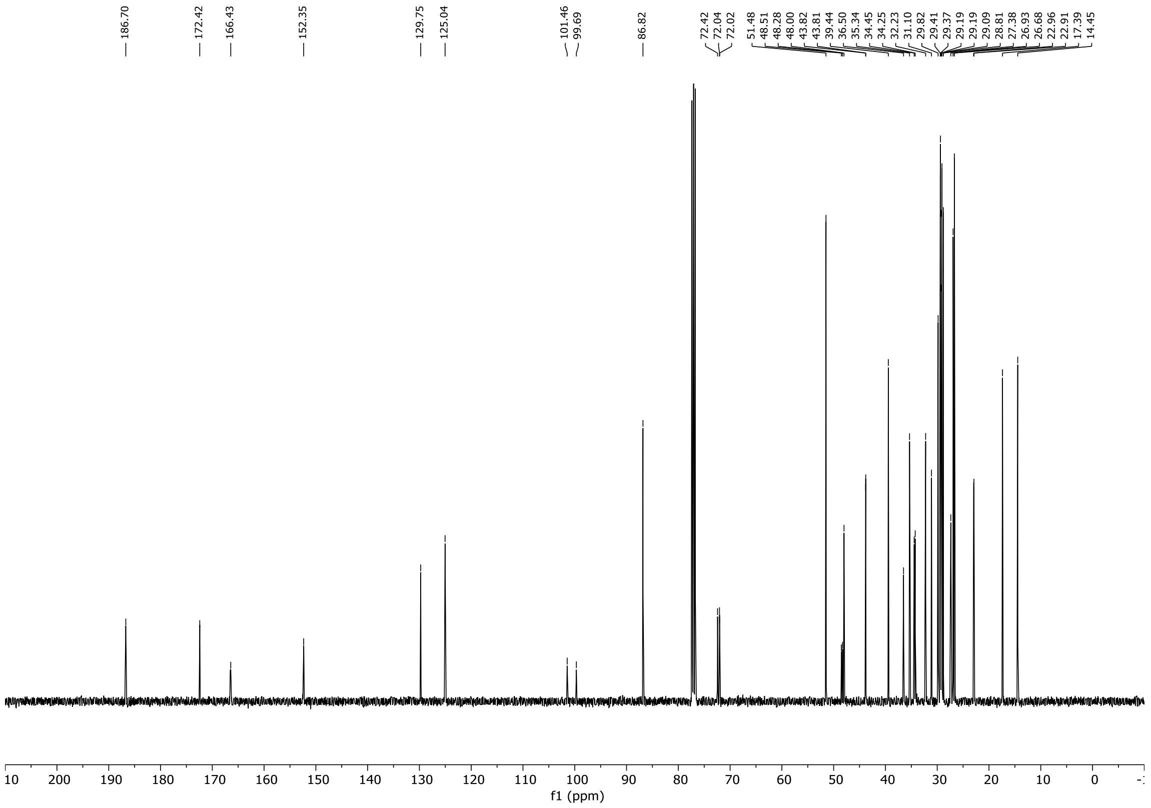

^{19}F NMR (377 MHz, $(CD_3)_2CO$) of compound **33**:

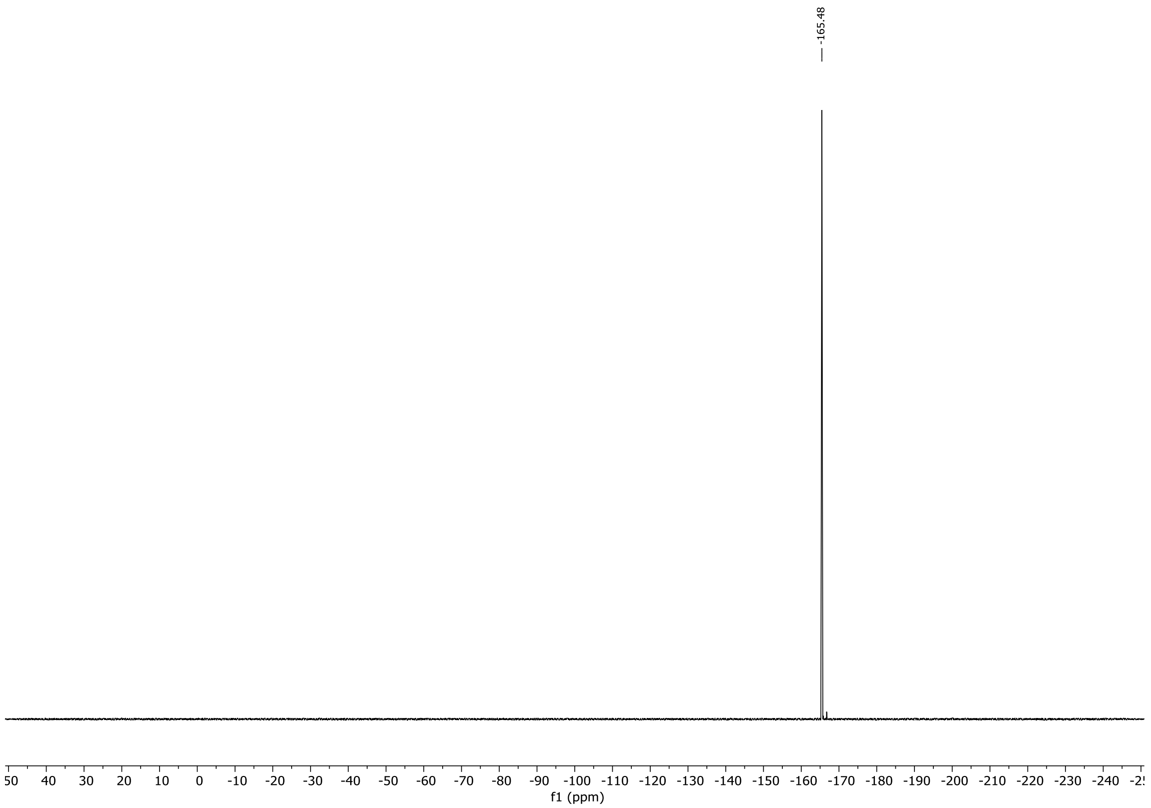

^{1}H NMR (400 MHz, CD_3CN) of compound **35**:

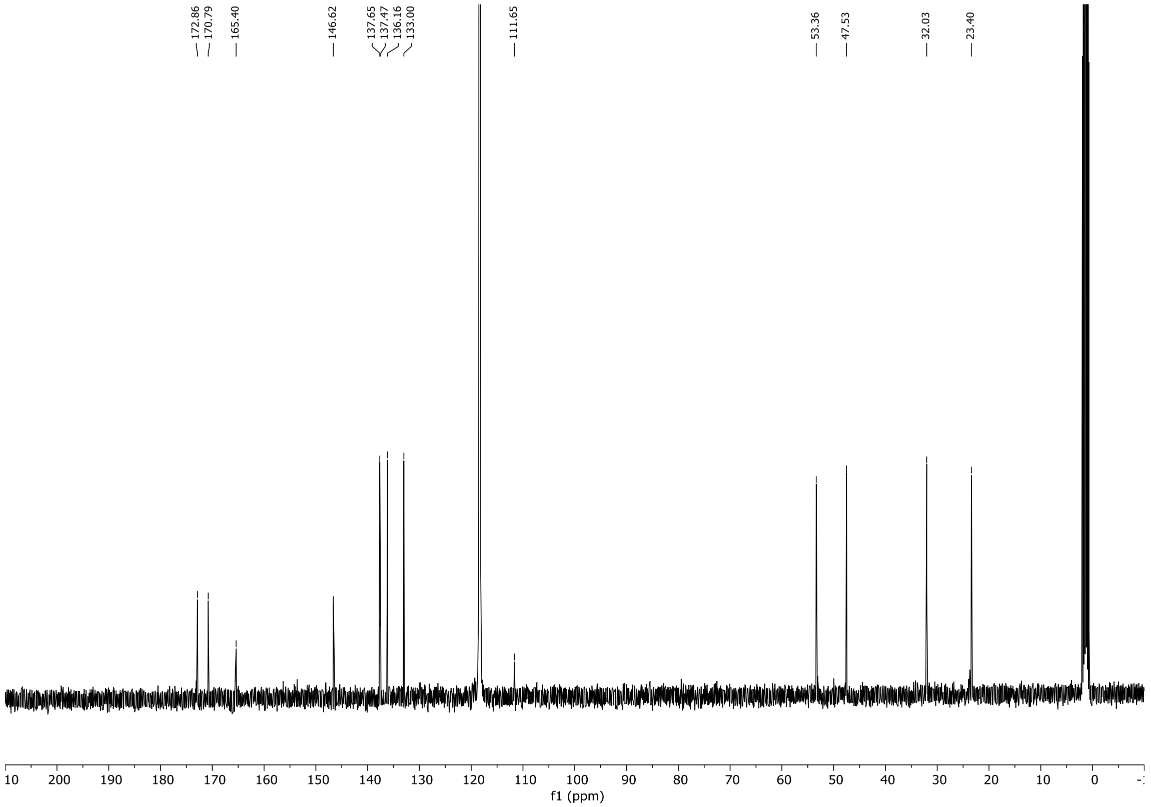

^{13}C NMR (101 MHz, CD_3CN) of compound **35**:

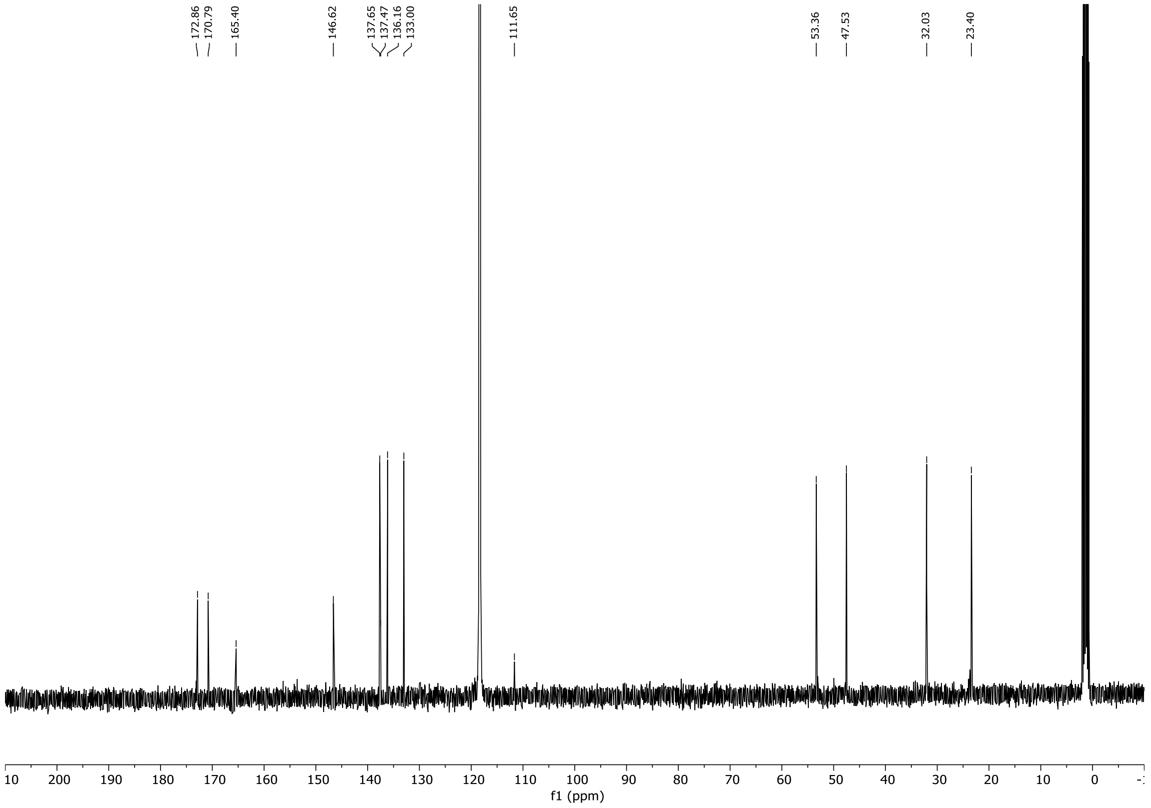

^{19}F NMR (376 MHz, CD_3CN) of compound **35**:

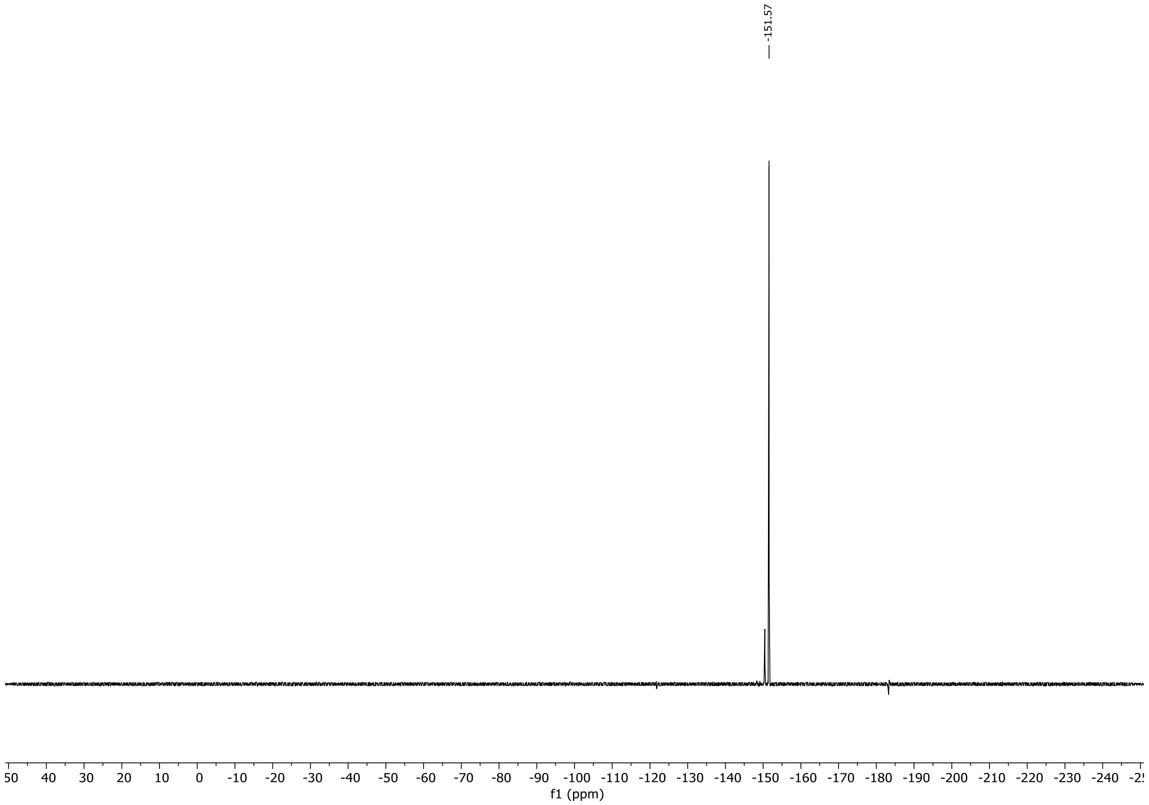

^{1}H NMR (400 MHz, $CDCl_3$) of compound **36**:

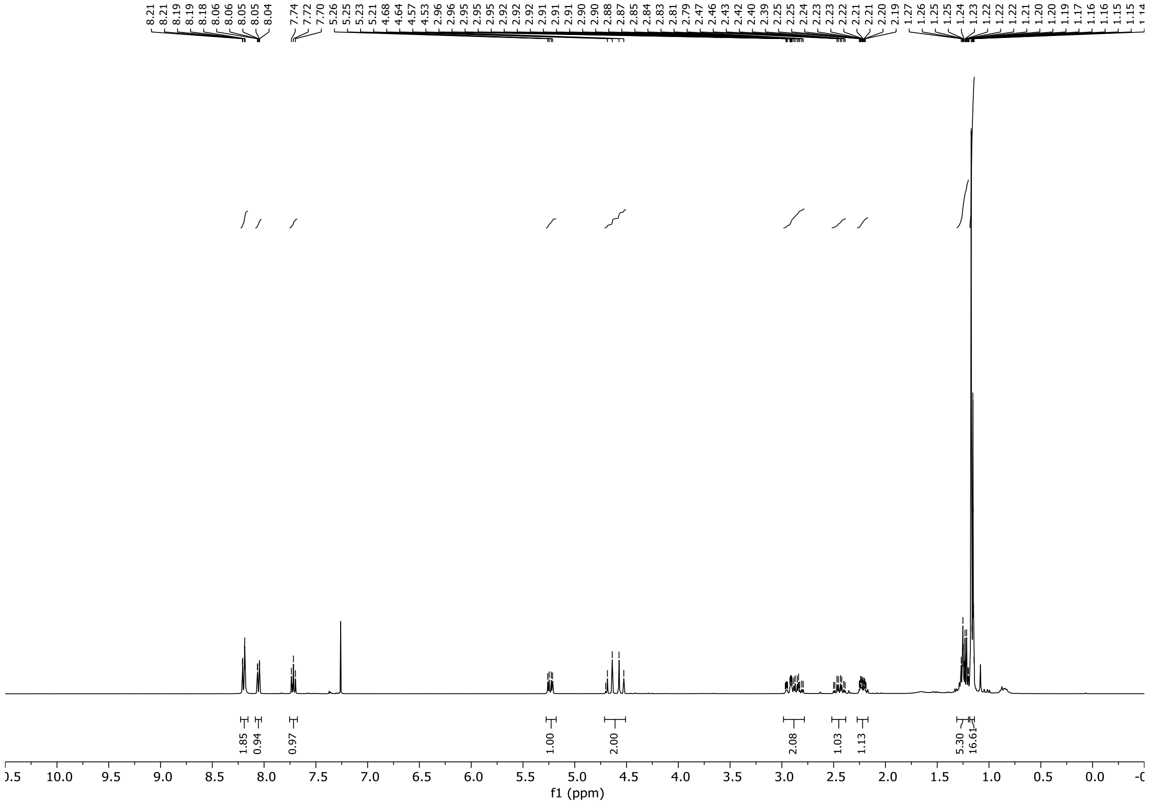

^{13}C NMR (101 MHz, $CDCl_3$) of compound **36**:

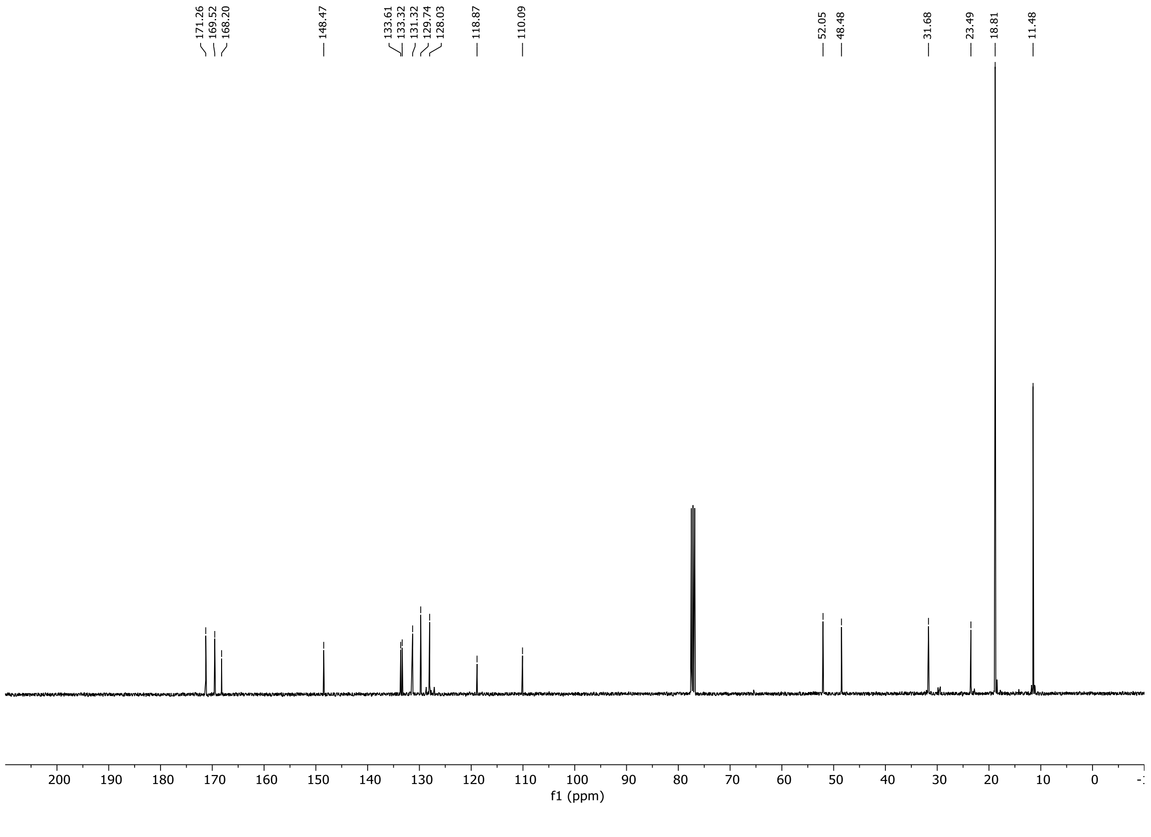

^{1}H NMR (500 MHz, $(CD_3)_2CO$) of compound **KH-5-210**:

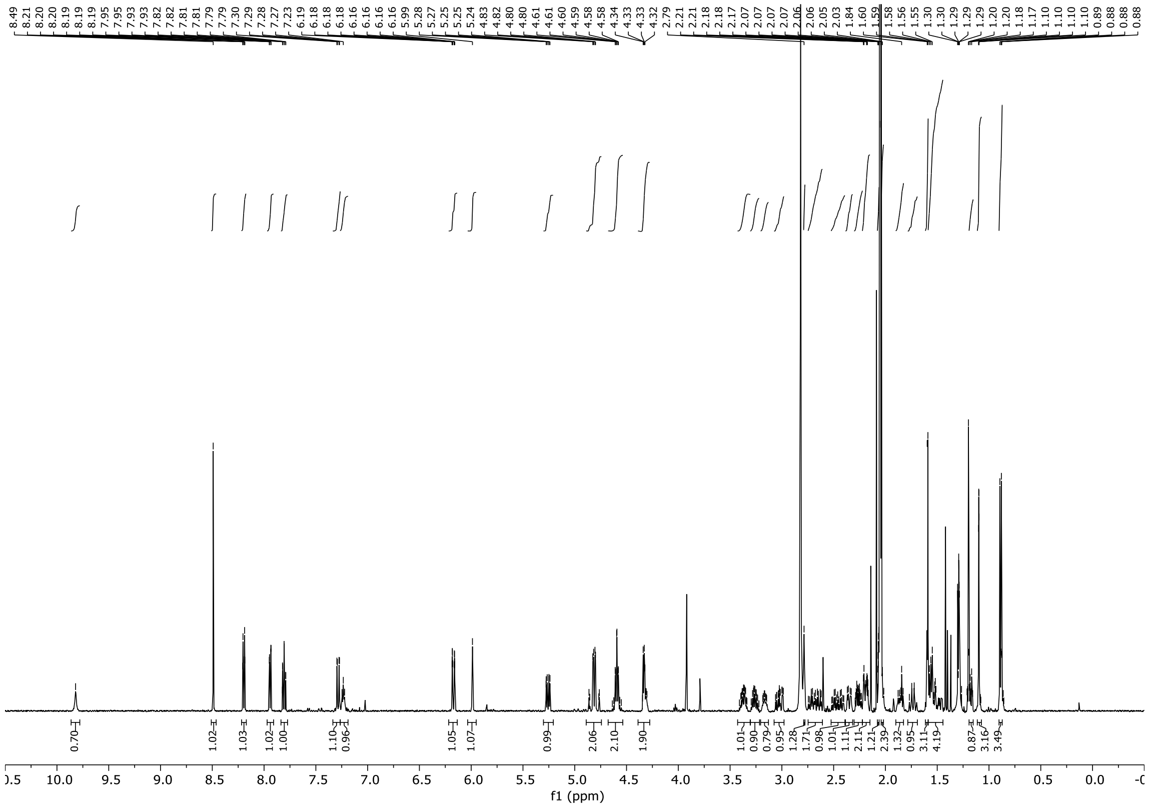

^{13}C NMR (126 MHz, $(CD_3)_2CO$) of compound **KH-5-210**:

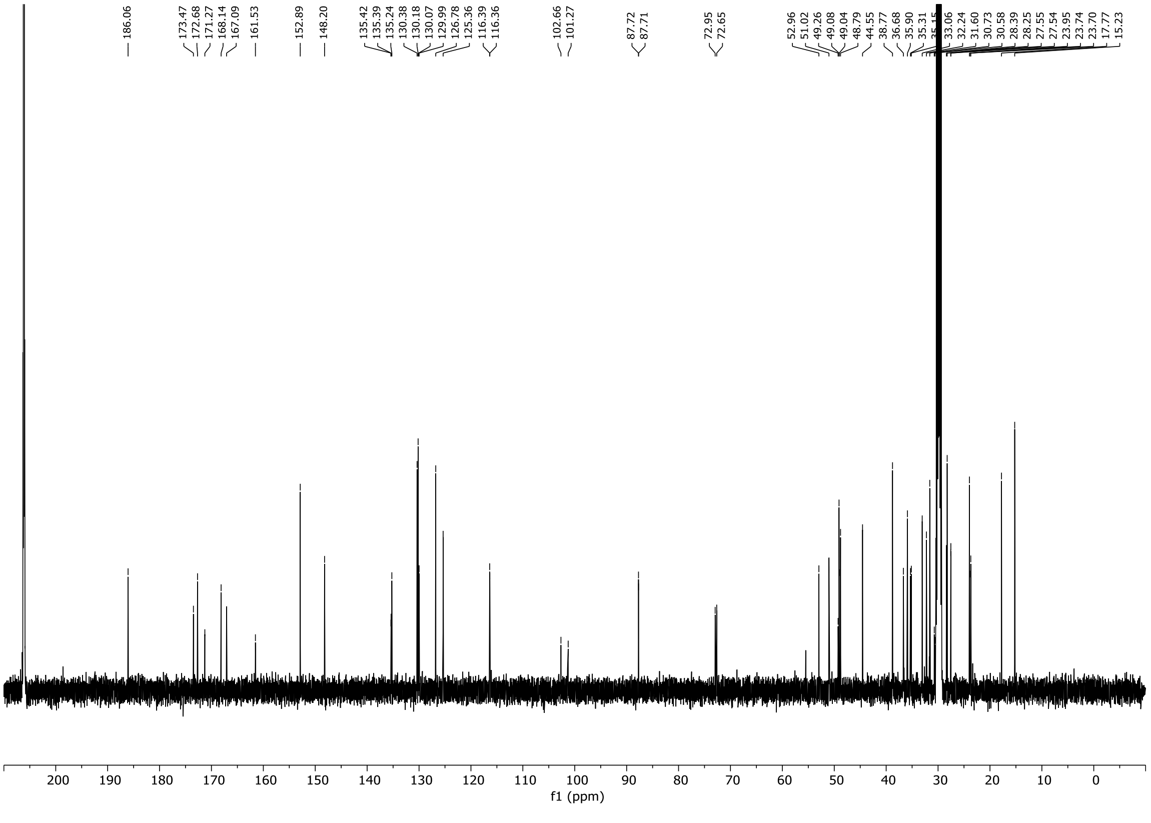

^{19}F NMR (471 MHz, $(CD_3)_2CO$) of compound **KH-5-210**:

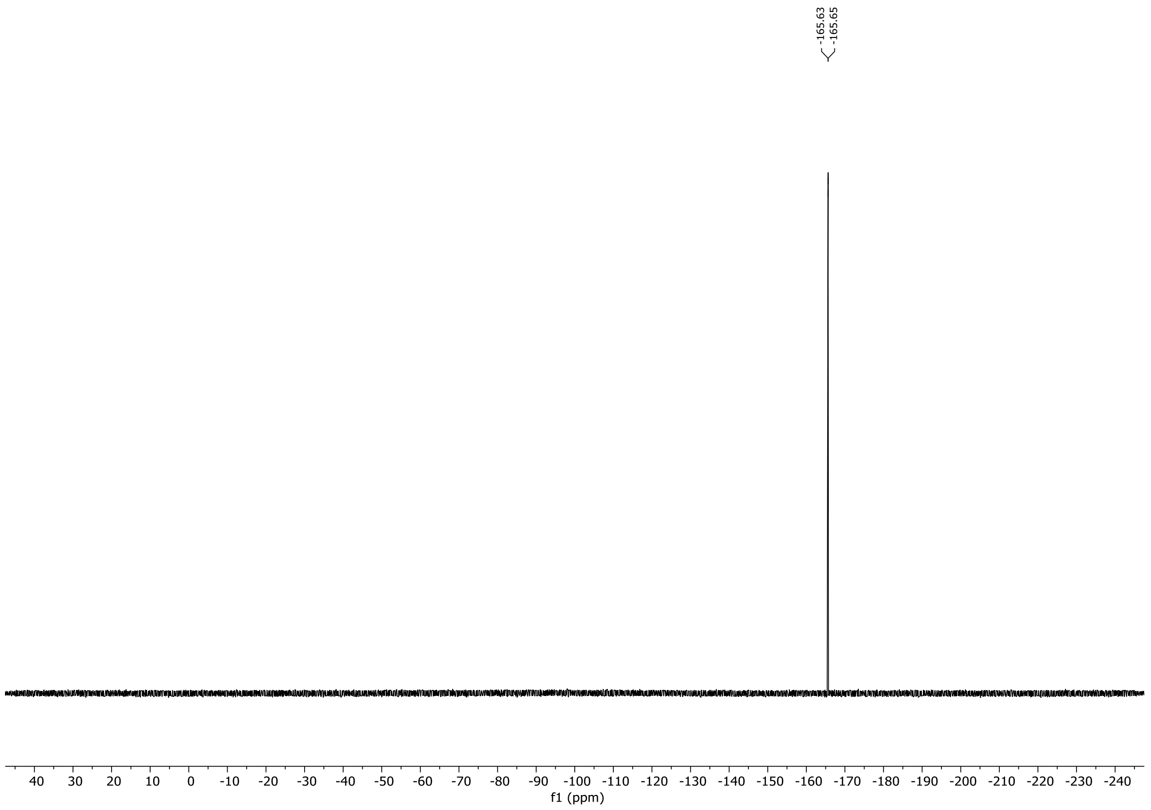

^{1}H NMR (400 MHz, $CDCl_3$) of compound **37**:

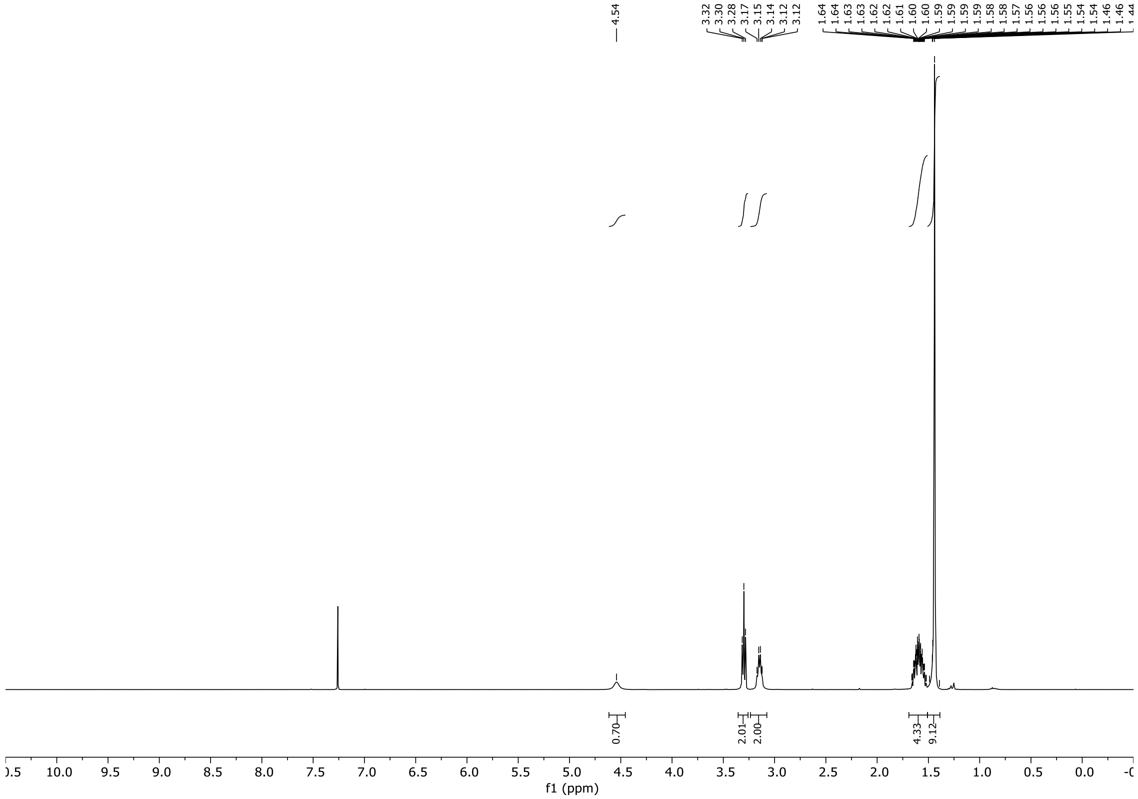

^{13}C NMR (101 MHz, $CDCl_3$) of compound **37**:

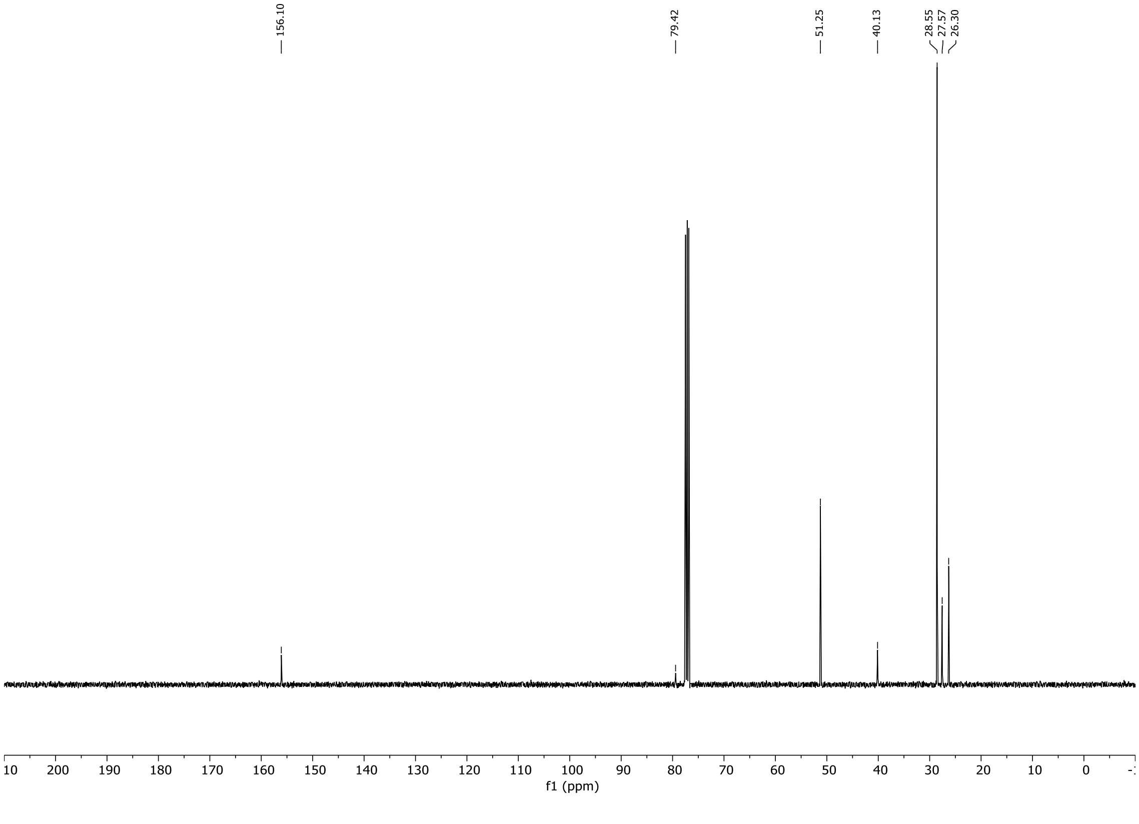

^{1}H NMR (500 MHz, $(CD_3)_2CO$) of compound **38**:

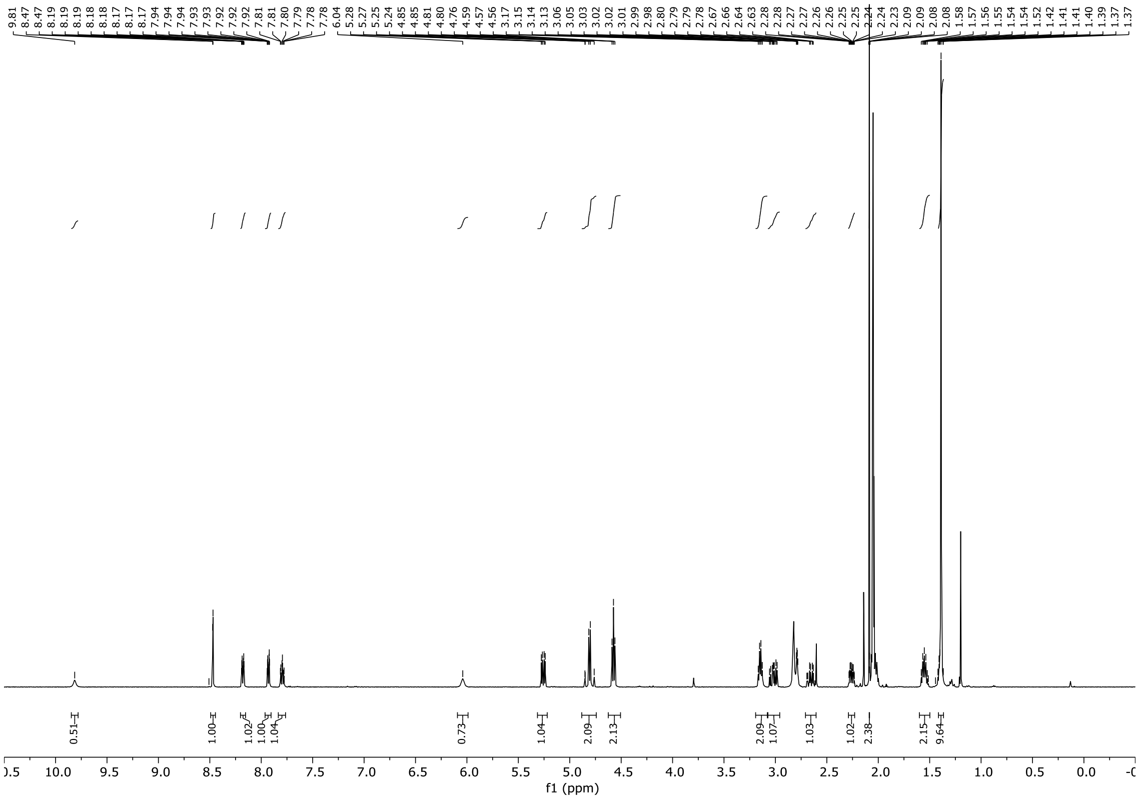

^{13}C NMR (126 MHz, $(CD_3)_2CO$) of compound **38**:

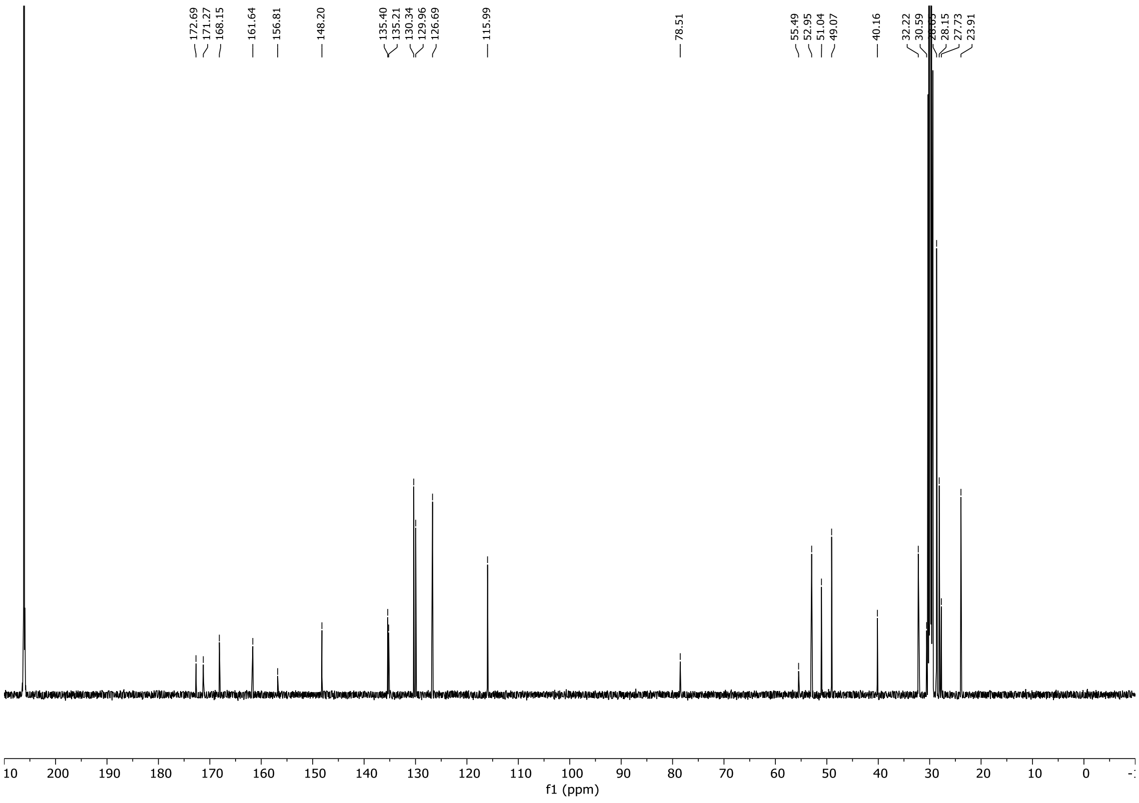

^{1}H NMR (500 MHz, $(CD_3)_2CO$) of compound **KH-5-224**:

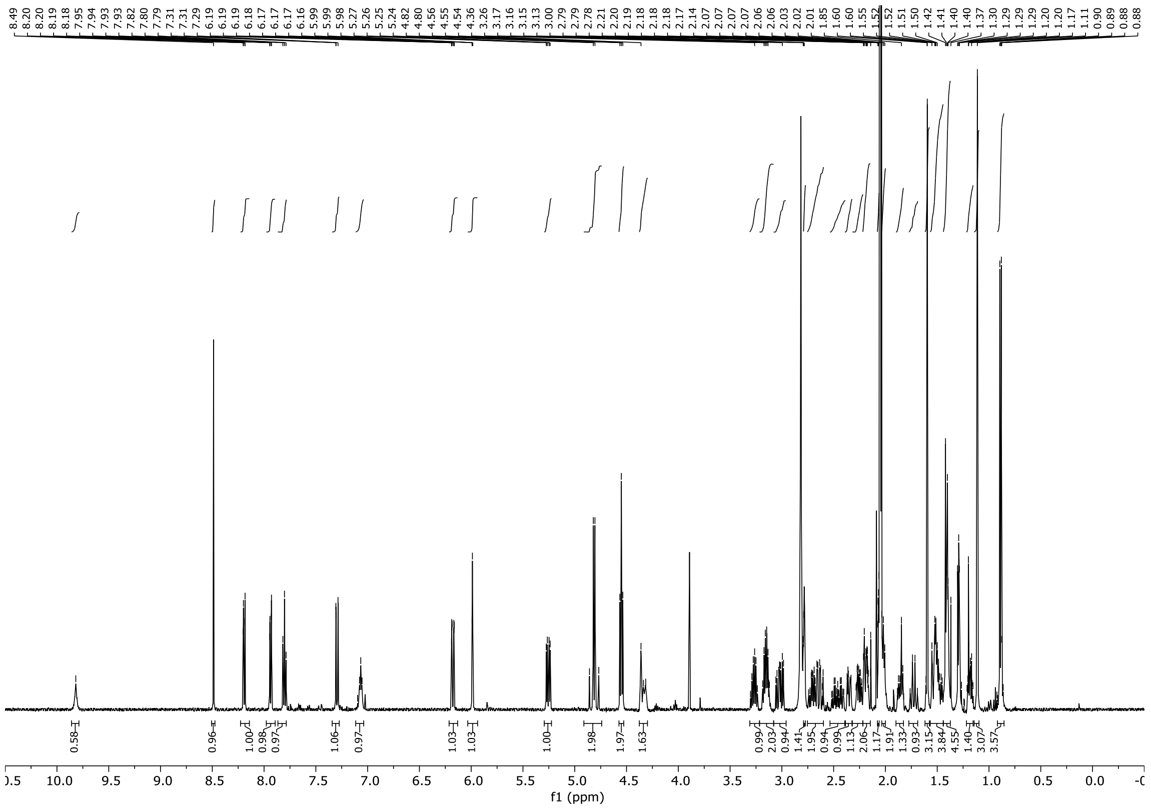

^{13}C NMR (126 MHz, $(CD_3)_2CO$) of compound **KH-5-224**:

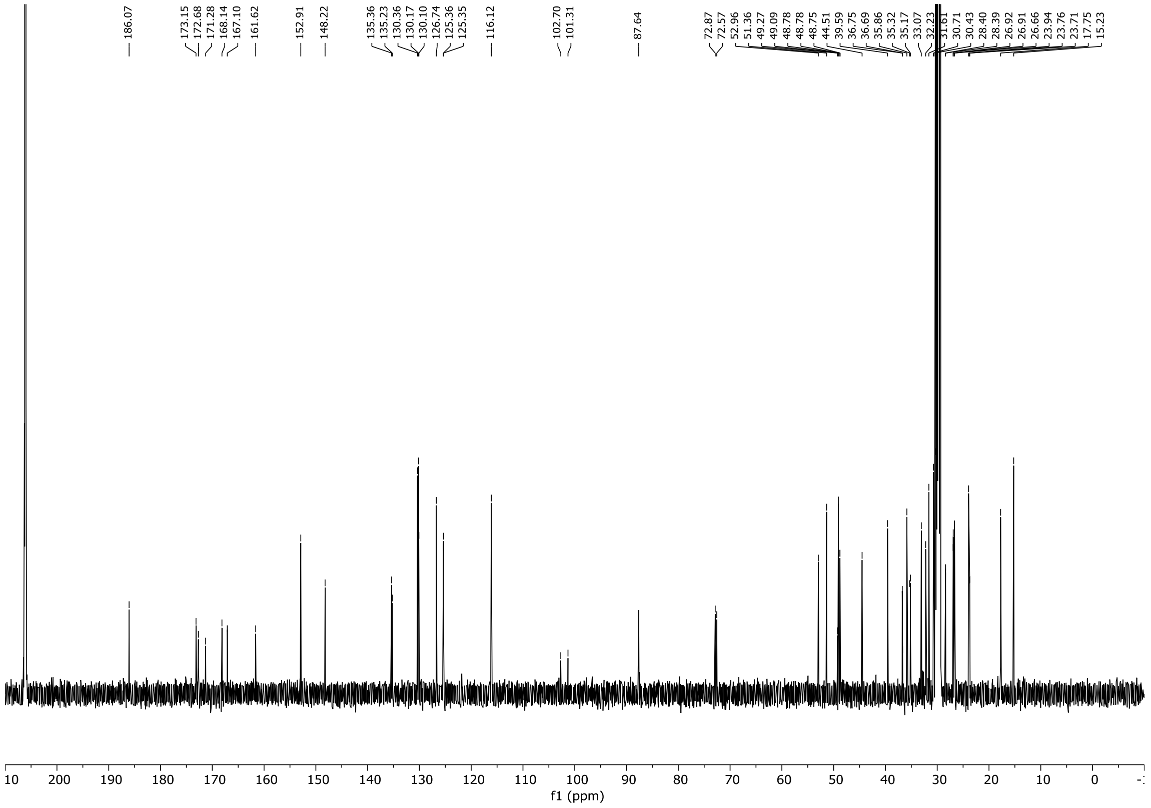

^{19}F NMR (471 MHz, $(CD_3)_2CO$) of compound **KH-5-224**:

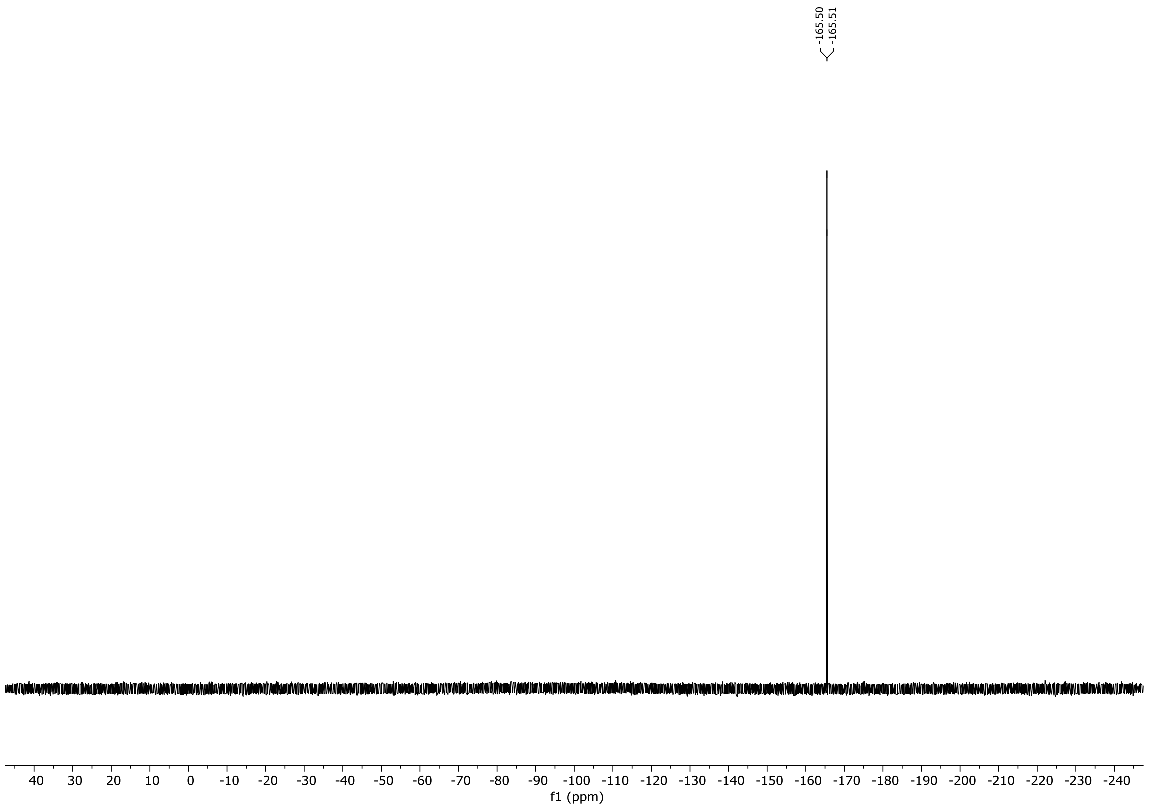

^{1}H NMR (500 MHz, $(CD_3)_2CO$) of compound **KH-5-225**:

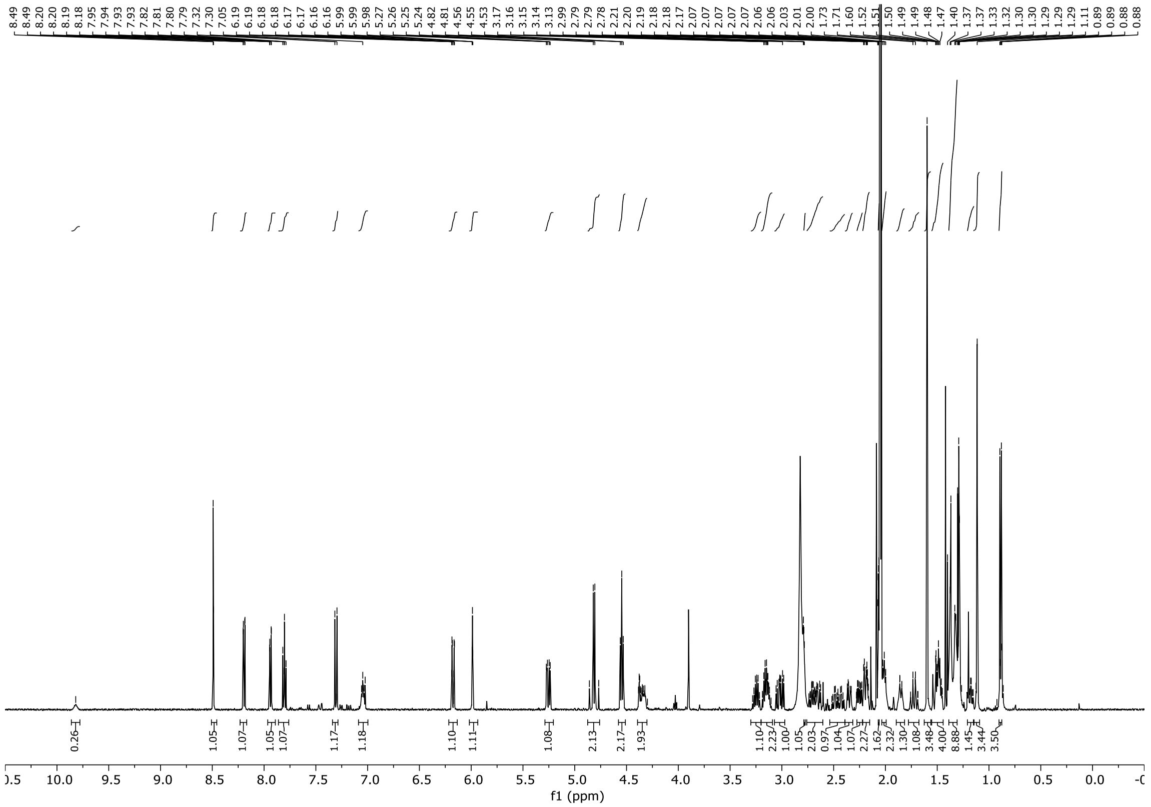

^{13}C NMR (126 MHz, $(CD_3)_2CO$) of compound **KH-5-225**:

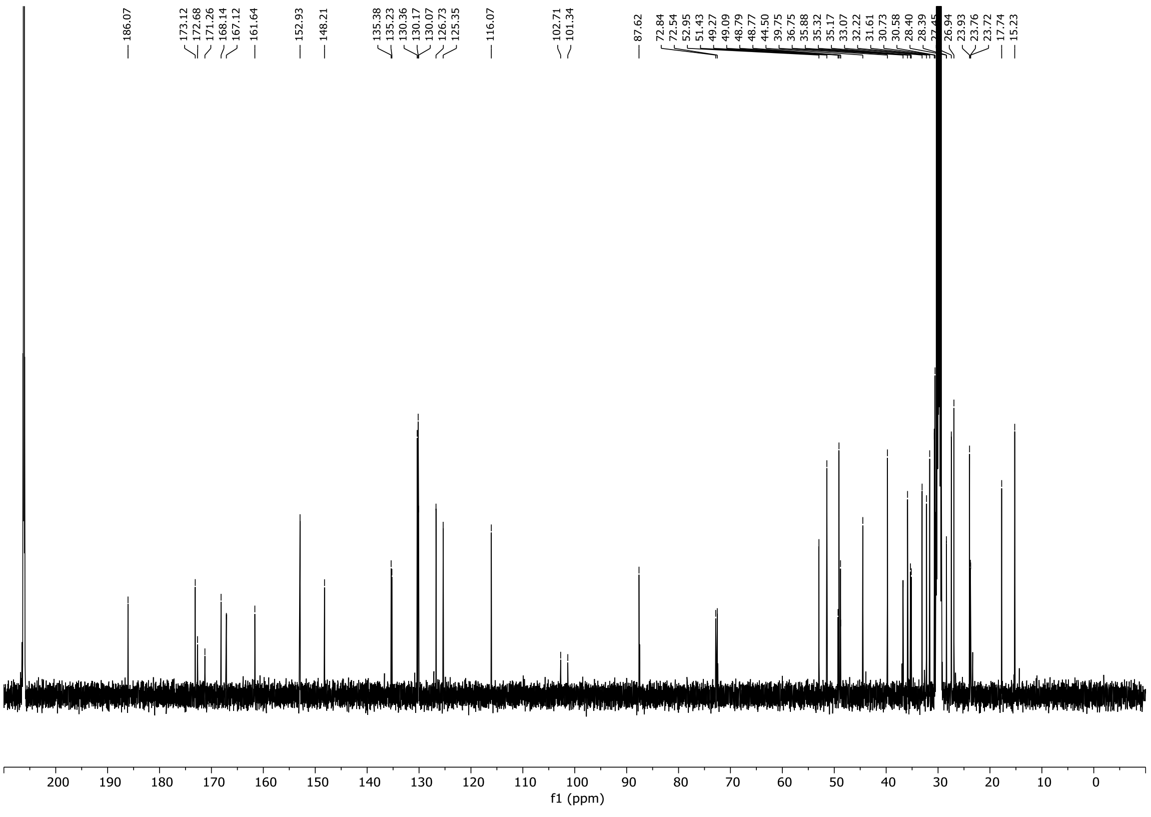

^{19}F NMR (471 MHz, $(CD_3)_2CO$) of compound **KH-5-225**:

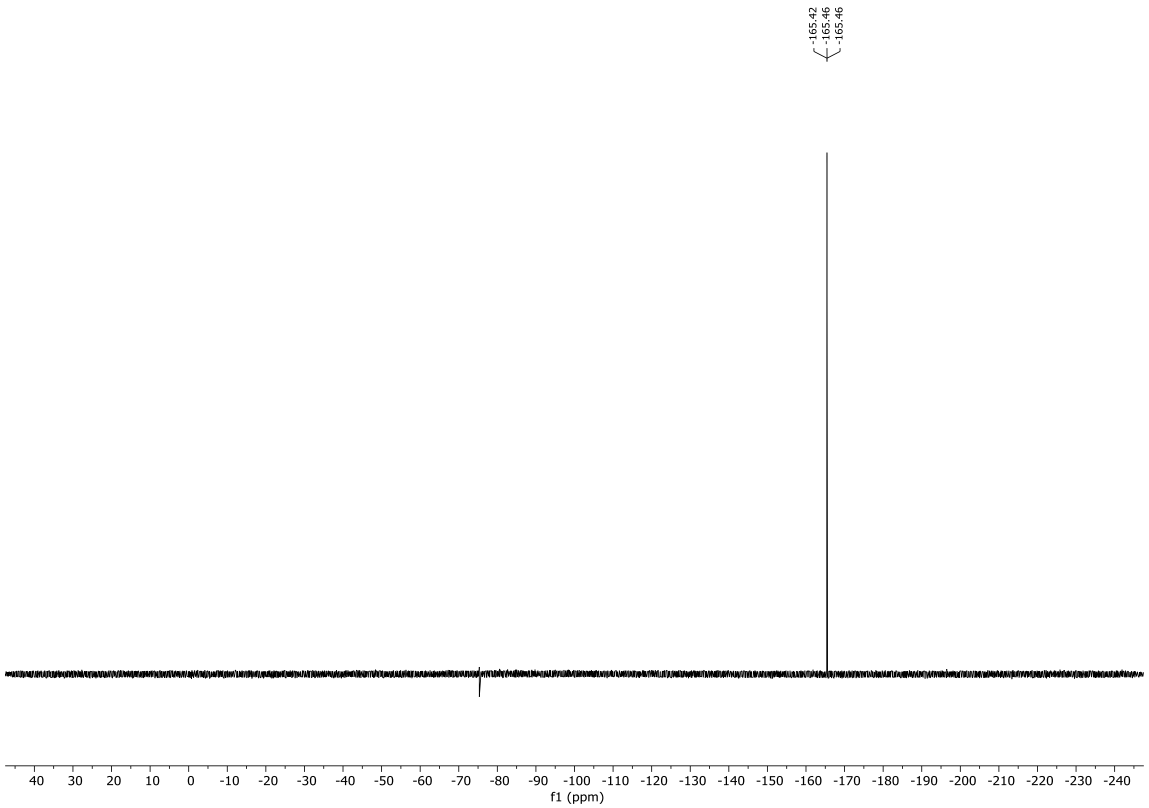

^{1}H NMR (500 MHz, $(CD_3)_2CO$) of compound **KH-5-226**:

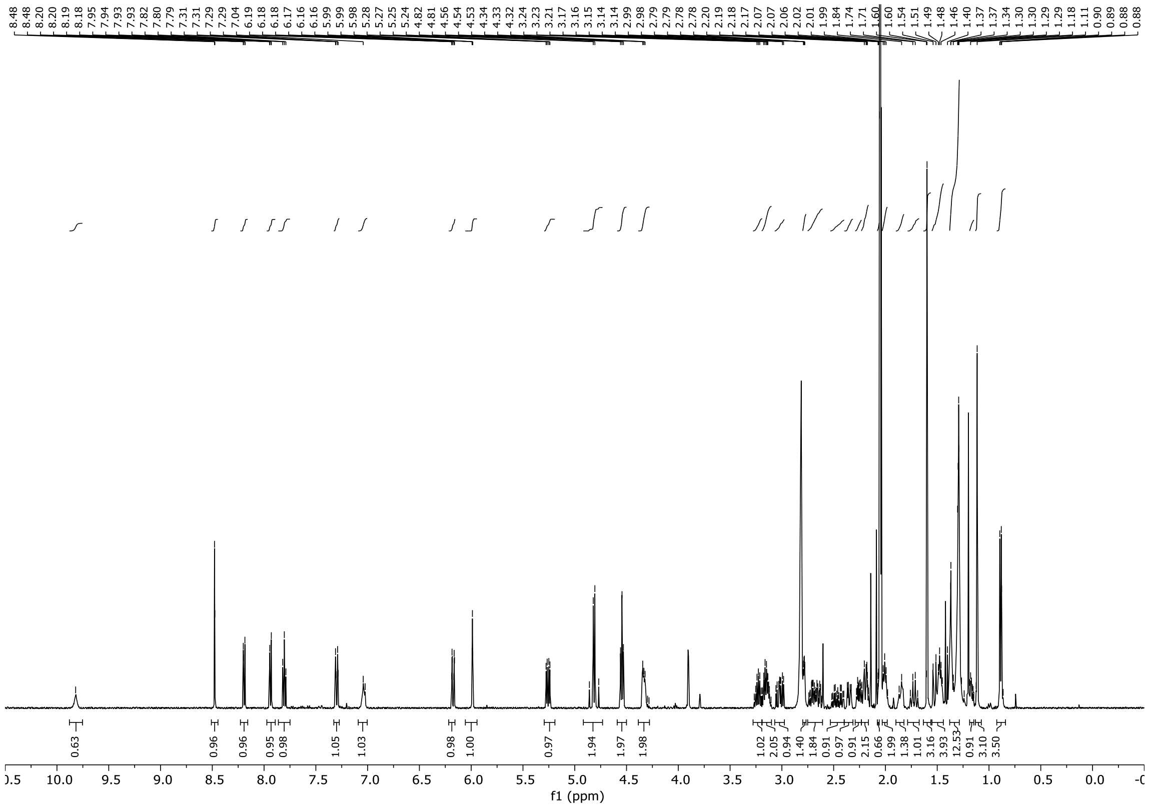

^{13}C NMR (126 MHz, $(CD_3)_2CO$) of compound **KH-5-226**:

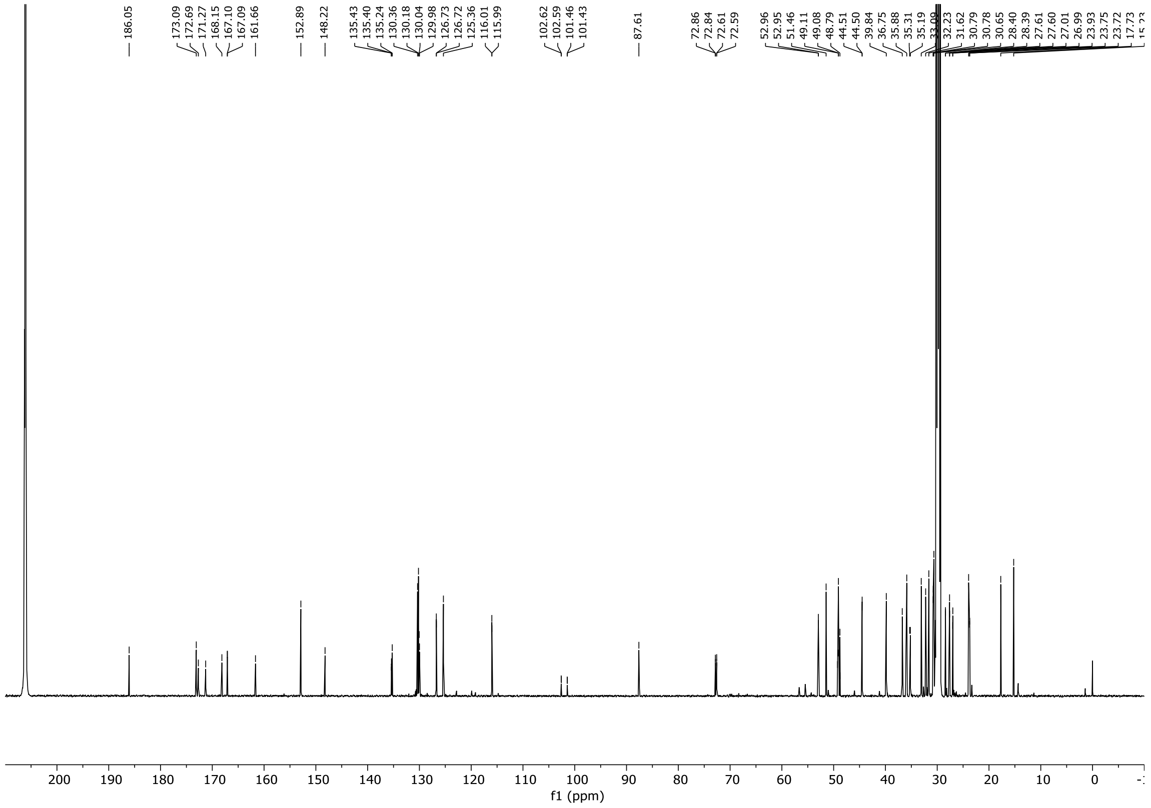

^{19}F NMR (471 MHz, $(CD_3)_2CO$) of compound **KH-5-226**:

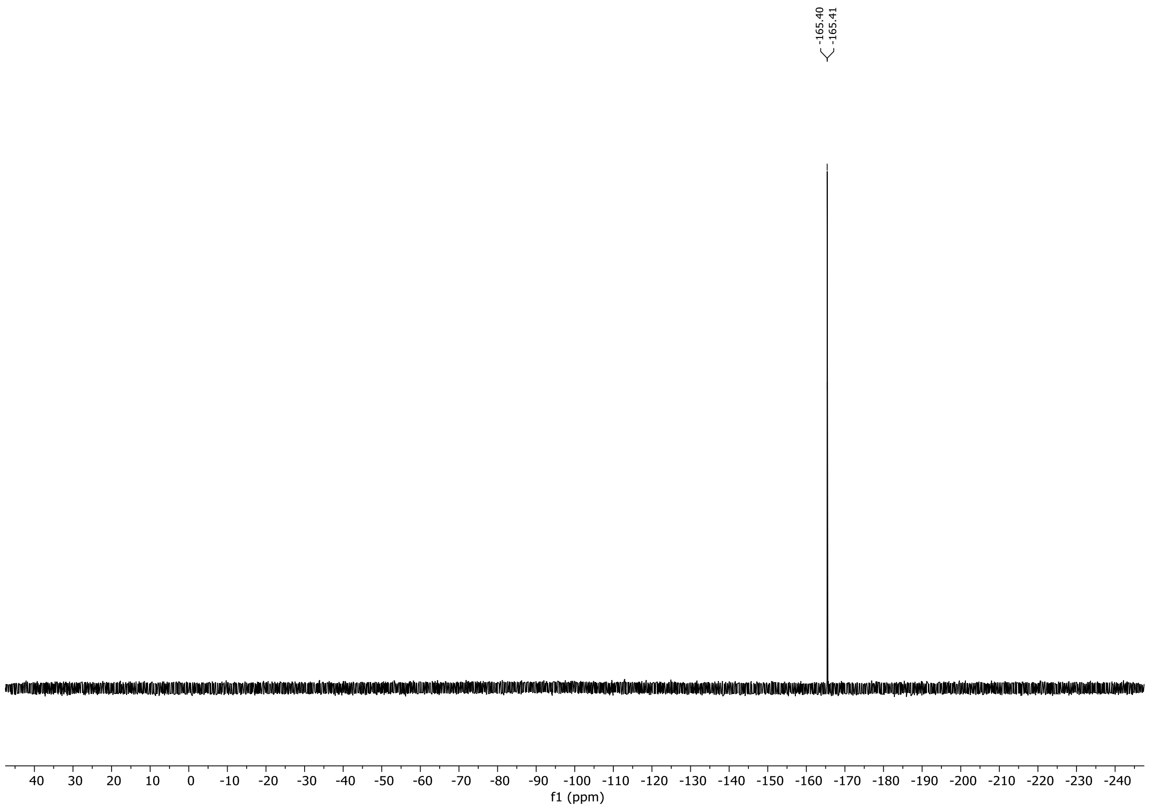

Me$_2$-Arylazopyrazole photoPROTACs

^{1}H NMR (600 MHz, $(CD_3)_2SO$) of compound **42**:

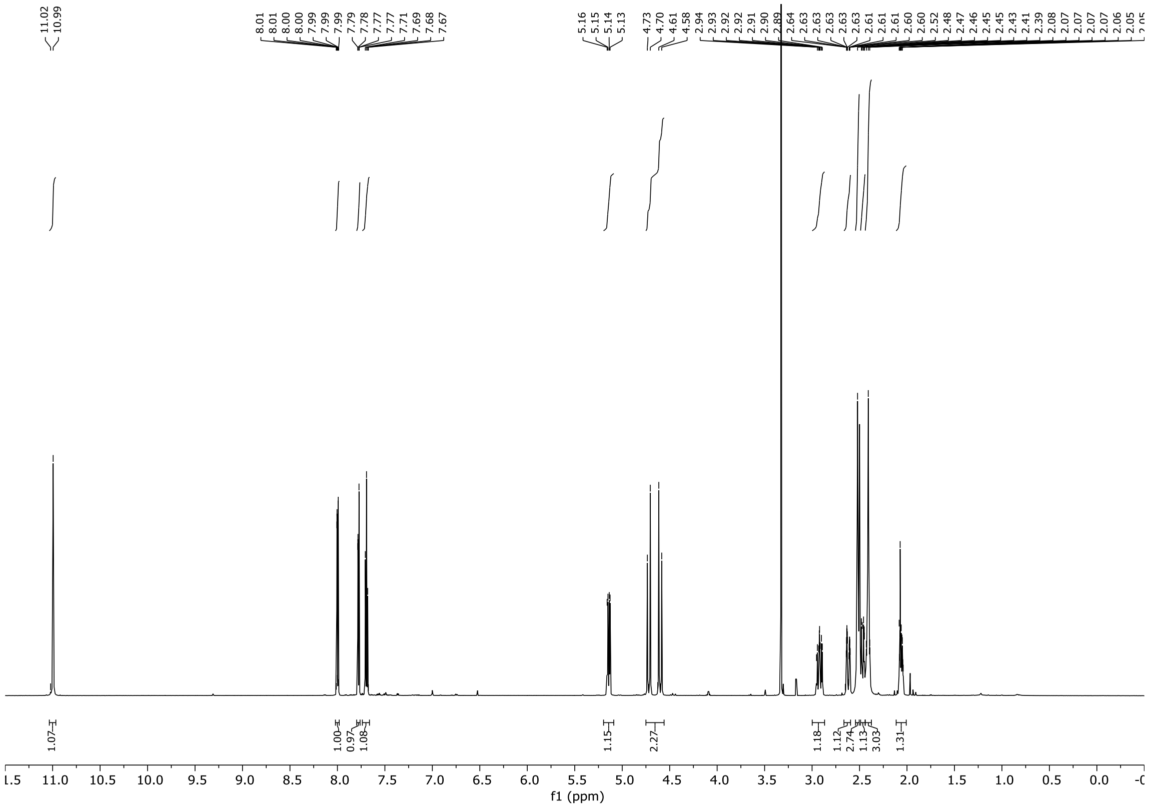

^{13}C NMR (151 MHz, $(CD_3)_2SO$) of compound **42**:

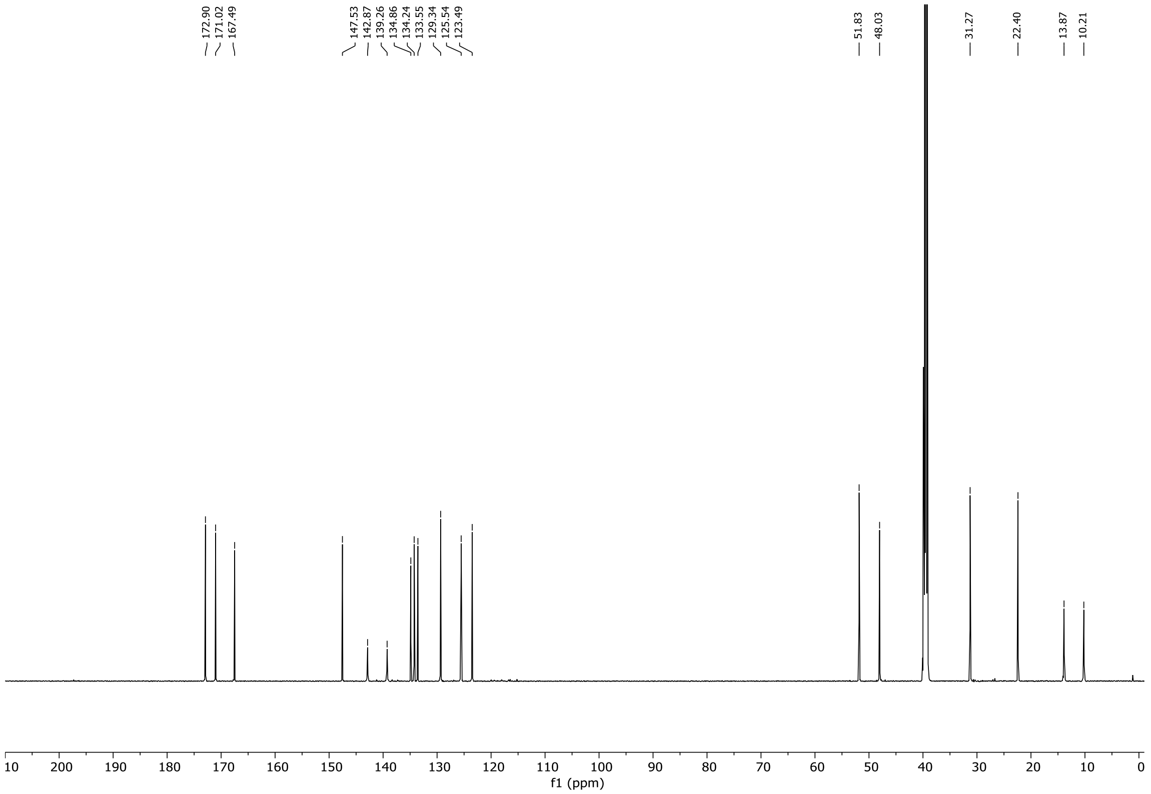

^{1}H NMR (400 MHz, $CDCl_3$) of compound **41**:

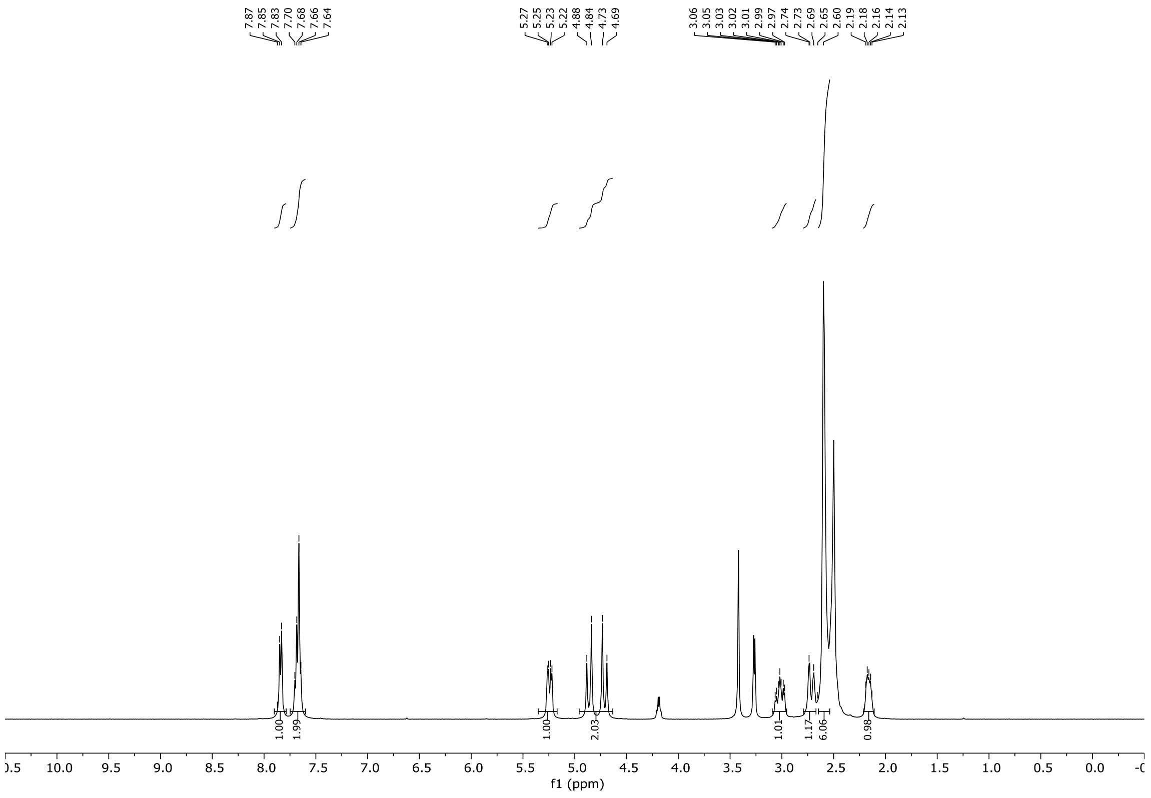

^{13}C NMR (101 MHz, $CDCl_3$) of compound **41**:

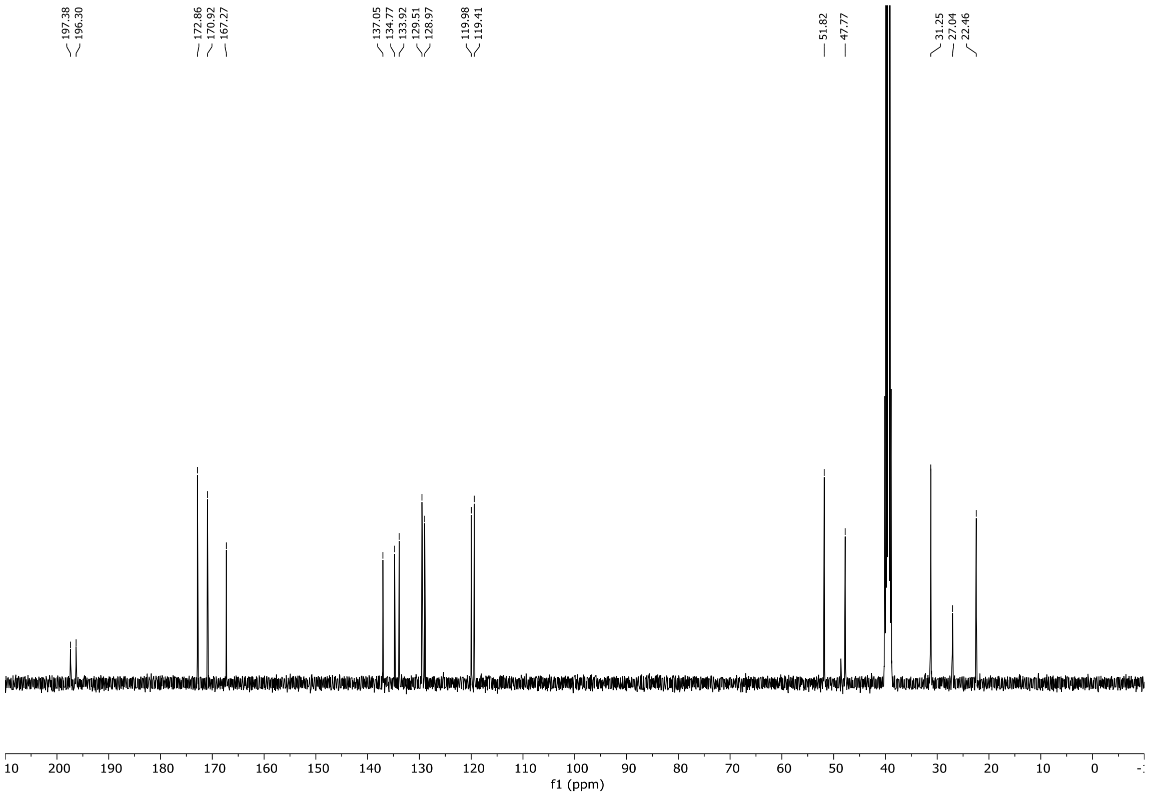

^1H NMR (500 MHz, $(CD_3)_2CO$) of compound **Me$_2$-arylazopyrazole photoswitch**:

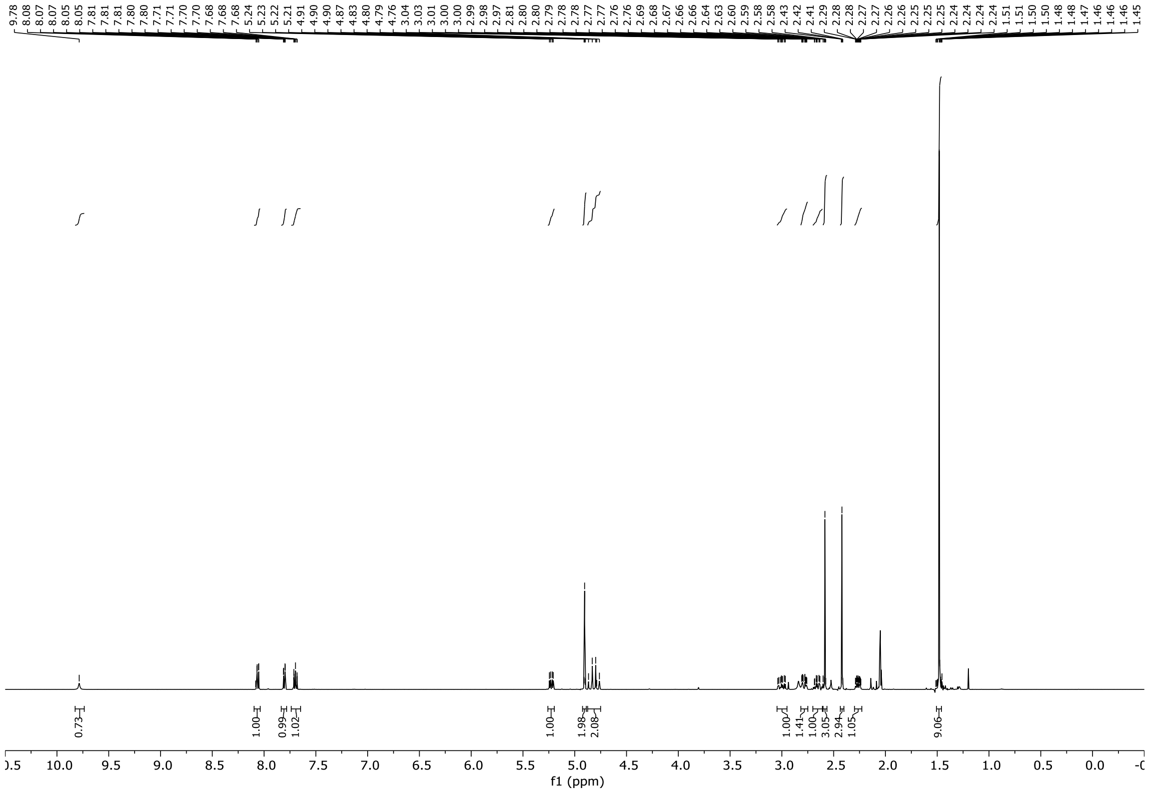

^{13}C NMR (126 MHz, $(CD_3)_2CO$) of compound **Me$_2$-arylazopyrazole photoswitch**:

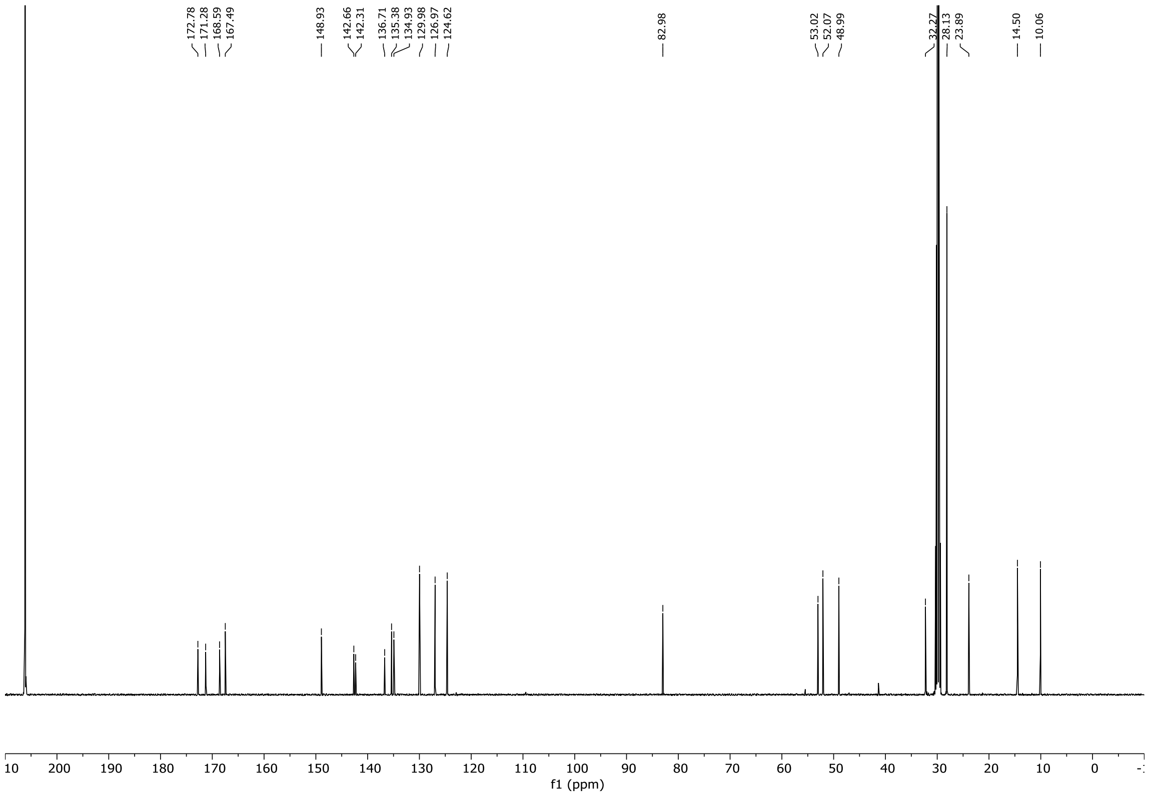

HSQC (500/126 MHz, $(CD_3)_2CO$) of compound **Me₂-arylazopyrazole photoswitch**:

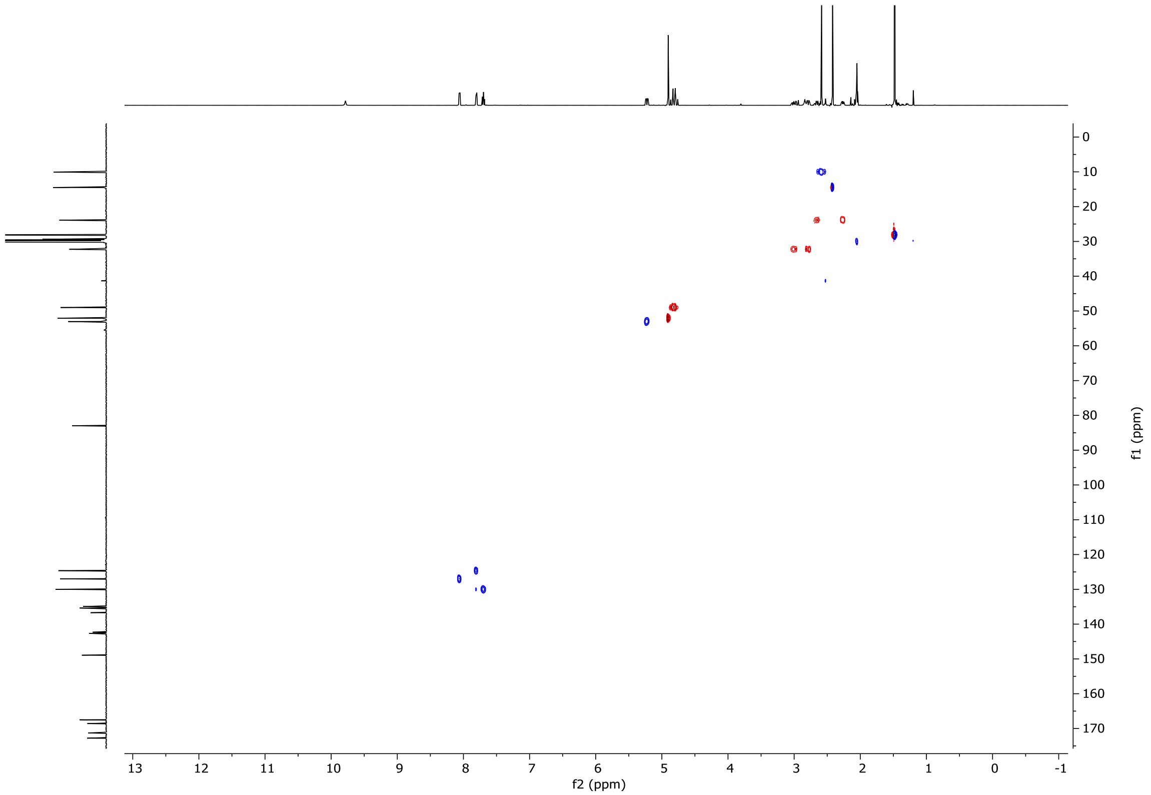

HMBC (500/126 MHz, $(CD_3)_2CO$) of compound **Me₂-arylazopyrazole photoswitch**:

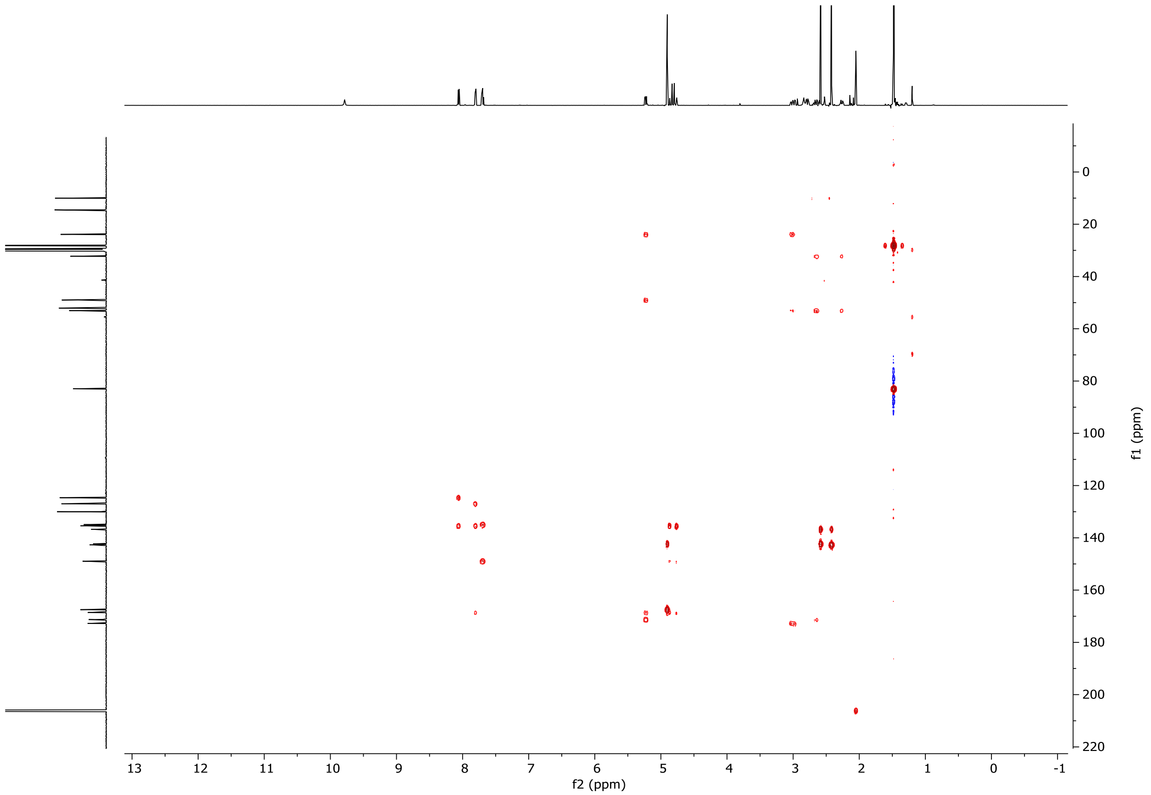

COSY (500 MHz, $(CD_3)_2CO$) of compound **Me₂-arylazopyrazole photoswitch**:

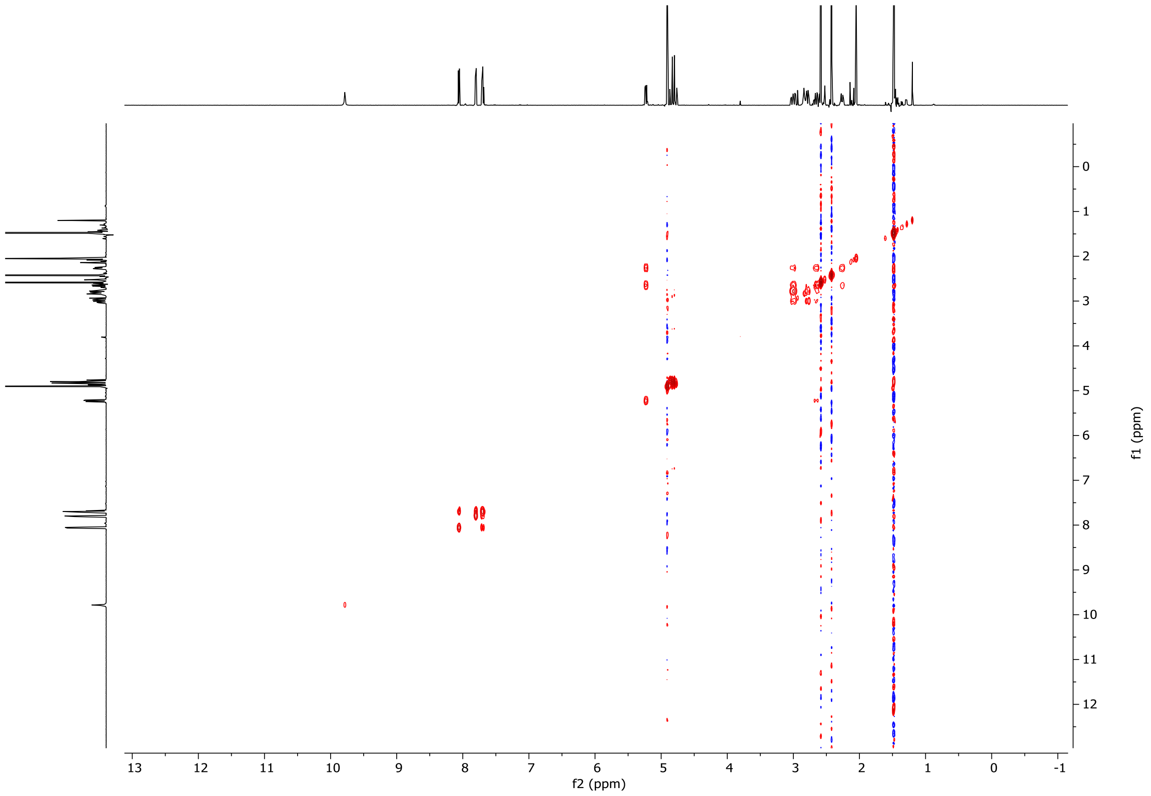

^{1}H NMR (500 MHz, $(CD_3)_2SO$) of compound **59**:

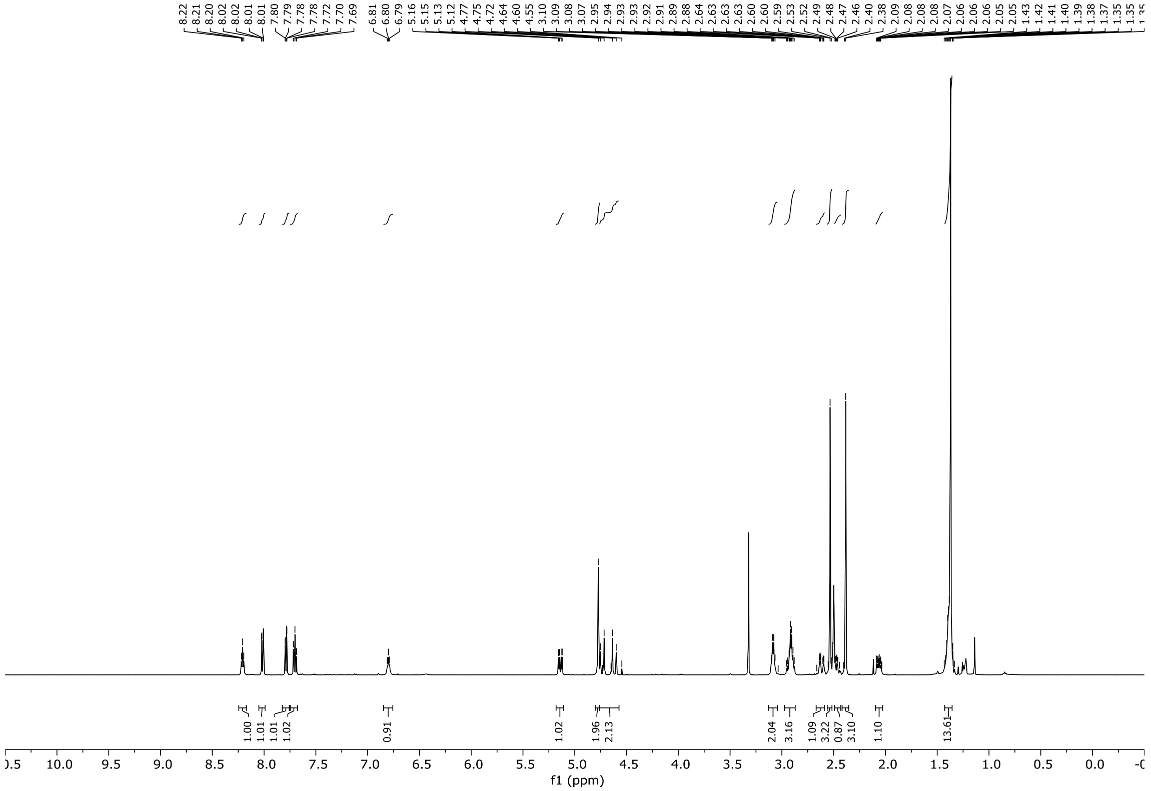

^{13}C NMR (126 MHz, $(CD_3)_2SO$) of compound **59**:

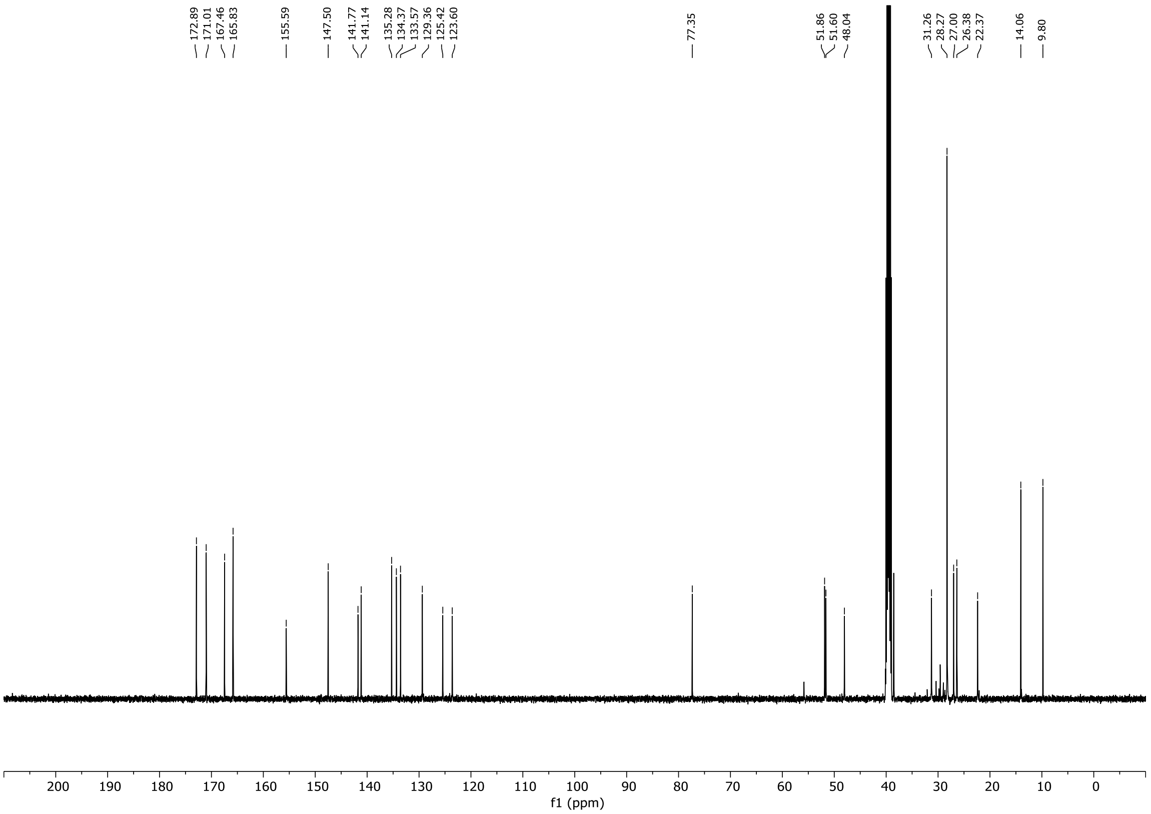

HSQC (500/126 MHz, $(CD_3)_2SO$) of compound **59**:

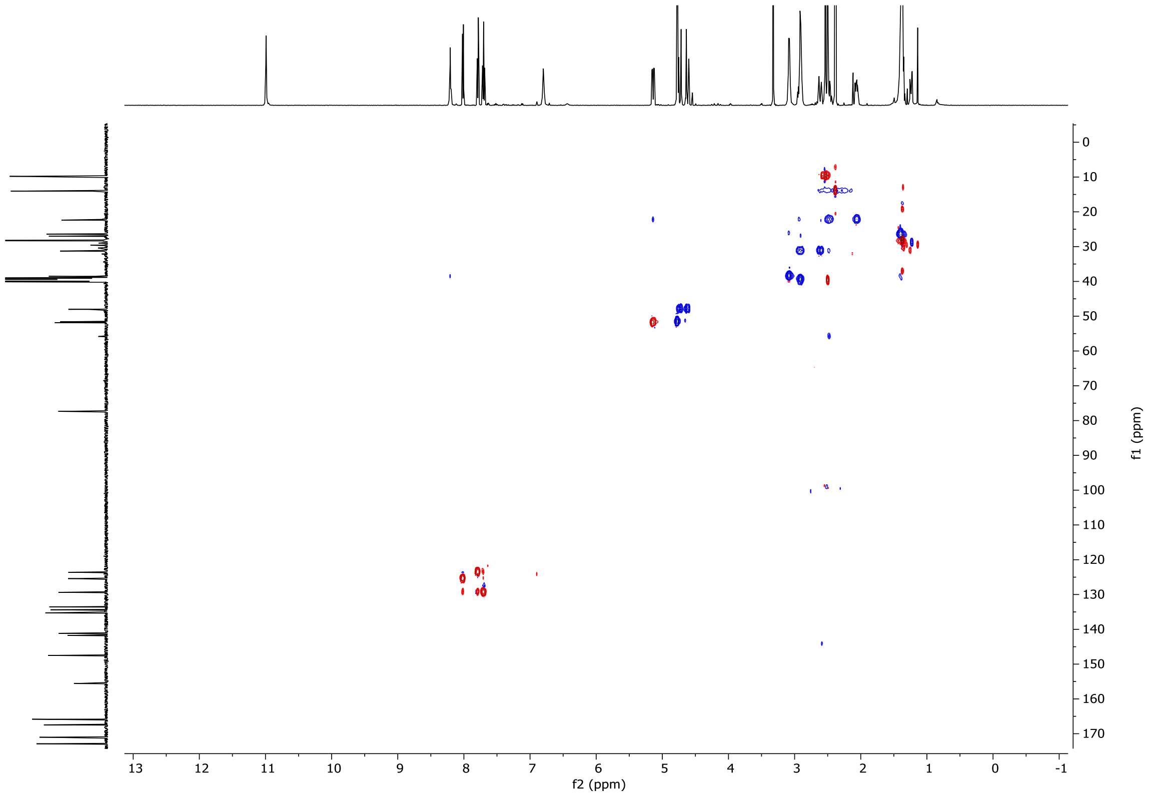

HMBC (500/126 MHz, $(CD_3)_2SO$) of compound **59**:

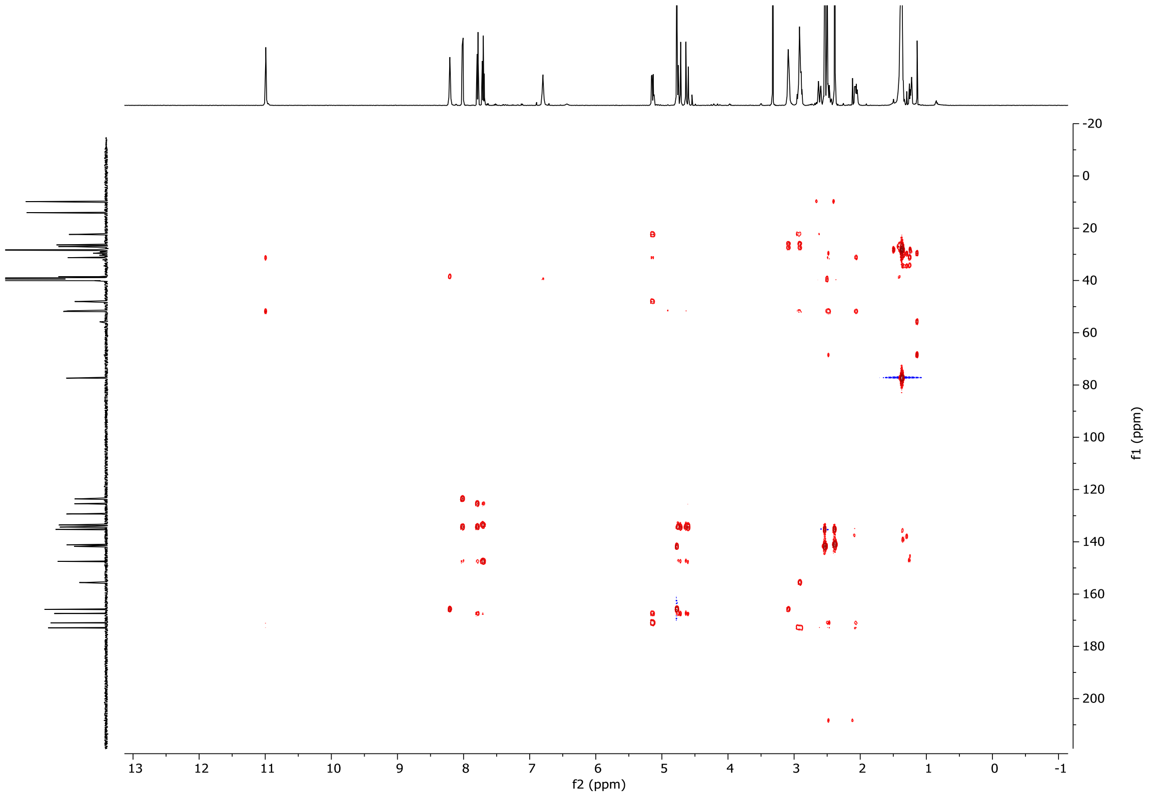

COSY (500 MHz, $(CD_3)_2SO$) of compound **59**:

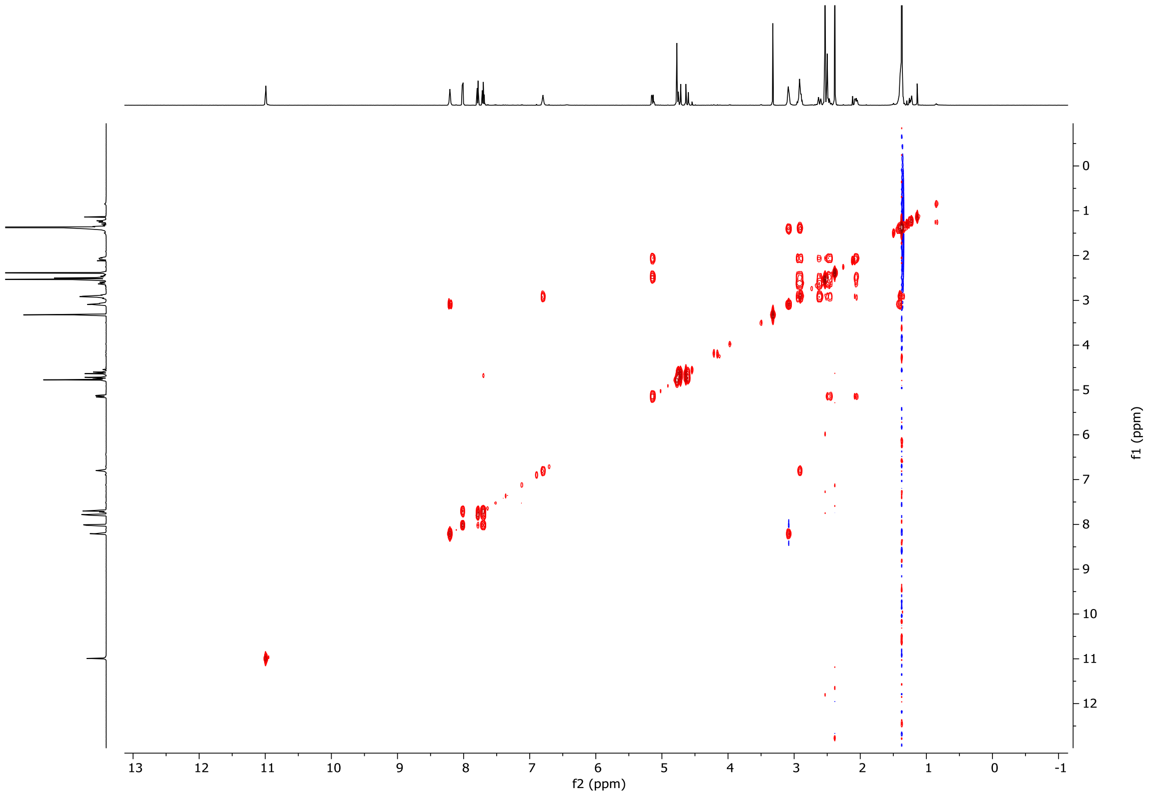

^{1}H NMR (500 MHz, $(CD_3)_2SO$) of compound **60**:

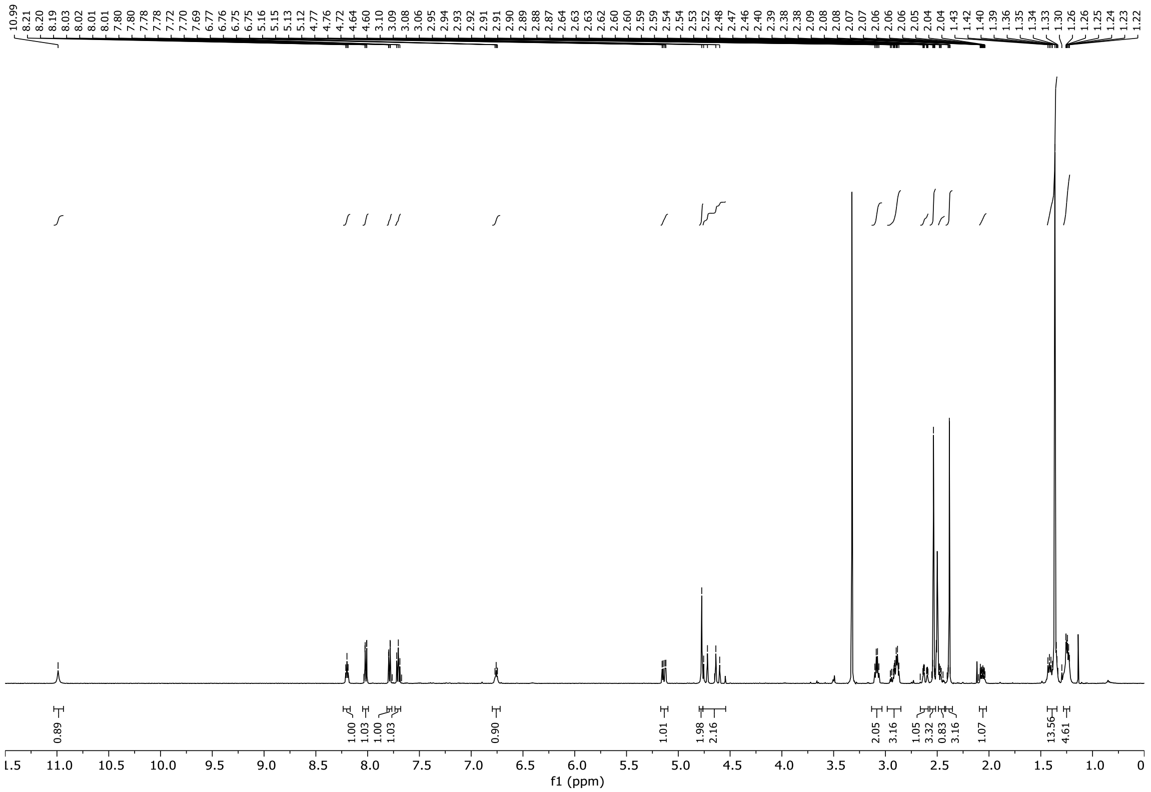

^{13}C NMR (126 MHz, $(CD_3)_2SO$) of compound **60**:

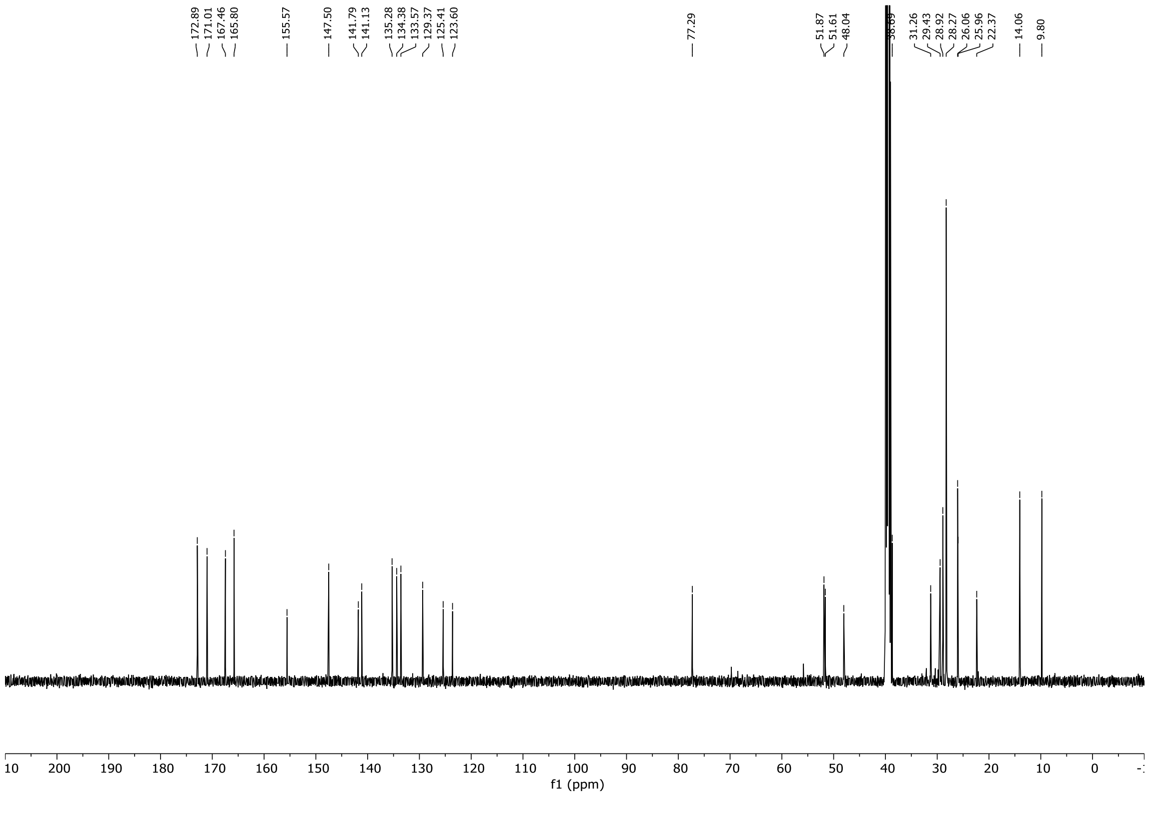

HSQC (500/126 MHz, $(CD_3)_2SO$) of compound **60**:

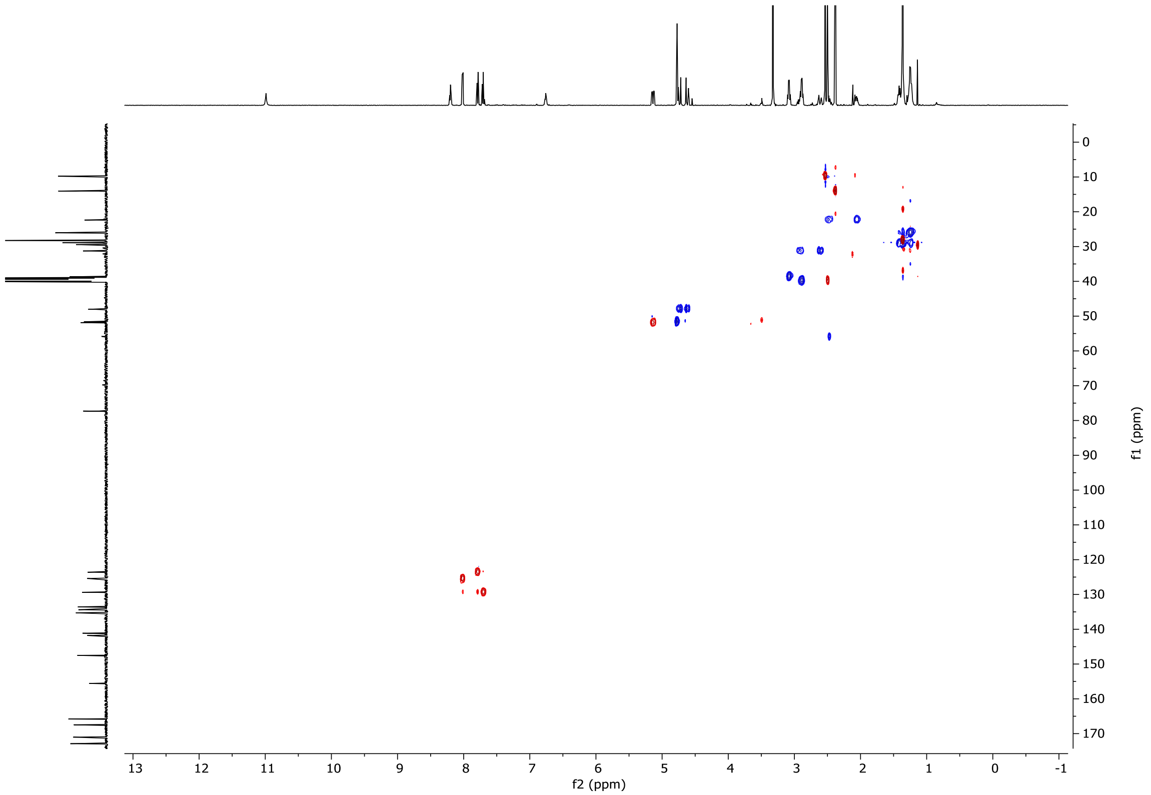

HMBC (500/126 MHz, $(CD_3)_2SO$) of compound **60**:

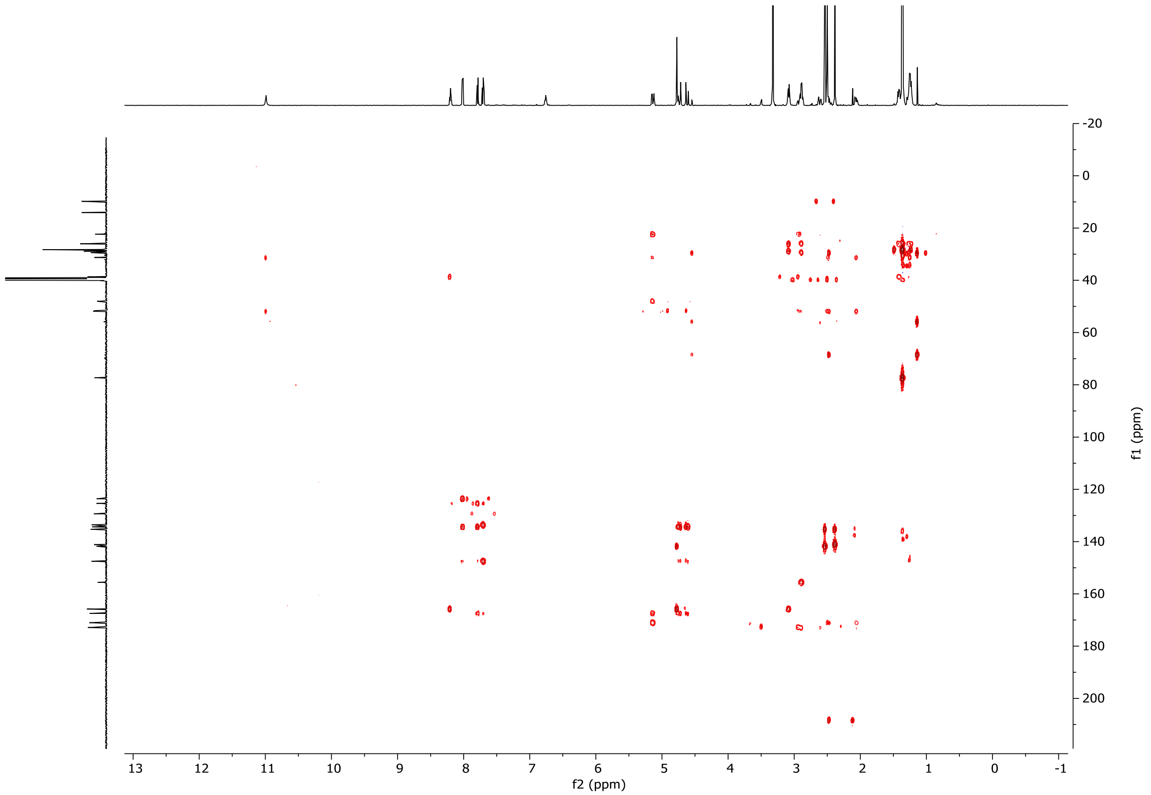

COSY (500 MHz, $(CD_3)_2SO$) of compound **60**:

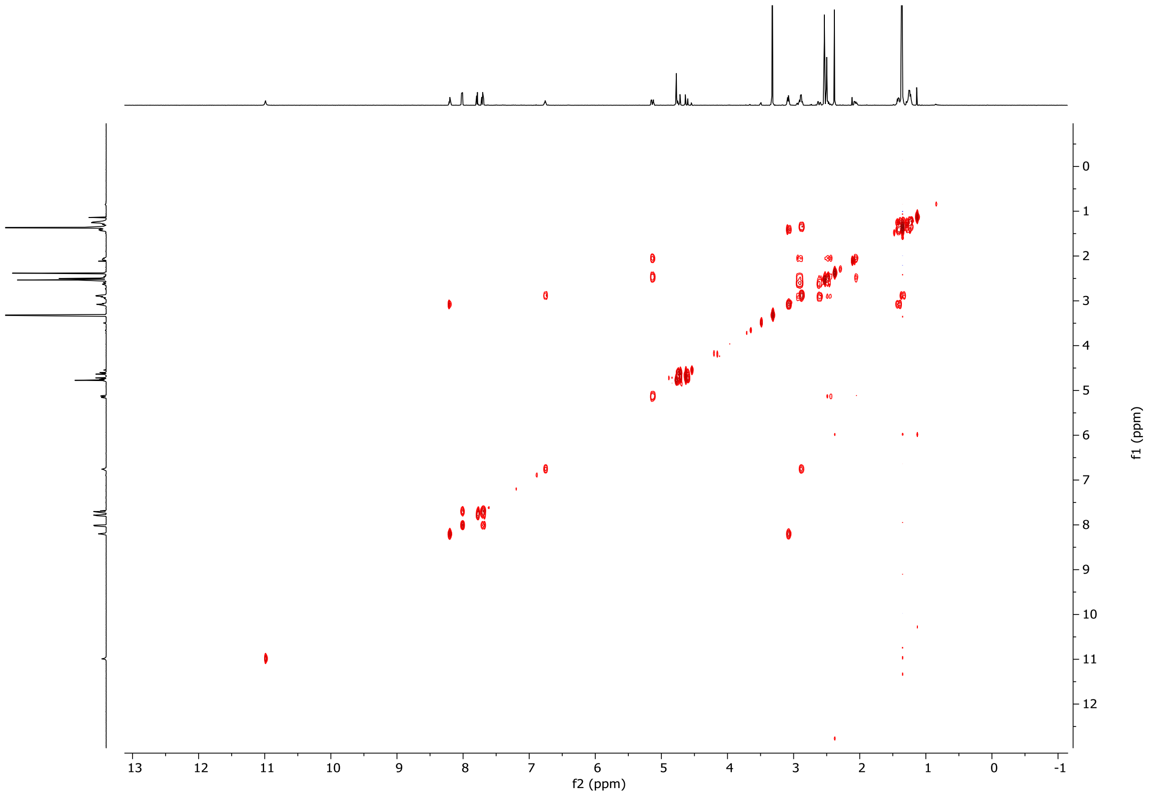

^{1}H NMR (500 MHz, $(CD_3)_2SO$) of compound **61**:

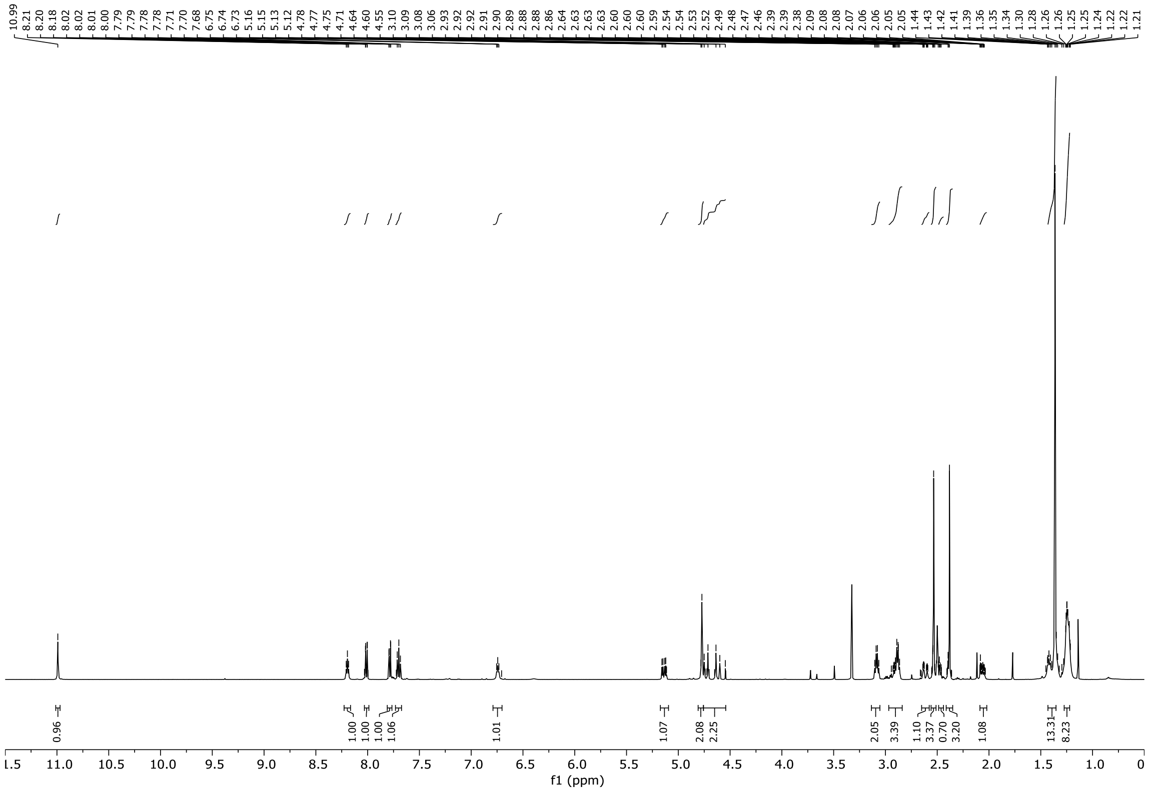

^{13}C NMR (126 MHz, $(CD_3)_2SO$) of compound **61**:

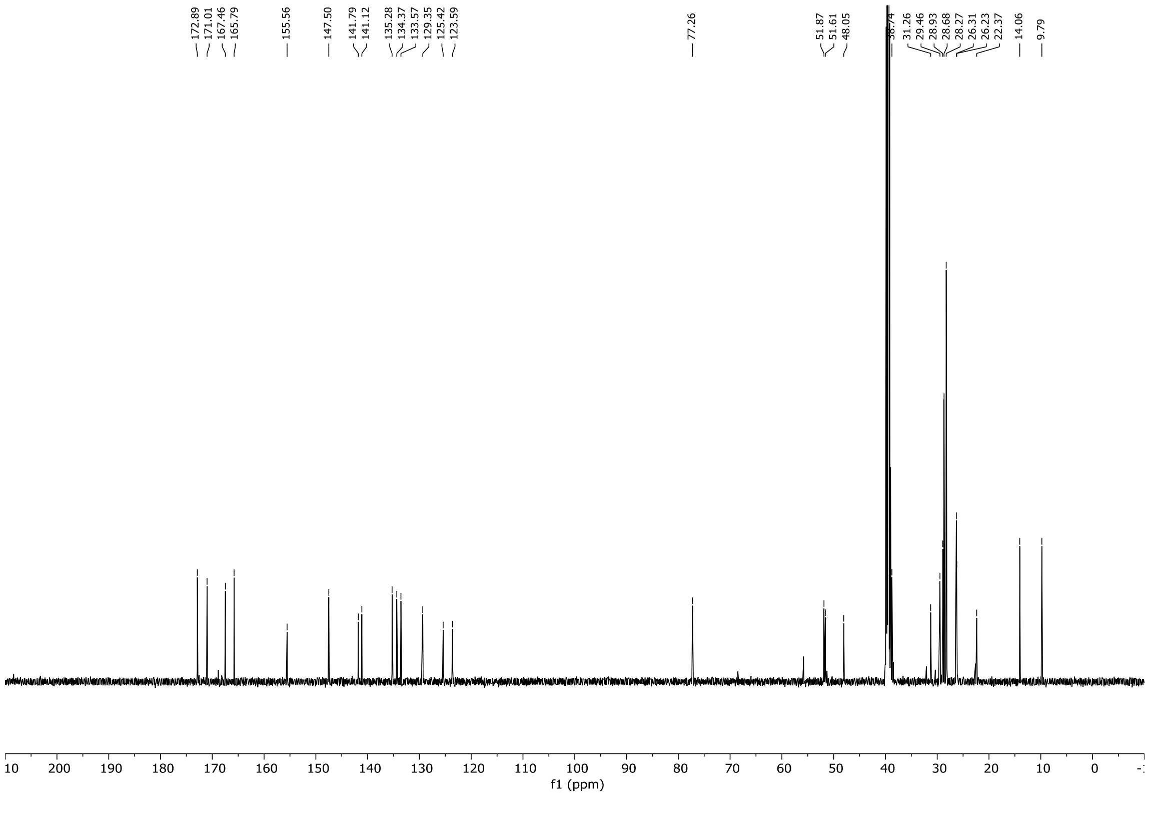

HSQC (500/126 MHz, $(CD_3)_2SO$) of compound **61**:

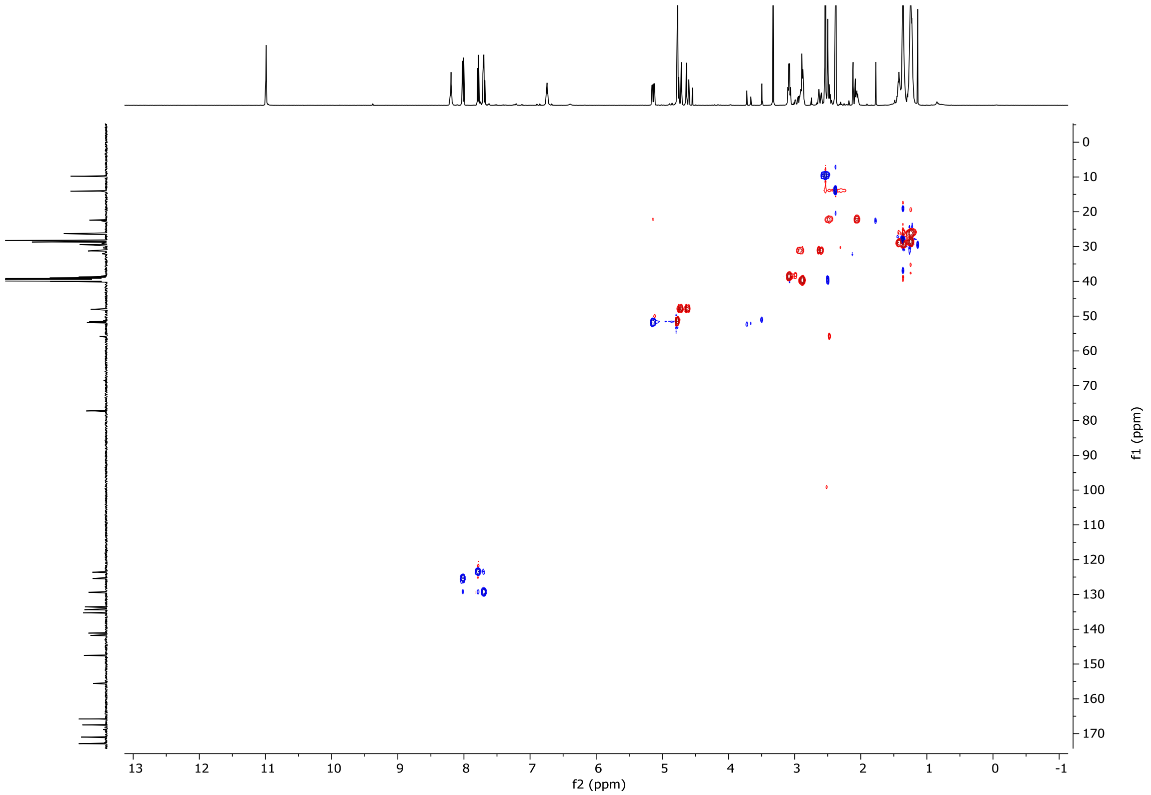

HMBC (500/126 MHz, $(CD_3)_2SO$) of compound **61**:

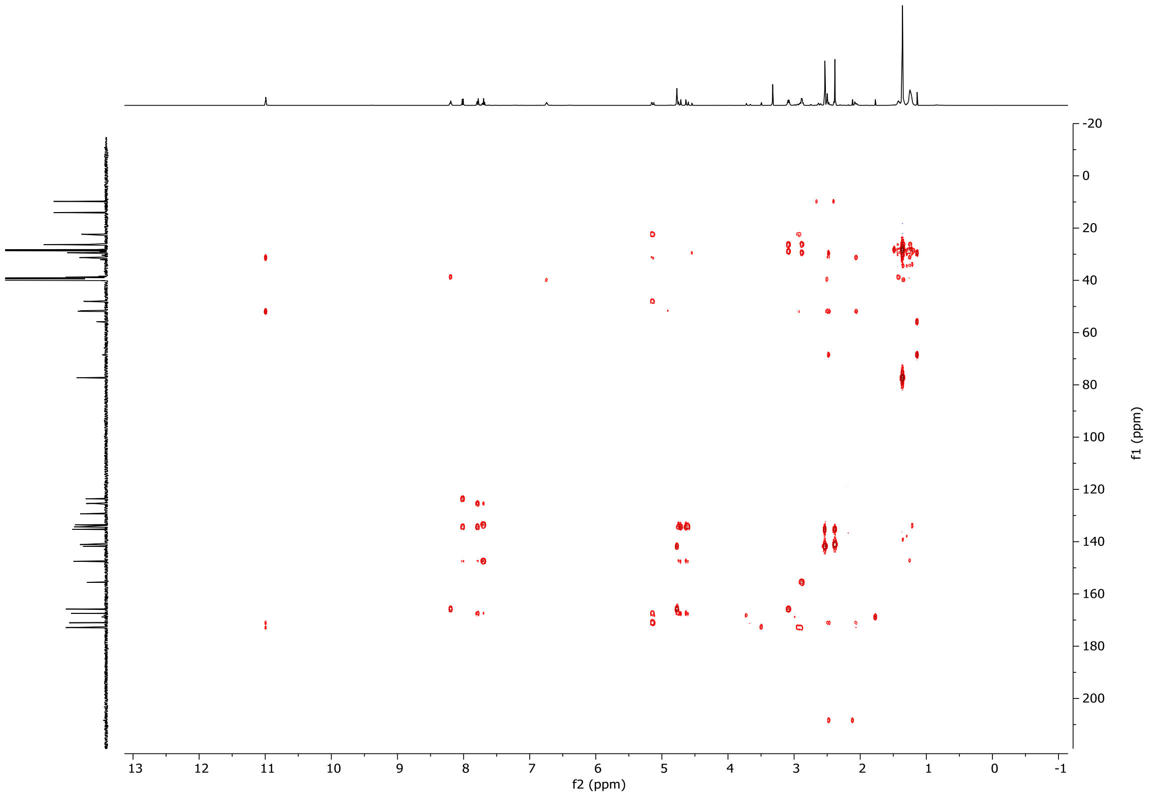

COSY (500 MHz, $(CD_3)_2SO$) of compound **61**:

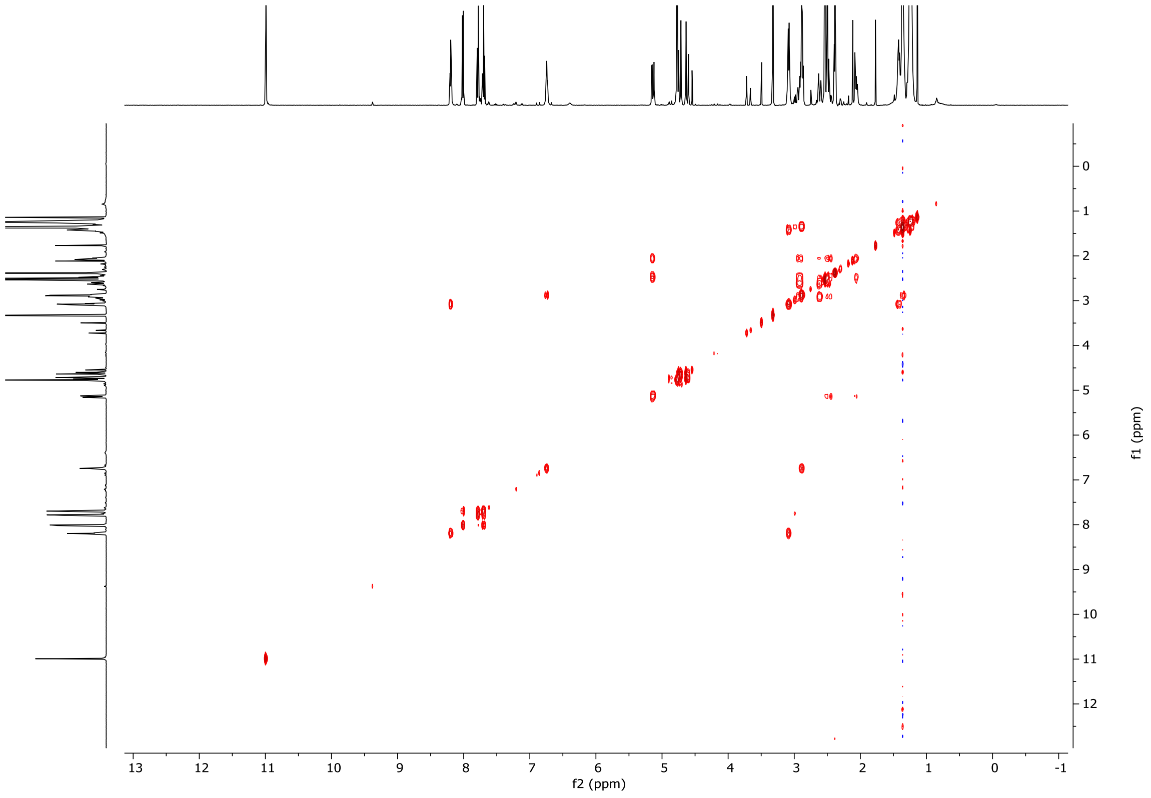

^{1}H NMR (500 MHz, $(CD_3)_2SO$) of compound **62**:

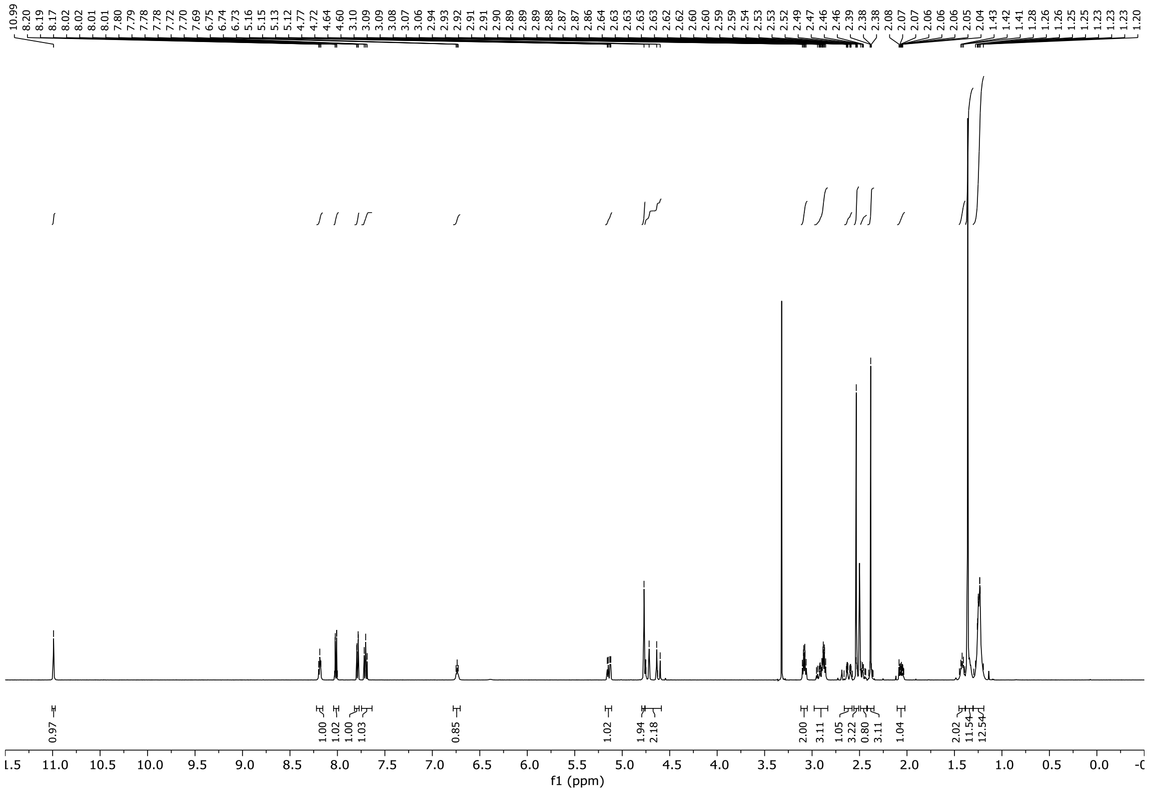

^{13}C NMR (126 MHz, $(CD_3)_2SO$) of compound **62**:

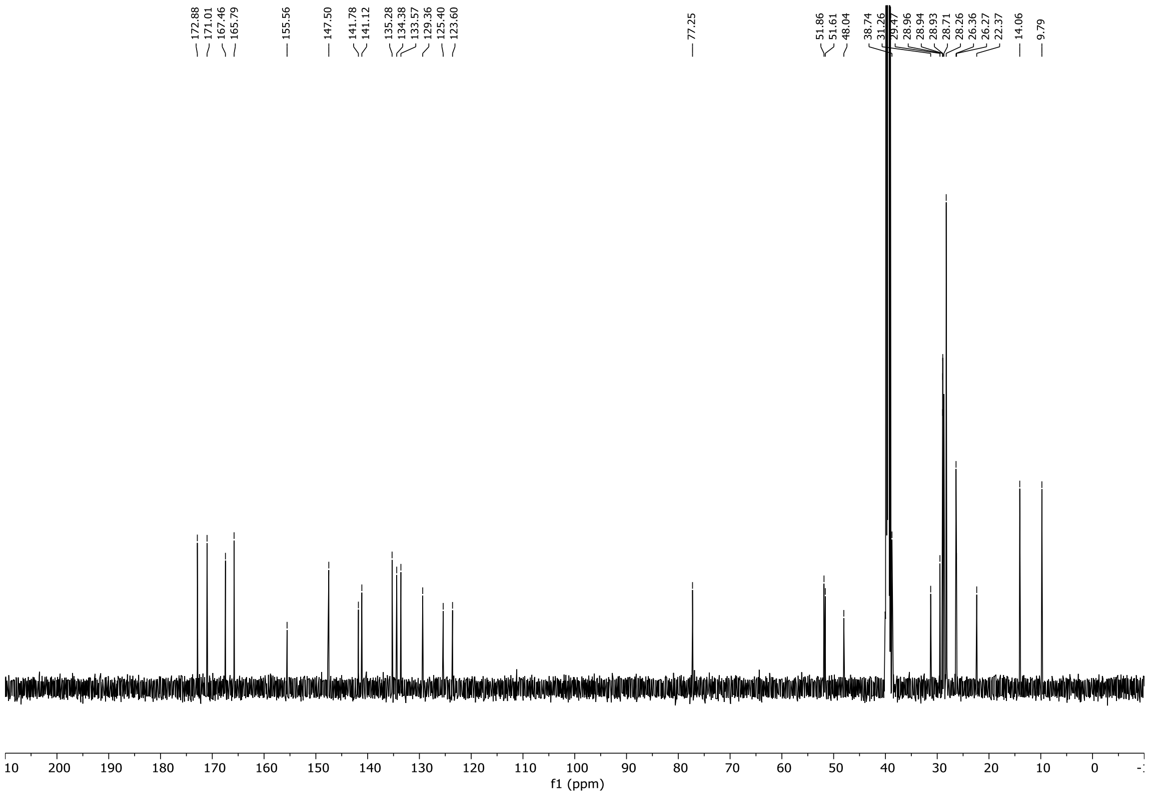

HSQC (500/126 MHz, $(CD_3)_2SO$) of compound **62**:

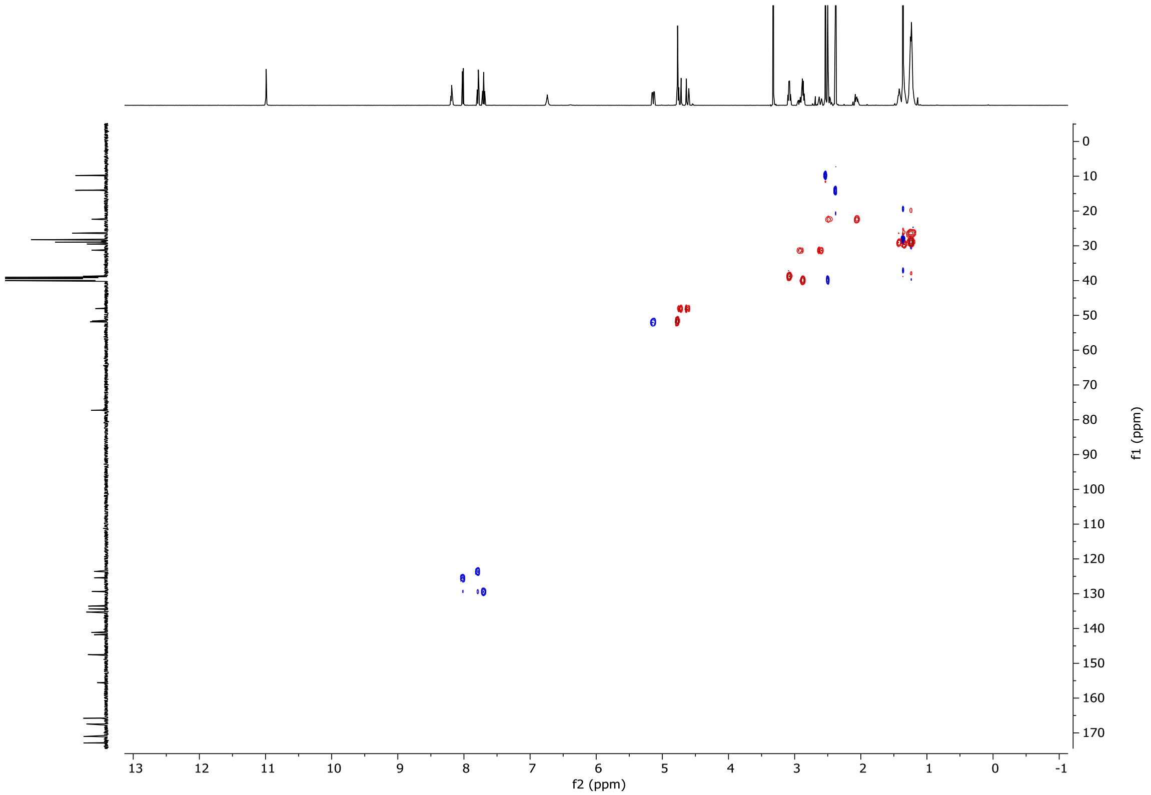

HMBC (500/126 MHz, $(CD_3)_2SO$) of compound **62**:

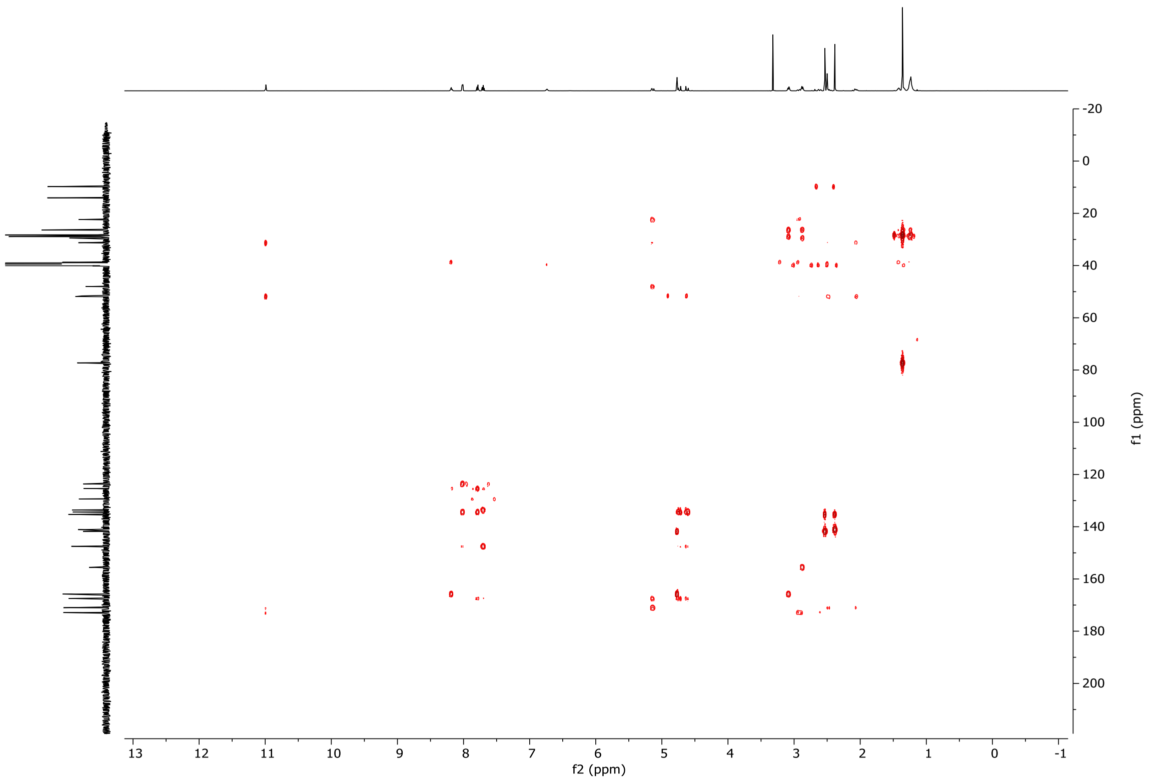

COSY (500 MHz, $(CD_3)_2SO$) of compound **62**:

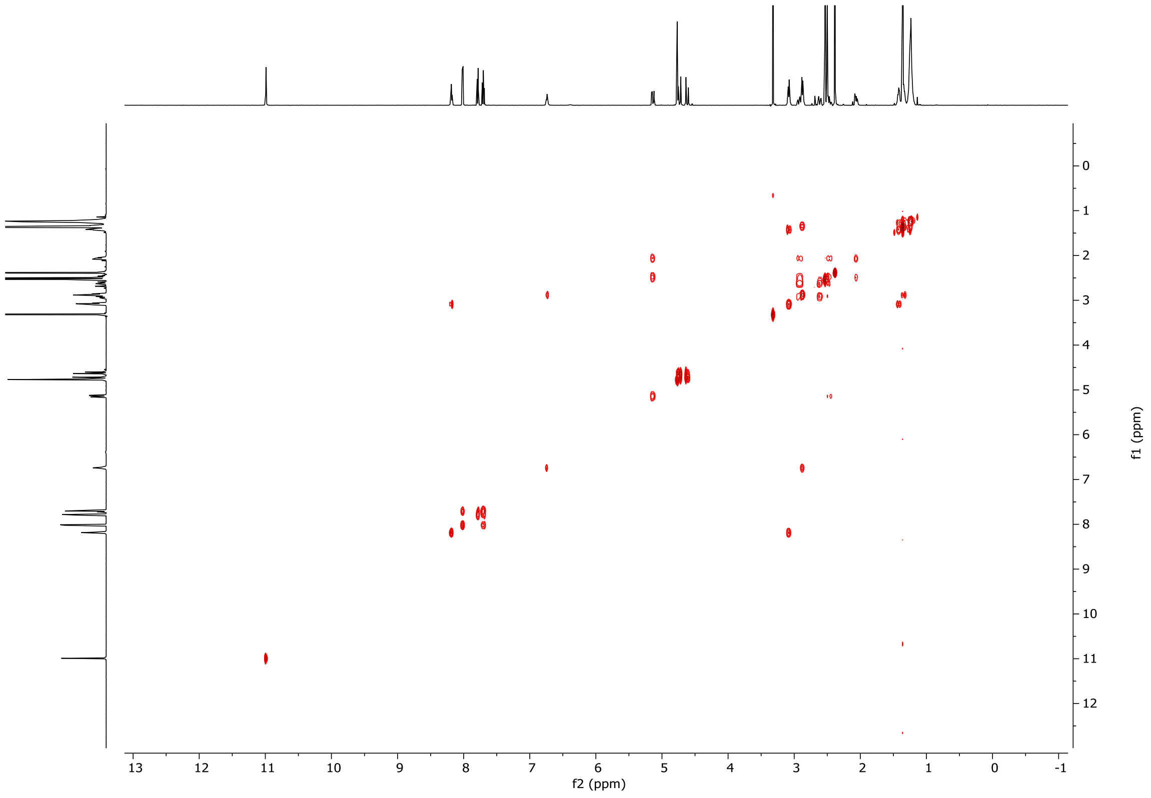

^{1}H NMR (500 MHz, $(CD_3)_2SO$) of compound **KH-5-298**:

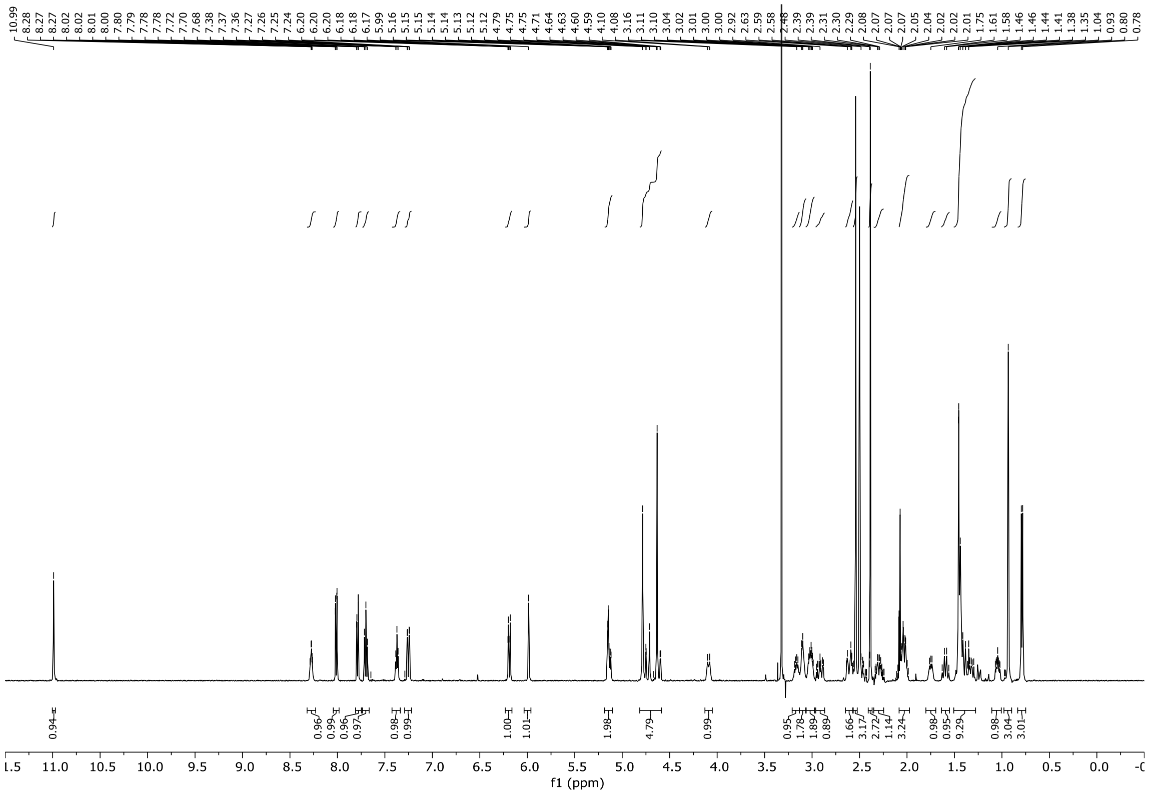

^{13}C NMR (126 MHz, $(CD_3)_2SO$) of compound **KH-5-298**:

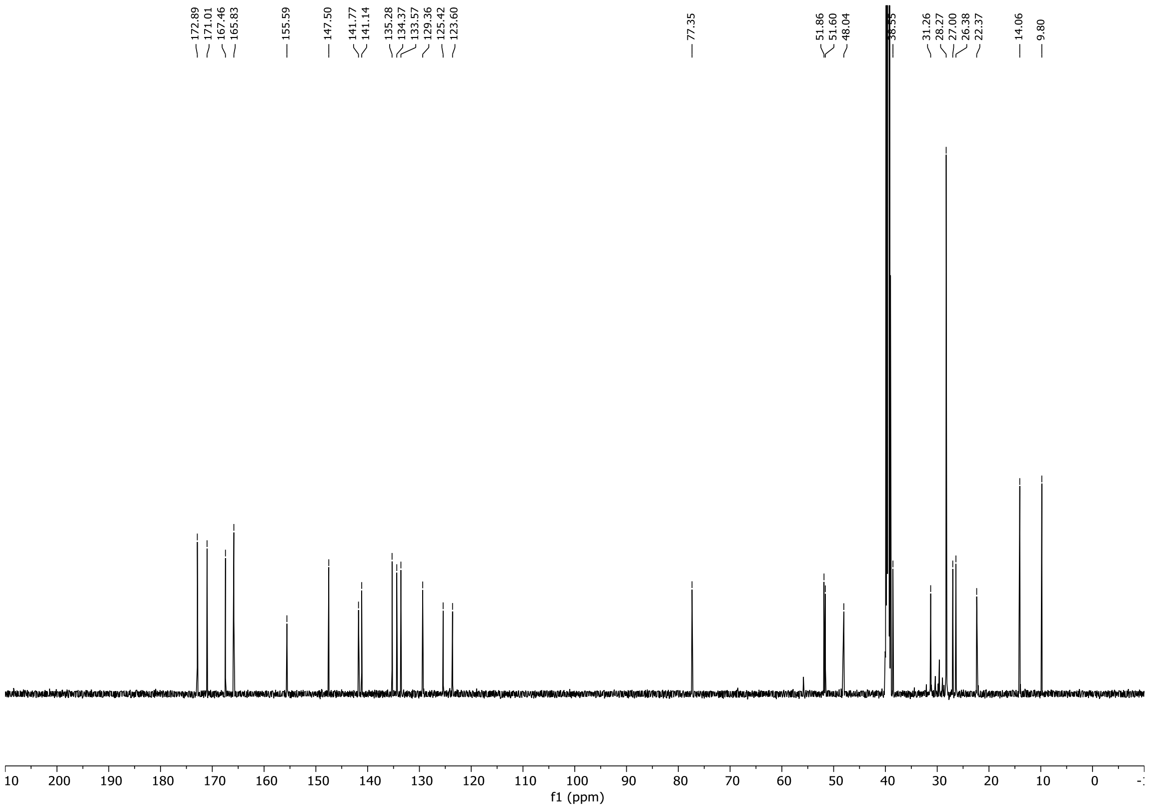

^{19}F NMR (471 MHz, $(CD_3)_2SO$) of compound **KH-5-298**:

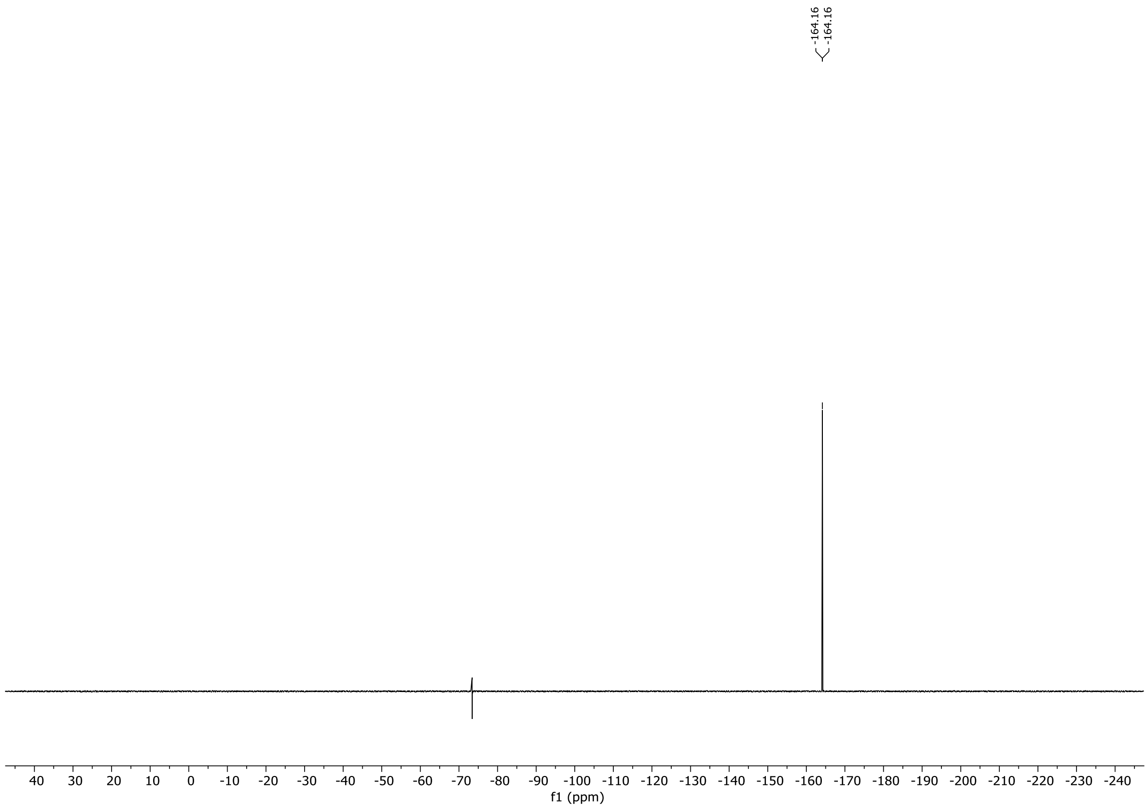

HSQC (500/126 MHz, $(CD_3)_2SO$) of compound **KH-5-298**:

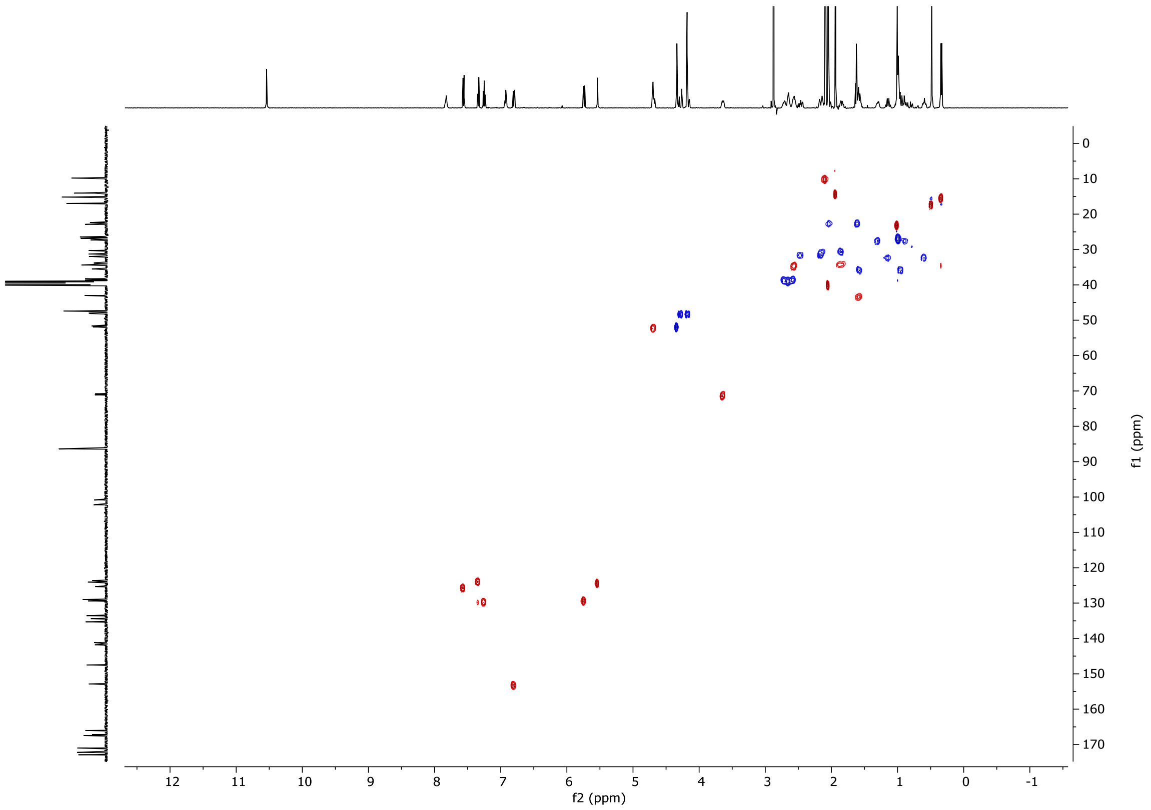

HMBC (500/126 MHz, $(CD_3)_2SO$) of compound **KH-5-298**:

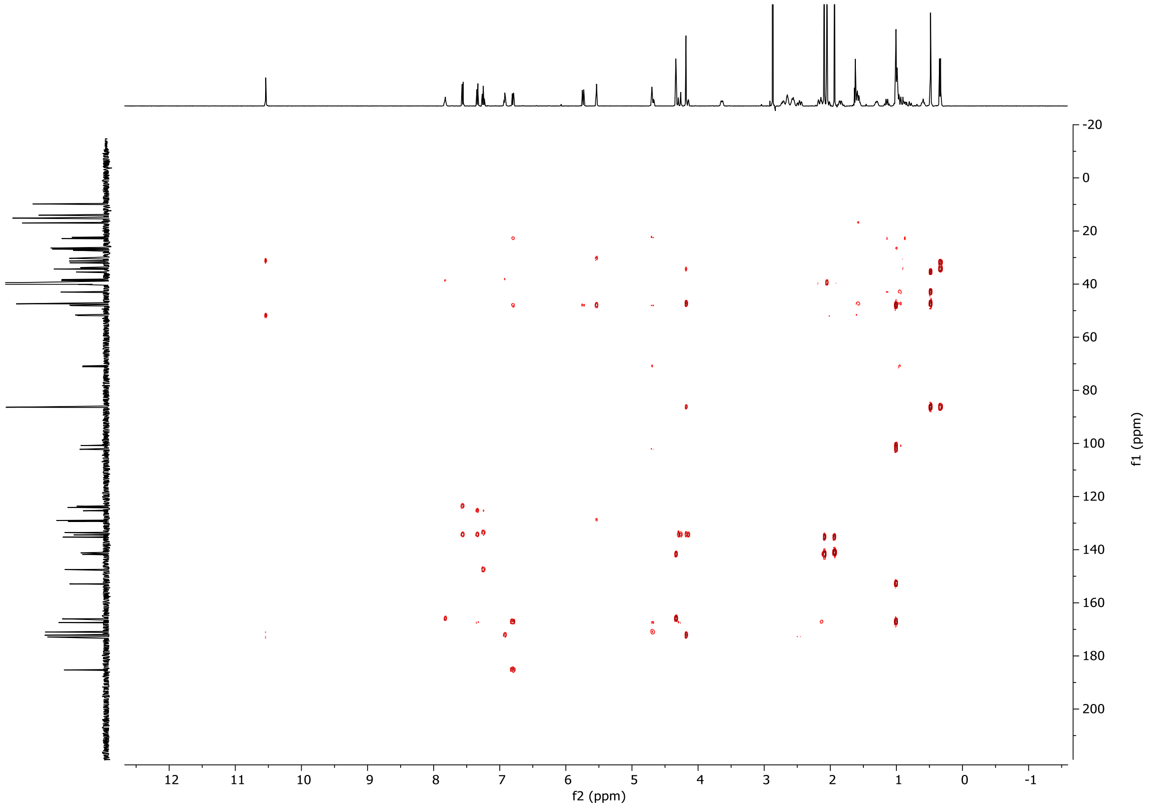

COSY (500 MHz, $(CD_3)_2SO$) of compound **KH-5-298**:

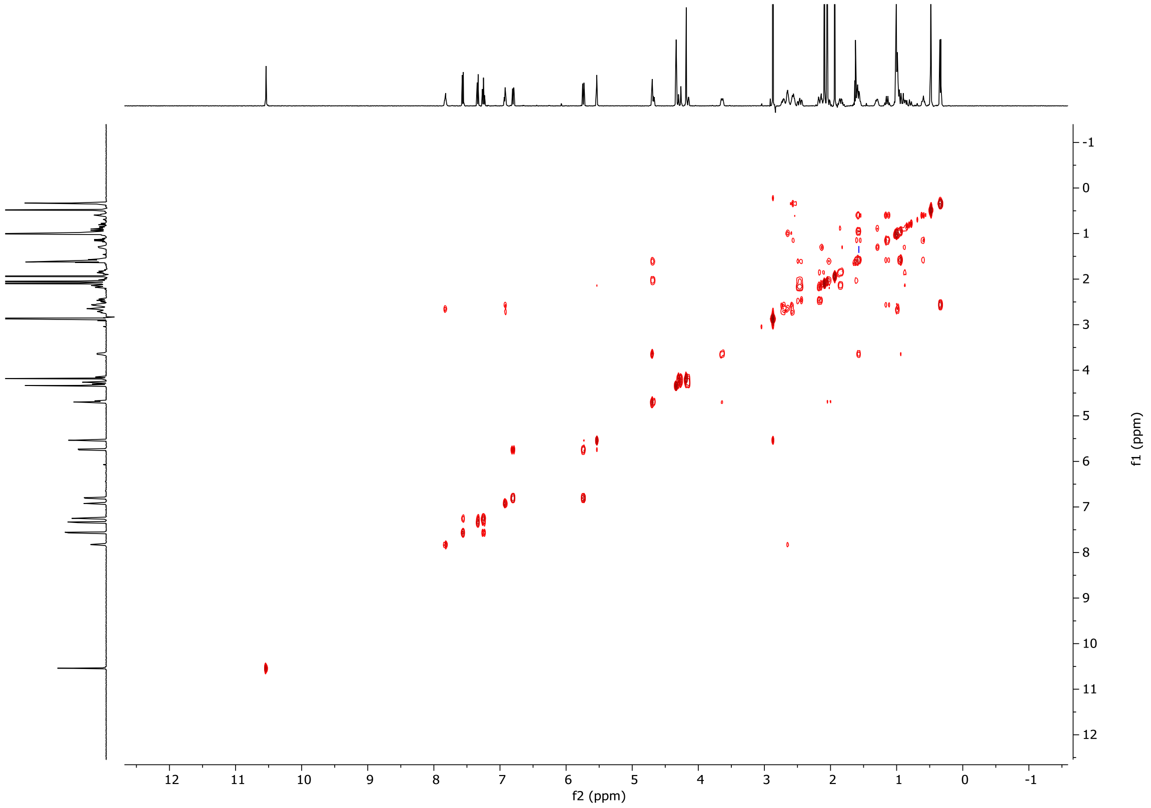

^{1}H NMR (500 MHz, $(CD_3)_2SO$) of compound **KH-5-302**:

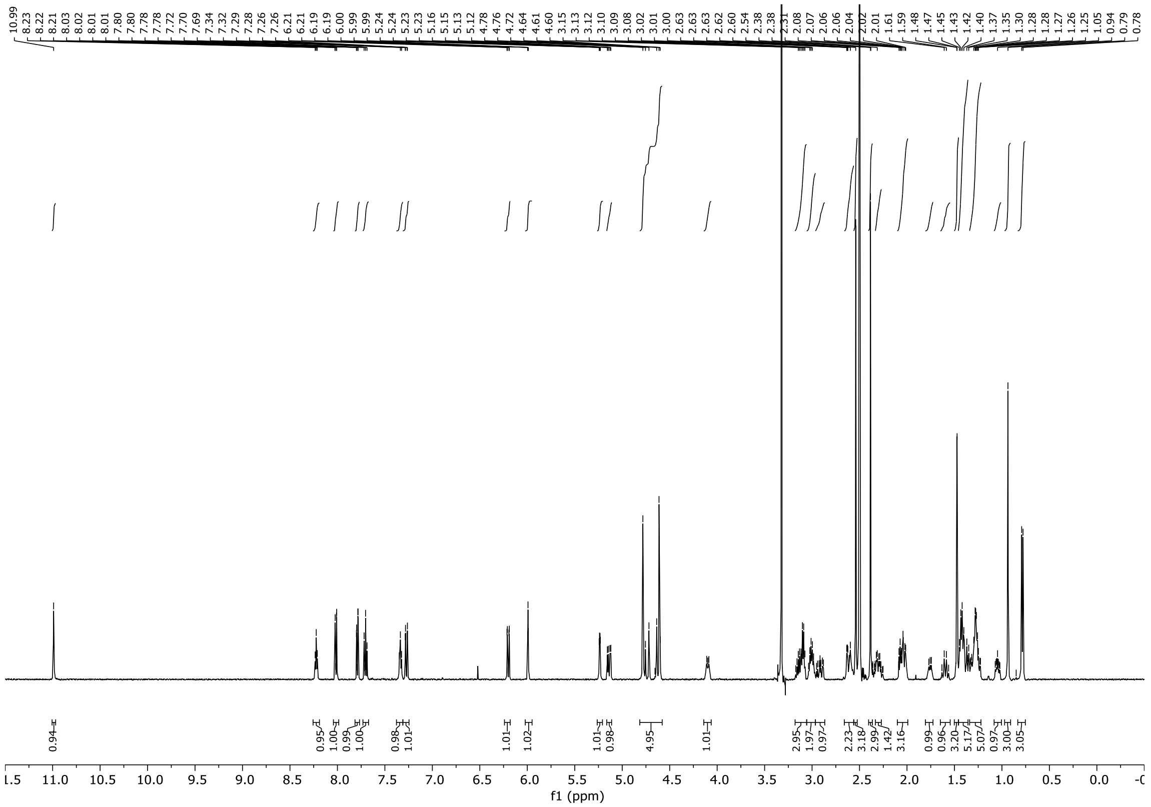

^{13}C NMR (126 MHz, $(CD_3)_2SO$) of compound **KH-5-302**:

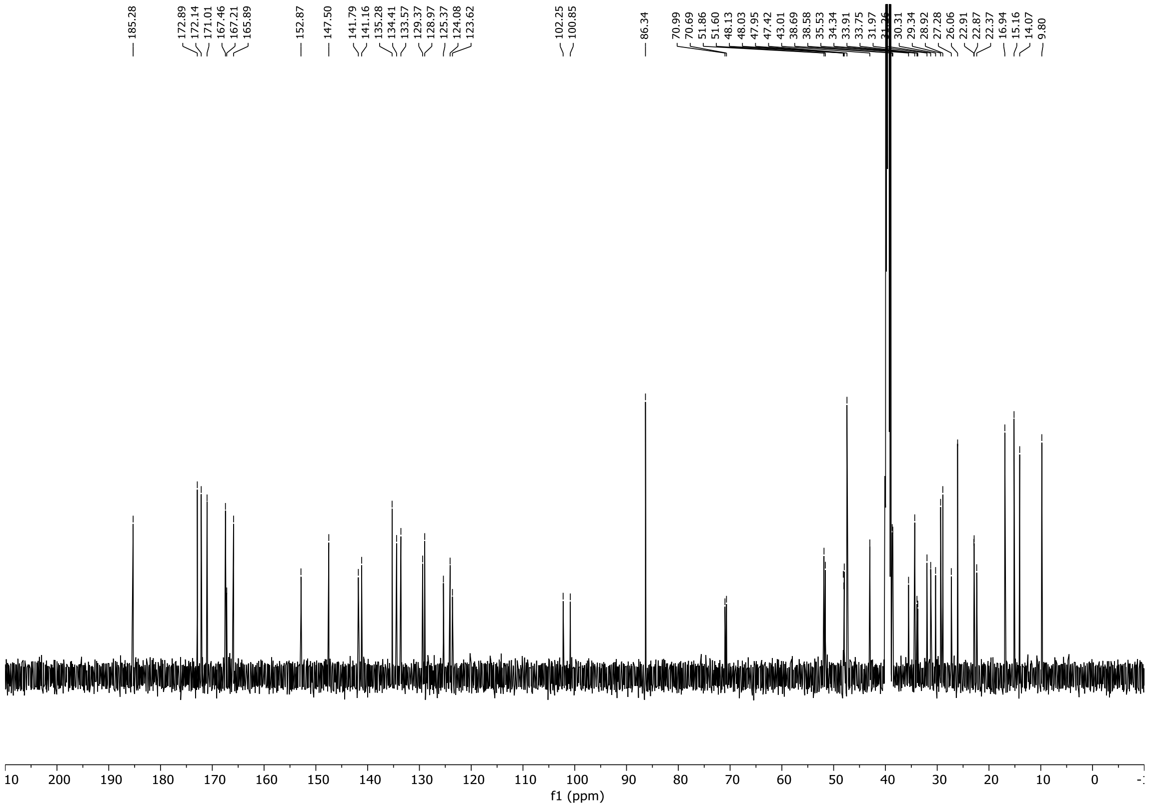

^{19}F NMR (471 MHz, $(CD_3)_2SO$) of compound **KH-5-302**:

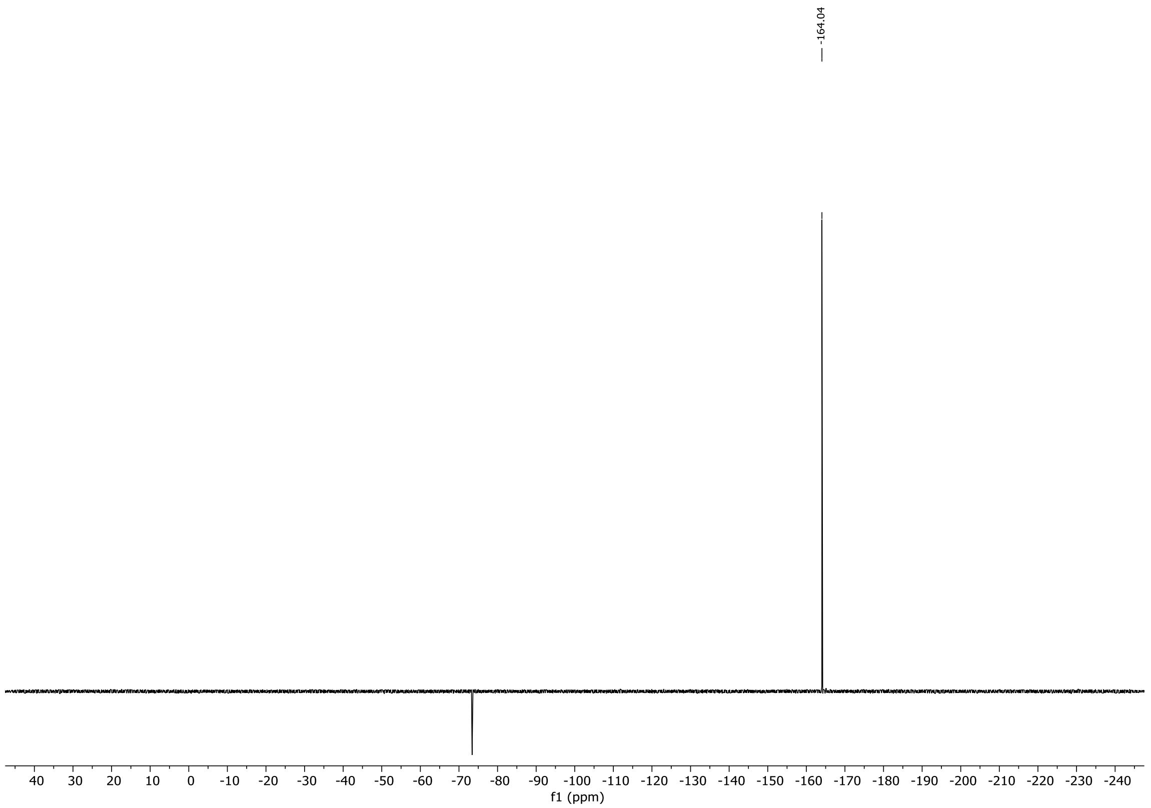

HSQC (500/126 MHz, $(CD_3)_2SO$) of compound **KH-5-302**:

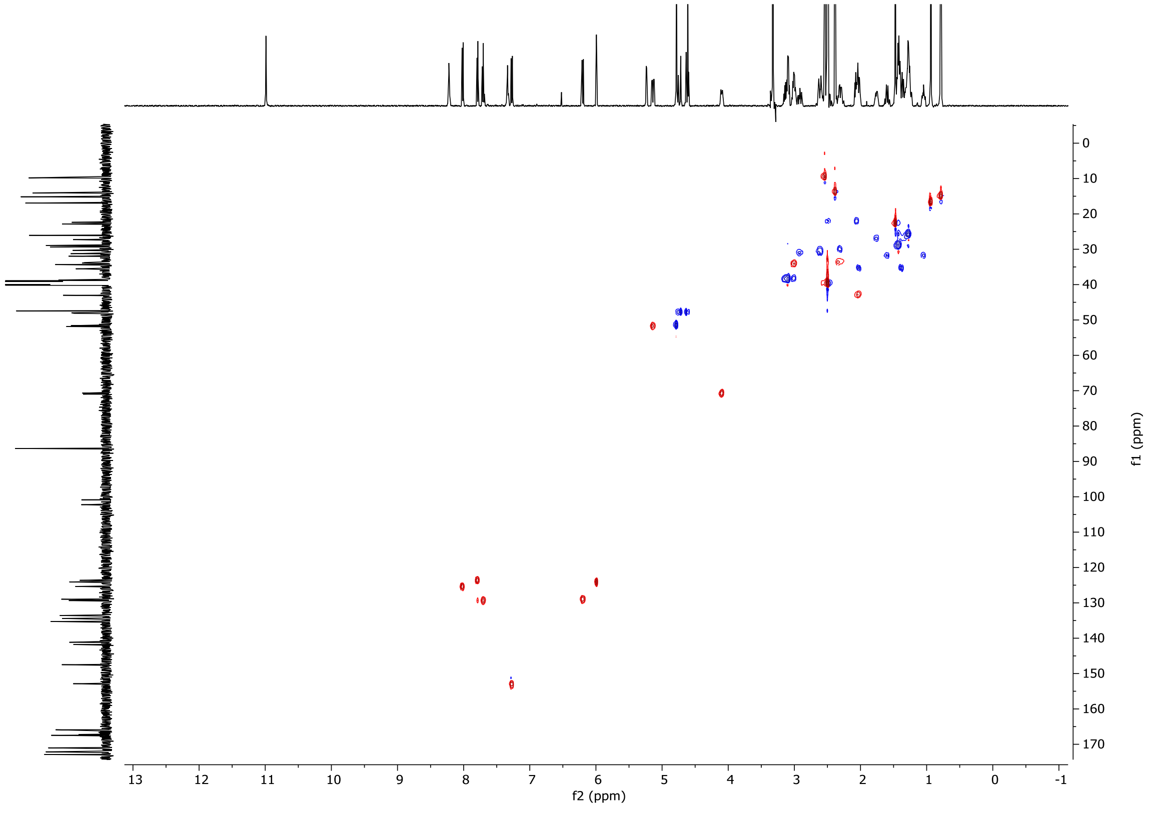

HMBC (500/126 MHz, $(CD_3)_2SO$) of compound **KH-5-302**:

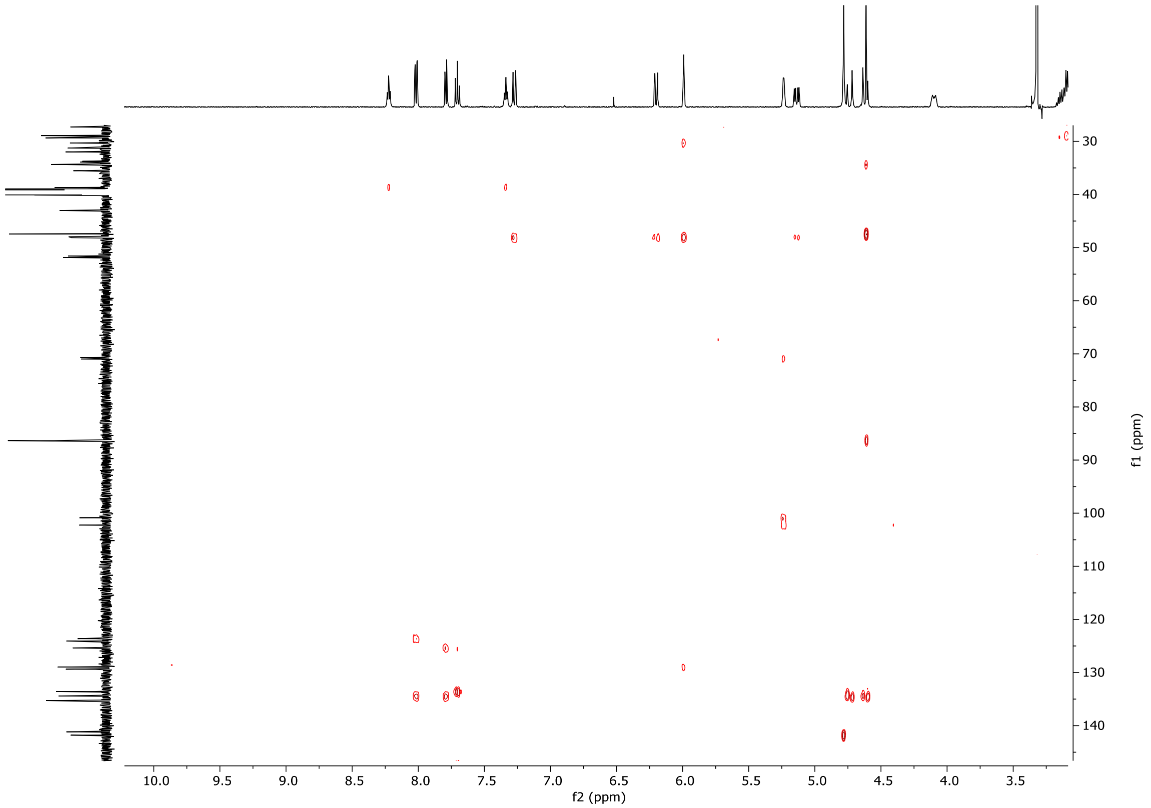

COSY (500 MHz, $(CD_3)_2SO$) of compound **KH-5-302**:

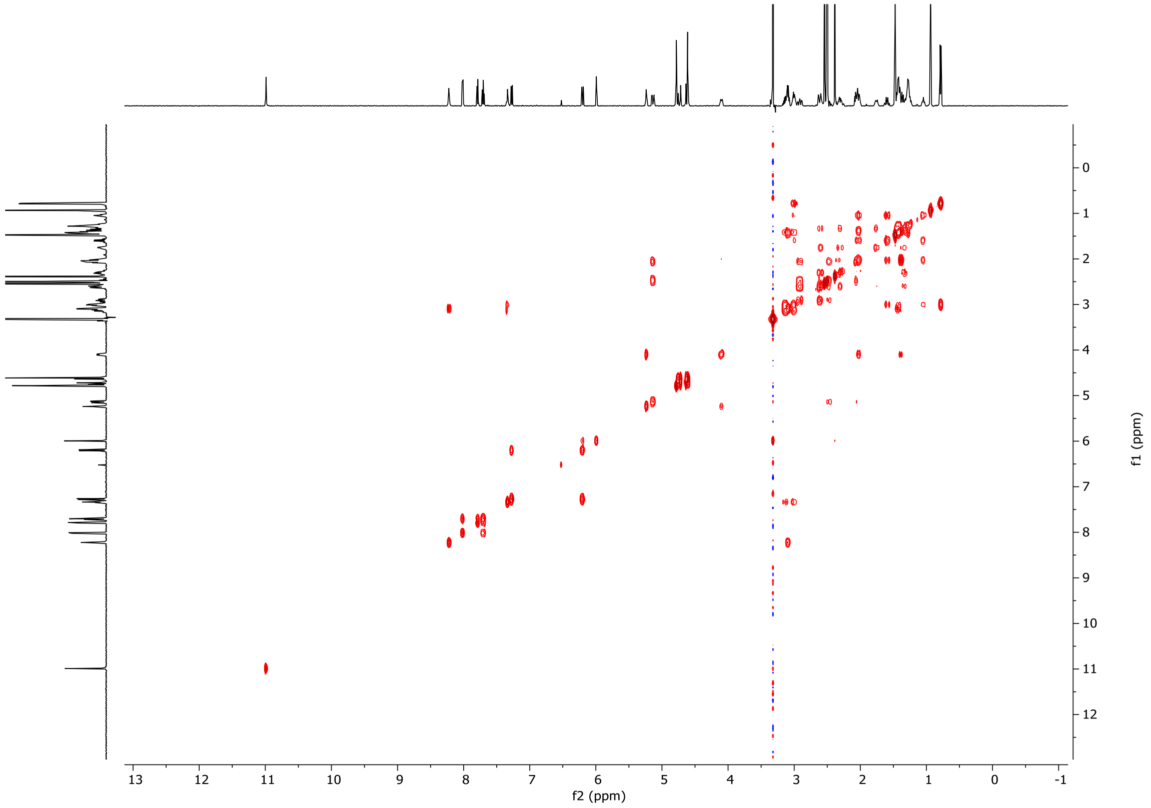

^{1}H NMR (500 MHz, $(CD_3)_2SO$) of compound **KH-5-306**:

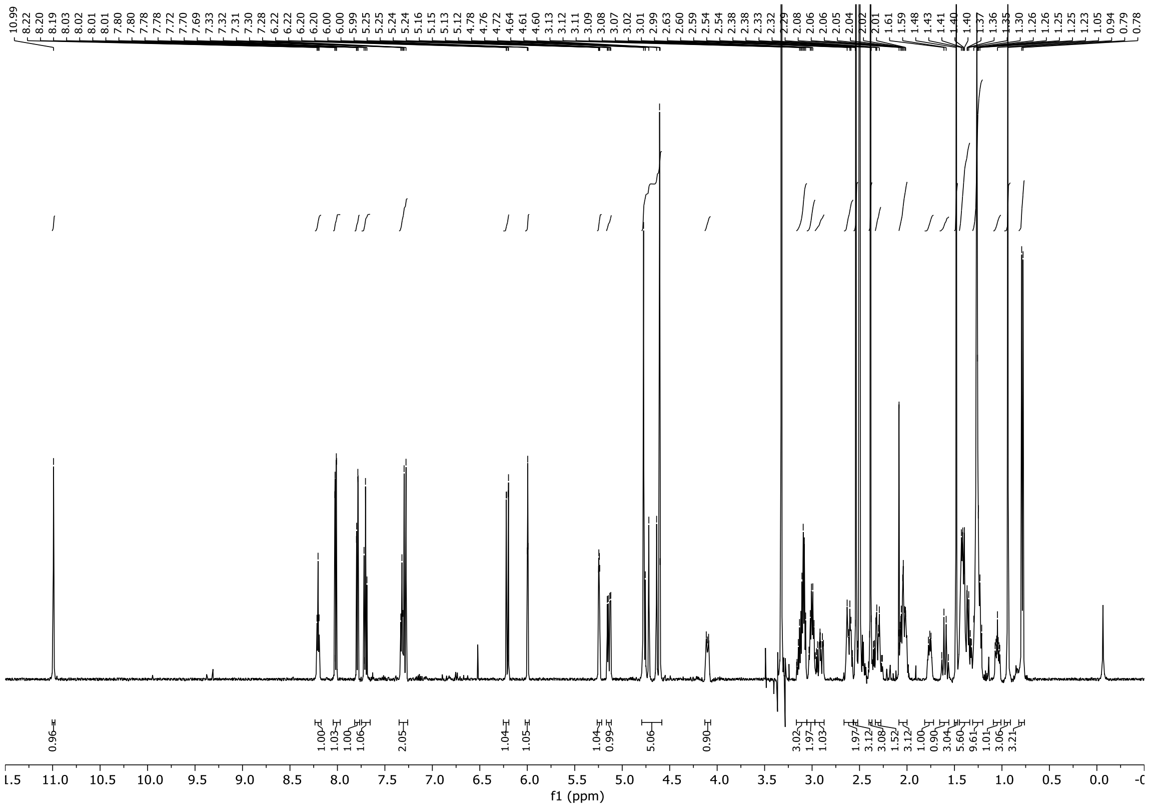

^{13}C NMR (126 MHz, $(CD_3)_2SO$) of compound **KH-5-306**:

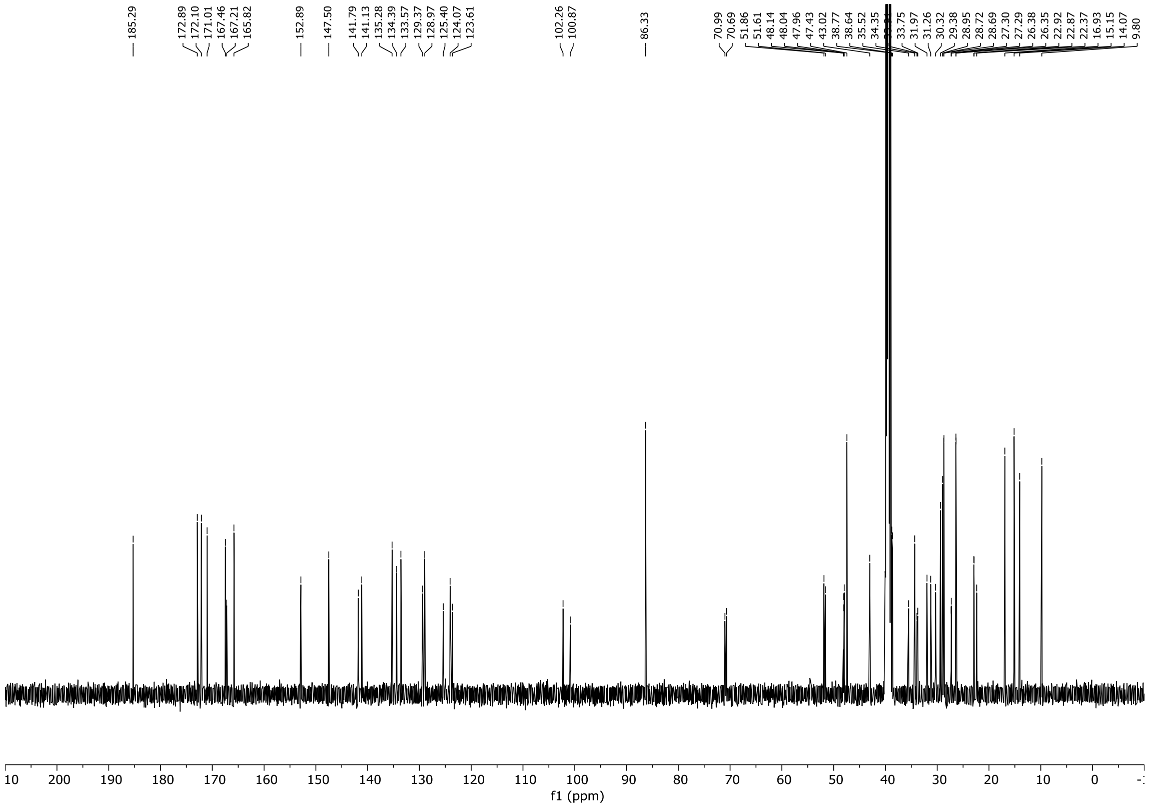

^{19}F NMR (471 MHz, $(CD_3)_2SO$) of compound **KH-5-306**:

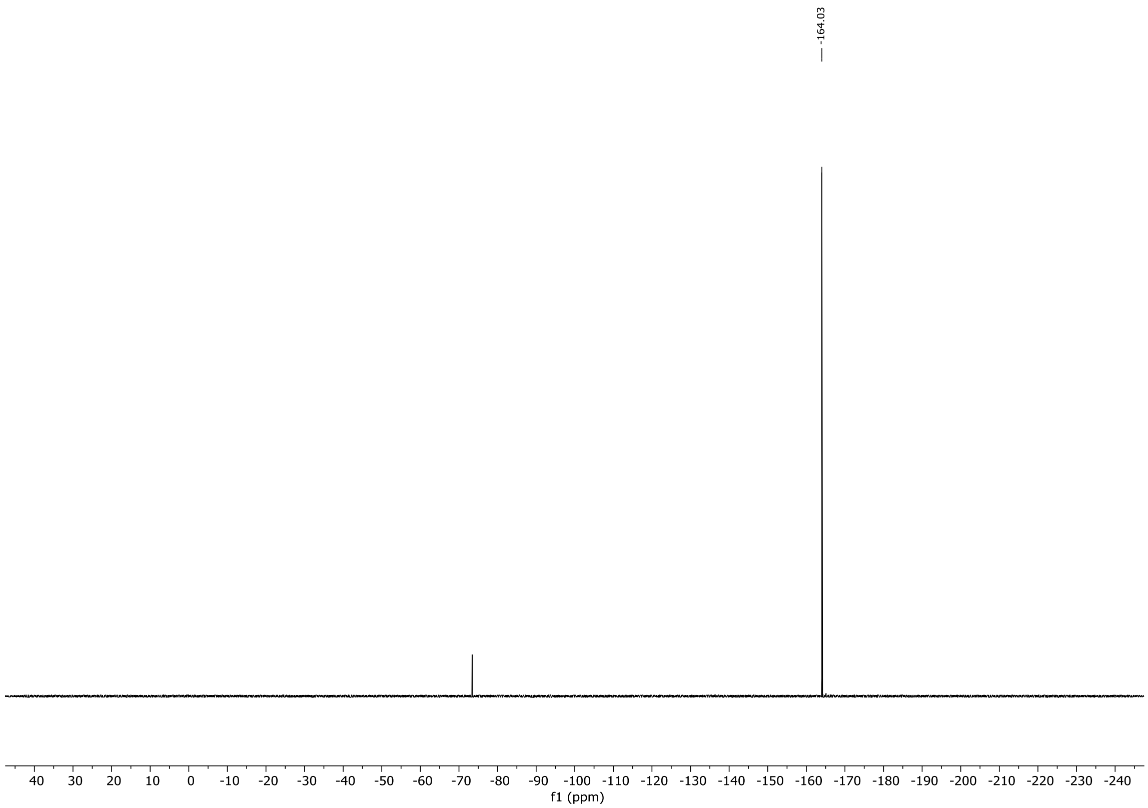

HSQC (500/126 MHz, $(CD_3)_2SO$) of compound **KH-5-306**:

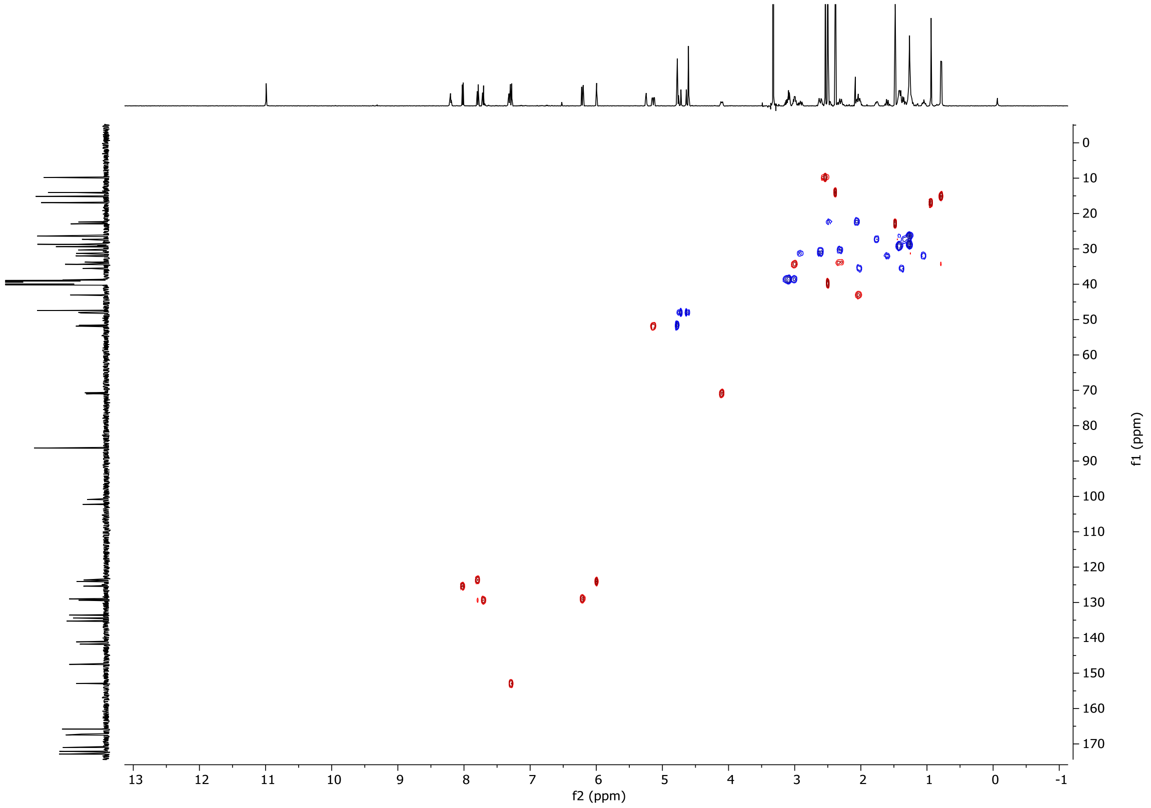

HMBC (500/126 MHz, $(CD_3)_2SO$) of compound **KH-5-306**:

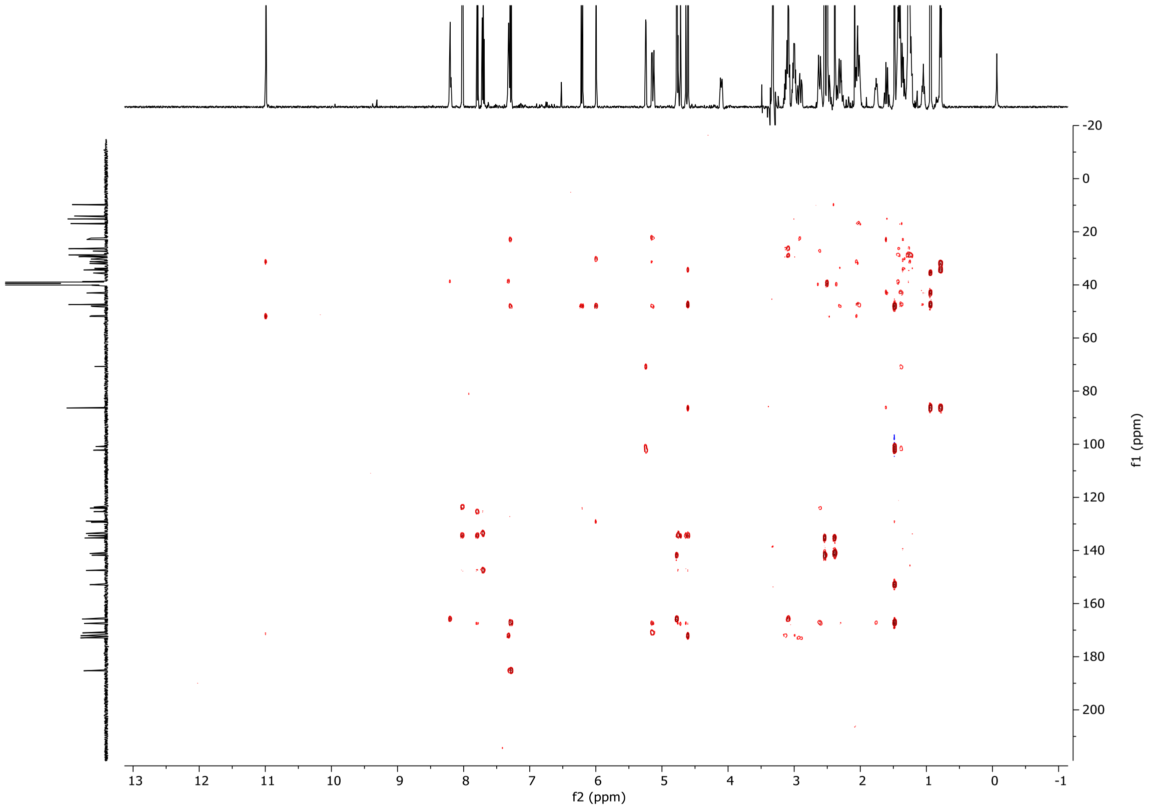

COSY (500 MHz, $(CD_3)_2SO$) of compound **KH-5-306**:

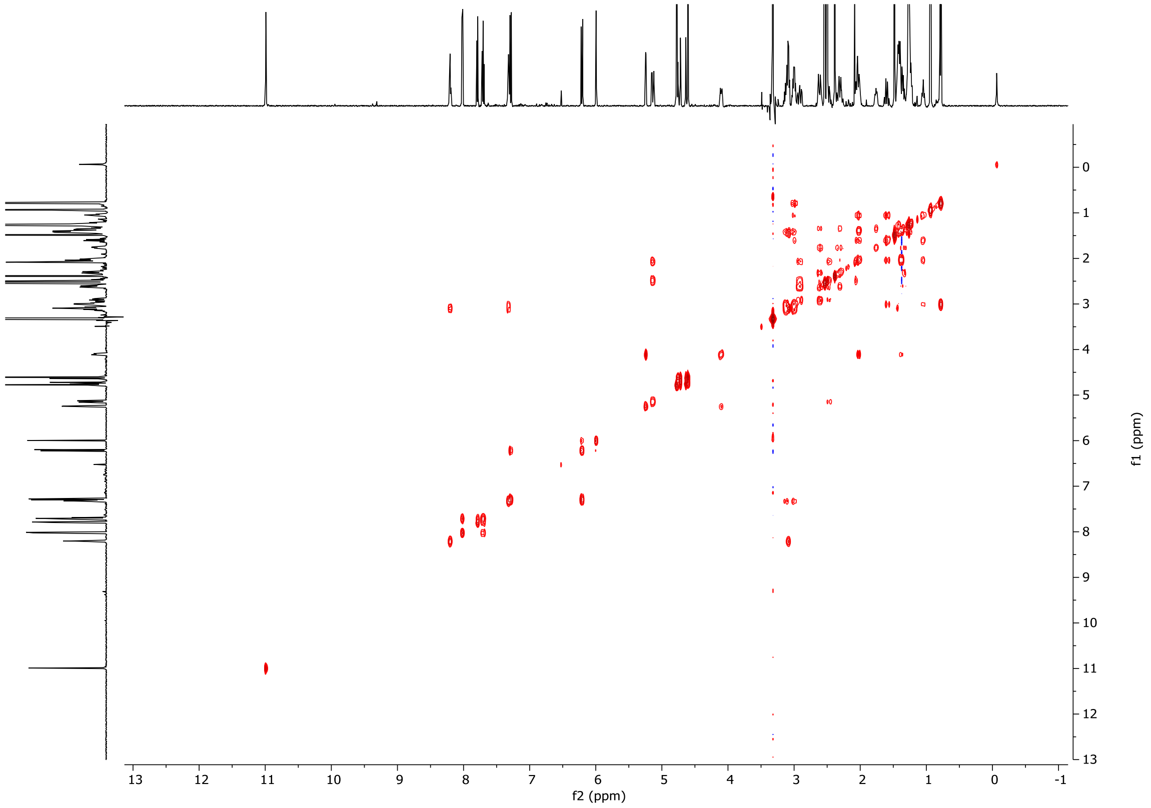

^{1}H NMR (500 MHz, $(CD_3)_2SO$) of compound **KH-5-309**:

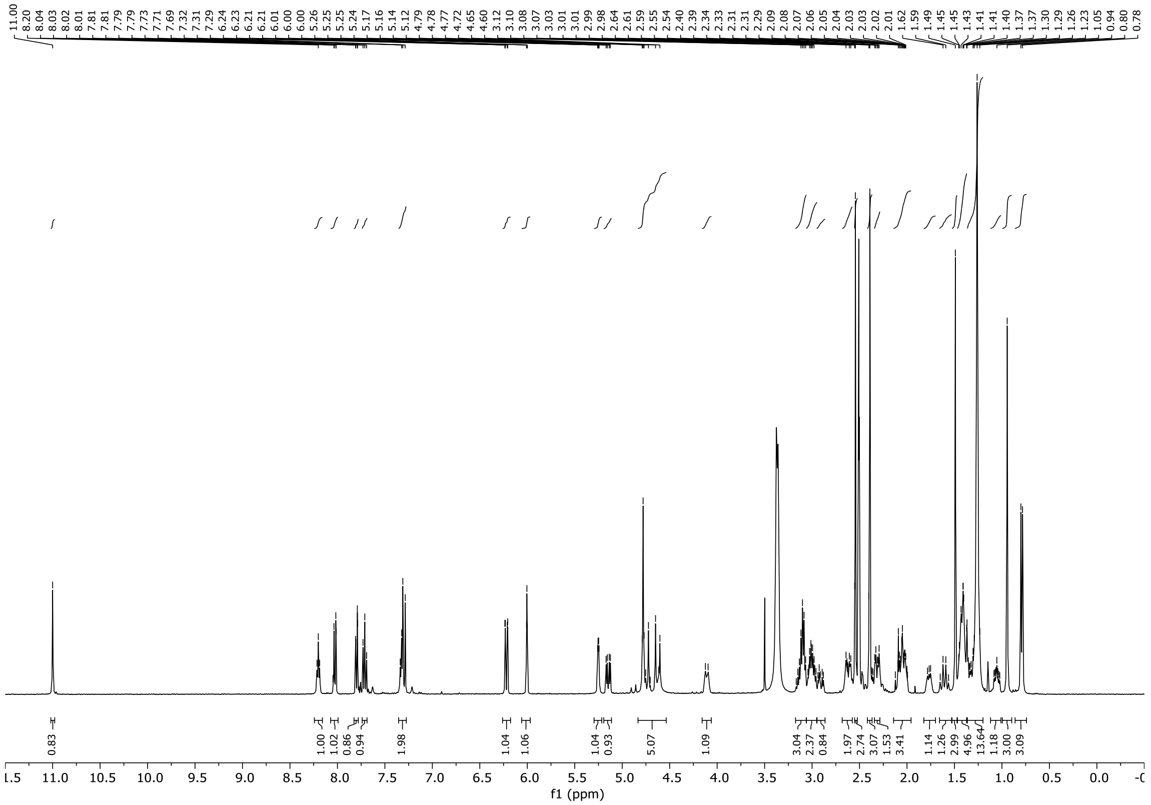

^{13}C NMR (126 MHz, $(CD_3)_2SO$) of compound **KH-5-309**:

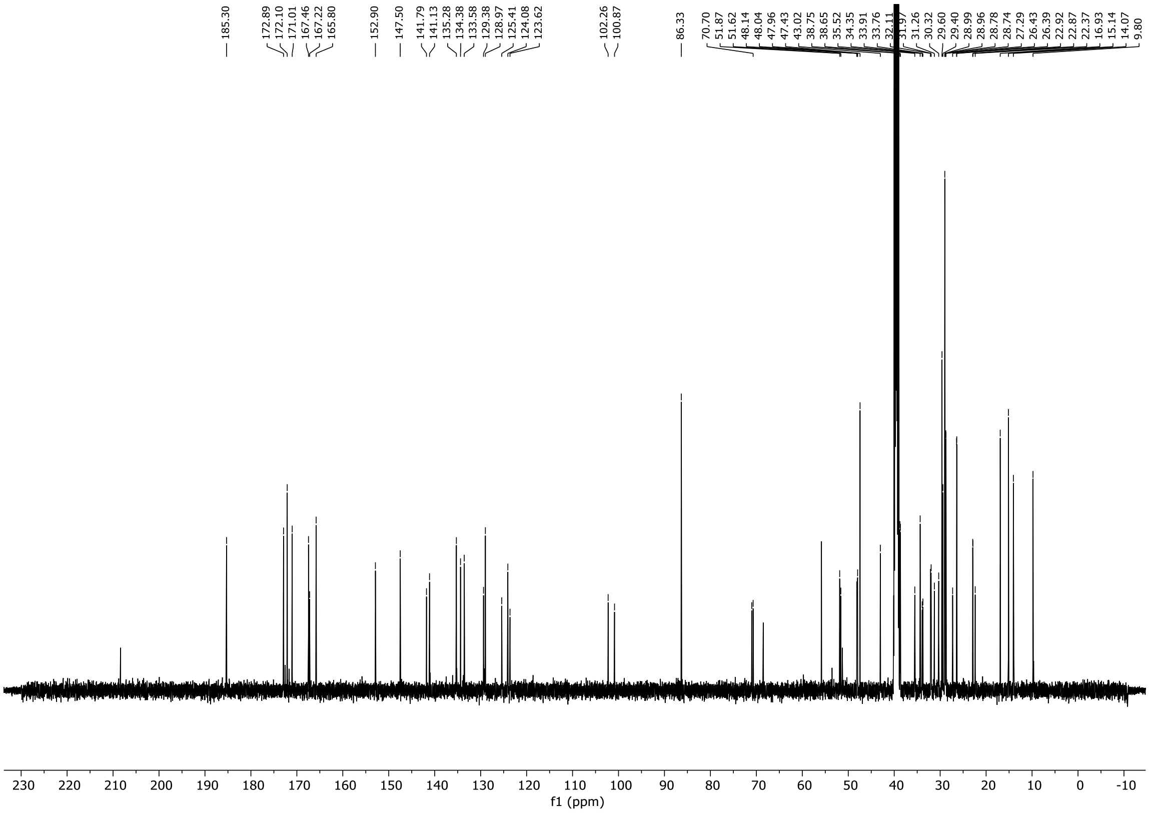

^{19}F NMR (471 MHz, $(CD_3)_2SO$) of compound **KH-5-309**:

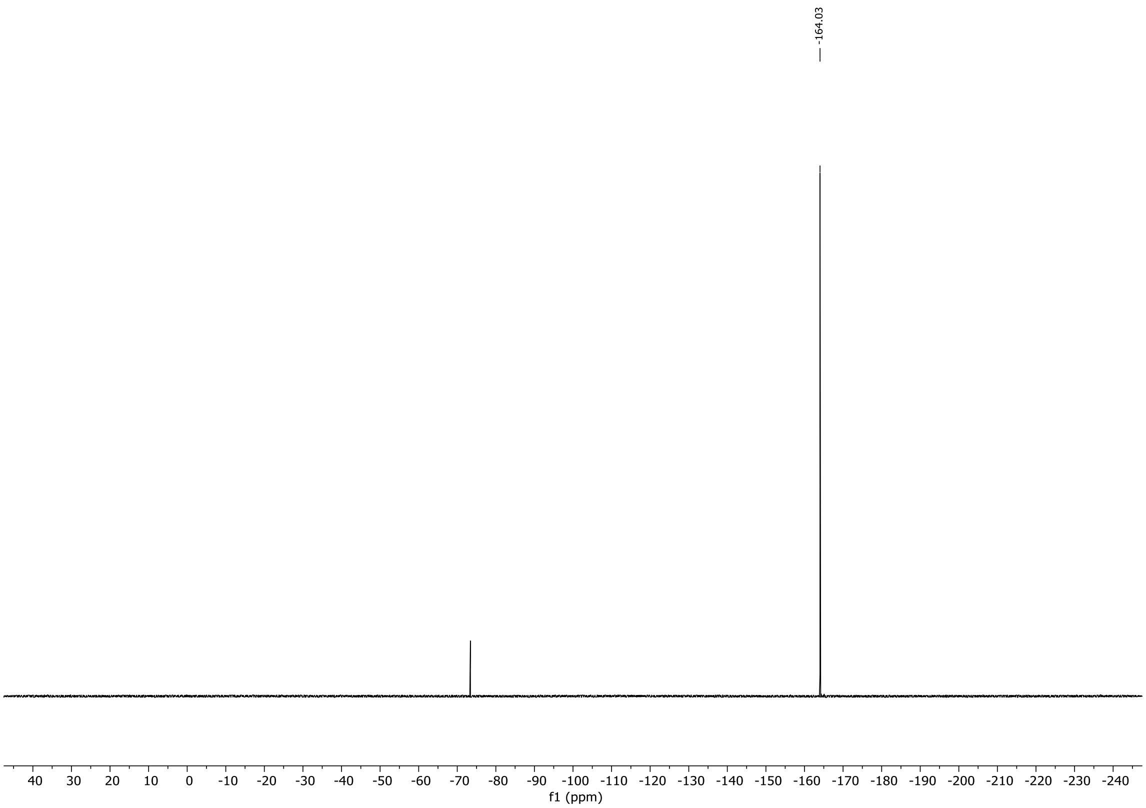

HSQC (500/126 MHz, $(CD_3)_2SO$) of compound **KH-5-309**:

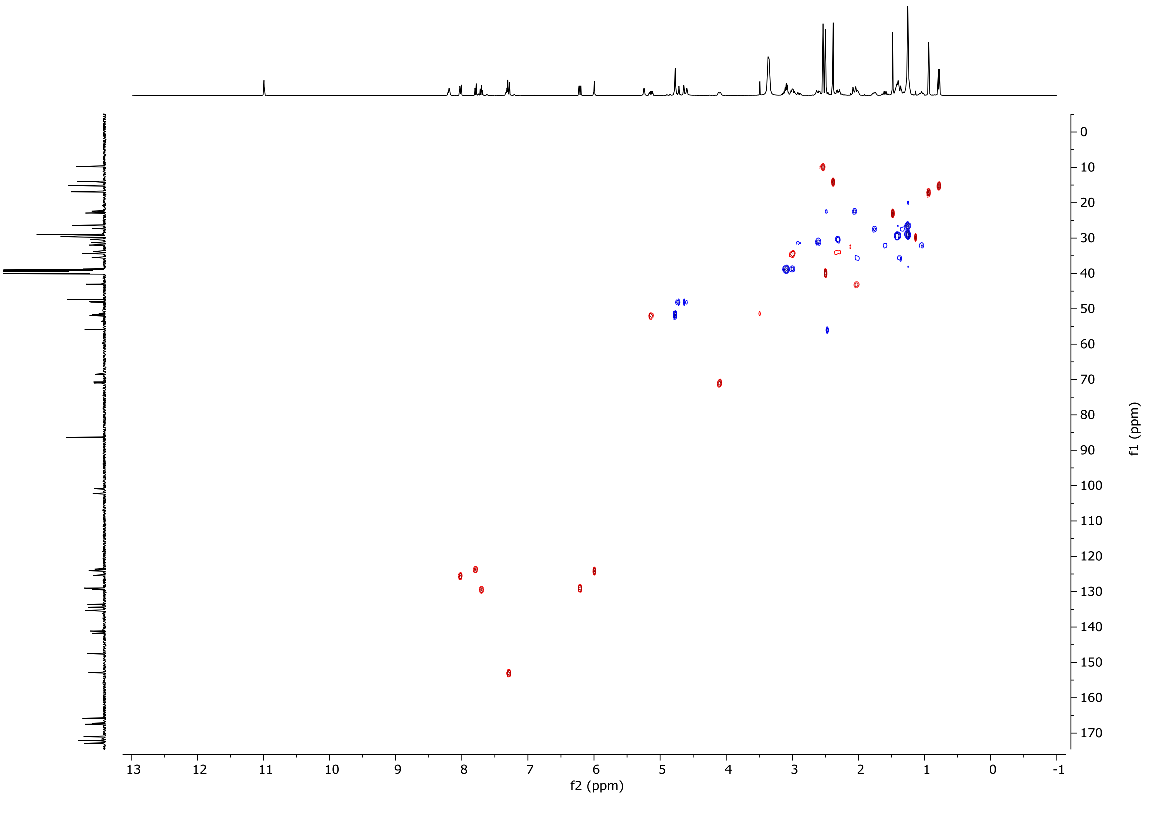

HMBC (500/126 MHz, $(CD_3)_2SO$) of compound **KH-5-309**:

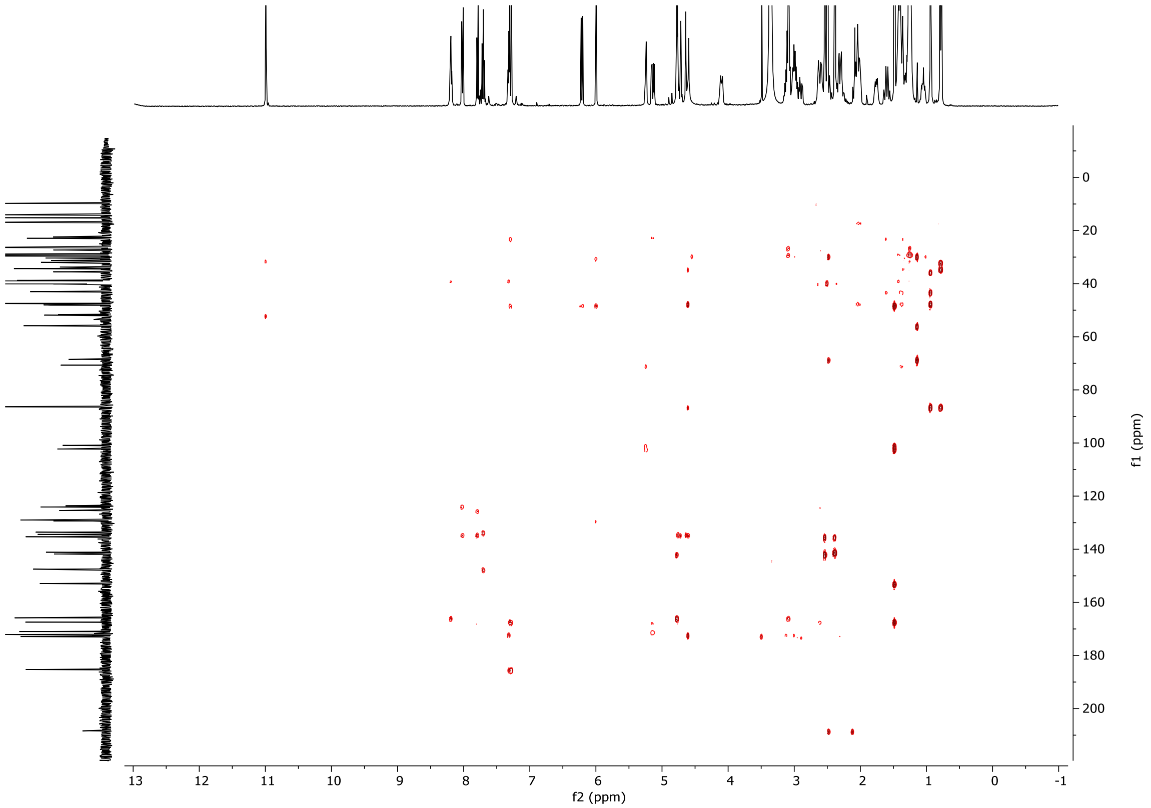

COSY (500 MHz, $(CD_3)_2SO$) of compound **KH-5-309**:

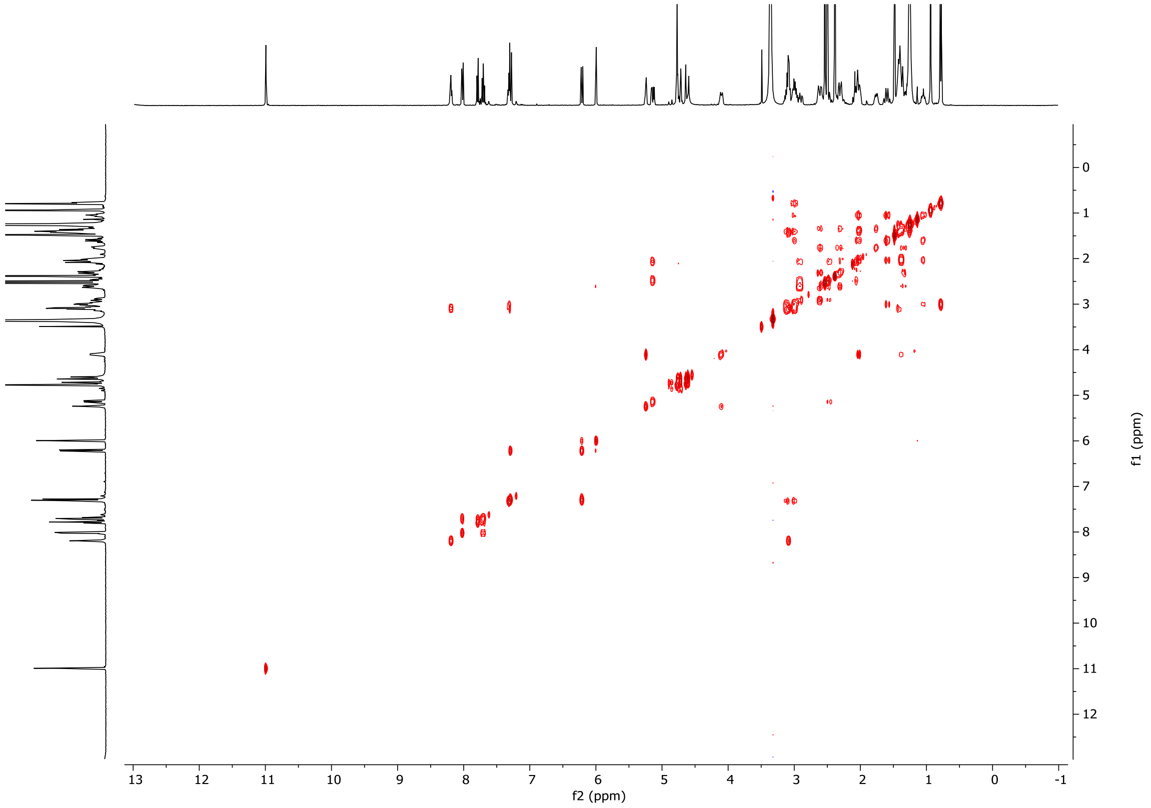

<u>$(CF_3)_2$-Arylazopyrazole photoPROTACs</u>

^1H NMR (600 MHz, $(CD_3)_2SO$) of compound **44**:

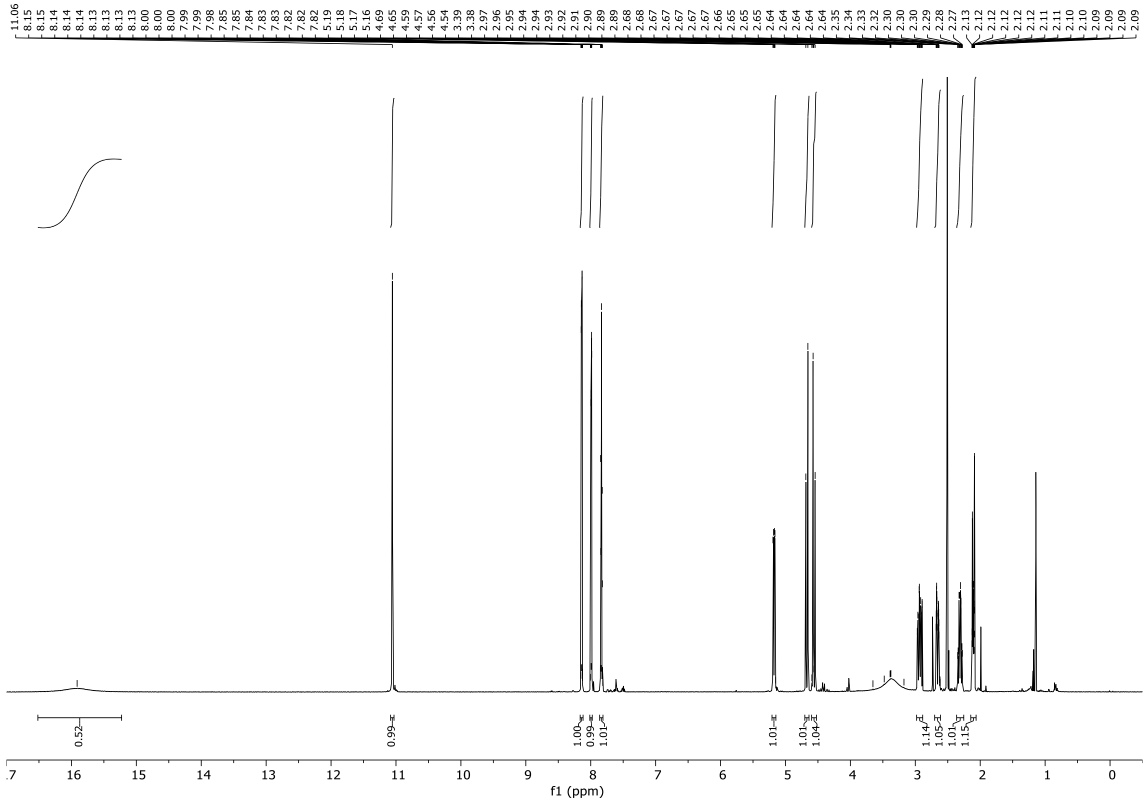

^{13}C NMR (151 MHz, $(CD_3)_2SO$) of compound **44**:

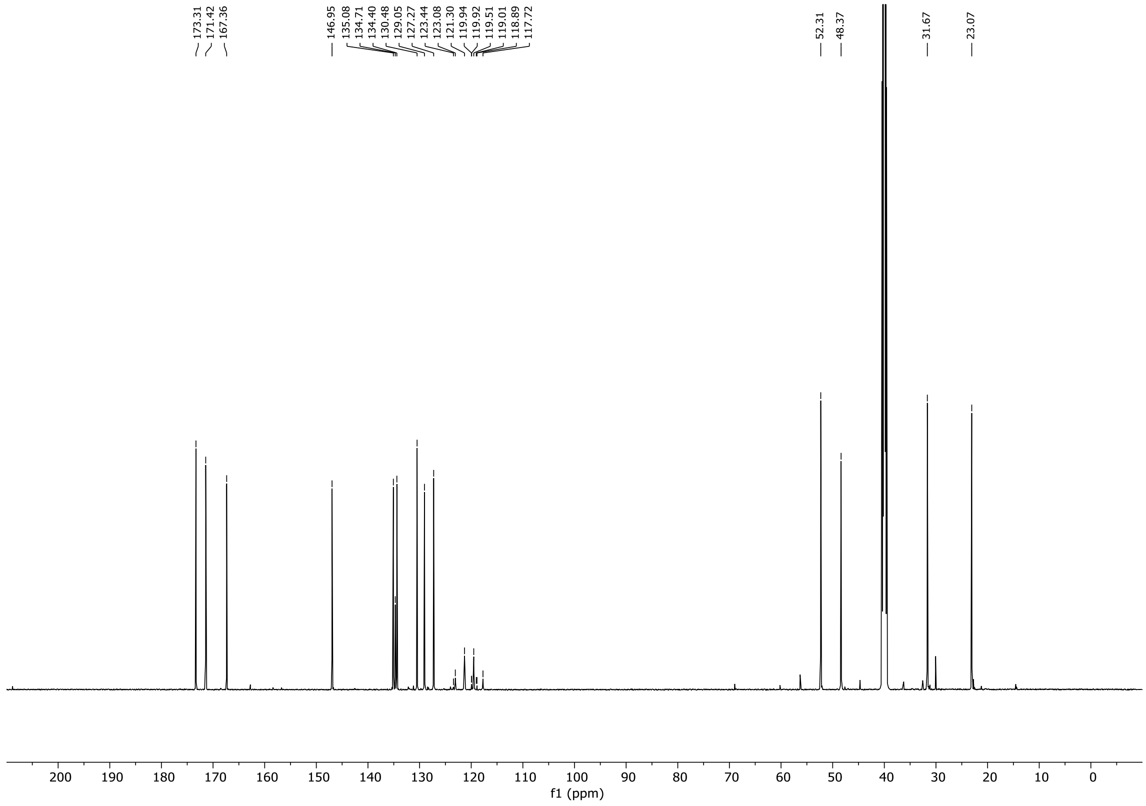

^{19}F NMR (565 MHz, $(CD_3)_2SO$) of compound **44**:

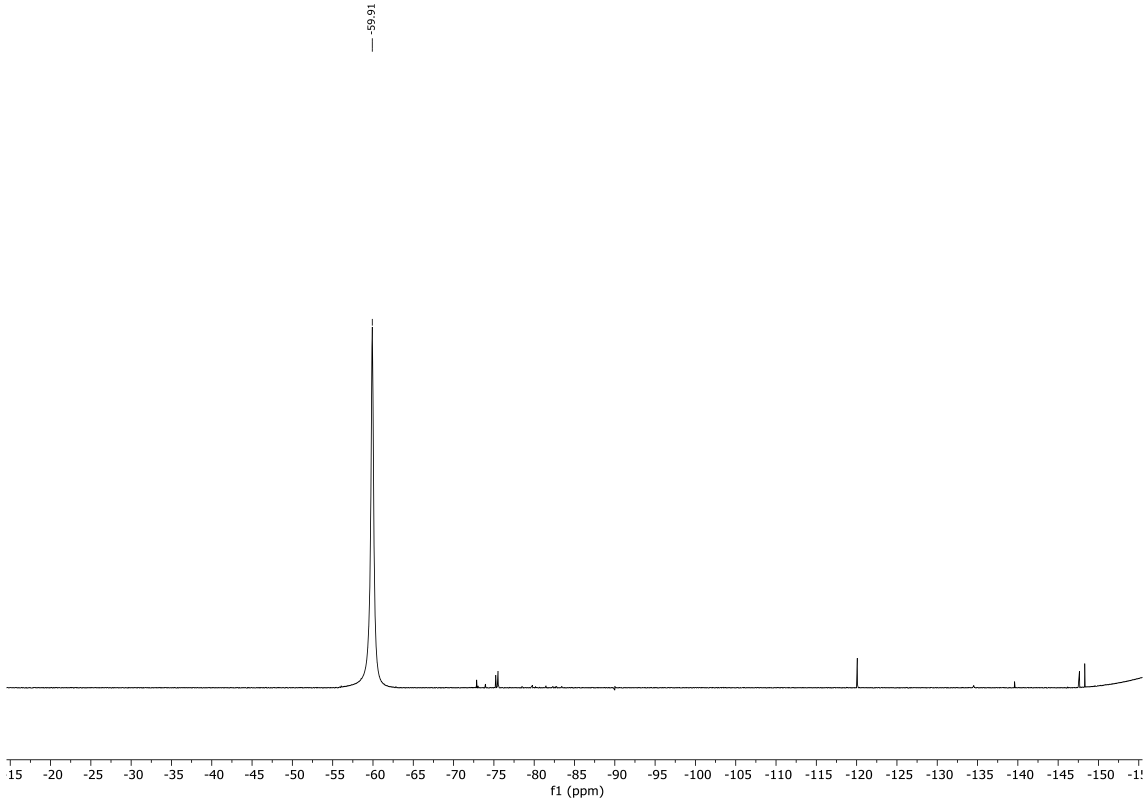

HSQC (600/151 MHz, $(CD_3)_2SO$) of compound **44**:

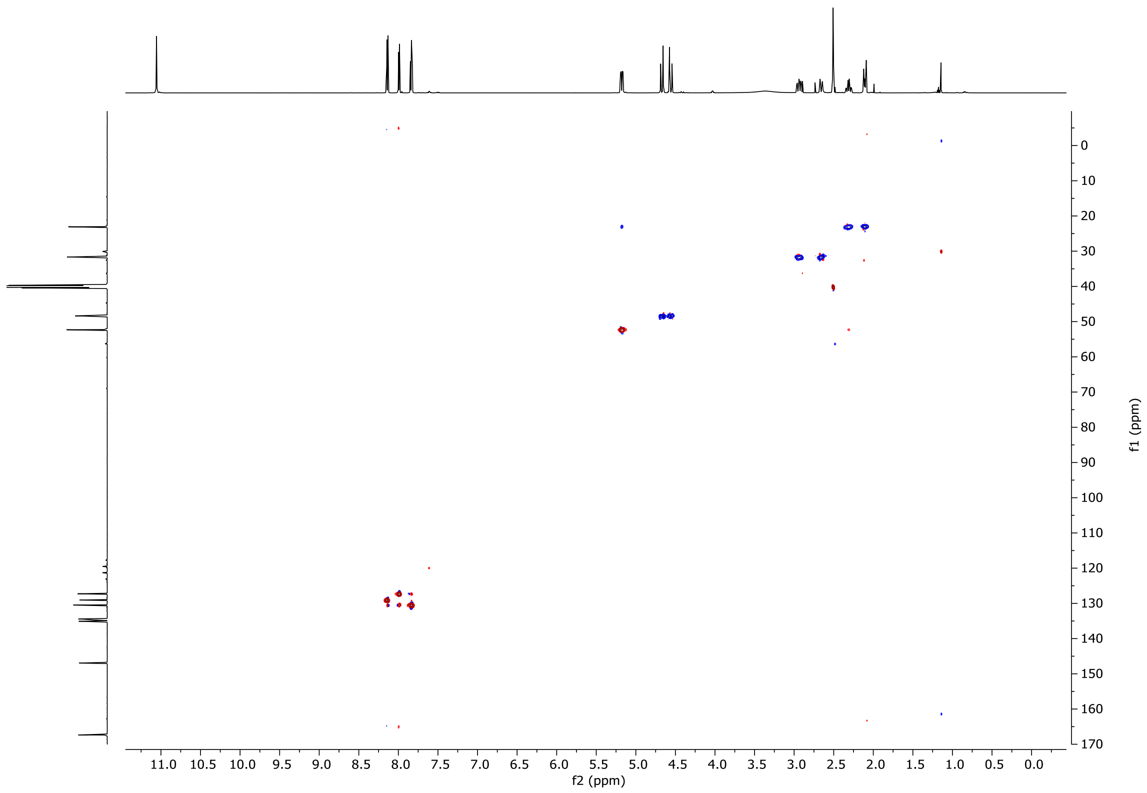

HMBC (600/151 MHz, $(CD_3)_2SO$) of compound **44**:

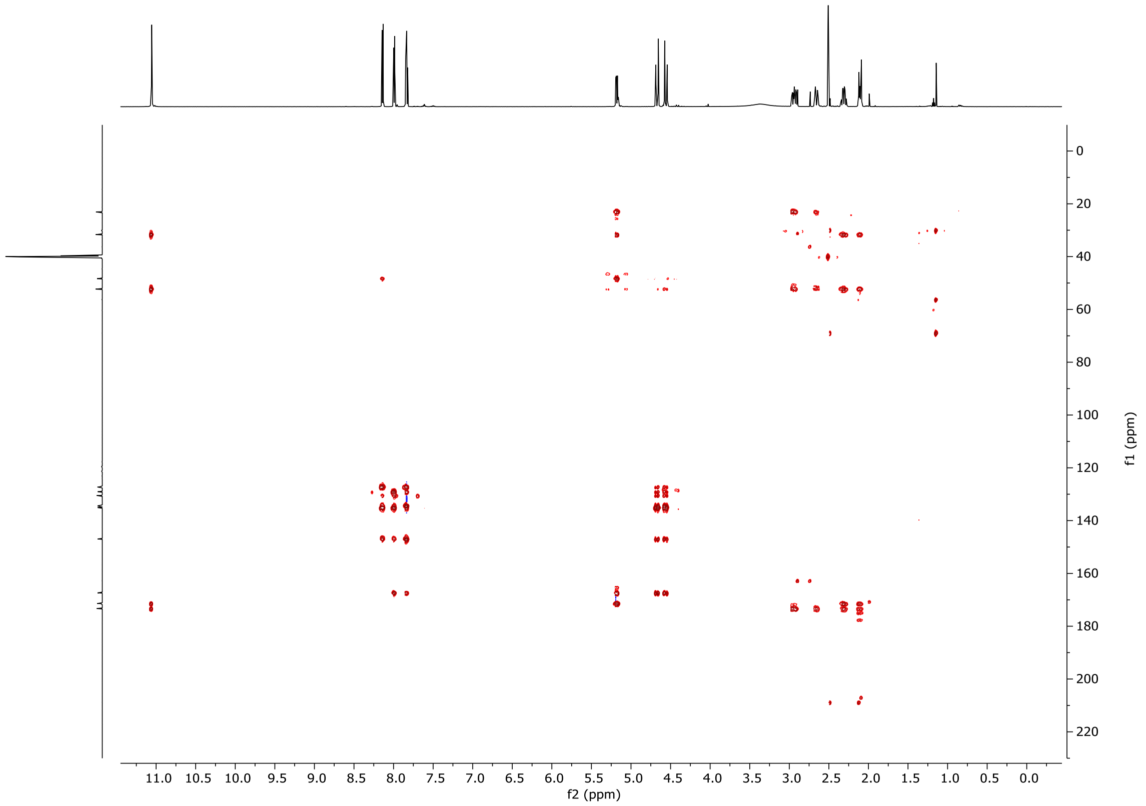

COSY (600 MHz, $(CD_3)_2SO$) of compound **44**:

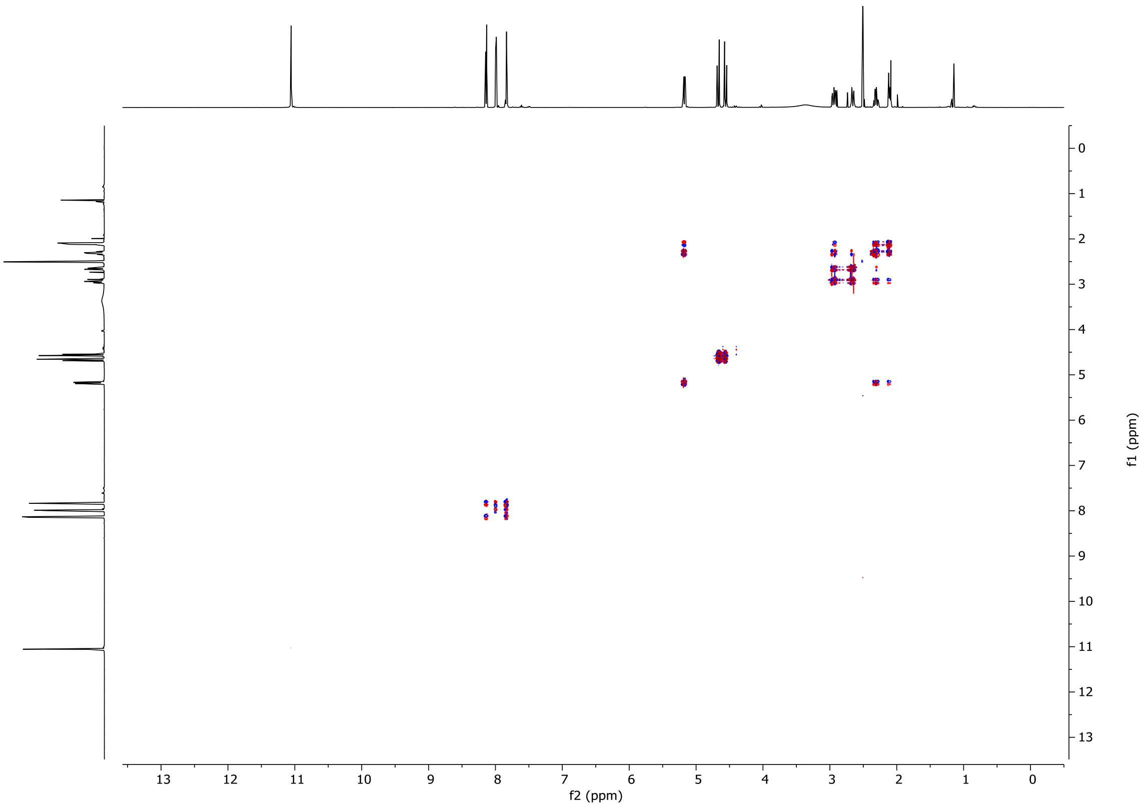

^{1}H NMR (400 MHz, $CDCl_3$) of compound **43**:

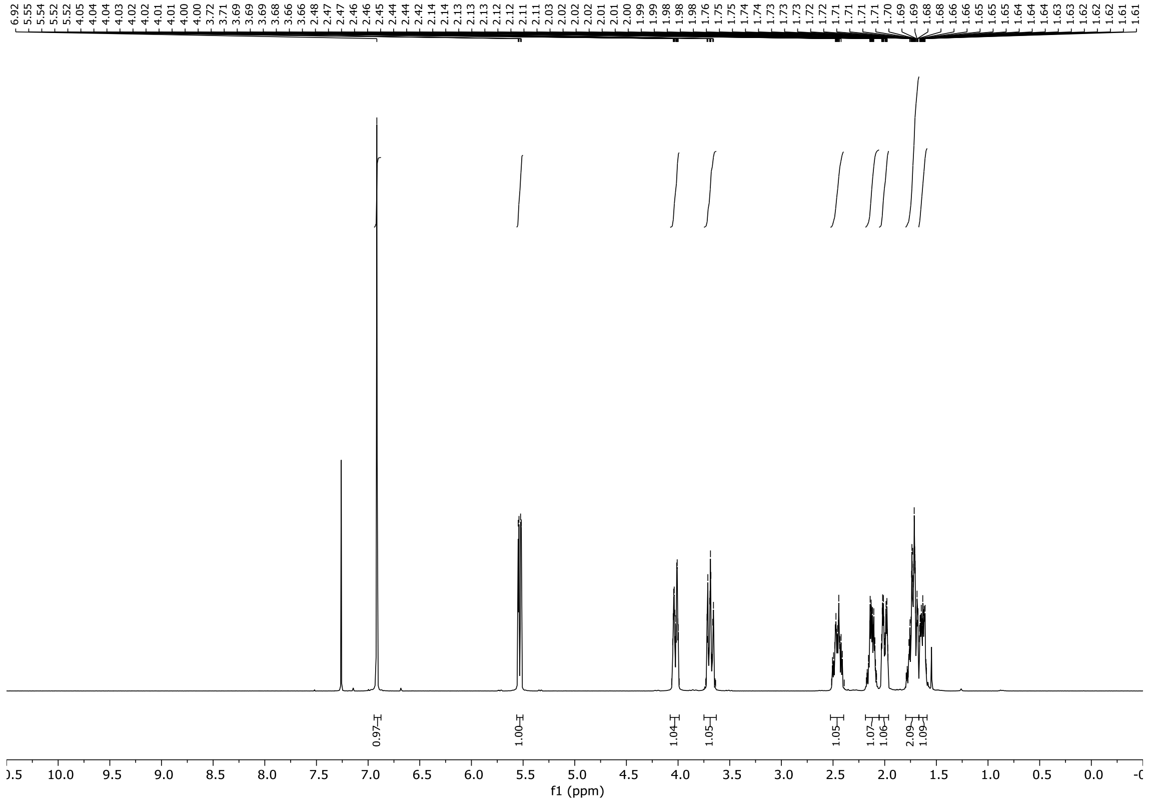

^{13}C NMR (101 MHz, $CDCl_3$) of compound **43**:

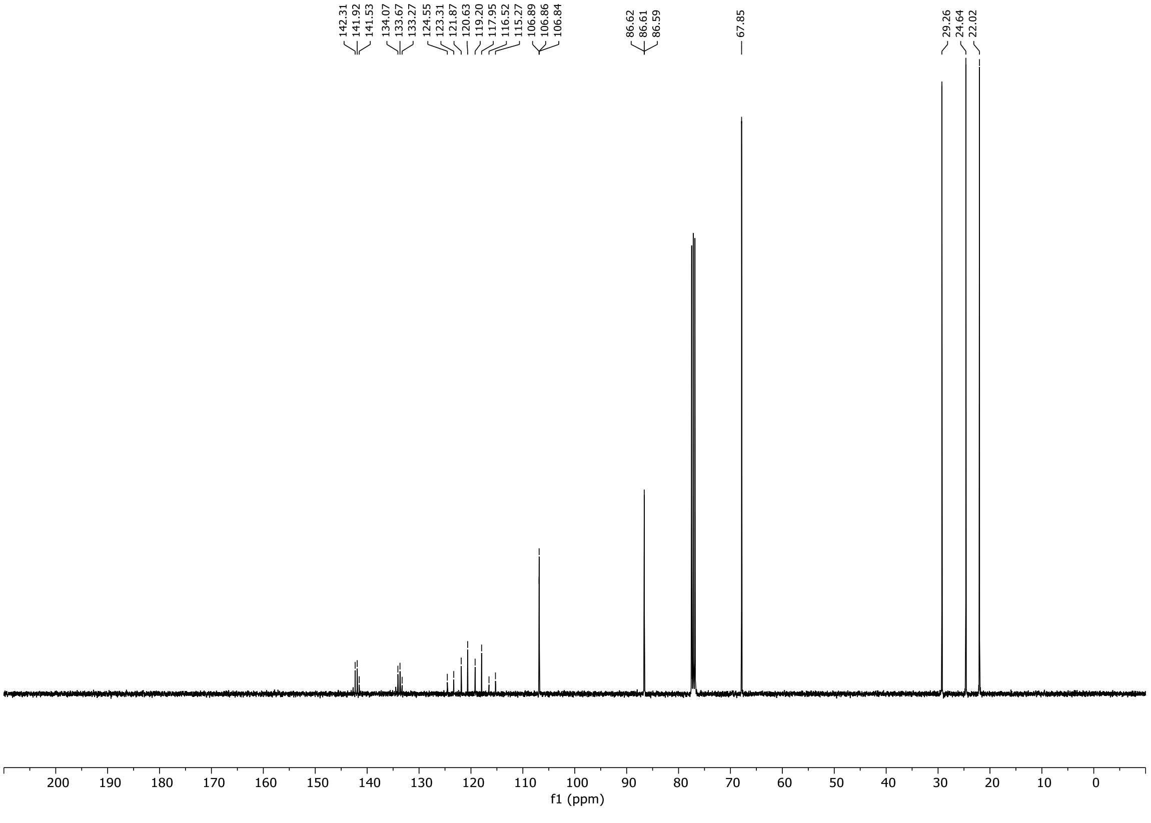

^{19}F NMR (376 MHz, $CDCl_3$) of compound **43**:

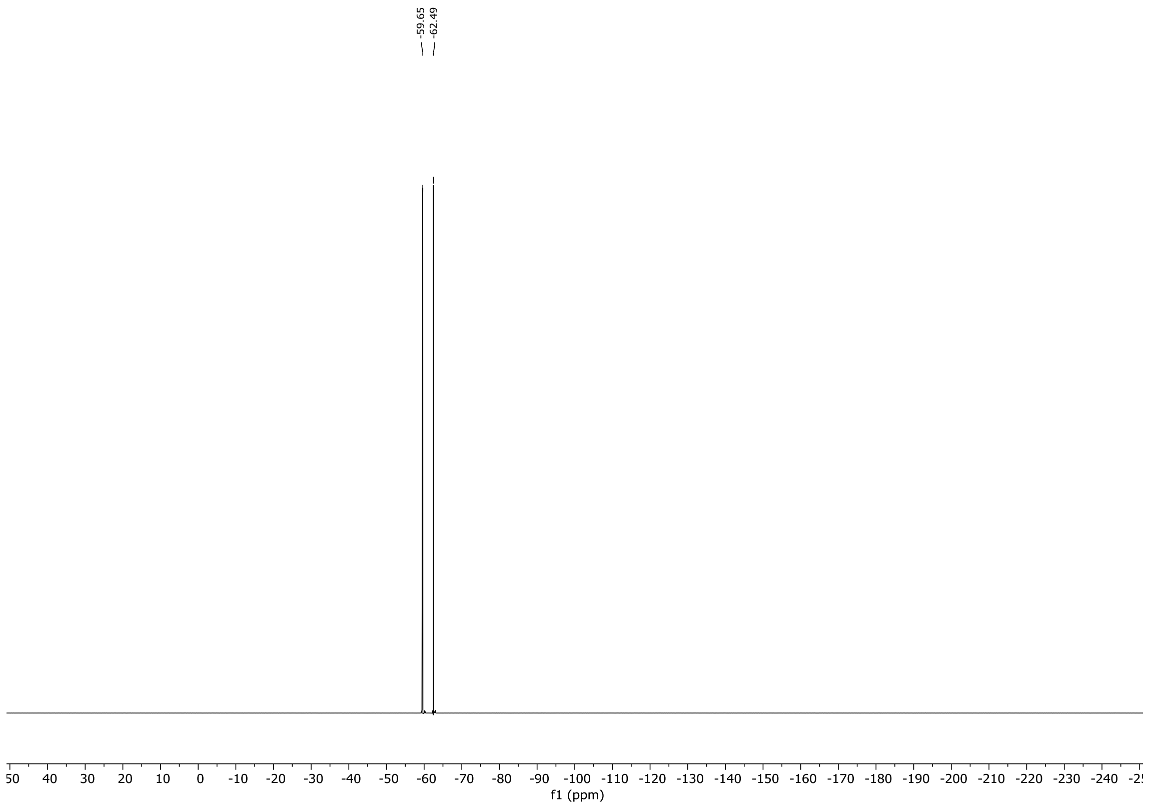

^{1}H NMR (500 MHz, $(CD_3)_2CO$) of compound **$(CF_3)_2$-arylazopyrazole photoswitch**:

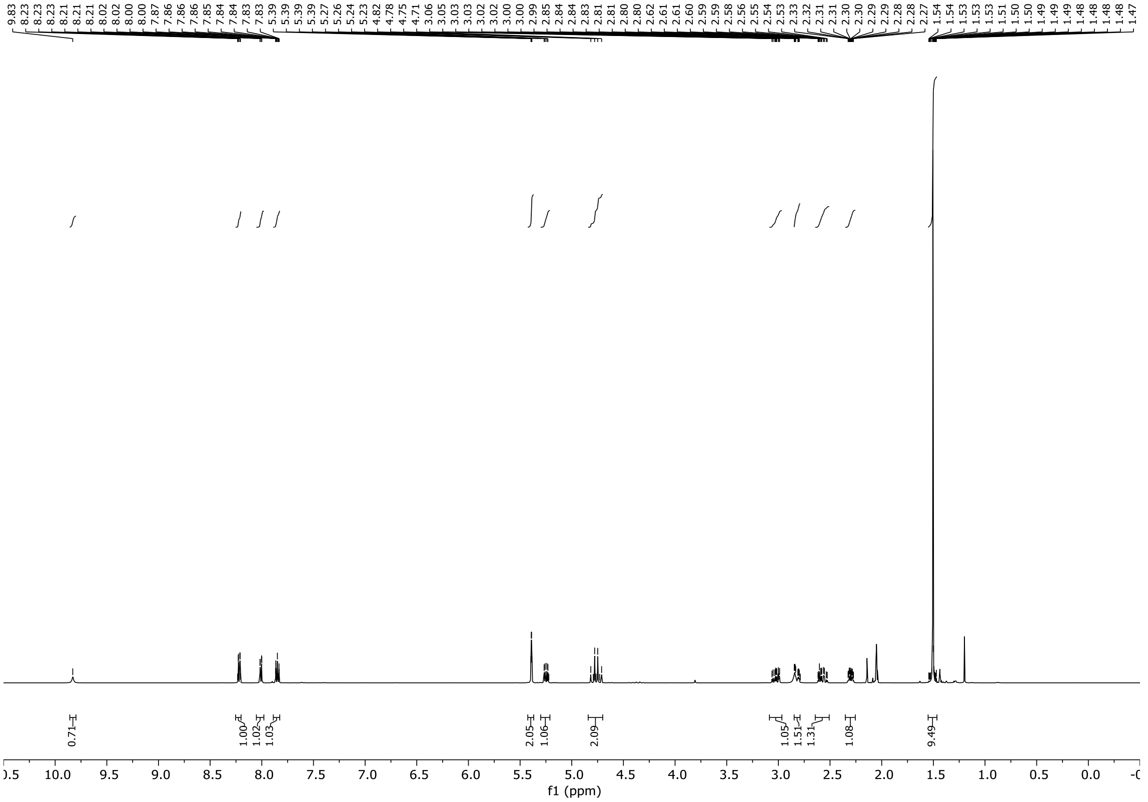

^{13}C NMR (126 MHz, $(CD_3)_2CO$) of compound **$(CF_3)_2$-arylazopyrazole photoswitch**:

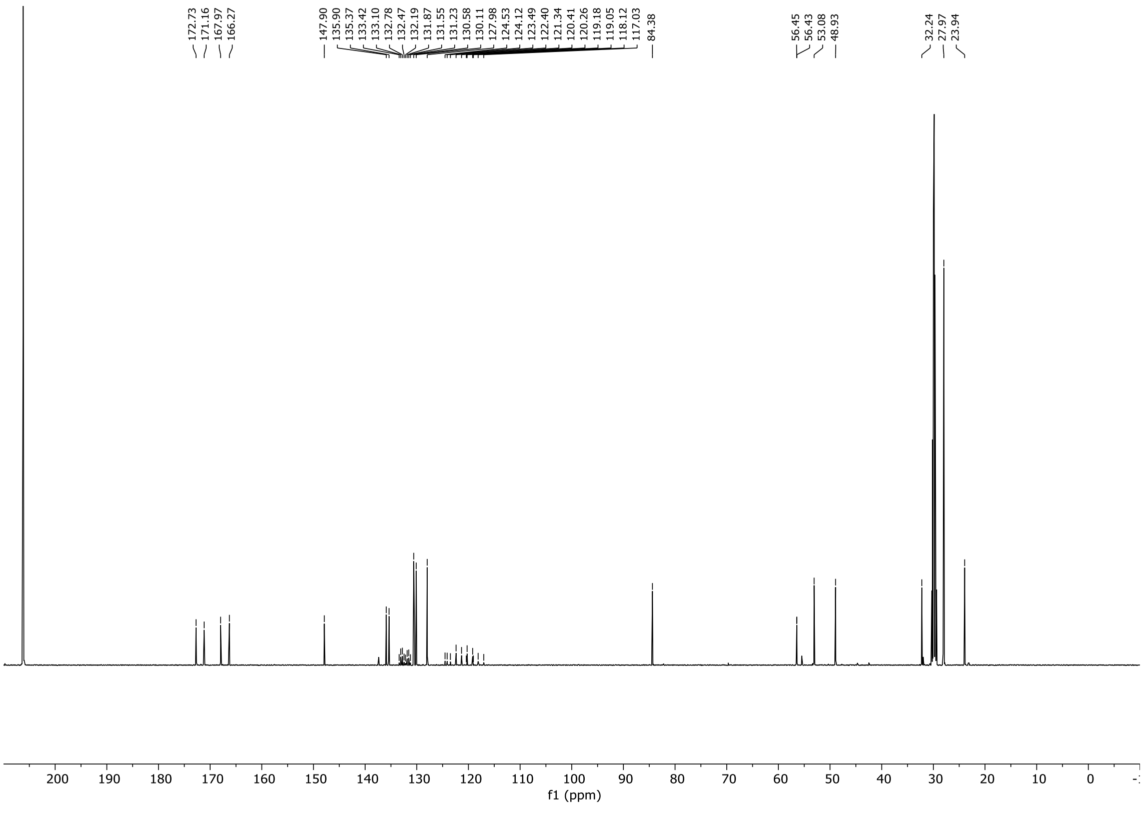

^{19}F NMR (471 MHz, $(CD_3)_2CO$) of compound **$(CF_3)_2$-arylazopyrazole photoswitch**:

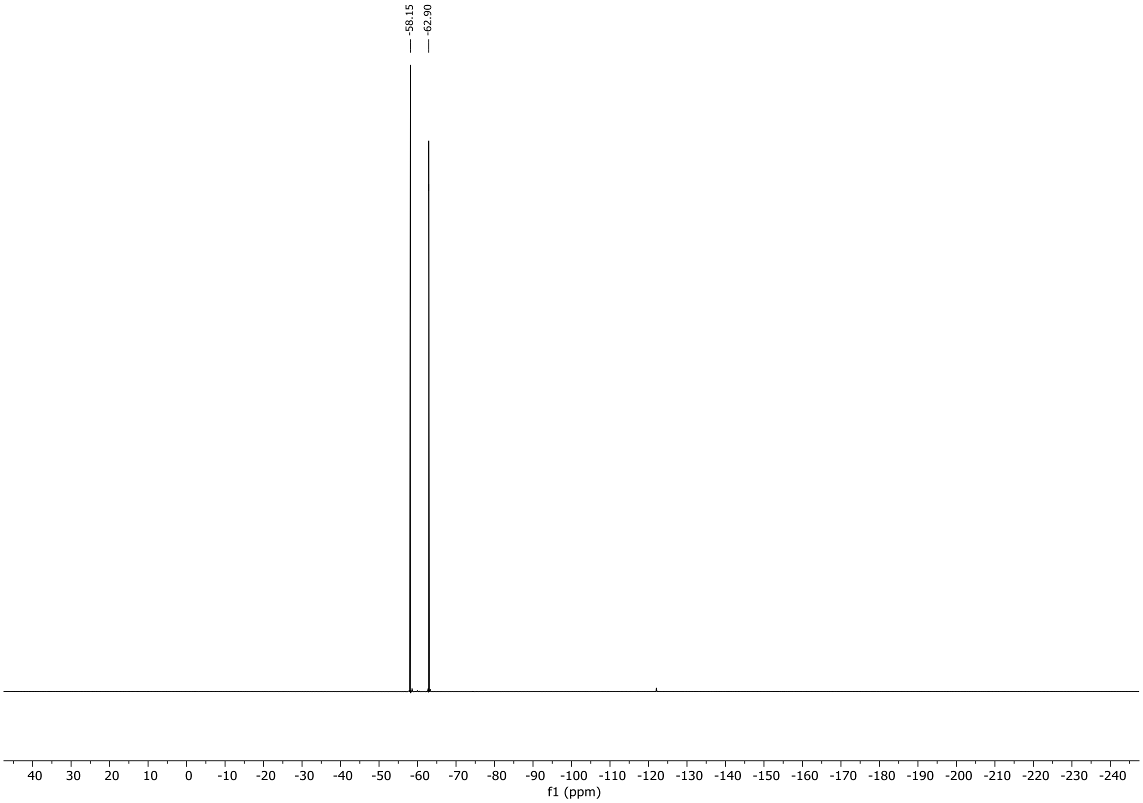

HSQC (500/126 MHz, $(CD_3)_2CO$) of compound **$(CF_3)_2$-arylazopyrazole photoswitch**:

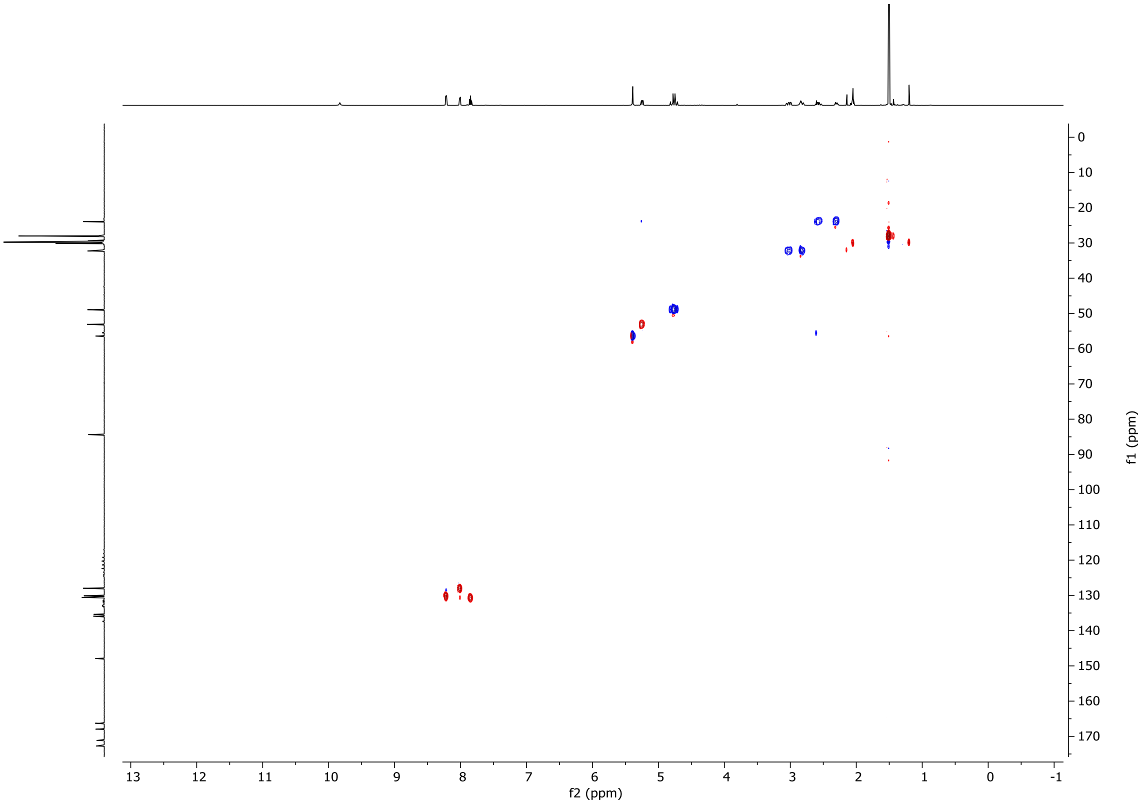

HMBC (500/126 MHz, $(CD_3)_2CO$) of compound **$(CF_3)_2$-arylazopyrazole photoswitch**:

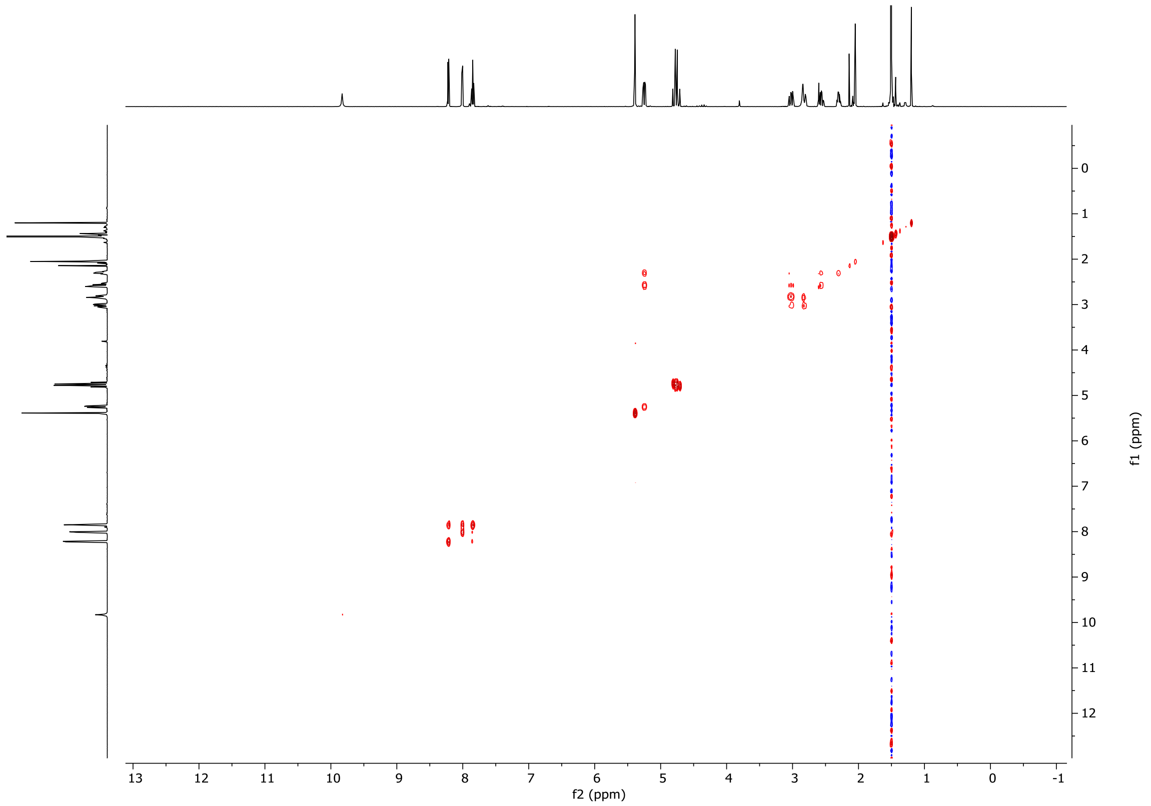

COSY (500 MHz, $(CD_3)_2CO$) of compound **$(CF_3)_2$-arylazopyrazole photoswitch**:

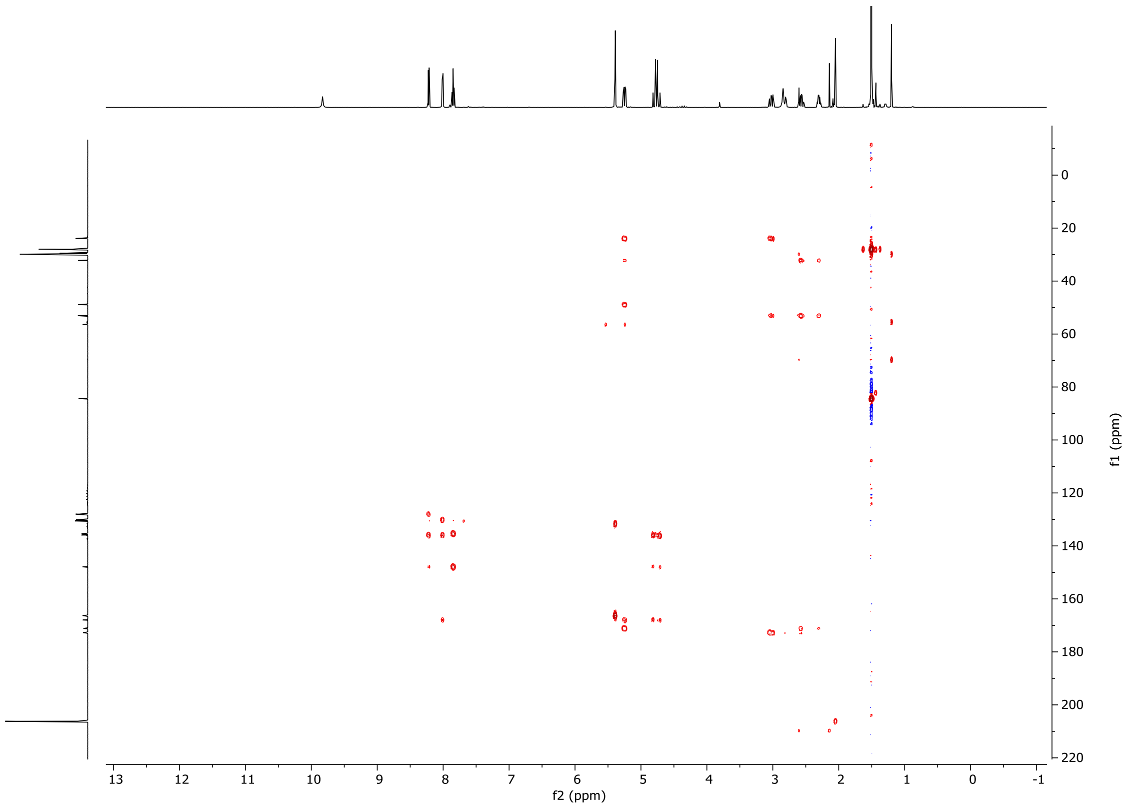

^{1}H NMR (500 MHz, $(CD_3)_2CO$) of compound **63**:

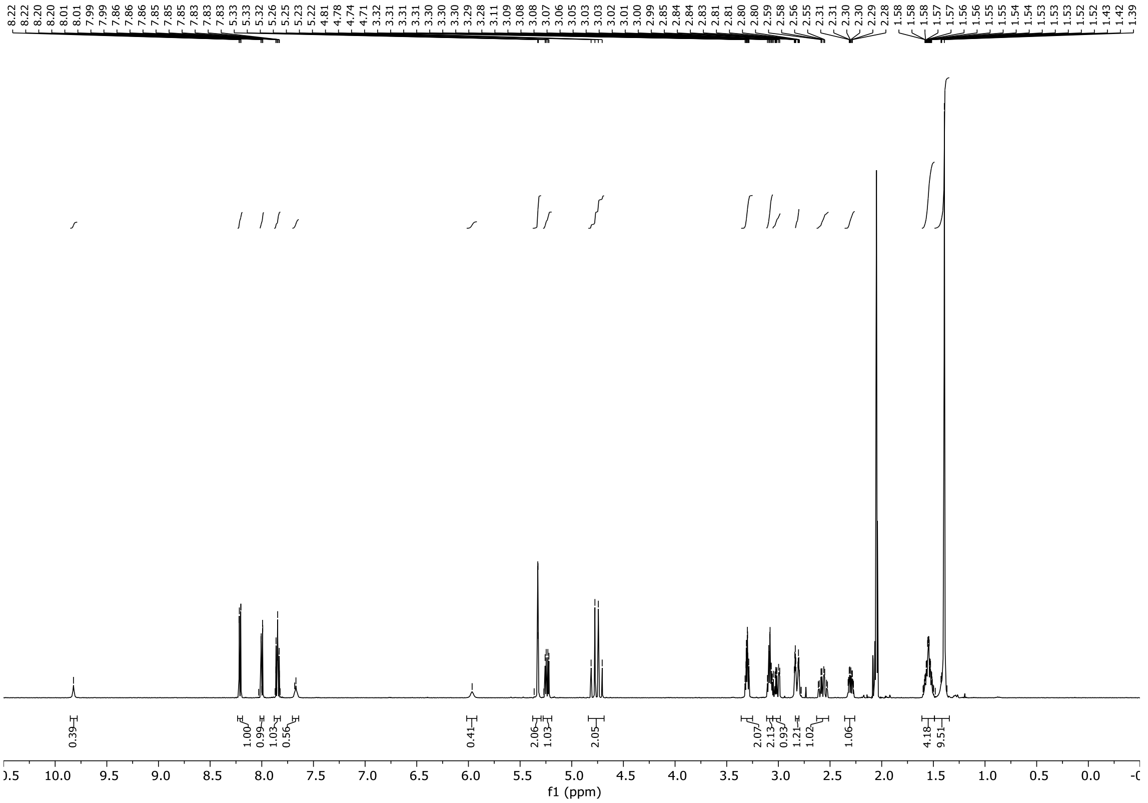

^{13}C NMR (126 MHz, $(CD_3)_2CO$) of compound **63**:

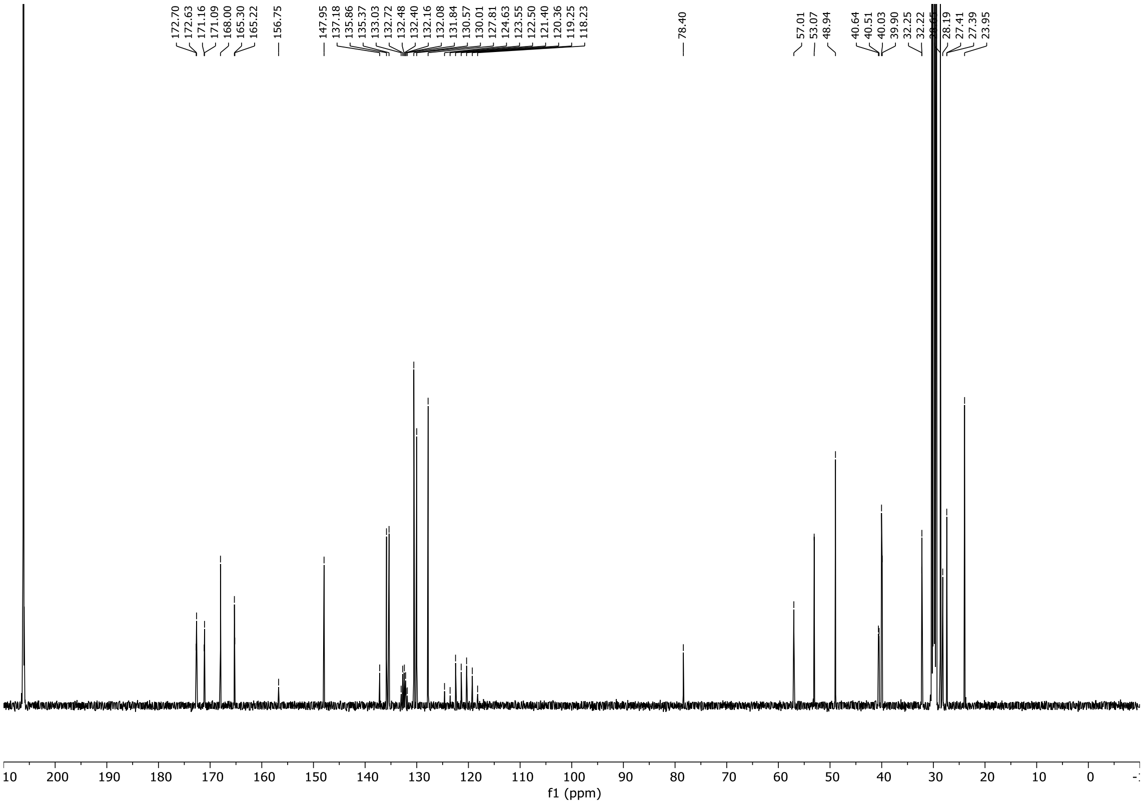

^{19}F NMR (471 MHz, $(CD_3)_2CO$) of compound **63**:

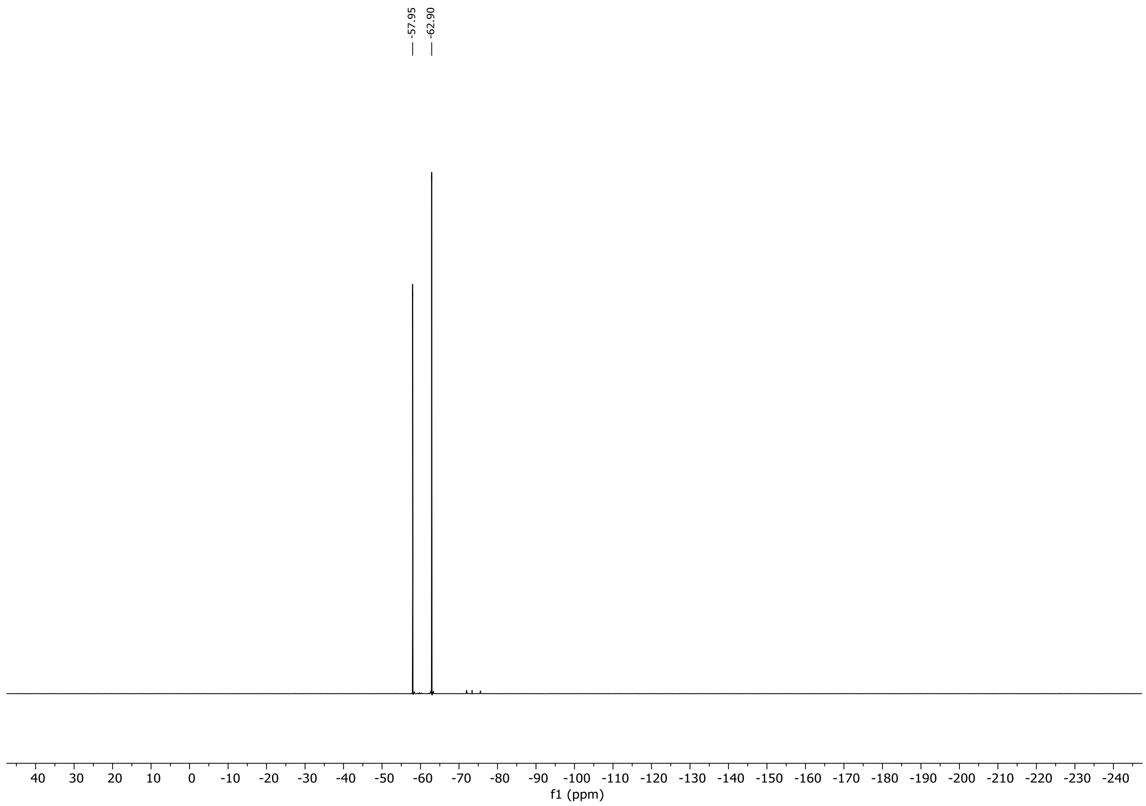

HSQC (400/101 MHz, $(CD_3)_2CO$) of compound **63**:

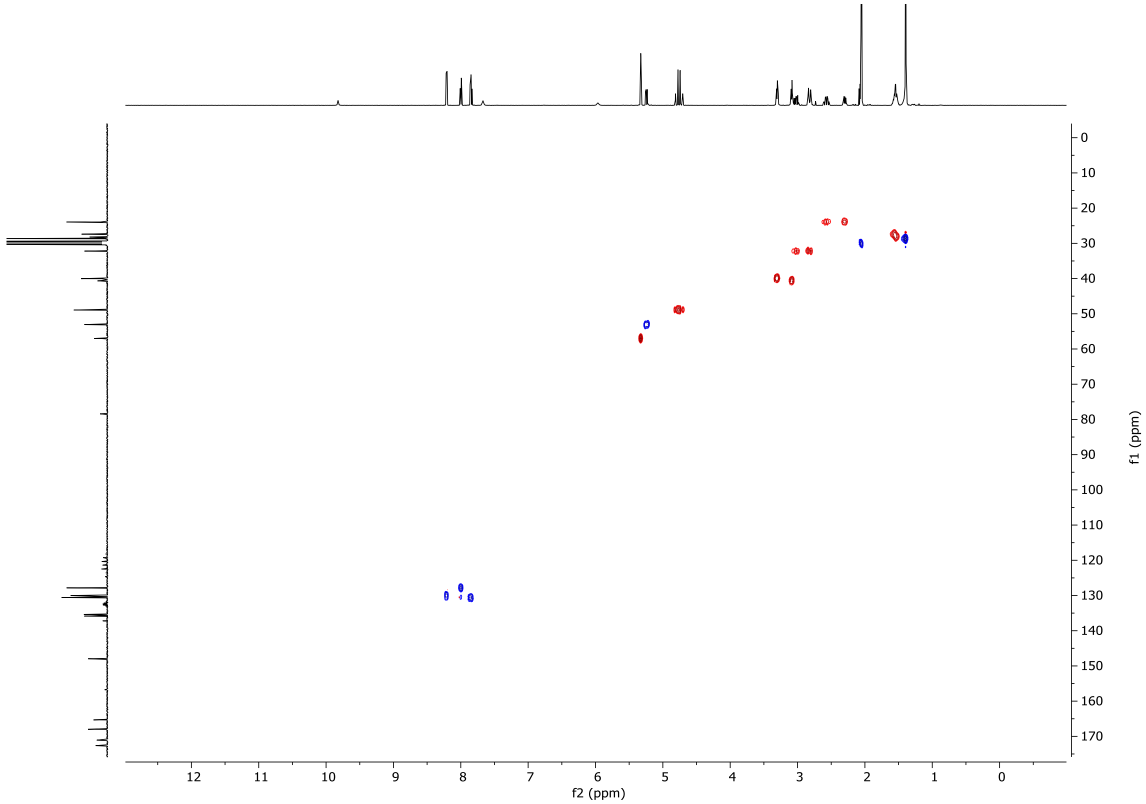

HMBC (400/101 MHz, $(CD_3)_2CO$) of compound **63**:

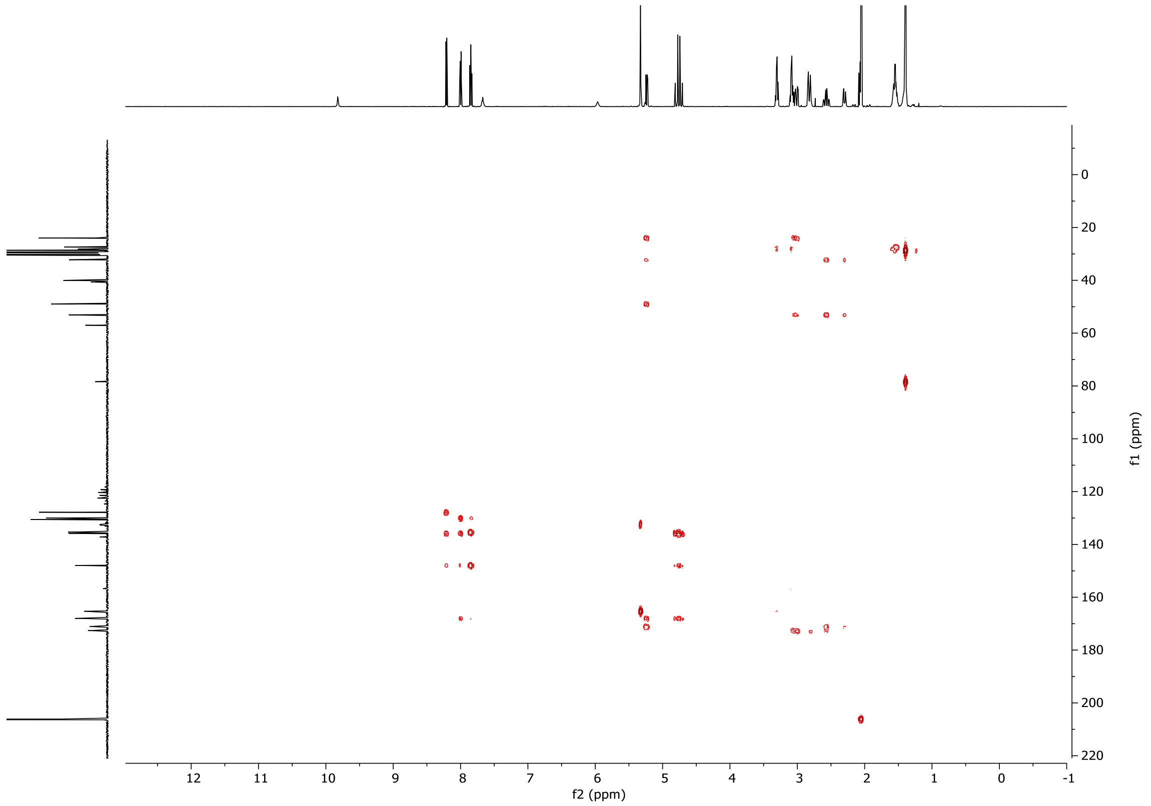

COSY (400 MHz, $(CD_3)_2CO$) of compound **63**:

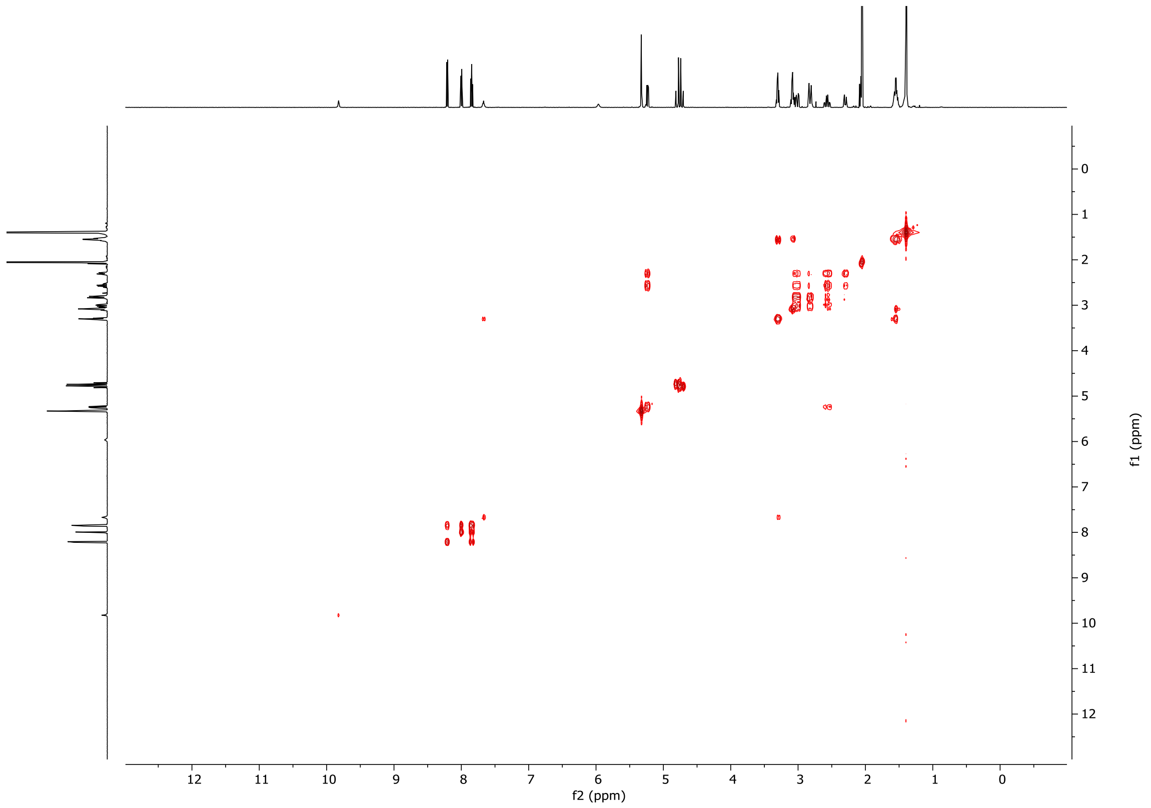

^{1}H NMR (500 MHz, $(CD_3)_2CO$) of compound **64**:

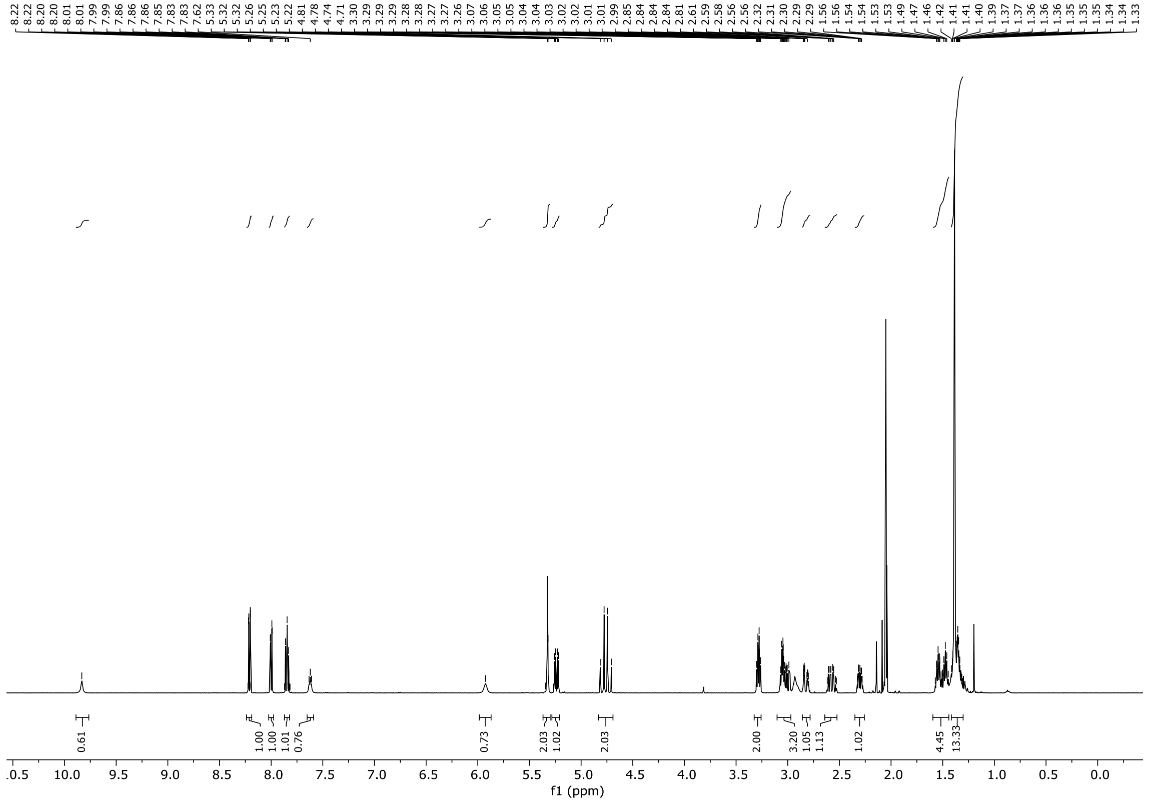

^{13}C NMR (126 MHz, $(CD_3)_2CO$) of compound **64**:

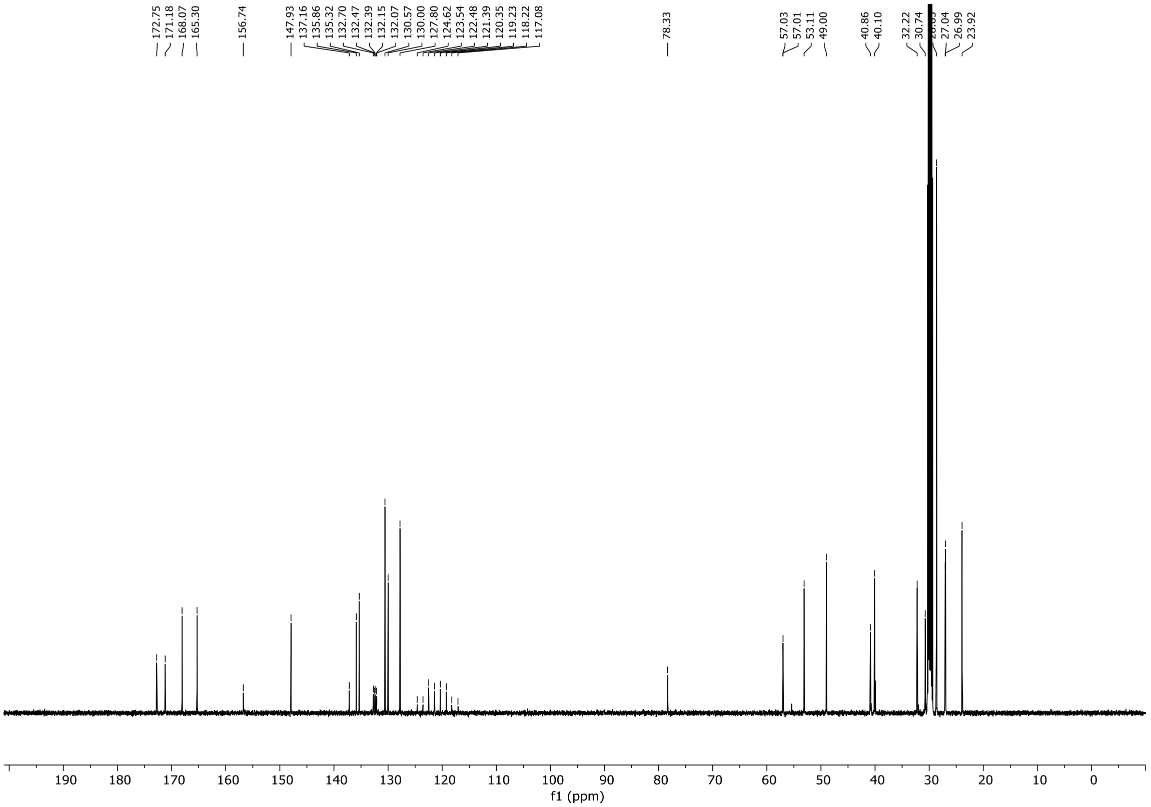

^{19}F NMR (471 MHz, $(CD_3)_2CO$) of compound **64**:

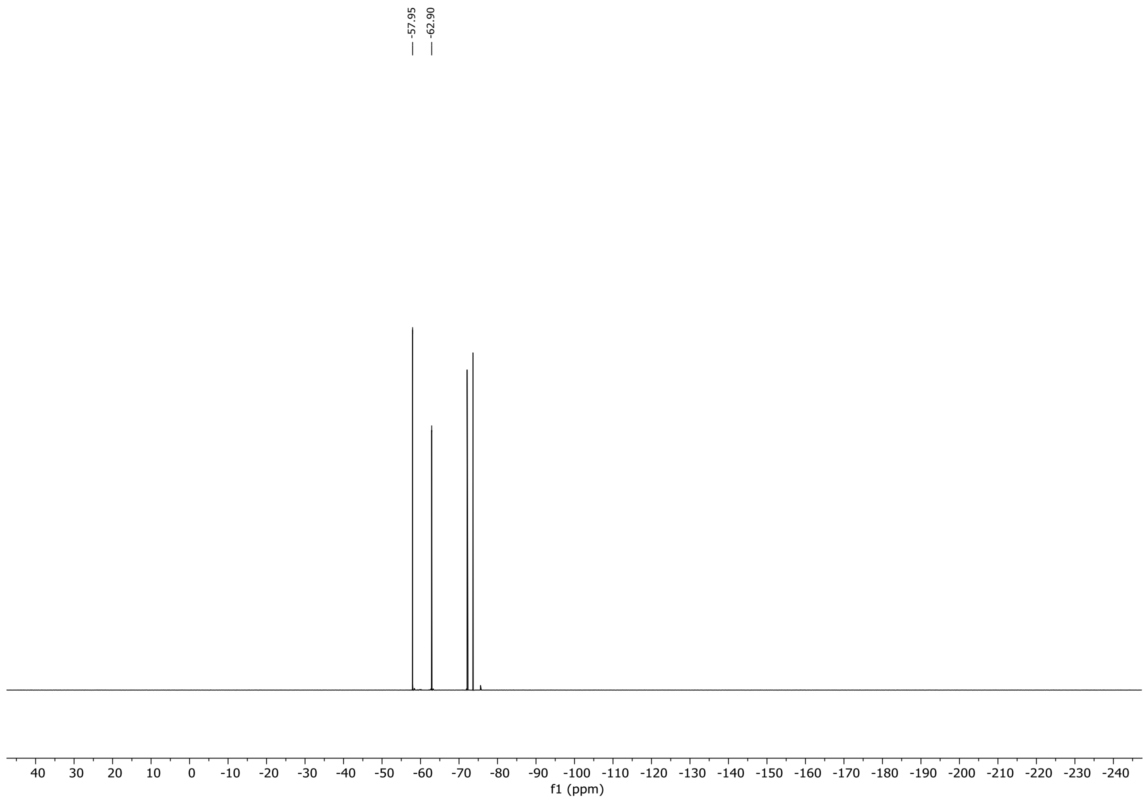

HSQC (400/101 MHz, $(CD_3)_2CO$) of compound **64**:

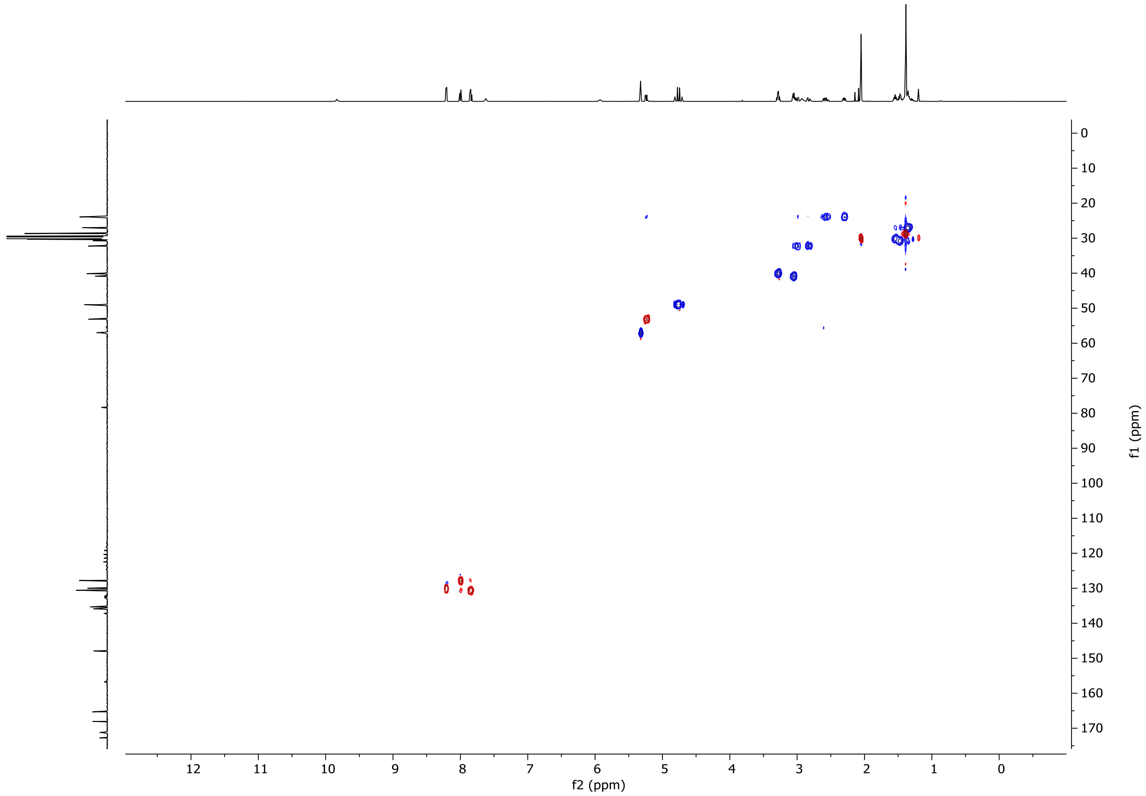

HMBC (400/101, $(CD_3)_2CO$) of compound **64**:

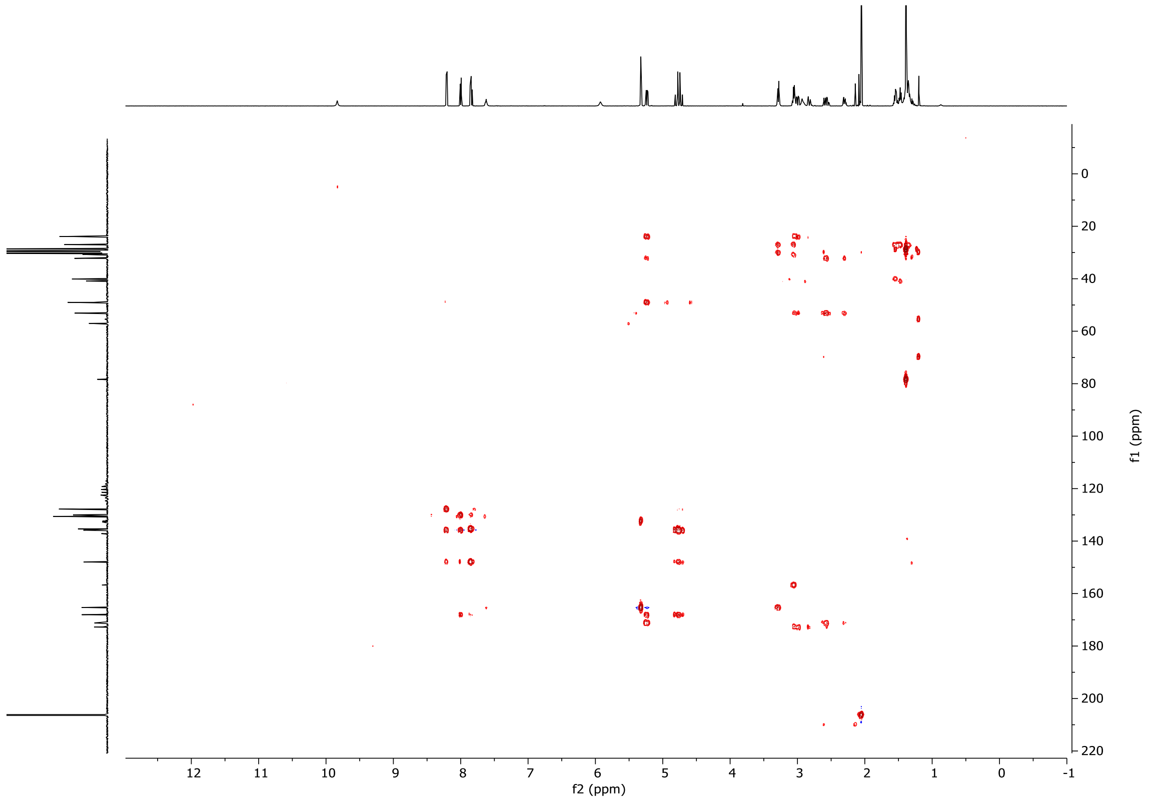

COSY (400 MHz, $(CD_3)_2CO$) of compound **64**:

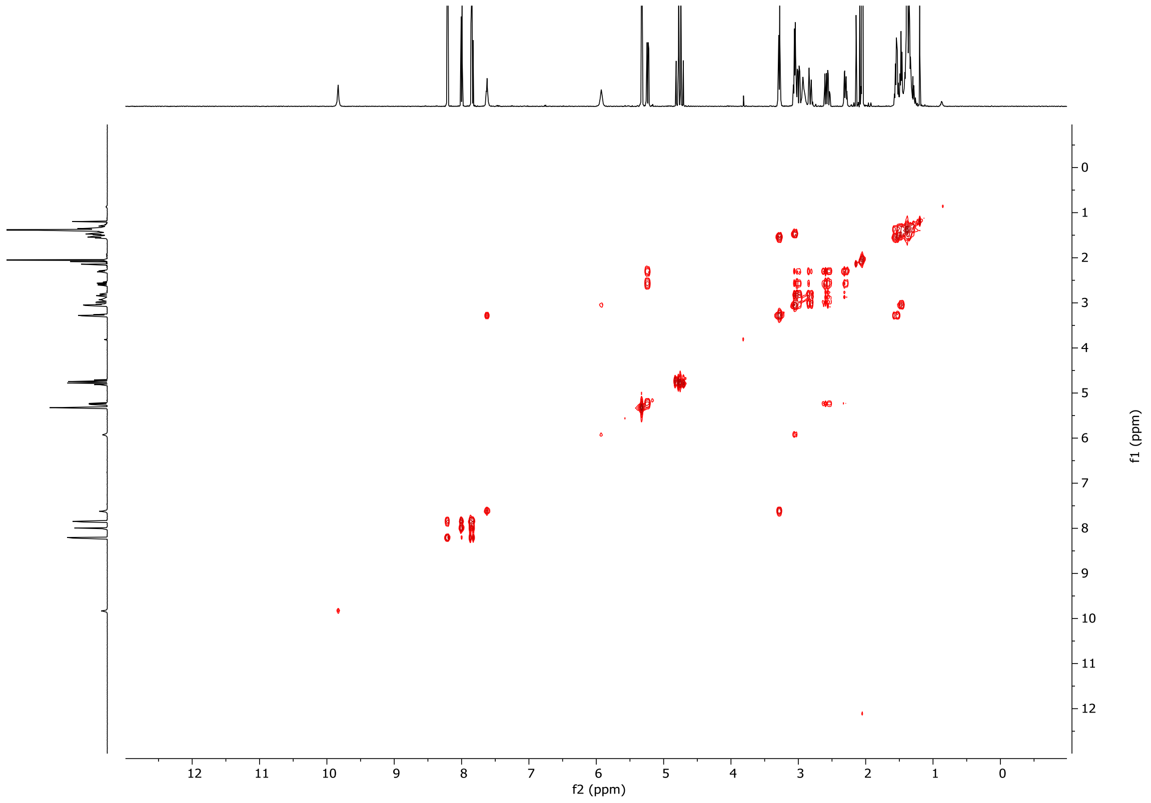

^{1}H NMR (500 MHz, $(CD_3)_2CO$) of compound **65**:

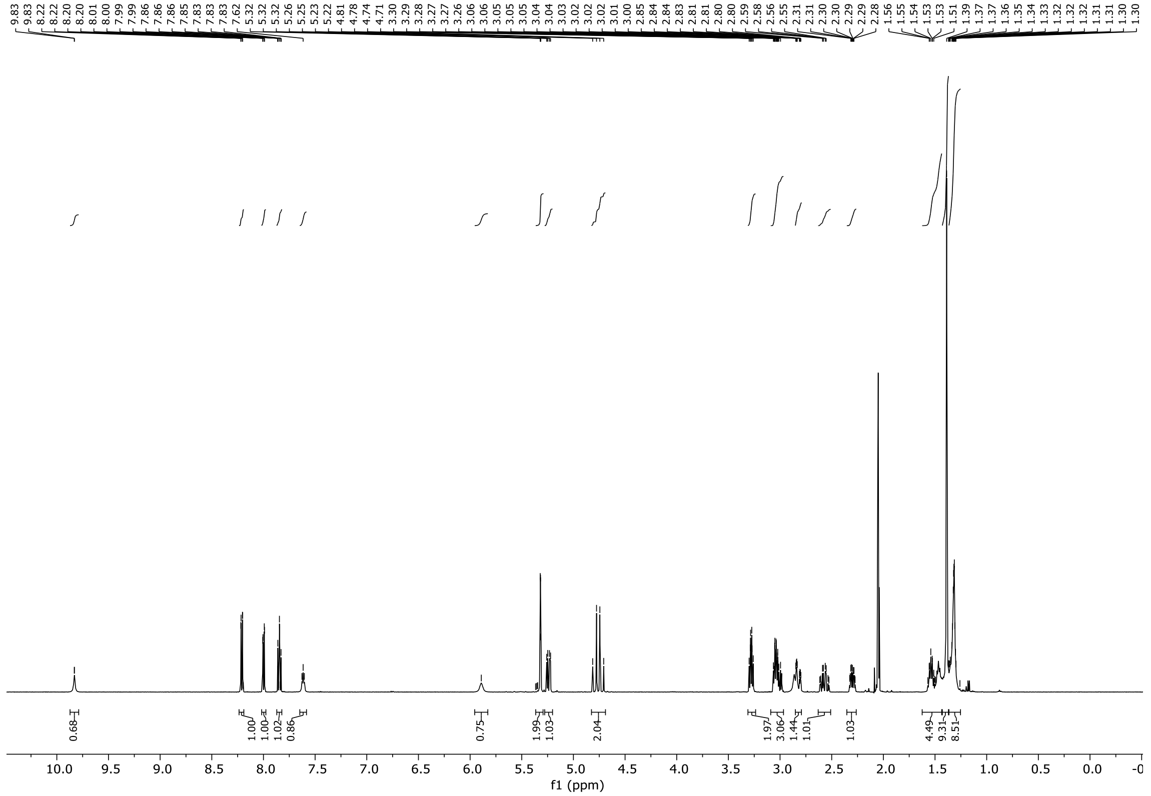

^{13}C NMR (126 MHz, $(CD_3)_2CO$) of compound **65**:

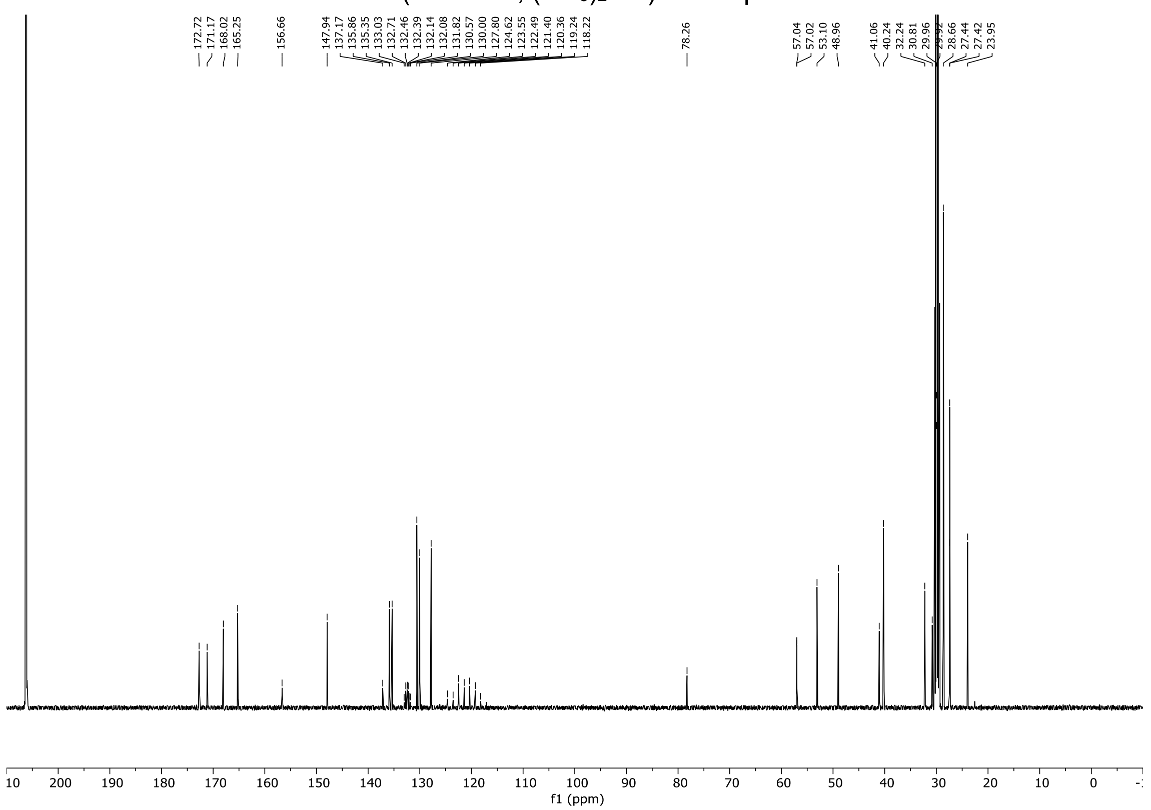

^{19}F NMR (471 MHz, $(CD_3)_2CO$) of compound **65**:

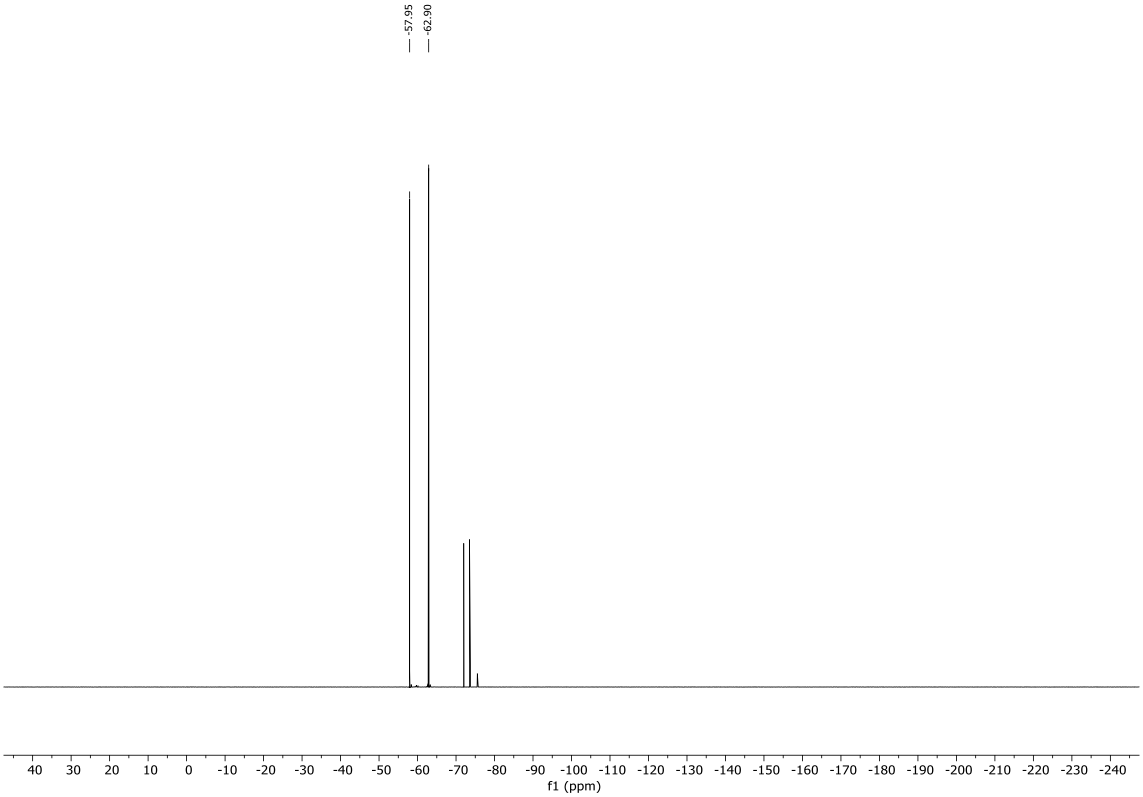

HSQC (400/101 MHz, $(CD_3)_2CO$) of compound **65**:

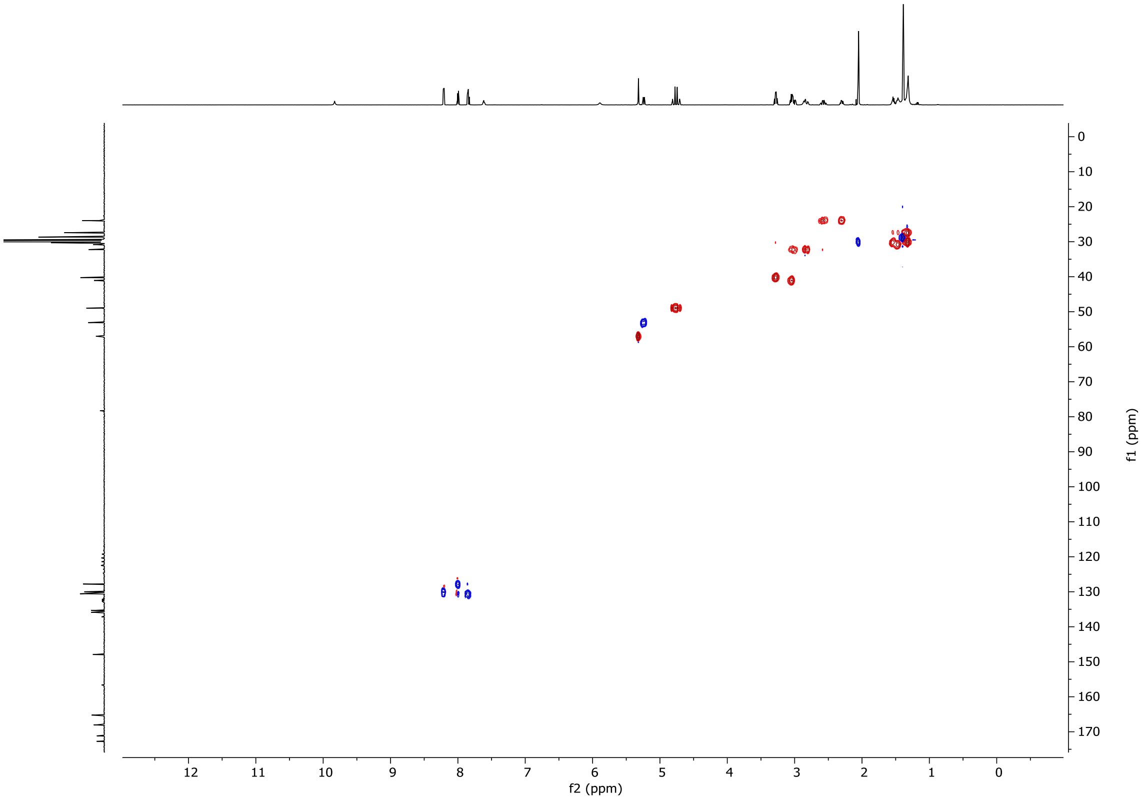

HMBC (400/101 MHz, $(CD_3)_2CO$) of compound **65**:

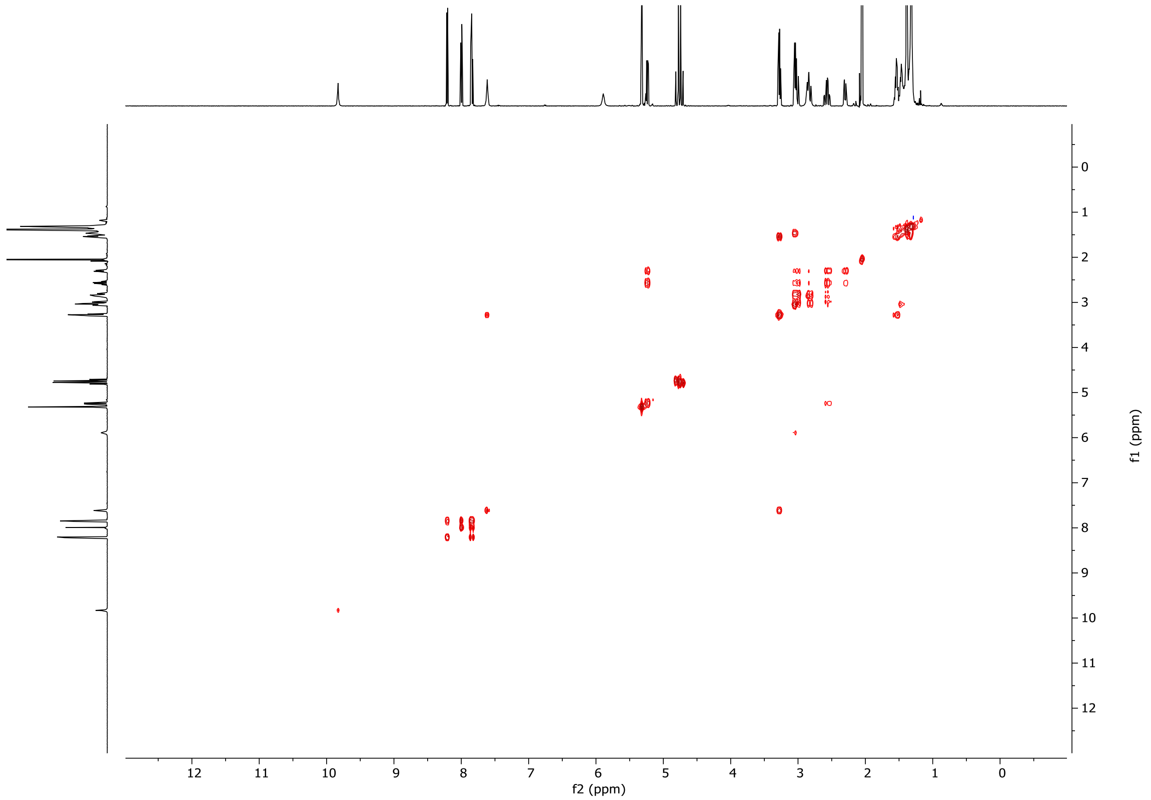

COSY (400 MHz, $(CD_3)_2CO$) of compound **65**:

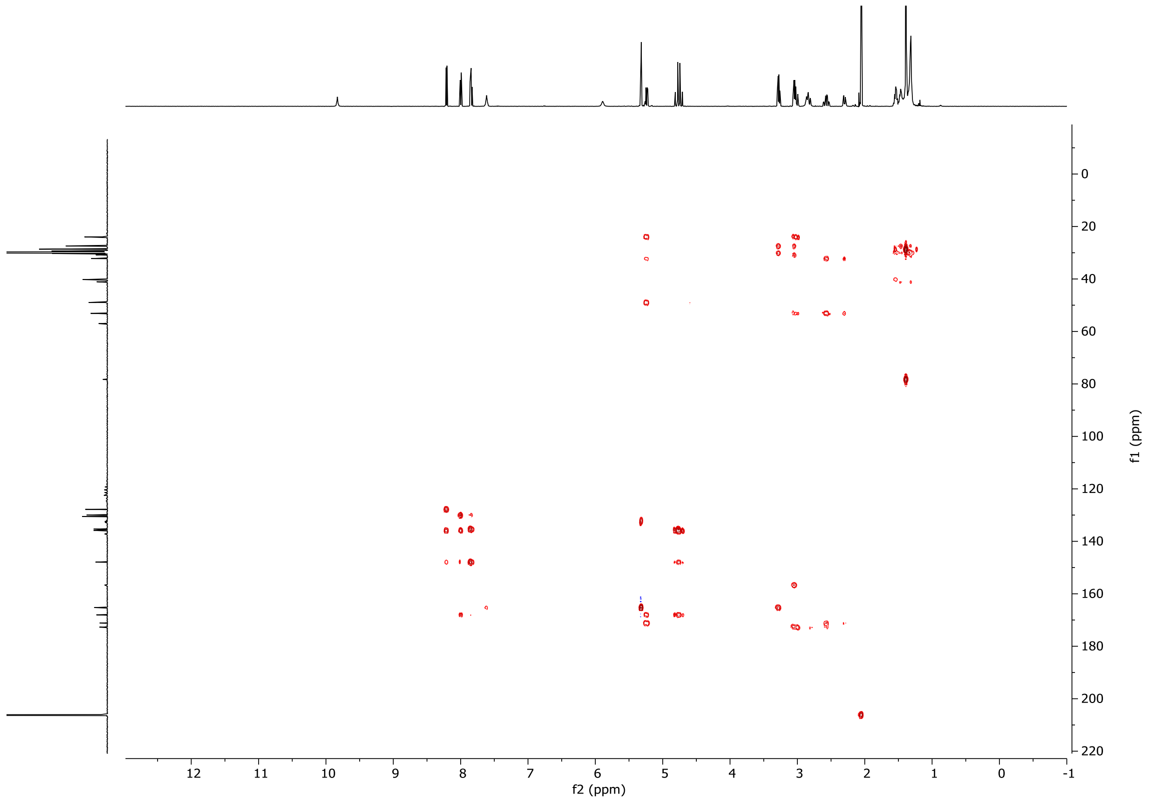

^{1}H NMR (500 MHz, $(CD_3)_2CO$) of compound **66**:

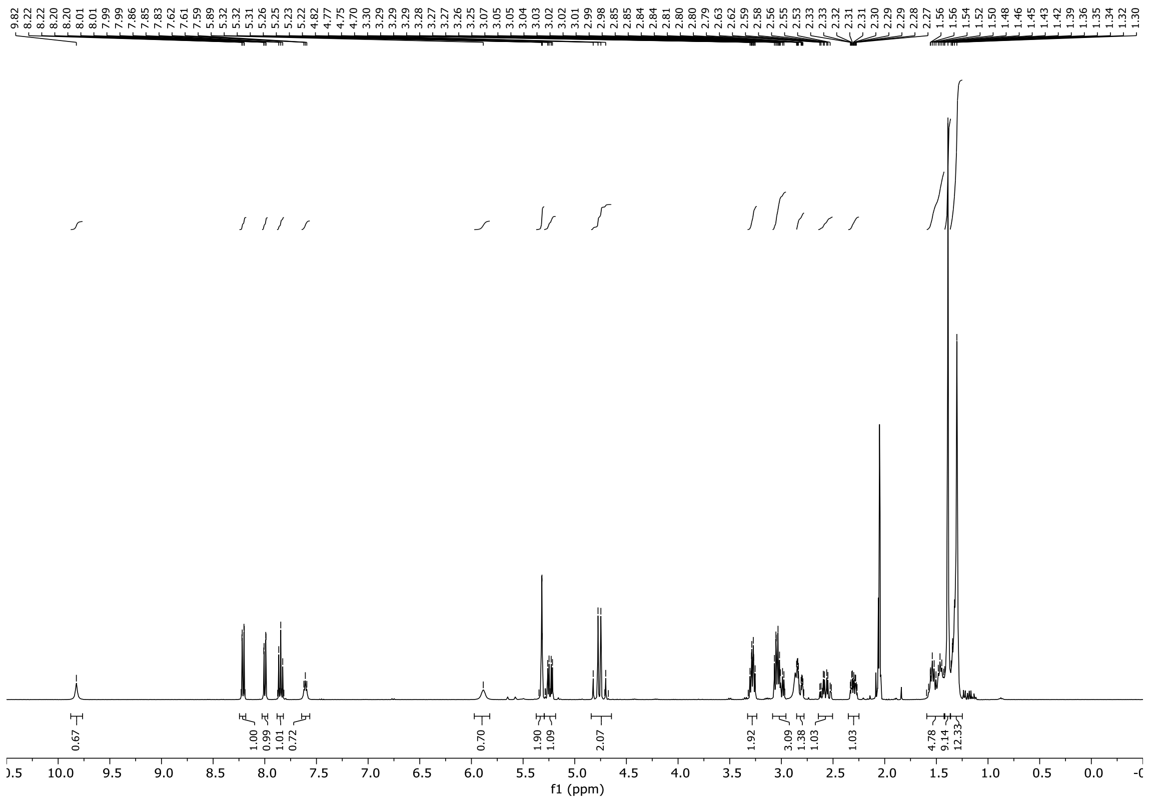

^{13}C NMR (126 MHz, $(CD_3)_2CO$) of compound **66**:

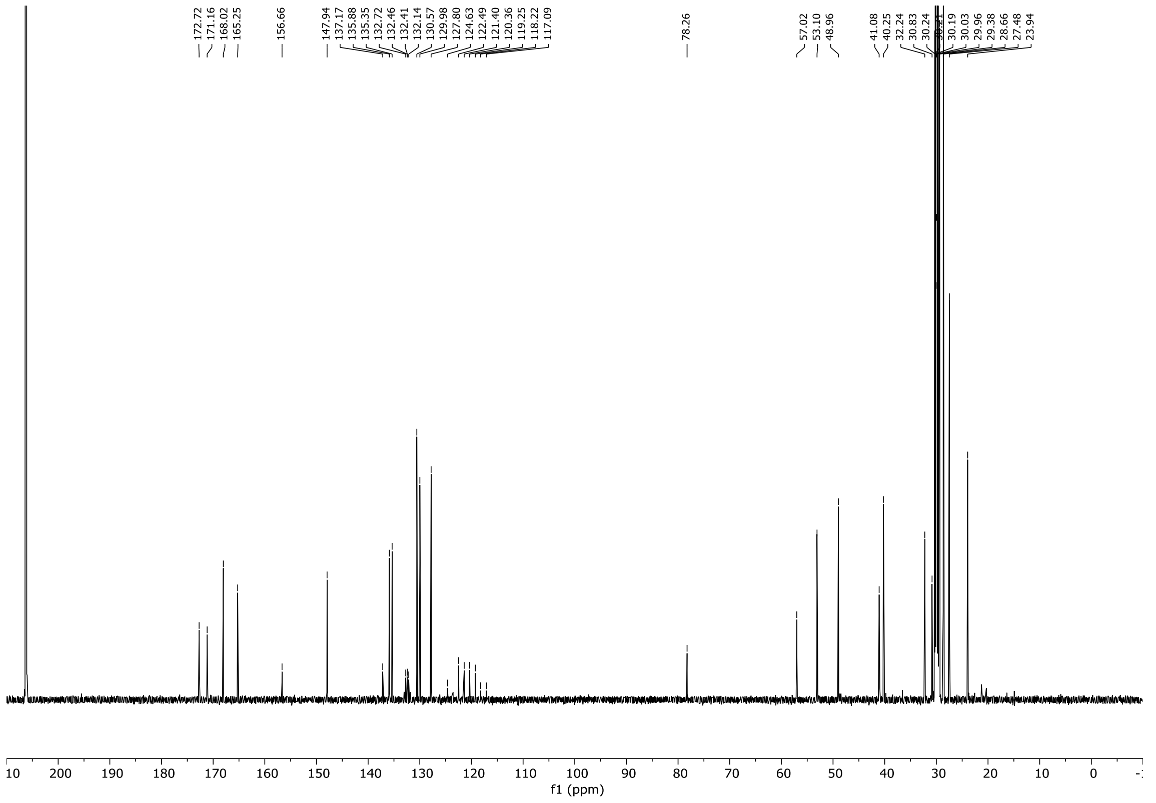

^{19}F NMR (471 MHz, $(CD_3)_2CO$) of compound **66**:

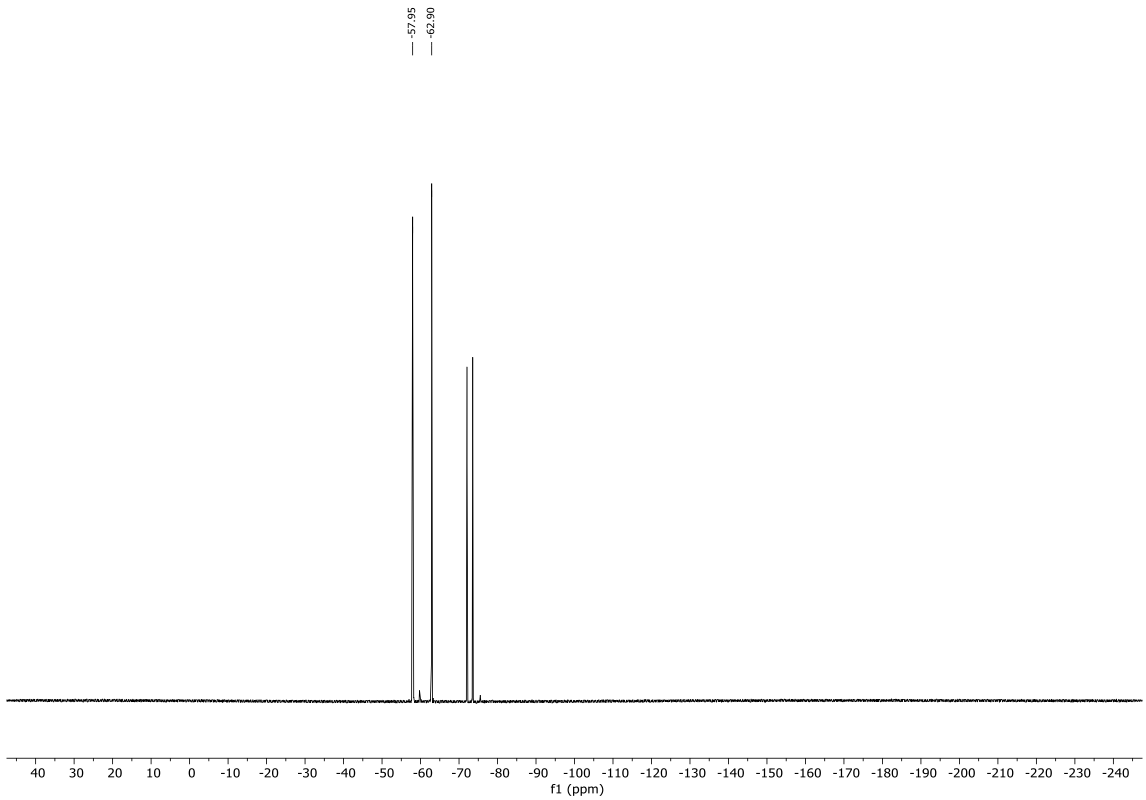

HSQC (400/101 MHz, $(CD_3)_2CO$) of compound **66**:

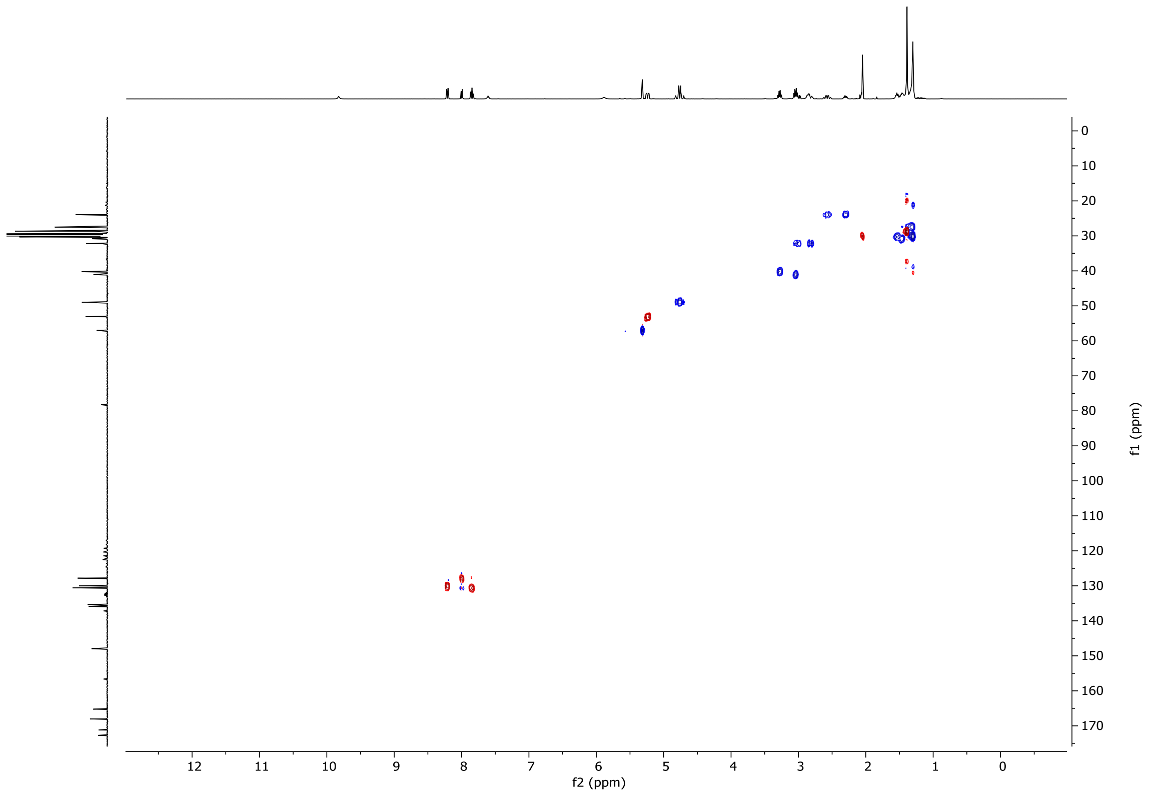

HMBC (400/101 MHz, $(CD_3)_2CO$) of compound **66**:

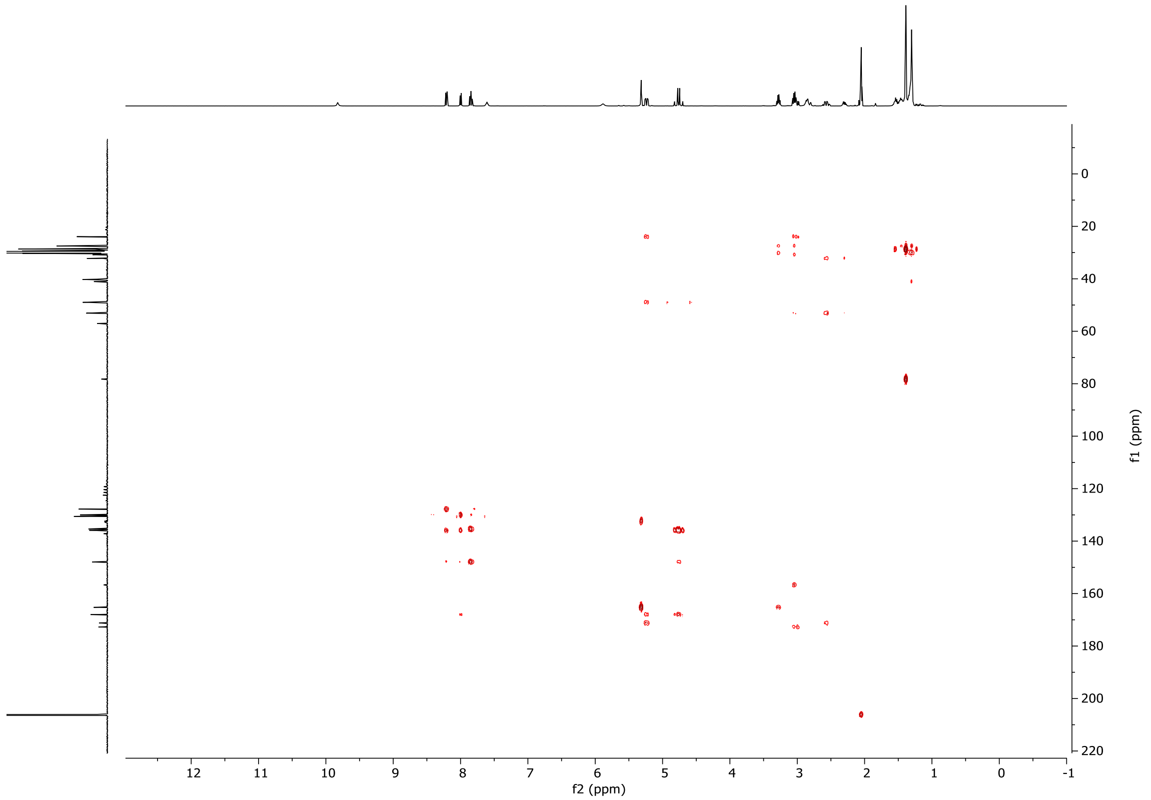

COSY (400 MHz, $(CD_3)_2CO$) of compound **66**:

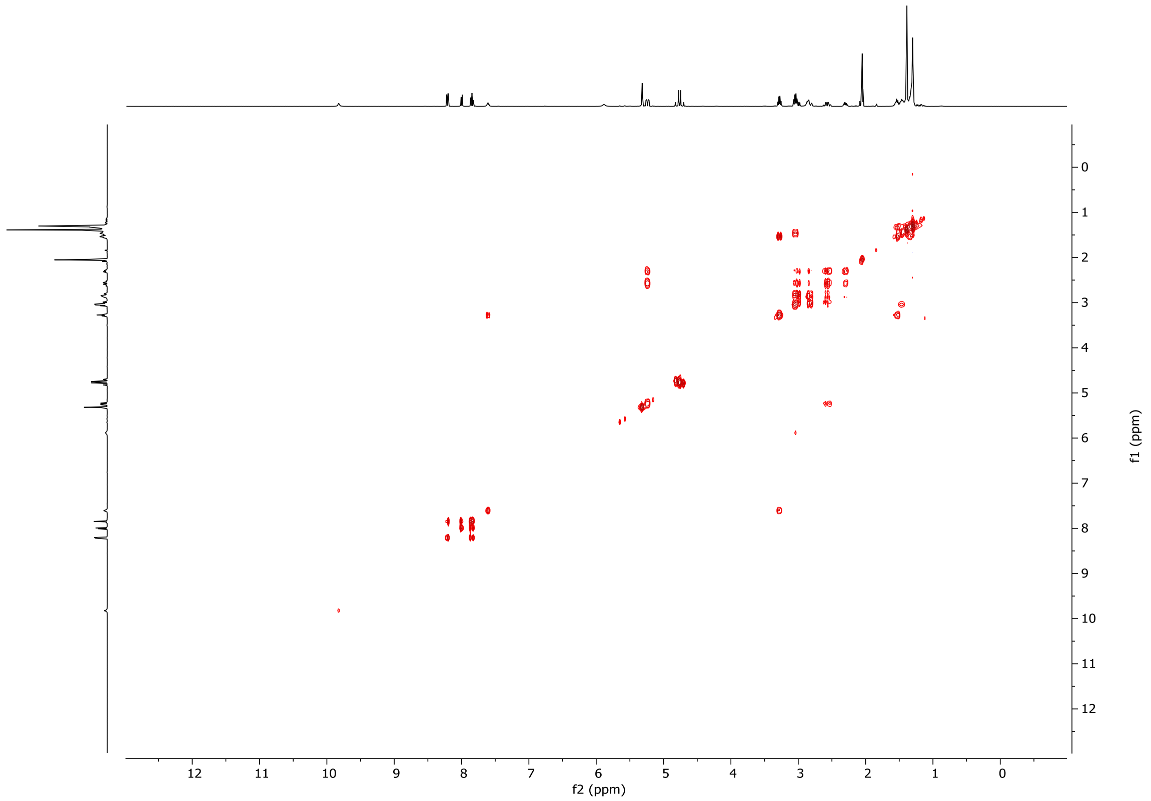

^{1}H NMR (500 MHz, $(CD_3)_2SO$) of compound **KH-5-327**:

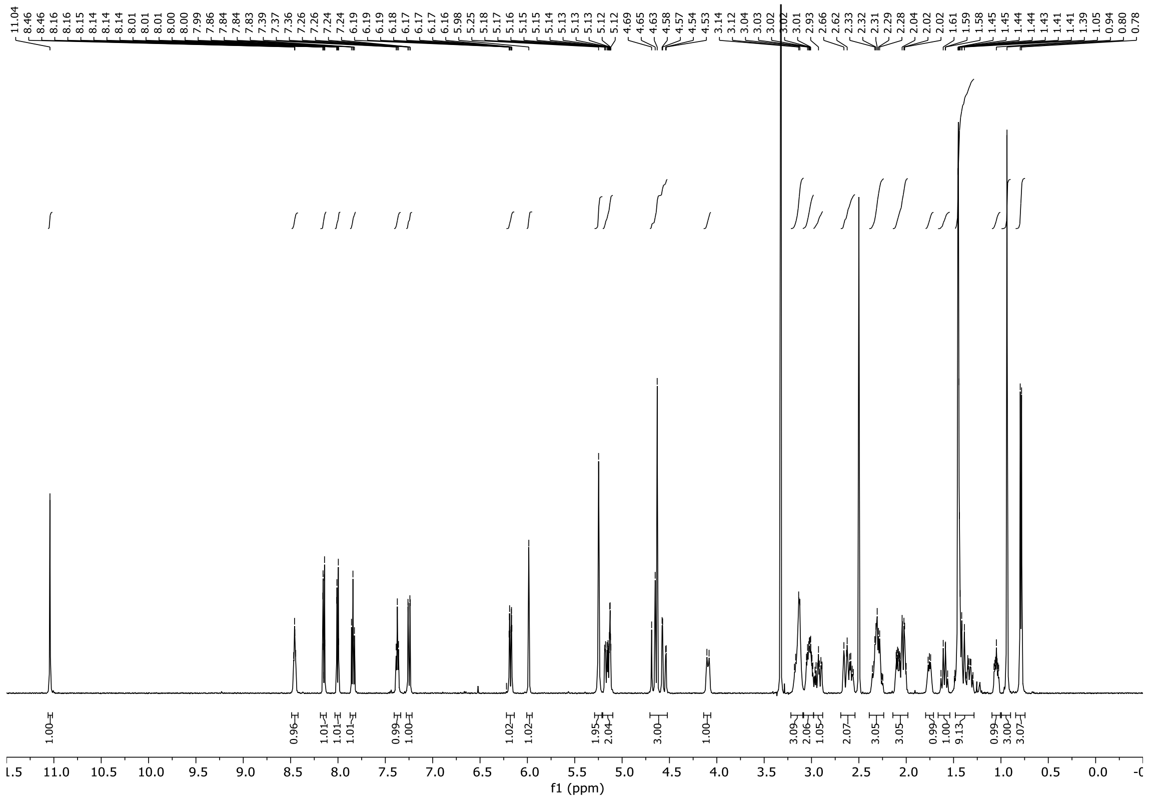

^{13}C NMR (126 MHz, $(CD_3)_2SO$) of compound **KH-5-327**:

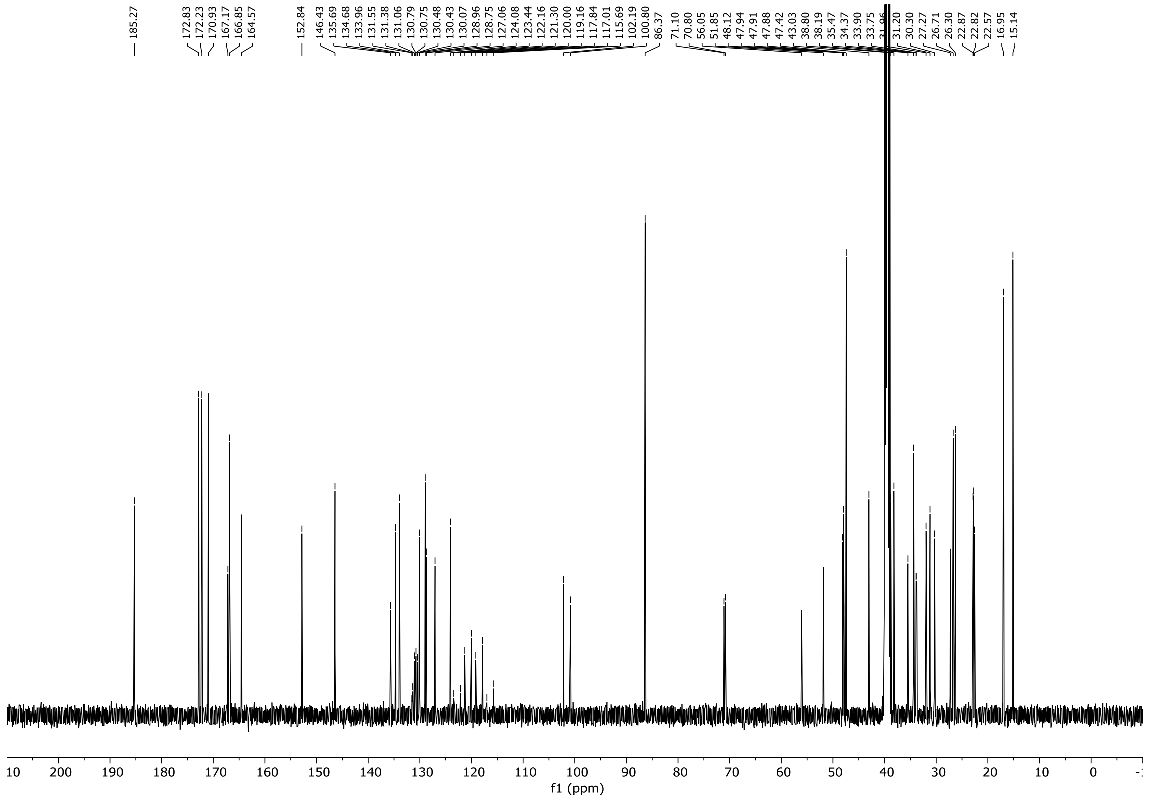

^{19}F NMR (377 MHz, $(CD_3)_2SO$) of compound **KH-5-327**:

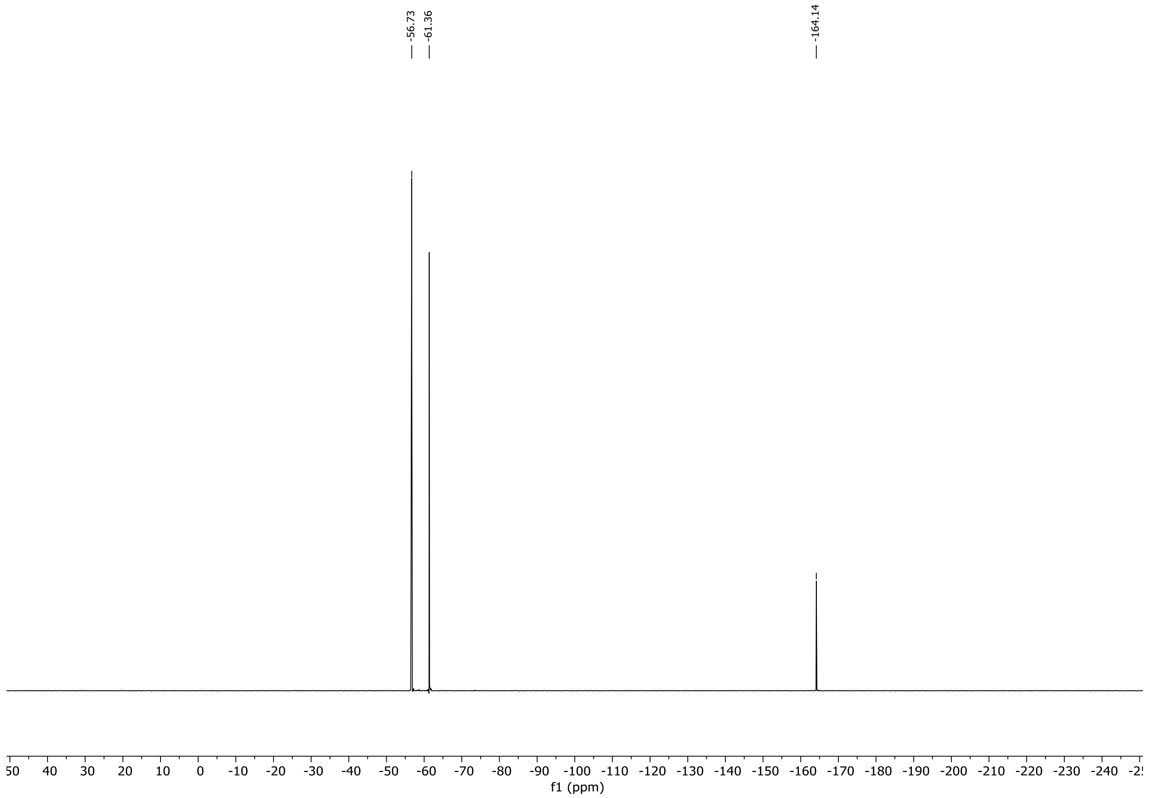

HSQC (400/101 MHz, $(CD_3)_2SO$) of compound **KH-5-327**:

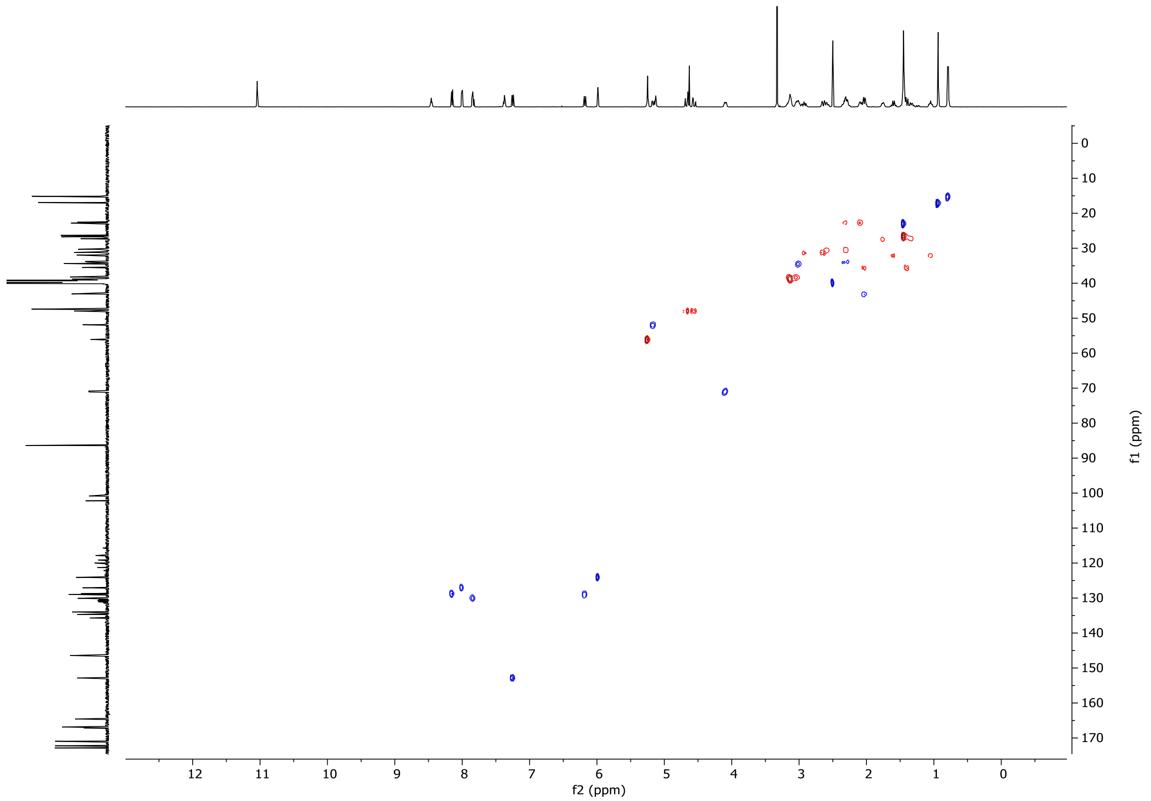

HMBC (400/101 MHz, $(CD_3)_2SO$) of compound **KH-5-327**:

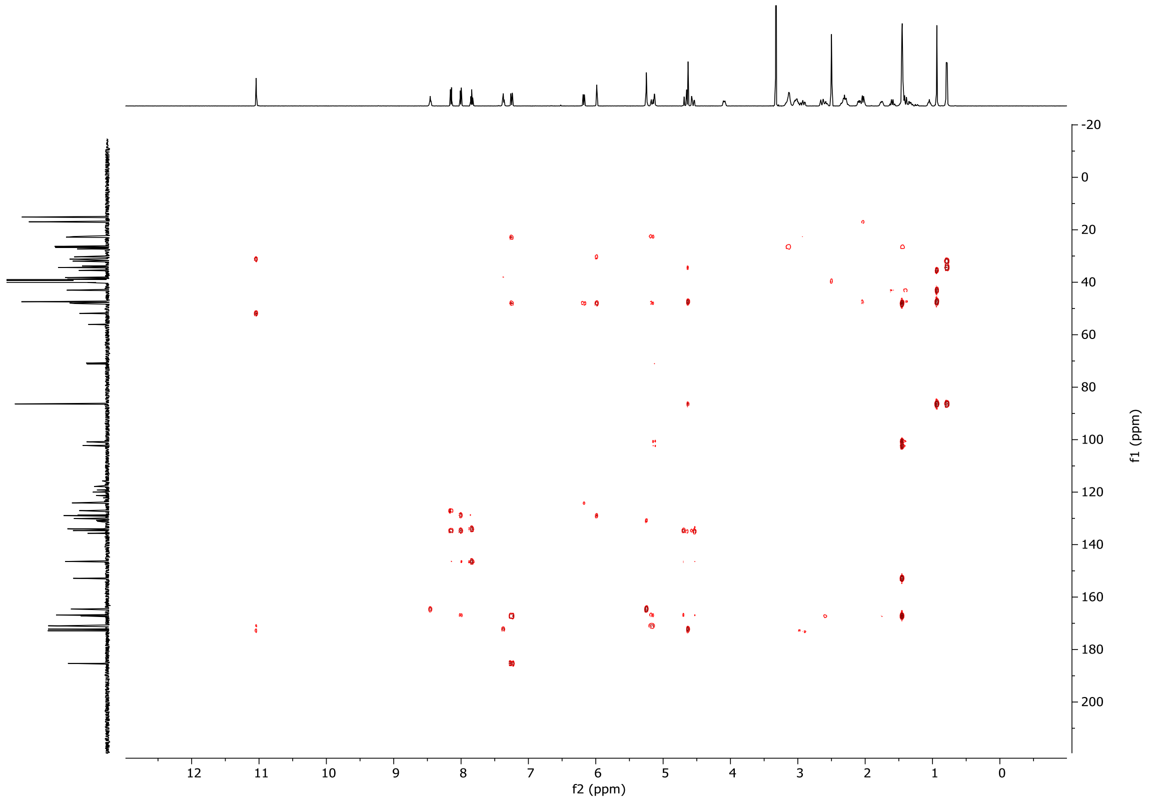

COSY (400 MHz, $(CD_3)_2SO$) of compound **KH-5-327**:

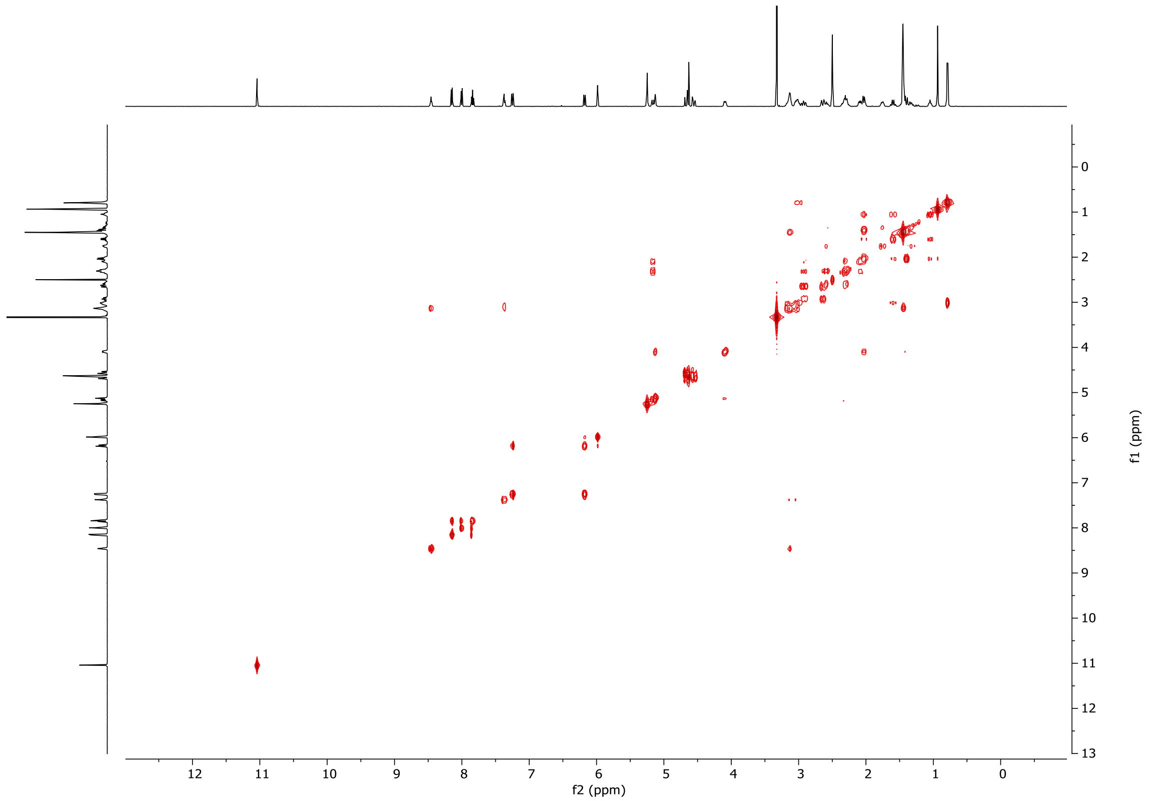

^{1}H NMR (500 MHz, $(CD_3)_2SO$) of compound **KH-5-331**:

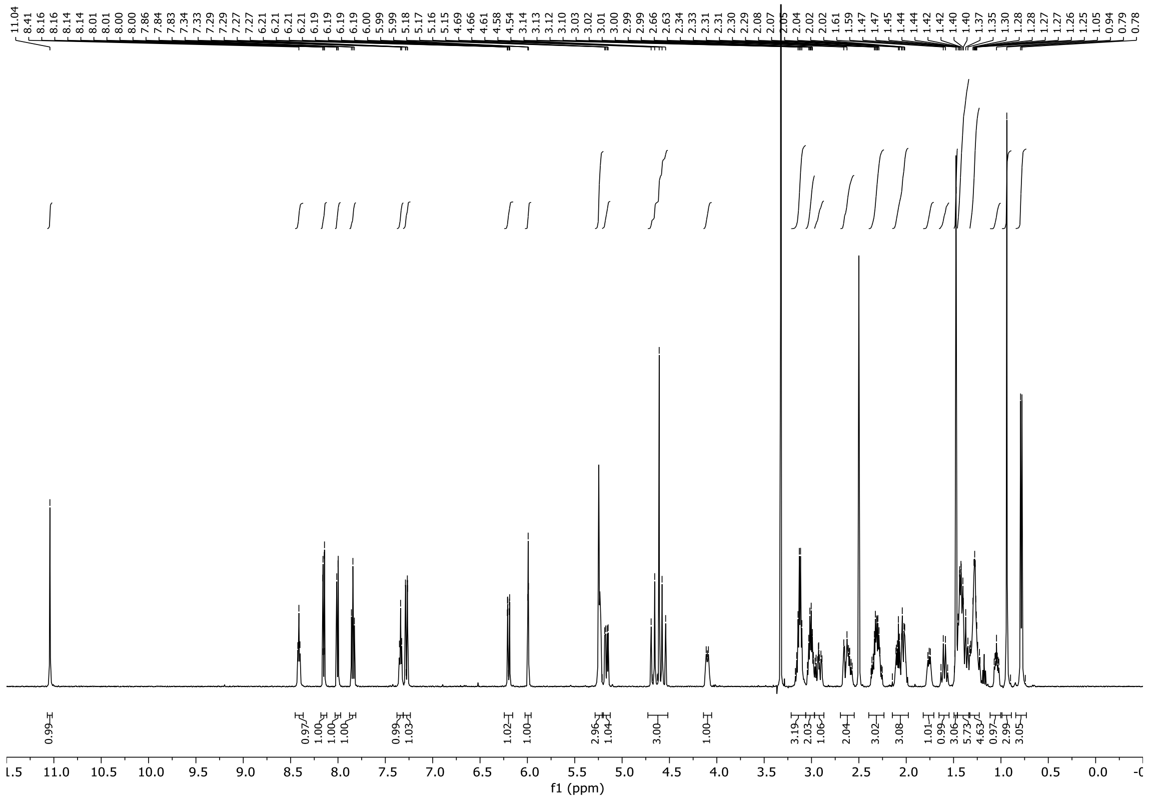

^{13}C NMR (126 MHz, $(CD_3)_2SO$) of compound **KH-5-331**:

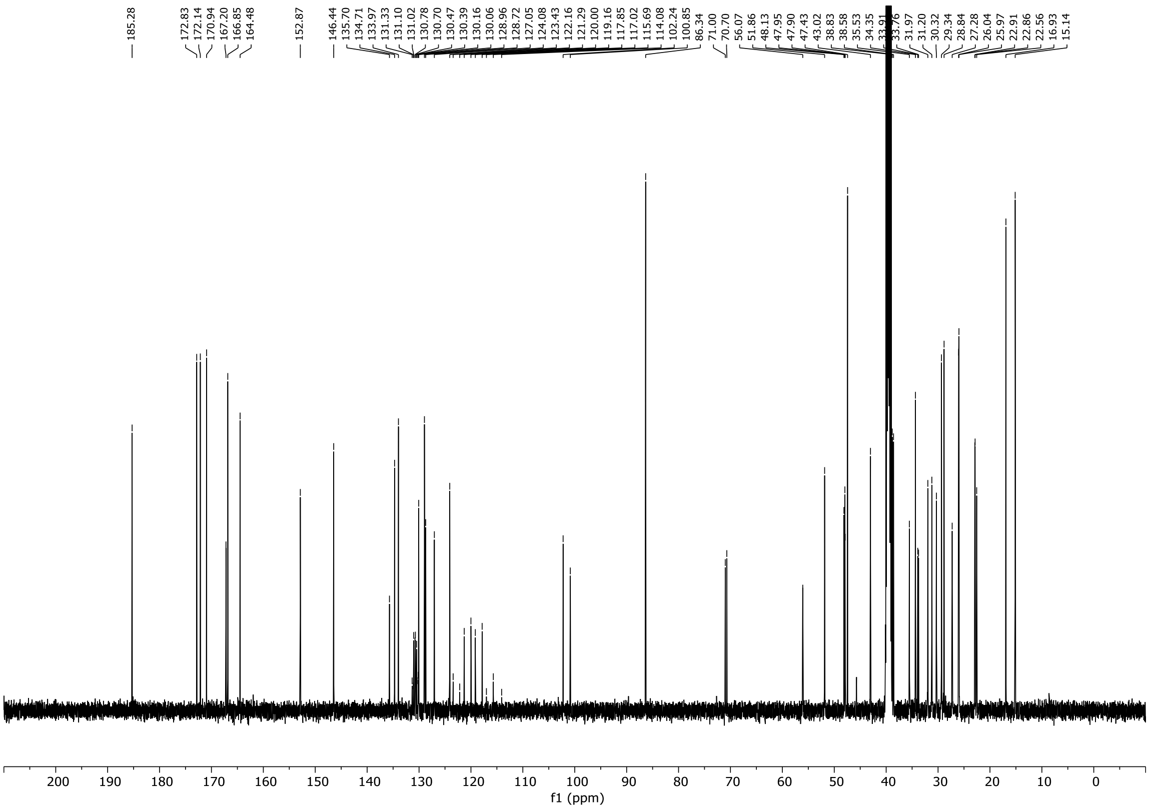

^{19}F NMR (471 MHz, $(CD_3)_2SO$) of compound **KH-5-331**:

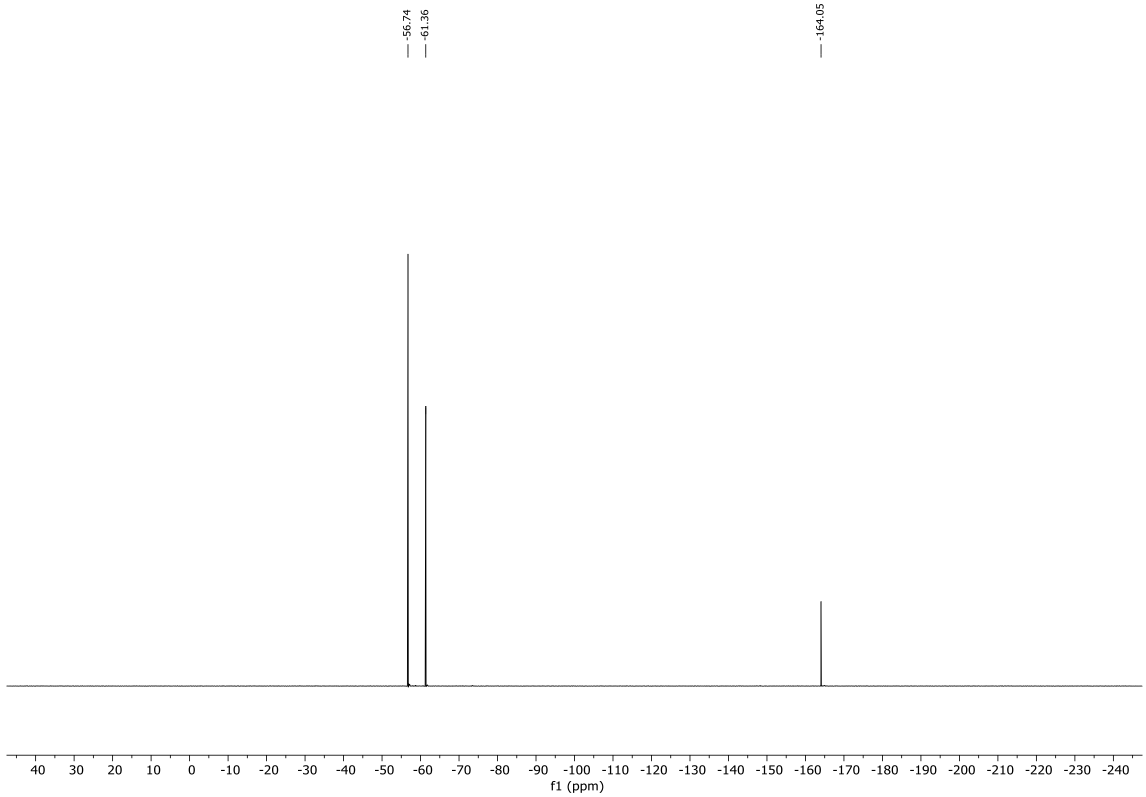

HSQC (400/101 MHz, $(CD_3)_2SO$) of compound **KH-5-331**:

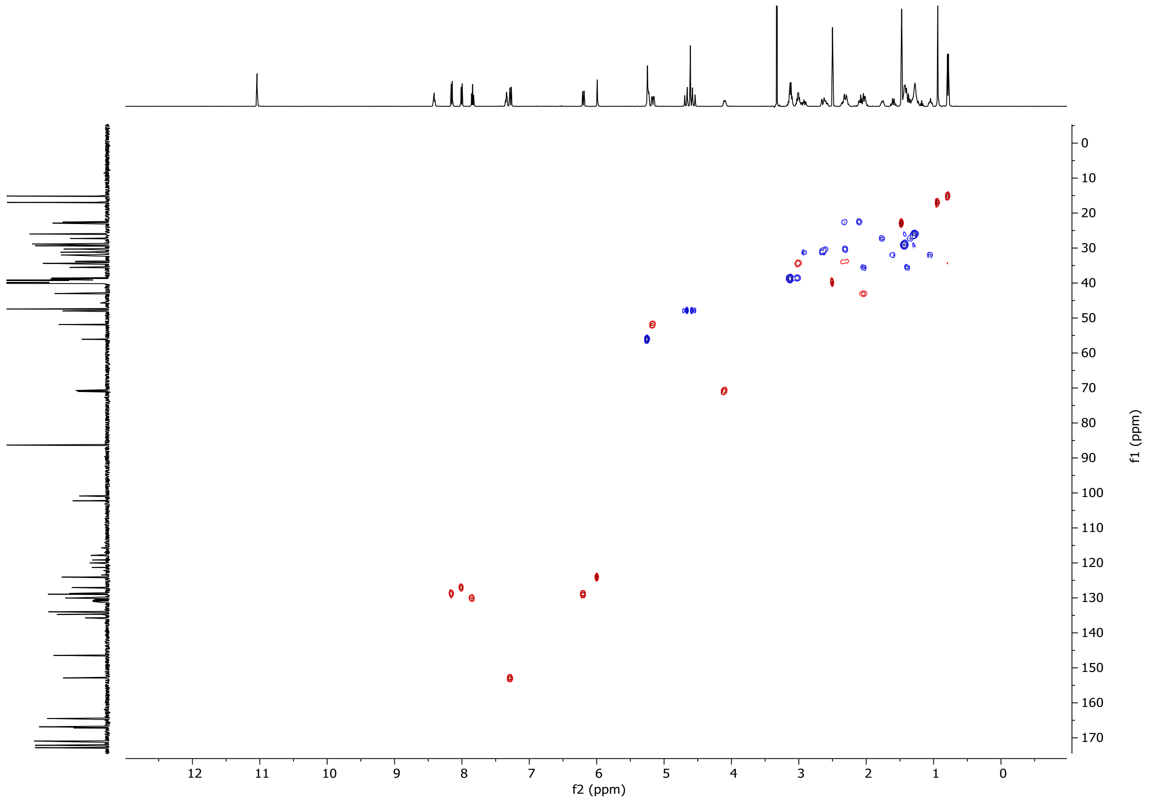

HMBC (400/101 MHz, $(CD_3)_2SO$) of compound **KH-5-331**:

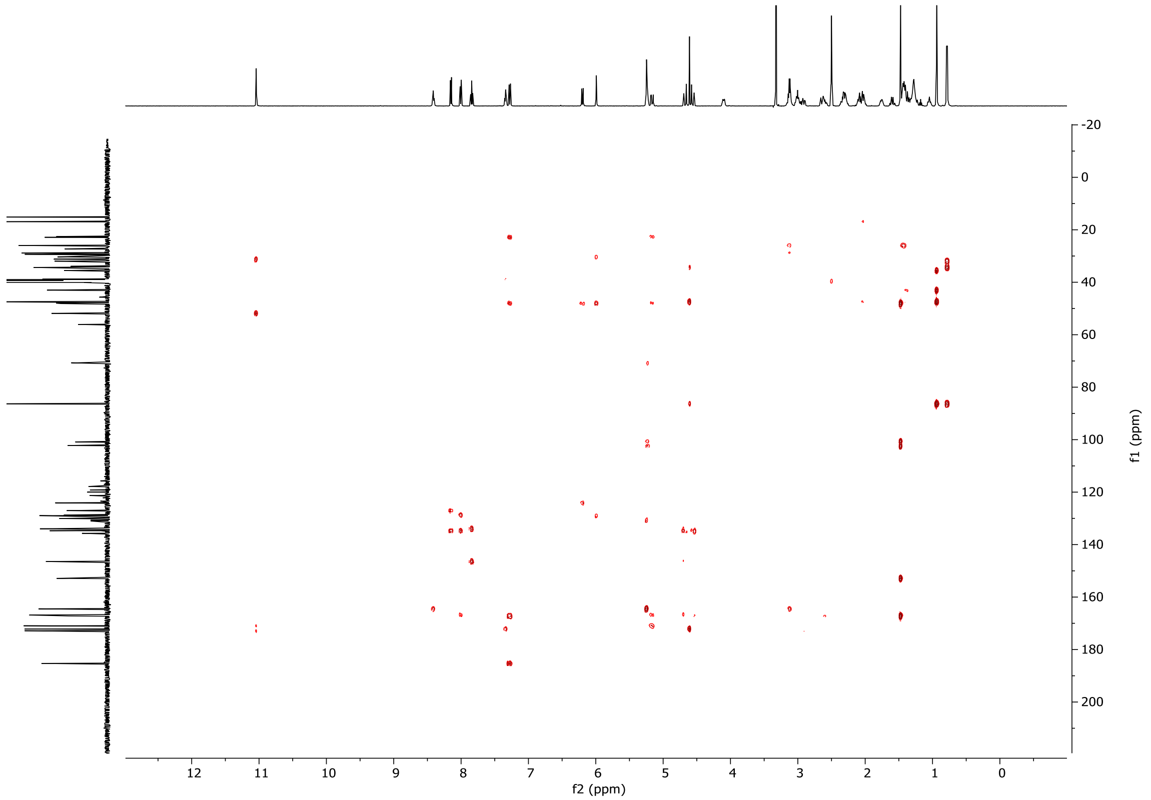

COSY (400 MHz, $(CD_3)_2SO$) of compound **KH-5-331**:

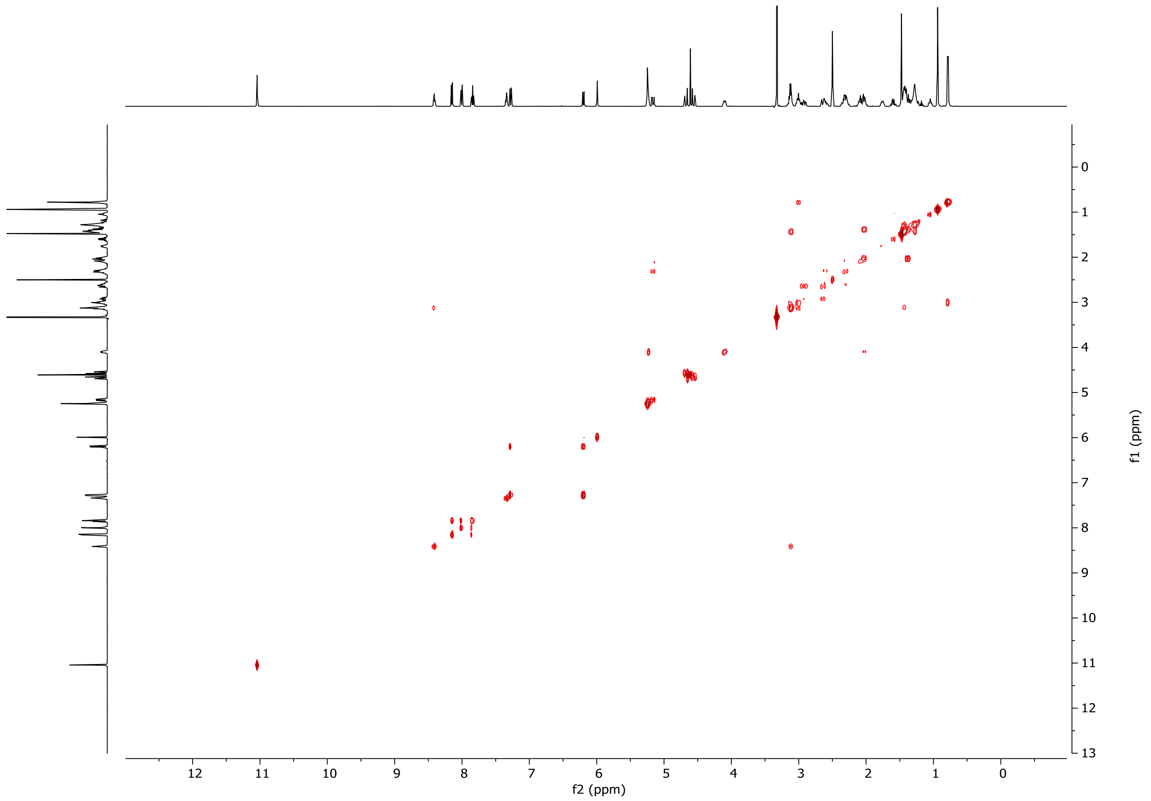

^{1}H NMR (500 MHz, $(CD_3)_2SO$) of compound **KH-5-336**:

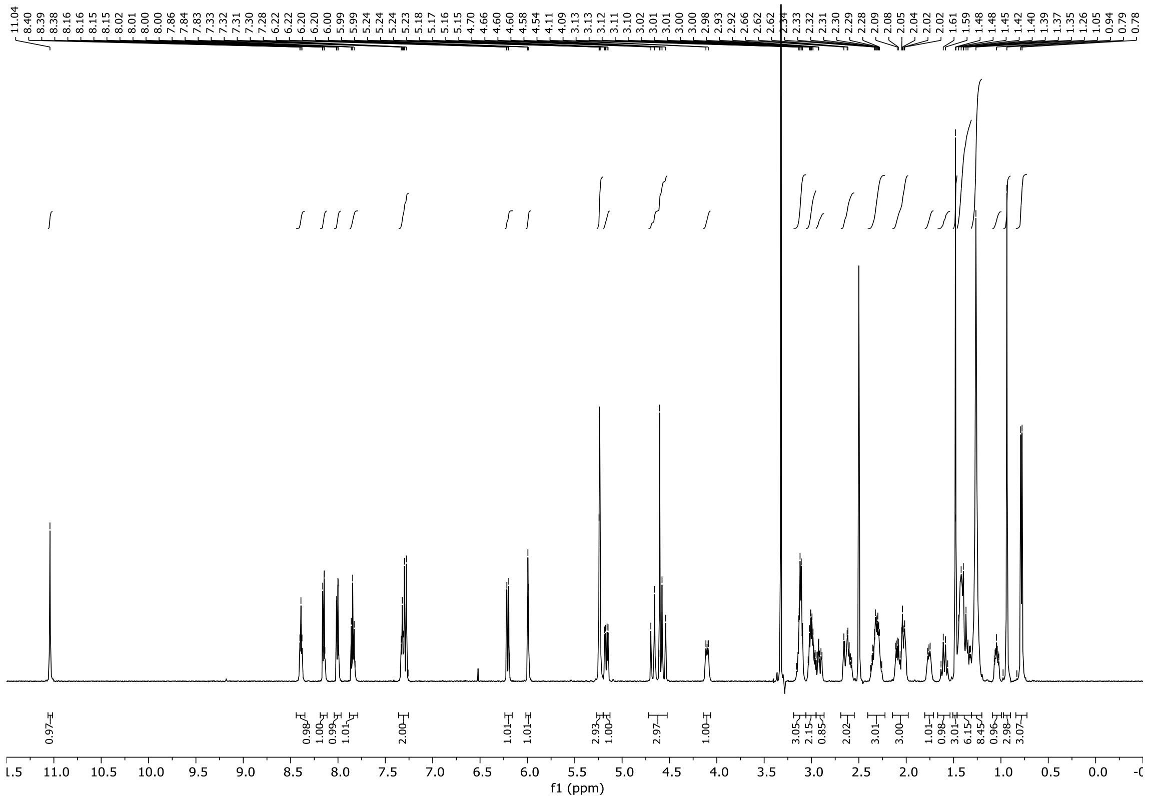

^{13}C NMR (126 MHz, $(CD_3)_2SO$) of compound **KH-5-336**:

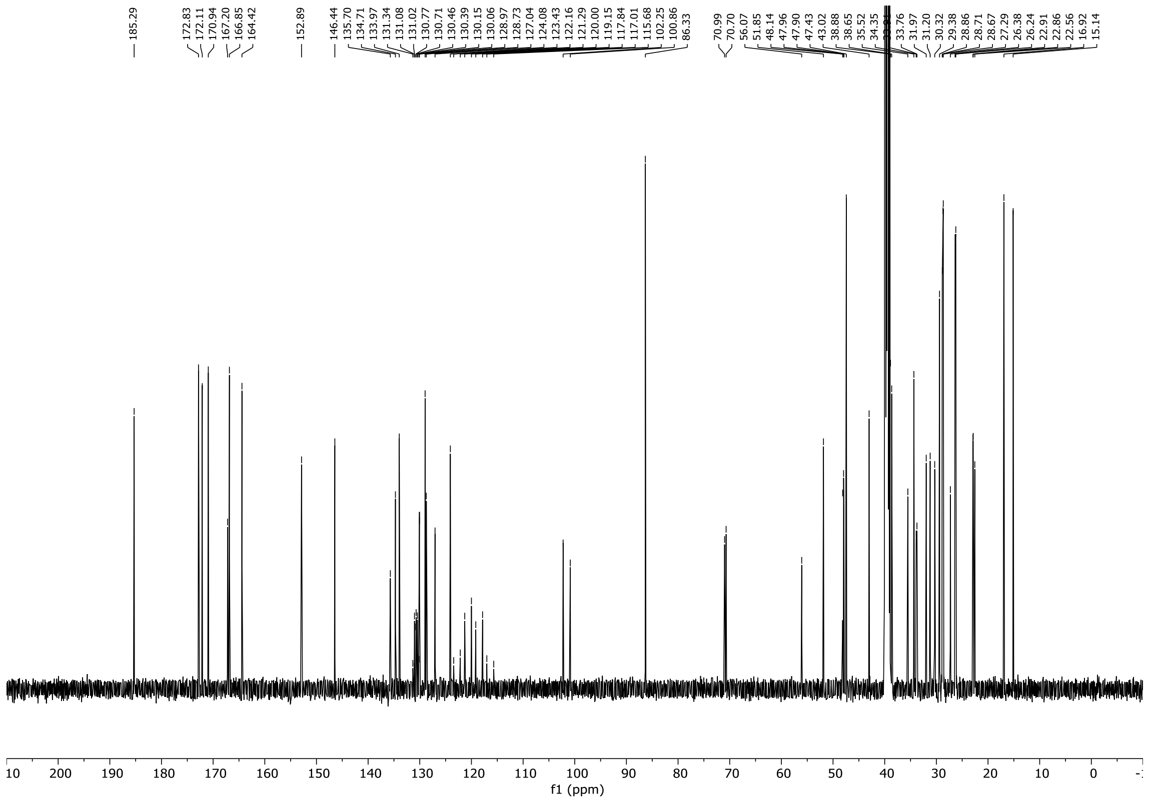

^{19}F NMR (471 MHz, $(CD_3)_2SO$) of compound **KH-5-336**:

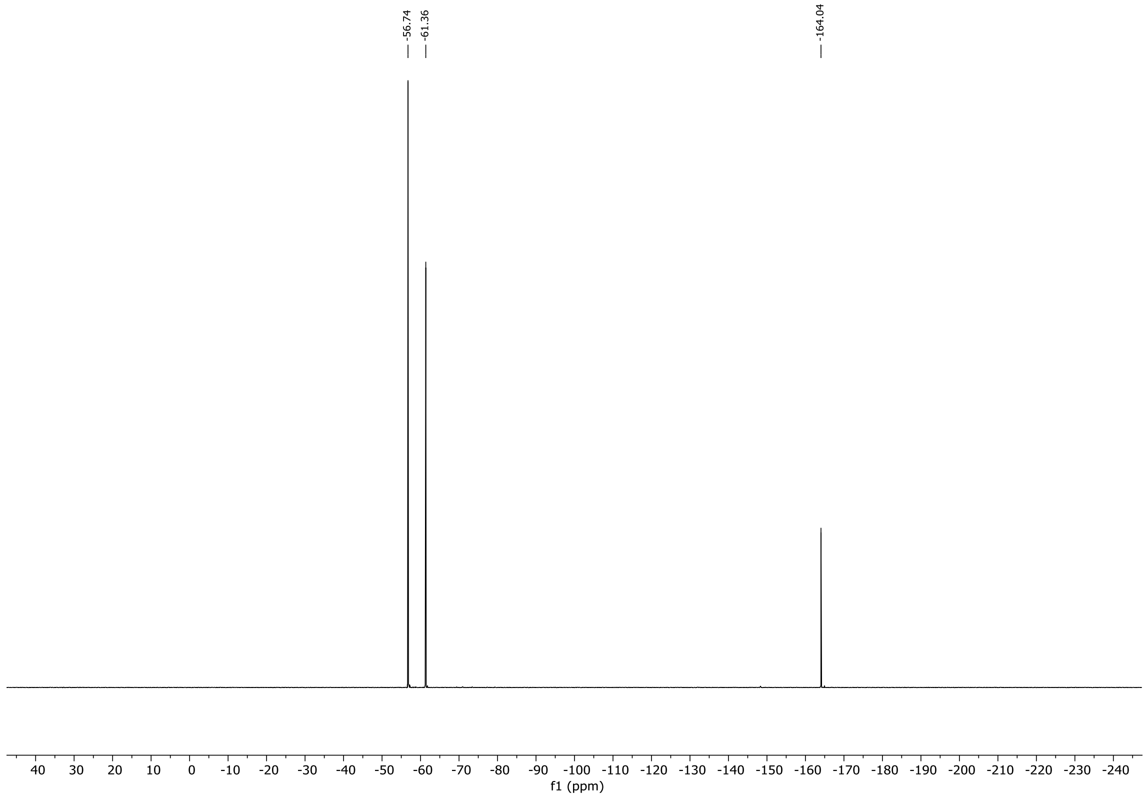

HSQC (500/126 MHz, $(CD_3)_2SO$) of compound **KH-5-336**:

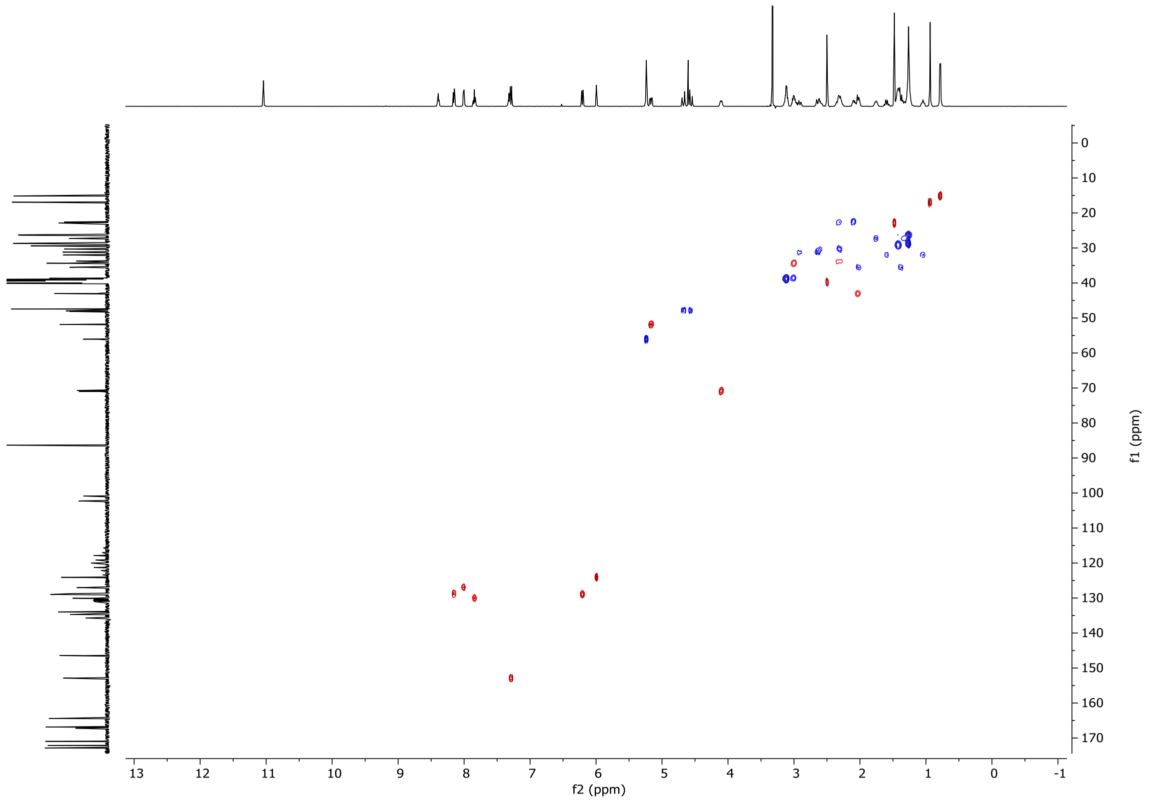

HMBC (500/126 MHz, $(CD_3)_2SO$) of compound **KH-5-336**:

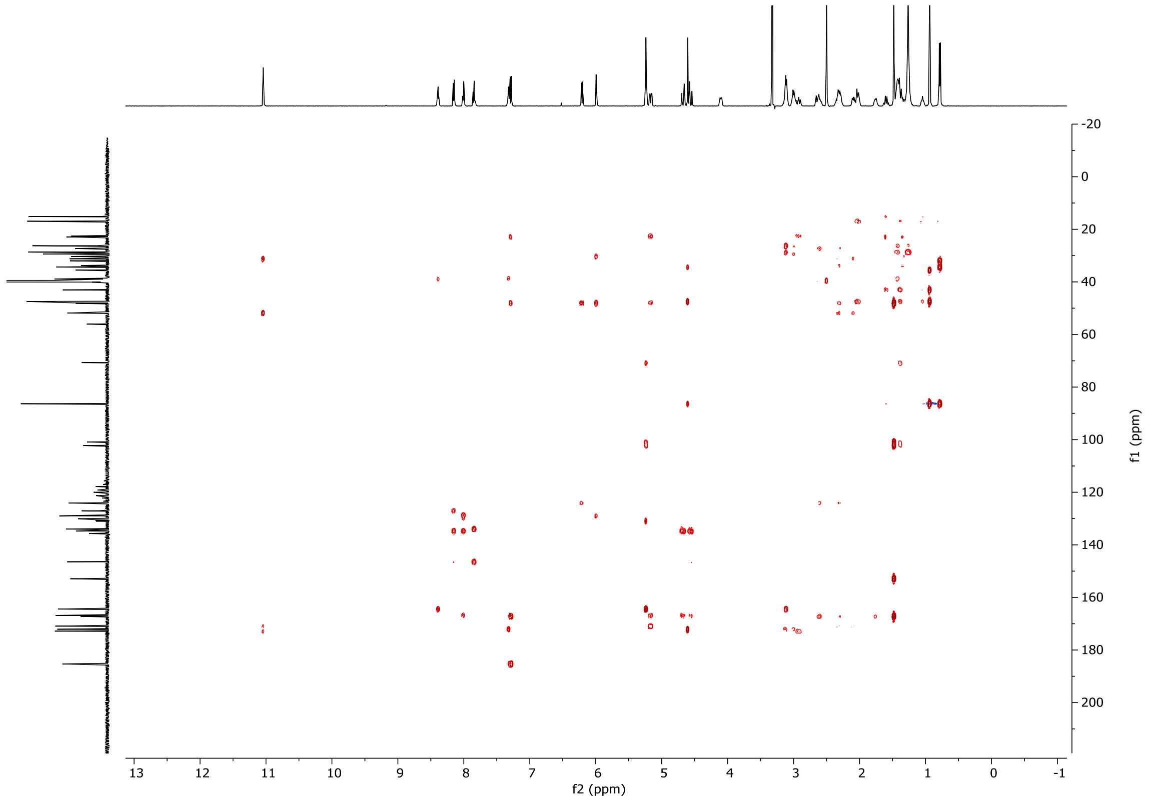

COSY (500 MHz, $(CD_3)_2SO$) of compound **KH-5-336**:

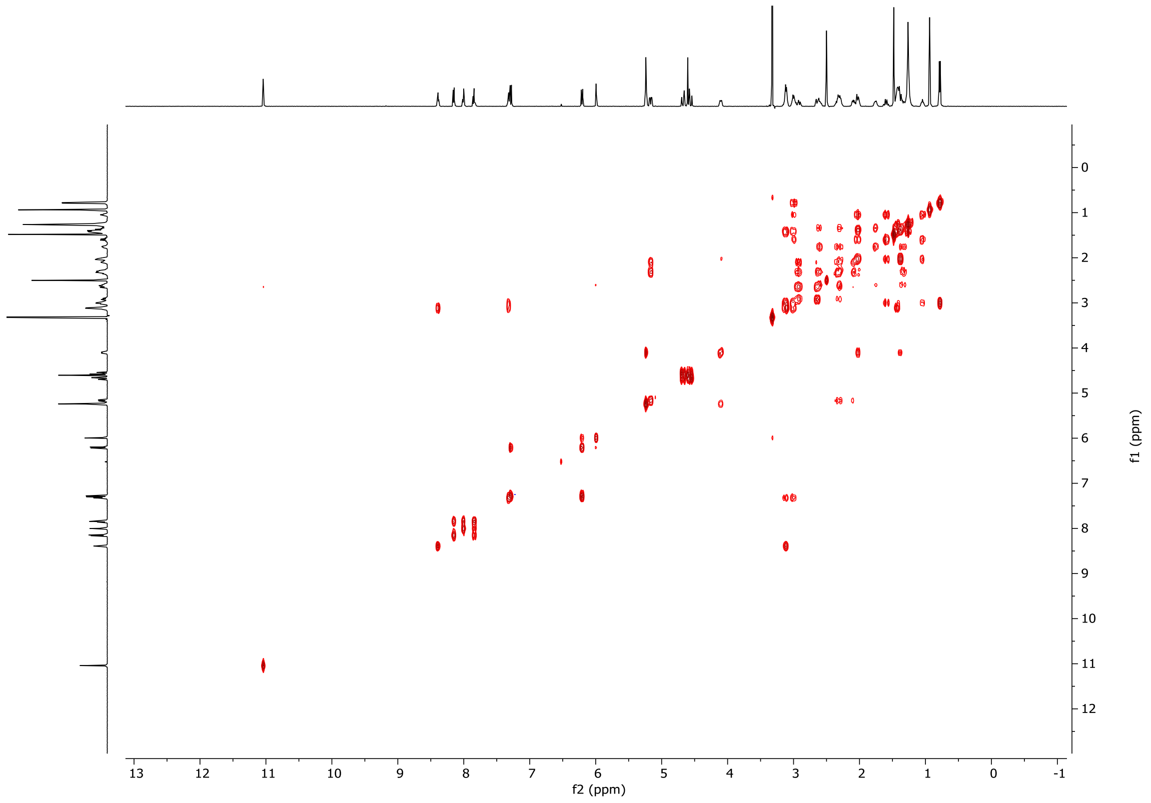

^{1}H NMR (500 MHz, $(CD_3)_2SO$) of compound **KH-5-340**:

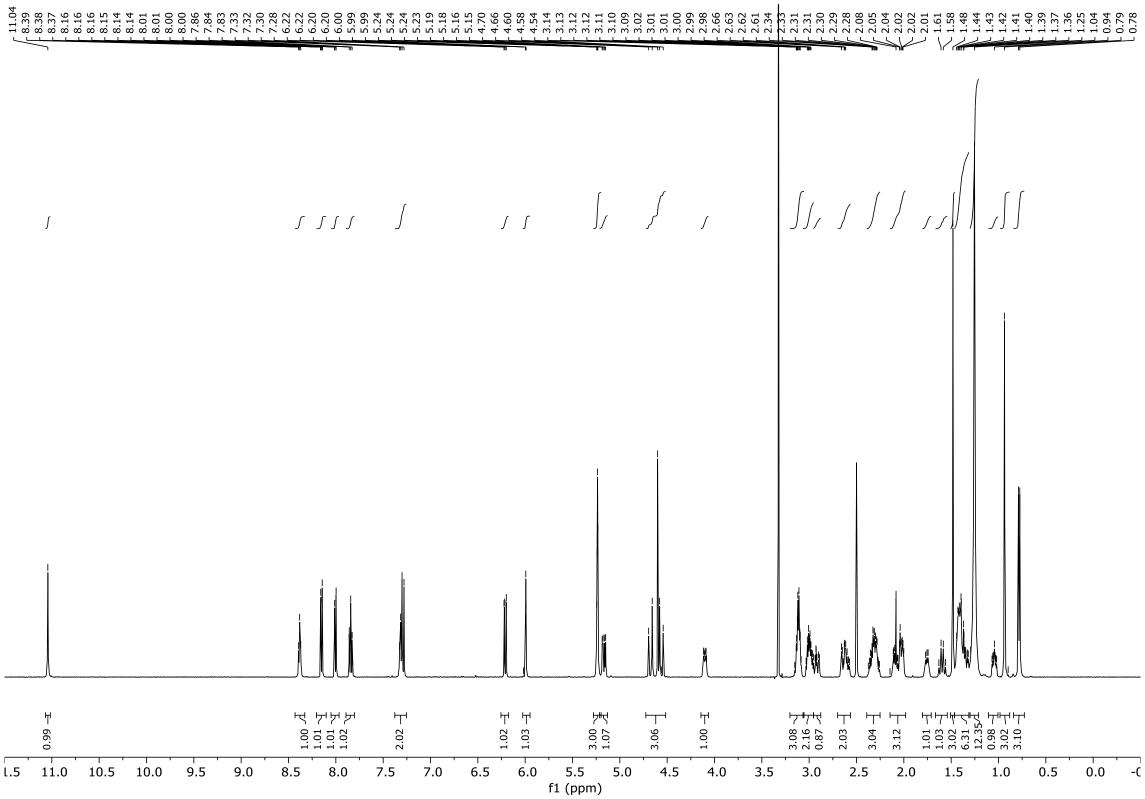

^{13}C NMR (126 MHz, $(CD_3)_2SO$) of compound **KH-5-340**:

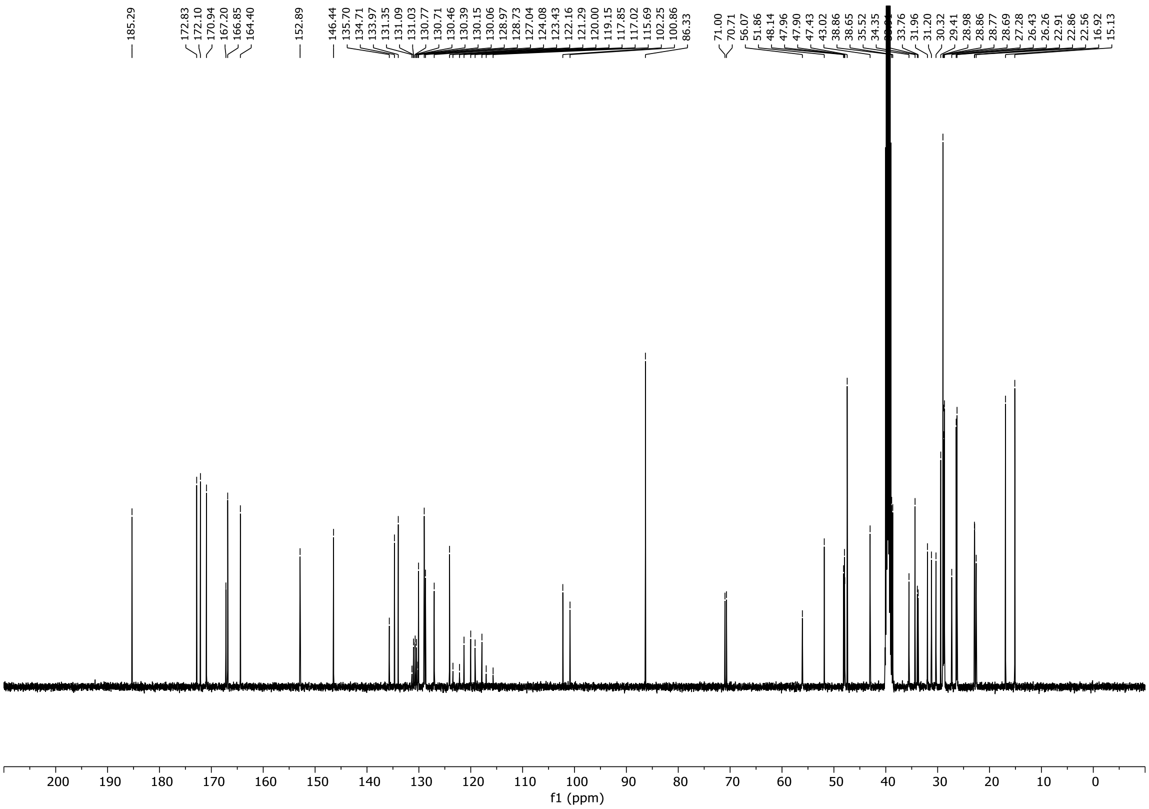

^{19}F NMR (471 MHz, $(CD_3)_2SO$) of compound **KH-5-340**:

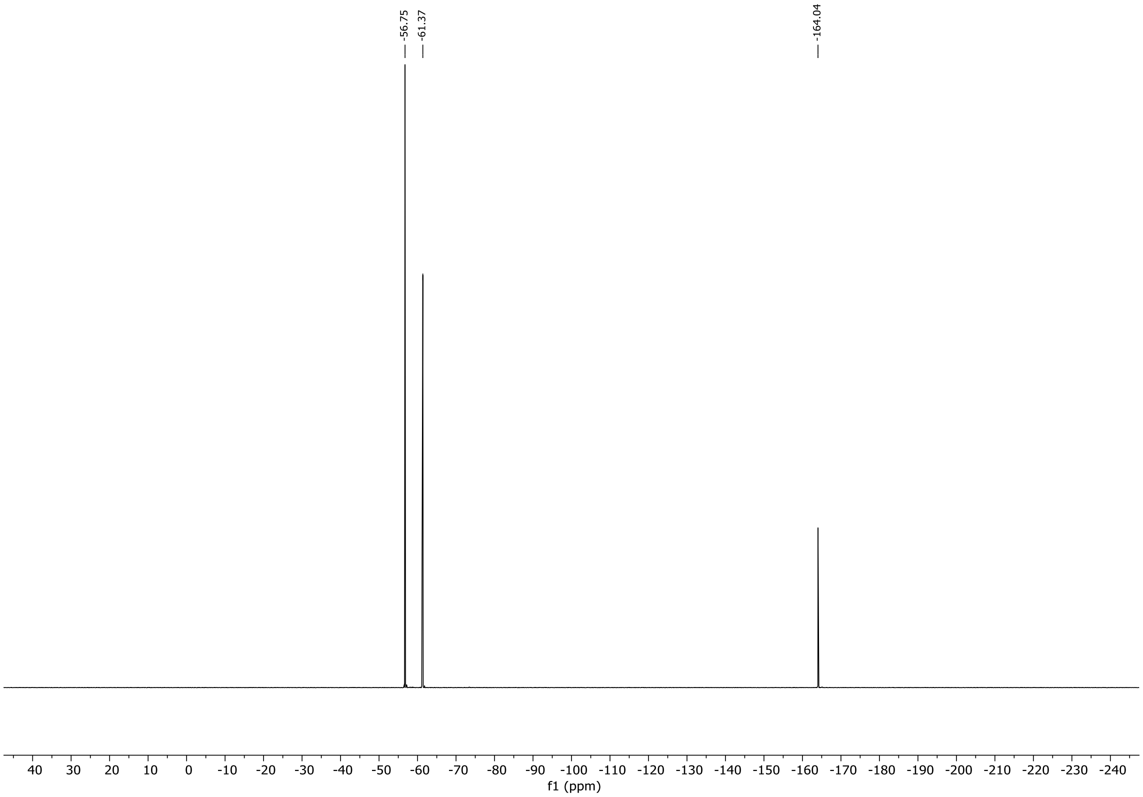

HSQC (500/126 MHz, $(CD_3)_2SO$) of compound **KH-5-340**:

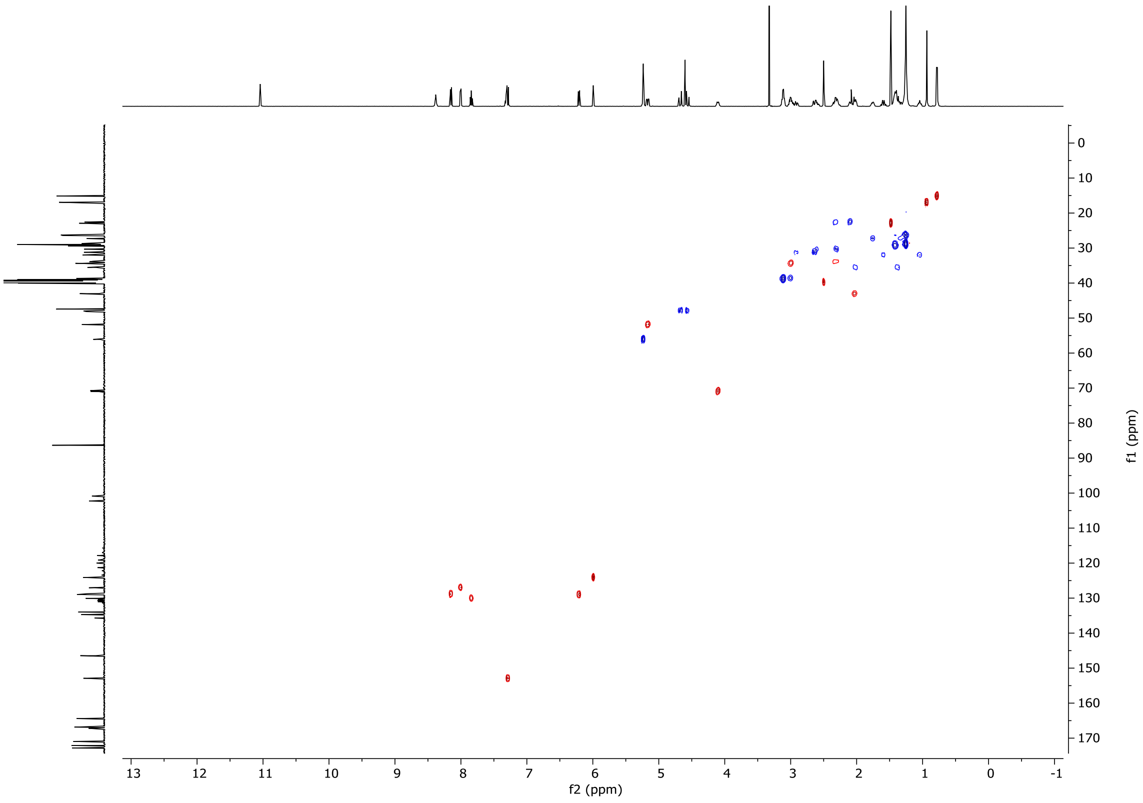

HMBC (500/126 MHz, $(CD_3)_2SO$) of compound **KH-5-340**:

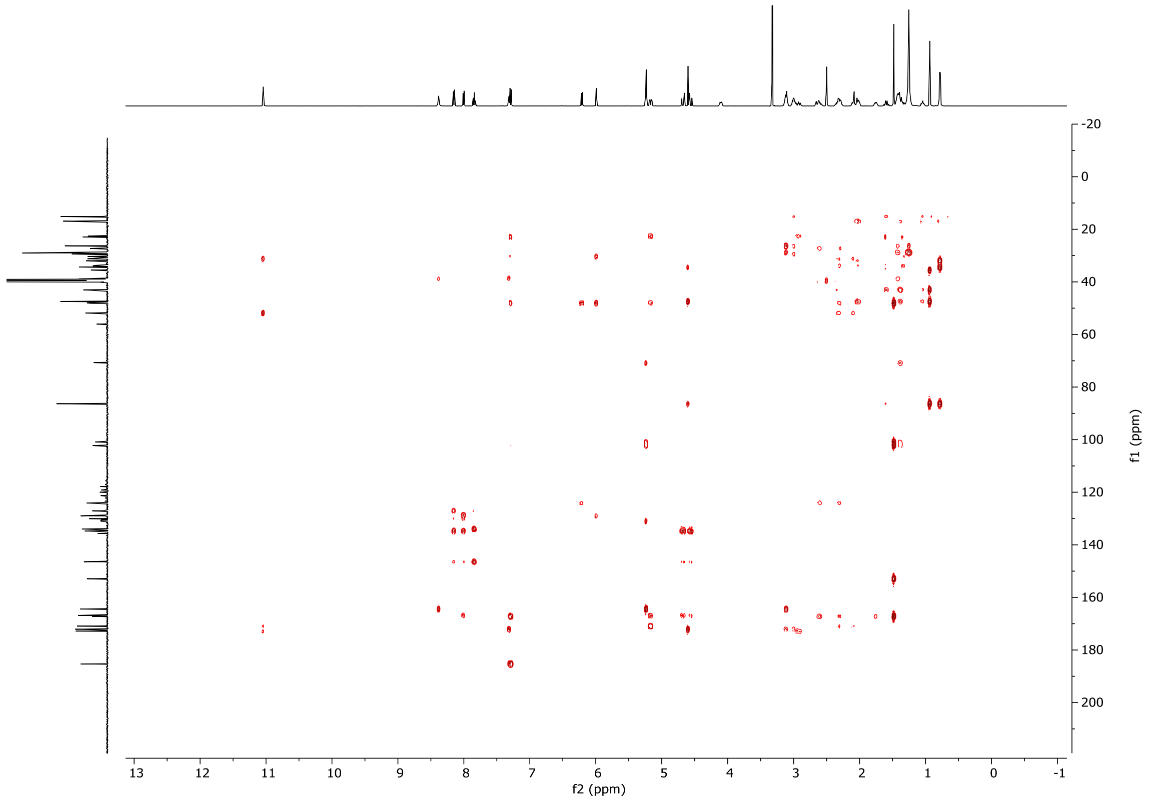

COSY (500 MHz, $(CD_3)_2SO$) of compound **KH-5-340**:

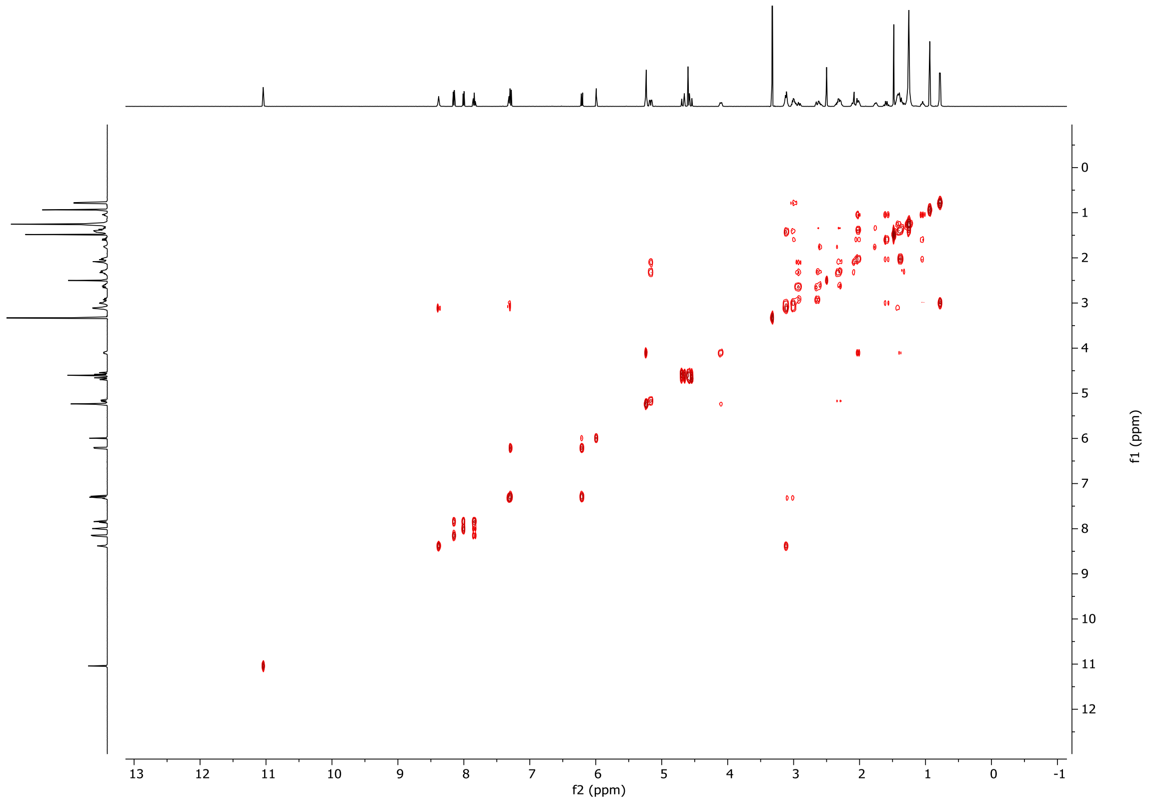

(OEt)$_2$-Arylazopyrazole photoPROTACs

^{1}H NMR (400 MHz, $CDCl_3$) of compound **51**:

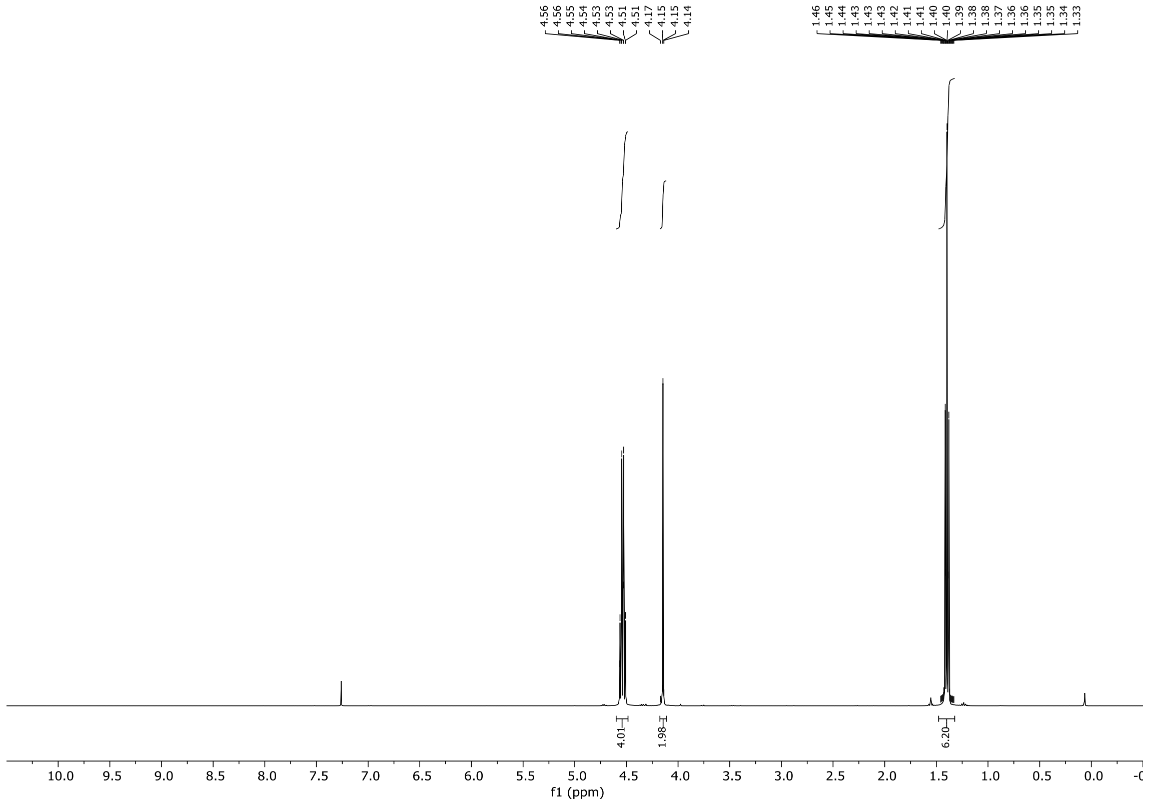

^{13}C NMR (101 MHz, $CDCl_3$) of compound **51**:

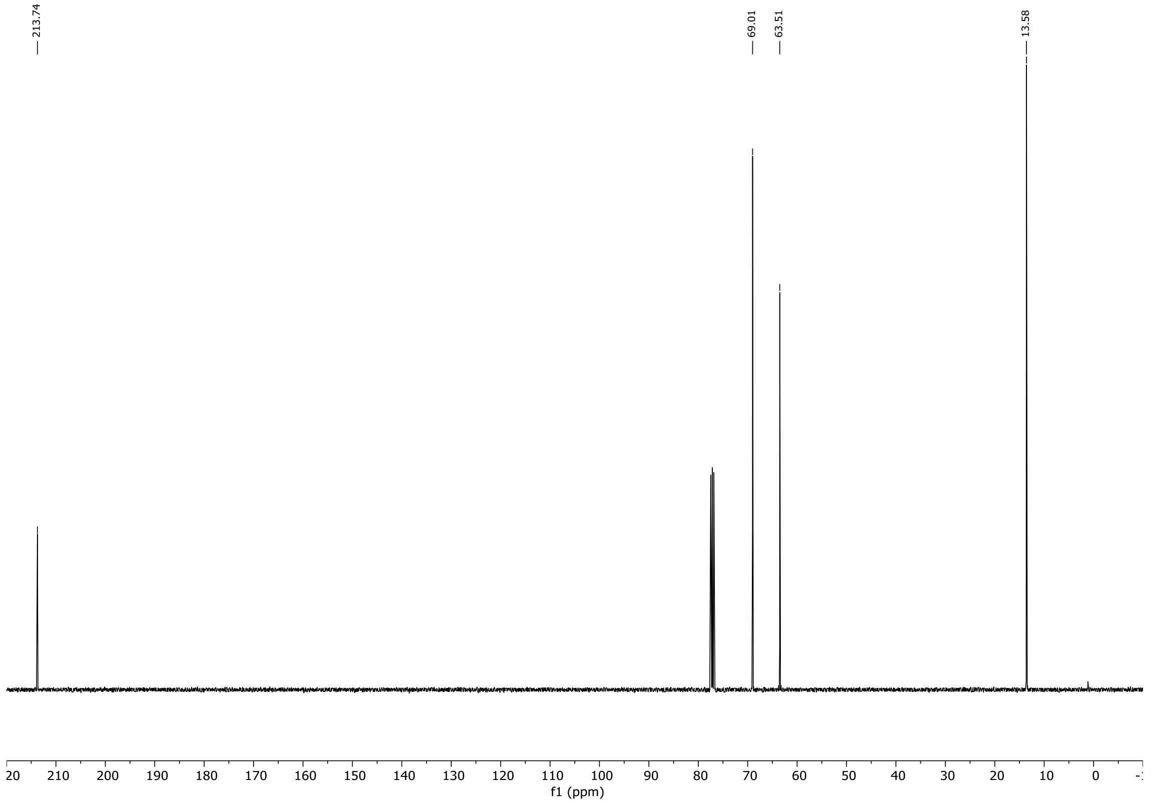

^{1}H NMR (400 MHz, $CDCl_3$) of compound **52**:

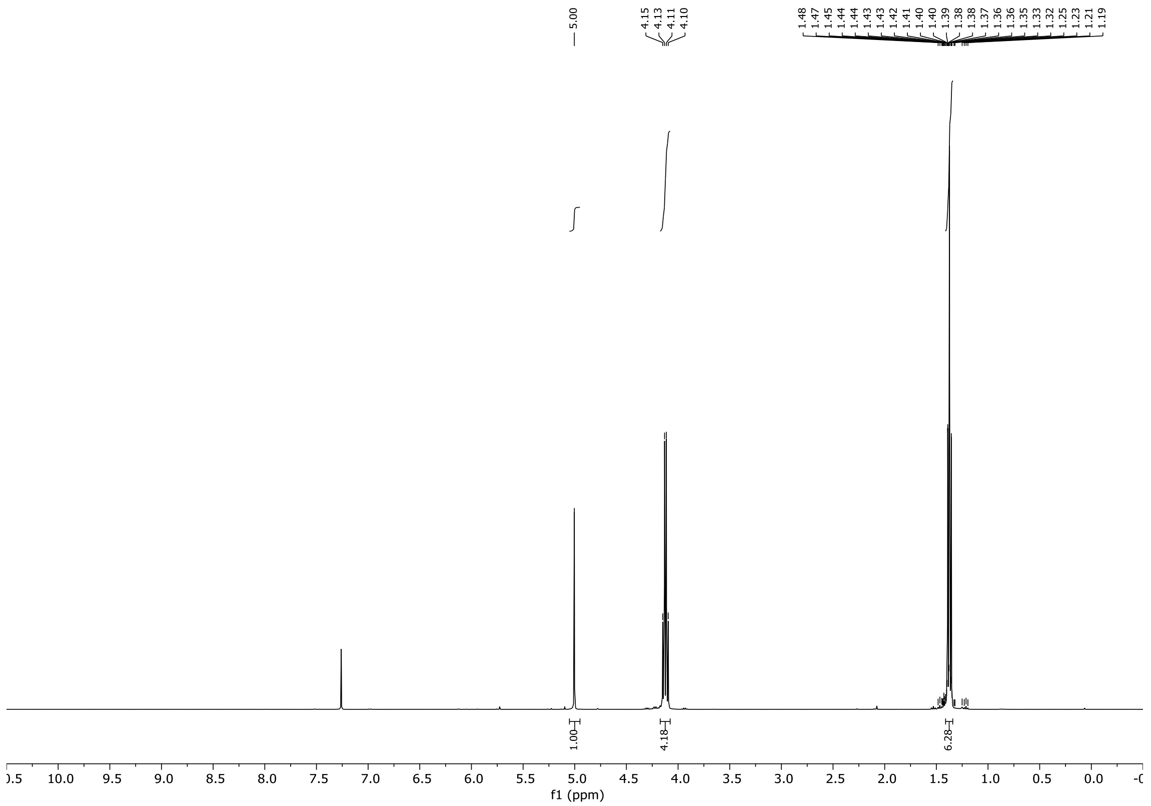

^{13}C NMR (101 MHz, $CDCl_3$) of compound **52**:

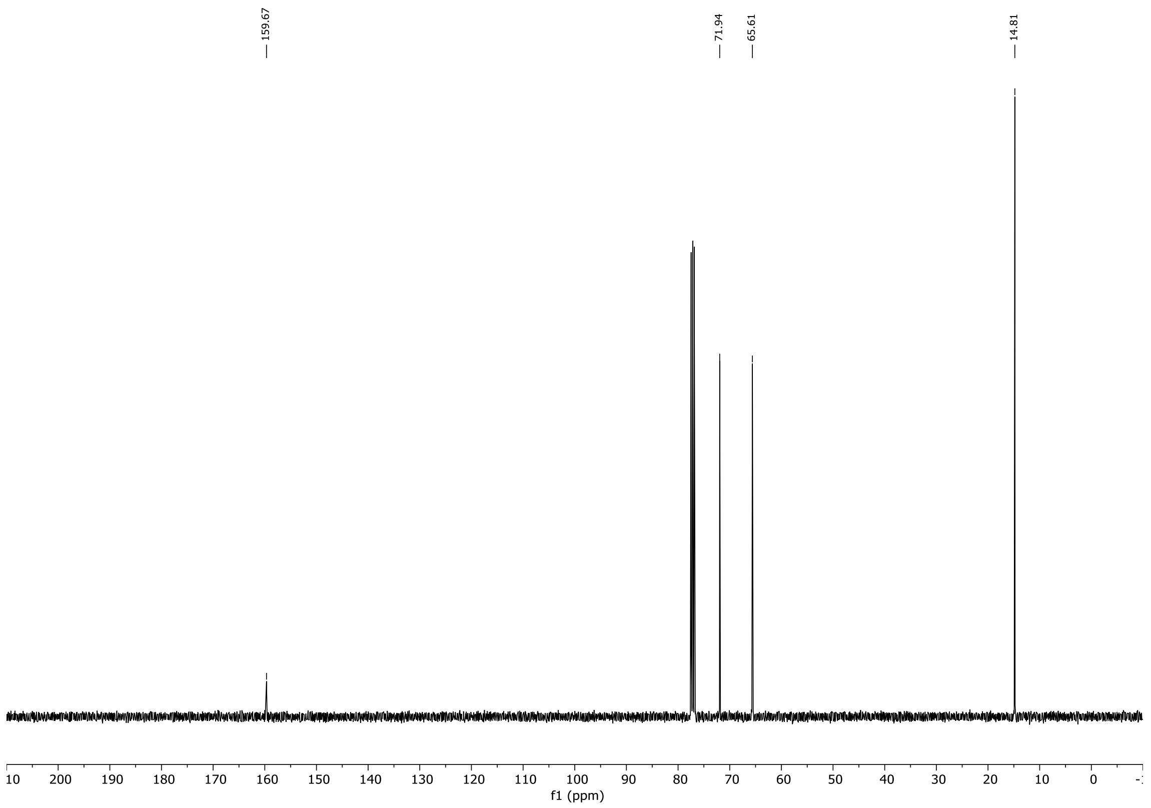

HSQC (400/101 MHz, $CDCl_3$) of compound **52**:

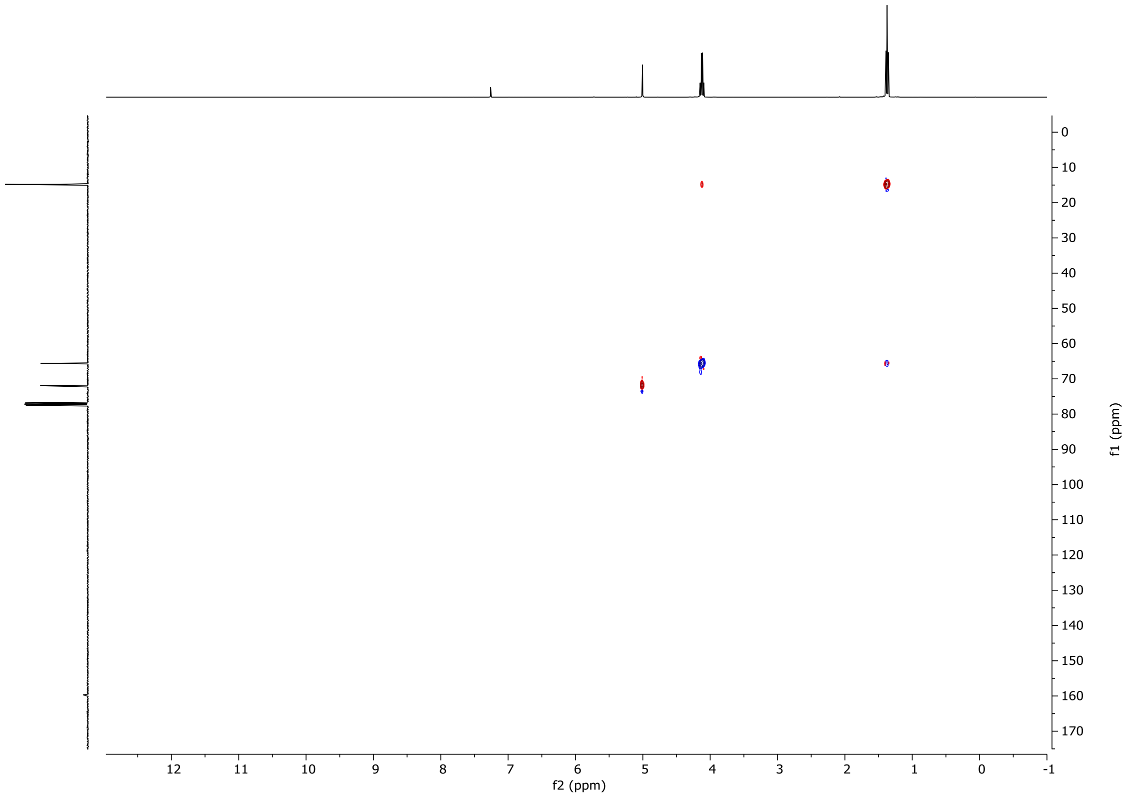

HMBC (400/101 MHz, $CDCl_3$) of compound **52**:

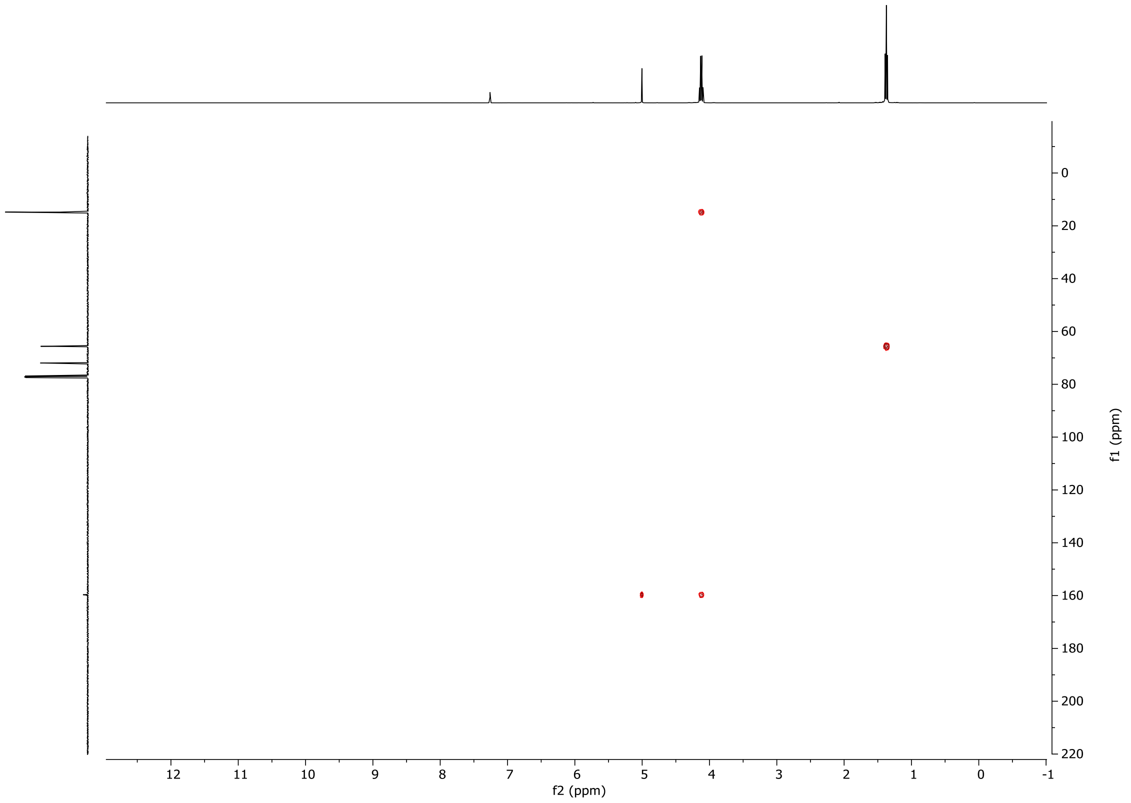

COSY (400 MHz, $CDCl_3$) of compound **52**:

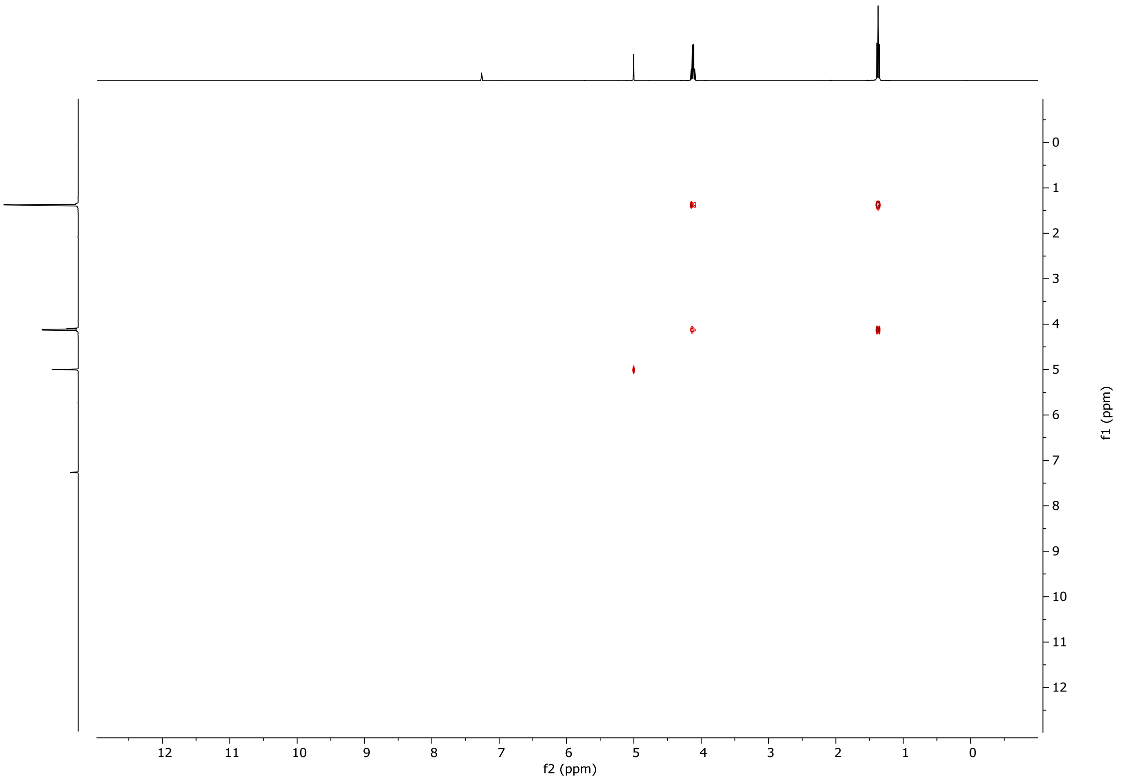

^{1}H NMR (400 MHz, $(CD_3)_2CO$) of compound **54**:

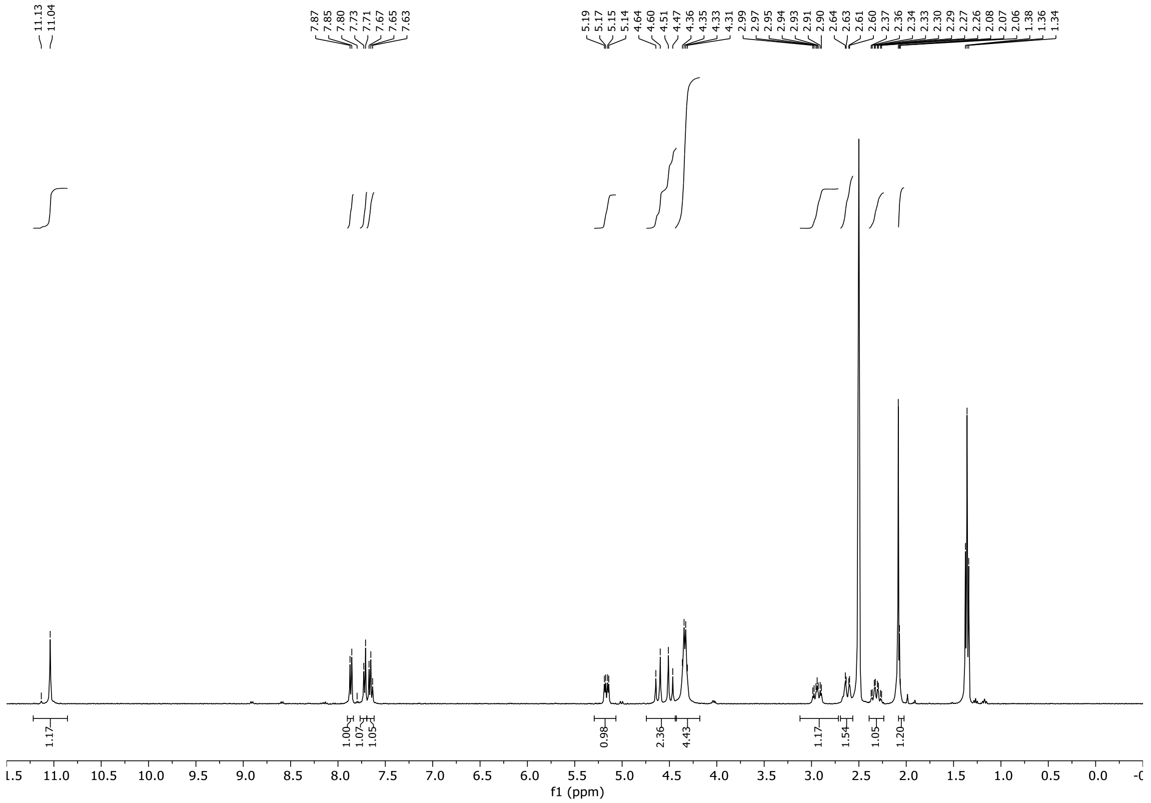

^{13}C NMR (101 MHz, $(CD_3)_2CO$) of compound **54**:

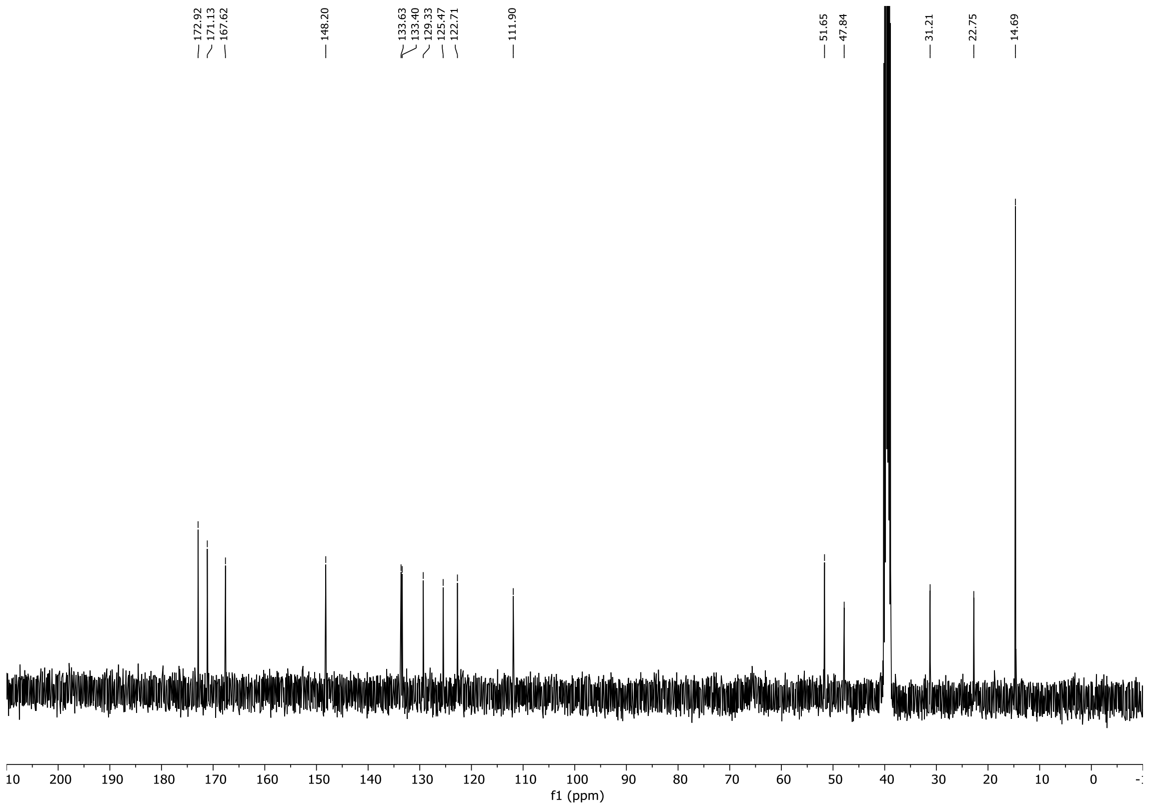

HSQC (400/101 MHz, $(CD_3)_2CO$) of compound **54**:

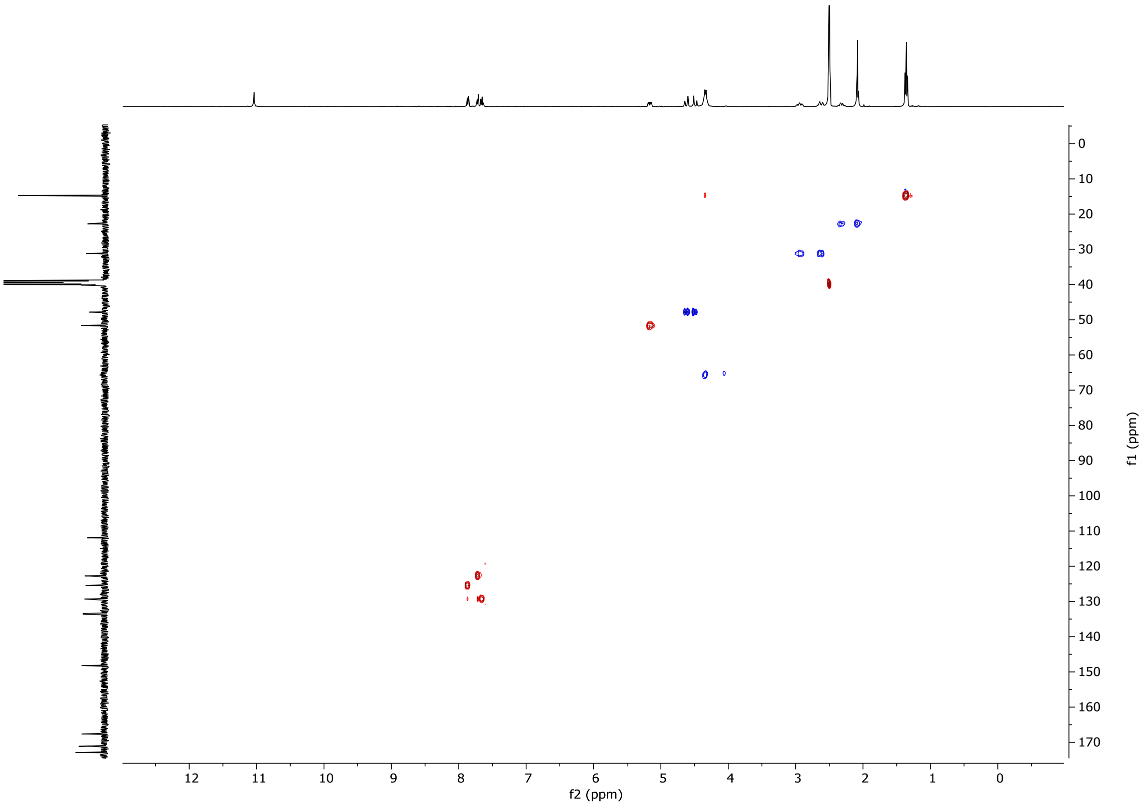

HMBC (400/101 MHz, $(CD_3)_2CO$) of compound **54**:

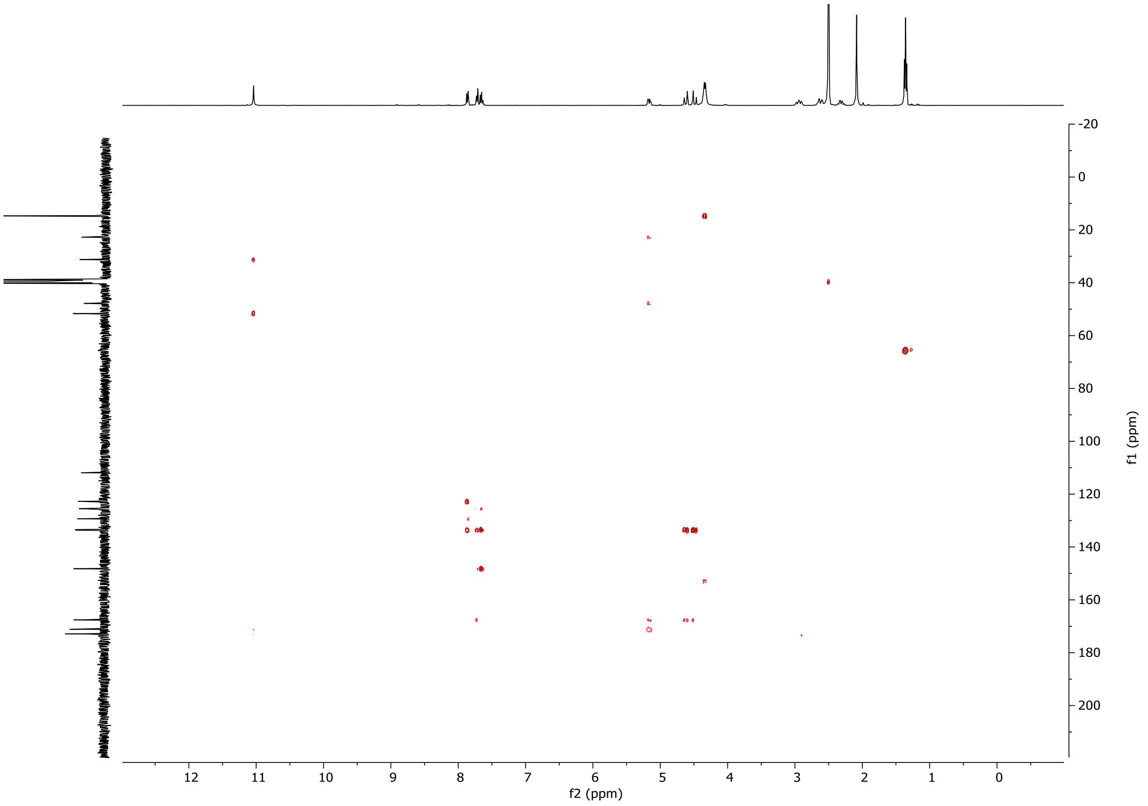

COSY (400 MHz, $(CD_3)_2CO$) of compound **54**:

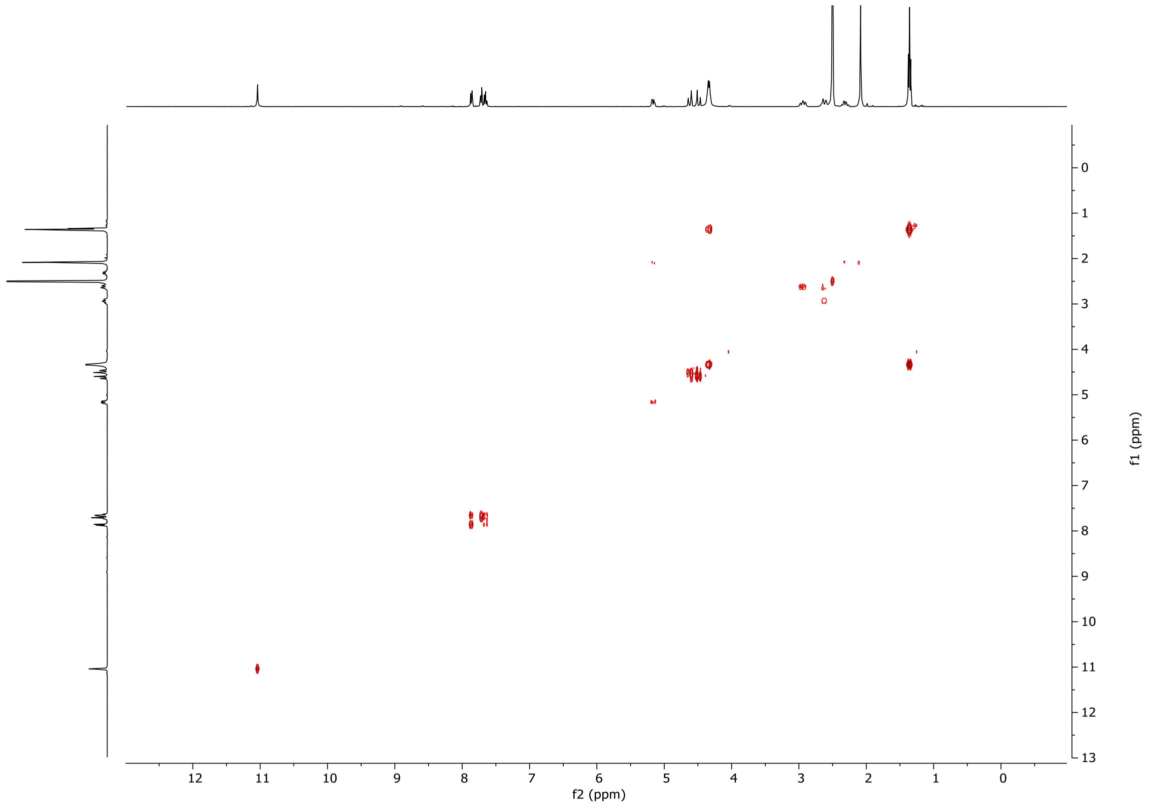

^{1}H NMR (400 MHz, $(CD_3)_2CO$) of compound **(OEt)$_2$-arylazopyrazole photoswitch**:

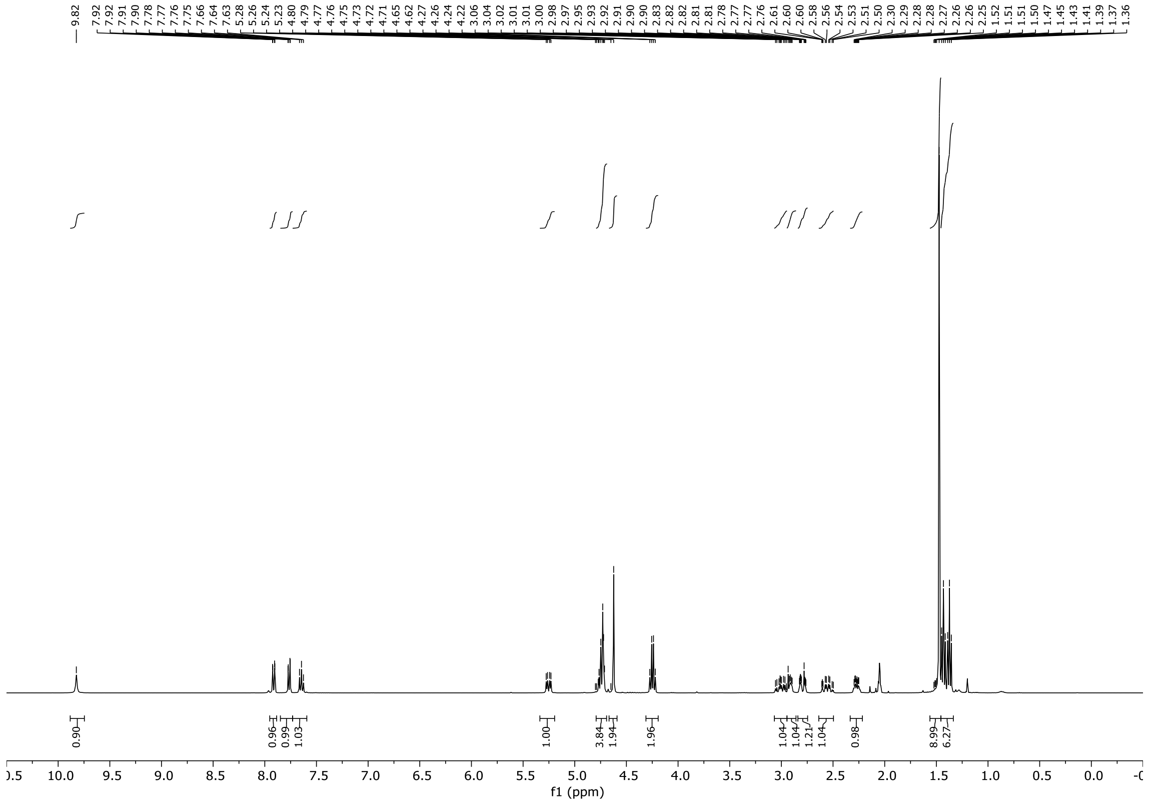

^{13}C NMR (101 MHz, $(CD_3)_2CO$) of compound **(OEt)$_2$-arylazopyrazole photoswitch**:

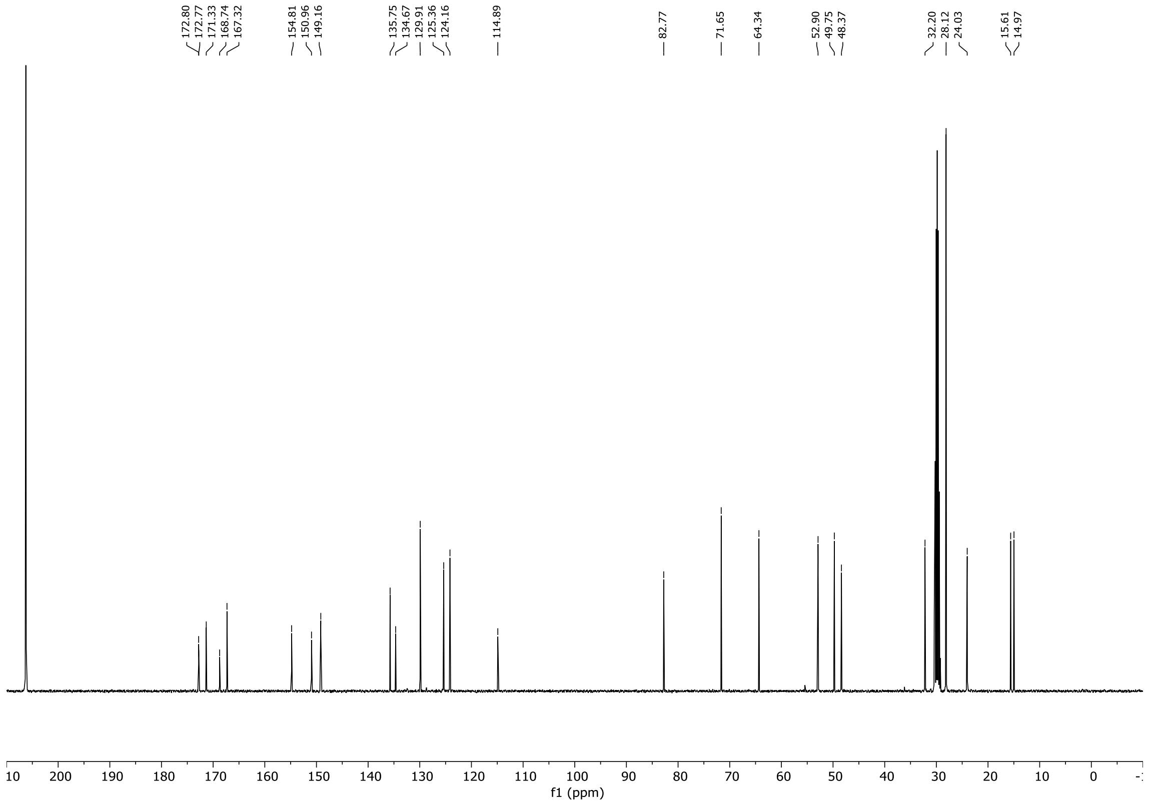

HSQC (400/101 MHz, $(CD_3)_2CO$) of compound **$(OEt)_2$-arylazopyrazole photoswitch**:

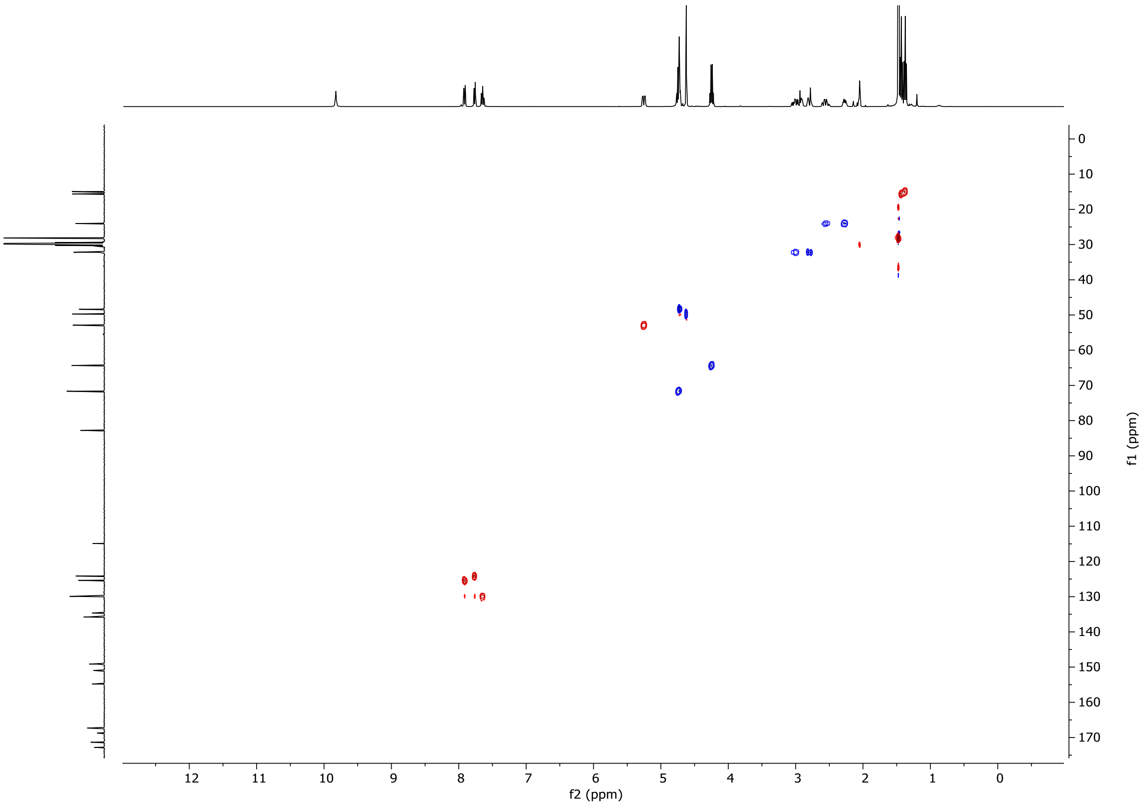

HMBC (400/101 MHz, $(CD_3)_2CO$) of compound **$(OEt)_2$-arylazopyrazole photoswitch**:

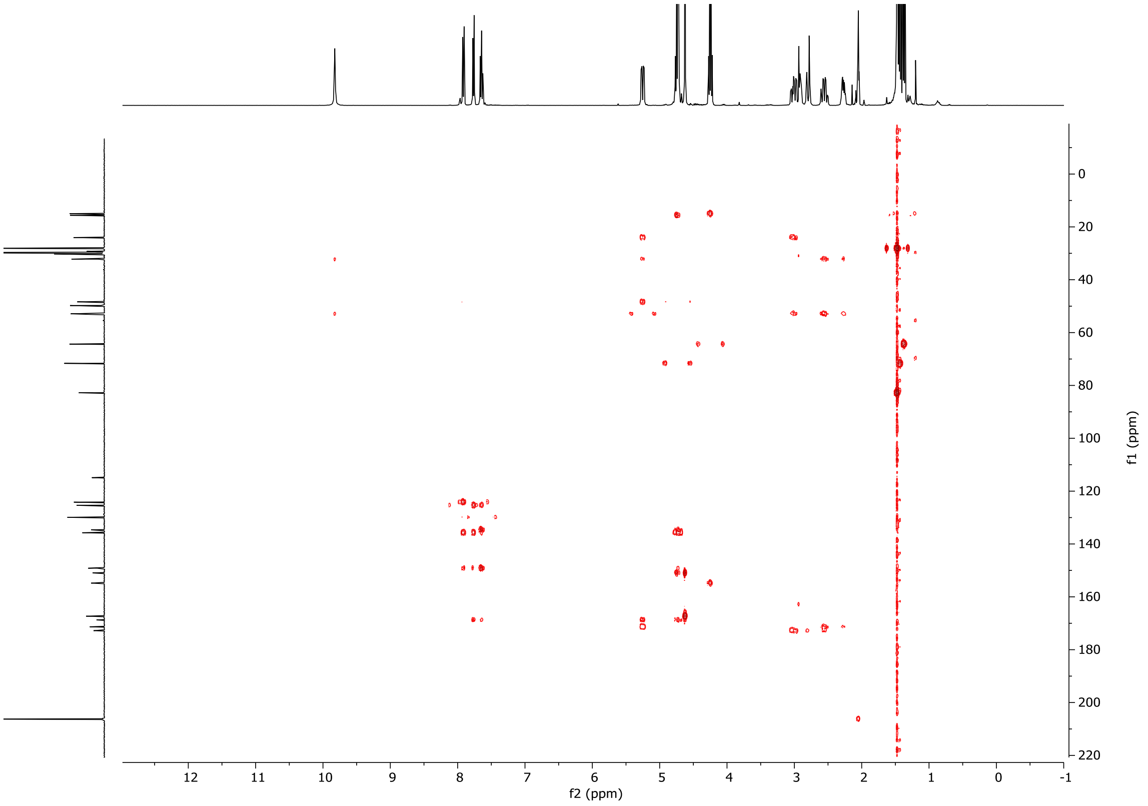

COSY (400 MHz, $(CD_3)_2CO$) of compound **(OEt)$_2$-arylazopyrazole photoswitch**:

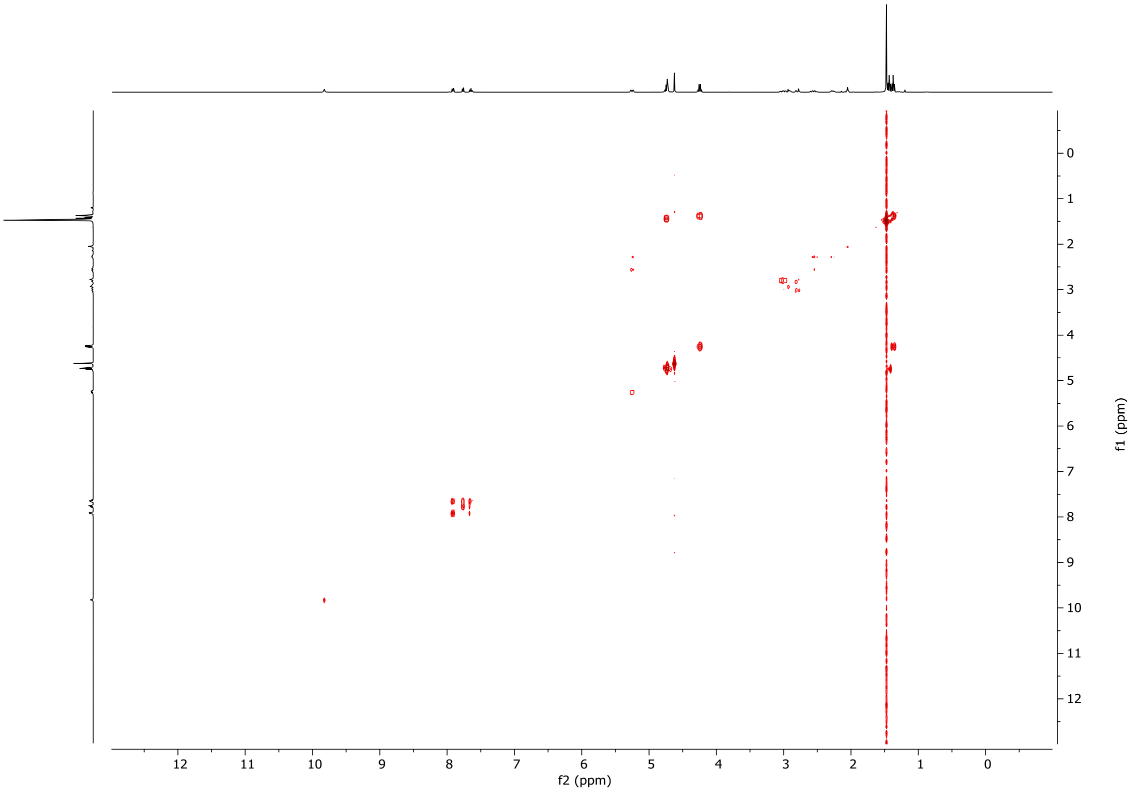

^{1}H NMR (400 MHz, $(CD_3)_2CO$) of compound **67**:

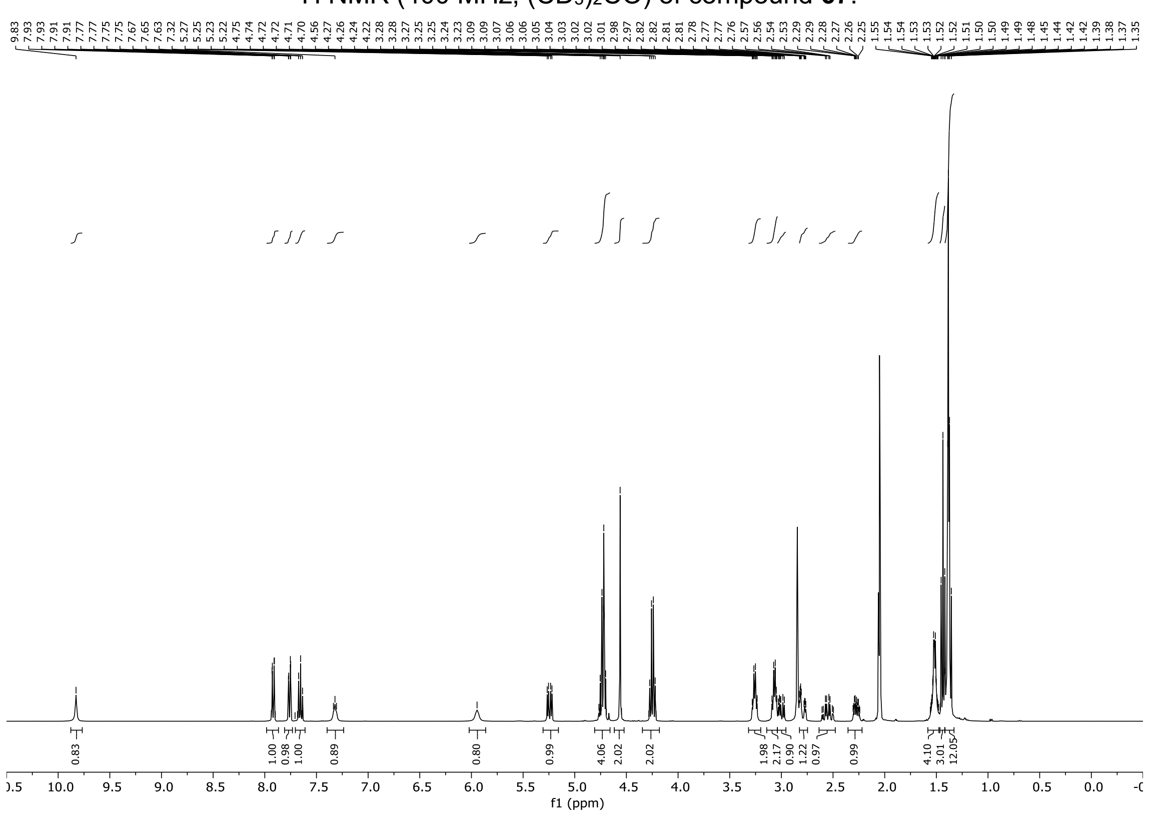

^{13}C NMR (101 MHz, $(CD_3)_2CO$) of compound **67**:

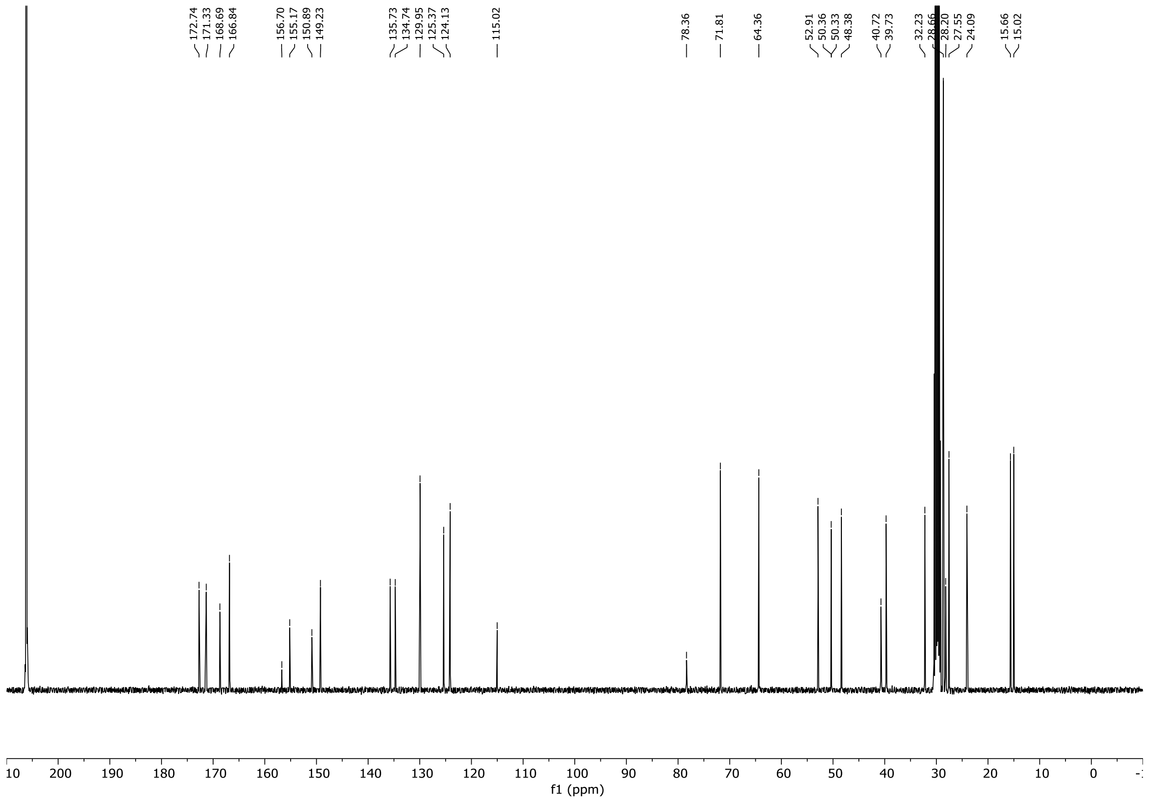

HSQC (400/101 MHz, $(CD_3)_2CO$) of compound **67**:

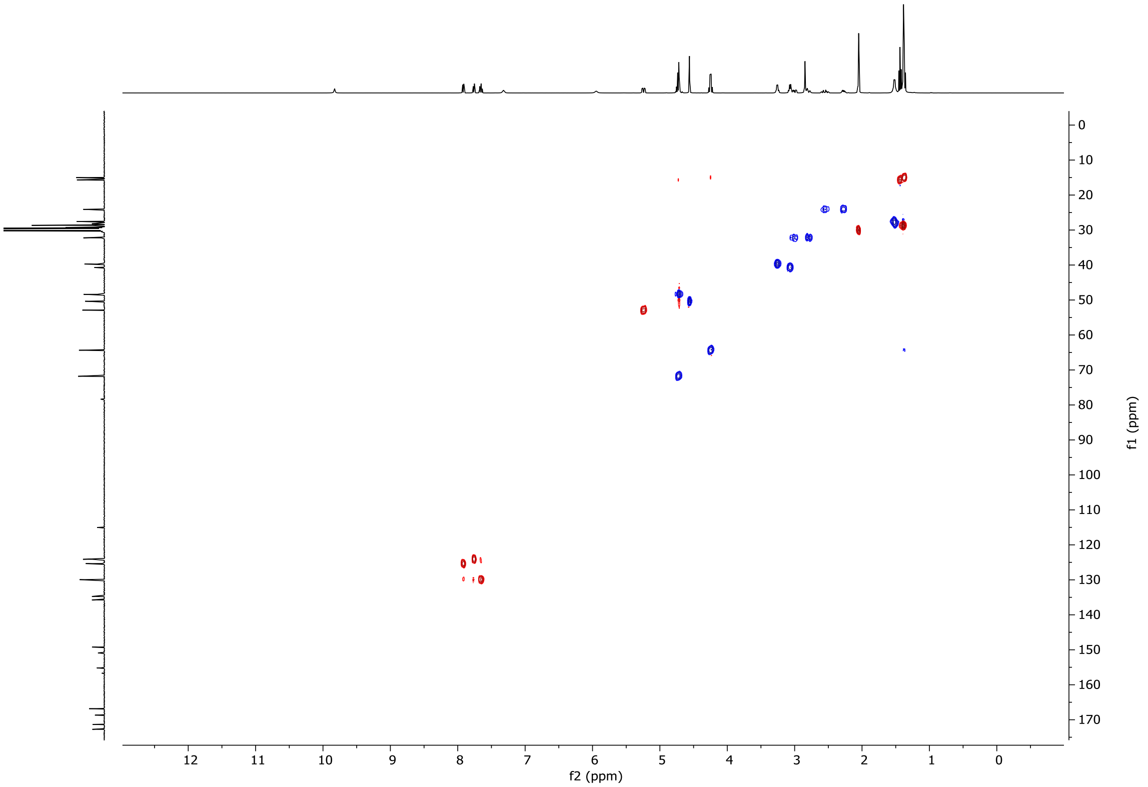

HMBC (400/101 MHz, $(CD_3)_2CO$) of compound **67**:

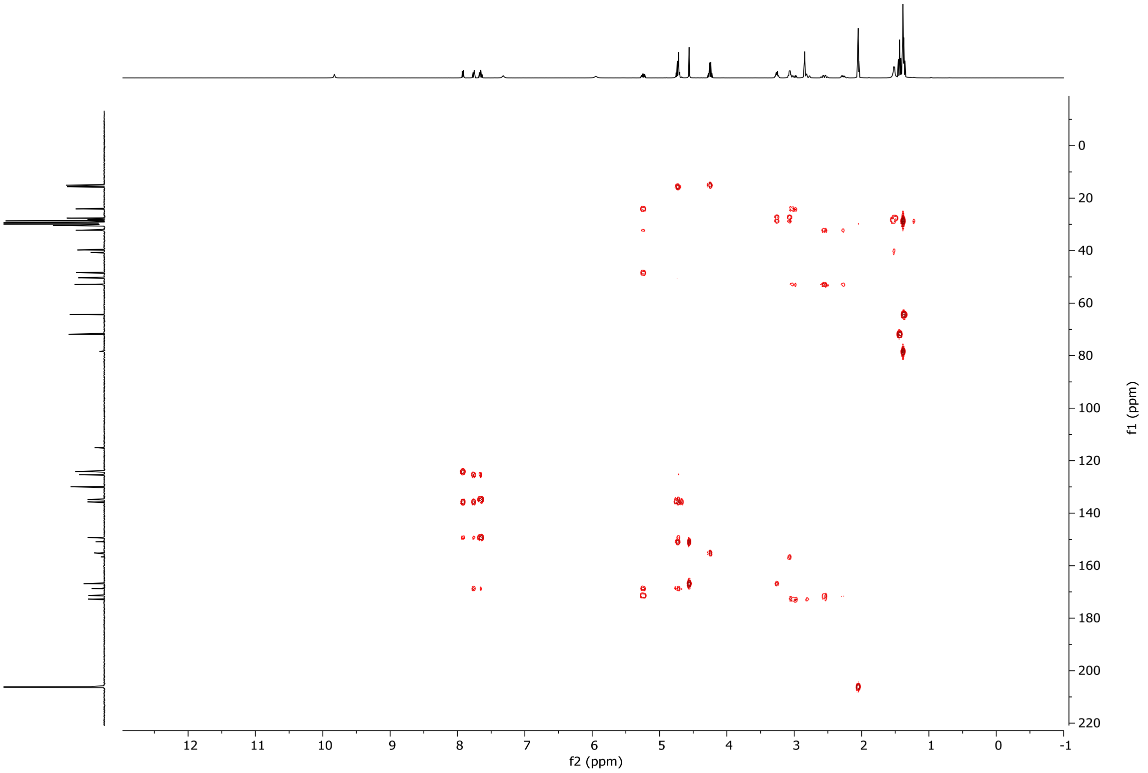

COSY (400 MHz, $(CD_3)_2CO$) of compound **67**:

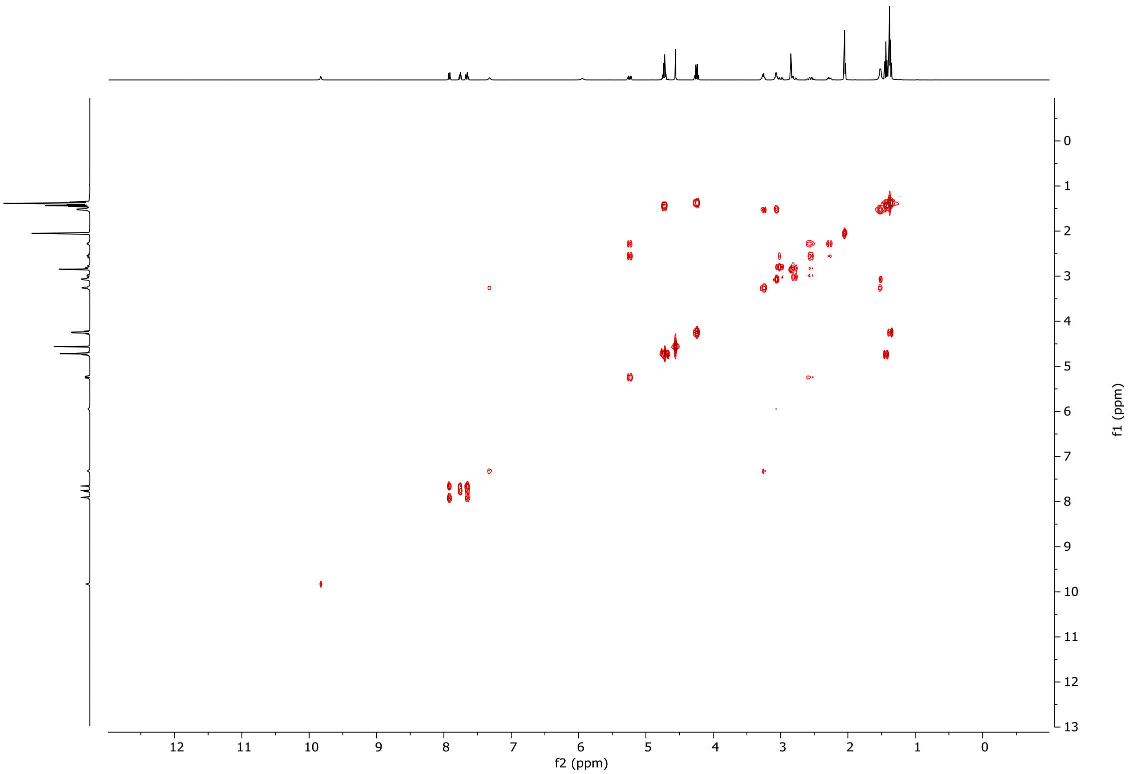

^{1}H NMR (400 MHz, $(CD_3)_2CO$) of compound **68**:

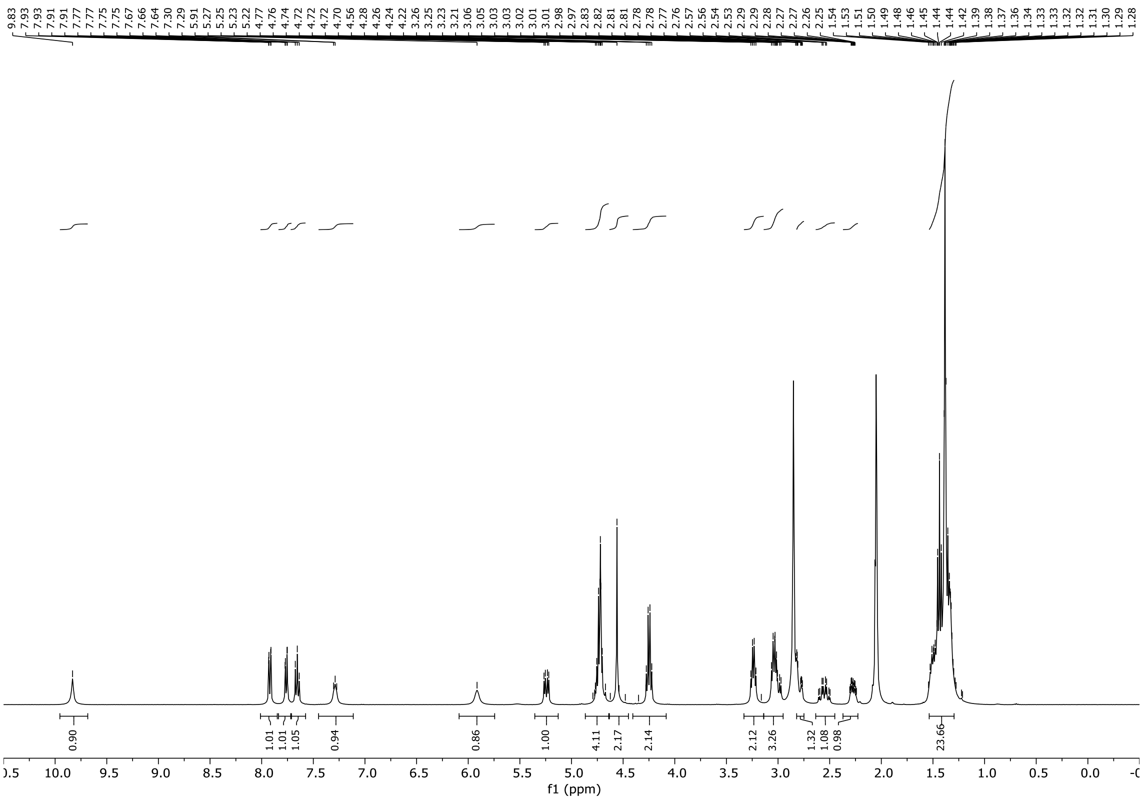

^{13}C NMR (101 MHz, $(CD_3)_2CO$) of compound **68**:

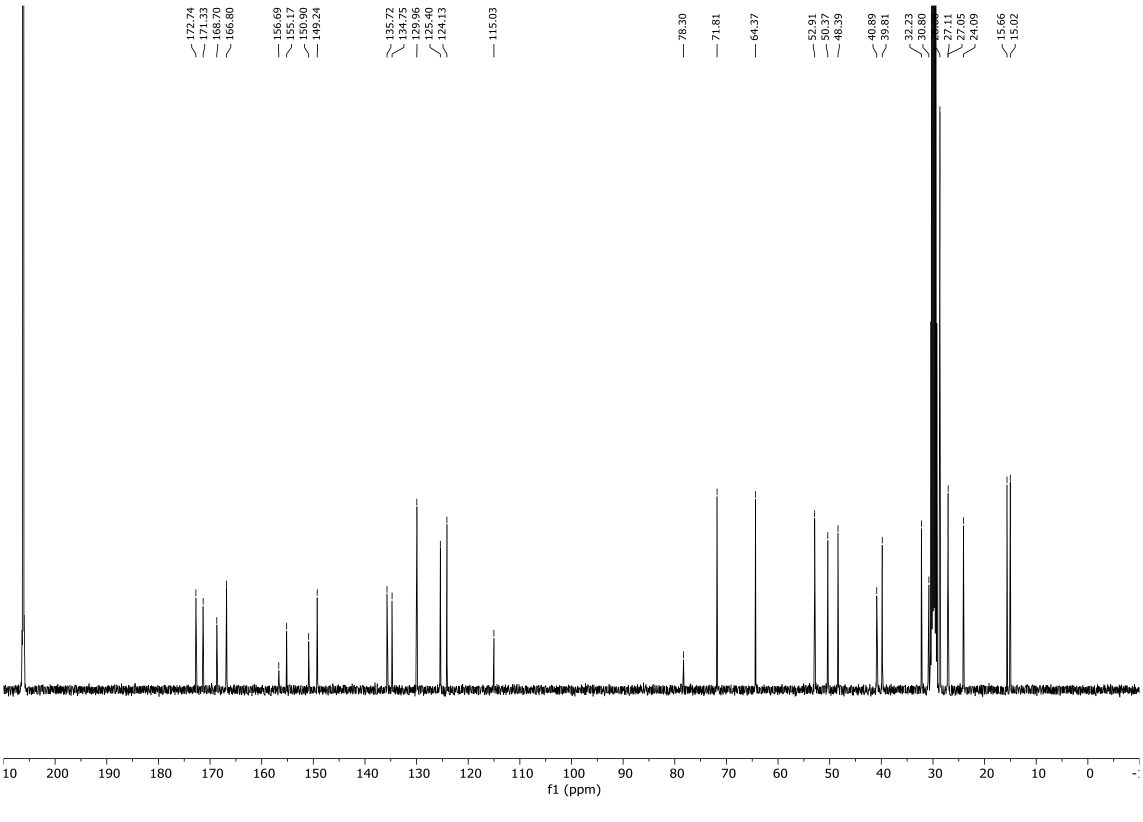

HSQC (400/101 MHz, $(CD_3)_2CO$) of compound **68**:

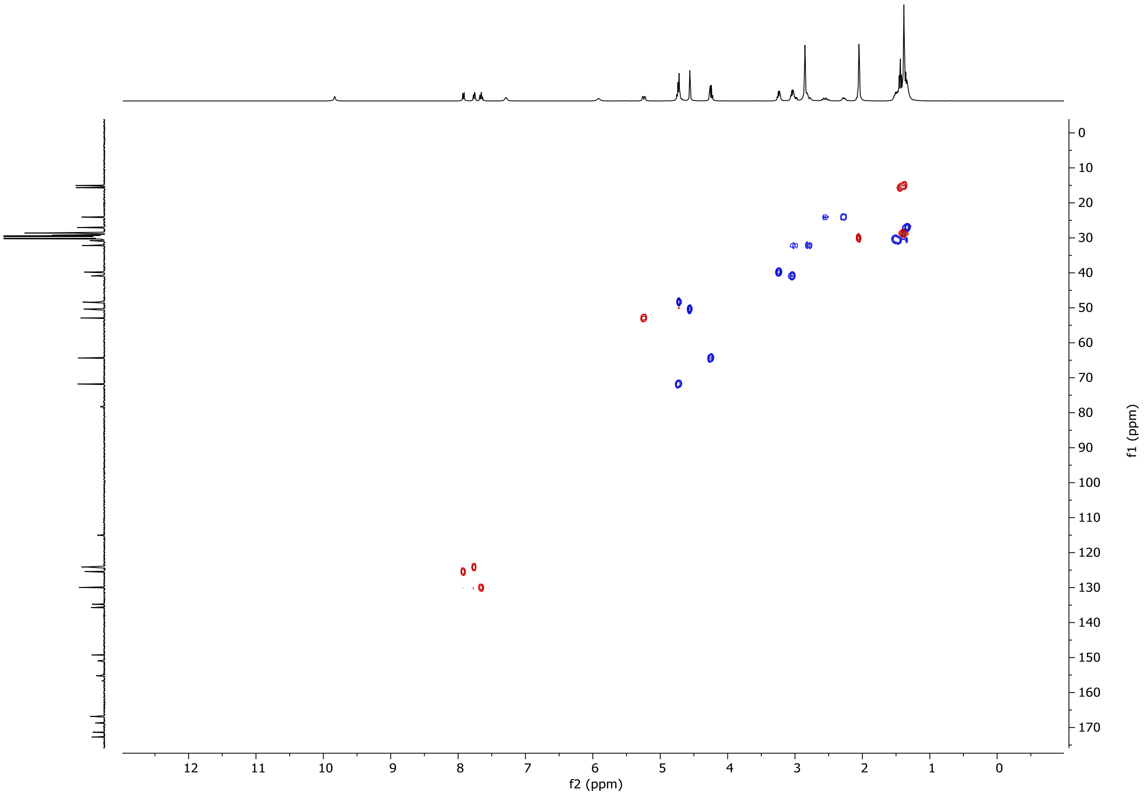

HMBC (400/101 MHz, $(CD_3)_2CO$) of compound **68**:

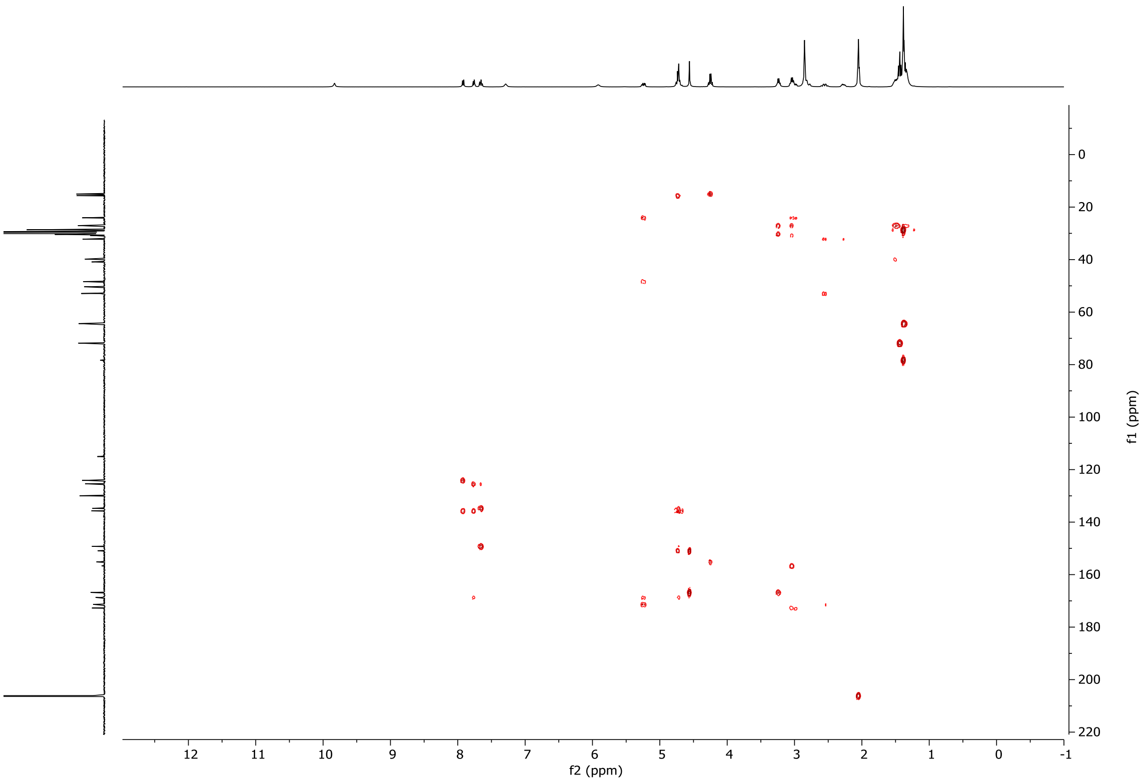

COSY (400 MHz, $(CD_3)_2CO$) of compound **68**:

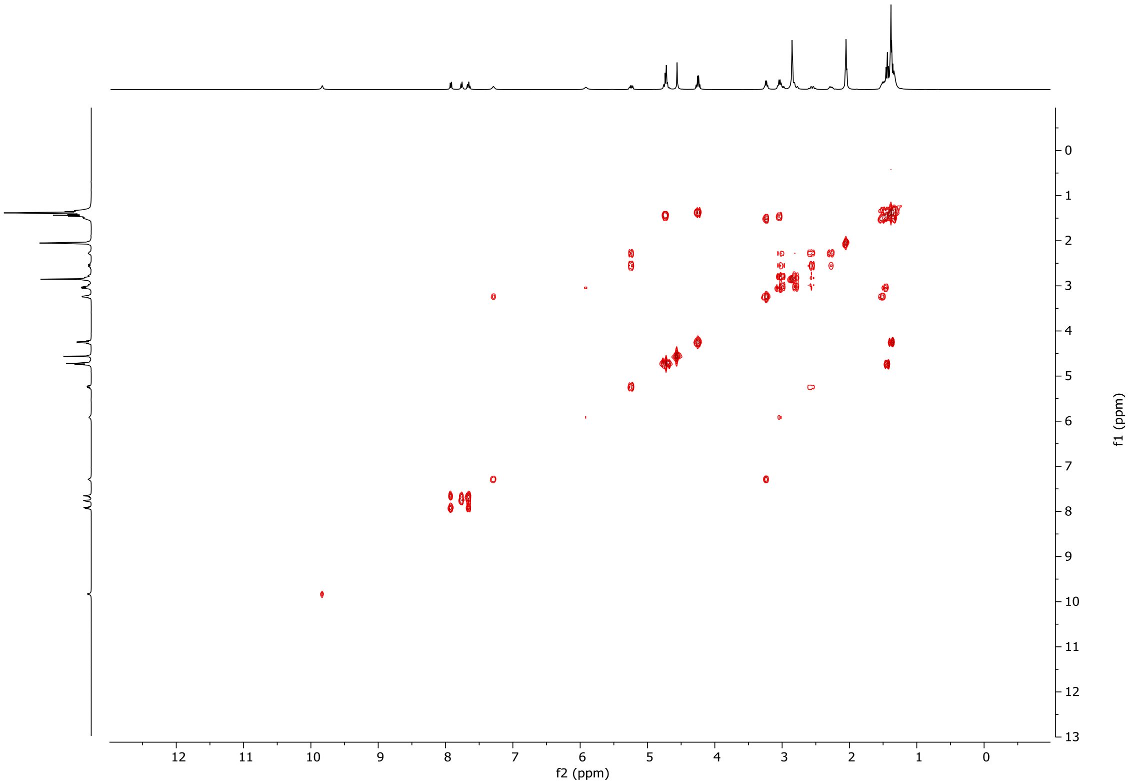

^1H NMR (400 MHz, $(CD_3)_2CO$) of compound **69**:

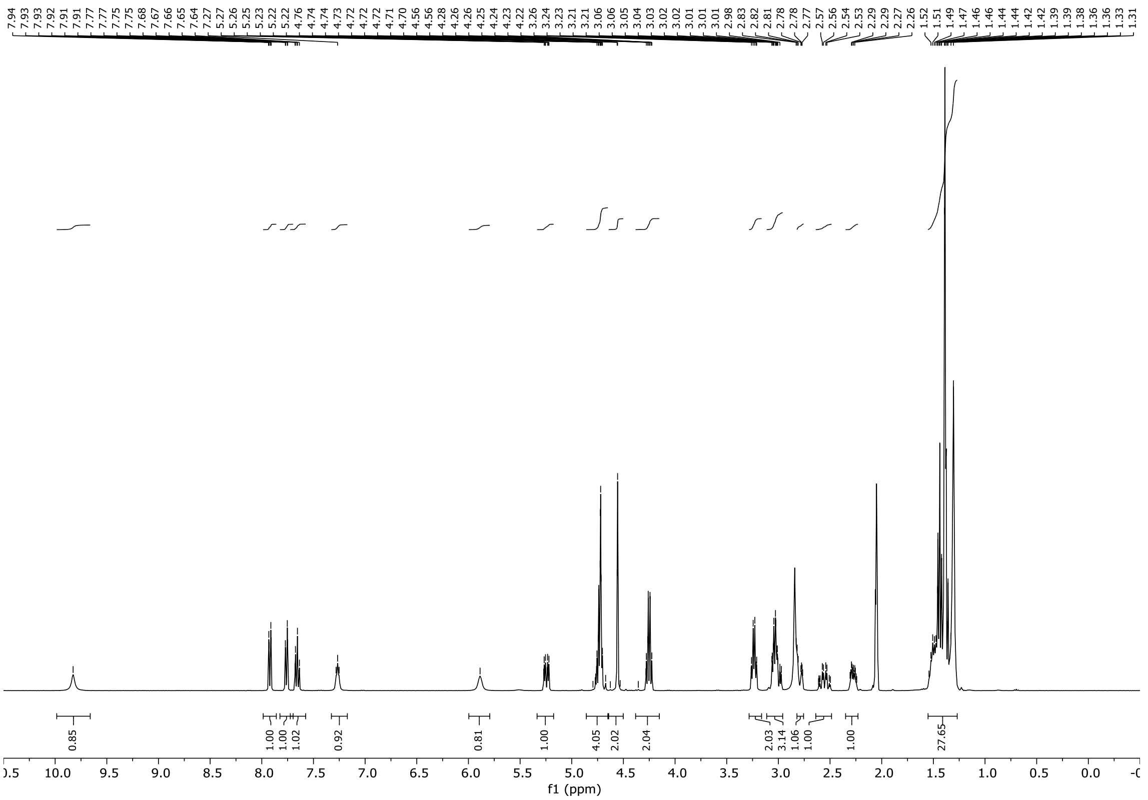

^{13}C NMR (101 MHz, $(CD_3)_2CO$) of compound **69**:

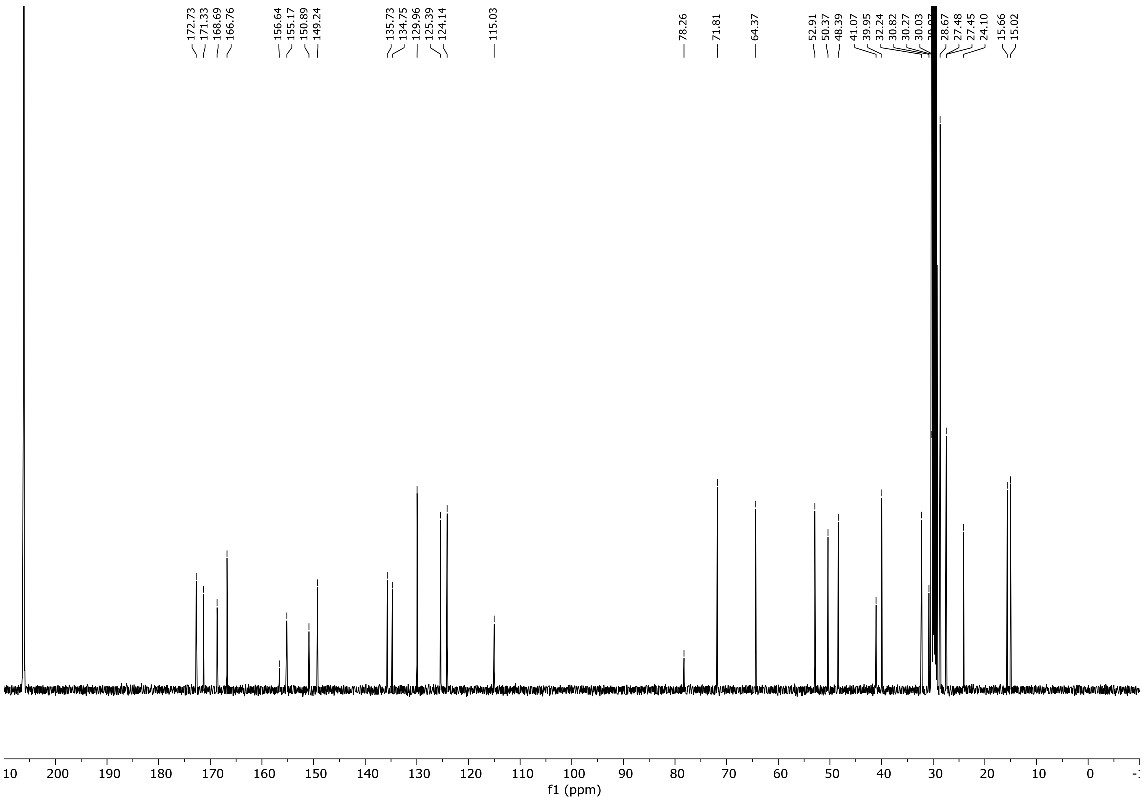

HSQC (400/101 MHz, $(CD_3)_2CO$) of compound **69**:

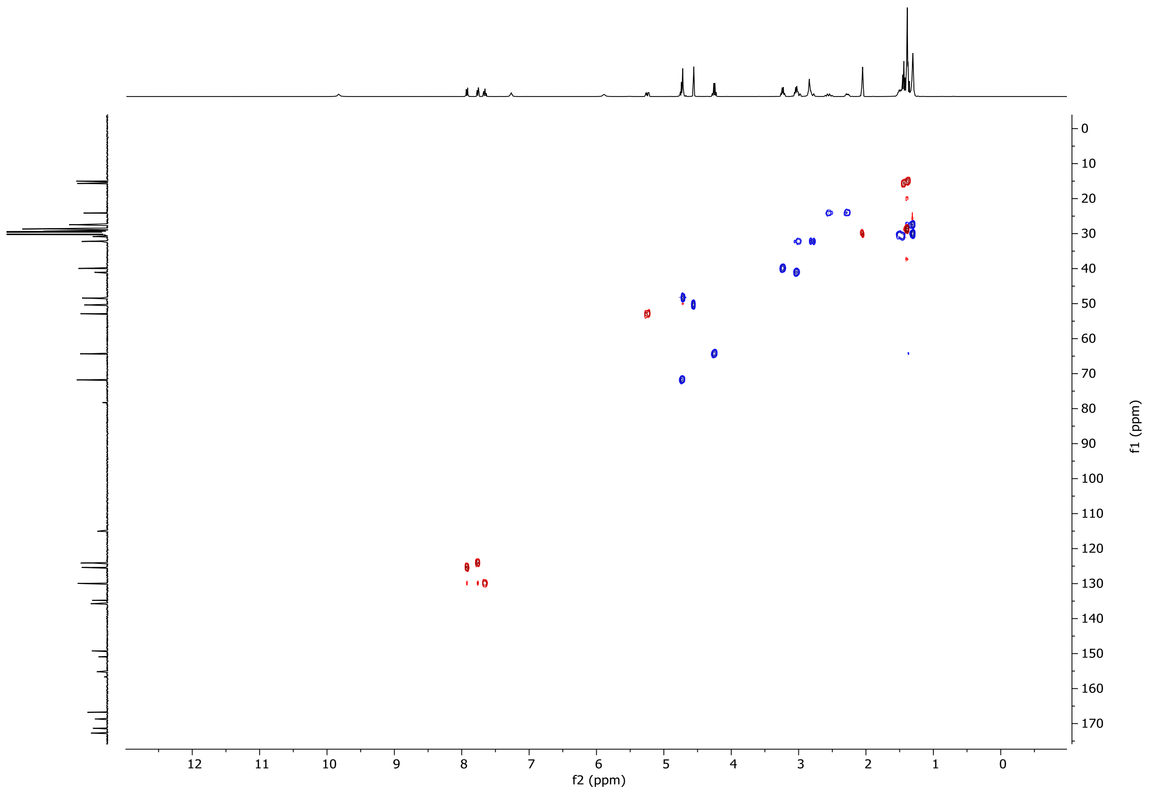

HMBC (400/101 MHz, $(CD_3)_2CO$) of compound **69**:

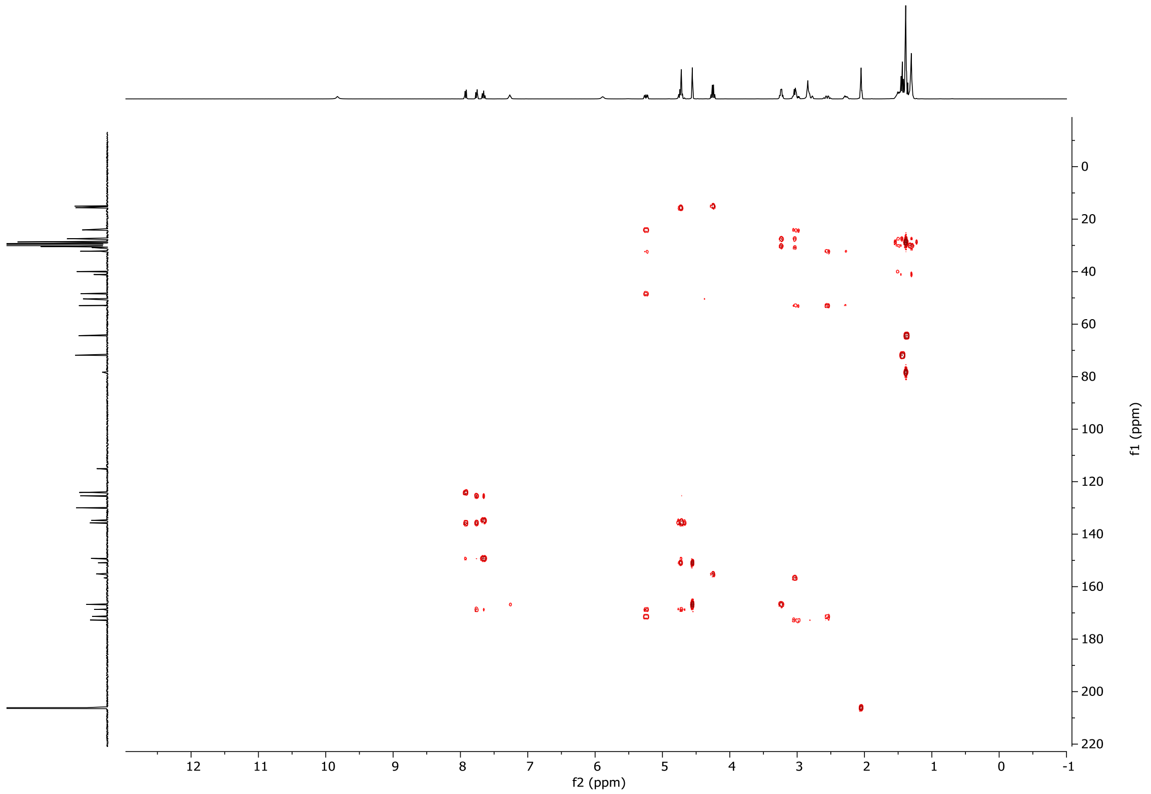

COSY (400 MHz, $(CD_3)_2CO$) of compound **69**:

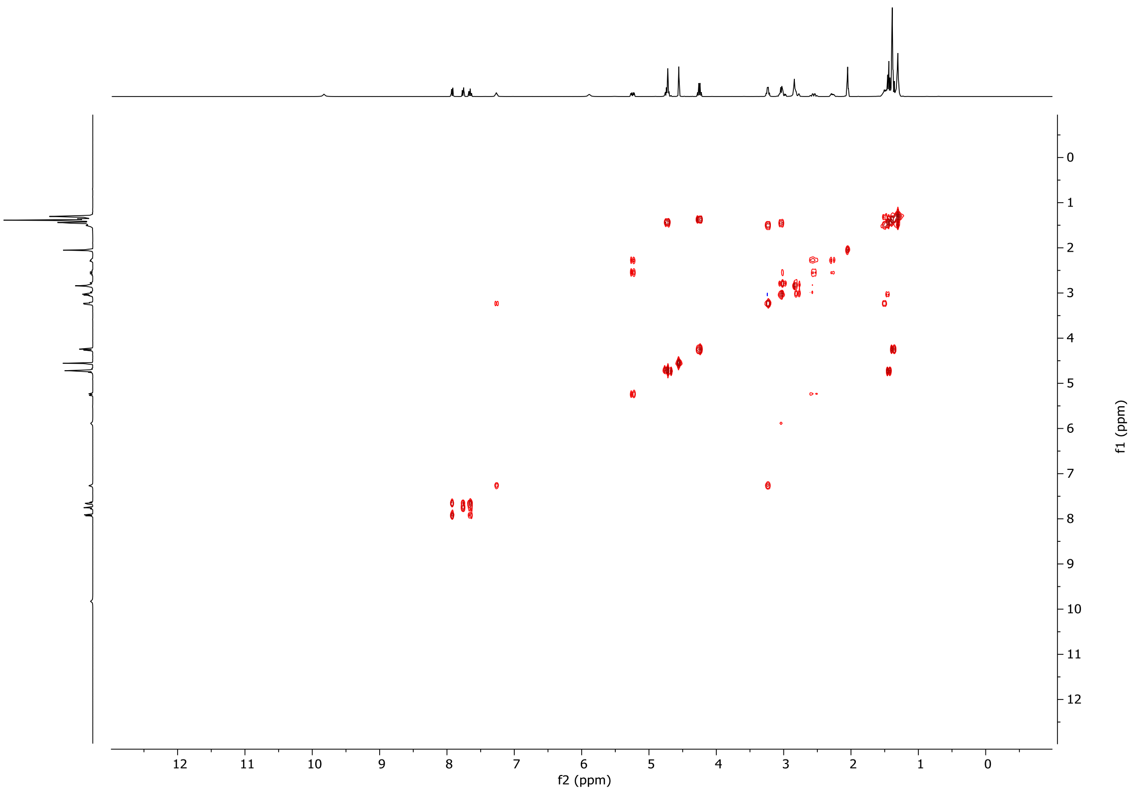

^{1}H NMR (400 MHz, $(CD_3)_2CO$) of compound **70**:

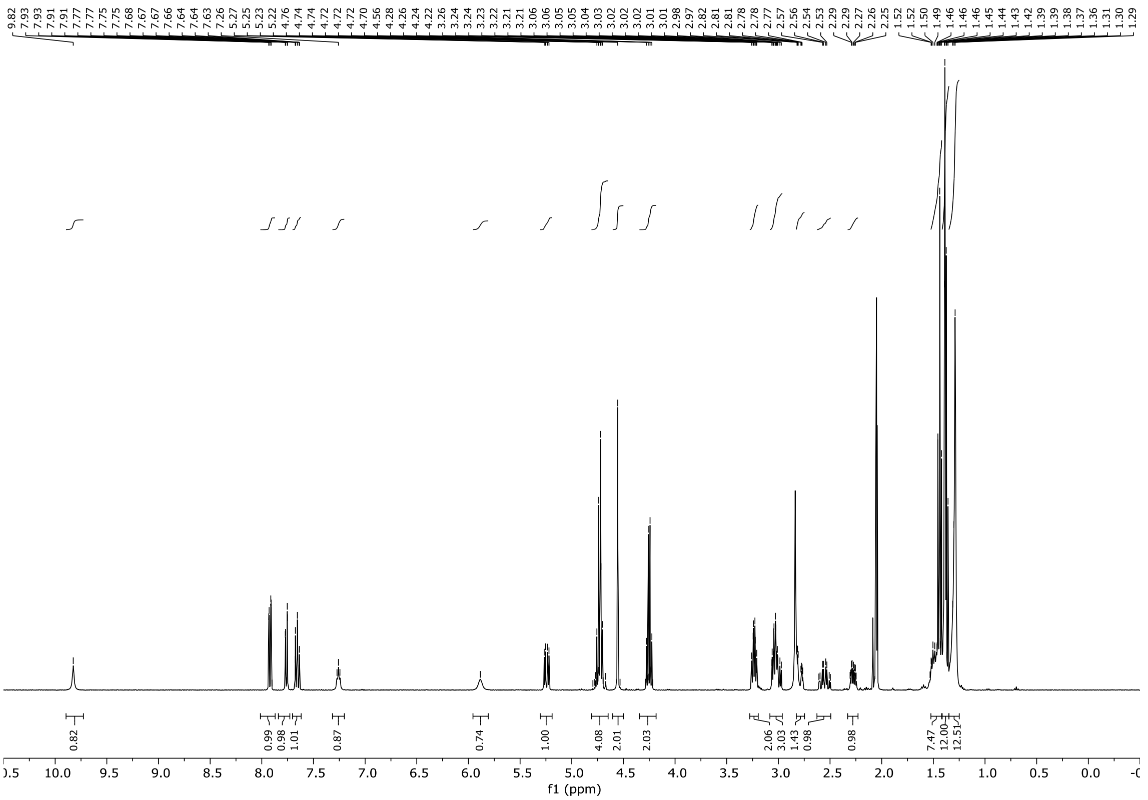

^{13}C NMR (101 MHz, $(CD_3)_2CO$) of compound **70**:

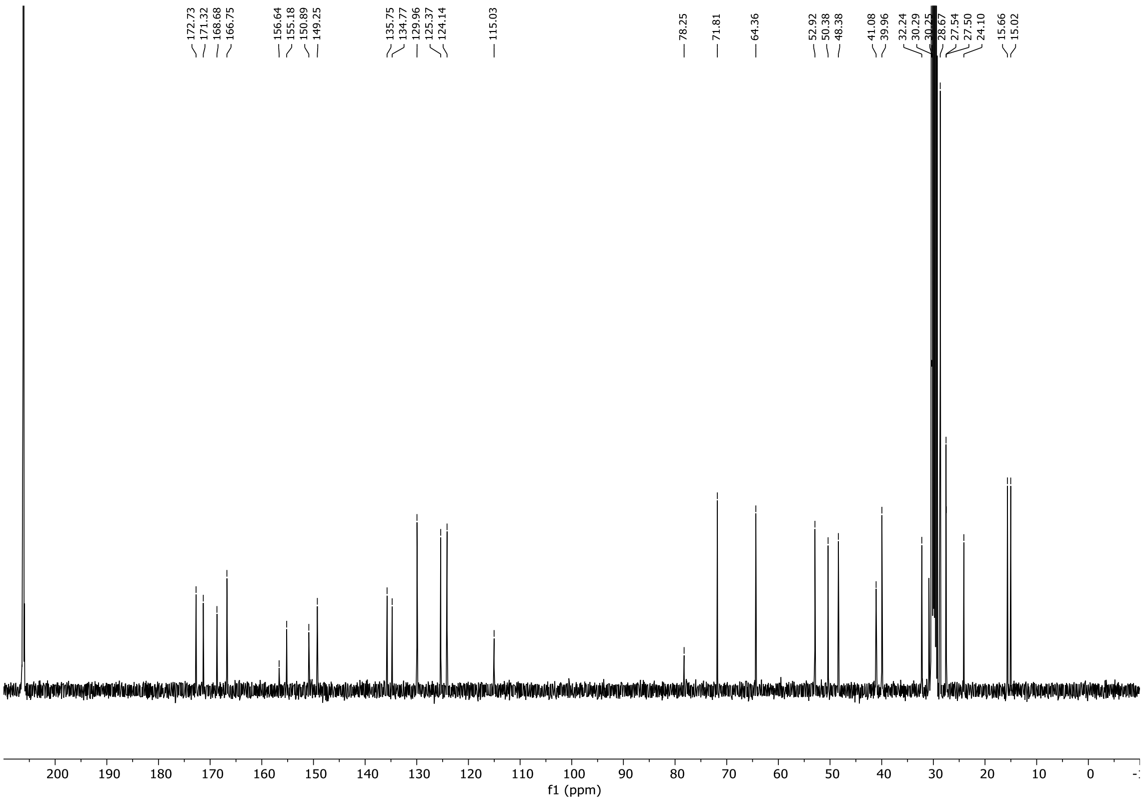

HSQC (400/101 MHz, $(CD_3)_2CO$) of compound **70**:

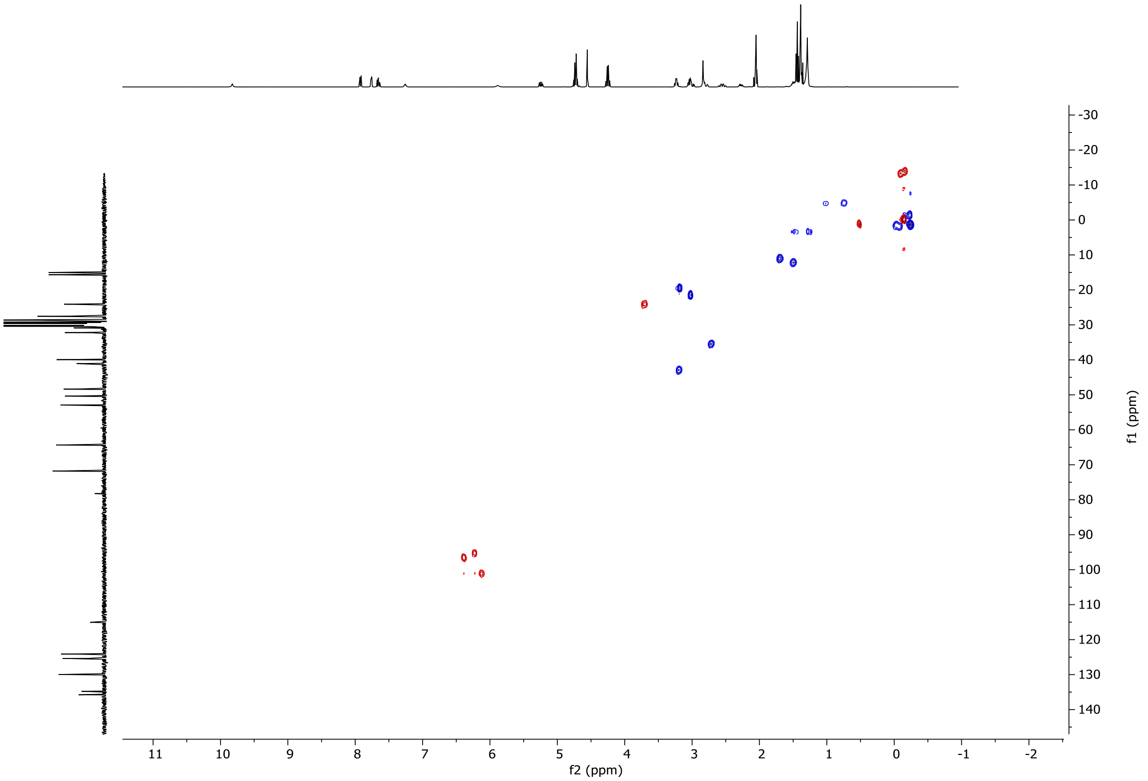

HMBC (400/101 MHz, $(CD_3)_2CO$) of compound **70**:

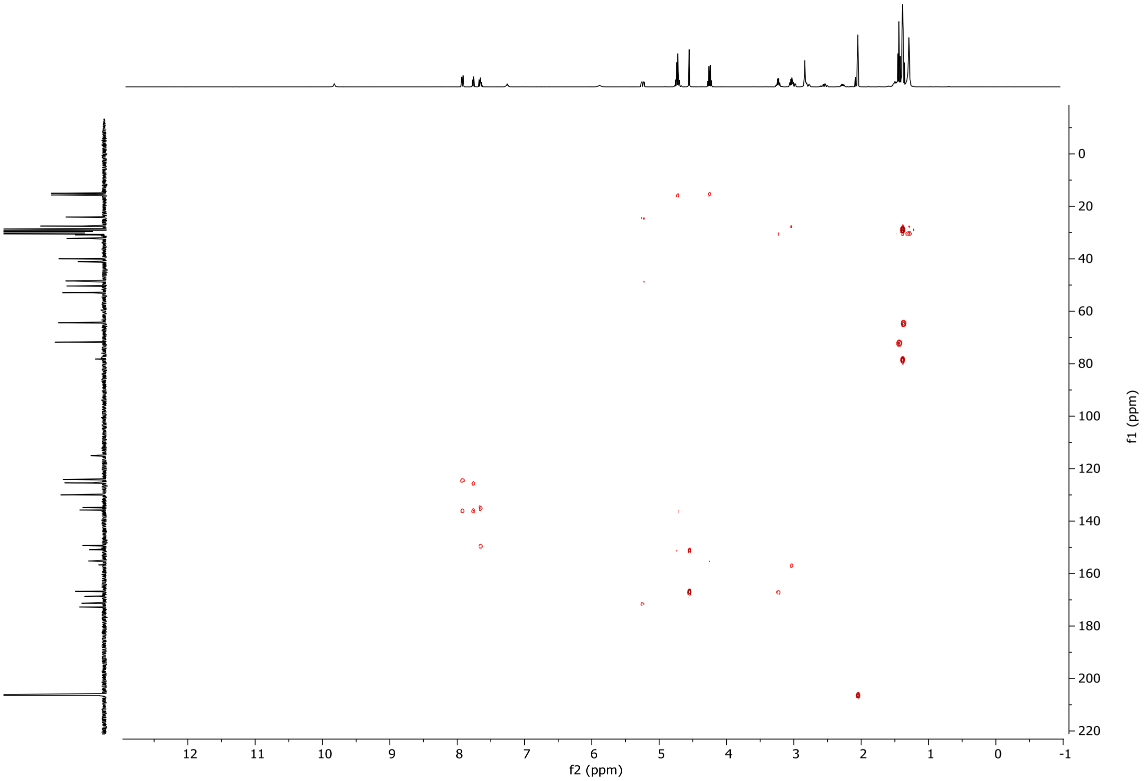

COSY (400 MHz, $(CD_3)_2CO$) of compound **70**:

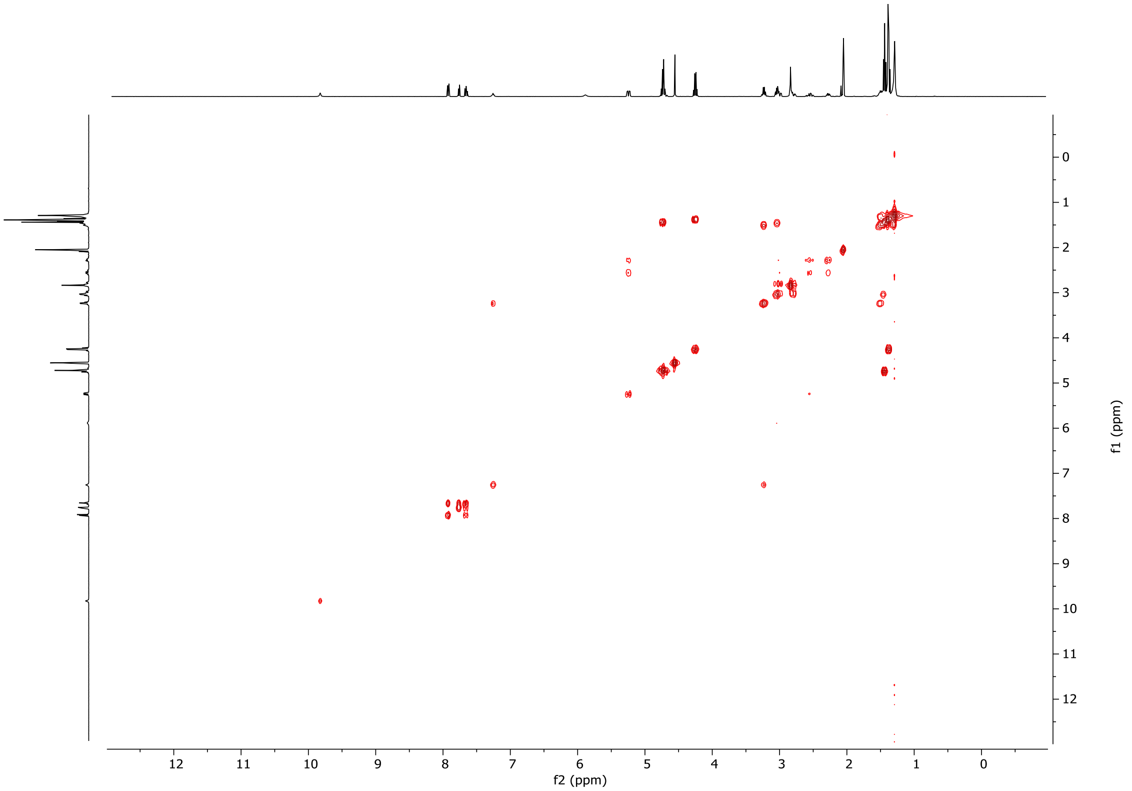

^1H NMR (500 MHz, $(CD_3)_2CO$) of compound **KH-5-450**:

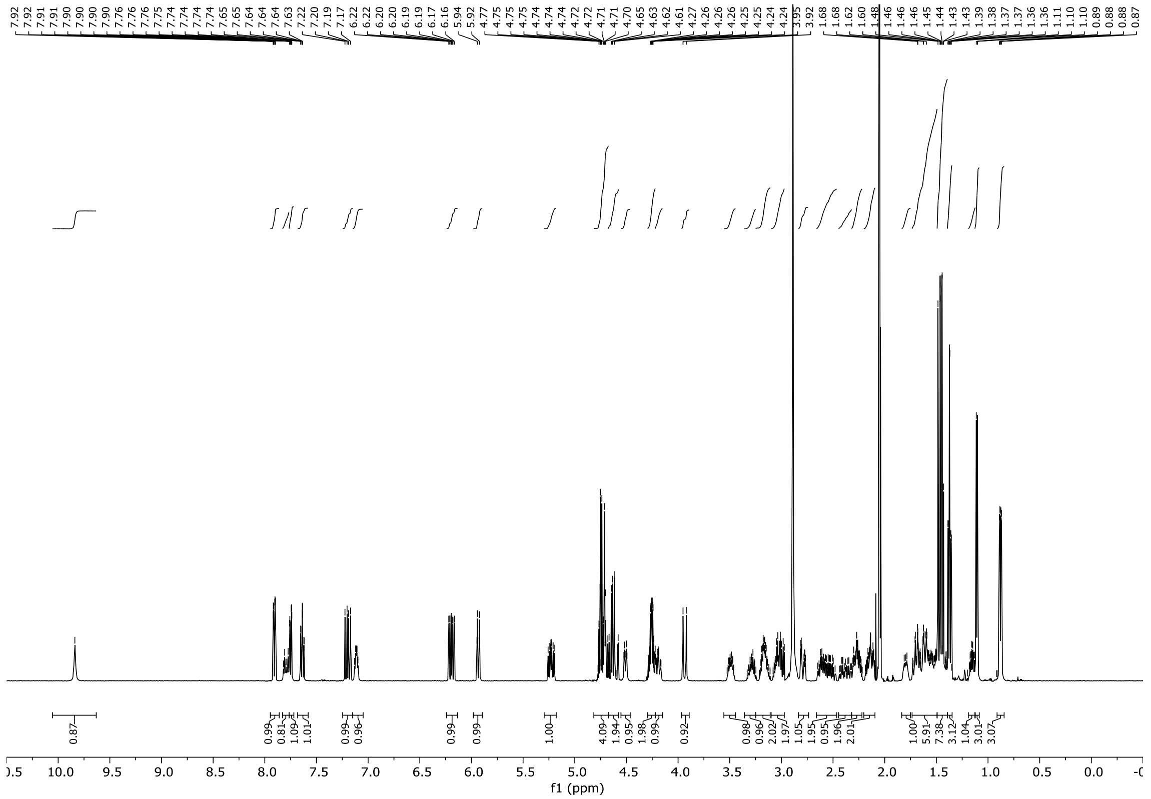

^{13}C NMR (126 MHz, $(CD_3)_2CO$) of compound **KH-5-450**:

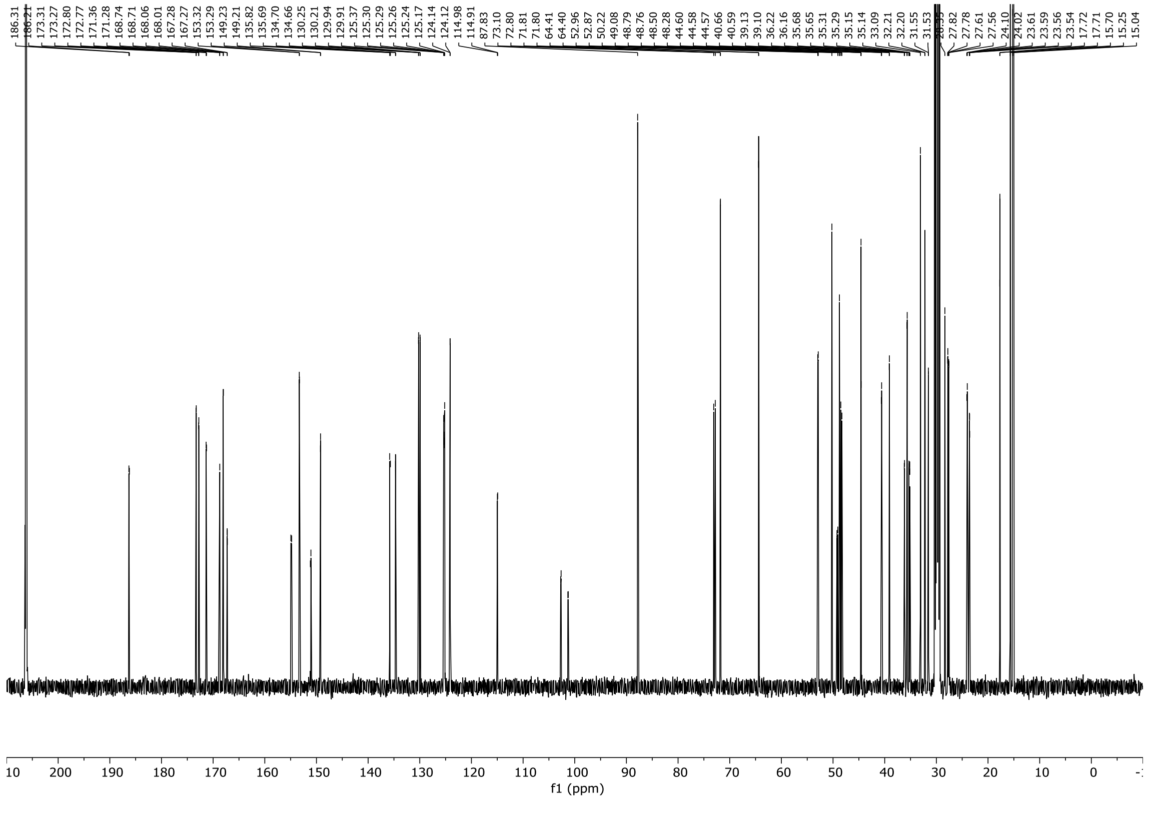

HSQC (400/101 MHz, $(CD_3)_2CO$) of compound **KH-5-450**:

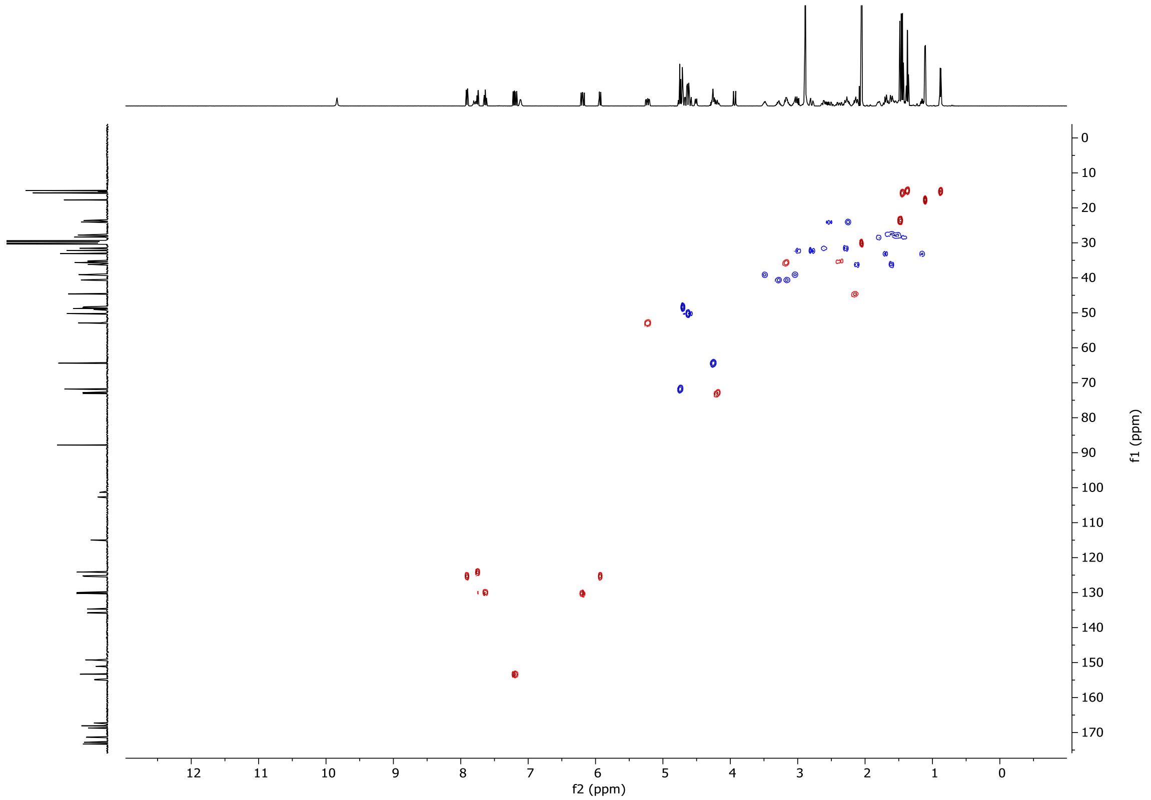

HMBC (400/101 MHz, $(CD_3)_2CO$) of compound **KH-5-450**:

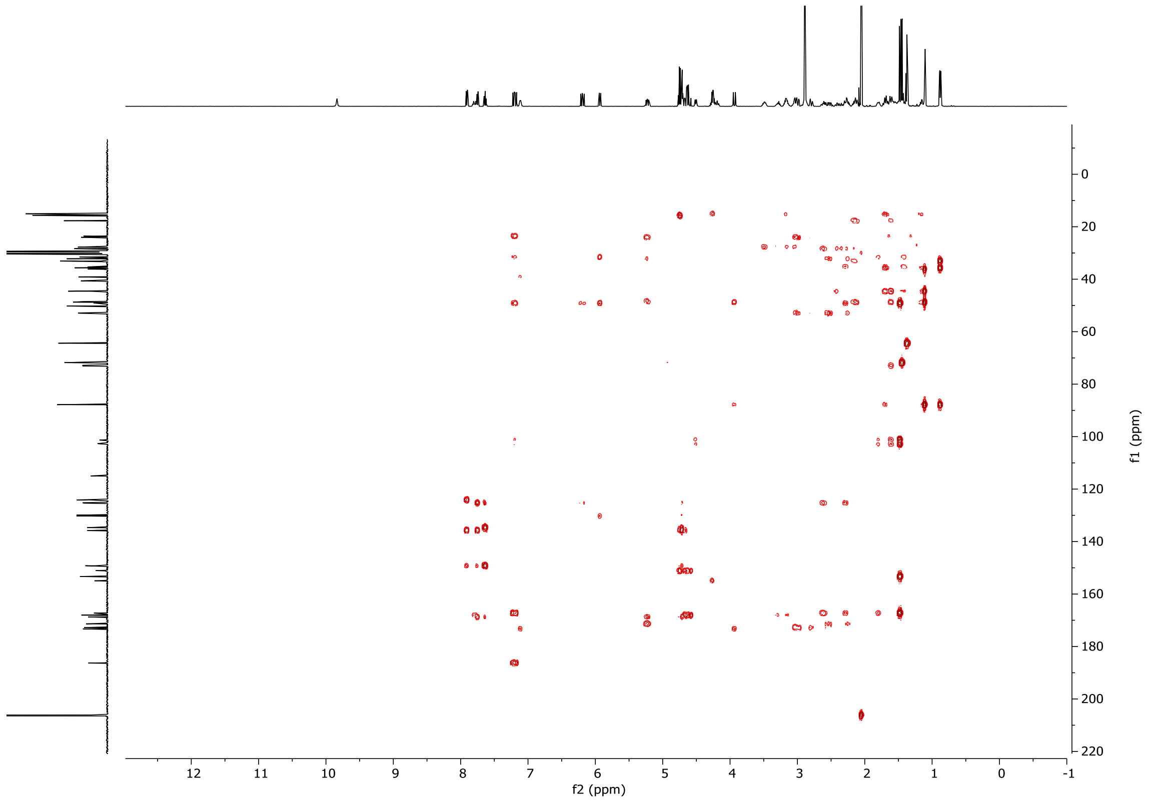

COSY (400 MHz, $(CD_3)_2CO$) of compound **KH-5-450**:

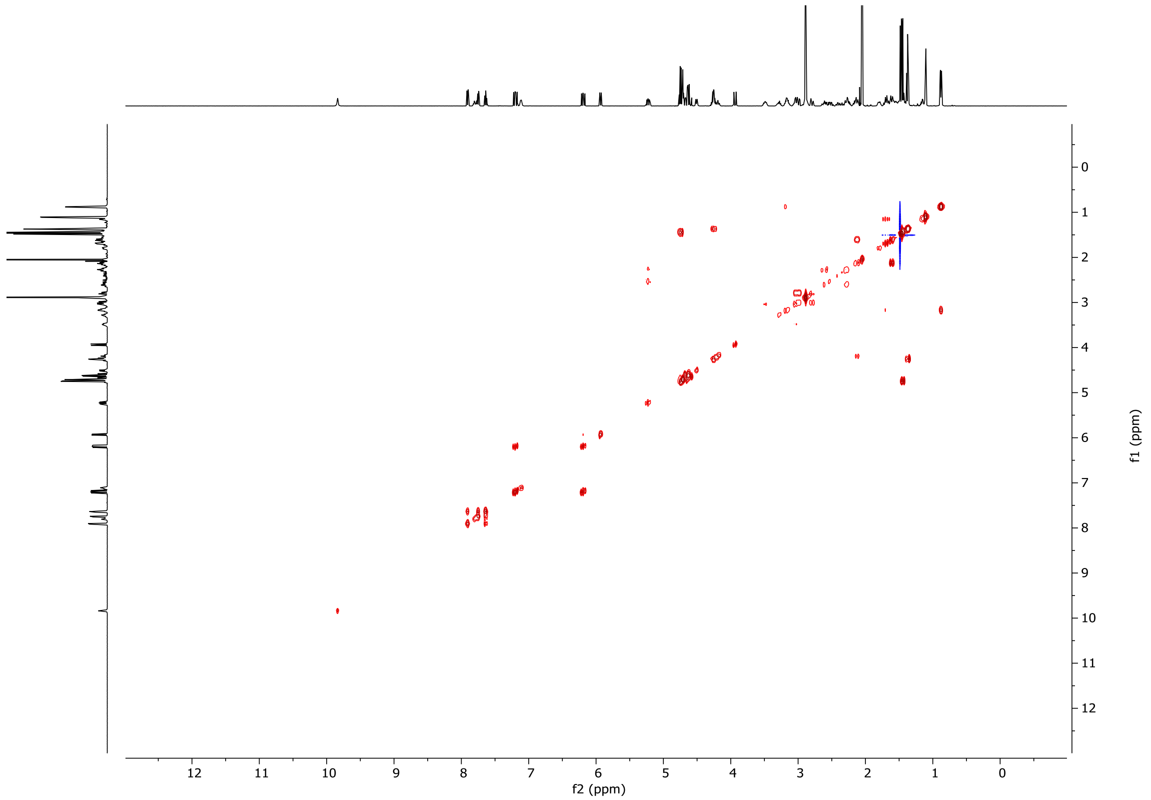

^1H NMR (500 MHz, $(CD_3)_2CO$) of compound **KH-5-452**:

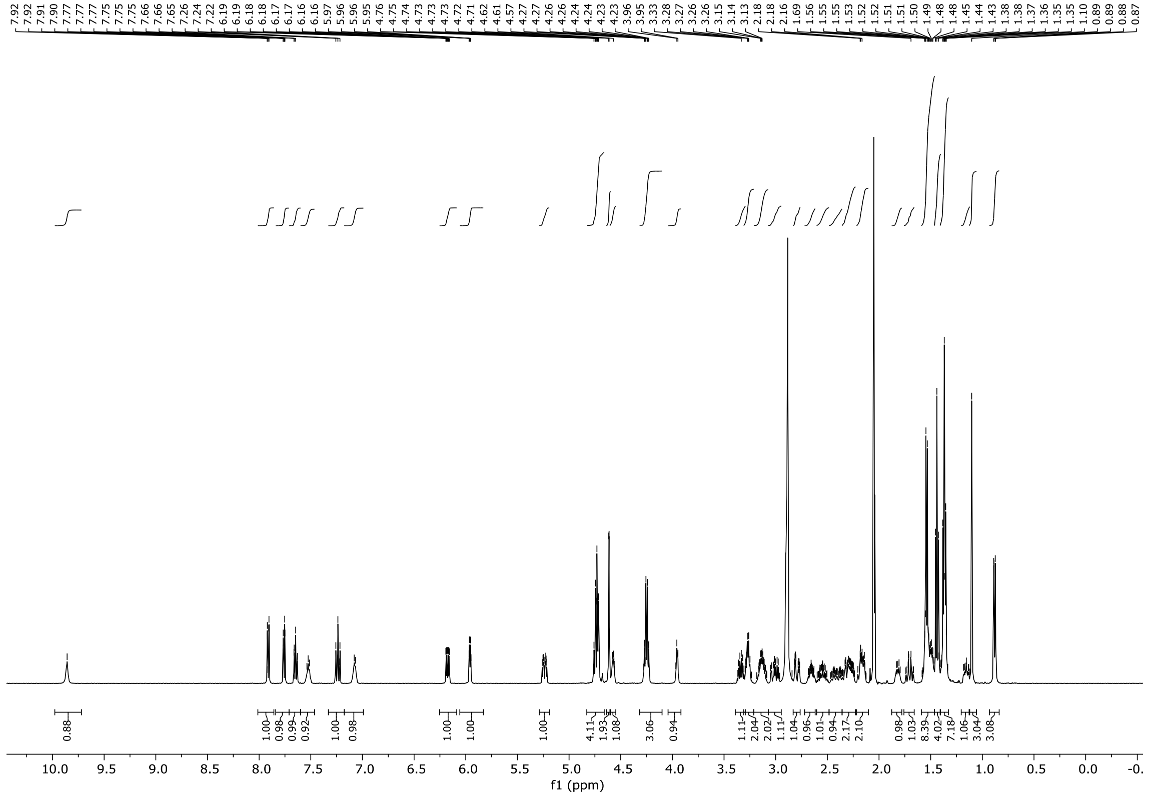

^{13}C NMR (126 MHz, $(CD_3)_2CO$) of compound **KH-5-452**:

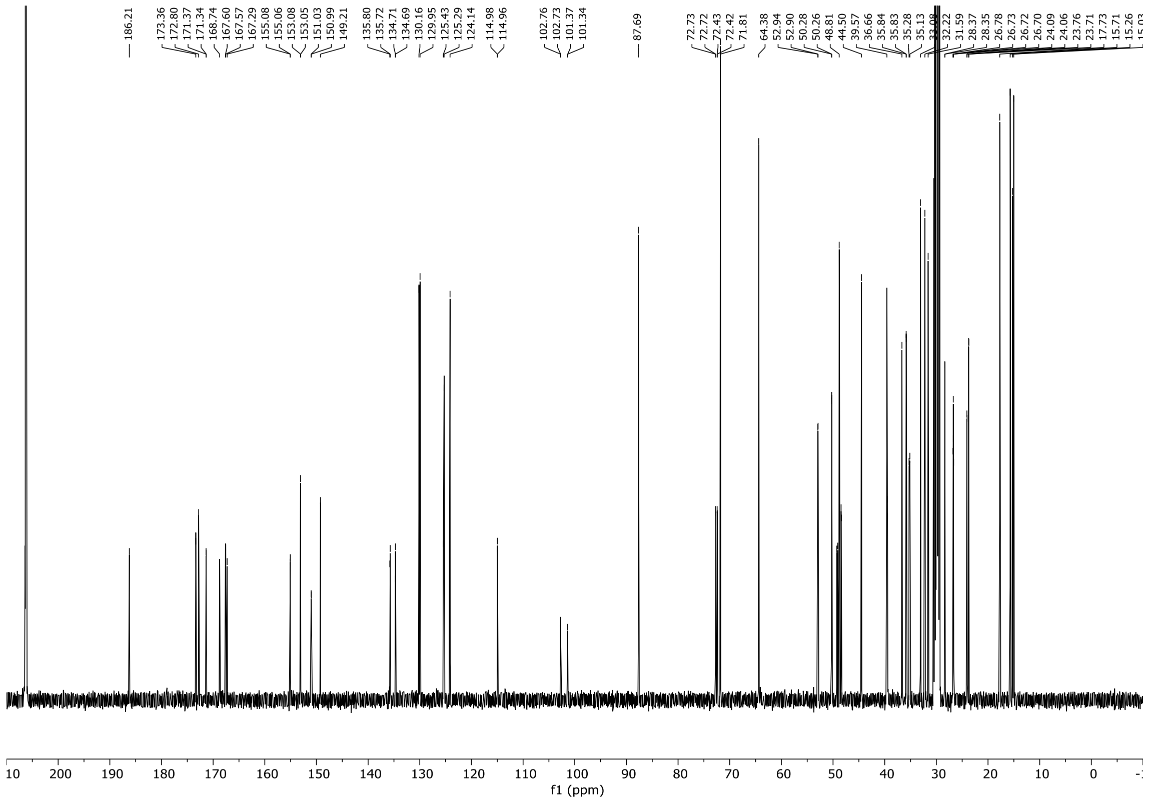

HSQC (400/101 MHz, $(CD_3)_2CO$) of compound **KH-5-452**:

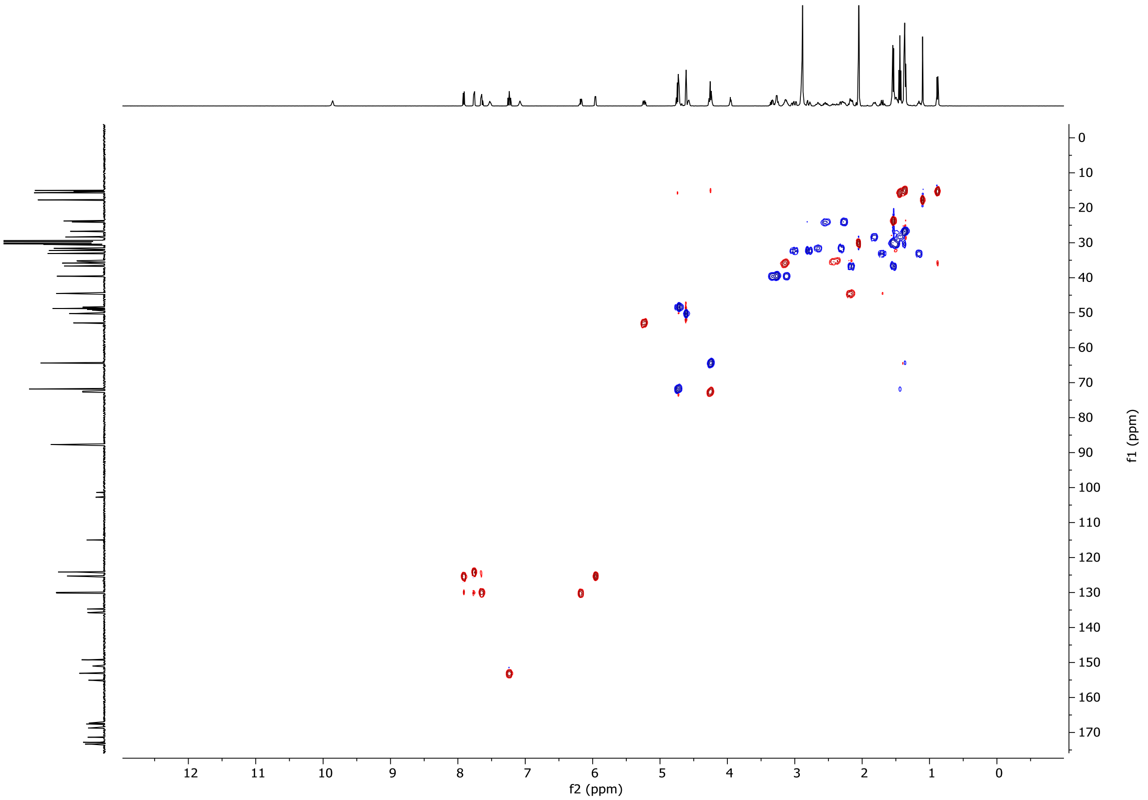

HMBC (400/101 MHz, $(CD_3)_2CO$) of compound **KH-5-452**:

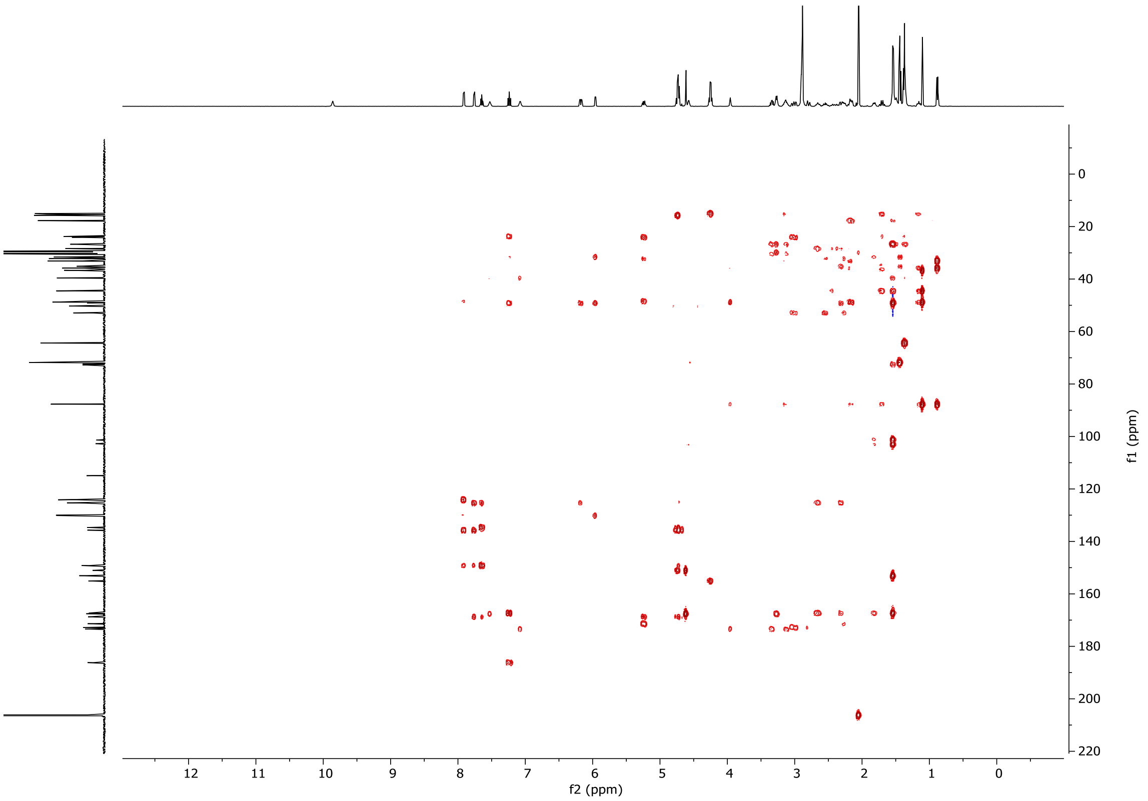

COSY (400 MHz, $(CD_3)_2CO$) of compound **KH-5-452**:

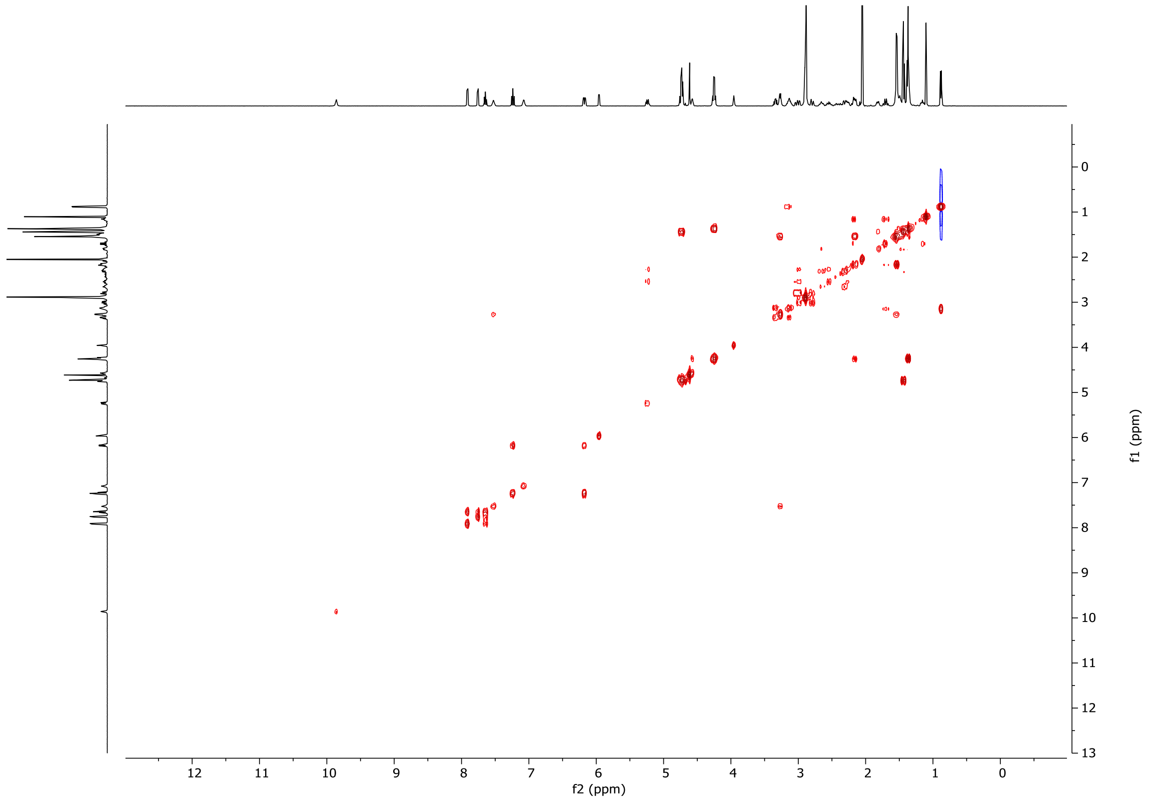

^{1}H NMR (500 MHz, $(CD_3)_2CO$) of compound **KH-5-454**:

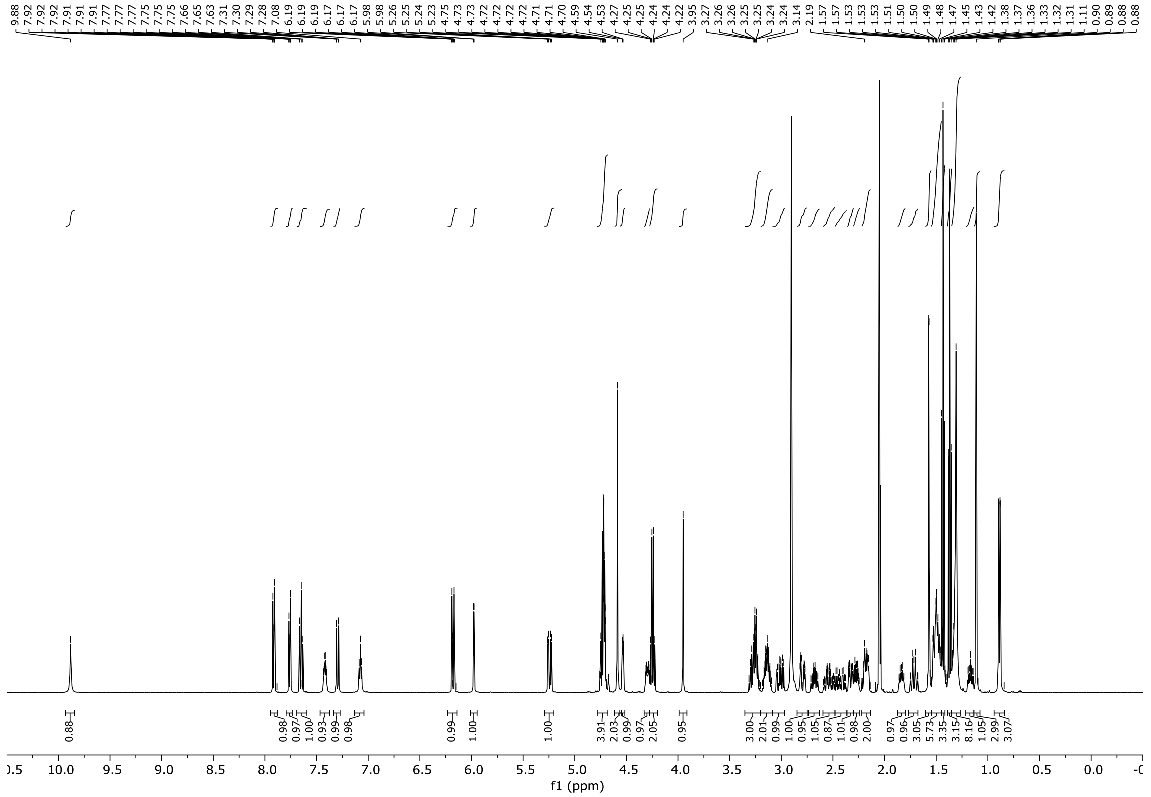

^{13}C NMR (126 MHz, $(CD_3)_2CO$) of compound **KH-5-454**:

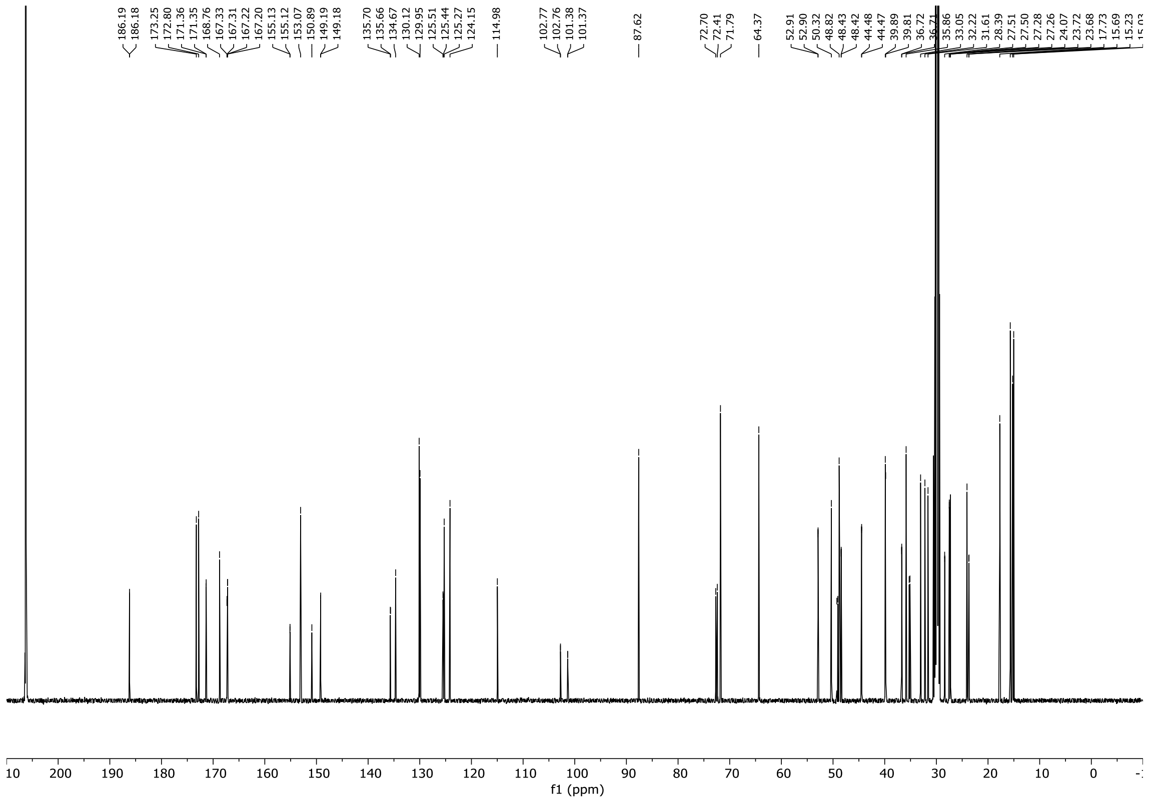

HSQC (400/101 MHz, $(CD_3)_2CO$) of compound **KH-5-454**:

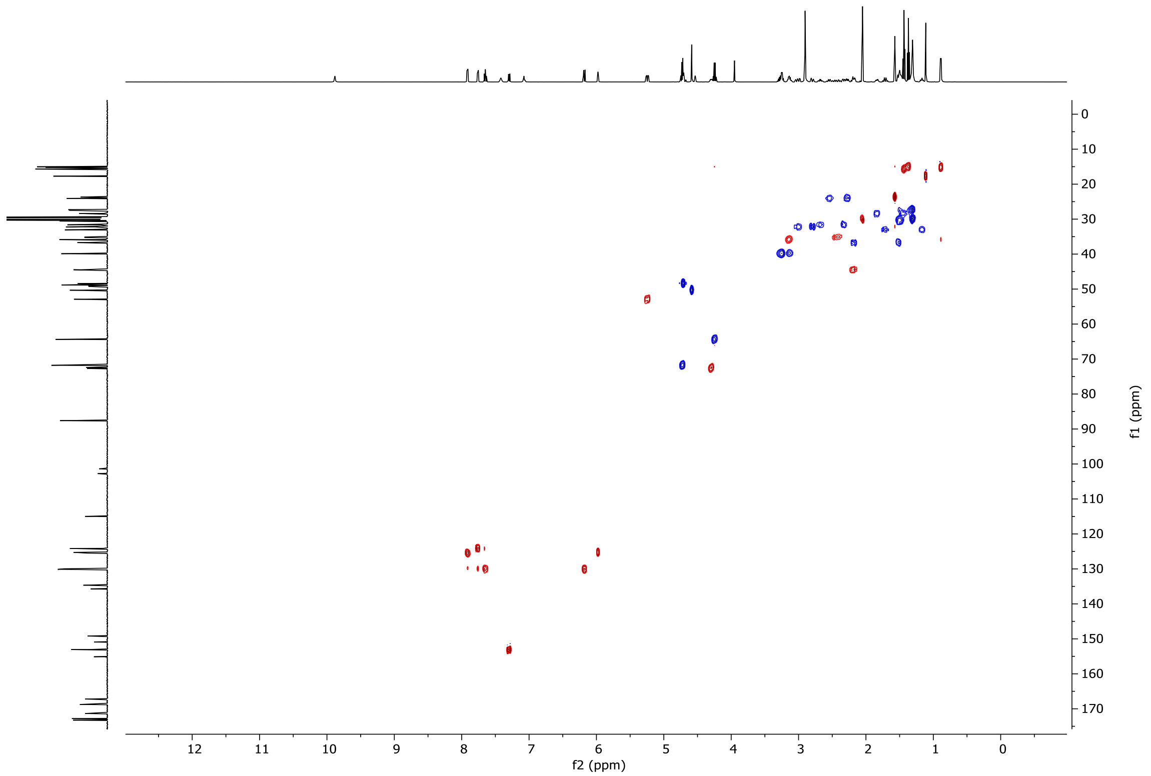

HMBC (400/101 MHz, $(CD_3)_2CO$) of compound **KH-5-454**:

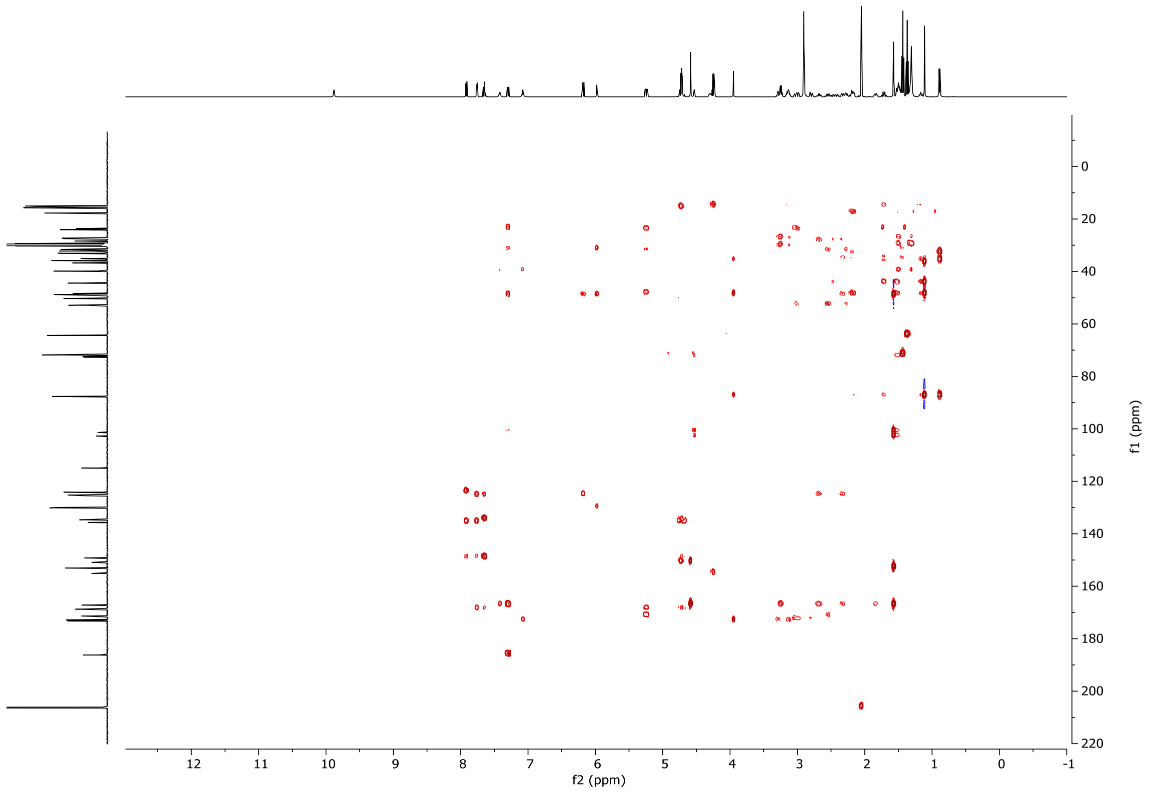

COSY (400 MHz, $(CD_3)_2CO$) of compound **KH-5-454**:

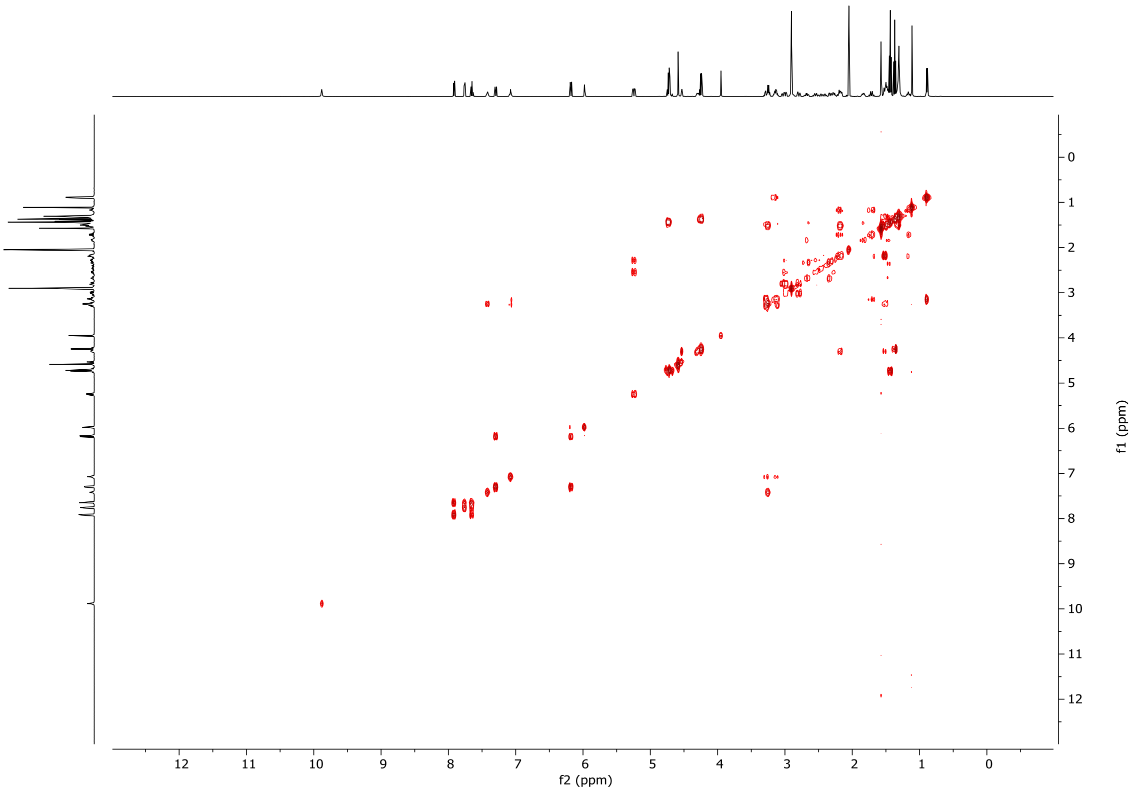

^{1}H NMR (500 MHz, $(CD_3)_2CO$) of compound **KH-5-456**:

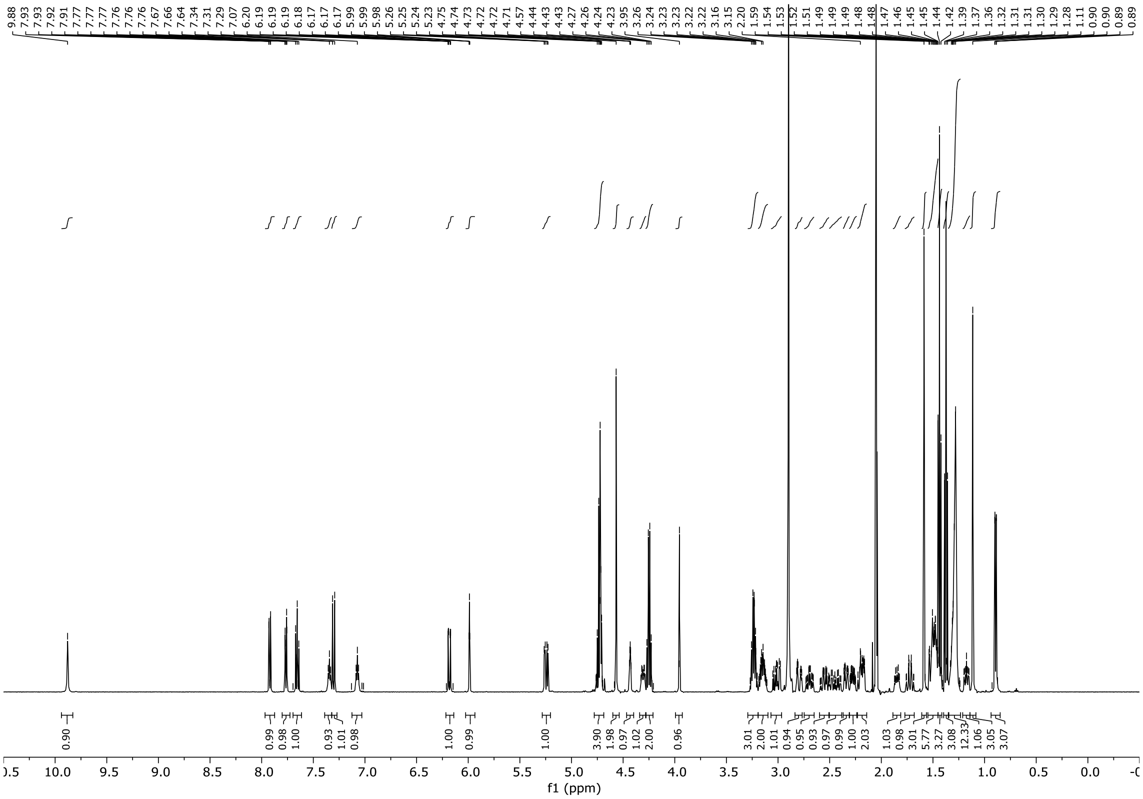

^{13}C NMR (126 MHz, $(CD_3)_2CO$) of compound **KH-5-456**:

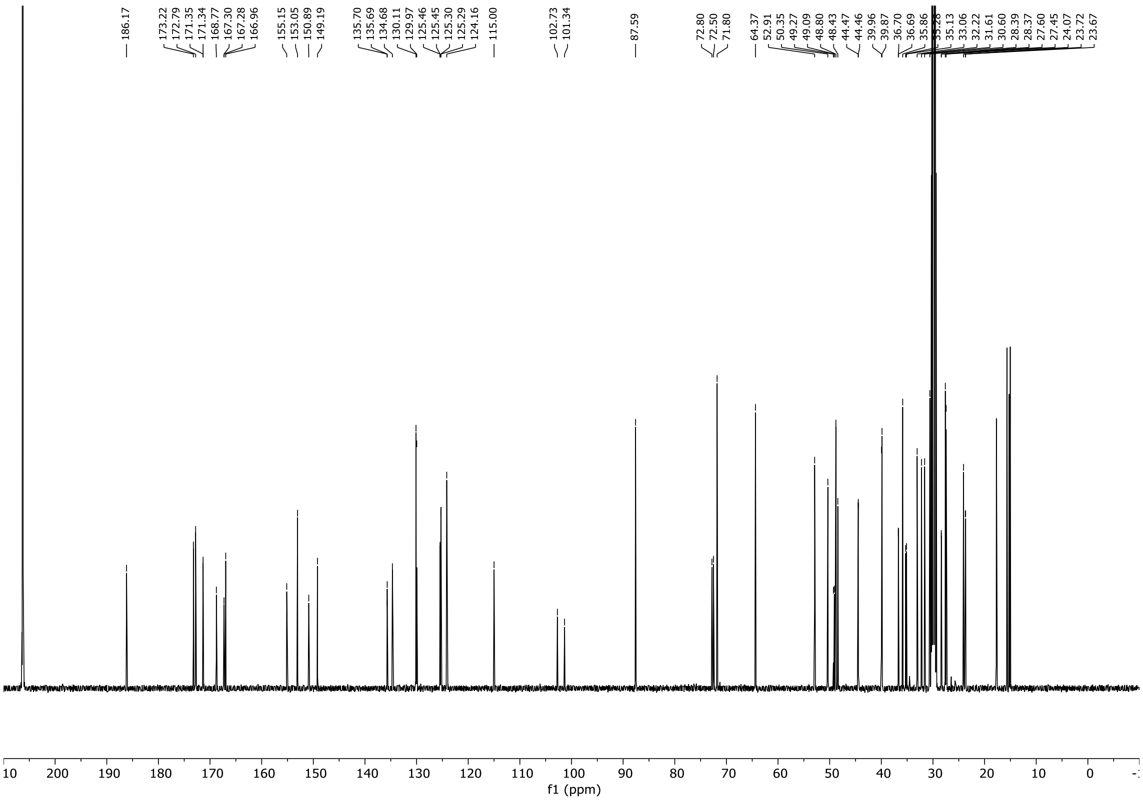

HSQC (400/101 MHz, $(CD_3)_2CO$) of compound **KH-5-456**:

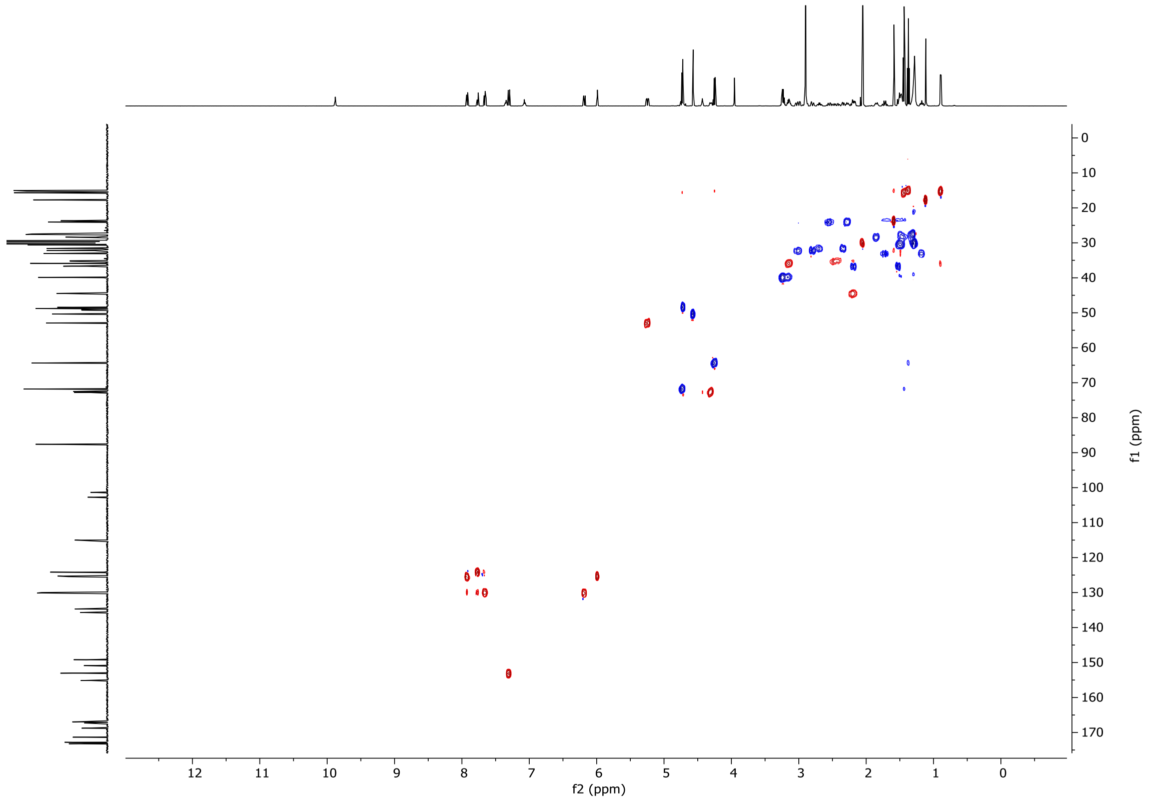

HMBC (400/101 MHz, $(CD_3)_2CO$) of compound **KH-5-456**:

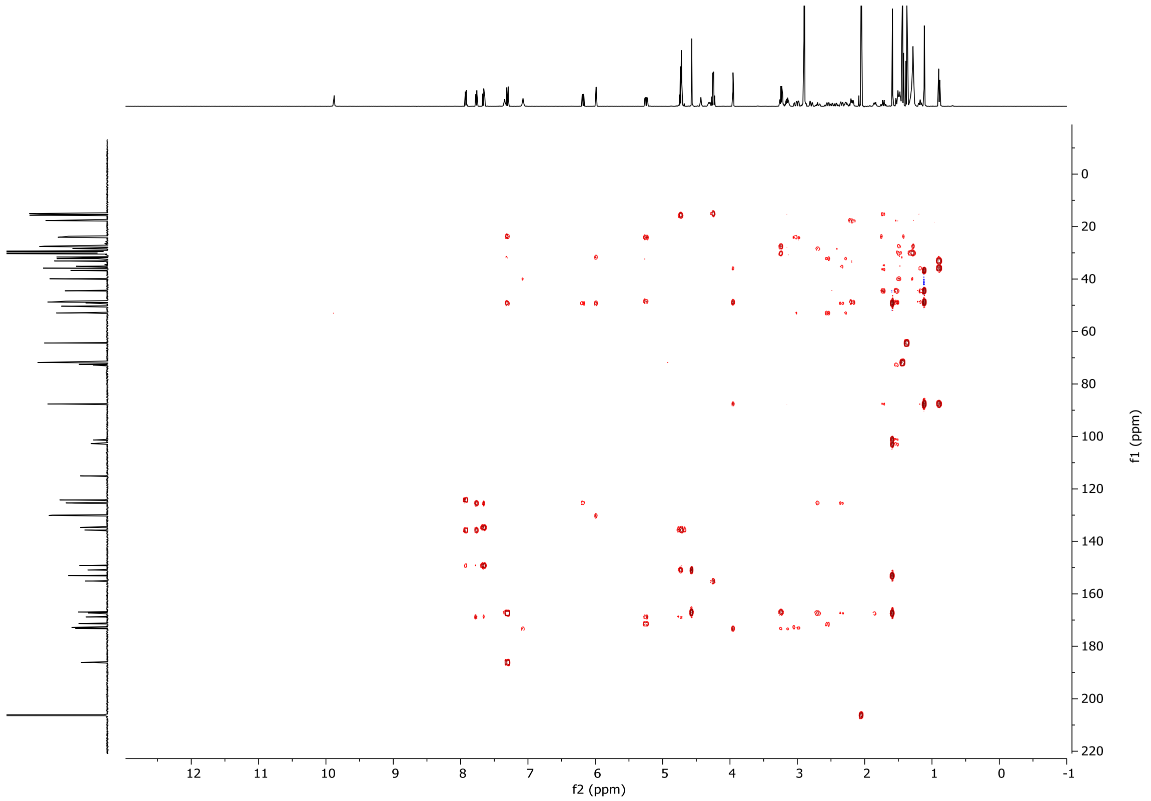

COSY (400 MHz, $(CD_3)_2CO$) of compound **KH-5-456**:

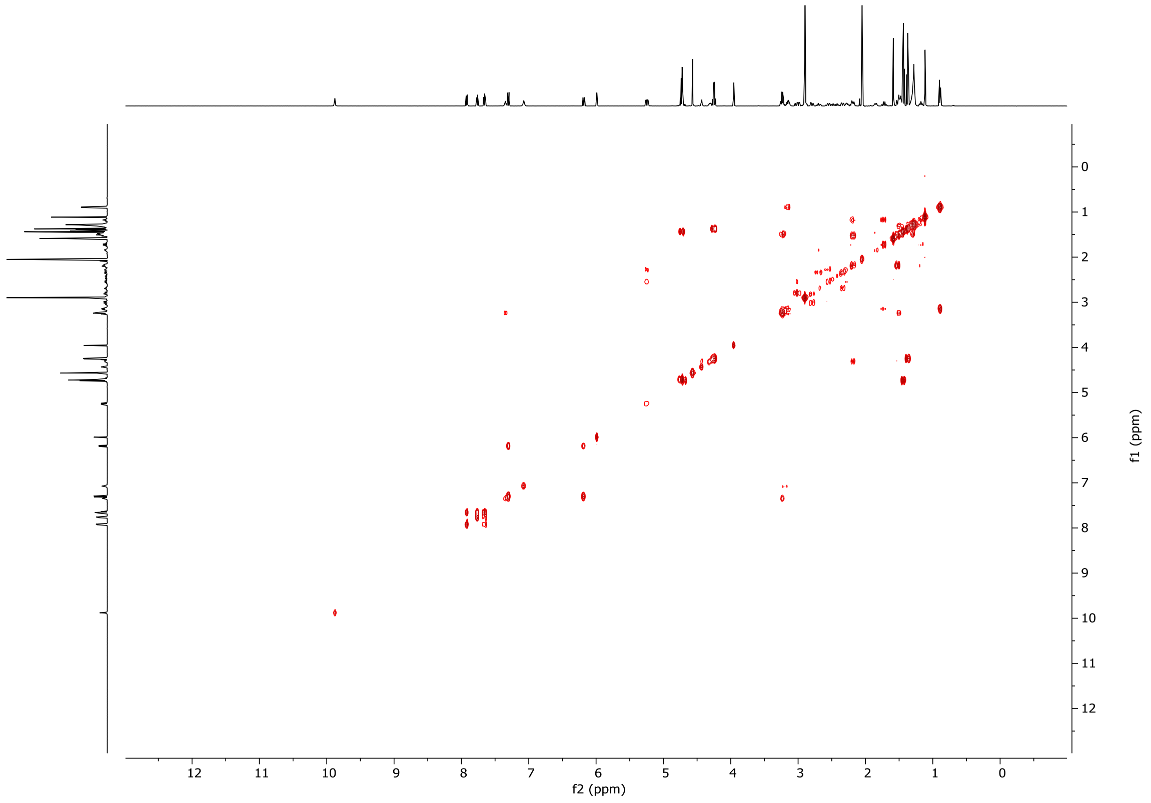

(NMe2)2-Arylazopyrazole Photoswitch

^{1}H NMR (400 MHz, $CDCl_3$) of compound **46**:

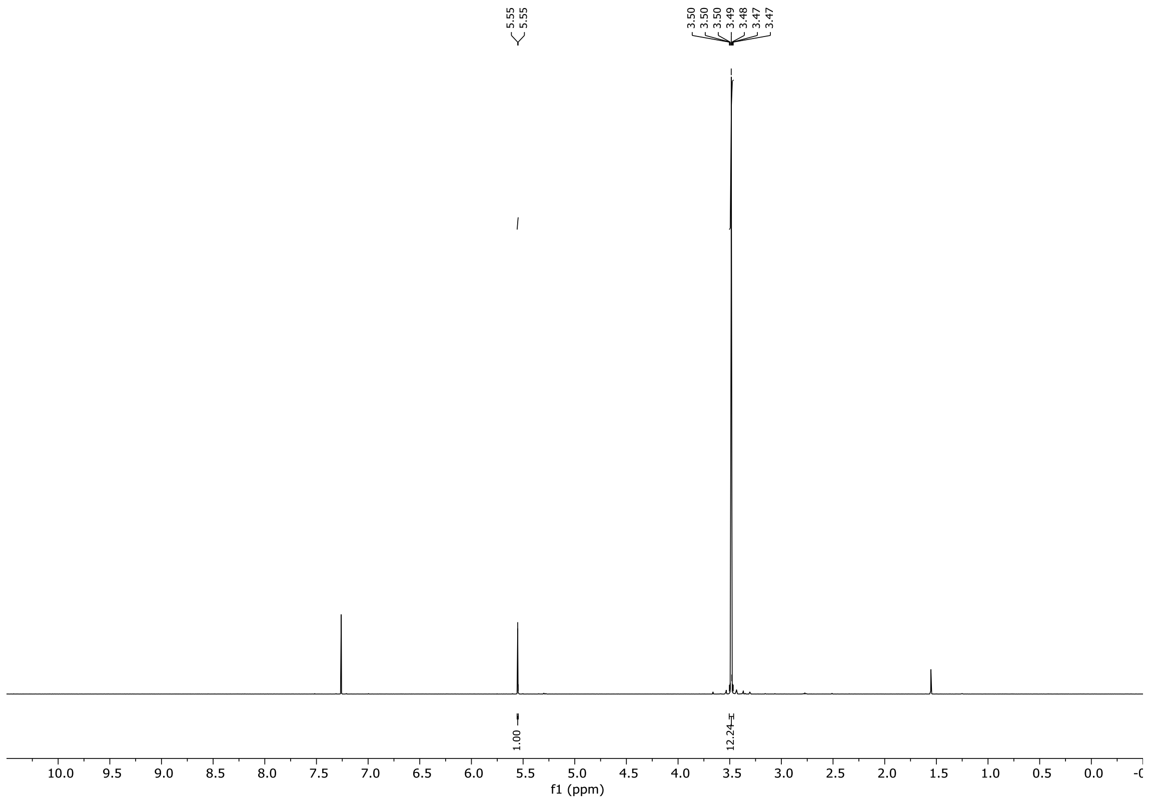

^{13}C NMR (101 MHz, $CDCl_3$) of compound **46**:

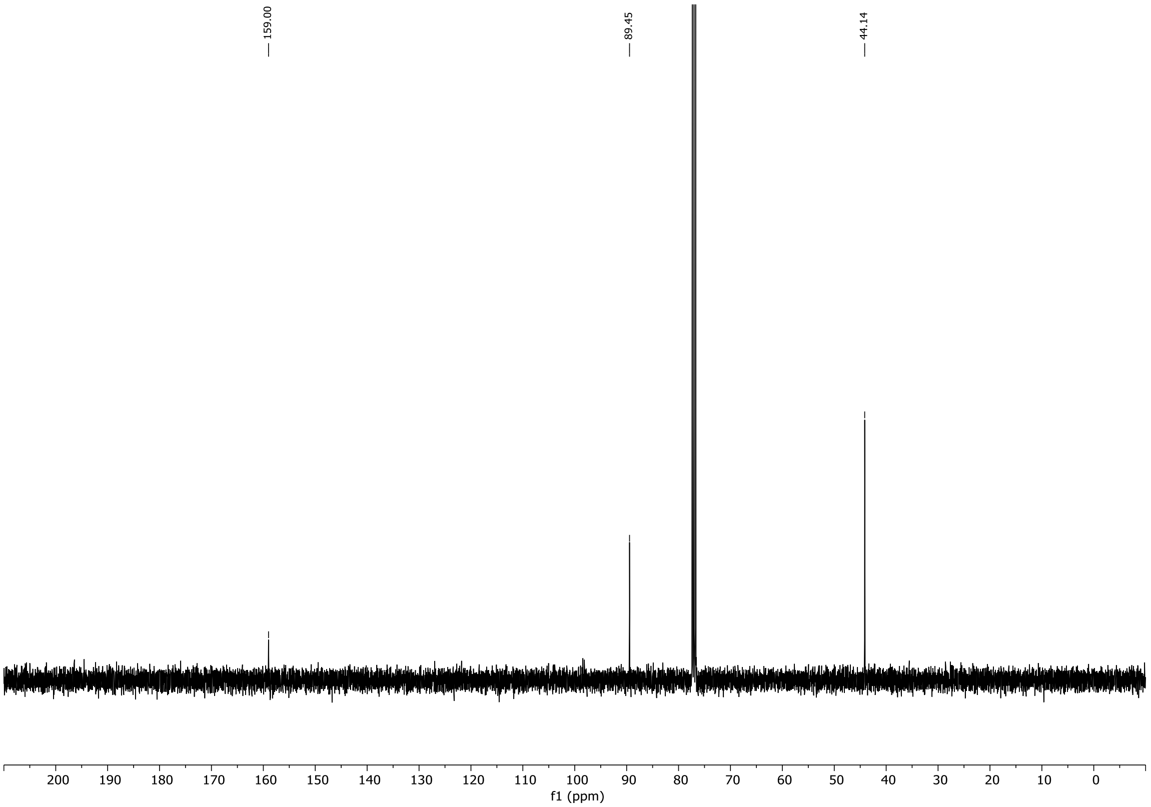

^{19}F NMR (377 MHz, $CDCl_3$) of compound **46**:

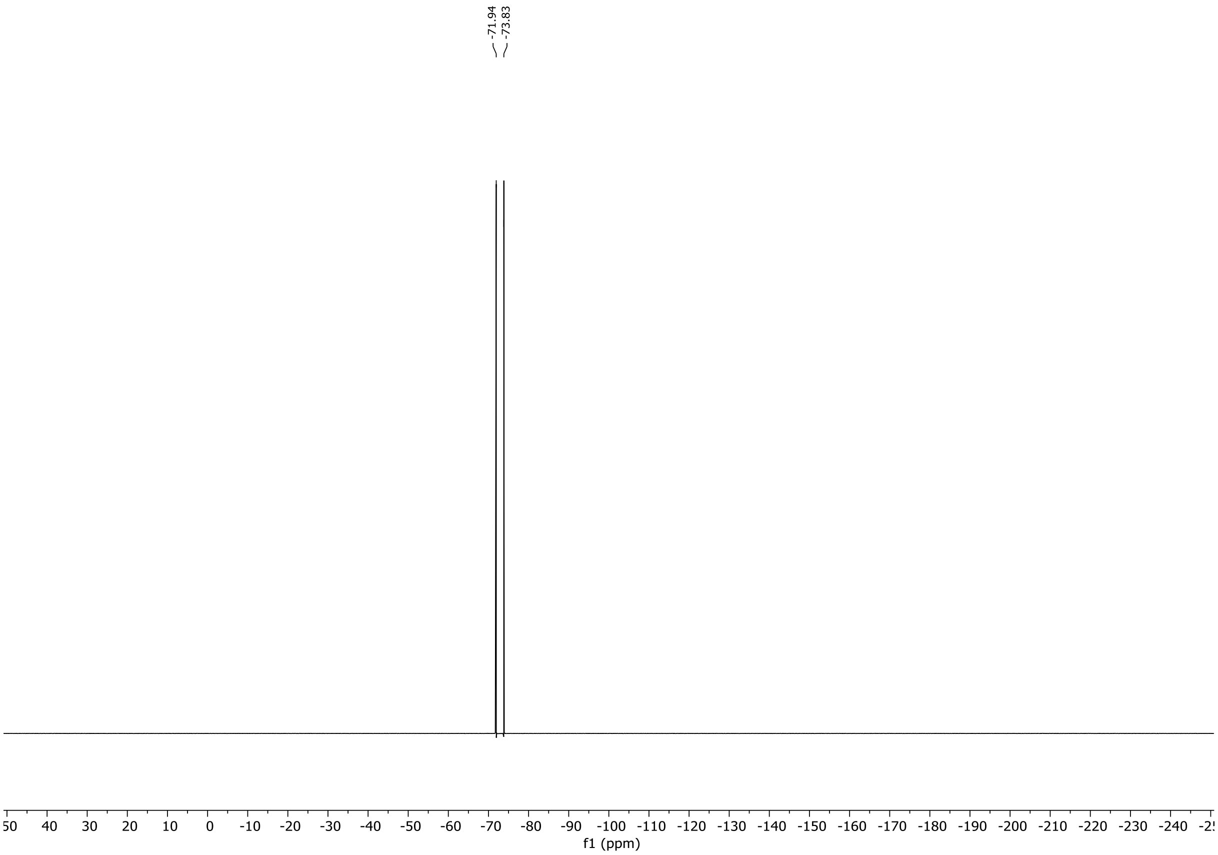

^1H NMR (400 MHz, $(CD_3)_2SO$) of compound **49**:

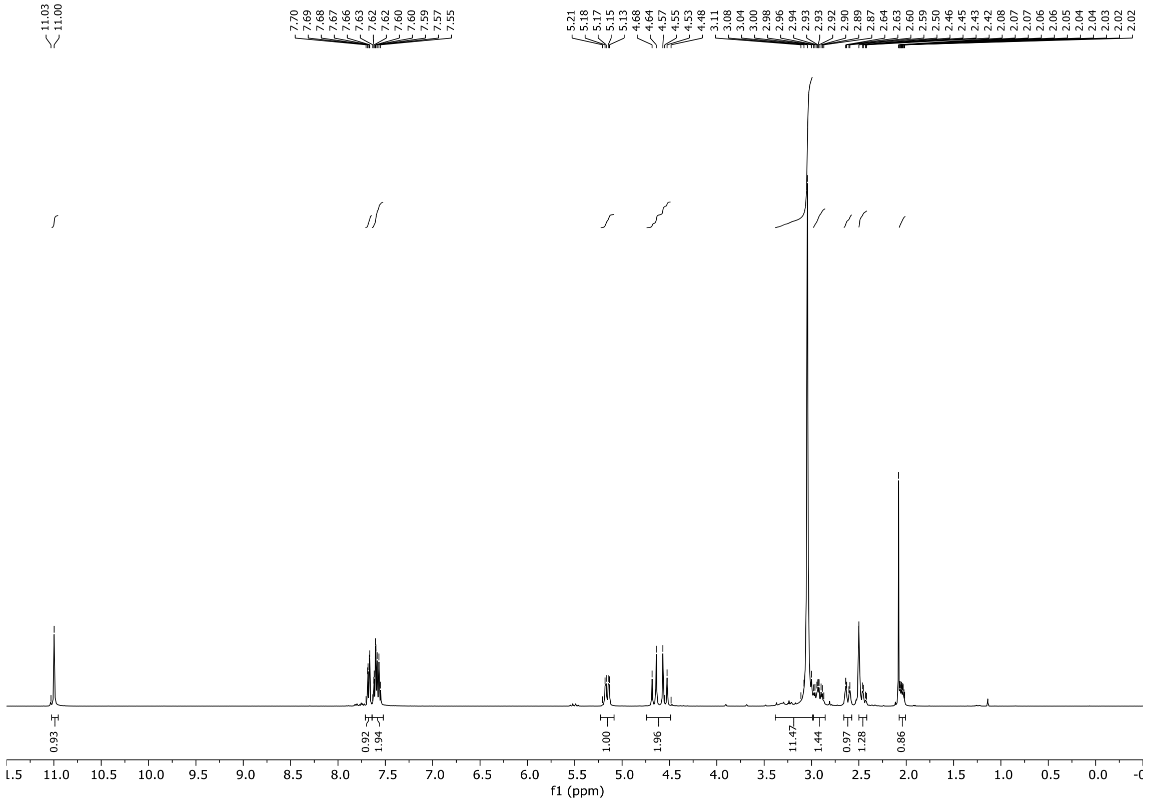

C NMR (101 MHz, $(CD_3)_2SO$) of compound **49**:

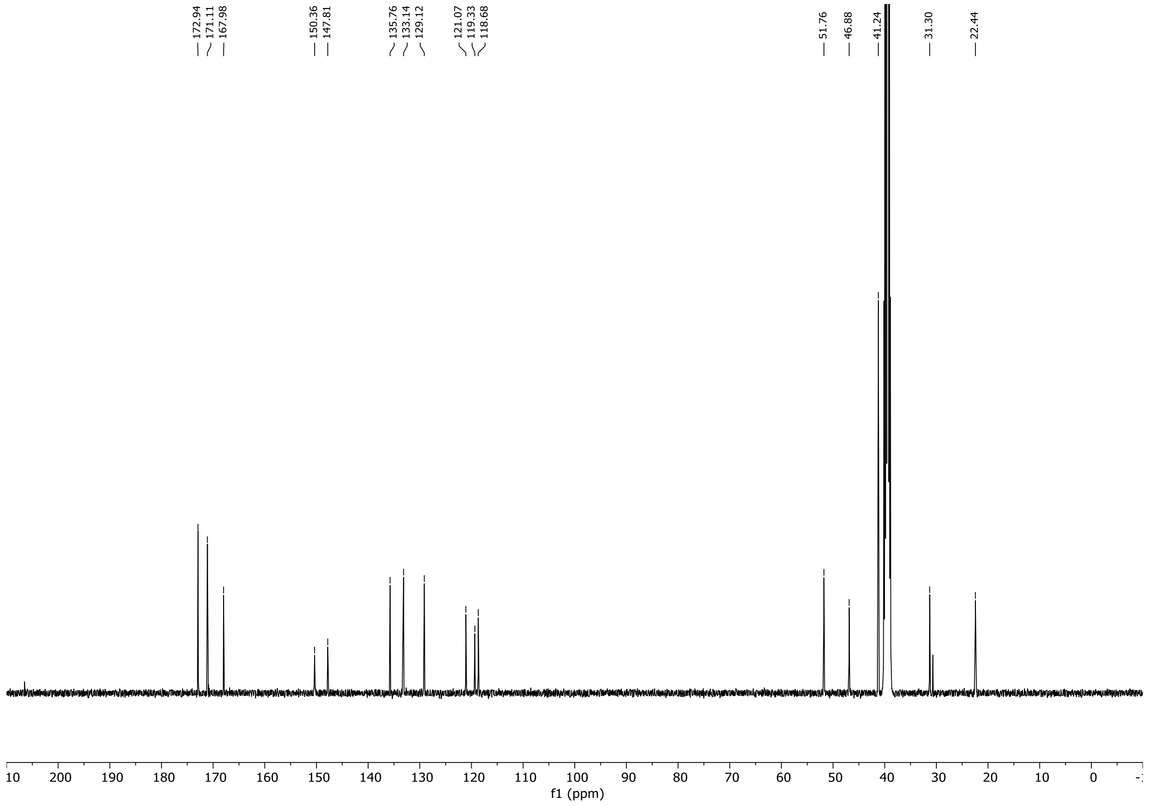

HSQC (400/101 MHz, $(CD_3)_2CO$) of compound **49**:

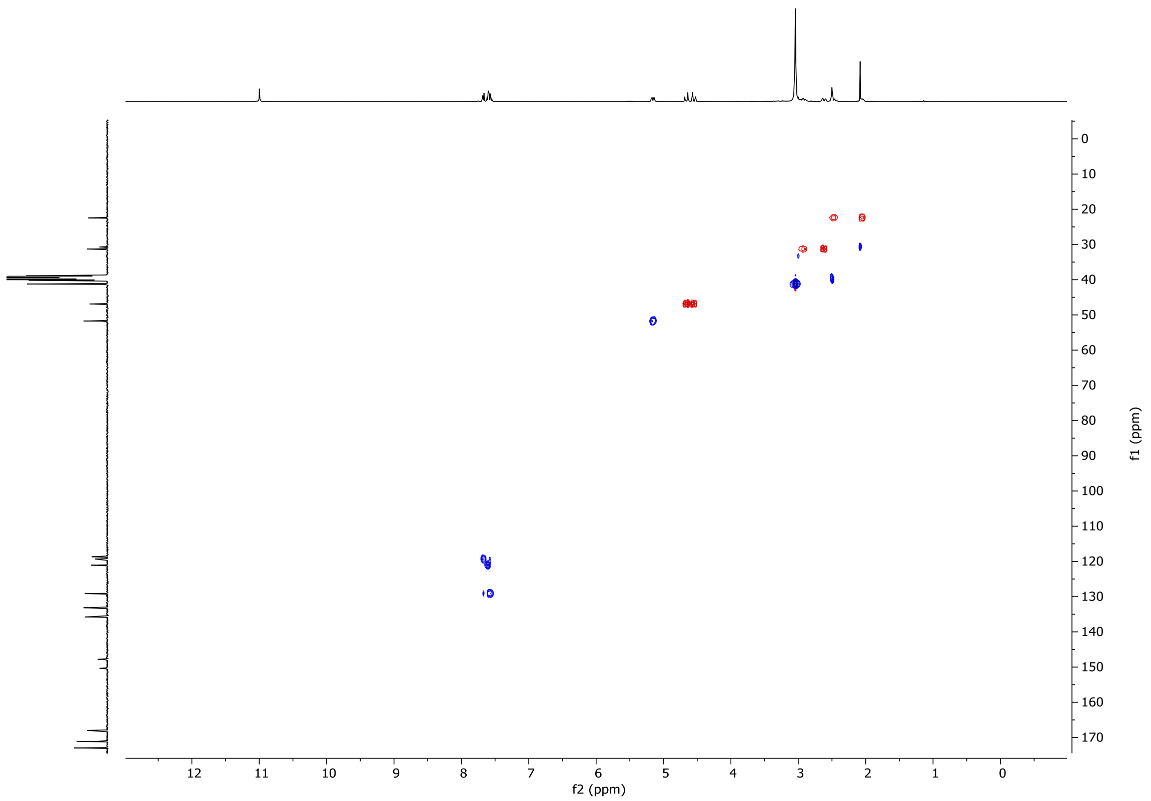

HMBC (400/101 MHz, $(CD_3)_2CO$) of compound **49**:

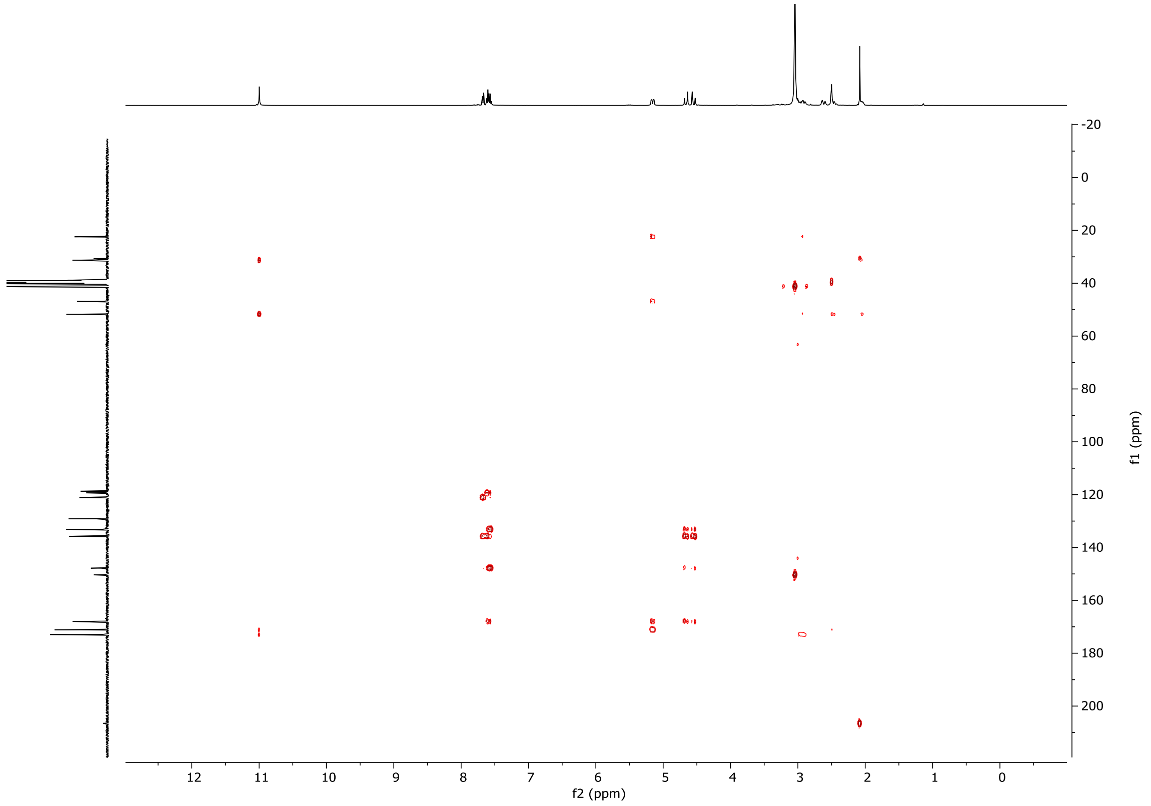

COSY (400 MHz, $(CD_3)_2CO$) of compound **49**:

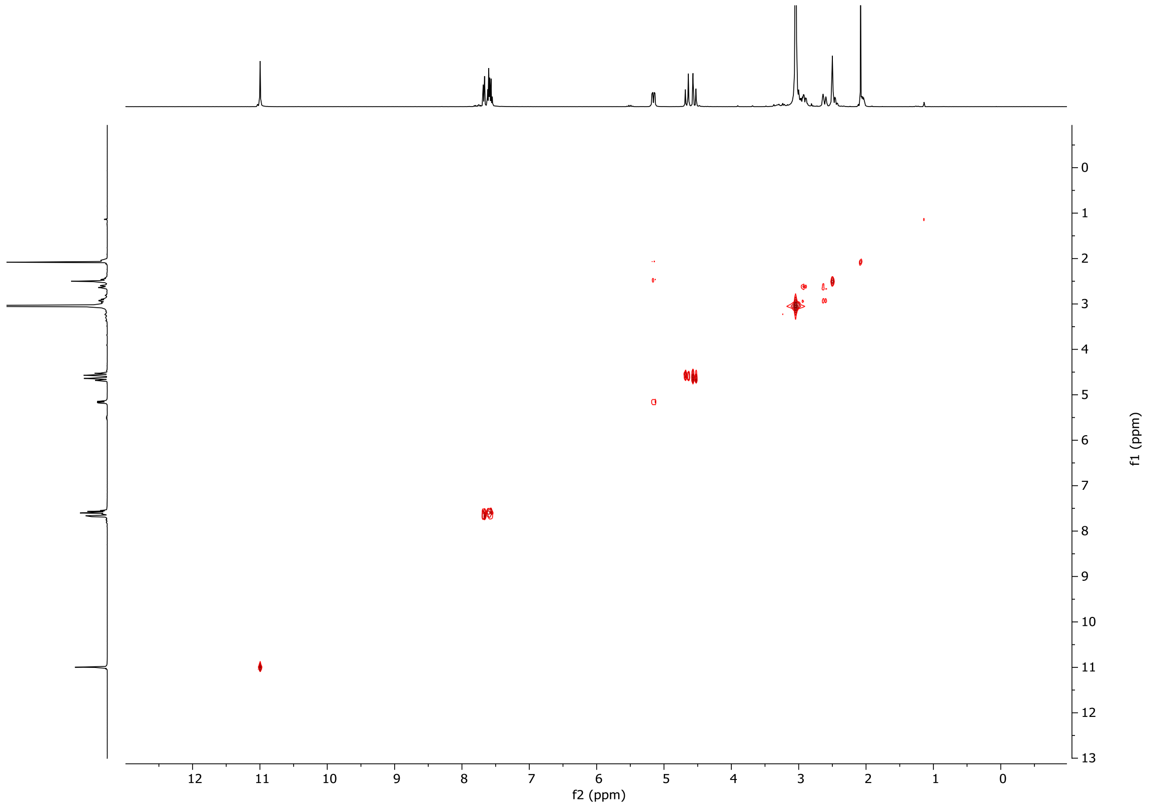

^{1}H NMR (400 MHz, $(CD_3)_2CO$) of compound **(NMe$_2$)$_2$-arylazopyrazole photoswitch**:

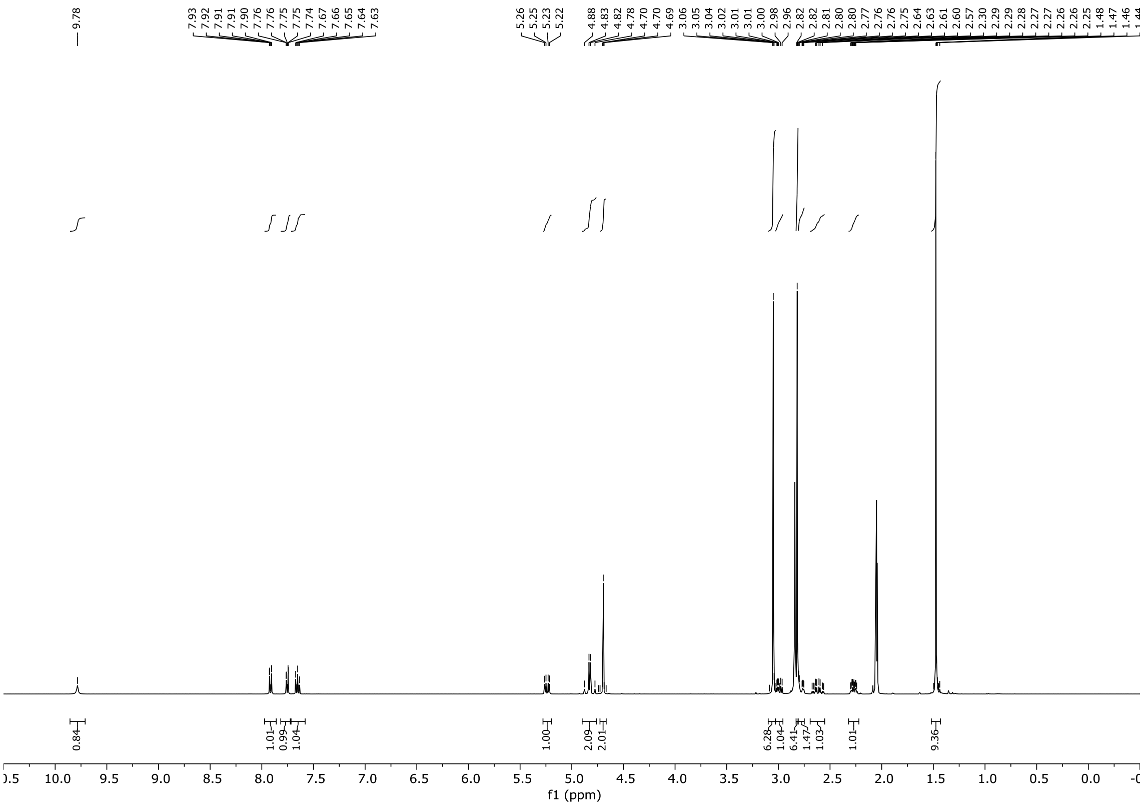

^{13}C NMR (101 MHz, $(CD_3)_2CO$) of compound **(NMe$_2$)$_2$-arylazopyrazole photoswitch**:

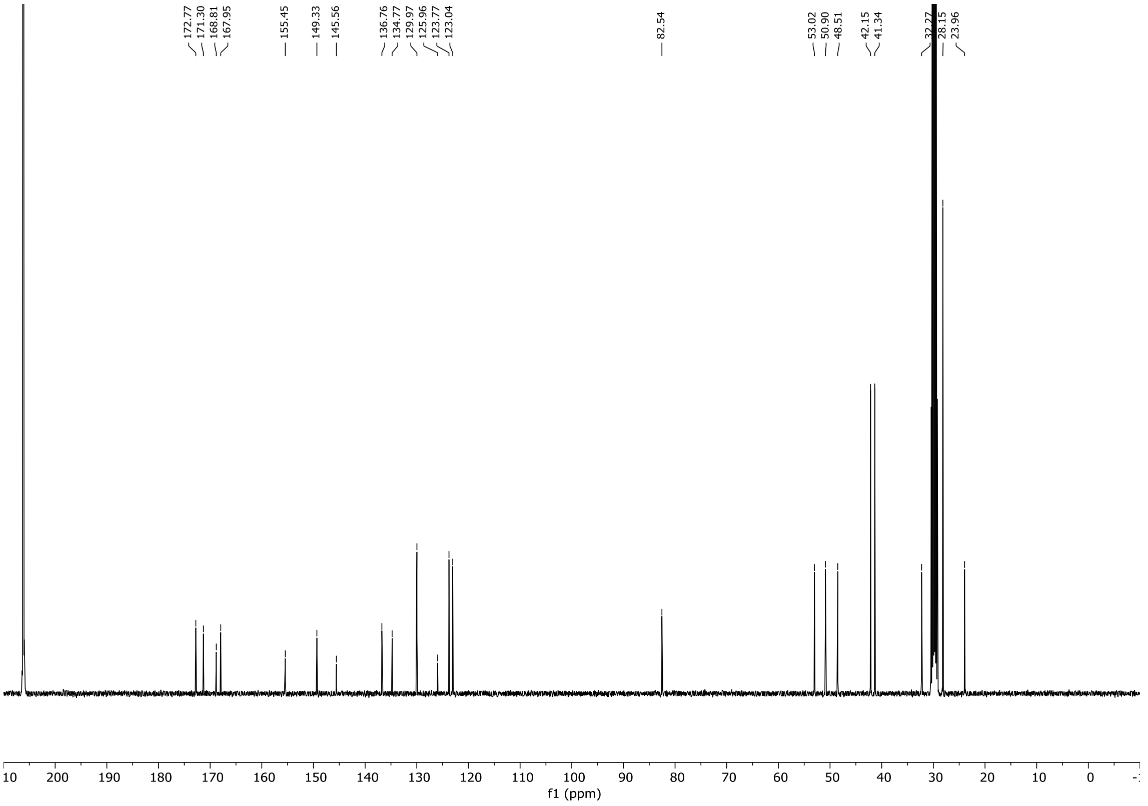

HSQC (400/101 MHz, $(CD_3)_2CO$) of compound **(NMe$_2$)$_2$-arylazopyrazole photoswitch**:

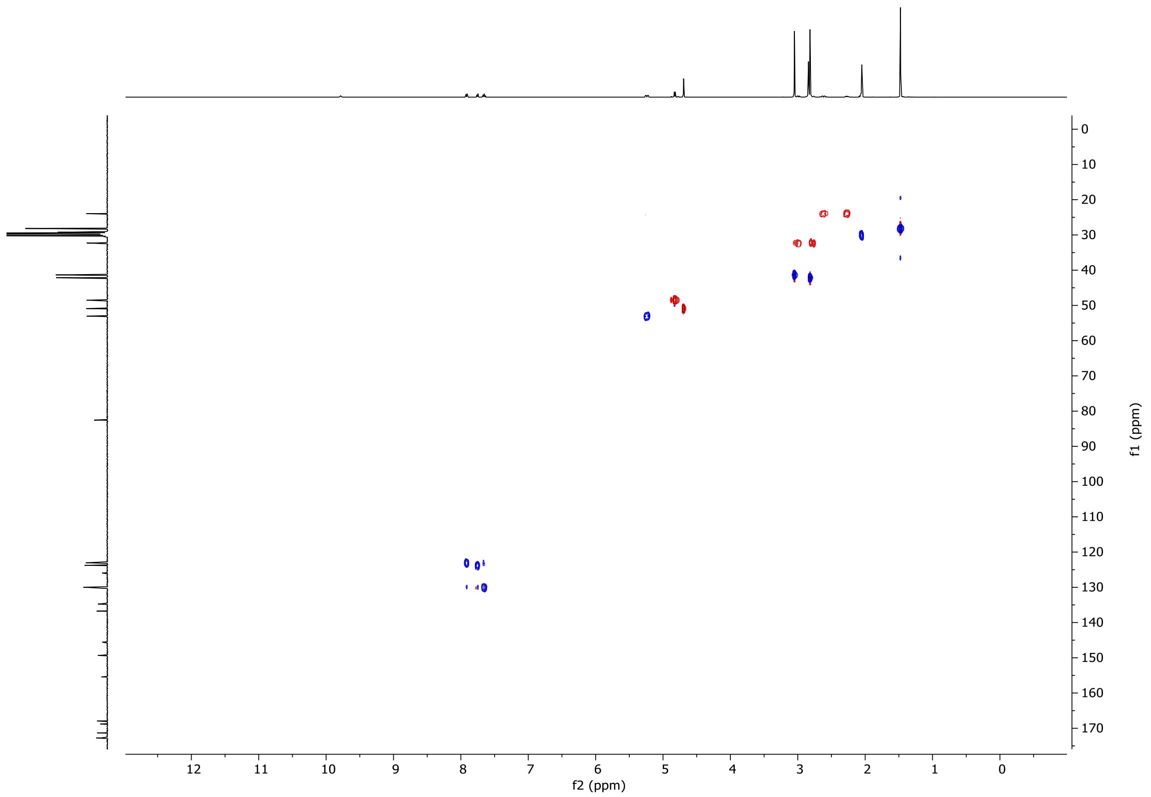

HMBC (400/101 MHz, $(CD_3)_2CO$) of compound **(NMe$_2$)$_2$-arylazopyrazole photoswitch**:

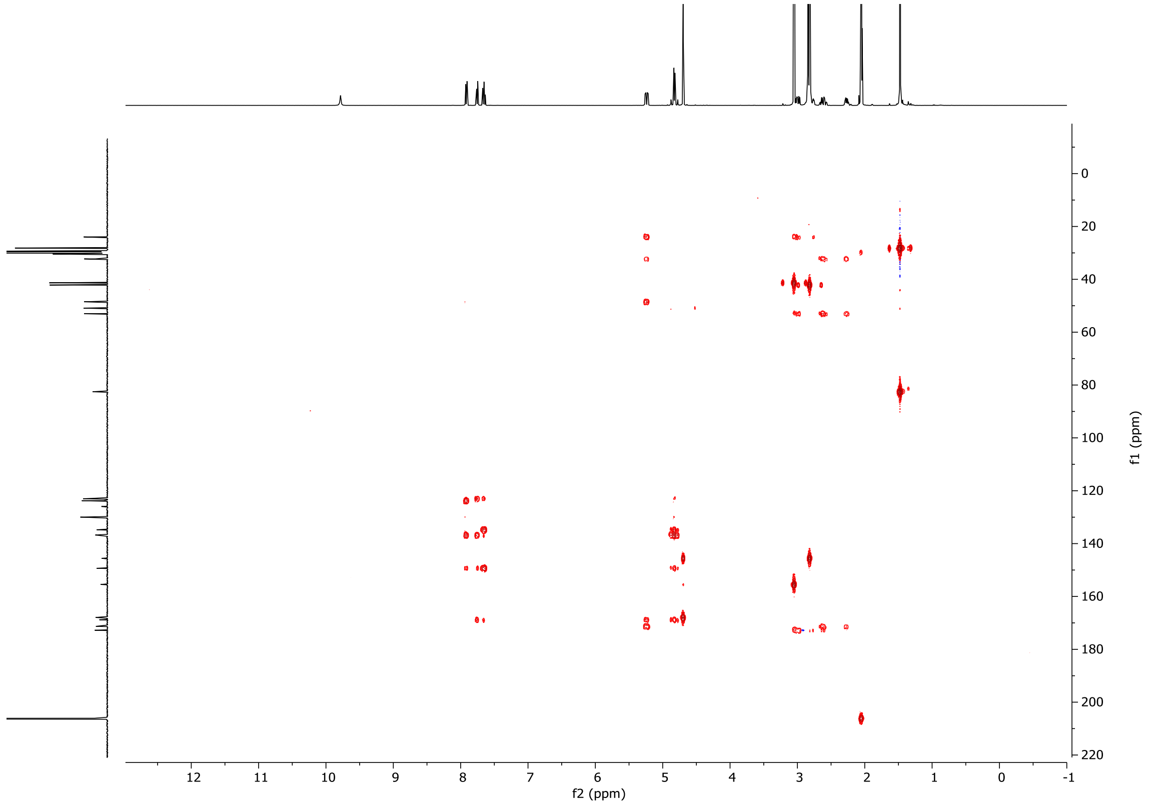

COSY (400 MHz, $(CD_3)_2CO$) of compound **$(NMe_2)_2$-arylazopyrazole photoswitch**:

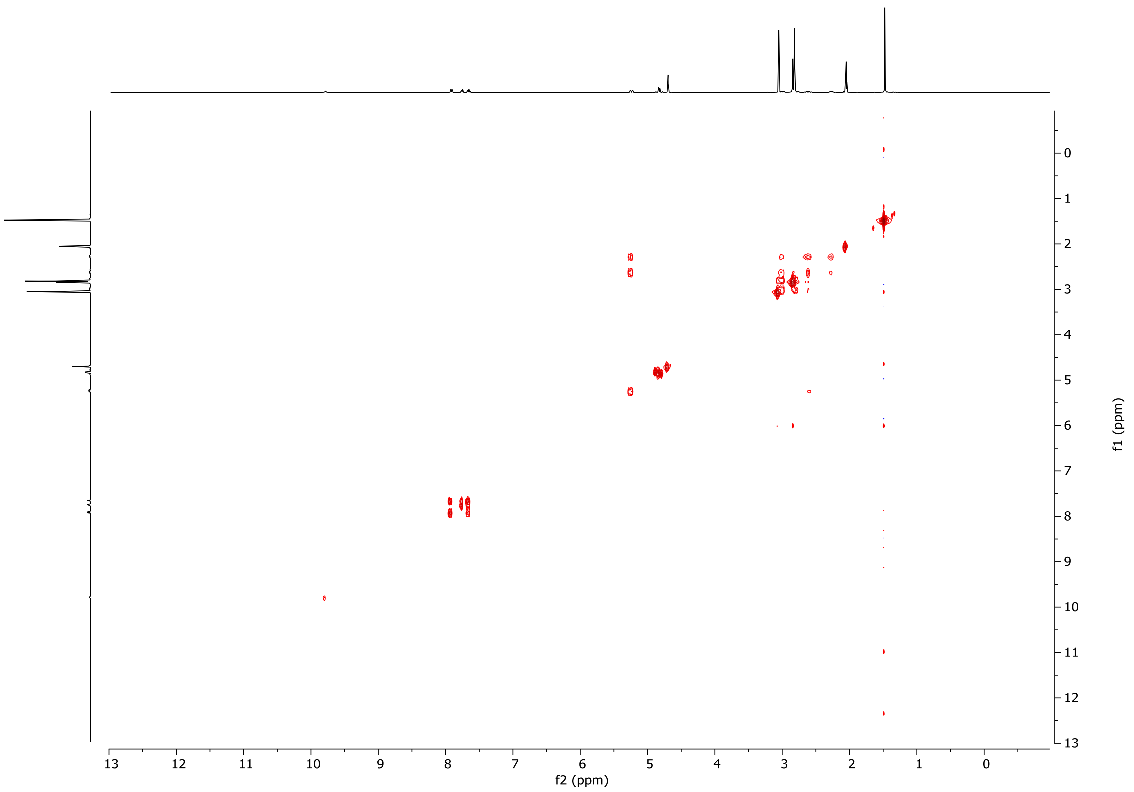

Supplement: Supplementary file 1 — Appendix 01 (PDF) [file pnas.2528760123.sapp.pdf]
